# Supplementary material for: Ligand-enabled ruthenium-catalyzed meta-C−H alkylation of (hetero)aromatic carboxylic acids
Source: Nat Commun. 2024 Jul 2;15:5552. doi: 10.1038/s41467-024-49362-2 (PMC11219896; doi:10.1038/s41467-024-49362-2)
Supplement: Supplementary file 1 — Supplementary Information [file 41467_2024_49362_MOESM1_ESM.pdf]

Supplementary Information

Ligand-Enabled Ruthenium-Catalyzed *meta*-C−H Alkylation of (hetero)Aromatic Carboxylic Acids

Xianglin Luo[1], Peichao Hou[1], Jiayi Shen[1], Yifeng Kuang[1], Fengchao Sun[1], Huanfeng Jiang[1], Lukas J. Goοßen[2]*, Liangbin Huang[1]*

[1]*Key Laboratory of Functional Molecular Engineering of Guangdong Province, State Key Laboratory of Pulp & Paper Engineering, School of Chemistry and Chemical Engineering, South China University of Technology, Guangzhou 510640, People's Republic of China*

[2]*Ruhr-Universität Bochum Lehrstuhl für Organische Chemie, Universitätsstraße 150, 44801 (Bochum)*

*Email: lukas.goossen@ruhr-uni-bochum.de; huanglb@scut.edu.cn

Table of Contents

Table of Contents 1

I. Supplementary Methods 2

1.1. General Experimental Information 2

1.2. Experimental Procedure for the Preparation of Tertiary Alkyl Bromide 4

1.3. Various Radical Precursors Screening for Carboxylate Directed *Meta*-C–H Functionalization under Ruthenium-Catalyzed σ-Bond Activation Conditions 8

1.4. Optimization of Reaction Conditions 10

1.5. General Procedures for Carboxylic Acid Directed *Meta*-Alkylation 19

1.6. Analysis Data for the Products 21

1.7. Control and Mechanistic Studies 49

1.8. Gram-Scale Reaction and Synthetic Application 60

1.9. Single Crystal Structure and Data 66

II. ^{1}H NMR, ^{13}C NMR and ^{19}F NMR Spectrum of All Products 80

III. Supplementary References 228

I. Supplementary Methods

1.1. General Experimental Information

Reagents

All compounds were used as received unless otherwise noted.

Metals: All metal catalysts were stored and handled in a nitrogen-filled glove box. Ag_2CO_3, AgTFA, AgOTf, $AgNTf_2$, AgF_6Sb, $RuCl_3$, $Ru(PPh_3)_4Cl_2$, $Ru_3(CO)_{12}$ and $[Ru(p\text{-cym})Cl_2]_2$ were purchased and used as received.

Additives: Lithium phosphate, sodium chloride, potassium chloride, tetrabutylammonium bromide, zinc acetate and zinc trifluoromethanesulfonate were purchased from Energy Chemical, lithium bromide, potassium bromide, magnesium acetate, iron(III)trifluoromethanesulfonate, D-camphorsulfonic acid (D-CSA) and lithium chloride were purchased from Bidepharm. Indium(III)trifluoromethanesulfonate and tin(III) trifluoromethanesulfonate were purchased from Shaoyuan.

Oxidant: Sodium persulfate was purchased from Innochem and (diacetoxyiodo)benzene was purchased from Bidepharm.

Other reagent: Di-tert-butyl peroxide (DTBP), tetrabutylammonium acetate (TBA-OAc), *N*-bromosuccinimide (NBS), 2-bromo-2-methylpropionyl bromide and benzenesulfonyl chloride were purchased from Innochem. triethylamine (NEt_3) and benzoylformic acid was purchased from Bidepharm. Corresponding alcohol or amine were purchased from Bidepharm or Energy Chemical.

Bases: Potassium carbonate, cesium carbonate, cesium acetate, potassium bicarbonate and sodium bicarbonate were purchased from Bidepharm, sodium carbonate, sodium acetate, potassium acetate, potassium phosphate, dipotassium hydrogen phosphate and lithium acetate were purchased from Energy Chemical.

Solvents: Toluene, 1,4-dioxane, 1,2-dichloroethane (DCE), tetrahydrofuran (THF), hexane, acetonitrile, *N*-methylpyrrolidone (NMP), 2,2,2-trifluoroethanol (TFA), *N*,*N*-dimethylformamide (DMF), Cyclopentyl methyl ether (CPME), acetic acid, 1,2-ethanediol, dichloromethane, chlorobenzene and tert-butyl methyl ether (TBME) were purchased from Energy Chemical. 1,1,1,3,3,3-hexafluoro-2-propanol (HFIP) and chlorobenzene were purchased from Bidepharm. tert-butanol (^{t}BuOH), acetic anhydride and methyl-tert-butyl ether were purchased from Innochem.

Ligands: 1-Adamantanoic acid, 2,4,6-trimethylbenzoic acid, 1,1'-binaphthyl-2,2'-diyl hydrogenphosphate (BNDHP), dicyclopentadiene iron and Piv-val-OH were purchased from Energy Chemical, **L1**-**L7, L9-L19** is purchased directly from Energy Chemical or Bidepharm or Laajoo. **L8** was synthesized as described in the corresponding literature[1].

Aromatic carboxylic acids: All aromatic carboxylic acids were purchased from Energy Chemical and Bidepharm.

Alkyl halides: 2-Bromo-2-methylpropane, 1-bromobutane and **1p**-**1q**, **1w** and **1ae-1ag** were purchased from Energy Chemical, other alkyl halides were prepared according to reported procedures[2-7].

NMR spectroscopy

^{1}H Nuclear magnetic resonance (NMR) spectroscopic chemical shifts were reported in ppm and referenced to TMS (tetramethylsilane) in $CDCl_3$ (δ = 0 ppm) or to the residual solvent peak for $CDCl_3$ (δ = 7.26 ppm). For ^{13}C NMR chemical shifts, the residual solvent peak ($CDCl_3$, δ = 77.00 ppm) was used as reference. NMR spectra were recorded on Avance Bruker NMR spectrometers operating at either 400 MHz or 500 MHz and data analysis was performed using the MestReNova software. Chemical shifts were reported in parts per million (ppm) and multiplicities were indicated by s (singlet), d (doublet), t (triplet), q (quartet), m (multiplet) and br (broad). Coupling constants (J) were reported in Hertz.

Gas Chromatography

GC analysis was performed on an Agilent 7890B GC equipped with HP-5 columns (30 m × 320 μm × 0.25 μm), FID detectors, and hydrogen as the carrier gas. A sample volume of 1 μL was injected at a temperature of 250 °C and a split ratio of 15:1. The initial inlet pressure was 2.7 psi but varied as the column flow was heated to a constant 1 mL/min for the duration of the run. The initial oven temperature of 60 °C was heated for 0 min followed by a temperature ramp of 50 °C/min up to 300 °C. The temperature was heated to 300 °C for 6 min. The total run time was 10.8 min and the FID temperature was 300 °C.

GC/MS analysis

GC/MS analyses were performed on a Shimadzu GCMS-QP2010SE equipped with an RTX-5MS column (30 m × 0.25 mm × 0.25 μm) with a quadrupole mass analyzer using helium as the carrier gas. The analytical method used in all cases was a 5 μL sample injection, an injection temperature of 250 ºC, and no split ratio. The initial inlet pressure was 7.8 psi, but varied as the column flow was heated

to a constant 1.7 mL/min for the duration of the run. The interface temperature was heated to 250 ºC, and the ion source (EI+, 30 eV) was heated to 250 ºC. The initial oven temperature was heated to 50 ºC for 1 min with the detector off, followed by a temperature ramp to 250 ºC at 30 ºC/min with the detector on. The temperature was heated at 250 ºC for 5 min, then to 280 ºC and heated for 11 min. The total run time was 25.17 min.

High Resolution Mass Spectrometry

HRMS spectra were acquired using an Agilent 6210 ESI/TOF mass spectrometer and a Thermo MAT 95XP double focusing magnetic sector analyzer (EI, 70 eV).

Thin layer/column chromatography

Thin layer chromatography was performed on TLC Silica Gel 60 F254 plates. Visualization was performed with potassium permanganate after inspection under UV light. Flash chromatography was performed using silica gel 60, particle size 0.040-0.063 mm using standard flash techniques.

1.2. Experimental Procedure for the Preparation of Tertiary Alkyl Bromide

1a-1b[2], **1f**[3], **1h**[4], **1r-1s**[4], **1i**[5], **1k-1m**[5], **1u**[5], **1ah-1ai**[5], **1ak**[5], **1am-1an**[5], **1y-1z**[6], **1ab**[7] were prepared according to the previous literature.

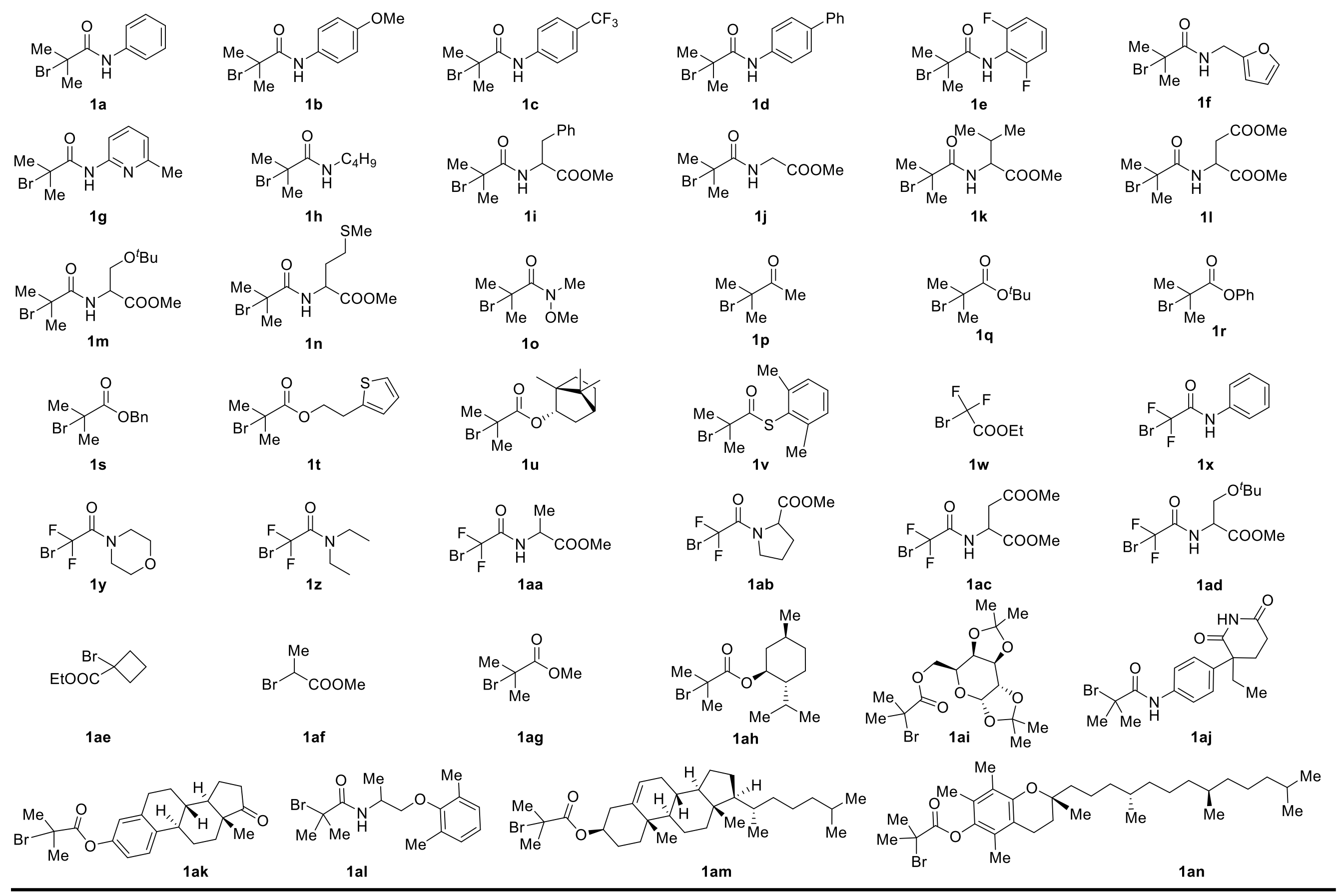

Typical Procedure I: Synthesis of Tertiary Alkyl Bromide

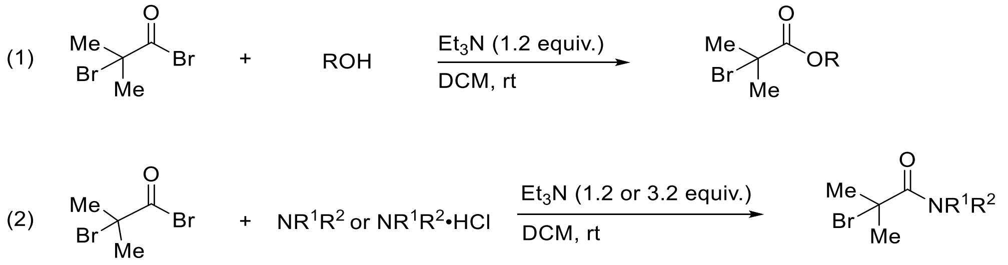

To a solution of alcohol or amine or amine hydrochloride (5 mmol, 1.0 equiv.) and Et_3N (1.2 equiv. or 3.2 equiv.) in DCM (30 mL) in an ice bath, 2-bromo-2-methylpropionyl bromide (1.2 equiv.) was added dropwise over 5 min. After completion of the addition, the reaction was allowed to return to room temperature and stirred overnight. The reaction mixture was concentrated and partitioned between EtOAc (100 mL) and H_2O (100 mL). The organic layer was extracted, dried over magnesium sulfate and concentrated in vacuo and purified by column chromatography to give the corresponding products.

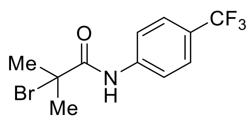

1c

2-Bromo-2-methyl-*N*-(4-(trifluoromethyl)phenyl)propenamide (1c)

According to the **typical procedure I** (1), 4-(trifluoromethyl)aniline (805 mg, 5 mmol), Et_3N (6 mmol), 2-bromo-2-methylpropanoyl bromide (709 uL, 6 mmol), DCM (30 mL) were used. The product **1c** (1.1 g, 71% yield) was obtained. **Physical state**: white solid; **MP** = 105 – 107 ºC; **^{1}H NMR** (500 MHz, $CDCl_3$) δ 8.51 (s, 1H), 7.58 (d, *J* = 8.5 Hz, 2H), 7.49 (d, *J* = 8.5 Hz, 2H), 1.95 (s, 6H); **^{13}C NMR** (125 MHz, $CDCl_3$) δ 170.3 (C_q), 140.4 (C_q), 126.6 (q, $J_{C\text{-}F}$ = 32.5 Hz, C_q), 126.2 (q, $J_{C\text{-}F}$ = 3.8 Hz, CH), 124.0 (q, $J_{C\text{-}F}$ = 270 Hz, C_q),

119.6 (CH), 62.4 (C_q), 32.3 (CH_3); **^{19}F NMR** (471 MHz, $CDCl_3$) δ -62.16; **ESI-HRMS** (m/z): $[M+H]^+$calcd for $C_{11}H_{12}BrF_3NO$, 310.0049, found: 310.0041.

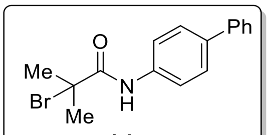

1d

N-([1,1'-Biphenyl]-4-yl)-2-bromo-2-methylpropanamide (1d)

According to the **typical procedure I** (1), [1,1'-biphenyl]-4-amine (845 mg, 5 mmol), Et_3N (6 mmol), 2-bromo-2-methylpropanoyl bromide (709 uL, 6 mmol), DCM (30 mL) were used. The product **1d** (1.2 g, 75% yield) was obtained. **Physical state**: white solid; **MP** = 98 – 100 ºC; **^{1}H NMR** (500 MHz, $CDCl_3$) δ 8.44 (s, 1H), 7.51 (d, J = 8.7 Hz, 2H), 7.48 – 7.43 (m, 4H), 7.31 (t, J = 7.7 Hz, 2H), 7.22 (t, J = 7.4 Hz, 1H), 1.95 (s, 6H); **^{13}C NMR** (125 MHz, $CDCl_3$) δ 170.0 (C_q), 140.2 (C_q), 137.6 (C_q), 136.5 (C_q), 128.7 (CH), 127.5 (CH), 127.1 (CH), 126.7 (CH), 120.3 (CH), 62.8 (C_q), 32.4 (CH_3); **ESI-HRMS** (m/z): $[M+H]^+$calcd for $C_{16}H_{17}BrNO$, 318.0488, found: 318.0483.

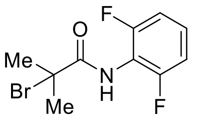

1e

2-Bromo-N-(2,6-difluorophenyl)-2-methylpropanamide (1e)

According to the **typical procedure I** (1), 2,6-difluoroaniline (645 mg, 5 mmol), Et_3N (6 mmol), 2-bromo-2-methylpropanoyl bromide (709 uL, 6 mmol), DCM (30 mL) were used. The product **1e** (1.1 g, 78% yield) was obtained. **Physical state**: white solid; **MP** = 96 – 98 ºC; **^{1}H NMR** (500 MHz, $CDCl_3$) δ 8.06 (s, 1H), 7.26 – 7.19 (m, 1H), 6.95 (t, J = 8.1 Hz, 2H), 2.05 (s, 6H); **^{13}C NMR** (125 MHz, $CDCl_3$) δ 170.7 (C_q), 158.8 (d, $J_{C\text{-}F}$ = 3.8 Hz, C_q), 156.8 (d, $J_{C\text{-}F}$ = 5.0 Hz, C_q), 128.0 (t, $J_{C\text{-}F}$ = 10.0 Hz, C_q), 113.6 (t, $J_{C\text{-}F}$ = 16.3 Hz, CH), 111.7 (d, $J_{C\text{-}F}$ = 3.8 Hz, CH), 111.5 (d, $J_{C\text{-}F}$ = 5.0 Hz, CH), 61.3 (C_q), 32.2 (CH_3); **^{19}F NMR** (471 MHz, $CDCl_3$) δ -118.10 (t, J = 6.5 Hz); **ESI-HRMS** (m/z): $[M+H]^+$calcd for $C_{10}H_{11}BrF_2NO$, 277.9987, found: 277.9983.

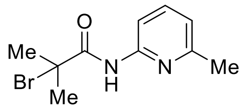

1g

2-Bromo-2-methyl-N-(6-methylpyridin-2-yl)propenamide (1g)

According to the **typical procedure I** (1), 6-methylpyridin-2-amine (540 mg, 5 mmol), Et_3N (6 mmol), 2-bromo-2-methylpropanoyl bromide (709 uL, 6 mmol), DCM (30 mL) were used. The product **1g** (1.08 g, 84% yield) was obtained. **Physical state**: colorless liquid; **^{1}H NMR** (500 MHz, $CDCl_3$) δ 8.80 (s, 1H), 7.95 (d, J = 8.2 Hz, 1H), 7.58 (t, J = 7.9 Hz, 1H), 6.89 (d, J = 7.4 Hz, 1H), 2.44 (s, 3H), 2.01 (s, 6H); **^{13}C NMR** (125 MHz, $CDCl_3$) δ 170.2 (C_q), 157.0 (C_q), 150.2 (C_q), 138.5 (CH), 119.7 (CH), 110.3 (CH), 61.0 (C_q), 32.0 (CH_3), 23.9 (CH_3); **ESI-HRMS** (m/z): $[M+H]^+$calcd for $C_{10}H_{14}BrN_2O$, 257.0284, found: 257.0278.

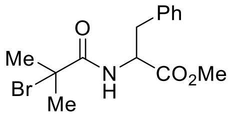

1i

Methyl-(2-bromo-2-methylpropanoyl)phenylalaninate (1i)

According to the **typical procedure I** (1), methyl phenylalaninate (895 mg, 5 mmol), Et_3N (16 mmol), 2-bromo-2-methylpropanoyl bromide (709 uL, 6 mmol), DCM (30 mL) were used. The product **1i** (1.2 g, 73% yield) was obtained. **Physical state**: white solid; **^{1}H NMR** (500 MHz, $CDCl_3$) δ 7.29 (t, J = 7.2 Hz, 2H), 7.24 (t, J = 7.2 Hz, 1H), 7.13 (d, J = 7.0 Hz, 2H), 7.07 (d, J = 7.0 Hz, 1H), 4.81 (q, J = 6.1 Hz, 1H), 3.73 (s, 3H), 3.20 (dd, J = 13.9, 5.6 Hz, 1H), 3.11 (dd, J = 13.9, 6.3 Hz, 1H), 1.91 (s, 3H), 1.86 (s, 3H); **^{13}C NMR** (125 MHz, $CDCl_3$) δ 171.32 (C_q), 171.31 (C_q), 135.4 (C_q), 129.2 (CH), 128.4 (CH), 127.1 (CH), 61.5 (CH), 53.7 (C_q), 52.2 (CH_3), 37.5 (CH_2), 32.1 (CH_3), 32.0 (CH_3); **ESI-HRMS** (m/z): $[M+H]^+$calcd for $C_{14}H_{19}BrNO_3$, 328.0543, found: 328.0538.

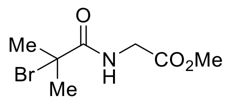

1j

Methyl-(2-bromo-2-methylpropanoyl)glycinate (1j)

According to the **typical procedure I** (1), methyl glycinate hydrochloride (625 mg, 5 mmol), Et_3N (16 mmol), 2-bromo-2-methylpropanoyl bromide (709 uL, 6 mmol), DCM (30 mL) were used. The product **1j** (782 mg, 66% yield) was obtained. **Physical state**: colorless liquid; **^{1}H NMR** (500 MHz, $CDCl_3$) δ 7.17 (s, 1H), 4.01 (d, *J* = 5.3 Hz, 2H), 3.74 (s, 3H), 1.94 (s, 6H); **^{13}C NMR** (125 MHz, $CDCl_3$) δ 172.3 (C_q), 169.8 (C_q), 61.6 (C_q), 52.3 (CH_3), 41.9 (CH_2), 32.3 (CH_3); **ESI-HRMS** (m/z): $[M+H]^+$calcd for $C_7H_{13}BrNO_3$, 238.0073, found: 238.0071.

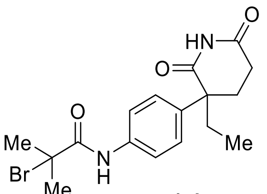

1aj

2-Bromo-*N*-(4-(3-ethyl-2,6-dioxopiperidin-3-yl)phenyl)-2-methylpropanamide (1aj)

According to the **typical procedure I** (1), 3-(4-aminophenyl)-3-ethylpiperidine-2,6-dione (1.16 g, 5 mmol), Et_3N (6 mmol), 2-bromo-2-methylpropanoyl bromide (709 uL, 6 mmol), DCM (30 mL) were used. The product **1aj** (1.48 g, 78% yield) was obtained. **Physical state**: white solid; **MP** = 180 – 182 ºC; **^{1}H NMR** (500 MHz, $CDCl_3$) δ 8.43 (d, *J* = 18.3 Hz, 2H), 7.49 (d, *J* = 8.3 Hz, 2H), 7.19 (d, *J* = 8.3 Hz, 2H), 2.55 – 2.50 (m, 1H), 2.36 – 2.27 (m, 2H), 2.18 – 2.11 (m, 1H), 1.97 (s, 6H), 1.87 – 1.81 (m, 1H), 1.21 – 1.17 (M, 1H), 0.79 (t, *J* = 7.4 Hz, 3H); **^{13}C NMR** (125 MHz, $CDCl_3$) δ 175.2 (C_q), 172.4 (C_q), 170.0 (C_q), 136.8 (C_q), 135.0 (C_q), 126.8 (CH), 120.3 (CH), 62.6 (C_q), 50.6 (C_q), 32.8 (CH_3), 32.3 (CH_2), 29.2 (CH_2), 26.9 (CH_2), 8.9 (CH_3); **ESI-HRMS** (m/z): $[M-H]^+$calcd for $C_{17}H_{20}BrN_2O_3$, 379.0663, found: 379.0662.

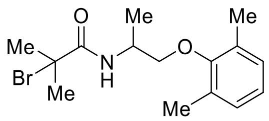

1al

2-Bromo-*N*-(1-(2,6-dimethylphenoxy)propan-2-yl)-2-methylpropanamide (1al)

According to the **typical procedure I** (1), 1-(2,6-dimethylphenoxy)propan-2-amine (895 mg, 5 mmol), Et_3N (16 mmol), 2-bromo-2-methylpropanoyl bromide (709 uL, 6 mmol), DCM (30 mL) were used. The product **1al** (1.32 g, 81% yield) was obtained. **Physical state**: white solid; **MP** = 62 – 64 ºC; **^{1}H NMR** (500 MHz, $CDCl_3$) δ 7.29 – 7.21 (m, 1H), 7.01 (d, *J* = 7.4 Hz, 2H), 6.93 (dd, *J* = 8.2, 6.7 Hz, 1H), 4.28 (ddt, *J* = 8.3, 6.8, 3.5 Hz, 1H), 3.83 (dd, *J* = 9.2, 3.8 Hz, 1H), 3.74 (dd, *J* = 9.2, 3.2 Hz, 1H), 2.29 (s, 6H), 1.99 (d, *J* = 1.8 Hz, 6H), 1.45 (d, *J* = 6.8 Hz, 3H); **^{13}C NMR** (125 MHz, $CDCl_3$) δ 171.4 (C_q), 154.7 (C_q), 130.7 (C_q), 128.9 (CH), 124.1 (CH), 73.4 (CH_2), 63.0 (C_q), 46.3 (CH), 32.6 (CH_3), 32.5 (CH_3), 17.4 (CH_3), 16.2 (CH_3); **ESI-HRMS** (m/z): $[M+H]^+$calcd for $C_{15}H_{23}BrNO_2$, 328.0907, found: 328.0900.

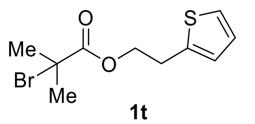

1t

2-(Thiophen-2-yl)ethyl 2-bromo-2-methylpropanoate (1t)

According to the **typical procedure I** (1), 2-(thiophen-2-yl)ethan-1-ol (640 mg, 5 mmol), Et_3N (6 mmol), 2-bromo-2-methylpropanoyl bromide (709 uL, 6 mmol), DCM (30 mL) were used. The product **1t** (1.08 g, 78% yield) was obtained. **Physical state**: colorless liquid; **^{1}H NMR** (400 MHz, $CDCl_3$) δ 7.17 (dd, *J* = 5.1, 1.2 Hz, 1H), 6.95 (dd, *J* = 5.1, 3.5 Hz, 1H), 6.92 – 6.87 (m, 1H), 4.39 (t, *J* = 6.6 Hz, 2H), 3.21 (t, *J* = 6.6 Hz, 2H), 1.93 (s, 6H); **^{13}C NMR** (100 MHz, $CDCl_3$) δ 171.4 (C_q), 139.4 (C_q), 126.8 (CH), 125.7 (CH), 124.0 (CH), 66.0

(CH_2), 55.6 (C_q), 30.6 (CH_3), 28.9 (CH_2).

Typical Procedure II: Synthesis of Tertiary Alkyl Bromide

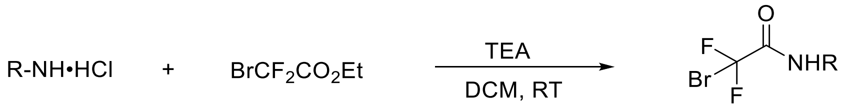

The procedure for the preparation of the compounds is as follows: alkylamine hydrochloride (6 mmol, 1.2 equiv.) was added to a dry 10 mL Schlenk flask and the flask was evacuated and refilled with N_2 three times. DCM (5 mL), ethyl bromodifluoroacetate (5 mmol, 1 equiv.) and TEA (7.5 mmol, 1.5 equiv.) were then added. The reaction was stirred overnight at room temperature. The reaction mixture was diluted with EtOAc, washed with 2M HCl (50 mL), aqueous $NaHCO_3$ and brine. The organic layer was dried over Na_2SO_4, filtered and concentrated. The residue was purified by silica gel chromatography to give the corresponding 1-bromo-1,1-difluoroacetamides.

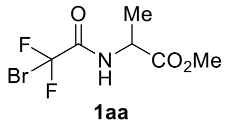

1aa

Methyl-(2-bromo-2,2-difluoroacetyl)alaninate (1aa)

According to the **typical procedure II**, L-Alanine methyl ester hydrochloride (834 mg, 6 mmol), TEA (7.5 mmol,1.12 g), ethyl bromodifluoroacetate (1.0 g, 5 mmol), DCM (5 mL) were used. The product **1aa** (912 mg, 70% yield) was obtained. **Physical state**: colorless liquid; **^{1}H NMR** (500 MHz, $CDCl_3$) δ 7.15 (s, 1H), 4.56 – 4.51 (m, 1H), 3.75 (s, 3H), 1.45 (d, *J* = 7.3 Hz, 3H); **^{13}C NMR** (125 MHz, $CDCl_3$) δ 171.9 (C_q), 159.3 (t, $J_{C\text{-}F}$ = 27.5 Hz, C_q), 111.3 (t, $J_{C\text{-}F}$ = 312.5 Hz, C_q), 52.8 (CH), 48.7 (CH_3), 17.4 (CH_3); **^{19}F NMR** (471 MHz, $CDCl_3$) δ -65.66; **ESI-HRMS** (m/z): $[M+H]^+$calcd for $C_6H_9F_2BrNO_3$, 259.9728, found: 259.9723.

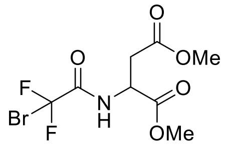

1ac

Dimethyl-(2-bromo-2,2-difluoroacetyl)aspartate (1ac)

According to the **typical procedure II**, dimethyl aspartate hydrochloride (1.2 g, 6 mmol), TEA (7.5 mmol,1.12 g), ethyl bromodifluoroacetate (1.0 g, 5 mmol), DCM (5 mL) were used. The product **1ac** (1.1 g, 71% yield) was obtained. Physical state: colorless liquid; **^{1}H NMR** (500 MHz, $CDCl_3$) δ 7.37 (d, *J* = 7.5 Hz, 1H), 4.80 (dt, *J* = 8.5, 4.4 Hz, 1H), 3.78 (s, 3H), 3.70 (s, 3H), 3.09 (dd, *J* = 17.5, 4.4 Hz, 1H), 2.90 (dd, *J* = 17.4, 4.6 Hz, 1H); **^{13}C NMR** (125 MHz, $CDCl_3$) δ 171.0 (C_q), 169.6 (C_q), 159.6 (t, $J_{C\text{-}F}$ = 28.8 Hz, C_q), 111.2 (t, $J_{C\text{-}F}$ = 313.8 Hz, C_q), 53.2 (CH), 52.3 (CH_3), 49.0 (CH_3), 35.1 (CH_2); **^{19}F NMR** (471 MHz, $CDCl_3$) δ -61.04 (d, *J* = 6.5 Hz); **ESI-HRMS** (m/z): $[M+H]^+$calcd for $C_8H_{11}BrF_2NO_5$, 317.9783, found: 317.9785.

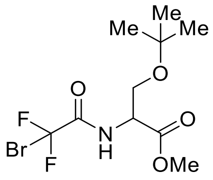

1ad

Methyl-*N*-(2-bromo-2,2-difluoroacetyl)-*O*-(tert-butyl)serinate (1ad)

According to the **typical procedure II**, methyl-*O*-(tert-butyl)serinate hydrochloride (1.3 g, 6 mmol), TEA (7.5 mmol,1.12 g), ethyl bromodifluoroacetate (1.0 g, 5 mmol), DCM (5 mL) were used. The product **1ad** (1.2 g, 72% yield) was obtained. Physical state: colorless liquid; **^{1}H NMR** (500 MHz, $CDCl_3$) δ 7.04 (s, 1H), 4.65 (dt, *J* = 8.2, 3.0 Hz, 1H), 3.84 (dd, *J* = 9.3, 2.7 Hz, 1H), 3.76 (s, 3H), 3.62 (dd, *J* = 9.3, 3.1 Hz, 1H), 1.12 (s, 9H); **^{13}C NMR** (125 MHz, $CDCl_3$) δ 169.4 (C_q), 159.5 (t, $J_{C\text{-}F}$ = 27.5 Hz, C_q), 111.4 (t, $J_{C\text{-}F}$ = 313.8 Hz, C_q), 73.8 (C_q), 61.1 (CH_2), 53.3 (CH), 52.7 (CH_3), 27.1 (CH_3); **^{19}F NMR** (471 MHz, $CDCl_3$) δ -60.85 (d, *J* = 28.2 Hz); **ESI-HRMS** (m/z): $[M+H]^+$calcd for $C_{10}H_{15}BrF_2NO_4$, 330.0158, found: 330.0161.

Typical Procedure III: Synthesis of Tertiary Alkyl Bromide

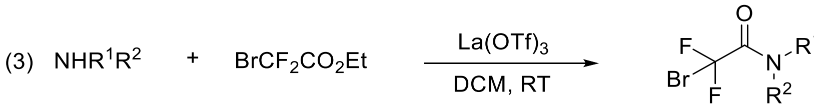

The procedure for the preparation of the compounds is as follows: a 10 mL tube equipped with a magnetic stirring bar was filled with La(OTf)$_3$ (5 mol%). The tube was backfilled with nitrogen, ethyl bromodifluoroacetate (1.2 equiv.), amine (1 equiv.) and DCM were added. The mixture was stirred at room temperature and monitored by TLC. The reaction mixture was diluted with EtOAc and washed with brine. The organic layer was dried over Na_2SO_4, filtered and concentrated. The residue was purified by silica gel chromatography to give the corresponding products.

1.3. Various Radical Precursors Screening for Carboxylate Directed *Meta*-C–H Functionalization under Ruthenium-Catalyzed σ-Bond Activation Conditions

Supplementary Figure 1. Carboxylate directed *meta*-C–H benzylation with toluene[8].

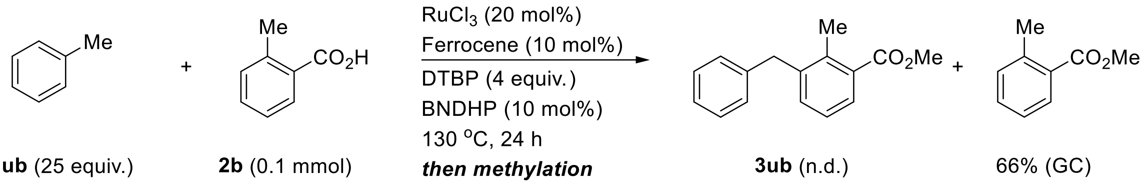

1ub (25 equiv.) **2b** (0.1 mmol) **3ub** (n.d.) 66% (GC)

Supplementary Figure 2. Carboxylate directed *meta*-C–H acylation with α-oxocarboxylic acids[9].

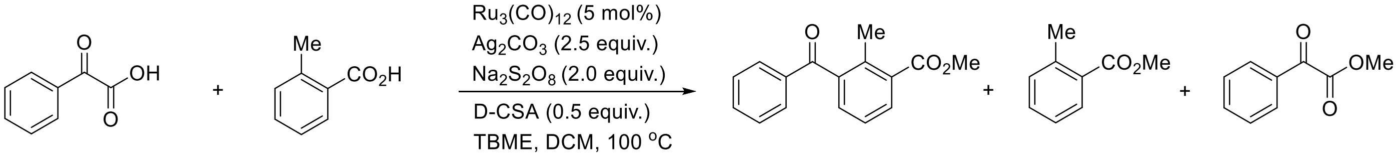

1vb (2.0 equiv.) **2b** (0.1 mmol) **3vb** (n.d.) 59% (GC) 27% (GC)

Supplementary Figure 3. Carboxylate directed *meta*-C–H sulfonation with *p*-toluenesulfonyl chloride[10].

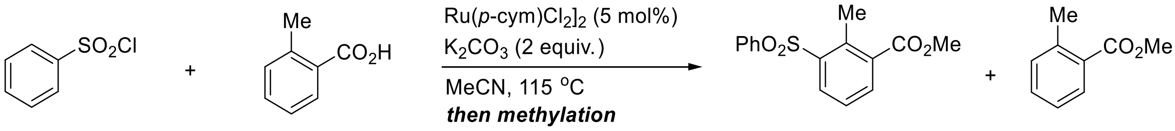

1wb (3.0 equiv.) **2b** (0.1 mmol) **3wb** (n.d.) 85% (GC)

Supplementary Figure 4. Carboxylate directed *meta*-C–H nitration with $Cu(NO_3)_2·3H_2O$[11].

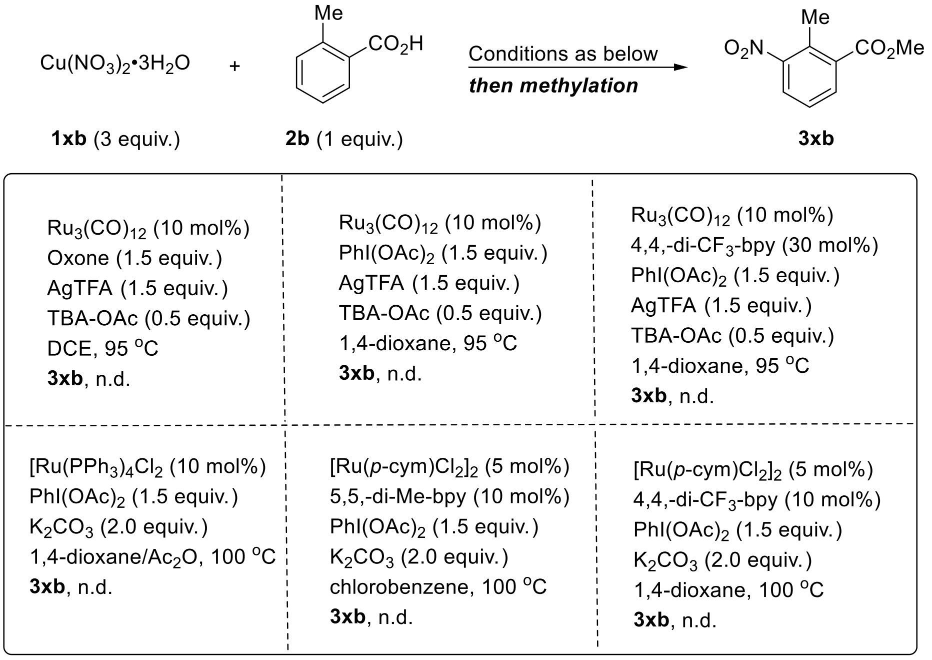

Supplementary Figure 5. Carboxylate directed *meta*-C–H bromination with TBATB[12] or NBS[13].

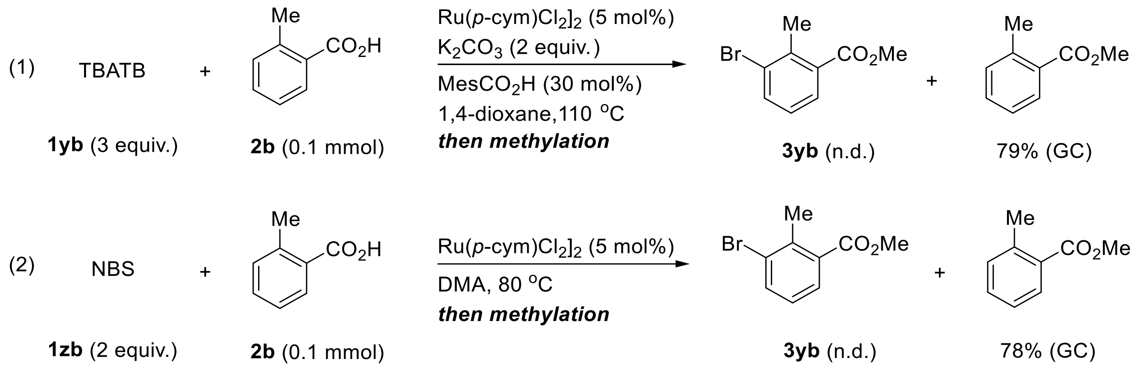

Supplementary Figure 6. Carboxylate directed *meta*-C–H alkylation with alkyl halides[6, 14-15].

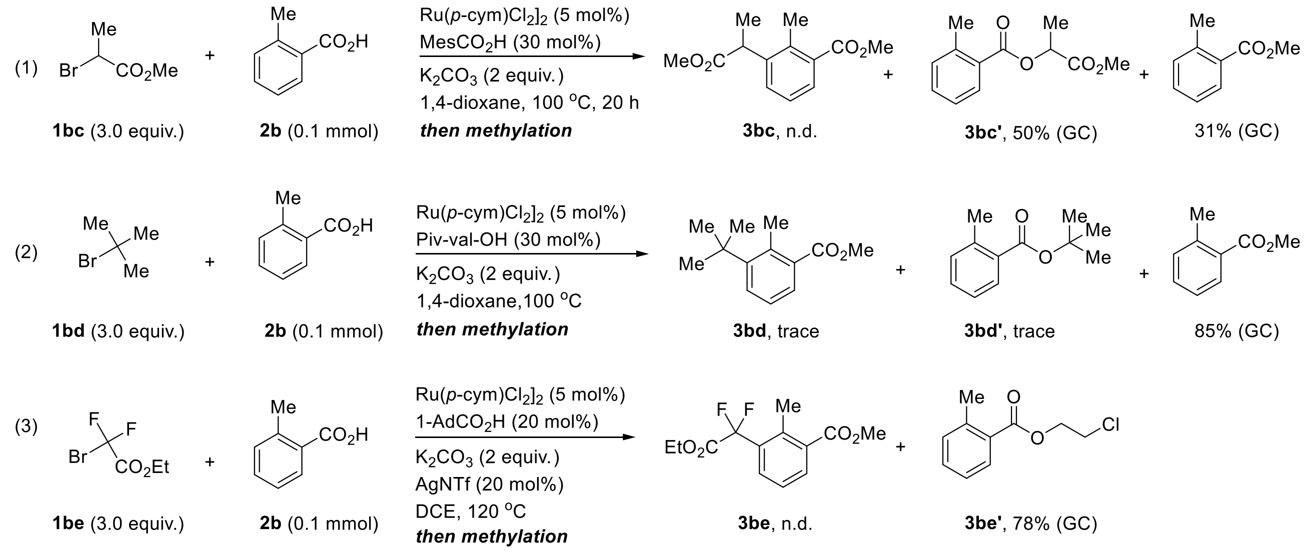

1.4. Optimization of Reaction Conditions

Supplementary Figure 7. Classical reaction conditions were tested for this carboxylate directed *meta*-C–H alkylation with **1a**[a].

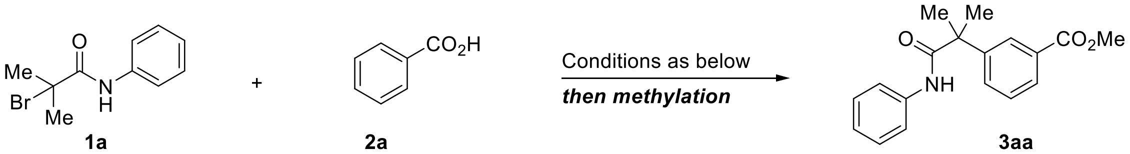

1a **2a** **3aa**

Lutz Ackermann, 2013[14]	Lutz Ackermann, 2015[15]	Lutz Ackermann, 2017[6]	Christopher G. Frost, 2015[16]
Ru(*p*-cym)Cl_2]$_2$ (5 mol%)	Ru(*p*-cym)Cl_2]$_2$ (5 mol%)	Ru(*p*-cym)Cl_2]$_2$ (5 mol%)	Ru(*p*-cym)Cl_2]$_2$ (5 mol%)
$MesCO_2H$ (30 mol%)	Piv-val-OH (30 mol%)	(4-$C_6H_4CF_3$)$_3$P (20 mol%)	KOAc (2 equiv.)
K_2CO_3 (2 equiv.)	K_2CO_3 (2 equiv.)	$MesCO_2H$ (20 mol%)	1,4-dioxane, 120 °C, 15 h
1,4-dioxane, 100 °C, 20 h	1,4-dioxane,100 °C, 20 h	Na_2CO_3 (2 equiv.)	**3aa**: n.d
**3aa**: n.d	**3aa**: n.d	1,4-dioxane, 60 °C, 18 h	
		**3aa**: n.d	

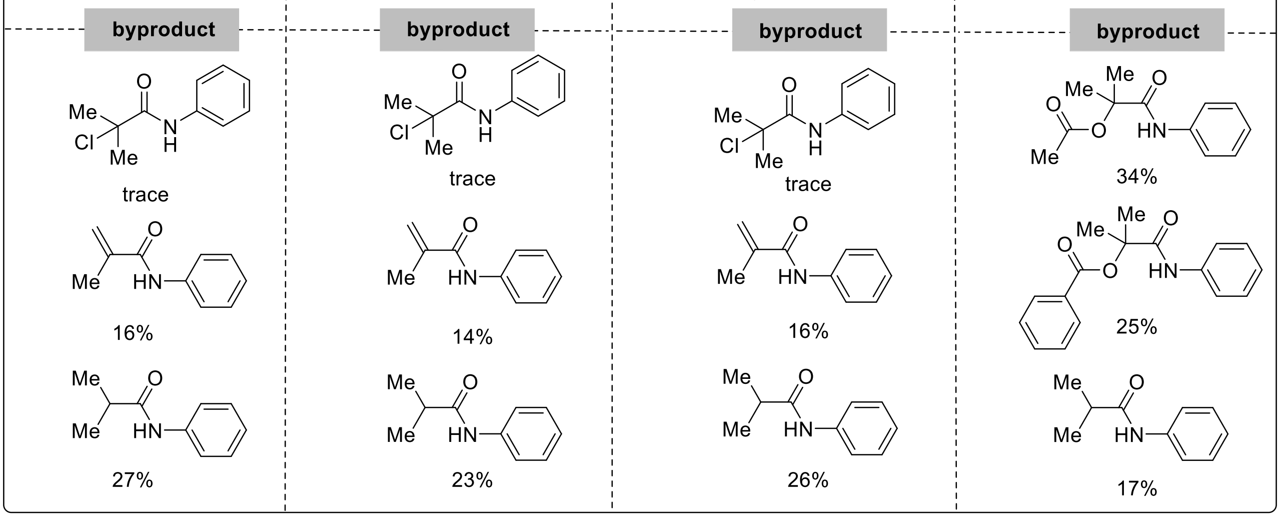

[a]Reaction conditions: **1a** (0.3 mmol), **2a** (0.1 mmol) under the above reaction conditions. GC Yields of the corresponding methyl esters were determined by GC analysis after esterification with K_2CO_3 (2.0 equiv.) and MeI (5.0 equiv.) in NMP using *n*-tetradecane as the internal standard.

Supplementary Table 1. Screening of the Ligand for *meta*-C–H alkylation of benzoic acid[a].

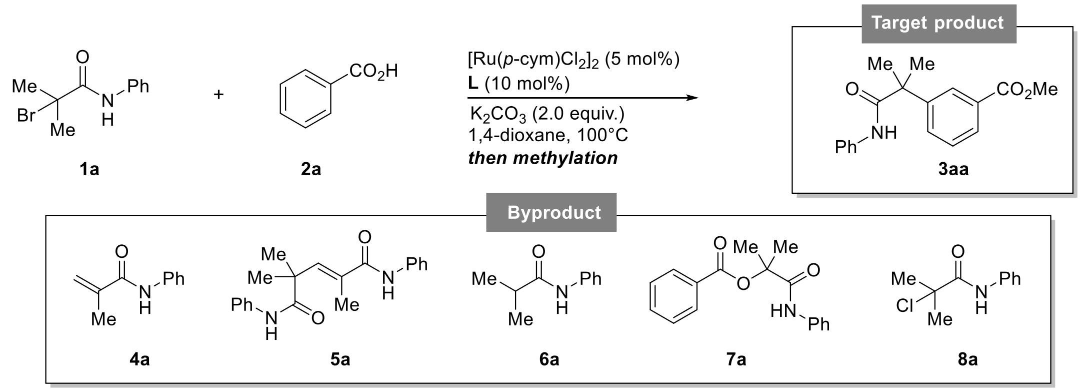

no ligand

3aa: n.d.
4a/5a/6a/7a/8a: 17%/--/26%/--/--

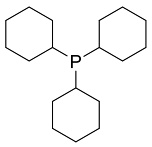

L1

3aa: <5
4a/5a/6a/7a/8a: 13%/--/36%/--/--

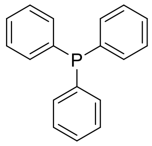

L2

3aa: 5%
4a/5a/6a/7a/8a:12%/--/35%/--/--

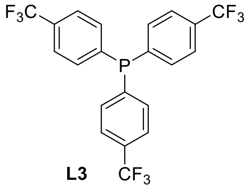

L3

3aa: 9%
4a/5a/6a/7a/8a: 17%/--/36%/--/--

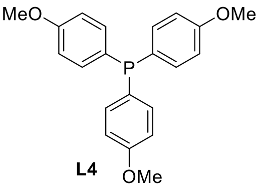

L4

3aa: <5
4a/5a/6a/7a/8a: 11%/--/26%/--/<5

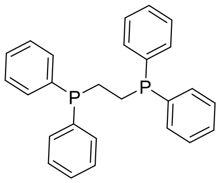

L5

3aa: n.d.
4a/5a/6a/7a/8a: 16%/--/21%/--/<5

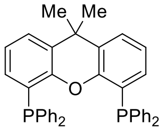

L6

3aa: n.d.
4a/5a/6a/7a/8a: 13%/--/31%/--/--

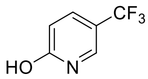

L7

3aa: n.d.
4a/5a/6a/7a/8a: 24%/--/39%/--/--

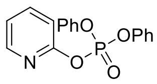

L8

3aa: n.d.
4a/5a/6a/7a/8a: 21%/--/31%/6%/<5

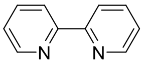

L9

3aa: 25%
4a/5a/6a/7a/8a: 6%/12%/19%/--/--

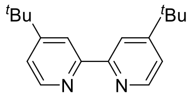

L10

3aa: 25%
4a/5a/6a/7a/8a: 6%/10%/15%/--/--

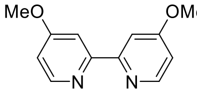

L11

3aa: 10%
4a/5a/6a/7a/8a: 7%/<5/15%/--/--

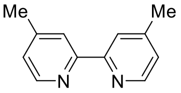

L12

3aa: 27%
4a/5a/6a/7a/8a: 6%/9%/15%/--/--

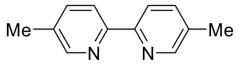

L13

3aa: 31%
4a/5a/6a/7a/8a: <5/12%/11%/--/--

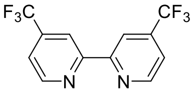

L14

3aa: <5
4a/5a/6a/7a/8a: 13%/--/28%/--/--

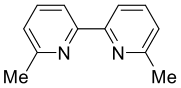

L15

3aa: n.d.
4a/5a/6a/7a/8a: 38%/--/17%/--/--

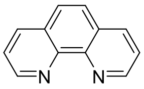

L16

3aa: <5
4a/5a/6a/7a/8a: 22%/--/43%/--/<5

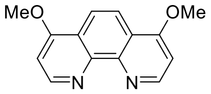

L17

3aa: 20%
4a/5a/6a/7a/8a: <5/7%/10%/--/--

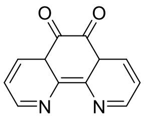

L18

3aa: n.d.
4a/5a/6a/7a/8a: <5/--/19%/--/--

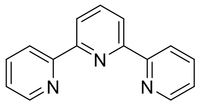

L19

3aa: n.d.
4a/5a/6a/7a/8a: 5%/--/15%/7%/--

[a]Reaction conditions: **1a** (0.1 mmol), **2a** (0.2 mmol), [Ru(*p*-cym)Cl2]2 (5 mol%), **L** (10 mol%), K_2CO_3 (2 equiv.), 1,4-dioxane (1 mL), 100 °C, N_2, 12 h.

[b]The yields of the corresponding methyl esters were determined by GC analysis after esterification with K_2CO_3 (2.0 equiv.) and MeI (5.0 equiv.) in NMP using *n*-tetradecane as internal standard. And the yields of product **3aa**, **4a**, **5a**, **6a**, **7a** and **8a** represent GC yields.

Supplementary Table 2. Screening the solvent for *meta*-C–H alkylation of benzoic acid[a].

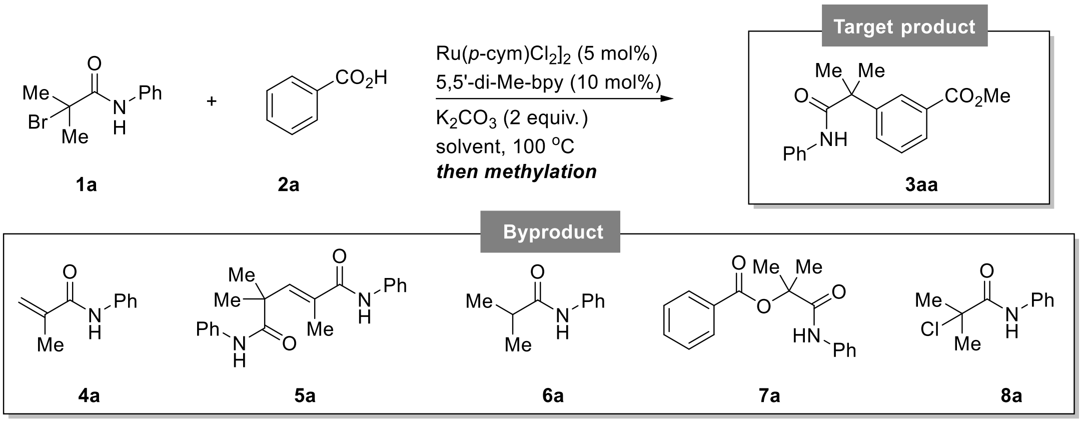

Entry	Solvent	**3aa**/(yield)%[b]	**4a**/**5a**/**6a**/**7a**/**8a**(yield)%[b]
1	1,4-dioxane	31	<5/12/11/--/<5
2[c]	THF	9	10/<5/19/--/<5
3	toluene	trace	13/<5/33/--/<5
4	hexane	n.d.	21/<5/36/--/<5
5	HFIP	n.d.	23/<5/30/--/<5
6	TFE	trace	9/<5/29/--/--
7	^{t}BuOH	41	<5/<5/<5/--/--
8	^{t}BuOH:HFIP = 3:1	trace	16/<5/6/--/--
9	^{t}BuOH:HFIP = 9:1	50	<5/<5/<5/--/--
10	^{t}BuOH:TFE = 9:1	46	<5/<5/<5/--/--
11	^{t}BuOH: HOAc = 9:1	37	16/<5/7/--/--
12	CH_3CN	n.d.	<5/<5/<5/--/--
13	DMF	9	15/<5/7/--/<5
14	NMP	7	6/<5/19/--/--

[a]Reaction conditions: **1a** (0.1 mmol), **2a** (0.2 mmol), [Ru(*p*-cym)Cl_2]$_2$ (5 mol%), 5,5'-di-Me-bpy (10 mol%), K_2CO_3 (2 equiv.), solvent (1 mL), 100 °C, N_2, 12 h. [b]The yields of the corresponding methyl esters were determined by GC analysis after esterification with K_2CO_3 (2.0 equiv.) and MeI (5.0 equiv.) in NMP using *n*-tetradecane as internal standard. And the yields of product **3aa**, **4a**, **5a**, **6a**, **7a** and **8a** represent GC yields.

Supplementary Table 3. Screening the base for *meta*-C–H alkylation of benzoic acid[a].

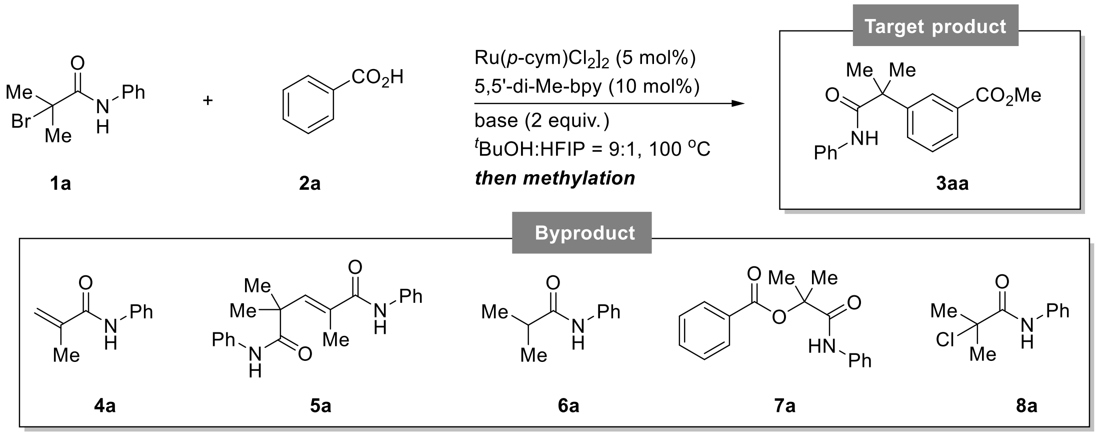

Entry	Base	**3aa**/(yield)%[b]	**4a**/**5a**/**6a**/**7a**/**8a**(yield)%[b]
1	K_2CO_3	50	<5/<5/<5/--/--
2	Na_2CO_3	8	7/<5/15/--/--
3	Cs_2CO_3	9	<5/<5/<5/--/--
4	K_3PO_4	30	<5/<5/<5/--/--
5	K_2HPO_4	59	<5/<5/<5/--/--
6	$KHCO_3$	36	<5/<5/<5/--/--
7	$NaHCO_3$	21	11/<5/23/--/--
8	LiOAc	n.d.	40/--/46/--/--
9	NaOAc	trace	12/<5/25/--/--
10	$Mg(OAc)_2$	trace	14/--/16/--/--
11	$Zn(OAc)_2$	trace	10/--/14/--/--
12	CsOAc	n.d.	<5/<5/<5/<5/<5
13	KOAc	61	<5/<5/<5/--/--
14[c]	KOAc	49	<5/<5/<5/--/--
15[d]	KOAc	51	<5/<5/<5/--/--

[a]Reaction conditions: **1a** (0.1 mmol), **2a** (0.2 mmol), [Ru(*p*-cym)Cl$_2$]$_2$ (5 mol%), 5,5'-di-Me-bpy (10 mol%), base (2 equiv.), ^{t}BuOH:HFIP = 9:1(1 mL), 100 °C, N_2, 12 h. [b]The yields of the corresponding methyl esters were determined by GC analysis after esterification with K_2CO_3 (2.0 equiv.) and MeI (5.0 equiv.) in NMP using *n*-tetradecane as internal standard. And the yields of product **3aa**, **4a**, **5a**, **6a**, **7a** and **8a** represent GC yields. [c]80 °C. [d]120 °C.

Supplementary Table 4. Screening the additive for *meta*-C–H alkylation of benzoic acid[a].

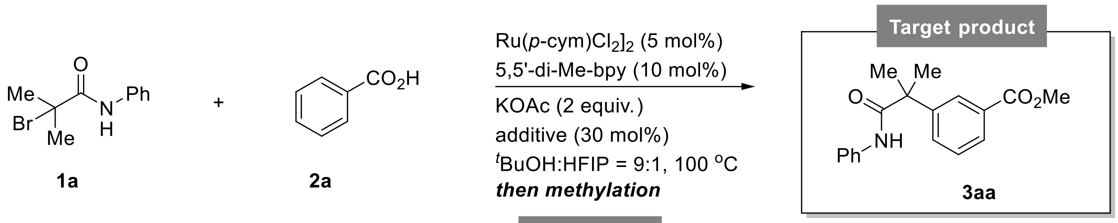

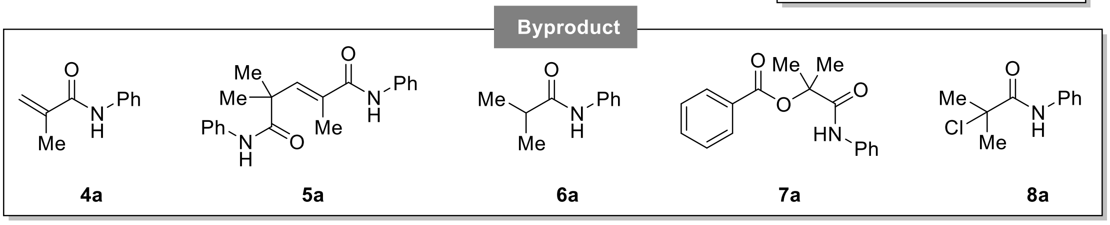

Entry	Additive	**3aa**/(yield)%[b]	**4a**/**5a**/**6a**/**7a**/**8a**(yield)%[b]
1	-	61	<5/<5/<5/--/--
2	AgOTf	53	<5/<5/<5/--/--
3	$AgNTf_2$	53	<5/<5/<5/--/--
4	KCl	57	5/<5/<5/--/--
5	NaCl	64	<5/<5/<5/--/--
6	LiCl	81	<5/--/<5/--/--
7	Li_3PO_4	71	<5/<5/<5/--/--
8	LiOTf	78	<5/--/<5/--/--
9	LiOAc	84	<5/--/<5/--/--
10	LiBr	86	<5/--/<5/--/--
11	Bu_4NBr	48	<5/<5/<5/--/--
12	KBr	51	<5/<5/<5/--/--
13	KOTf	60	<5/<5/<5/--/--
14	KOAc	51	<5/<5/<5/--/--
15	$Mg(OAc)_2$	61	<5/<5/<5/--/--
16	$MgBr_2$	66	<5/<5/<5/--/--
17	$Zn(OAc)_2$	59	<5/<5/<5/--/--
18	$Sc(OTf)_3$	85	<5/<5/<5/--/--
19[c]	KBr (2 equiv.)	15	15/--/30/--/--
20[c]	KCl (2 equiv.)	24	14/--/25/--/--

[a]Reaction conditions: **1a** (0.1 mmol), **2a** (0.2 mmol), $[Ru(p\text{-}cym)Cl_2]_2$ (5 mol%), 5,5'-di-Me-bpy (10 mol%), KOAc (2 equiv.), additive (30 mol%), ^{t}BuOH:HFIP = 9:1(1 mL), 100 °C, N_2, 12 h. [b]The yields of the corresponding methyl esters were determined by GC analysis after esterification with K_2CO_3 (2.0 equiv.)

and MeI (5.0 equiv.) in NMP using *n*-tetradecane as internal standard. and the yields of product **3aa**, **4a**, **5a**, **6a**, **7a** and **8a** represent GC yields. [c]$Zn(OAc)_2$ (2 equiv.) instead of KOAc (2 equiv.).

Supplementary Table 5. Some variation from standard conditions for carboxylate directed *meta*-C–H alkylation[a].

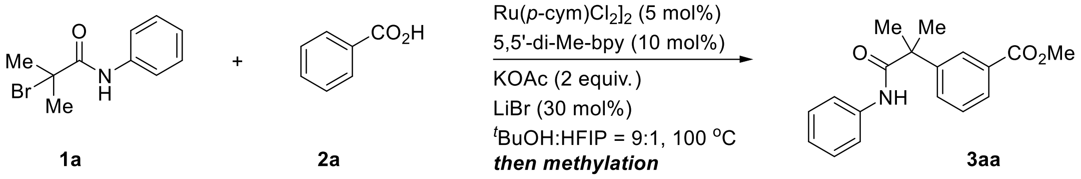

Entry	Variation from standard conditions[a]	Yield of **3aa** (%) [b]
1	-	86 (78)
2[c]	-	85
3	without ligand	n.d.
4	without base	n.d.
5	without LiBr	61

[a]Reaction conditions: **1a** (0.1 mmol), **2a** (0.2 mmol), [Ru(*p*-cym)Cl_2]$_2$ (5 mol%), 5,5'-di-Me-bpy (10 mol%), KOAc (2 equiv.), LiBr (30 mol%), ^{t}BuOH:HFIP = 9:1(1 mL), 100 ºC, N_2, 12 h. [b]The yields of the corresponding methyl esters were determined by GC analysis after esterification with K_2CO_3 (2.0 equiv.) and MeI (5.0 equiv.) in NMP using *n*-tetradecane as internal standard. [c][Ru(*p*-cym)Cl_2]$_2$ (2.5 mol%), 5,5'-di-Me-bpy (5 mol%).

Supplementary Table 6. Optimization of reaction conditions for carboxylate directed *meta*-C–H difluoroalkylation[a].

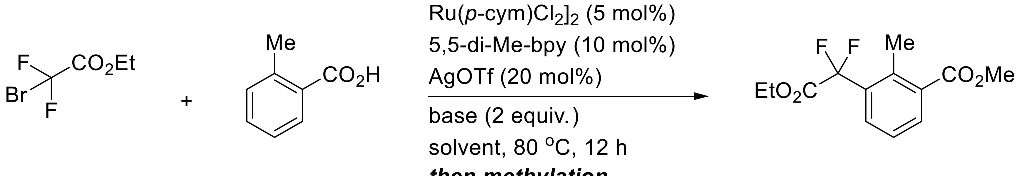

Entry	Base	Solvent	Yield of **3bb** (%)[b]
1	K_2CO_3	1,4-dioxane	n.d.
2	KOAc	1,4-dioxane	19
3	K_3PO_4	1,4-dioxane	11
4	K_2HPO_4	1,4-dioxane	n.d.
5	Na_2CO_3	1,4-dioxane	n.d.
6	$NaHCO_3$	1,4-dioxane	n.d.
7	NaOAc	1,4-dioxane	n.d.
8	KOAc	THF	19
9	KOAc	1,2-ethanediol	n.d.
10	KOAc	CPME	10
11	KOAc	DMF	n.d.
12	KOAc	toluene	<5
13	KOAc	HFIP	n.d.
14	KOAc	TFE	n.d.
15	KOAc	^{n}BuOH	n.d.
16	KOAc	^{i}PrOH	29
17	KOAc	^{t}BuOH	49
18	KOAc	^{t}BuOH/HFIP	n.d.
19[c]	KOAc	^{t}BuOH	61

[a]Reaction conditions: **1bb** (0.1 mmol), **2b** (0.2 mmol), [Ru(*p*-cym)Cl_2]$_2$ (5 mol%), 5,5'-di-Me-bpy (10 mol%), AgOTf (20 mol%), base (2 equiv.), solvent (1 mL), 80 °C, N_2, 12 h. [b]GC yield with n-tetradecane as internal standard; [c]100 °C.

Supplementary Table 7. Optimization of reaction conditions for carboxylate directed *meta*-C–H difluoroalkylation[a].

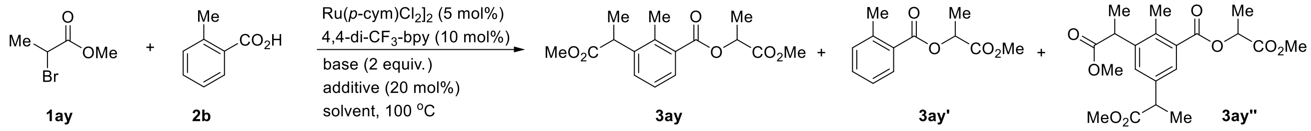

Entry	Base	Additive	Solvent	Yield of **3ay/3ay'/3ay''** (%)[b]
1	K_2CO_3	LiBr	1,4-dioxane	14/--/33
2	KOAc	LiBr	1,4-dioxane	9/--/33
3	KOAc	LiBr	^{t}BuOH	20/--/33
4	KOAc	LiBr	^{t}BuOH/HFIP=9:1	20/--/21
5	K_2CO_3	LiBr	^{t}BuOH/HFIP=9:1	15/9/33
6	K_2CO_3	LiBr	HFIP	n.d./--/<5
7	K_2CO_3	LiBr	^{t}BuOH/HFIP=10:1	<5/<5/<5
8	K_2CO_3	LiBr	1,4-dioxane/HFIP=10:1	14/20/13
9	K_3PO_4	LiBr	1,4-dioxane/HFIP=10:1	26/<5/41
10	K_2HPO_4	LiBr	1,4-dioxane/HFIP=10:1	11/--/31
11	$KHCO_3$	LiBr	1,4-dioxane/HFIP=10:1	--/--/64
12	$NaHCO_3$	LiBr	1,4-dioxane/HFIP=10:1	<5/--/77
13	Na_2CO_3	LiBr	1,4-dioxane/HFIP=10:1	--/--/76
14	KOAc	LiBr	1,4-dioxane/HFIP=10:1	<5/--/37
15	Na_2CO_3	-	^{t}BuOH/HFIP=10:1	29/7/29
16	Na_2CO_3	-	^{t}BuOH/HFIP=20:1	26/11/33
17	Na_2CO_3	AgOTf	^{t}BuOH/HFIP=20:1	40/14/20
18	Na_2CO_3	AgNTf	^{t}BuOH/HFIP=20:1	37/11/14
19	Na_2CO_3	$AgSbF_6$	^{t}BuOH/HFIP=20:1	31/7/24
20	Na_2CO_3	$In(OTf)_3$	^{t}BuOH/HFIP=20:1	15/<5/25
21	Na_2CO_3	$Zn(OTf)_3$	^{t}BuOH/HFIP=20:1	33/<5/20
22	Na_2CO_3	$Sn(OTf)_3$	^{t}BuOH/HFIP=20:1	--/--/74
23	Na_2CO_3	$Fe(OTf)_3$	^{t}BuOH/HFIP=20:1	30/9/29
24	Na_2CO_3	$Fe(OTf)_3$	^{t}BuOH/HFIP=20:1	30/9/29

[a]Reaction conditions: **1ay** (0.4 mmol), **2b** (0.1 mmol), $[Ru(p\text{-cym})Cl_2]_2$ (5 mol%), 4,4-di-CF_3-bpy (10 mol%), base (2 equiv.), additive (20 mol%), solvent (1 mL), 100 °C, N_2, 12 h. [b]GC yield with n-tetradecane as internal standard (uncalibrated yield).

1.5. General Procedures for Carboxylic Acid Directed *Meta*-Alkylation

Procedure A: A 4 mL oven dried vessel was charged with alkyl bromide **1** (0.3 mmol, 1.0 equiv.), aromatic carboxylic acids **2** (0.6 mmol, 2.0 equiv.), $[Ru(p\text{-cym})Cl_2]_2$ (4.6 mg, 2.5 mol%), 5,5'-di-Me-bpy (2.8 mg, 5 mol%), KOAc (58.8 mg, 2.0 equiv.), LiBr (7.8 mg, 30 mol%), the vessel was flushed with 3 alternating vacuum and nitrogen purging cycles, followed by the addition of ^{t}BuOH (1.8 mL) and HFIP (0.2 mL), and the resulting mixture was stirred at 100 °C for 12 h. The mixture was then cooled to RT. Diluted HCl (1M, 100 mL) was added to the reaction mixture with stirring until pH = 1 and extracted twice with ethyl acetate (100 mL). The organic phase was dried over $MgSO_4$, filtered and concentrated. The crude product was purified by flash column chromatography (petroleum ether/ethyl acetate/formic acid = 80/20/1) to give the corresponding products or the crude product was isolated as the corresponding methylation product to which K_2CO_3 (2 equiv.), MeI (5 equiv.) and NMP (2.0 mL) were added at 60 °C for a further 2 h. Saturated sodium chloride solution was then added and the resulting mixture was extracted with ethyl acetate (3× 20 mL). The combined organic layers were dried over $MgSO_4$, filtered and the volatile components were removed under reduced pressure. The residue was purified by column chromatography (petroleum ether/ethyl acetate) to give the alkylation product in the form of its methyl ester.

Procedure B: A 4 mL oven dried vessel was filled with alkyl bromide **1** (0.3 mmol, 1.0 equiv.), aromatic carboxylic acids **2** (0.6 mmol, 2.0 equiv.), $[Ru(p\text{-cym})Cl_2]_2$ (4.6 mg, 2.5 mol%), 5,5'-di-Me-bpy (2.8 mg, 5 mol%), KOAc (58.8 mg, 2.0 equiv.), LiBr (7.8 mg, 30 mol%), the vessel was purged three times with alternating vacuum and nitrogen, 1,4-dioxane (2 mL) was added and the resulting mixture was stirred at 100 °C for 12 h. The mixture was then cooled to RT. Diluted HCl (1M, 100 mL) was added to the reaction mixture with stirring until pH = 1 and extracted twice with ethyl acetate (100 mL). The organic phase was dried over $MgSO_4$, filtered and concentrated. The crude product was purified by flash column chromatography (petroleum ether/ethyl acetate/formic acid = 80/20/1) to give the corresponding products or the crude product was isolated as the corresponding methylation product to which K_2CO_3 (2 equiv.), MeI (5 equiv.) and NMP (2.0 mL) were added at 60 °C for a further 2 h. Saturated sodium chloride solution was then added and the resulting mixture was extracted with ethyl acetate (3× 20 mL). The combined organic layers were dried over $MgSO_4$, filtered and the volatile components were removed under reduced pressure. The residue was purified by column chromatography (petroleum ether/ethyl acetate) to give the alkylation product in the form of its methyl ester.

Procedure C: A 4 mL oven dried vessel was filled with alkyl bromide **1** (1.2 mmol, 4 equiv.), aromatic carboxylic acids **2** (0.3 mmol, 1.0 equiv.), $[Ru(p\text{-cym})Cl_2]_2$ (9.2 mg, 5.0 mol%), 4,4'-di-CF_3-bpy (8.8 mg, 10 mol%), Na_2CO_3 (63.6 mg, 2.0 equiv.), AgOTf (15.4 mg, 20 mol%), the vessel was purged three times with alternating vacuum and nitrogen, ^{t}BuOH (2 mL), HFIP (100 uL) was added and the resulting mixture was stirred at 100 °C for 12 h. The mixture was then cooled to RT. Diluted HCl (1M, 100 mL) was added to the reaction mixture with stirring until pH = 1 and extracted twice with ethyl acetate (100 mL). The organic phase was dried over $MgSO_4$, filtered and concentrated. The crude product was purified by flash column chromatography (petroleum ether/ethyl acetate/formic acid = 80/20/1) to give the corresponding products or the crude product was isolated as the corresponding methylation product to which K_2CO_3 (2 equiv.), MeI (5 equiv.) and NMP (2.0 mL) were added at 60 °C for a further 2 h. Saturated sodium chloride solution was then added and the resulting mixture was extracted with ethyl acetate (3× 20 mL). The combined organic layers were dried over $MgSO_4$, filtered and the volatile components were removed under reduced pressure. The residue was purified by column chromatography (petroleum ether/ethyl acetate) to give the alkylation product in the form of its methyl ester.

Procedure D: A 4 mL oven dried vessel was filled with alkyl bromide **1** (0.3 mmol, 1.0 equiv.), aromatic carboxylic acids **2** (0.6 mmol, 2.0 equiv.), $[Ru(p\text{-cym})Cl_2]_2$ (9.2 mg, 5.0 mol%), 5,5'-di-Me-bpy (5.6 mg, 10 mol%), AgOTf (15.3 mg, 20 mol%), KOAc (58.8 mg, 2.0 equiv.), the vessel was purged three times with alternating vacuum and nitrogen, ^{t}BuOH (2 mL) was added and the resulting mixture was stirred at 100 °C for 12 h. The mixture was then cooled to RT. Diluted HCl (1M, 100 mL) was added to the reaction mixture with stirring until pH = 1 and extracted twice with ethyl acetate (100 mL). The organic phase was dried over $MgSO_4$, filtered and concentrated. The crude product was purified by flash column chromatography (petroleum ether/ethyl acetate/formic acid = 80/20/1) to give the corresponding products or the crude product was isolated as the corresponding methylation product to which K_2CO_3 (2 equiv.), MeI (5 equiv.) and NMP (2.0 mL) were added at 60 °C for a further 2 h. Saturated sodium chloride solution was then added and the resulting mixture was extracted with ethyl acetate (3× 20 mL). The combined organic layers were dried over $MgSO_4$, filtered and the volatile components were removed under reduced pressure. The residue was purified by column chromatography (petroleum ether/ethyl acetate) to give the alkylation product in the form of its methyl ester.

Procedure E: A 4 mL oven dried vessel was filled with alkyl bromide **1** (0.3 mmol, 1.0 equiv.), aromatic carboxylic acids **2** (0.6 mmol, 2.0 equiv.), $[Ru(p\text{-cym})Cl_2]_2$ (4.6 mg, 2.5 mol%), 4,4'-di-CF_3-bpy (4.4 mg, 5 mol%), KOAc (58.8 mg, 2.0 equiv.), LiBr (7.8 mg, 30 mol%), the vessel was purged three times with alternating vacuum and nitrogen, ^{t}BuOH (1.8 mL) and HFIP (0.2 mL) were added and the resulting mixture was stirred at 100 °C for 12 h. The mixture was then cooled to RT. Diluted HCl (1M, 100 mL) was added to the reaction

mixture with stirring until pH = 1 and extracted twice with ethyl acetate (100 mL). The organic phase was dried over $MgSO_4$, filtered and concentrated. The crude product was purified by flash column chromatography (petroleum ether/ethyl acetate/formic acid = 80/20/1) to give the corresponding products or the crude product was isolated as the corresponding methylation product to which K_2CO_3 (2 equiv.), MeI (5 equiv.) and NMP (2.0 mL) were added at 60 °C for a further 2 h. Saturated sodium chloride solution was then added and the resulting mixture was extracted with ethyl acetate (3× 20 mL). The combined organic layers were dried over $MgSO_4$, filtered and the volatile components were removed under reduced pressure. The residue was purified by column chromatography (petroleum ether/ethyl acetate) to give the alkylation product in the form of its methyl ester.

1.6. Analysis Data for the Products

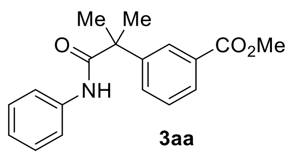

3aa

Methyl-3-(2-methyl-1-oxo-1-(phenylamino)propan-2-yl)benzoate (3aa)

Compound **3aa** was prepared following **Procedure A**, starting from 2-bromo-2-methyl-*N*-phenylpropanamide (108 mg, 0.45 mmol) and benzoic acid (37 mg, 0.3 mmol). After purification, **3aa** was obtained as yellow solid (69.5 mg, 78% yield); **MP** = 108 – 110 ºC; **^{1}H NMR** (500 MHz, $CDCl_3$) δ 8.14 (s, 1H), 7.99 (d, *J* = 7.7 Hz, 1H), 7.63 (d, *J* = 7.8 Hz, 1H), 7.47 (t, *J* = 7.8 Hz, 1H), 7.36 (d, *J* = 8.1 Hz, 2H), 7.26 (t, *J* = 7.8 Hz, 2H), 7.07 (t, *J* = 7.4 Hz, 1H), 6.78 (s, 1H), 3.93 (s, 3H), 1.70 (s, 6H); **^{13}C NMR** (125 MHz, $CDCl_3$) δ 174.8 (C_q), 166.8 (C_q), 145.1 (C_q), 137.8 (C_q), 131.3 (CH), 130.8 (CH), 129.1 (C_q), 128.9 (CH), 128.6 (CH), 127.1 (CH), 124.3 (CH), 119.8 (CH), 52.3 (CH_3), 48.0 (C_q), 27.0 (CH_3); **ESI-HRMS** (m/z): $[M-H]^+$calcd for $C_{18}H_{18}NO_3$, 296.1292, found: 296.1296.

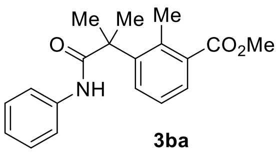

3ba

Methyl-2-methyl-3-(2-methyl-1-oxo-1-(phenylamino)propan-2-yl)benzoate (3ba)

Compound **3ba** was prepared following **Procedure A**, starting from 2-bromo-2-methyl-*N*-phenylpropanamide (72 mg, 0.3 mmol) and 2-methylbenzoic acid (82 mg, 0.6 mmol). After purification, **3ba** was obtained as yellow solid (70.9 mg, 76% yield); **MP** = 107 – 109 ºC; **^{1}H NMR** (500 MHz, $CDCl_3$) δ 7.71 (d, *J* = 7.7 Hz, 1H), 7.66 (d, *J* = 7.9 Hz, 1H), 7.34 (dd, *J* = 7.8, 3.9 Hz, 3H), 7.28 – 7.25 (m, 2H), 7.07 (t, *J* = 7.3 Hz, 1H), 6.79 (s, 1H), 3.88 (s, 3H), 2.45 (s, 3H), 1.68 (s, 6H); **^{13}C NMR** (125 MHz, $CDCl_3$) δ 176.1 (C_q), 168.9 (C_q), 143.4 (C_q), 137.9 (C_q), 137.8 (C_q), 133.8 (CH), 129.3 (CH), 129.1 (C_q), 128.9 (CH), 126.2 (CH), 124.4 (CH), 120.0 (CH), 52.2 (CH_3), 48.3 (C_q), 27.5 (CH_3), 17.7 (CH_3); **ESI-HRMS** (m/z): $[M+H]^+$calcd for $C_{19}H_{22}NO_3$, 312.1594 , found: 312.1586.

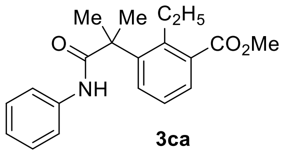

3ca

Methyl-2-ethyl-3-(2-methyl-1-oxo-1-(phenylamino)propan-2-yl)benzoate (3ca)

Compound **3ca** was prepared following **Procedure A**, starting from 2-bromo-2-methyl-*N*-phenylpropanamide (72 mg, 0.3 mmol) and 2-ethylbenzoic acid (90 mg, 0.6 mmol). After purification, **3ca** was obtained as yellow solid (40.9 mg, 42% yield); **MP** = 109 – 111 ºC; **^{1}H NMR** (500 MHz, $CDCl_3$) δ 7.56 (t, *J* = 8.3 Hz, 2H), 7.24 (t, *J* = 7.9 Hz, 3H), 7.16 (t, *J* = 7.6 Hz, 2H), 6.97 (t, *J* = 7.3 Hz, 1H), 6.74 (s, 1H), 3.79 (s, 3H), 2.93 (q, *J* = 7.3 Hz, 2H), 1.58 (s, 6H), 0.91 (t, *J* = 7.3 Hz, 3H); **^{13}C NMR** (125 MHz, $CDCl_3$) δ 176.2 (C_q), 169.5 (C_q), 144.1 (C_q), 142.6 (C_q), 137.7 (C_q), 133.5 (C_q), 129.6 (CH), 129.1 (CH), 128.8 (CH), 126.1 (CH), 124.2 (CH), 119.9 (CH), 52.2 (CH_3), 48.3 (C_q), 28.1 (CH_3), 22.5 (CH_2), 15.4 (CH_3); **ESI-HRMS** (m/z): $[M+H]^+$calcd for $C_{20}H_{24}NO_3$, 326.1751, found: 326.1745.

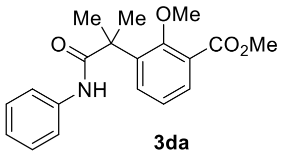

3da

Methyl-2-methoxy-3-(2-methyl-1-oxo-1-(phenylamino)propan-2-yl)benzoate (3da)

Compound **3da** was prepared following **Procedure A**, starting from 2-bromo-2-methyl-*N*-phenylpropanamide (72 mg, 0.3 mmol) and 2-methoxybenzoic acid (91 mg, 0.6 mmol). After purification, **3da** was obtained as white solid (63.8 mg, 65% yield); **MP** = 120 – 122 ºC; **^{1}H NMR** (400 MHz, $CDCl_3$) δ 7.73 (dd, *J* = 7.8, 1.7 Hz, 1H), 7.60 (dd, *J* = 7.8, 1.7 Hz, 1H), 7.46 (d, *J* = 7.7 Hz, 2H), 7.28 (t, *J* = 7.9 Hz, 2H), 7.17 (t, *J* = 7.8 Hz, 1H), 7.06 (t, *J* = 7.4 Hz, 1H), 7.01 (s, 1H), 3.91 (s, 3H), 3.66 (s, 3H), 1.66 (s, 6H); **^{13}C NMR** (100 MHz, $CDCl_3$) δ 175.6 (C_q), 167.1 (C_q), 158.6 (C_q), 138.7 (C_q), 138.2 (CH), 131.0 (C_q), 130.2 (CH), 128.9 (CH), 123.8 (C_q), 123.5 (CH), 122.7

(CH), 119.4 (C_q), 61.7 (CH_3), 52.3 (CH_3), 46.2 (C_q), 26.3 (CH_3); **ESI-HRMS** (m/z): $[M+H]^+$calcd for $C_{19}H_{22}NO_4$, 328.1543, found: 328.1538.

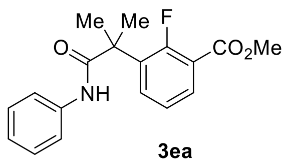

3ea

Methyl-2-fluoro-3-(2-methyl-1-oxo-1-(phenylamino)propan-2-yl)benzoate (3ea)

Compound **3ea** was prepared following **Procedure A**, starting from 2-bromo-2-methyl-*N*-phenylpropanamide (72 mg, 0.3 mmol) and 2-fluorobenzoic acid (84 mg, 0.6 mmol). After purification, **3ea** was obtained as grey solid (85.1 mg, 90% yield); **MP** = 120 – 122 ºC; **^{1}H NMR** (500 MHz, $CDCl_3$) δ 7.84 – 7.80 (m, 1H), 7.57 (td, *J* = 7.7, 1.6 Hz, 1H), 7.35 (d, *J* = 7.7 Hz, 2H), 7.23 – 7.18 (m, 3H), 7.01 (t, *J* = 7.4 Hz, 1H), 6.91 (s, 1H), 3.82 (s, 3H), 1.61 (s, 6H); **^{13}C NMR (**125 MHz, $CDCl_3$) δ 174.1 (C_q), 164.8 (d, $J_{C\text{-}F}$ = 3 Hz, C_q), 160.1 (d, $J_{C\text{-}F}$ = 263 Hz, C_q) , 137.8 (C_q), 133.6 (d, $J_{C\text{-}F}$ = 14 Hz, CH), 131.7 (d, $J_{C\text{-}F}$ = 5 Hz, CH), 131.5 (CH), 128.9 (CH), 124.3 (CH), 124.2 (d, $J_{C\text{-}F}$ = 4 Hz, C_q), 120.2 (CH), 119.7 (d, $J_{C\text{-}F}$ = 11 Hz, C_q), 52.4 (CH_3), 45.9 (C_q), 26.0 (CH_3); **^{19}F NMR** (376 MHz, $CDCl_3$) δ -108.0 (s); **ESI-HRMS** (m/z): $[M+H]^+$calcd for $C_{18}H_{19}FNO_3$, 316.1343, found: 316.1336.

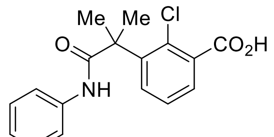

3fa

2-Chloro-3-(2-methyl-1-oxo-1-(phenylamino)propan-2-yl)benzoic acid (3fa)

Compound **3fa** was prepared following **Procedure A**, starting from 2-bromo-2-methyl-*N*-phenylpropanamide (72 mg, 0.3 mmol) and 2-chlorobenzoic acid (94 mg, 0.6 mmol). Methylation step did not run, and product was isolated as acid. After purification, **3fa** was obtained as colorless liquid (75.1 mg, 79% yield); **^{1}H NMR** (500 MHz, DMSO-*d$_6$*) δ 9.00 (s, 1H), 7.72 (d, *J* = 7.6 Hz, 1H), 7.55 (dd, *J* = 13.3, 7.8 Hz, 3H), 7.47 (t, *J* = 7.7 Hz, 1H), 7.27 (t, *J* = 7.8 Hz, 2H), 7.03 (t, *J* = 7.3 Hz, 1H), 1.62 (s, 6H); **^{13}C NMR** (125 MHz, DMSO-*d$_6$*) δ 174.7 (C_q), 168.4 (C_q), 143.9 (C_q), 139.8 (C_q), 135.5 (CH), 130.8 (C_q), 130.6 (C_q), 128.8 (CH), 128.3 (CH), 127.5 (CH), 123.8 (CH), 121.2 (CH), 48.3 (C_q), 26.7 (CH_3); **ESI-HRMS** (m/z): $[M+H]^+$calcd for $C_{17}H_{17}ClNO_3$, 318.0891, found: 318.0884.

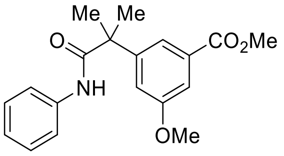

3ga

Methyl-3-methoxy-5-(2-methyl-1-oxo-1-(phenylamino)propan-2-yl)benzoate (3ga)

Compound **3ga** was prepared following **Procedure A**, starting from 2-bromo-2-methyl-*N*-phenylpropanamide (72 mg, 0.3 mmol) and 3-methoxybenzoic acid (91 mg, 0.6 mmol). After purification, **3ga** was obtained as white solid (75.5 mg, 77% yield); **MP** = 122 – 125 ºC ; **^{1}H NMR** (500 MHz, $CDCl_3$) δ 7.62 (s, 1H), 7.36 – 7.31 (m, 3H), 7.19 (dd, *J* = 10.8, 5.2 Hz, 2H), 7.09 – 7.06 (m, 1H), 6.98 (t, *J* = 7.4 Hz, 1H), 6.87 (s, 1H), 3.83 (s, 3H), 3.72 (s, 3H), 1.59 (s, 6H); **^{13}C NMR** (125 MHz, $CDCl_3$) δ 174.7 (C_q), 166.7 (C_q), 159.9 (C_q), 146.6 (C_q), 137.9 (C_q), 131.8 (C_q), 128.8 (CH), 124.2 (CH), 119.7 (CH), 119.7 (CH), 118.2 (CH), 112.4 (CH), 55.4 (CH_3), 52.3 (CH_3), 48.0 (C_q), 26.9 (CH_3); **ESI-HRMS** (m/z): $[M+H]^+$calcd for $C_{19}H_{22}NO_4$, 328.1543, found: 328.1537.

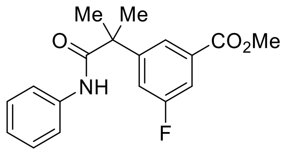

3ha

Methyl-3-fluoro-5-(2-methyl-1-oxo-1-(phenylamino)propan-2-yl)benzoate (3ha)

Compound **3ha** was prepared following **Procedure A**, starting from 2-bromo-2-methyl-*N*-phenylpropanamide (72 mg, 0.3 mmol) and

3-fluorobenzoic acid (84 mg, 0.6 mmol). After purification, **3ha** was obtained as white solid (76.5 mg, 81% yield); **MP** = 125 – 127 ºC ; **^{1}H NMR** (500 MHz, $CDCl_3$) δ 7.82 (t, *J* = 1.5 Hz, 1H), 7.54 (ddd, *J* = 8.6, 2.3, 1.3 Hz, 1H), 7.31 (d, *J* = 7.7 Hz, 2H), 7.28 – 7.24 (m, 1H), 7.19 (dd, *J* = 10.8, 5.1 Hz, 2H), 6.99 (t, *J* = 7.4 Hz, 1H), 6.90 (s, 1H), 3.82 (s, 3H), 1.59 (s, 6H); **^{13}C NMR** (125 MHz, $CDCl_3$) δ 173.9 (C_q), 165.6 (d, J_{C-F} = 4 Hz, C_q), 162.7 (d, J_{C-F} = 248 Hz, C_q) ,147.8 (d, J_{C-F} = 6 Hz, C_q), 137.6 (C_q), 132.6 (d, J_{C-F} = 8 Hz, C_q), 128.9 (CH), 124.4 (CH), 123.0 (CH), (d, J_{C-F} = 3 Hz, CH), 119.9 (CH), 118.3 (d, J_{C-F} = 23 Hz, CH), 115.5 (d, J_{C-F} = 23 Hz, CH), 52.5 (CH_3), 47.9 (C_q), 26.8 (CH_3); **^{19}F NMR** (471 MHz, $CDCl_3$) δ -110.84 (t, J_{C-F} = 9 Hz); **ESI-HRMS** (m/z): [M-H]$^+$calcd for $C_{18}H_{17}FNO_3$, 314.1198, found: 314.1198.

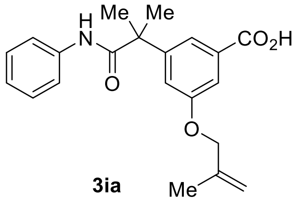

3ia

3-(2-Methyl-1-oxo-1-(phenylamino)propan-2-yl)-5-((2-methylallyl)oxy)benzoic acid (3ia)

Compound **3ia** was prepared following **Procedure A**, starting from 2-bromo-2-methyl-*N*-phenylpropanamide (72 mg, 0.3 mmol) and 3-((2-methylallyl)oxy)benzoic acid (115 mg, 0.6 mmol). Methylation step did not run, and product was isolated as acid. After purification, **3ia** was obtained as white solid (84.7 mg, 80% yield); **^{1}H NMR** (400 MHz, DMSO-*d*$_6$) δ 13.05 (s, 1H), 9.16 (s, 1H), 7.62 – 7.50 (m, 4H), 7.37 (dd, *J* = 2.5, 1.3 Hz, 1H), 7.27 (t, *J* = 7.9 Hz, 2H), 7.18 – 7.15 (m, 1H), 7.03 (t, *J* = 7.4 Hz, 1H), 5.07 (s, 1H), 4.95 (s, 1H), 4.53 (s, 2H), 1.77 (s, 3H), 1.58 (s, 6H); **^{13}C NMR** (125 MHz, DMSO-*d*$_6$) δ 174.7 (C_q), 167.6 (C_q), 158.7 (C_q), 148.1 (C_q), 141.1 (C_q), 139.6 (C_q), 132.5 (C_q), 128.9 (CH), 123.9 (CH), 120.8 (CH), 119.9 (CH), 118.3 (CH), 113.0 (CH), 112.9 (CH_2), 71.6 (CH_2), 47.8 (C_q), 27.1 (CH_3), 19.7 (CH_3); **ESI-HRMS** (m/z): [M-H]$^+$calcd for $C_{21}H_{22}NO_4$, 352.1554, found: 352.1553.

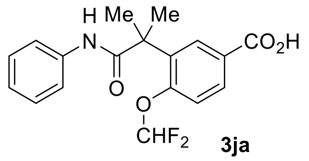

3ja

4-(Difluoromethoxy)-3-(2-methyl-1-oxo-1-(phenylamino)propan-2-yl)benzoic acid (3ja)

Compound **3ja** was prepared following **Procedure A**, starting from 2-bromo-2-methyl-*N*-phenylpropanamide (72 mg, 0.3 mmol) and 4-(difluoromethoxy)benzoic acid (113 mg, 0.6 mmol). Methylation step did not run, and product was isolated as acid. After purification, **3ja** was obtained as colorless liquid (62.8 mg, 60% yield); **^{1}H NMR** (500 MHz, DMSO-*d*$_6$) δ 8.97 (s, 1H), 8.11 (s, 1H), 7.96 (d, *J* = 8.1 Hz, 1H), 7.52 (d, *J* = 8.0 Hz, 2H), 7.29 – 7.19 (m, 4H), 7.01 (t, *J* = 7.3 Hz, 1H), 1.57 (s, 6H); **^{13}C NMR** (125 MHz, DMSO-*d*$_6$) δ 174.7 (C_q), 167.6 (C_q), 153.1 (C_q), 139.8 (C_q), 135.5 (C_q), 130.1 (d, J_{C-F} = 63 Hz, CH), 128.6 (CH), 123.6 (CH), 121.2 (C_q), 118.6 (CH), 116.5 (d, J_{C-F} = 8 Hz, C_q), 114.5 (CH), 45.9 (C_q), 26.2 (CH_3); **^{19}F NMR** (471 MHz, DMSO-*d*$_6$) δ -81.8(s), -81.9 (s); **ESI-HRMS** (m/z): [M-H]$^+$calcd for $C_{18}H_{16}F_2NO_4$, 348.1053, found: 348.1051.

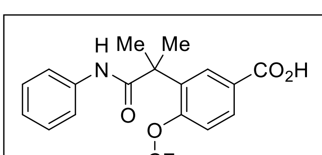

3ka

3-(2-Methyl-1-oxo-1-(phenylamino)propan-2-yl)-4-(trifluoromethoxy)benzoic acid (3ka)

Compound **3ka** was prepared following **Procedure A**, starting from 2-bromo-2-methyl-*N*-phenylpropanamide (72 mg, 0.3 mmol) and 4-(trifluoromethoxy)benzoic acid (124 mg, 0.6 mmol). Methylation step did not run, and product was isolated as acid. After purification, **3ka** was obtained as white solid (67.2 mg, 61% yield); **MP** = 223 – 225 ºC ; **^{1}H NMR** (500 MHz, DMSO-*d*$_6$) δ 9.06 (s, 1H), 8.18 (d, *J* = 2.1 Hz, 1H), 8.01 (dd, *J* = 8.6, 2.1 Hz, 1H), 7.52 (d, *J* = 7.6 Hz, 2H), 7.42 (dd, *J* = 8.6, 1.8 Hz, 1H), 7.26 (t, *J* = 7.9 Hz, 2H), 7.01 (t, *J* = 7.4 Hz, 1H), 1.57 (s, 6H); **^{13}C NMR** (125 MHz, DMSO-*d*$_6$) δ 174.2 (C_q), 167.0 (C_q), 150.5 (C_q), 139.7 (C_q), 136.5 (CH), 132.2 (CH), 130.5 (d, J_{C-F} = 25 Hz, C_q), 129.0 (CH), 128.7 (CH), 123.6 (C_q), 121.3 (q, J_{C-F} = 265 Hz, CH), 120.8 (C_q), 117.8 (CH), 45.9 (C_q), 26.2 (CH_3); **^{19}F NMR** (376 MHz, $CDCl_3$) δ -55.8 (s), -57.7 (s); **ESI-HRMS** (m/z): [M-H]$^+$calcd for $C_{18}H_{15}F_3NO_4$, 366.0959, found: 366.0957.

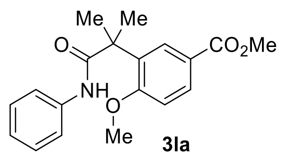

3la

Methyl-4-methoxy-3-(2-methyl-1-oxo-1-(phenylamino)propan-2-yl)benzoate (3la)

Compound **3la** was prepared following **Procedure A**, starting from 2-bromo-2-methyl-*N*-phenylpropanamide (72 mg, 0.3 mmol) and 4-methoxybenzoic acid (91 mg, 0.6 mmol). After purification, **3la** was obtained as white solid (85.3 mg, 87% yield); **MP** = 174 – 176 ºC; **^{1}H NMR** (500 MHz, $CDCl_3$) δ 8.13 (d, *J* = 2.1 Hz, 1H), 8.04 (dd, *J* = 8.5, 2.1 Hz, 1H), 7.36 (d, *J* = 7.6 Hz, 2H), 7.27 (d, *J* = 6.9 Hz, 2H), 7.05 (t, *J* = 7.4 Hz, 1H), 6.94 (d, *J* = 8.6 Hz, 1H), 6.82 (s, 1H), 3.93 (s, 3H), 3.81 (s, 3H), 1.64 (s, 6H); **^{13}C NMR** (125 MHz, $CDCl_3$) δ 175.5 (C_q), 166.8 (C_q), 161.1 (C_q), 138.2 (C_q), 133.1 (CH), 131.3 (CH), 128.8 (CH), 128.3 (CH), 123.8 (C_q), 122.7 (CH), 119.7 (C_q), 111.0 (CH), 55.8 (CH_3), 52.1 (CH_3), 46.0 (C_q), 25.6 (CH_3); **ESI-HRMS** (m/z): $[M+H]^+$calcd for $C_{19}H_{22}NO_4$, 328.1543, found: 328.1538.

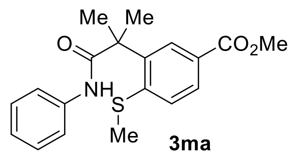

3ma

Methyl-3-(2-methyl-1-oxo-1-(phenylamino)propan-2-yl)-4-(methylthio)benzoate (3ma)

Compound **3ma** was prepared following **Procedure A**, starting from 2-bromo-2-methyl-*N*-phenylpropanamide (72 mg, 0.3 mmol) and 4-(methylthio)benzoic acid (101 mg, 0.6 mmol). After purification, **3ma** was obtained as white solid (31.9 mg, 31% yield); **MP** = 138 – 140 ºC; **^{1}H NMR** (500 MHz, $CDCl_3$) δ 8.09 (d, *J* = 1.8 Hz, 1H), 7.86 (dd, *J* = 8.2, 1.8 Hz, 1H), 7.31 (d, *J* = 8.1 Hz, 3H), 7.19 (dd, *J* = 9.1, 6.8 Hz, 2H), 6.99 (d, *J* = 7.4 Hz, 1H), 6.89 (s, 1H), 3.85 (s, 3H), 2.36 (s, 3H), 1.67 (s, 6H); **^{13}C NMR** (125 MHz, $CDCl_3$) δ 174.9 (C_q), 166.7 (C_q), 145.7 (C_q), 142.2 (C_q), 138.1 (C_q), 128.9 (CH), 128.8 (CH), 127.73 (CH), 127.73 (CH), 127.0 (C_q), 124.1 (CH), 120.0 (CH), 52.2 (CH_3), 48.5 (C_q), 26.5 (CH_3), 17.1 (CH_3); **ESI-HRMS** (m/z): $[M+H]^+$calcd for $C_{19}H_{22}SNO_3$, 344.1315, found: 344.1307.

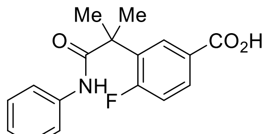

3na

4-Fluoro-3-(2-methyl-1-oxo-1-(phenylamino)propan-2-yl)benzoic acid (3na)

Compound **3na** was prepared following **Procedure A**, starting from 2-bromo-2-methyl-*N*-phenylpropanamide (72 mg, 0.3 mmol) and 4-fluorobenzoic acid (84 mg, 0.6 mmol). Methylation step did not run, and product was isolated as acid. After purification, **3na** was obtained as yellow liquid (28.9 mg, 32% yield); **^{1}H NMR** (500 MHz, DMSO-d_6) δ 9.12 (s, 1H), 8.09 (dd, *J* = 7.8, 2.2 Hz, 1H), 7.94 (ddd, *J* = 8.5, 4.9, 2.2 Hz, 1H), 7.58 – 7.50 (m, 2H), 7.29 – 7.25 (m, 2H), 7.24 (s, 1H), 7.09 – 6.97 (m, 1H), 1.58 (s, 6H); **^{13}C NMR** (125 MHz, DMSO-d_6) δ 174.4 (C_q), 167.2 (C_q), 163.7 (d, J_{C-F} = 250 Hz, C_q), 139.7 (C_q), 133.4 (d, J_{C-F} = 13.8 Hz, CH), 131.0 (d, J_{C-F} = 10 Hz, CH), 130.2 (d, J_{C-F} = 6.3 Hz, CH), 128.8 (CH), 127.5 (d, J_{C-F} = 2.5 Hz, C_q), 123.8 (C_q), 120.9 (CH), 116.5 (d, J_{C-F} = 22.5 Hz, CH), 45.4 (C_q), 26.0 (CH_3); **^{19}F NMR** (376 MHz, $CDCl_3$) δ -100.67; **ESI-HRMS** (m/z): $[M+H]^+$calcd for $C_{17}H_{17}FNO_3$, 302.1187, found: 302.1184.

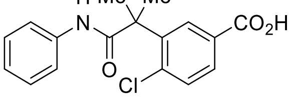

3oa

4-Chloro-3-(2-methyl-1-oxo-1-(phenylamino)propan-2-yl)benzoic acid (3oa)

Compound **3oa** was prepared following **Procedure A**, starting from 2-bromo-2-methyl-*N*-phenylpropanamide (72 mg, 0.3 mmol) and 4-chlorobenzoic acid (94 mg, 0.6 mmol). Methylation step did not run, and product was isolated as acid. After purification, **3oa** was obtained as white solid (51.4 mg, 54% yield); **MP** = 160 – 162 ºC; **^{1}H NMR** (500 MHz, DMSO-d_6) δ 9.02 (s, 1H), 8.17 (d, *J* = 2.0 Hz, 1H), 7.86 (dd, *J* = 8.2, 2.0 Hz, 1H), 7.53 (dd, *J* = 12.1, 7.9 Hz, 3H), 7.29 – 7.22 (m, 2H), 7.02 (t, *J* = 7.4 Hz, 1H), 1.62 (s, 6H); **^{13}C NMR**

(125 MHz, DMSO-d_6) δ 174.3 (C_q), 167.3 (C_q), 142.9 (C_q), 139.7 (C_q), 138.3 (C_q), 131.4 (CH), 130.2 (CH), 130.1 (CH), 129.7 (CH), 128.8 (C_q), 123.7 (CH), 121.0 (CH), 47.8 (C_q), 26.3 (CH_3); **ESI-HRMS** (m/z): $[M-H]^+$calcd for $C_{17}H_{15}ClNO_3$, 316.0746, found: 316.0745.

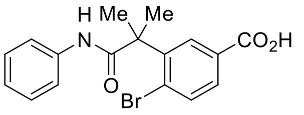

3pa

4-Bromo-3-(2-methyl-1-oxo-1-(phenylamino)propan-2-yl)benzoic acid (3pa)

Compound **3pa** was prepared following **Procedure A**, starting from 2-bromo-2-methyl-*N*-phenylpropanamide (72 mg, 0.3 mmol) and 4-bromobenzoic acid (120 mg, 0.6 mmol). Methylation step did not run, and product was isolated as acid. After purification, **3pa** was obtained as white solid (53.1 mg, 49% yield); **^{1}H NMR** (500 MHz, DMSO-d_6) δ 13.28 (s, 1H), 9.00 (s, 1H), 8.16 (d, *J* = 2.0 Hz, 1H), 7.79 – 7.70 (m, 2H), 7.54 (d, *J* = 8.4 Hz, 2H), 7.28 – 7.21 (m, 2H), 7.02 (t, *J* = 7.4 Hz, 1H), 1.64 (s, 6H); **^{13}C NMR** (125 MHz, DMSO-d_6) δ 174.2 (C_q), 167.5 (C_q), 144.3 (C_q), 139.8 (C_q), 135.2 (CH), 130.7 (CH), 130.4 (CH), 129.7 (C_q), 129.0 (C_q), 128.7 (CH), 123.7 (CH), 121.1 (CH), 49.0 (C_q), 26.6 (CH_3); **ESI-HRMS** (m/z): $[M-H]^+$calcd for $C_{17}H_{15}BrNO_5$, 360.0241, found: 360.0244.

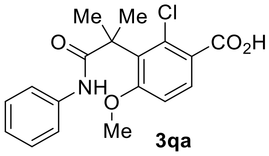

3qa

2-Chloro-4-methoxy-3-(2-methyl-1-oxo-1-(phenylamino)propan-2-yl)benzoic acid (3qa)

Compound **3qa** was prepared following **Procedure A**, starting from 2-bromo-2-methyl-*N*-phenylpropanamide (72 mg, 0.3 mmol) and 2-chloro-4-methoxybenzoic acid (112 mg, 0.6 mmol). Methylation step did not run, and product was isolated as acid. After purification, **3qa** was obtained as colorless liquid (30.2 mg, 29% yield); **^{1}H NMR** (500 MHz, DMSO-d_6) δ 9.05 (s, 1H), 7.59 (d, *J* = 8.6 Hz, 1H), 7.55 (d, *J* = 8.0 Hz, 2H), 7.25 (t, *J* = 7.8 Hz, 2H), 7.07 (d, *J* = 8.7 Hz, 1H), 6.99 (t, *J* = 7.3 Hz, 1H), 3.74 (s, 3H), 1.66 (s, 6H); **^{13}C NMR** (125 MHz, DMSO-d_6) δ 176.0 (C_q), 168.1 (C_q), 161.4 (C_q), 140.4 (C_q), 132.6 (C_q), 132.0 (CH), 129.6 (CH), 128.7 (C_q), 127.5 (CH), 123.2 (C_q), 120.6 (CH), 111.5 (CH), 56.7 (CH_3), 48.9 (C_q), 27.2 (CH_3); **ESI-HRMS** (m/z): $[M+H]^+$calcd for $C_{18}H_{19}ClNO_4$, 348.0997, found: 348.0988.

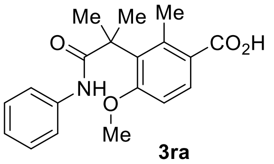

3ra

4-Methoxy-2-methyl-3-(2-methyl-1-oxo-1-(phenylamino)propan-2-yl)benzoic acid (3ra)

Compound **3ra** was prepared following **Procedure A**, starting from 2-bromo-2-methyl-*N*-phenylpropanamide (72 mg, 0.3 mmol) and 4-methoxy-2-methylbenzoic acid (100 mg, 0.6 mmol). Methylation step did not run, and product was isolated as acid. After purification, **3ra** was obtained as yellow liquid (42.2 mg, 43% yield); **^{1}H NMR** (500 MHz, DMSO-d_6) δ 8.91 (s, 1H), 7.64 (d, *J* = 8.6 Hz, 1H), 7.56 (d, *J* = 8.1 Hz, 2H), 7.25 (t, *J* = 7.8 Hz, 2H), 6.99 (d, *J* = 7.3 Hz, 1H), 6.91 (d, *J* = 8.7 Hz, 1H), 3.69 (s, 3H), 2.49 (s, 3H), 1.61 (s, 6H); **^{13}C NMR** (125 MHz, DMSO-d_6) δ 177.3 (C_q), 170.2 (C_q), 160.7 (C_q), 140.4 (C_q), 139.4 (C_q), 133.4 (CH), 129.9 (CH), 128.8 (CH), 127.2 (C_q), 123.1 (CH), 120.4 (C_q), 110.0 (CH), 56.2 (CH_3), 48.2 (C_q), 27.3 (CH_3), 19.7 (CH_3); **ESI-HRMS** (m/z): $[M+H]^+$calcd for $C_{19}H_{22}NO_4$, 328.1907, found: 328.1898.

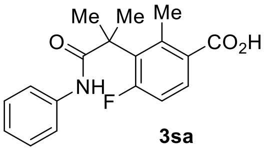

3sa

4-Fluoro-2-methyl-3-(2-methyl-1-oxo-1-(phenylamino)propan-2-yl)benzoic acid (3sa)

Compound **3sa** was prepared following **Procedure A**, starting from 2-bromo-2-methyl-*N*-phenylpropanamide (72 mg, 0.3 mmol) and

4-fluoro-2-methylbenzoic acid (92 mg, 0.6 mmol). Methylation step did not run, and product was isolated as acid. After purification, **3sa** was obtained as colorless liquid (71.8 mg, 76% yield); **^{1}H NMR** (400 MHz, DMSO-d_6) δ 12.94 (s, 1H), 9.31 (s, 1H), 7.64 (dd, J = 8.6, 5.3 Hz, 1H), 7.55 (d, J = 8.5 Hz, 2H), 7.34 – 7.23 (m, 2H), 7.14 – 7.09 (m, 1H), 7.08 – 7.03 (m, 1H), 2.39 (s, 3H), 1.65 (d, J = 4.1 Hz, 6H); **^{13}C NMR** (100 MHz, DMSO-d$_6$) δ 176.2 (C$_q$), 169.7 (C$_q$), 163.7 (d, $J_{C\text{-}F}$ = 311.3 Hz, C$_q$), 140.2 (d, $J_{C\text{-}F}$ = 6.3 Hz, C$_q$), 139.8 (C$_q$), 131.7 (d, $J_{C\text{-}F}$ = 12.5 Hz, CH), 131.0 (d, $J_{C\text{-}F}$ = 3.8 Hz, CH), 130.0 (d, $J_{C\text{-}F}$ = 13.8 Hz, CH), 128.9 (C$_q$), 123.8 (C$_q$), 120.8 (CH), 114.8 (d, $J_{C\text{-}F}$ = 33.8 Hz, CH), 47.8 (C$_q$), 27.8 (d, $J_{C\text{-}F}$ = 10.0 Hz, CH$_3$), 18.4 (CH$_3$); **^{19}F NMR** (471 MHz, CDCl$_3$) δ -98.1 (s); **ESI-HRMS** (m/z): [M+H]$^+$calcd for $C_{18}H_{19}FNO_3$, 316.1343, found: 316.1335.

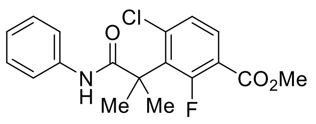

3ta

Methyl-4-chloro-2-fluoro-3-(2-methyl-1-oxo-1-(phenylamino)propan-2-yl)benzoate (3ta)

Compound **3ta** was prepared following **Procedure A**, starting from 2-bromo-2-methyl-*N*-phenylpropanamide (72.3 mg, 0.3 mmol) and 4-chloro-2-fluorobenzoic acid (104.4 mg, 0.6 mmol). After purification, **3ta** was obtained as colorless liquid (29.3 mg, 28% yield); **^{1}H NMR** (400 MHz, CDCl$_3$) δ 7.75 (dd, J = 8.5, 7.2 Hz, 1H), 7.43 (d, J = 7.6 Hz, 2H), 7.34 – 7.23 (m, 3H), 7.16 – 7.05 (m, 2H), 3.91 (s, 3H), 1.83 (d, J = 4.7 Hz, 6H); **^{13}C NMR** (125 MHz, CDCl$_3$) δ 174.3 (C$_q$), 164.2 (d, J = 3.8 Hz, C$_q$), 161.7 (d, J = 263.8 Hz, C$_q$), 140.5 (d, J = 6.3 Hz, C$_q$), 137.9 (C$_q$), 131.0 (d, J = 2.5 Hz, CH), 130.4 (d, J = 13.8 Hz, CH), 128.9 (CH), 127.1 (d, J = 3.8 Hz, CH), 124.4 (C$_q$), 120.3 (CH), 119.3 (d, J = 13.8 Hz, C$_q$), 52.6 (CH$_3$), 49.2 (d, J = 2.5 Hz, C$_q$), 26.9 (d, J = 8.8 Hz, CH$_3$); **^{19}F NMR** (376 MHz, CDCl$_3$) δ -104.08 (s); **ESI-HRMS (m/z):** [M+H]$^+$calcd for $C_{18}H_{18}ClFNO_3$, 350.0954, found: 350.0949.

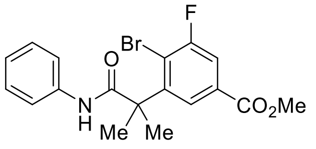

3ua

Methyl-4-bromo-3-fluoro-5-(2-methyl-1-oxo-1-(phenylamino)propan-2-yl)benzoate (3ua)

Compound **3ua** was prepared following **Procedure A**, starting from 2-bromo-2-methyl-*N*-phenylpropanamide (72.3 mg, 0.3 mmol) and 4-bromo-3-fluorobenzoic acid (130.8 mg, 0.6 mmol). After purification, **3ua** was obtained as colorless liquid (38.9 mg, 33% yield); **^{1}H NMR** (400 MHz, CDCl$_3$) δ 8.00 (s, 1H), 7.66 (dd, J = 8.1, 1.9 Hz, 1H), 7.40 (d, J = 7.8 Hz, 2H), 7.31 – 7.23 (m, 3H), 7.08 (t, J = 7.4 Hz, 1H), 3.89 (s, 3H), 1.75 (s, 6H); **^{13}C NMR** (125 MHz, CDCl$_3$) δ 173.5 (C$_q$), 165.2 (d, J = 2.5 Hz, C$_q$), 160.4 (C$_q$), 158.4 (C$_q$), 146.1 (C$_q$), 137.7 (C$_q$), 130.6 (d, J = 7.5 Hz, CH), 128.7 (CH), 124.4 (t, J = 7.5 Hz, CH), 120.4 (CH), 117.4 (d, J = 21.3 Hz, CH), 116.0 (d, J = 26.3 Hz, C$_q$), 52.6 (CH$_3$), 49.5 (C$_q$), 26.4 (CH$_3$); **^{19}F NMR** (375 MHz, CDCl$_3$) δ -99.68 (d, J = 7.9 Hz); **ESI-HRMS (m/z)**: [M+H]$^+$calcd for $C_{18}H_{18}BrFNO_3$, 394.0449, found: 394.0444.

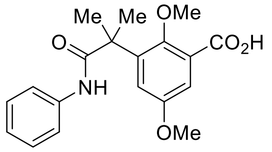

3va

2,5-Dimethoxy-3-(2-methyl-1-oxo-1-(phenylamino)propan-2-yl)benzoic acid (3va)

Compound **3va** was prepared following **Procedure A**, starting from 2-bromo-2-methyl-*N*-phenylpropanamide (72 mg, 0.3 mmol) and 2,5-dimethoxybenzoic acid (109 mg, 0.6 mmol). Methylation step did not run, and product was isolated as acid. After purification, **3va** was obtained as white solid (82.3 mg, 80% yield); **MP** = 230 – 233 ºC ; **^{1}H NMR** (500 MHz, DMSO-d$_6$) δ 8.84 (s, 1H), 7.65 (d, J = 8.1 Hz, 2H), 7.25 (t, J = 7.9 Hz, 2H), 7.13 (dd, J = 6.8, 3.0 Hz, 2H), 7.00 (t, J = 7.4 Hz, 1H), 3.81 (s, 3H), 3.53 (s, 3H), 1.52 (s, 6H); **^{13}C NMR** (125 MHz, DMSO-d$_6$) δ 175.5 (C$_q$), 168.2 (C$_q$), 154.2 (C$_q$), 152.2 (C$_q$), 141.1 (C$_q$), 140.1 (CH), 128.8 (CH), 125.5 (CH), 123.3 (C$_q$), 120.6 (CH), 118.2 (CH), 113.0 (C$_q$), 61.6 (CH$_3$), 55.9 (CH$_3$), 46.2 (C$_q$), 26.8 (CH$_3$); **ESI-HRMS** (m/z): [M+H]$^+$calcd for $C_{19}H_{22}NO_5$, 344.1492, found: 344.1486.

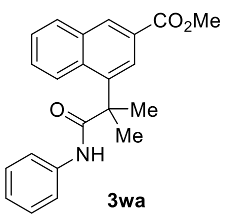

3wa

Methyl-4-(2-methyl-1-oxo-1-(phenylamino)propan-2-yl)-2-naphthoate (3wa)

Compound **3wa** was prepared following **Procedure A**, starting from 2-bromo-2-methyl-*N*-phenylpropanamide (72 mg, 0.3 mmol) and 2-naphthoic acid (103 mg, 0.6 mmol). After purification, **3wa** was obtained as white solid (61.4 mg, 59% yield); **MP** = 165 – 167 ºC ; **^{1}H NMR** (500 MHz, $CDCl_3$) δ 8.58 (s, 1H), 8.27 (d, *J* = 1.4 Hz, 1H), 8.08 (d, *J* = 8.4 Hz, 1H), 7.98 (d, *J* = 7.8 Hz, 1H), 7.61 – 7.52 (m, 2H), 7.25 – 7.18 (m, 4H), 7.06 – 6.98 (m, 1H), 6.86 (s, 1H), 3.97 (s, 3H), 1.87 (s, 6H); **^{13}C NMR** (125 MHz, $CDCl_3$) δ 176.3 (C_q), 167.1 (C_q), 140.6 (CH), 137.7 (C_q), 133.7 (C_q), 133.3 (C_q), 132.1 (C_q), 130.8 (CH), 129.0 (CH), 128.8 (CH), 126.6 (C_q), 125.0 (CH), 124.3 (CH), 123.3 (CH), 120.1 (CH), 52.4 (CH_3), 48.0 (C_q), 27.7 (CH_3); **ESI-HRMS** (m/z): $[M+H]^+$calcd for $C_{22}H_{22}NO_3$, 348.1594, found: 348.1588.

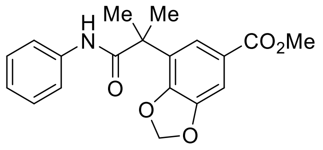

3xa

Methyl-7-(2-methyl-1-oxo-1-(phenylamino)propan-2-yl)benzo[d][1,3]dioxole-5-carboxylate (3xa)

Compound **3xa** was prepared following **Procedure A**, starting from 2-bromo-2-methyl-*N*-phenylpropanamide (72 mg, 0.3 mmol) and benzo[d][1,3]dioxole-5-carboxylic acid (100 mg, 0.6 mmol). After purification, **3xa** was obtained as yellow solid (45 mg, 44% yield); **MP** = 142 – 144 ºC ; **^{1}H NMR** (500 MHz, $CDCl_3$) δ 7.67 (s, 1H), 7.37 (s, 1H), 7.33 (d, *J* = 8.1 Hz, 2H), 7.20 (t, *J* = 7.4 Hz, 2H), 7.01 (d, *J* = 6.4 Hz, 2H), 5.93 (s, 2H), 3.82 (s, 3H), 1.60 (s, 6H); **^{13}C NMR** (125 MHz, $CDCl_3$) δ 173.9 (C_q), 166.3 (C_q), 149.2 (C_q), 148.0 (C_q), 137.9 (CH), 128.9 (CH), 126.1 (CH), 124.4 (C_q), 124.2 (CH), 122.4 (C_q), 119.9 (CH), 109.2 (C_q), 102.1 (CH_2), 52.20 (CH_3), 45.6 (C_q), 25.3 (CH_3); **ESI-HRMS** (m/z): $[M+H]^+$calcd for $C_{19}H_{20}NO_5$, 342.1336, found: 342.1329.

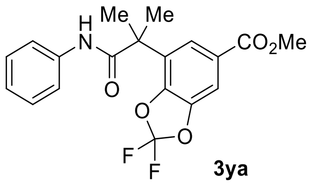

3ya

Methyl-2,2-difluoro-7-(2-methyl-1-oxo-1-(phenylamino)propan-2-yl)benzo[d][1,3]dioxole-5-carboxylate (3ya)

Compound **3ya** was prepared following **Procedure A**, starting from 2-bromo-2-methyl-*N*-phenylpropanamide (72 mg, 0.3 mmol) and 2,2-difluorobenzo[d][1,3]dioxole-5-carboxylic acid (121 mg, 0.6 mmol). After purification, **3ya** was obtained as white solid (59.9 mg, 53% yield); **MP** = 173 – 175 ºC; **^{1}H NMR** (500 MHz, $CDCl_3$) δ 7.93 (d, *J* = 1.6 Hz, 1H), 7.69 (d, *J* = 1.5 Hz, 1H), 7.41 – 7.36 (m, 2H), 7.33 – 7.27 (m, 2H), 7.16 – 7.05 (m, 2H), 3.92 (s, 3H), 1.74 (s, 6H); **^{13}C NMR** (125 MHz, $CDCl_3$) δ 172.6 (C_q), 165.4 (C_q), 144.7 (C_q), 143.9 (C_q), 137.3 (C_q), 131.4 (t, J_{C-F} = 256.3 Hz, CH), 128.9 (CH), 127.8 (CH), 126.5 (CH), 124.8 (C_q), 124.0 (CH), 120.6 (C_q), 110.2 (CH), 52.6 (CH_3), 45.8 (C_q), 25.4 (CH_3); **^{19}F NMR** (471 MHz, $CDCl_3$) δ -49.2; **ESI-HRMS** (m/z): $[M+H]^+$calcd for $C_{19}H_{18}F_2NO_5$, 378.1148, found: 378.1141.

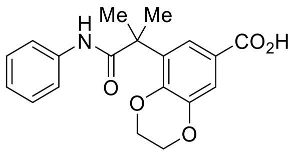

3za

8-(2-Methyl-1-oxo-1-(phenylamino)propan-2-yl)-2,3-dihydrobenzo[b][1,4]dioxine-6-carboxylic acid (3za)

Compound **3za** was prepared following **Procedure A**, starting from 2-bromo-2-methyl-*N*-phenylpropanamide (72 mg, 0.3 mmol) and

2,3-dihydrobenzo[b][1,4]dioxine-6-carboxylic acid (108 mg, 0.6 mmol). Methylation step did not run, and product was isolated as acid. After purification, **3za** was obtained as white solid (76.7 mg, 72% yield); **MP** = 240 – 243 ºC; **^{1}H NMR** (500 MHz, DMSO-d_6) δ 8.82 (s, 1H), 7.62 (s, 1H), 7.51 (d, *J* = 8.0 Hz, 2H), 7.37 (s, 1H), 7.25 (t, *J* = 7.6 Hz, 2H), 7.00 (t, *J* = 7.3 Hz, 1H), 4.17 (d, *J* = 25.6 Hz, 4H), 1.53 (s, 6H); **^{13}C NMR** (125 MHz, DMSO-d_6) δ 175.2 (C_q), 167.6 (C_q), 163.6 (C_q), 145.7 (C_q), 143.5 (C_q), 139.9 (CH), 134.5 (CH), 128.7 (CH), 123.5 (C_q), 123.4 (CH), 121.2 (CH), 121.1 (C_q), 117.5 (CH), 64.6 (CH_2), 64.2 (CH_2), 45.5 (C_q), 26.0 (CH_3); **ESI-HRMS** (m/z): $[M+H]^+$calcd for $C_{19}H_{20}NO_5$, 342.1336, found: 342.1328.

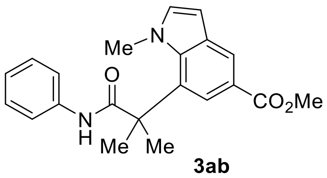

3ab

Methyl-1-methyl-7-(2-methyl-1-oxo-1-(phenylamino)propan-2-yl)-1H-indole-5-carboxylate (3ab)

Compound **3ab** was prepared following **Procedure A**, starting from 2-bromo-2-methyl-*N*-phenylpropanamide (72.3 mg, 0.3 mmol) and 1-methyl-1H-indole-5-carboxylic acid (105 mg, 0.6 mmol). After purification, **3ab** was obtained as colorless liquid (96.6 mg, 92% yield); **^{1}H NMR** (400 MHz, $CDCl_3$) δ 8.35 (d, *J* = 1.6 Hz, 1H), 8.11 (d, *J* = 1.7 Hz, 1H), 7.43 – 7.33 (m, 3H), 7.30 – 7.22 (m, 2H), 7.12 – 7.02 (m, 2H), 6.66 (d, *J* = 3.2 Hz, 1H), 3.89 (s, 3H), 3.89 (d, *J* = 4.0 Hz, 3H), 1.86 (s, 6H); **^{13}C NMR** (100 MHz, $CDCl_3$) δ 176.4 (C_q), 167.7 (C_q), 137.6 (C_q), 136.4 (C_q), 133.5 (CH), 131.3 (CH), 128.8 (CH), 127.6 (C_q), 124.5 (CH), 124.3 (C_q), 122.2 (CH), 121.1 (C_q), 119.8 (CH), 104.1 (CH), 51.8 (CH_3), 47.4 (C_q), 37.8 (CH_3), 28.6 (CH_3); **ESI-HRMS (m/z):** $[M+H]^+$calcd for $C_{21}H_{23}N_2O_3$, 351.1703, found: 351.1699.

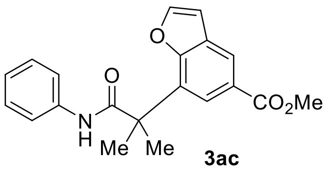

3ac

Methyl-7-(2-methyl-1-oxo-1-(phenylamino)propan-2-yl)benzofuran-5-carboxylate (3ac)

Compound **3ac** was prepared following **Procedure A**, starting from 2-bromo-2-methyl-*N*-phenylpropanamide (72.3 mg, 0.3 mmol) and benzofuran-5-carboxylic acid (97.2 mg, 0.6 mmol). After purification, **3ac** was obtained as colorless liquid (54.6 mg, 54% yield); **^{1}H NMR** (400 MHz, $CDCl_3$) δ 8.31 (d, *J* = 1.6 Hz, 1H), 8.11 (d, *J* = 1.6 Hz, 1H), 7.69 (d, *J* = 2.2 Hz, 1H), 7.38 – 7.31 (m, 2H), 7.28 – 7.21 (m, 2H), 7.10 – 7.01 (m, 2H), 6.84 (d, *J* = 2.2 Hz, 1H), 3.95 (s, 3H), 1.83 (s, 6H); **^{13}C NMR** (100 MHz, $CDCl_3$) δ 174.4 (C_q), 167.1 (C_q), 155.1 (C_q), 146.5 (CH), 137.9 (C_q), 128.7 (CH), 128.2 (CH), 125.5 (C_q), 124.1 (CH), 123.5 (CH), 122.9 (CH), 120.0 (C_q), 107.0 (CH), 52.2 (CH_3), 46.0 (C_q), 25.6 (CH_3); **ESI-HRMS (m/z)**: $[M+H]^+$calcd for $C_{20}H_{20}NO_4$, 338.1387, found: 338.1384.

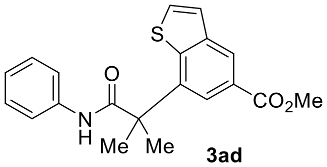

3ad

Methyl-7-(2-methyl-1-oxo-1-(phenylamino)propan-2-yl)benzo[b]thiophene-5-carboxylate (3ad)

Compound **3ad** was prepared following **Procedure A**, starting from 2-bromo-2-methyl-*N*-phenylpropanamide (72.3 mg, 0.3 mmol) and benzo[b]thiophene-5-carboxylic acid (106.8 mg, 0.6 mmol). After purification, **3ad** was obtained as colorless liquid (45.5 mg, 43% yield); **^{1}H NMR** (400 MHz, $CDCl_3$) δ 8.50 (d, *J* = 1.3 Hz, 1H), 8.17 (d, *J* = 1.5 Hz, 1H), 7.53 (d, *J* = 5.5 Hz, 1H), 7.44 (d, *J* = 5.5 Hz, 1H), 7.33 (d, *J* = 7.7 Hz, 2H), 7.25 (t, *J* = 7.9 Hz, 2H), 7.06 (t, *J* = 7.3 Hz, 1H), 6.97 (s, 1H), 3.97 (s, 3H), 1.88 (s, 6H); **^{13}C NMR** (100 MHz, $CDCl_3$) δ 174.0 (C_q), 167.3 (C_q), 142.8 (C_q), 140.8 (C_q), 138.9 (C_q), 137.8 (C_q), 128.8 (CH), 128.2 (CH), 126.8 (CH), 125.3 (CH), 124.5 (C_q), 124.3 (CH), 121.9 (CH), 120.1 (CH), 52.3 (CH_3), 48.5 (C_q), 25.7 (CH_3); **ESI-HRMS (m/z)**: $[M+H]^+$calcd for $C_{20}H_{20}SNO_3$, 354.1158, found: 354.1154.

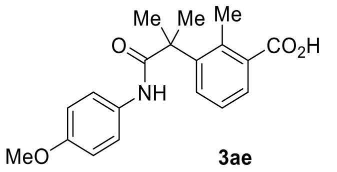

3ae

3-(1-((4-Methoxyphenyl)amino)-2-methyl-1-oxopropan-2-yl)-2-methylbenzoic acid (3ae)

Compound **3ae** was prepared following **Procedure A**, starting from 2-bromo-*N*-(4-methoxyphenyl)-2-methylpropanamide (81 mg, 0.3 mmol) and 2-methylbenzoic acid (82 mg, 0.6 mmol). Methylation step did not run, and product was isolated as acid. After purification, **3ae** was obtained as white solid (47.1 mg, 48% yield); **MP** = 165 –167 ºC ; **^{1}H NMR** (500 MHz, DMSO-*d*$_6$) δ 12.89 (s, 1H), 8.87 (s, 1H), 7.64 (d, *J* = 7.8 Hz, 1H), 7.58 (d, *J* = 7.6 Hz, 1H), 7.42 (d, *J* = 9.0 Hz, 2H), 7.32 (t, *J* = 7.8 Hz, 1H), 6.85 (d, *J* = 9.0 Hz, 2H), 3.70 (s, 3H), 2.35 (s, 3H), 1.56 (s, 6H); **^{13}C NMR** (125 MHz, DMSO-d$_6$) δ 176.1 (C$_q$), 170.6 (C$_q$), 155.9 (C$_q$), 144.9 (C$_q$), 136.2 (C$_q$), 134.7 (C$_q$), 132.6 (CH), 129.6 (C$_q$), 127.9 (CH), 126.1 (CH), 122.7 (CH), 114.0 (CH), 55.6 (CH$_3$), 47.7 (C$_q$), 27.8 (CH$_3$), 17.3 (CH$_3$); **ESI-HRMS** (m/z): [M-H]$^+$calcd for $C_{19}H_{20}NO_4$, 326.1398, found: 326.1397.

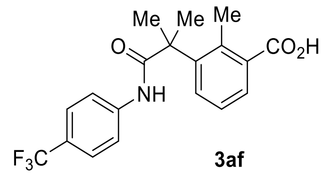

3af

2-Methyl-3-(2-methyl-1-oxo-1-((4-(trifluoromethyl)phenyl)amino)propan-2-yl)benzoic acid (3af)

Compound **3af** was prepared following **Procedure A**, starting from 2-bromo-2-methyl-*N*-(4-(trifluoromethyl)phenyl)propanamide (93 mg, 0.3 mmol) and 2-methylbenzoic acid (82 mg, 0.6 mmol). Methylation step did not run, and product was isolated as acid. After purification, **3af** was obtained as brown liquid (76.7 mg, 70% yield); **^{1}H NMR** (500 MHz, DMSO-*d*$_6$) δ 12.90 (s, 1H), 9.42 (s, 1H), 7.83 (d, *J* = 8.6 Hz, 2H), 7.67 (dd, *J* = 13.2, 8.4 Hz, 3H), 7.62 (d, *J* = 7.6 Hz, 1H), 7.37 (t, *J* = 7.8 Hz, 1H), 2.34 (s, 3H), 1.60 (s, 6H); **^{13}C NMR** (125 MHz, DMSO-d$_6$) δ 177.1 (C$_q$), 170.5 (C$_q$), 144.3 (C$_q$), 143.3 (C$_q$), 136.0 (C$_q$), 134.8 (C$_q$), 132.0 (d, $J_{C\text{-}F}$ = 23 Hz, C$_q$), 130.8 (d, $J_{C\text{-}F}$ = 36 Hz, CH), 129.7 (CH), 128.1 (CH), 126.2 (q, $J_{C\text{-}F}$ = 4 Hz, CH), 123.9 (q, $J_{C\text{-}F}$ = 33 Hz, C$_q$), 120.6 (CH), 48.1 (C$_q$), 27.6 (CH$_3$), 17.2 (CH$_3$); **^{19}F NMR** (376 MHz, CDCl$_3$) δ -62.23 (s); **ESI-HRMS** (m/z): [M-H]$^+$calcd for $C_{19}H_{17}F_3NO_3$, 364.1166, found: 364.1165.

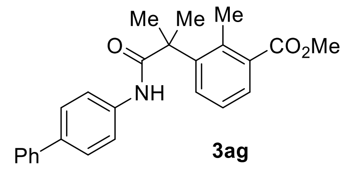

3ag

Methyl-3-(1-([1,1'-biphenyl]-4-ylamino)-2-methyl-1-oxopropan-2-yl)-2-methylbenzoate (3ag)

Compound **3ag** was prepared following **Procedure A**, starting from *N*-([1,1'-biphenyl]-4-yl)-2-bromo-2-methylpropanamide (95 mg, 0.3 mmol) and 2-methylbenzoic acid (82 mg, 0.6 mmol). After purification, **3ag** was obtained as colorless liquid (65 mg, 56% yield); **^{1}H NMR** (500 MHz, CDCl$_3$) δ 7.72 (dd, *J* = 7.7, 0.8 Hz, 1H), 7.67 (d, *J* = 7.9 Hz, 1H), 7.56 – 7.50 (m, 3H), 7.49 (d, *J* = 1.9 Hz, 1H), 7.44 – 7.37 (m, 4H), 7.36 – 7.28 (m, 2H), 6.93 (s, 1H), 3.87 (s, 3H), 2.47 (s, 3H), 1.68 (s, 6H); **^{13}C NMR** (125 MHz, CDCl$_3$) δ 176.1 (C$_q$), 168.9 (C$_q$), 143.3 (C$_q$), 140.4 (C$_q$), 137.8 (C$_q$), 137.1 (C$_q$), 137.1 (C$_q$), 133.6 (CH), 129.3 (C$_q$), 129.1 (CH), 128.7 (CH), 127.4 (CH), 127.0 (CH), 126.7 (CH), 126.1 (CH), 120.2 (CH), 52.1 (CH$_3$), 48.2 (C$_q$), 27.4 (CH$_3$), 17.7 (CH$_3$); **ESI-HRMS** (m/z): [M+H]$^+$calcd for $C_{25}H_{26}NO_3$, 388.1907, found: 388.1900.

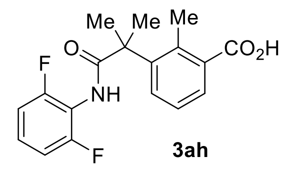

3ah

3-(1-((2,6-Difluorophenyl)amino)-2-methyl-1-oxopropan-2-yl)-2-methylbenzoic acid (3ah)

Compound **3ah** was prepared following **Procedure A**, starting from 2-bromo-*N*-(2,6-difluorophenyl)-2-methylpropanamide (83 mg, 0.3

mmol) and 2-methylbenzoic acid (82 mg, 0.6 mmol). Methylation step did not run, and product was isolated as acid. After purification, **3ah** was obtained as brown solid (77.9 mg, 78% yield); **MP** = 113 – 115 ºC; **^{1}H NMR** (500 MHz, DMSO-d$_6$) δ 8.87 (s, 1H), 7.64 (d, *J* = 7.9 Hz, 1H), 7.60 (d, *J* = 7.6 Hz, 1H), 7.35 – 7.29 (m, 2H), 7.10 (dd, *J* = 13.9, 5.8 Hz, 2H), 2.42 (s, 3H), 1.58 (s, 6H); **^{13}C NMR** (125 MHz, DMSO-d$_6$) δ 176.9 (C$_q$), 170.6 (C$_q$), 159.9 (d, J_{C-F} = 5 Hz, C$_q$), 158.0 (d, J_{C-F} = 5 Hz, C$_q$), 144.2 (C$_q$), 136.6 (C$_q$), 134.6 (C$_q$), 129.6 (CH), 128.7 (t, J_{C-F} = 10 Hz, CH), 128.2 (CH), 126.1 (CH), 115.6 (t, J_{C-F} = 18 Hz, CH), 112.2 (d, J_{C-F} = 5 Hz, C$_q$), 112.1 (d, J_{C-F} = 5 Hz, CH), 47.6 (C$_q$), 27.9 (CH$_3$), 17.5 (CH$_3$); **^{19}F NMR** (471 MHz, CDCl$_3$) δ -118.14 (t, *J* = 9.4 Hz); **ESI-HRMS** (m/z): [M-H]$^+$calcd for $C_{18}H_{16}F_2NO_3$, 332.1104, found: 332.1104.

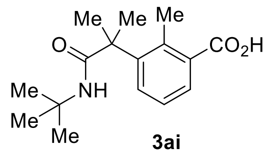

3ai

3-(1-(Tert-butylamino)-2-methyl-1-oxopropan-2-yl)-2-methylbenzoic acid (3ai)

Compound **3ai** was prepared following **Procedure A**, starting from 2-bromo-*N*-(tert-butyl)-2-methylpropanamide (66 mg, 0.3 mmol) and 2-methylbenzoic acid (82 mg, 0.6 mmol). Methylation step did not run, and product was isolated as acid. After purification, **3ai** was obtained as white solid (29.9 mg, 36% yield); **MP** = 131 – 133 ºC; **^{1}H NMR** (500 MHz, DMSO-d$_6$) δ 7.53 (t, *J* = 7.3 Hz, 2H), 7.26 (t, *J* = 7.7 Hz, 1H), 6.03 (s, 1H), 2.32 (s, 3H), 1.43 (s, 6H), 1.21 (s, 9H); **^{13}C NMR** (125 MHz, DMSO-d$_6$) δ 176.6 (C$_q$), 170.5 (C$_q$), 145.2 (C$_q$), 136.3 (C$_q$), 134.5 (C$_q$), 129.3 (CH), 127.8 (CH), 125.9 (CH), 50.7 (C$_q$), 47.6 (C$_q$), 28.6 (CH$_3$), 27.8 (CH$_3$), 17.8 (CH$_3$); **ESI-HRMS** (m/z): [M-H]$^+$calcd for $C_{16}H_{22}NO_3$, 276.1605, found: 276.1603.

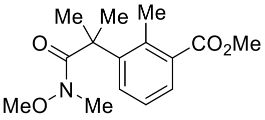

3aj

Methyl-3-(1-(methoxy(methyl)amino)-2-methyl-1-oxopropan-2-yl)-2-methylbenzoate (3aj)

Compound **3aj** was prepared following **Procedure A**, starting from 2-bromo-*N*-methoxy-*N*,2-dimethylpropanamide (62.7 mg, 0.3 mmol) and 2-methylbenzoic acid (81.6 mg, 0.6 mmol). After purification, **3aj** was obtained as colorless liquid (26.8 mg, 32% yield); **^{1}H NMR** (400 MHz, CDCl$_3$) δ 7.58 (d, *J* = 7.6 Hz, 1H), 7.52 (d, *J* = 7.9 Hz, 1H), 7.27 (t, *J* = 7.8 Hz, 1H), 3.89 (s, 3H), 3.12 (s, 3H), 2.64 (s, 3H), 2.42 (s, 3H), 1.57 (s, 6H); **^{13}C NMR** (100 MHz, CDCl$_3$) δ 178.3 (C$_q$), 169.4 (C$_q$), 145.2 (C$_q$), 135.8 (C$_q$), 132.9 (C$_q$), 128.2 (CH), 127.5 (CH), 125.8 (CH), 58.8 (CH$_3$), 52.0 (CH$_3$), 46.7 (C$_q$), 33.3 (CH$_3$), 26.4 (CH$_3$), 17.2 (CH$_3$); **ESI-HRMS (m/z)**: [M+H]$^+$calcd for $C_{15}H_{22}NO_4$, 280.1543, found: 280.1541.

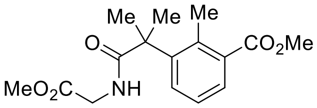

3ak

Methyl-3-(1-((2-methoxy-2-oxoethyl)amino)-2-methyl-1-oxopropan-2-yl)-2-methylbenzoate (3ak)

Compound **3ak** was prepared following **Procedure B**, starting from methyl-(2-bromo-2-methylpropanoyl)glycinate (71.1 mg, 0.3 mmol) and 2-methylbenzoic acid (81.6 mg, 0.6 mmol). After purification, **3ak** was obtained as colorless liquid (52.5 mg, 57% yield); **^{1}H NMR** (400 MHz, CDCl$_3$) δ 7.69 – 7.64 (m, 1H), 7.60 (dd, *J* = 8.0, 1.4 Hz, 1H), 7.27 (t, *J* = 7.8 Hz, 1H), 5.66 (t, *J* = 5.7 Hz, 1H), 3.95 (d, *J* = 5.5 Hz, 2H), 3.88 (s, 3H), 3.68 (s, 3H), 2.38 (s, 3H), 1.60 (s, 6H); **^{13}C NMR** (100 MHz, CDCl$_3$) δ 178.1 (C$_q$), 170.1 (C$_q$), 169.0 (C$_q$), 143.3 (C$_q$), 137.8 (C$_q$), 133.4 (CH), 129.2 (CH), 128.8 (C$_q$), 125.8 (CH), 52.1 (CH$_3$), 52.0 (CH$_3$), 47.2 (C$_q$), 41.3 (CH$_2$), 27.4 (CH$_3$), 17.9 (CH$_3$); **ESI-HRMS (m/z)**: [M+H]$^+$calcd for $C_{16}H_{22}NO_5$, 308.1492, found: 308.1490.

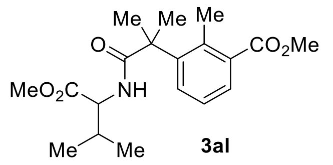

Methyl-3-(1-((1-methoxy-3-methyl-1-oxobutan-2-yl)amino)-2-methyl-1-oxopropan-2-yl)-2-methylbenzoate (3al)

Compound **3al** was prepared following **Procedure B**, starting from methyl-(2-bromo-2-methylpropanoyl)valinate (83.7 mg, 0.3 mmol) and 2-methylbenzoic acid (81.6 mg, 0.6 mmol). After purification, **3al** was obtained as colorless liquid (61.8 mg, 59% yield); **^{1}H NMR** (400 MHz, $CDCl_3$) δ 7.69 (d, *J* = 7.6 Hz, 1H), 7.62 (d, *J* = 7.6 Hz, 1H), 7.30 (t, *J* = 7.8 Hz, 1H), 5.55 (d, *J* = 8.6 Hz, 1H), 4.49 (dd, *J* = 8.6, 5.0 Hz, 1H), 3.88 (s, 3H), 3.67 (s, 3H), 2.38 (s, 3H), 2.12 – 2.01 (m, 1H), 1.60 (d, *J* = 8.2 Hz, 6H), 0.85 (d, *J* = 6.8 Hz, 3H), 0.72 (d, *J* = 6.9 Hz, 3H); **^{13}C NMR** (100 MHz, $CDCl_3$) δ 177.8 (C_q), 172.1 (C_q), 168.9 (C_q), 143.4 (C_q), 137.8 (C_q), 133.3 (CH), 129.2 (CH), 128.9 (C_q), 125.9 (CH), 57.2 (CH), 52.0 (CH_3), 52.0 (CH_3), 47.4 (C_q), 30.9 (CH), 27.5 (CH_3), 27.3 (CH_3), 18.9 (CH_3), 18.0 (CH_3), 17.6 (CH_3); **ESI-HRMS (m/z)**: $[M+H]^+$calcd for $C_{19}H_{28}NO_5$, 350.1962, found: 350.1959.

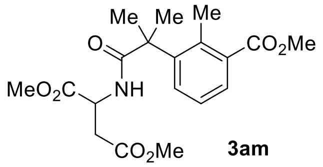

Dimethyl-(2-(3-(methoxycarbonyl)-2-methylphenyl)-2-methylpropanoyl)aspartate (3am)

Compound **3am** was prepared following **Procedure B**, starting from dimethyl-(2-bromo-2-methylpropanoyl)aspartate (92.7 mg, 0.3 mmol) and 2-methylbenzoic acid (81.6 mg, 0.6 mmol). After purification, **3am** was obtained as colorless liquid (92.1 mg, 81% yield); **^{1}H NMR** (400 MHz, $CDCl_3$) δ 7.68 (dd, *J* = 7.8, 1.3 Hz, 1H), 7.60 (dd, *J* = 7.9, 1.4 Hz, 1H), 7.29 (t, *J* = 7.9 Hz, 1H), 6.12 (d, *J* = 8.0 Hz, 1H), 4.82 – 4.76 (m, 1H), 3.88 (s, 3H), 3.69 (s, 3H), 3.58 (s, 3H), 2.94 (dd, *J* = 17.0, 4.8 Hz, 1H), 2.80 (dd, *J* = 17.0, 4.7 Hz, 1H), 2.35 (s, 3H), 1.61 (s, 3H), 1.56 (s, 3H); **^{13}C NMR** (125 MHz, $CDCl_3$) δ 177.4 (C_q), 171.0 (C_q), 170.8 (C_q), 168.8 (C_q), 143.1 (C_q), 137.6 (C_q), 133.1 (CH), 129.2 (CH), 128.8 (C_q), 125.8 (CH), 52.5 (CH), 51.9 (CH_3), 51.8 (CH_3), 48.6 (CH_3), 47.1 (C_q), 35.6 (CH_2), 27.4 (CH_3), 27.0 (CH_3), 17.7 (CH_3); **ESI-HRMS (m/z)**: $[M+H]^+$calcd for $C_{19}H_{26}NO_7$, 380.1704, found: 380.1702.

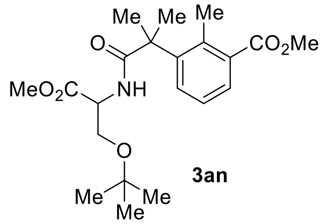

Methyl-3-(1-((3-(tert-butoxy)-1-methoxy-1-oxopropan-2-yl)amino)-2-methyl-1-oxopropan-2-yl)-2-methylbenzoate (3an)

Compound **3an** was prepared following **Procedure B**, starting from methyl *N*-(2-bromo-2-methylpropanoyl)-O-(tert-butyl)serinate (96.9 mg, 0.3 mmol) and 2-methylbenzoic acid (81.6 mg, 0.6 mmol). After purification, **3an** was obtained as colorless liquid (101.4 mg, 86% yield); **^{1}H NMR** (400 MHz, $CDCl_3$) δ 7.66 (d, *J* = 7.7 Hz, 1H), 7.60 (d, *J* = 7.3 Hz, 1H), 7.26 (t, *J* = 7.9 Hz, 1H), 5.93 (d, *J* = 8.2 Hz, 1H), 4.65 (dt, *J* = 8.3, 3.3 Hz, 1H), 3.85 (s, 3H), 3.72 – 3.67 (m, 1H), 3.66 (s, 3H), 3.42 (dd, *J* = 8.9, 3.4 Hz, 1H), 2.40 (s, 3H), 1.64 (s, 3H), 1.55 (s, 3H), 0.93 (s, 9H); **^{13}C NMR** (100 MHz, $CDCl_3$) δ 177.3 (C_q), 170.8 (C_q), 168.8 (C_q), 143.5 (C_q), 137.9 (C_q), 133.2 (CH), 129.2 (CH), 128.7 (C_q), 125.7 (CH), 73.0 (C_q), 61.4 (CH_2), 52.4 (CH), 52.1 (CH_3), 51.8 (CH_3), 47.2 (C_q), 27.8 (CH_3), 26.9 (CH_3), 26.7 (CH_3), 18.0 (CH_3); **ESI-HRMS (m/z)**: $[M+H]^+$calcd for $C_{21}H_{32}NO_6$, 394.2224, found: 394.2222.

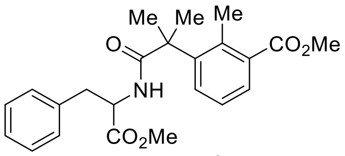

Methyl-3-(1-((1-methoxy-1-oxo-3-phenylpropan-2-yl)amino)-2-methyl-1-oxopropan-2-yl)-2 methylbenzoate (3ao)

Compound **3ao** was prepared following **Procedure B**, starting from methyl-(2-bromo-2-methylpropanoyl)phenylalaninate (98 mg, 0.3 mmol) and 2-methylbenzoic acid (82 mg, 0.6 mmol). After purification, **3ao** was obtained as grey liquid (72.7 mg, 61% yield); **^{1}H NMR** (500 MHz, $CDCl_3$) δ 7.69 (d, *J* = 7.7 Hz, 1H), 7.51 (d, *J* = 7.8 Hz, 1H), 7.27 (d, *J* = 5.4 Hz, 1H), 7.14 (d, *J* = 4.8 Hz, 3H), 6.89 – 6.84 (m, 2H), 5.41 (d, *J* = 7.8 Hz, 1H), 4.83 (dd, *J* = 13.2, 7.6 Hz, 1H), 3.90 (s, 3H), 3.68 (s, 3H), 3.03 (dd, *J* = 13.9, 5.3 Hz, 1H), 2.88 (dd, *J* = 13.9, 7.6 Hz, 1H), 2.21 (s, 3H), 1.56 (s, 3H), 1.45 (s, 3H); **^{13}C NMR** (125 MHz, $CDCl_3$) δ 177.5 (C_q), 171.8 (C_q), 168.8 (C_q), 143.2 (C_q), 138.0 (C_q), 135.6 (C_q), 133.2 (CH), 129.2 (CH), 128.9 (C_q), 128.7 (CH), 128.5 (CH), 126.9 (CH), 125.7(CH), 52.9 (CH), 52.1 (CH_3), 52.0 (CH_3), 47.2 (C_q), 37.6 (CH_2), 27.6 (CH_3), 26.7 (CH_3), 17.8 (CH_3); **ESI-HRMS** (m/z): $[M+H]^+$calcd for $C_{23}H_{28}NO_5$, 398.2326, found: 398.2318.

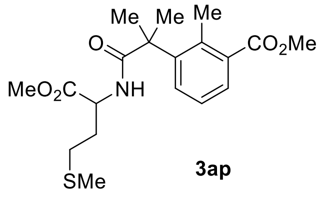

3ap

Methyl-3-(1-((1-methoxy-4-(methylthio)-1-oxobutan-2-yl)amino)-2-methyl-1-oxopropan-2-yl)-2-methylbenzoate (3ap)

Compound **3ap** was prepared following **Procedure B**, starting from methyl-(2-bromo-2-methylpropanoyl)methioninate (93.3 mg, 0.3 mmol) and 2-methylbenzoic acid (81.6 mg, 0.6 mmol). After purification, **3ap** was obtained as colorless liquid (60.6 mg, 53% yield); **^{1}H NMR** (400 MHz, $CDCl_3$) δ 7.68 (dd, *J* = 7.8, 1.3 Hz, 1H), 7.61 (dd, *J* = 7.9, 1.4 Hz, 1H), 7.29 (t, *J* = 7.8 Hz, 1H), 5.90 (d, *J* = 7.9 Hz, 1H), 4.71 – 4.66 (m, 1H), 3.89 (s, 3H), 3.70 (s, 3H), 2.39 (s, 3H), 2.37 (d, *J* = 7.3 Hz, 2H), 2.09 – 2.01 (m, 1H), 1.99 (s, 3H), 1.92 – 1.85 (m, 1H), 1.60 (d, *J* = 6.8 Hz, 6H); **^{13}C NMR** (125 MHz, $CDCl_3$) δ 177.8 (C_q), 172.1 (C_q), 168.9 (C_q), 143.3 (C_q), 137.7 (C_q), 133.4 (CH), 129.2 (CH), 128.8 (C_q), 125.9 (CH), 52.3 (CH), 52.0 (CH_3), 51.6 (CH_3), 47.3 (C_q), 30.9 (CH_2), 29.9 (CH_2), 27.4 (CH_3), 27.3 (CH_3), 18.0 (CH_3), 15.2 (CH_3); **ESI-HRMS (m/z):** $[M+H]^+$calcd for $C_{19}H_{28}SNO_5$, 382.1683, found: 382.1670.

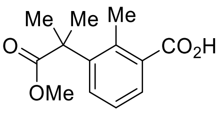

3aq

3-(1-Methoxy-2-methyl-1-oxopropan-2-yl)-2-methylbenzoic acid (3aq)

Compound **3aq** was prepared following **Procedure A**, starting from methyl-2-bromo-2-methylpropanoate (54 mg, 0.3 mmol) and 2-methylbenzoic acid (81.6 mg, 0.6 mmol). Methylation step did not run, and product was isolated as acid. After purification, **3aq** was obtained as colorless liquid (46 mg, 65% yield); **^{1}H NMR** (500 MHz, DMSO-*d*$_6$) δ 12.94 (s, 1H), 7.54 (t, *J* = 7.7 Hz, 2H), 7.27 (t, *J* = 7.8 Hz, 1H), 3.61 (s, 3H), 2.23 (s, 3H), 1.51 (s, 6H); **^{13}C NMR** (125 MHz, DMSO-*d*$_6$) δ 178.2 (C_q), 170.5 (C_q), 144.3 (C_q), 135.4 (C_q), 134.7 (C_q), 128.3 (CH), 127.9 (CH), 126.1 (CH), 52.7 (CH_3), 46.6 (C_q), 27.5 (CH_3), 17.1 (CH_3); **ESI-HRMS (m/z):** $[M-H]^+$calcd for $C_{13}H_{15}O_4$, 235.0976, found: 235.0971.

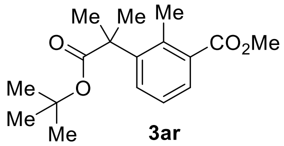

3ar

Methyl-3-(1-(tert-butoxy)-2-methyl-1-oxopropan-2-yl)-2-methylbenzoate (3ar)

Compound **3ar** was prepared following **Procedure A**, starting from tert-butyl-2-bromo-2-methylpropanoate (66.6 mg, 0.3 mmol) and 2-methylbenzoic acid (81.6 mg, 0.6 mmol). After purification, **3ar** was obtained as colorless liquid (80 mg, 81% yield); **^{1}H NMR** (400 MHz, $CDCl_3$) δ 7.65 (dd, *J* = 7.7, 1.4 Hz, 1H), 7.49 (dd, *J* = 8.0, 1.4 Hz, 1H), 7.22 (t, *J* = 7.8 Hz, 1H), 3.89 (s, 3H), 2.43 (s, 3H), 1.56 (s, 6H), 1.41 (s, 9H); **^{13}C NMR** (100 MHz, $CDCl_3$) δ 176.9 (C_q), 169.2 (C_q), 144.6 (C_q), 136.9 (C_q), 132.3 (CH), 128.5 (CH), 128.1 (C_q), 125.3 (CH), 80.5 (C_q), 51.9 (C_q), 47.3 (CH_3), 27.6 (CH_3), 27.2 (CH_3), 17.9 (CH_3); **ESI-HRMS (m/z)**: $[M-H]^+$calcd for $C_{17}H_{23}O_4$, 291.1602, found: 291.1607.

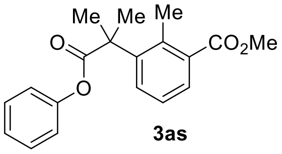

3as

Methyl-2-methyl-3-(2-methyl-1-oxo-1-phenoxypropan-2-yl)benzoate (3as)

Compound **3as** was prepared following **Procedure A**, starting from phenyl 2-bromo-2-methylpropanoate (72.6 mg, 0.3 mmol) and 2-methylbenzoic acid (81.6 mg, 0.6 mmol). After purification, **3as** was obtained as colorless liquid (75.8 mg, 81% yield); **^{1}H NMR** (400 MHz, $CDCl_3$) δ 7.74 (d, J = 7.7 Hz, 1H), 7.63 (d, J = 7.3 Hz, 1H), 7.42 – 7.35 (m, 2H), 7.32 (t, J = 7.8 Hz, 1H), 7.23 (t, J = 7.4 Hz, 1H), 7.08 – 7.01 (m, 2H), 3.93 (s, 3H), 2.60 (s, 3H), 1.78 (s, 6H); **^{13}C NMR** (100 MHz, $CDCl_3$) δ 176.5 (C_q), 169.0 (C_q), 150.8 (C_q), 143.6 (C_q), 136.7 (C_q), 132.7 (CH), 129.3 (CH), 128.6 (C_q), 128.5 (CH), 125.7 (CH), 125.7 (CH), 121.2 (CH), 52.0 (CH_3), 46.9 (C_q), 27.2 (CH_3), 17.7 (CH_3); **ESI-HRMS (m/z)**: $[M+H]^+$calcd for $C_{19}H_{21}O_4$, 313.1434, found: 313.1430.

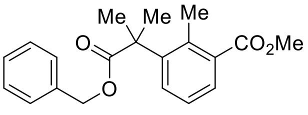

3at

Methyl-3-(1-(benzyloxy)-2-methyl-1-oxopropan-2-yl)-2-methylbenzoate (3at)

Compound **3at** was prepared following **Procedure A**, starting from benzyl-2-bromo-2-methylpropanoate (76.8 mg, 0.3 mmol) and 2-methylbenzoic acid (81.6 mg, 0.6 mmol). After purification, **3at** was obtained as colorless liquid (85.1 mg, 87% yield); **^{1}H NMR** (400 MHz, $CDCl_3$) δ 7.67 (d, J = 7.7 Hz, 1H), 7.54 (d, J = 7.3 Hz, 1H), 7.36 – 7.30 (m, 3H), 7.29 – 7.22 (m, 3H), 5.16 (s, 2H), 3.90 (s, 3H), 2.30 (s, 3H), 1.63 (s, 6H); **^{13}C NMR** (100 MHz, $CDCl_3$) δ 177.6 (C_q), 169.1 (C_q), 144.0 (C_q), 136.6 (C_q), 135.7 (C_q), 132.7 (CH), 128.4 (CH), 128.3 (C_q), 128.2 (CH), 128.01 (CH), 127.97 (CH), 125.5 (CH), 66.6(CH_2), 51.9 (CH_3), 46.6 (C_q), 27.2 (CH_3), 17.3 (CH_3); **ESI-HRMS (m/z)**: $[M+H]^+$calcd for $C_{20}H_{23}O_4$, 327.1590, found: 327.1588.

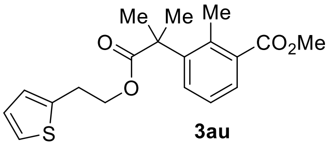

3au

Methyl-2-methyl-3-(2-methyl-1-oxo-1-(2-(thiophen-2-yl)ethoxy)propan-2-yl)benzoate (3au)

Compound **3au** was prepared following **Procedure A**, starting from 2-(thiophen-2-yl)ethyl-2-bromo-2-methylpropanoate (82.8 mg, 0.3 mmol) and 2-methylbenzoic acid (81.6 mg, 0.6 mmol). After purification, **3au** was obtained as colorless liquid (82 mg, 79% yield); **^{1}H NMR** (400 MHz, $CDCl_3$) δ 7.69 (d, J = 7.6 Hz, 1H), 7.54 (d, J = 7.9 Hz, 1H), 7.27 (t, J = 7.8 Hz, 1H), 7.11 (d, J = 5.1 Hz, 1H), 6.88 (dd, J = 5.2, 3.4 Hz, 1H), 6.68 (d, J = 3.3 Hz, 1H), 4.31 (t, J = 6.6 Hz, 2H), 3.90 (s, 3H), 3.09 (t, J = 6.6 Hz, 2H), 2.32 (s, 3H), 1.61 (s, 6H); **^{13}C NMR** (100 MHz, $CDCl_3$) δ 177.8 (C_q), 169.0 (C_q), 143.9 (C_q), 139.6 (C_q), 136.8 (C_q), 132.5 (CH), 128.4 (CH), 128.3 (CH), 126.7 (CH), 125.4 (CH), 123.8 (CH), 65.3 (CH_2), 51.9 (CH_3), 46.6 (C_q), 28.9 (CH_2), 27.2 (CH_3), 17.3 (CH_3); **ESI-HRMS (m/z)**: $[M+H]^+$calcd for $C_{19}H_{23}SO_4$, 347.1312, found: 347.1303.

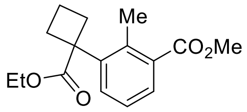

3av

Methyl-3-(1-(ethoxycarbonyl)cyclobutyl)-2-methylbenzoate (3av)

Compound **3av** was prepared following **Procedure A**, starting from ethyl-1-bromocyclobutane-1-carboxylate (61.8 mg, 0.3 mmol) and 2-methylbenzoic acid (81.6 mg, 0.6 mmol). After purification, **3av** was obtained as colorless liquid (64.6 mg, 78% yield); **^{1}H NMR** (400 MHz, $CDCl_3$) δ 7.66 (dd, J = 7.8, 1.4 Hz, 1H), 7.40 (dd, J = 7.8, 1.4 Hz, 1H), 7.24 (t, J = 7.8 Hz, 1H), 4.12 (q, J = 7.1 Hz, 2H), 3.88 (s, 3H), 2.89 – 2.82 (m, 2H), 2.61 – 2.49 (m, 2H), 2.33 (s, 3H), 2.28 – 2.14 (m, 1H), 1.95 – 1.80 (m, 1H), 1.17 (t, J = 7.1 Hz, 3H); **^{13}C NMR** (100 MHz, $CDCl_3$) δ 175.5 (C_q), 168.9 (C_q), 143.7 (C_q), 137.1 (C_q), 131.8 (CH), 130.5 (CH), 128.3 (C_q), 125.2 (CH), 61.0 (CH_2), 53.1

(CH_3), 51.9 (C_q), 32.9 (CH_2), 17.1 (CH_2), 16.8 (CH_3), 14.0 (CH_3); **ESI-HRMS (m/z)**: $[M+H]^+$calcd for $C_{16}H_{21}O_4$, 277.1434, found: 277.1429.

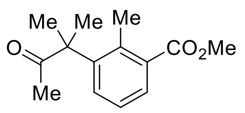

3aw

Methyl-2-methyl-3-(2-methyl-3-oxobutan-2-yl)benzoate (3aw)

Compound **3aw** was prepared following **Procedure A**, starting from 3-bromo-3-methylbutan-2-one (49.2 mg, 0.3 mmol) and 2-methylbenzoic acid (81.6 mg, 0.6 mmol). After purification, **3aw** was obtained as colorless liquid (20.4 mg, 29% yield); **^{1}H NMR** (400 MHz, $CDCl_3$) δ 7.66 (d, *J* = 7.7 Hz, 1H), 7.57 (d, *J* = 7.9 Hz, 1H), 7.29 (t, *J* = 7.8 Hz, 1H), 3.88 (s, 3H), 2.31 (s, 3H), 1.94 (s, 3H), 1.49 (s, 6H); **^{13}C NMR** (100 MHz, $CDCl_3$) δ 212.8 (C_q), 169.1 (C_q), 144.2 (C_q), 136.8 (C_q), 133.1 (CH), 128.9 (CH), 128.6 (C_q), 126.0 (CH), 53.2 (C_q), 52.1 (CH_3), 26.0 (CH_3), 25.7 (CH_3), 17.5 (CH_3); **ESI-HRMS (m/z)**: $[M+H]^+$calcd for $C_{14}H_{19}O_3$, 235.1329, found: 235.1326.

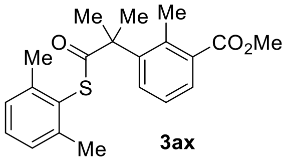

3ax

Methyl-3-(1-((2,6-dimethylphenyl)thio)-2-methyl-1-oxopropan-2-yl)-2-methylbenzoate (3ay)

Compound **3ay** was prepared following **Procedure A**, starting from *S*-(2,6-dimethylphenyl) 2-bromo-2-methylpropanethioate (85.8 mg, 0.3 mmol) and 2-methylbenzoic acid (81.6 mg, 0.6 mmol). After purification, **3ay** was obtained as colorless liquid (71.6 mg, 67% yield); **^{1}H NMR** (400 MHz, $CDCl_3$) δ 7.78 (dd, *J* = 7.7, 1.3 Hz, 1H), 7.68 (dd, *J* = 8.0, 1.4 Hz, 1H), 7.33 (t, *J* = 7.8 Hz, 1H), 7.20 (dd, *J* = 8.4, 6.5 Hz, 1H), 7.12 (d, *J* = 7.5 Hz, 2H), 3.91 (s, 3H), 2.55 (s, 3H), 2.32 (s, 6H), 1.72 (s, 6H); **^{13}C NMR** (100 MHz, $CDCl_3$) δ 203.0 (C_q), 168.9 (C_q), 143.0 (C_q), 142.6 (C_q), 138.6 (C_q), 132.8 (C_q), 129.9 (CH), 129.7 (C_q), 129.3 (CH), 128.2 (CH), 127.3 (CH), 125.7 (CH), 54.7 (C_q), 52.0 (CH_3), 27.7 (CH_3), 21.6 (CH_3), 19.3 (CH_3); **ESI-HRMS (m/z)**: $[M+H]^+$calcd for $C_{21}H_{25}SO_3$, 357.1519, found: 357.1515.

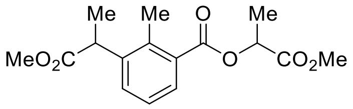

3ay

1-Methoxy-1-oxopropan-2-yl 3-(1-methoxy-1-oxopropan-2-yl)-2-methylbenzoate (3ay)

Compound **3ay** was prepared following **Procedure C**, starting from methyl-2-bromopropanoate (199 mg, 1.2 mmol) and 2-methylbenzoic acid (41 mg, 0.3 mmol). After purification, **3ay** was obtained as colorless liquid (40.6 mg, 45% yield); **^{1}H NMR** (500 MHz, $CDCl_3$) δ 7.76 – 7.72 (m, 1H), 7.43 – 7.40 (m, 1H), 7.29 – 7.24 (m, 1H), 5.31 (q, *J* = 7.1 Hz, 1H), 4.08 (q, *J* = 7.1 Hz, 1H), 3.79 (s, 3H), 3.66 (s, 3H), 2.55 (s, 3H), 1.61 (d, *J* = 7.1 Hz, 3H), 1.48 (d, *J* = 7.1 Hz, 3H); **^{13}C NMR** (125 MHz, $CDCl_3$) δ 174.9 (C_q), 171.3 (C_q), 167.8 (C_q), 140.6 (C_q), 136.6 (CH), 131.1 (C_q), 130.3 (CH), 128.9 (C_q), 125.9 (CH), 69.0 (CH), 52.4 (CH_3), 52.1 (CH_3), 41.1 (CH), 18.0 (CH_3), 16.9 (CH_3), 16.1 (CH_3); **ESI-HRMS** (m/z): $[M+H]^+$calcd for $C_{16}H_{21}O_6$, 309.1333, found: 309.1325.

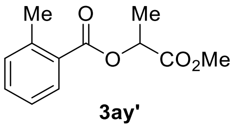

3ay'

Methoxy-1-oxopropan-2-yl 2-methylbenzoate (3ay')

Compound **3ay'** was prepared following **Procedure C**, starting from methyl-2-bromopropanoate (199 mg, 1.2 mmol) and 2-methylbenzoic acid (41 mg, 0.3 mmol). After purification, **3ay'** was obtained as colorless liquid (18.3 mg, 27% yield); **^{1}H NMR** (400 MHz, $CDCl_3$) δ 8.01 (dd, *J* = 8.3, 1.5 Hz, 1H), 7.48 – 7.40 (m, 1H), 7.28 (d, *J* = 7.3 Hz, 2H), 5.34 (q, *J* = 7.1 Hz, 1H), 3.81 (s, 3H), 2.63 (s, 3H), 1.64 (d, *J* = 7.1 Hz, 3H); **^{13}C NMR** (100 MHz, $CDCl_3$) δ 171.4 (C_q), 166.9 (C_q), 140.5 (C_q), 132.3 (CH), 131.7 (CH), 130.8 (C_q), 128.8 (CH), 125.7 (CH), 68.9 (CH), 52.4 (CH_3), 21.6 (CH_3), 17.1 (CH_3).

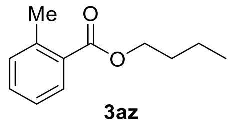

3az

Butyl-2-methylbenzoate (3az)

Compound **3ay** was prepared following **Procedure C**, starting from 1-bromobutane (163 mg, 1.2 mmol) and 2-methylbenzoic acid (41 mg, 0.3 mmol). After purification, **3az** was obtained as colorless liquid (18.4 mg, 32% yield); **^{1}H NMR** (400 MHz, $CDCl_3$) δ 7.93 (dd, *J* = 8.0, 1.5 Hz, 1H), 7.41 (td, *J* = 7.5, 1.5 Hz, 1H), 7.27 (dd, *J* = 8.0, 4.5 Hz, 2H), 4.33 (t, *J* = 6.6 Hz, 2H), 2.62 (s, 3H), 1.77 (dq, *J* = 8.6, 6.7 Hz, 2H), 1.55 – 1.45 (m, 2H), 1.01 (t, *J* = 7.4 Hz, 3H); **^{13}C NMR** (100 MHz, $CDCl_3$) δ 167.8 (C_q), 140.0 (C_q), 131.8 (CH), 131.7 (CH), 130.5 (C_q), 130.0 (CH), 125.7 (CH), 64.6 (CH_2), 30.8 (CH_2), 21.8 (CH_3), 19.4 (CH_2), 13.8 (CH_3).

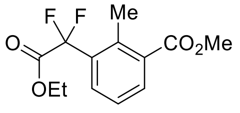

3bb

Methyl-3-(2-ethoxy-1,1-difluoro-2-oxoethyl)-2-methylbenzoate (3bb)

Compound **3bb** was prepared following **Procedure D**, starting from ethyl-2-bromo-2,2-difluoroacetate (39 uL, 0.3 mmol) and 2-methylbenzoic acid (81.6 mg, 0.6 mmol). After purification, **3bb** was obtained as colorless liquid (39.2 mg, 48% yield); **^{1}H NMR** (500 MHz, $CDCl_3$) δ 7.87 (d, *J* = 7.8 Hz, 1H), 7.77 (dd, *J* = 7.9, 1.4 Hz, 1H), 7.35 (t, *J* = 7.9 Hz, 1H), 4.33 (q, *J* = 7.1 Hz, 2H), 3.91 (s, 3H), 2.53 (s, 3H), 1.30 (t, *J* = 7.2 Hz, 3H); **^{13}C NMR** (125 MHz, $CDCl_3$) δ 168.1 (C_q), 163.9 (t, $J_{C\text{-}F}$ = 35 Hz, C_q), 137.4 (t, $J_{C\text{-}F}$ = 2.5 Hz, C_q), 132.9 (t, $J_{C\text{-}F}$ = 23.8 Hz, C_q), 132.3 (CH), 129.2 (t, $J_{C\text{-}F}$ = 8.8 Hz, CH), 125.7 (C_q), 113.7 (t, $J_{C\text{-}F}$ = 250 Hz, C_q), 63.3 (CH_2), 52.3 (CH_3), 16.7 (CH_3), 13.8 (CH_3); **^{19}F NMR** (471 MHz, $CDCl_3$) δ -100.19 (s); **ESI-HRMS** (m/z): $[M+H]^+$calcd for $C_{13}H_{15}F_2O_4$, 273.0933, found: 273.0932.

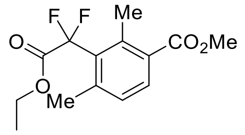

3cb

Methyl-3-(2-ethoxy-1,1-difluoro-2-oxoethyl)-2,4-dimethylbenzoate (3cb)

Compound **3cb** was prepared following **Procedure D**, starting from ethyl-2-bromo-2,2-difluoroacetate (39 uL, 0.3 mmol) and 2,4-dimethylbenzoic acid (90 mg, 0.6 mmol). After purification, **3cb** was obtained as colorless liquid (49.8 mg, 58% yield); **^{1}H NMR** (500 MHz, $CDCl_3$) δ 7.65 (d, *J* = 8.0 Hz, 1H), 7.10 (d, *J* = 8.1 Hz, 1H), 4.33 (q, *J* = 7.1 Hz, 2H), 3.88 (s, 3H), 2.52 (q, *J* = 3.1, 1.8 Hz, 6H), 1.31 (t, *J* = 7.1 Hz, 3H); **^{13}C NMR** (125 MHz, $CDCl_3$) δ 168.5 (C_q), 164.1 (t, $J_{C\text{-}F}$ = 33.8 Hz, C_q), 141.6 (t, $J_{C\text{-}F}$ = 2.5 Hz, C_q), 138.4 (t, $J_{C\text{-}F}$ = 3.8 Hz, C_q), 131.6 (d, $J_{C\text{-}F}$ = 22.5 Hz, C_q), 131.4 (CH), 131.2 (CH), 130.1 (C_q), 115.8 (t, $J_{C\text{-}F}$ = 252.5 Hz, C_q), 63.2 (CH_2), 52.1 (CH_3), 22.4 (CH_3), 18.1 (CH_3), 13.8 (CH_3); **^{19}F NMR** (471 MHz, $CDCl_3$) δ -93.6 (t, *J* = 12.5 Hz), -101.2 (s); **ESI-HRMS** (m/z): $[M+H]^+$calcd for $C_{14}H_{17}F_2O_4$, 287.1089, found: 287.1088.

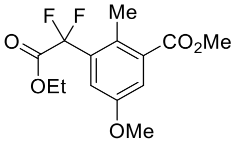

3db

Methyl-3-(2-ethoxy-1,1-difluoro-2-oxoethyl)-5-methoxy-2-methylbenzoate (3db)

Compound **3db** was prepared following **Procedure D**, starting from ethyl-2-bromo-2,2-difluoroacetate (39 uL, 0.3 mmol) and 5-methoxy-2-methylbenzoic acid (99.6 mg, 0.6 mmol). After purification, **3db** was obtained as colorless liquid (58.9 mg, 65% yield); **^{1}H NMR** (500 MHz, $CDCl_3$) δ 7.38 (d, *J* = 2.8 Hz, 1H), 7.31 (d, *J* = 2.8 Hz, 1H), 4.31 (q, *J* = 7.1 Hz, 2H), 3.89 (s, 3H), 3.83 (s, 3H), 2.41 (t, *J* = 1.7 Hz, 3H), 1.29 (t, *J* = 7.1 Hz, 3H); **^{13}C NMR** (125 MHz, $CDCl_3$) δ 167.9 (C_q), 163.7 (t, $J_{C\text{-}F}$ = 33.8 Hz, C_q), 157.0 (C_q), 134.0 (C_q), 133.8 (t, $J_{C\text{-}F}$ = 8.8 Hz, C_q), 128.8 (t, $J_{C\text{-}F}$ = 3.8 Hz, C_q), 117.2 (CH), 115.6 (t, $J_{C\text{-}F}$ = 10 Hz, CH), 113.4 (t, $J_{C\text{-}F}$ = 250 Hz, C_q), 63.3 (CH_2),

55.5 (CH_3), 52.2 (CH_3), 15.7 (CH_3), 13.8 (CH_3); **^{19}F NMR** (471 MHz, $CDCl_3$) δ -100.38 (s); **ESI-HRMS** (m/z): $[M+H]^+$calcd for $C_{14}H_{17}F_2O_5$, 303.1039, found: 303.1038.

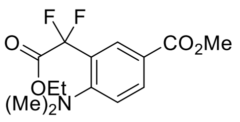

3eb

Methyl-4-(dimethylamino)-3-(2-ethoxy-1,1-difluoro-2-oxoethyl)benzoate (3eb)

Compound **3eb** was prepared following **Procedure D**, starting from ethyl-2-bromo-2,2-difluoroacetate (39 uL, 0.3 mmol) and 4-(dimethylamino)benzoic acid (99.0 mg, 0.6 mmol). After purification, **3eb** was obtained as colorless liquid (42.4 mg, 47% yield); **^{1}H NMR** (500 MHz, $CDCl_3$) δ 8.38 (d, J = 2.1 Hz, 1H), 8.14 (dd, J = 8.4, 2.1 Hz, 1H), 7.33 (d, J = 8.4 Hz, 1H), 4.27 (q, J = 7.1 Hz, 2H), 3.90 (s, 3H), 2.60 (s, 6H), 1.27 (t, J = 7.1 Hz, 3H); **^{13}C NMR** (125 MHz, $CDCl_3$) δ 166.0 (C_q), 163.7 (t, $J_{C\text{-}F}$ = 32.5 Hz, C_q), 156.7 (t, $J_{C\text{-}F}$ = 5.0 Hz, C_q), 133.2 (CH), 130.7 (t, $J_{C\text{-}F}$ = 25.0 Hz, CH), 128.2 (t, $J_{C\text{-}F}$ = 7.5 Hz, C_q), 126.8 (C_q), 122.3 (CH), 112.0 (t, $J_{C\text{-}F}$ = 250 Hz, C_q), 62.3 (CH_2), 52.2 (CH_3), 45.1 (CH_3), 14.0 (CH_3); **^{19}F NMR** (471 MHz, $CDCl_3$) δ -97.97 (s); **ESI-HRMS** (m/z): $[M+H]^+$calcd for $C_{14}H_{18}F_2NO_4$, 302.1198, found: 302.1197.

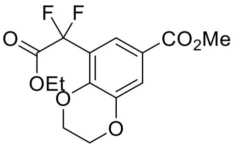

3fb

Methyl-8-(2-ethoxy-1,1-difluoro-2-oxoethyl)-2,3-dihydrobenzo[b][1,4]dioxine-6-carboxylate (3fb)

Compound **3fb** was prepared following **Procedure D**, starting from ethyl-2-bromo-2,2-difluoroacetate (39 uL, 0.3 mmol) and 2,3-dihydrobenzo[b][1,4]dioxine-6-carboxylic acid (108 mg, 0.6 mmol). After purification, **3fb** was obtained as colorless liquid (70.2 mg, 74% yield); **^{1}H NMR** (500 MHz, $CDCl_3$) δ 7.90 (d, J = 2.1 Hz, 1H), 7.67 (d, J = 2.1 Hz, 1H), 4.33 (t, J = 7.1 Hz, 2H), 4.30 – 4.26 (m, 4H), 3.88 (s, 3H), 1.29 (t, J = 7.1 Hz, 3H); **^{13}C NMR** (125 MHz, $CDCl_3$) δ 165.8 (C_q), 163.3 (t, $J_{C\text{-}F}$ = 33.8 Hz, C_q), 145.1 (t, $J_{C\text{-}F}$ = 5.0 Hz, C_q), 143.4 (C_q), 123.1 (CH), 121.8 (t, $J_{C\text{-}F}$ = 25 Hz, C_q), 121.4 (C_q), 120.3 (t, $J_{C\text{-}F}$ = 7.5 Hz, CH), 111.5 (t, $J_{C\text{-}F}$ = 247.5 Hz, C_q), 64.7 (CH_2), 63.8 (CH_2), 62.9 (CH_2), 52.2 (CH_3), 13.9 (CH_3); **^{19}F NMR** (471 MHz, $CDCl_3$) δ -102.50 (s); **ESI-HRMS** (m/z): $[M+H]^+$calcd for $C_{14}H_{15}F_2O_6$, 317.0831, found: 317.0828.

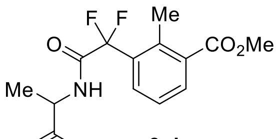

3gb

Methyl-3-(1,1-difluoro-2-((1-methoxy-1-oxopropan-2-yl)amino)-2-oxoethyl)-2-methylbenzoate (3gb)

Compound **3gb** was prepared following **Procedure D**, starting from methyl-(2-bromo-2,2-difluoroacetyl)alaninate (77.7 mg, 0.3 mmol) and 2-methylbenzoic acid (81.6 mg, 0.6 mmol). After purification, **3gb** was obtained as colorless liquid (47.4 mg, 48% yield); **^{1}H NMR** (500 MHz, $CDCl_3$) δ 7.86 (d, J = 7.8 Hz, 1H), 7.75 (d, J = 7.9 Hz, 1H), 7.32 (t, J = 7.8 Hz, 1H), 7.10 (s, 1H), 4.65 – 4.59 (m, 1H), 3.88 (s, 3H), 3.77 (s, 3H), 2.54 (s, 3H), 1.49 (d, J = 7.2 Hz, 3H); **^{13}C NMR** (125 MHz, $CDCl_3$) δ 172.3 (C_q), 168.0 (C_q), 163.2 (t, $J_{C\text{-}F}$ = 30 Hz, C_q), 137.7 (t, $J_{C\text{-}F}$ = 2.5 Hz, C_q), 132.9 (C_q), 132.4 (CH), 132.3 (q, $J_{C\text{-}F}$ = 16.3 Hz, CH), 129.7 (t, $J_{C\text{-}F}$ = 8.8 Hz, C_q), 125.6 (CH), 115.3 (t, $J_{C\text{-}F}$ = 252.5 Hz, C_q), 52.7 (CH_3), 52.2 (CH), 48.4 (CH_3), 17.9 (CH_3), 16.8 (CH_3); **^{19}F NMR** (471 MHz, $CDCl_3$) δ -99.13 (s); **ESI-HRMS** (m/z): $[M+H]^+$calcd for $C_{15}H_{18}F_2NO_5$, 330.1148, found: 330.1146.

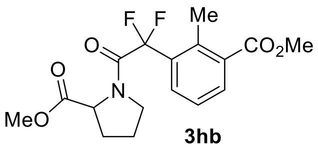

3hb

Methyl-(2,2-difluoro-2-(3-(methoxycarbonyl)-2-methylphenyl)acetyl)prolinate (3hb)

Compound **3hb** was prepared following **Procedure D**, starting from methyl-(2-bromo-2,2-difluoroacetyl)prolinate (85.5 mg, 0.3 mmol)

and 2-methylbenzoic acid (81.6 mg, 0.6 mmol). After purification, **3hb** was obtained as colorless liquid (67.3 mg, 67% yield); **^{1}H NMR** (500 MHz, $CDCl_3$) δ 7.86 (d, *J* = 7.8 Hz, 1H), 7.79 (d, *J* = 7.8 Hz, 1H), 7.33 (t, *J* = 7.7 Hz, 1H), 4.58 (dd, *J* = 8.6, 4.1 Hz, 1H), 3.90 (s, 3H), 3.74 (s, 3H), 3.71 – 3.58 (m, 2H), 2.55 (s, 3H), 2.26 – 2.17 (m, 1H), 2.10 – 1.88 (m, 3H); **^{13}C NMR** (125 MHz, $CDCl_3$) δ 171.7 (C_q), 168.1 (C_q), 162.2 (t, $J_{C\text{-}F}$ = 31.3 Hz, C_q), 138.3 (C_q), 133.0 (t, $J_{C\text{-}F}$ = 11.3 Hz, C_q), 132.14 (CH), 132.10 (CH), 129.2 (t, $J_{C\text{-}F}$ = 8.8 Hz, C_q), 125.4 (CH), 116.1 (t, $J_{C\text{-}F}$ = 250 Hz, C_q), 60.3 (CH), 52.3 (CH_3), 52.1(CH_3), 47.5 (t, $J_{C\text{-}F}$ = 3.8 Hz, CH_2), 28.2 (CH_2), 25.2 (CH_2), 16.9 (CH_3); **ESI-HRMS** (m/z): $[M+H]^+$calcd for $C_{17}H_{20}F_2NO_5$, 356.1304, found: 356.1302.

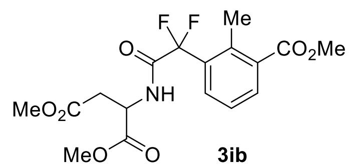

3ib

Dimethyl-(2,2-difluoro-2-(3-(methoxycarbonyl)-2-methylphenyl)acetyl)aspartate (3ib)

Compound **3ib** was prepared following **Procedure D**, starting from dimethyl-(2-bromo-2,2-difluoroacetyl)aspartate (95 mg, 0.3 mmol) and 2-methylbenzoic acid (81.6 mg, 0.6 mmol). After purification, **3ib** was obtained as colorless liquid (67.3 mg, 58% yield); **^{1}H NMR** (500 MHz, $CDCl_3$) δ 7.86 (d, *J* = 7.8 Hz, 1H), 7.76 (d, *J* = 7.9 Hz, 1H), 7.55 (d, *J* = 7.4 Hz, 1H), 7.33 (t, *J* = 7.8 Hz, 1H), 4.87 (dt, *J* = 8.5, 4.4 Hz, 1H), 3.88 (s, 3H), 3.77 (s, 3H), 3.69 (s, 3H), 3.10 (dd, *J* = 17.4, 4.2 Hz, 1H), 2.90 (dd, *J* = 17.4, 4.5 Hz, 1H), 2.53 (s, 3H); **^{13}C NMR** (125 MHz, $CDCl_3$) δ 171.2 (C_q), 170.0 (C_q), 168.0 (C_q), 163.5 (t, $J_{C\text{-}F}$ = 31.3 Hz, C_q), 137.7 (t, $J_{C\text{-}F}$ = 2.5 Hz, C_q), 132.8 (C_q), 132.43 (CH), 132.35 (t, $J_{C\text{-}F}$ = 22.5 Hz, CH), 129.7 (t, $J_{C\text{-}F}$ = 8.8 Hz, C_q), 125.6 (CH), 115.2 (t, $J_{C\text{-}F}$ = 252.5 Hz, C_q), 53.0 (CH), 52.2 (CH_3), 52.2 (CH_3), 48.7 (CH_3), 35.4 (CH_2), 16.7 (CH_3); **^{19}F NMR** (471 MHz, $CDCl_3$) δ -99.46 (s); **ESI-HRMS** (m/z): $[M+H]^+$calcd for $C_{17}H_{20}F_2NO_7$, 388.1202, found: 388.1198.

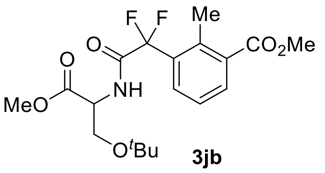

3jb

Methyl-3-(2-((3-(tert-butoxy)-1-methoxy-1-oxopropan-2-yl)amino)-1,1-difluoro-2-oxoethyl)-2-methylbenzoate (3jb)

Compound **3jb** was prepared following **Procedure D**, starting from methyl-*N*-(2-bromo-2,2-difluoroacetyl)-*O*-(tert-butyl)serinate (99 mg, 0.3 mmol) and 2-methylbenzoic acid (81.6 mg, 0.6 mmol). After purification, **3jb** was obtained as yellow liquid (75.8 mg, 63% yield); **^{1}H NMR** (500 MHz, $CDCl_3$) δ 7.86 (d, *J* = 7.8 Hz, 1H), 7.78 (d, *J* = 7.7 Hz, 1H), 7.33 (t, *J* = 7.8 Hz, 1H), 7.28 (s, 1H), 4.73 (dt, *J* = 8.4, 3.0 Hz, 1H), 3.88 (s, 3H), 3.86 (dd, *J* = 9.2, 2.8 Hz, 1H), 3.76 (s, 3H), 3.63 (dd, *J* = 9.3, 3.2 Hz, 1H), 2.57 (s, 3H), 1.13 (s, 9H); **^{13}C NMR** (125 MHz, $CDCl_3$) δ 169.8 (C_q), 168.0 (C_q), 163.4 (t, $J_{C\text{-}F}$ = 30.0 Hz, C_q), 137.8 (t, $J_{C\text{-}F}$ = 2.5 Hz, C_q), 132.9 (C_q), 132.44 (q, $J_{C\text{-}F}$ = 46.3, 22.5 Hz, CH), 132.35 (CH), 129.8 (t, $J_{C\text{-}F}$ = 8.8Hz, C_q), 125.6, (CH) 115.4 (t, $J_{C\text{-}F}$ = 251.3 Hz, C_q), 73.7 (C_q), 61.3 (CH_2), 53.0 (CH), 52.6 (CH_3), 52.1 (CH_3), 27.2 (CH_3), 16.8 (CH_3); **^{19}F NMR** (471 MHz, $CDCl_3$) δ -99.31 (s); **ESI-HRMS** (m/z): $[M-H]^+$calcd for $C_{19}H_{24}F_2NO_6$, 400.1577, found: 400.1578.

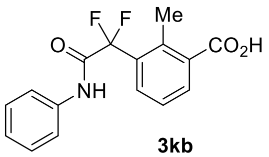

3kb

3-(1,1-Difluoro-2-oxo-2-(phenylamino)ethyl)-2-methylbenzoic acid (3kb)

Compound **3kb** was prepared following **Procedure D**, starting from ethyl-2-bromo-2,2-difluoroacetate (39 uL, 0.3 mmol) and 2-methylbenzoic acid (81.6 mg, 0.6 mmol). Methylation step did not run, and product was isolated as acid. After purification, **3kb** was obtained as yellow solid (34.8 mg, 38% yield); **MP** = 168 – 170 ºC ; **^{1}H NMR** (500 MHz, DMSO-*d*$_6$) δ 13.22 (s, 1H), 10.85 (s, 1H), 7.85 (d, *J* = 7.7 Hz, 1H), 7.78 (d, *J* = 7.6 Hz, 1H), 7.69 (d, *J* = 7.8 Hz, 2H), 7.47 (t, *J* = 7.8 Hz, 1H), 7.38 (t, *J* = 7.9 Hz, 2H), 7.19 (t, *J* = 7.4 Hz, 1H), 2.51 (s, 3H); **^{13}C NMR** (125 MHz, DMSO-*d*$_6$) δ 169.4 (C_q), 162.1 (t, $J_{C\text{-}F}$ = 31.3 Hz, C_q), 137.5 (C_q), 136.2 (C_q), 135.1 (C_q), 133.0 (t, $J_{C\text{-}F}$ = 22.5 Hz, CH), 132.1 (C_q), 129.4 (t, $J_{C\text{-}F}$ = 8.8 Hz, CH), 129.3 (CH), 126.5 (CH), 125.6 (CH), 121.6 (CH), 116.1 (t, $J_{C\text{-}F}$ = 252.5 Hz, C_q), 16.9 (CH_3); **^{19}F NMR** (471 MHz, DMSO) δ -97.28 (s); **ESI-HRMS** (m/z): $[M-H]^+$calcd for $C_{16}H_{12}F_2NO_3$, 304.0791, found:

304.0791.

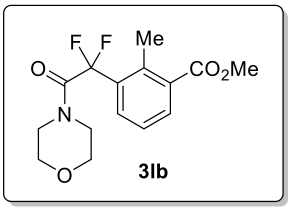

Methyl-3-(1,1-difluoro-2-morpholino-2-oxoethyl)-2-methylbenzoate (3lb)

Compound **3lb** was prepared following **Procedure D**, starting from 2-bromo-2,2-difluoro-1-morpholinoethan-1-one (72.9 mg, 0.3 mmol) and 2-methylbenzoic acid (81.6 mg, 0.6 mmol). After purification, **3lb** was obtained as colorless liquid (65.7 mg, 70% yield); **^{1}H NMR** (500 MHz, $CDCl_3$) δ 7.85 (d, *J* = 7.8 Hz, 1H), 7.65 (d, *J* = 7.9 Hz, 1H), 7.31 (t, *J* = 7.8 Hz, 1H), 3.88 (s, 3H), 3.72 – 3.66 (m, 4H), 3.57 (s, 4H), 2.50 (s, 3H); **^{13}C NMR** (125 MHz, $CDCl_3$) δ 167.9 (C_q), 161.7 (t, $J_{C\text{-}F}$ = 30 Hz, C_q), 138.0 (t, $J_{C\text{-}F}$ = 3.8 Hz, C_q), 133.6 (t, $J_{C\text{-}F}$ = 22.5 Hz, C_q), 133.1 (CH), 132.1 (CH), 128.6 (t, $J_{C\text{-}F}$ = 8.8 Hz, C_q), 125.5 (CH), 116.5 (t, $J_{C\text{-}F}$ = 252.5 Hz, C_q), 66.6 (CH_2), 66.4 (CH_2), 52.1 (CH_3), 46.6 (CH_2), 43.6 (CH_2), 17.1 (t, $J_{C\text{-}F}$ = 1.3 Hz, CH_3); **^{19}F NMR** (471 MHz, $CDCl_3$) δ -93.58 (s); **ESI-HRMS** (m/z): $[M+H]^+$calcd for $C_{15}H_{18}F_2NO_4$, 314.1198, found: 314.1197.

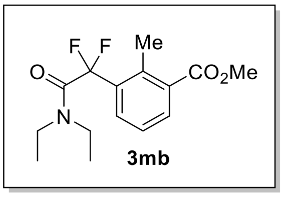

Methyl-3-(2-(diethylamino)-1,1-difluoro-2-oxoethyl)-2-methylbenzoate (3mb)

Compound **3mb** was prepared following **Procedure D**, starting from 2-bromo-*N*,*N*-diethyl-2,2-difluoroacetamide (68.7 mg, 0.3 mmol) and 2-methylbenzoic acid (81.6 mg, 0.6 mmol) and. After purification, **3mb** was obtained as colorless liquid (48.4 mg, 54% yield); **^{1}H NMR** (500 MHz, $CDCl_3$) δ 7.85 (d, *J* = 7.7 Hz, 1H), 7.69 (d, *J* = 7.7 Hz, 1H), 7.32 (t, *J* = 7.8 Hz, 1H), 3.90 (s, 3H), 3.46 – 3.42 (m, 2H), 3.39 (q, *J* = 7.9, 7.0 Hz, 2H), 2.52 (s, 3H), 1.20 (t, *J* = 7.1 Hz, 3H), 1.05 (t, *J* = 7.0 Hz, 3H); **^{13}C NMR** (125 MHz, $CDCl_3$) δ 168.1 (C_q), 162.4 (t, $J_{C\text{-}F}$ = 30 Hz, C_q), 138.0 (t, $J_{C\text{-}F}$ = 2.5 Hz, C_q), 134.3 (t, $J_{C\text{-}F}$ = 23.8 Hz, C_q), 132.9 (CH), 131.9 (CH), 128.6 (t, $J_{C\text{-}F}$ = 8.8 Hz, C_q), 125.5 (CH), 116.4 (t, $J_{C\text{-}F}$ = 251.3 Hz, C_q), 52.1 (CH_3), 42.1 (t, $J_{C\text{-}F}$ = 5.0 Hz, CH_2), 41.8 (CH_2), 17.0 (CH_3), 13.7 (CH_3), 12.2 (CH_3); **^{19}F NMR** (471 MHz, $CDCl_3$) δ -94.17 (s), -94.85 (s); **ESI-HRMS** (m/z): $[M+H]^+$calcd for $C_{15}H_{20}F_2NO_3$, 300.1406, found: 300.1404.

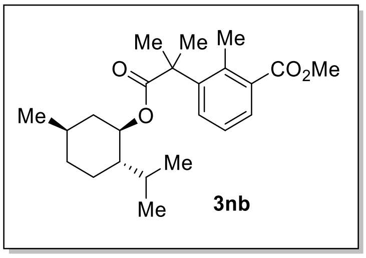

Methyl-3-(1-((5-isopropyl-2-methylcyclohexyl)oxy)-2-methyl-1-oxopropan-2-yl)-2-methylbenzoate (3nb)

Compound **3nb** was prepared following **Procedure A**, starting from phenyl-5-isopropyl-2-methylcyclohexyl 2-bromo-2-methylpropanoate (91.2 mg, 0.3 mmol) and *o*-methylbenzoic acid (81.6 mg, 0.6 mmol). After purification, **3nb** was obtained as colorless liquid (81.9 mg, 73% yield); **^{1}H NMR** (400 MHz, $CDCl_3$) δ 7.61 (d, *J* = 8.4 Hz, 1H), 7.48 (d, *J* = 7.2 Hz, 1H), 7.21 (t, *J* = 7.8 Hz, 1H), 4.65 – 4.56 (m, 1H), 3.86 (s, 3H), 2.37 (s, 3H), 2.14 – 1.95 (m, 1H), 1.80 – 1.60 (m, 3H), 1.57 (d, *J* = 4.3 Hz, 6H), 1.52 – 1.40 (m, 1H), 1.29 – 1.22 (m, 1H), 1.07 – 0.93 (m, 1H), 0.87 (d, *J* = 6.6 Hz, 4H), 0.82 (dd, *J* = 12.0, 4.1 Hz, 1H), 0.77 (d, *J* = 7.1 Hz, 3H), 0.70 (d, *J* = 7.0 Hz, 3H); **^{13}C NMR** (100 MHz, $CDCl_3$) δ 177.2 (C_q), 169.2 (C_q), 144.1 (C_q), 136.8 (C_q), 132.5 (CH), 128.4 (CH), 128.1 (C_q), 125.4 (CH), 74.9 (CH), 51.9 (CH_3), 46.9 (C_q), 46.7 (CH), 40.0 (CH), 34.1 (CH_2), 31.2 (CH), 27.3 (CH_2), 27.0 (CH_3), 25.6 (CH_2), 22.9 (CH_3), 22.0 (CH_3), 20.7 (CH_3), 17.8 (CH_3), 15.7 (CH_3); **ESI-HRMS (m/z)**: $[M+H]^+$calcd for $C_{23}H_{35}O_4$, 375.2530, found: 375.2524.

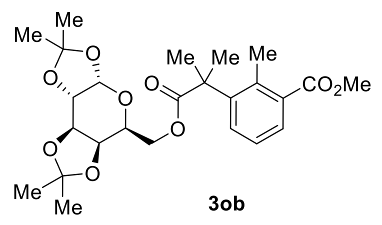

3ob

Methyl-2-methyl-3-(2-methyl-1-oxo-1-(((3a*S*,5*S*,5a*R*,8a*R*,8b*S*)-2,2,7,7-tetramethyltetrahydro-5H-bis([1,3]dioxolo)[4,5-b:4',5'-d]pyran-5-yl)methoxy)propan-2-yl)benzoate (3ob)

Compound **3ob** was prepared following **Procedure A**, starting from 2,2,7,7-tetramethyltetrahydro-5H-bis([1,3]dioxolo)[4,5-b:4',5'-d]pyran-5-yl 2-bromo-2-methylpropanoate (118.2 mg, 0.3 mmol) and 2-methylbenzoic acid (81.6 mg, 0.6 mmol). After purification, **3ob** was obtained as colorless liquid (82.1 mg, 59% yield); **^{1}H NMR** (400 MHz, $CDCl_3$) δ 7.61 (d, *J* = 7.7 Hz, 1H), 7.49 (d, *J* = 7.3 Hz, 1H), 7.20 (t, *J* = 7.8 Hz, 1H), 5.46 (d, *J* = 4.9 Hz, 1H), 4.53 (dd, *J* = 7.9, 2.5 Hz, 1H), 4.30 – 4.24 (m, 2H), 4.19 (dd, *J* = 11.5, 7.6 Hz, 1H), 4.05 (dd, *J* = 7.9, 1.9 Hz, 1H), 3.91 (ddd, *J* = 7.0, 4.7, 1.8 Hz, 1H), 3.84 (s, 3H), 2.34 (s, 3H), 1.59 (d, *J* = 2.5 Hz, 6H), 1.39 (d, *J* = 4.5 Hz, 6H), 1.27 (d, *J* = 4.8 Hz, 6H); **^{13}C NMR** (100 MHz, $CDCl_3$) δ 177.7 (C_q), 169.1 (C_q), 144.0 (C_q), 136.9 (C_q), 132.5 (CH), 128.4 (CH), 128.2 (C_q), 125.4 (CH), 109.4 (C_q), 108.6 (C_q), 96.1 (CH), 70.8 (CH), 70.5 (CH), 70.3 (CH), 65.8 (CH), 63.7 (CH_3), 51.9 (C_q), 46.7 (CH_3), 27.3 (CH_3), 27.2 (CH_3), 25.8 (CH_3), 24.9 (CH_3), 24.2 (CH_3), 17.4 (CH_3); **ESI-HRMS (m/z)**: $[M+H]^+$calcd for $C_{25}H_{35}O_9$, 479.2276, found: 479.2269.

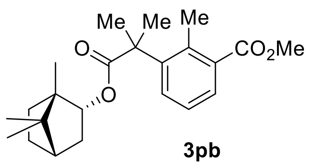

3pb

Methyl-2-methyl-3-(2-methyl-1-oxo-1-(((1S,2R,4S)-1,7,7-trimethylbicyclo[2.2.1]heptan-2-yl)oxy)propan-2-yl)benzoate (3pb)

Compound **3pb** was prepared following **Procedure A**, starting from (1S,2R,4S)-1,7,7-trimethylbicyclo[2.2.1]heptan-2-yl 2-bromo-2-methylpropanoate (90.6 mg, 0.3 mmol) and 2-methylbenzoic acid (81.6 mg, 0.6 mmol). After purification, **3pb** was obtained as colorless liquid (78.1 mg, 70% yield); **^{1}H NMR** (400 MHz, $CDCl_3$) δ 7.62 (d, *J* = 7.1 Hz, 1H), 7.49 (d, *J* = 7.3 Hz, 1H), 7.21 (t, *J* = 7.8 Hz, 1H), 4.82 – 4.77 (m, 1H), 3.85 (s, 3H), 2.36 (s, 3H), 2.35 – 2.26 (m, 1H), 1.69 – 1.60 (m, 2H), 1.58 (d, *J* = 3.5 Hz, 6H), 1.49 – 1.41 (m, 1H), 1.14 – 0.97 (m, 2H), 0.91 (d, *J* = 3.4 Hz, 1H), 0.86 (s, 3H), 0.79 (s, 3H), 0.71 (s, 3H); **^{13}C NMR** (100 MHz, $CDCl_3$) δ 177.9 (C_q), 169.1 (C_q), 144.3 (C_q), 136.6 (C_q), 132.5 (CH), 128.3 (CH), 128.1 (C_q), 125.4 (CH), 80.5 (CH), 51.9 (C_q), 48.6 (C_q), 47.6 (C_q), 46.8 (C_q), 44.7 (C_q), 36.3 (CH_2), 27.8 (CH_2), 27.1 (CH_2), 26.7 (CH_3), 19.5 (CH_3), 18.8 (CH_3), 17.7 (CH_3), 13.3 (CH_3); **ESI-HRMS (m/z)**: $[M+H]^+$calcd for $C_{23}H_{33}O_4$, 373.2373, found: 373.2365.

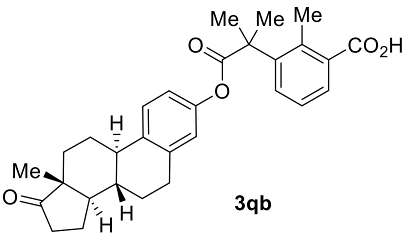

3qb

2-Methyl-3-(2-methyl-1-((14-methyl-15-oxo-7,8,9,11,12,13,14,15,16,17-decahydro-6H-cyclopenta[a]phenanthren-3-yl)oxy)-1-oxopropan-2-yl)benzoic acid (3qb)

Compound **3qb** was prepared following **Procedure A**, starting from (8R,9S,13S,14S)-14-methyl-15-oxo-7,8,9,11,12,13,14,15,16,17-decahydro-6H-cyclopenta[a]phenanthren-3-yl 2-bromo-2-methylpropanoate (125.4 mg, 0.3 mmol) and 2-methylbenzoic acid (81.6 mg, 0.6 mmol). Methylation step did not run, and product was isolated as acid. After purification, **3qb** was obtained as white solid (102.4 mg, 72% yield); **^{1}H NMR** (400 MHz, DMSO-*d*$_6$) δ 12.97 (s, 1H), 7.62 (d, *J* = 7.8 Hz, 2H), 7.43 – 7.23 (m, 2H), 6.88 – 6.67 (m, 2H), 2.82 (dd, *J* = 9.3, 4.3 Hz, 2H), 2.54 – 2.49 (m, 1H), 2.46 (s, 3H), 2.35 – 2.29 (m, 1H), 2.23 – 2.15 (m, 1H), 2.10 – 2.00 (m, 1H), 1.97 – 1.87 (m, 2H), 1.76 (d, *J* = 10.1 Hz, 1H), 1.66 (s, 6H), 1.59 – 1.43 (m, 3H), 1.42 – 1.27 (m, 3H), 0.81 (s, 3H); **^{13}C NMR** (100 MHz, DMSO-*d*$_6$) δ 176.7 (C_q), 170.4 (C_q), 148.8 (C_q), 143.8 (C_q), 138.4 (C_q), 137.8 (C_q), 135.6 (C_q), 134.6 (C_q), 128.6 (CH), 128.3 (CH), 126.9 (CH),

126.3 (CH), 121.5 (CH), 118.8 (CH), 50.0 (C_q), 47.7 (C_q), 46.9 (CH), 44.0 (CH), 37.9 (CH), 35.8 (CH_2), 31.8 (CH_2), 29.3 (CH_2), 27.4 (CH_2), 26.2 (CH_3), 25.8 (CH_2), 21.6 (CH_2), 17.8 (CH_3), 13.9 (CH_3); **ESI-HRMS (m/z)**: $[M-H]^+$calcd for $C_{30}H_{33}O_5$, 473.2333, found: 473.2336.

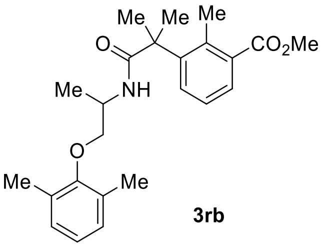

3rb

Methyl-3-(1-((1-(2,6-dimethylphenoxy)propan-2-yl)amino)-2-methyl-1-oxopropan-2-yl)-2-methylbenzoate (3rb)

Compound **3rb** was prepared following **Procedure A**, starting from 2-bromo-*N*-(1-(2,6-dimethylphenoxy)propan-2-yl)-2-methylpropanamide (98 mg, 0.3 mmol) and 2-methylbenzoic acid (82 mg, 0.6 mmol). After purification, **3rb** was obtained as grey liquid (56 mg, 47% yield); **^{1}H NMR** (500 MHz, $CDCl_3$) δ 7.69 (d, *J* = 7.2 Hz, 1H), 7.60 (d, *J* = 7.8 Hz, 1H), 7.27 (t, *J* = 7.8 Hz, 1H), 6.93 (d, *J* = 7.4 Hz, 2H), 6.87 (dd, *J* = 8.3, 6.5 Hz, 1H), 5.70 (d, *J* = 8.0 Hz, 1H), 4.36 – 4.24 (m, 1H), 3.87 (s, 3H), 3.63 (ddd, *J* = 20.4, 9.1, 3.6 Hz, 2H), 2.45 (s, 3H), 2.02 (s, 6H), 1.61 (d, *J* = 3.7 Hz, 6H), 1.31 (d, *J* = 6.8 Hz, 3H); **^{13}C NMR** (125 MHz, $CDCl_3$) δ 177.1 (C_q), 168.8 (C_q), 154.5 (C_q), 143.8 (C_q), 137.9 (C_q), 133.3 (CH), 130.5 (C_q), 129.2 (CH), 128.9 (CH), 128.8 (C_q), 125.8 (CH), 124.0 (CH), 73.7 (CH_2), 52.0 (CH_3), 47.2 (CH), 45.6 (C_q), 27.6 (CH_3), 27.4 (CH_3), 18.1 (CH_3), 17.4 (CH_3), 15.9 (CH_3); **ESI-HRMS** (m/z): $[M-H]^+$calcd for $C_{24}H_{30}NO_4$, 396.1816, found: 396.1817.

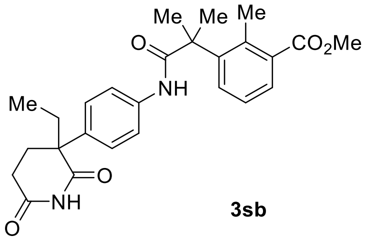

3sb

Methyl-3-(1-((4-(3-ethyl-2,6-dioxopiperidin-3-yl)phenyl)amino)-2-methyl-1-oxopropan-2-yl)-2 methylbenzoate (3sb)

Compound **3sb** was prepared following **Procedure A**, starting from 2-bromo-*N*-(4-(3-ethyl-2,6-dioxopiperidin-3-yl)phenyl)-2-methylpropanamide (114 mg, 0.3 mmol) and 2-methylbenzoic acid (82 mg, 0.6 mmol). After purification, **3sb** was obtained as white solid (86.4 mg, 64% yield); **MP** = 191 – 193 ºC ; **^{1}H NMR** (500 MHz, $CDCl_3$) δ 8.34 (s, 1H), 7.70 (d, *J* = 7.6 Hz, 1H), 7.66 (d, *J* = 7.8 Hz, 1H), 7.35 (t, *J* = 6.1 Hz, 2H), 7.32 (d, *J* = 7.8 Hz, 1H), 7.16 (d, *J* = 8.6 Hz, 2H), 6.99 (s, 1H), 3.88 (s, 3H), 2.58 – 2.52 (m, 1H), 2.44 (s, 3H), 2.38 – 2.31 (m, 2H), 2.23 – 2.14 (m, 1H), 2.01 - 1.95 (m, 1H), 1.88 – 1.82 (m, 1H), 1.67 (s, 6H), 0.83 (t, *J* = 7.4 Hz, 3H); **^{13}C NMR** (125 MHz, $CDCl_3$) δ 176.3 (C_q), 175.1 (C_q), 172.4 (C_q), 168.9 (C_q), 143.2 (C_q), 137.7 (C_q), 137.2 (C_q), 134.3 (C_q), 133.6 (CH), 129.3 (CH), 129.0 (C_q), 126.7 (CH), 126.1 (CH), 120.3 (CH), 52.2 (C_q), 50.5 (CH_3), 48.1 (C_q), 32.7 (CH_2), 29.1 (CH_2), 27.4 (CH_3), 26.8 (CH_2), 17.6 (CH_3), 8.9 (CH_3); **ESI-HRMS** (m/z): $[M-H]^+$calcd for $C_{26}H_{29}N_2O_5$, 449.2082, found: 449.2080.

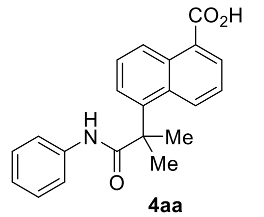

4aa

5-(2-Methyl-1-oxo-1-(phenylamino)propan-2-yl)-1-naphthoic acid (4aa)

Compound **4aa** was prepared following **Procedure E**, starting from 2-bromo-2-methyl-*N*-phenylpropanamide (72.3 mg, 0.3 mmol) and 1-naphthoic acid (103 mg, 0.6 mmol). Methylation step did not run, and product was isolated as acid. After purification, **4aa** was obtained as white solid (71.9 mg, 72% yield); **MP** = 228 – 230ºC; **^{1}H NMR** (500 MHz, DMSO-d_6) δ 8.94 (s, 1H), 8.77 (d, *J* = 8.6 Hz, 1H), 8.28 (d, *J* = 8.7 Hz, 1H), 8.02 (dd, *J* = 7.2, 1.1 Hz, 1H), 7.80 (d, *J* = 7.3 Hz, 1H), 7.73 – 7.67 (m, 1H), 7.56 (dd, *J* = 8.8, 7.2 Hz, 1H), 7.45 (d, *J* = 7.5 Hz, 2H), 7.26 – 7.15 (m, 2H), 6.99 (t, *J* = 7.4 Hz, 1H), 1.75 (s, 6H); **^{13}C NMR** (125 MHz, DMSO-d_6) δ 176.9 (C_q), 169.6 (C_q), 141.4

(C_q), 139.4 (C_q), 132.0 (C_q), 131.8 (C_q), 130.3 (CH), 128.8 (CH), 128.8 (CH), 128.7 (CH), 127.4 (CH), 125.6 (C_q), 125.5 (CH), 125.2 (CH), 124.0 (CH), 121.2 (CH), 47.8 (C_q), 28.2 (CH_3); **ESI-HRMS** (m/z): $[M+H]^+$calcd for $C_{21}H_{20}NO_3$, 334.1438, found: 334.1441.

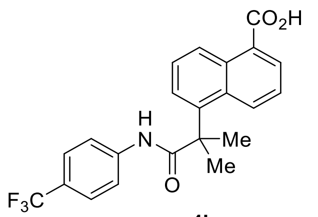

4ba

5-(2-Methyl-1-oxo-1-((4-(trifluoromethyl)phenyl)amino)propan-2-yl)-1-naphthoic acid (4ba)

Compound **4ba** was prepared following **Procedure E**, starting from 2-bromo-2-methyl-*N*-(4-(trifluoromethyl)phenyl)propanamide (92.7 mg, 0.3 mmol) and 1-naphthoic acid (103 mg, 0.6 mmol). Methylation step did not run, and product was isolated as acid. After purification, **4ba** was obtained as brown liquid (96.2 mg, 80% yield); **^{1}H NMR** (500 MHz, DMSO-d_6) δ 9.31 (s, 1H), 8.79 (d, J = 8.6 Hz, 1H), 8.23 (d, J = 8.7 Hz, 1H), 8.02 (d, J = 7.1 Hz, 1H), 7.84 (d, J = 7.4 Hz, 1H), 7.74 (dd, J = 12.4, 7.3 Hz, 3H), 7.64 – 7.54 (m, 3H), 1.79 (s, 6H); **^{13}C NMR** (125 MHz, DMSO-d_6) δ 177.6 (C_q), 169.6 (C_q), 143.1 (C_q), 140.9 (C_q), 132.0 (C_q), 131.7 (C_q), 130.5 (C_q), 130.1 (CH), 128.6 (CH), 128.4 (CH), 127.3 (C_q), 126.1 (q, $J_{C\text{-}F}$ = 4 Hz, CH), 125.9 (CH), 125.8 (d, $J_{C\text{-}F}$ = 18.8 Hz, CH), 125.3 (CH), 123.8 (q, $J_{C\text{-}F}$ = 32 Hz, C_q), 120.8 (CH), 48.1 (C_q), 28.0 (CH_3); **^{19}F NMR** (471 MHz, DMSO-d_6) δ -60.45 (s); **ESI-HRMS** (m/z): $[M-H]^+$calcd for $C_{22}H_{17}F_3NO_3$, 400.1166, found: 400.1165.

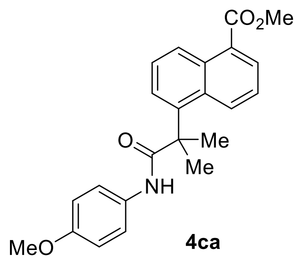

4ca

Methyl-5-(1-((4-methoxyphenyl)amino)-2-methyl-1-oxopropan-2-yl)-1-naphthoate (4ca)

Compound **4ca** was prepared following **Procedure E**, starting from 2-bromo-*N*-(4-methoxyphenyl)-2-methylpropanamide (81.3 mg, 0.3 mmol) and 1-naphthoic acid (103.2 mg, 0.6 mmol). After purification, **4ca** was obtained as colorless liquid (72.4 mg, 64% yield); **^{1}H NMR** (400 MHz, $CDCl_3$) δ 8.86 (d, J = 8.7 Hz, 1H), 8.25 (d, J = 8.7 Hz, 1H), 8.09 (d, J = 7.1 Hz, 1H), 7.75 (d, J = 7.4 Hz, 1H), 7.62 (ddd, J = 8.7, 7.3, 1.3 Hz, 1H), 7.53 – 7.44 (m, 1H), 7.06 (d, J = 8.8 Hz, 2H), 6.77 (s, 1H), 6.74 – 6.66 (m, 2H), 3.99 (d, J = 1.4 Hz, 3H), 3.70 (d, J = 1.5 Hz, 3H), 1.83 (s, 6H); **^{13}C NMR** (125 MHz, $CDCl_3$) δ 176.6 (C_q), 168.1 (C_q), 156.4 (C_q), 140.4 (C_q), 132.4 (C_q), 131.4 (C_q), 130.6 (C_q), 129.6 (CH), 129.3 (CH), 128.7 (CH), 126.9 (C_q), 126.2 (CH), 124.9 (CH), 124.5 (CH), 122.2 (CH), 113.8 (CH), 55.3 (CH_3), 52.2 (CH_3), 47.8 (C_q), 27.9 (CH_3); **ESI-HRMS** (m/z): $[M-H]^+$calcd for $C_{23}H_{22}NO_4$, 376.1554, found: 376.1552.

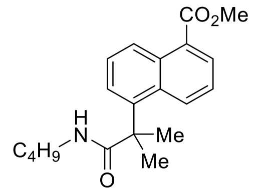

4da

Methyl-5-(1-(butylamino)-2-methyl-1-oxopropan-2-yl)-1-naphthoate (4da)

Compound **4da** was prepared following **Procedure E**, starting from 2-bromo-*N*-butyl-2-methylpropanamide (66.3 mg, 0.3 mmol) and 1-naphthoic acid (103 mg, 0.6 mmol). After purification, **4da** was obtained as white solid (49.1 mg, 50% yield); **MP** = 75 – 77 ºC；**^{1}H NMR** (400 MHz, $CDCl_3$) δ 8.77 (d, J = 8.0 Hz, 1H), 8.13 (d, J = 8.7 Hz, 1H), 8.06 (d, J = 7.2 Hz, 1H), 7.63 (d, J = 7.3 Hz, 1H), 7.58 – 7.50 (m, 1H), 7.50 – 7.41 (m, 1H), 5.13 (s, 1H), 3.96 (d, J = 1.7 Hz, 3H), 3.05 (q, J = 6.1 Hz, 2H), 1.70 (s, 6H), 1.22 – 1.08 (m, 2H), 0.95 (dd, J = 14.6, 7.2 Hz, 2H), 0.69 (dd, J = 7.8, 6.9 Hz, 3H); **^{13}C NMR** (100 MHz, $CDCl_3$) δ 178.1 (C_q), 168.1 (C_q), 141.0 (C_q), 132.2 (C_q), 131.4 (C_q), 130.0 (CH), 129.2 (CH), 128.4 (CH), 126.7 (C_q), 125.7 (CH), 124.4 (CH), 124.2 (CH), 52.1 (CH_3), 47.0 (C_q), 39.3 (CH_2), 31.1 (CH_2), 28.0 (CH_3), 19.6 (CH_2), 13.4 (CH_3); **ESI-HRMS** (m/z): $[M+H]^+$calcd for $C_{20}H_{26}NO_3$, 328.1907, found: 328.1897.

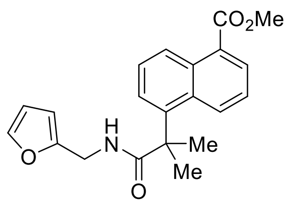

4ea

Methyl-5-(1-((furan-2-ylmethyl)amino)-2-methyl-1-oxopropan-2-yl)-1-naphthoate (4ea)

Compound **4ea** was prepared following **Procedure E**, starting from 2-bromo-*N*-(furan-2-ylmethyl)-2-methylpropanamide (73.5 mg, 0.3 mmol) and 1-naphthoic acid (103 mg, 0.6 mmol). After purification, **4ea** was obtained as white solid (43.2 mg, 41% yield); **MP** = 116 – 118 ºC ; **^{1}H NMR** (500 MHz, $CDCl_3$) δ 8.71 (d, *J* = 8.6 Hz, 1H), 7.96 (dd, *J* = 15.6, 7.9 Hz, 2H), 7.57 (d, *J* = 7.3 Hz, 1H), 7.48 (t, *J* = 8.0 Hz, 1H), 7.29 (t, *J* = 8.0 Hz, 1H), 7.02 (s, 1H), 6.08 (s, 1H), 5.86 (d, *J* = 2.9 Hz, 1H), 5.30 (d, *J* = 5.3 Hz, 1H), 4.18 (d, *J* = 5.8 Hz, 2H), 3.91 (s, 3H), 1.65 (s, 6H); **^{13}C NMR** (125 MHz, $CDCl_3$) δ 178.2 (C_q), 168.3 (C_q), 151.1 (C_q), 141.8 (C_q), 140.7 (CH), 132.4 (C_q), 131.4 (C_q), 129.8 (CH), 129.2 (CH), 128.6 (CH), 126.9 (C_q), 126.1 (CH), 124.6 (CH), 124.4 (CH), 110.1 (CH), 107.1 (CH), 52.3 (CH_3), 47.3 (C_q), 36.7 (CH_2), 28.1 (CH_3); **ESI-HRMS** (m/z): $[M+H]^+$calcd for $C_{21}H_{22}NO_4$, 352.1543, found: 352.1538.

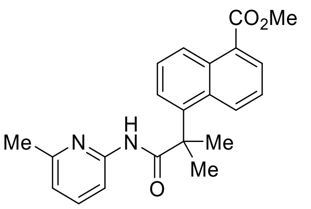

4fa

Methyl-5-(2-methyl-1-((6-methylpyridin-2-yl)amino)-1-oxopropan-2-yl)-1-naphthoate (4fa)

Compound **4fa** was prepared following **Procedure E**, starting from2-bromo-2-methyl-*N*-(6-methylpyridin-2-yl)propanamide (76.8 mg, 0.3 mmol) and 1-naphthoic acid (103 mg, 0.6 mmol). After purification, **4fa** was obtained as colorless liquid (67 mg, 62% yield); **^{1}H NMR** (500 MHz, $CDCl_3$) δ 8.86 (d, *J* = 8.7 Hz, 1H), 8.22 (d, *J* = 8.7 Hz, 1H), 8.10 (d, *J* = 8.3 Hz, 1H), 8.05 (d, *J* = 7.1 Hz, 1H), 7.74 (d, *J* = 7.1 Hz, 1H), 7.62 (dd, *J* = 8.5, 7.5 Hz, 1H), 7.52 (t, *J* = 7.9 Hz, 1H), 7.45 (dd, *J* = 8.6, 7.4 Hz, 1H), 7.41 (s, 1H), 6.75 (d, *J* = 7.5 Hz, 1H), 3.97 (s, 3H), 2.22 (s, 3H), 1.83 (s, 6H); **^{13}C NMR** (125 MHz, $CDCl_3$) δ 176.9 (C_q), 168.0 (C_q), 156.5 (C_q), 150.5 (C_q), 139.6 (C_q), 138.3 (C_q), 132.4 (C_q), 131.4 (CH), 129.2 (CH), 129.1 (CH), 128.7 (C_q), 126.9 (CH), 126.3 (CH), 124.9 (CH), 124.6 (CH), 119.0 (CH), 110.1 (CH), 52.2 (CH_3), 48.2 (C_q), 27.7 (CH_3), 23.6 (CH_3); **ESI-HRMS** (m/z): $[M+H]^+$calcd for $C_{22}H_{23}N_2O_3$, 363.1703, found: 363.1696.

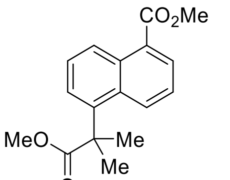

4ga

Methyl-5-(1-methoxy-2-methyl-1-oxopropan-2-yl)-1-naphthoate (4ga)

Compound **4ga** was prepared following **Procedure E**, starting from methyl-2-bromo-2-methylpropanoate (54 mg, 0.3 mmol) and 1-naphthoic acid (103 mg, 0.6 mmol). After purification, **4ga** was obtained as white solid (66 mg, 77% yield); **MP** = 74 – 76 ºC ; **^{1}H NMR** (500 MHz, $CDCl_3$) δ 8.80 (ddd, *J* = 8.0, 1.5, 0.8 Hz, 1H), 8.09 (dd, *J* = 7.2, 1.0 Hz, 1H), 8.06 (dd, *J* = 8.7, 0.8 Hz, 1H), 7.63 – 7.58 (m, 2H), 7.50 (dd, *J* = 8.7, 7.2 Hz, 1H), 4.01 (s, 3H), 3.58 (s, 3H), 1.79 (s, 6H); **^{13}C NMR** (125 MHz, $CDCl_3$) δ 179.2 (C_q), 168.3 (C_q), 140.9 (C_q), 132.1 (C_q), 131.5 (C_q), 128.8 (CH), 128.8 (CH), 128.5 (CH), 126.9 (C_q), 125.3 (CH), 124.5 (CH), 123.2 (CH), 52.4 (CH_3), 52.2 (CH_3), 46.4 (C_q), 27.6 (CH_3); **ESI-HRMS** (m/z): $[M+H]^+$calcd for $C_{17}H_{19}O_4$, 287.1278, found: 287.1273.

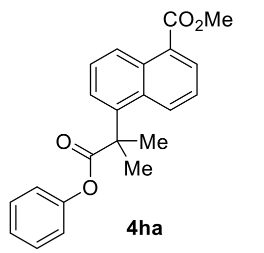

Methyl-5-(2-methyl-1-oxo-1-phenoxypropan-2-yl)-1-naphthoate (4ha)

Compound **4ha** was prepared following **Procedure E**, starting from phenyl-2-bromo-2-methylpropanoate (72.6 mg, 0.3 mmol) and 1-naphthoic acid (103.2 mg, 0.6 mmol). After purification, **4ha** was obtained as colorless liquid (90.8 mg, 87% yield); **^{1}H NMR** (400 MHz, $CDCl_3$) δ 8.89 (d, *J* = 8.6 Hz, 1H), 8.35 (d, *J* = 8.7 Hz, 1H), 8.19 (dd, *J* = 7.2, 0.8 Hz, 1H), 7.74 (dd, *J* = 7.4, 1.0 Hz, 1H), 7.70 – 7.56 (m, 2H), 7.30 (t, *J* = 7.9 Hz, 2H), 7.17 (t, *J* = 7.4 Hz, 1H), 6.89 – 6.76 (m, 2H), 4.04 (s, 3H), 1.96 (s, 6H); **^{13}C NMR** (125 MHz, $CDCl_3$) δ 177.4 (C_q), 168.2 (C_q), 150.7 (C_q), 140.4 (C_q), 132.2 (C_q), 131.4 (C_q), 129.2 (CH), 129.0 (CH), 128.9 (CH), 128.6 (CH), 127.0 (CH), 125.7 (C_q), 125.5 (CH), 124.6 (CH), 123.4 (CH), 121.2 (CH), 52.2 (CH_3), 46.5 (C_q), 27.5 (CH_3); **ESI-HRMS** (m/z): $[M+H]^+$calcd for $C_{22}H_{21}O_4$, 349.1434, found: 349.1429.

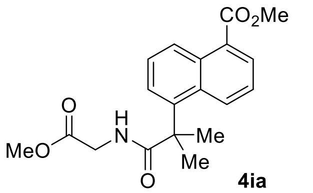

Methyl-5-(1-((2-methoxy-2-oxoethyl)amino)-2-methyl-1-oxopropan-2-yl)-1-naphthoate (4ia)

Compound **4ia** was prepared following **Procedure E**, starting from methyl-(2-bromo-2-methylpropanoyl)glycinate (71 mg, 0.3 mmol) and 1-naphthoic acid (103 mg, 0.6 mmol), and 1,4-dioxane instead of ^{t}BuOH/HFIP. After purification, **4ia** was obtained as colorless liquid (45.3 mg, 44% yield); **^{1}H NMR** (500 MHz, $CDCl_3$) δ 8.79 (d, *J* = 8.7 Hz, 1H), 8.12 (d, *J* = 8.7 Hz, 1H), 8.08 (d, *J* = 7.2 Hz, 1H), 7.68 (d, *J* = 7.3 Hz, 1H), 7.62 – 7.54 (m, 1H), 7.53 – 7.44 (m, 1H), 5.64 (t, *J* = 5.7 Hz, 1H), 3.98 (s, 3H), 3.84 (d, *J* = 5.6 Hz, 2H), 3.57 (s, 3H), 1.76 (s, 6H); **^{13}C NMR** (125 MHz, $CDCl_3$) δ 178.5 (C_q), 169.8 (C_q), 168.1 (C_q), 140.4 (C_q), 132.3 (C_q), 131.4 (C_q), 130.0 (CH), 129.2 (CH), 128.5 (CH), 126.8(C_q), 126.0 (CH), 124.5 (CH), 124.4 (CH), 52.2 (CH_3), 51.9 (CH_3), 47.0 (C_q), 41.3 (CH_2), 27.9 (CH_3); **ESI-HRMS** (m/z): $[M+H]^+$calcd for $C_{19}H_{22}NO_5$, 344.1492, found: 344.1490.

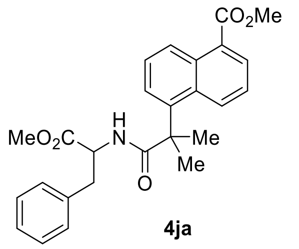

Methyl-5-(1-((1-methoxy-1-oxo-3-phenylpropan-2-yl)amino)-2-methyl-1-oxopropan-2-yl)-1-naphthoate (4ja)

Compound **4ja** was prepared following **Procedure E**, starting from methyl-(2-bromo-2-methylpropanoyl)phenylalaninate (98 mg, 0.3 mmol) and 1-naphthoic acid (103 mg, 0.6 mmol). and 1,4-dioxane instead of ^{t}BuOH/HFIP. After purification, **4ja** was obtained as yellow liquid (77.9 mg, 60% yield); **^{1}H NMR** (500 MHz, $CDCl_3$) δ 8.84 (d, *J* = 8.4 Hz, 1H), 8.10 (d, *J* = 7.7 Hz, 2H), 7.64 – 7.53 (m, 2H), 7.49 – 7.42 (m, 1H), 7.03 (t, *J* = 7.4 Hz, 1H), 6.92 (t, *J* = 7.6 Hz, 2H), 6.35 (d, *J* = 7.4 Hz, 2H), 5.38 (d, *J* = 8.2 Hz, 1H), 4.90 – 4.76 (m, 1H), 4.05 (s, 3H), 3.59 (s, 3H), 2.83 (dd, *J* = 13.8, 6.6 Hz, 1H), 2.73 (dd, *J* = 13.8, 5.4 Hz, 1H), 1.74 (s, 3H), 1.67 (s, 3H); **^{13}C NMR** (125 MHz, $CDCl_3$) δ 177.8 (C_q), 171.6 (C_q), 168.3 (C_q), 140.3 (C_q), 135.0 (C_q), 132.4 (C_q), 131.5 (C_q), 129.9 (CH), 129.3 (CH), 128.7 (CH), 128.4 (CH), 128.3 (C_q), 126.9 (CH), 126.8 (CH), 126.0 (CH), 124.6 (CH), 124.5 (CH), 52.6 (CH), 52.3 (CH_3), 52.1 (CH_3), 47.1 (C_q), 37.5 (CH_2), 28.2 (CH_3), 27.3 (CH_3); **ESI-HRMS** (m/z): $[M+H]^+$calcd for $C_{26}H_{28}NO_5$, 434.1962, found: 434.1954.

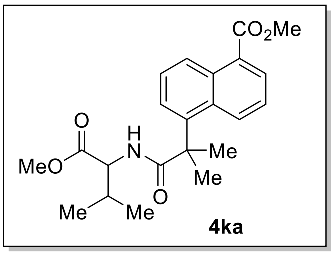

4ka

Methyl-5-(1-((1-methoxy-3-methyl-1-oxobutan-2-yl)amino)-2-methyl-1-oxopropan-2-yl)-1-naphthoate (4ka)

Compound **4ka** was prepared following **Procedure E**, starting from methyl-(2-bromo-2-methylpropanoyl)valinate (83.7 mg, 0.3 mmol) and 1-naphthoic acid (103 mg, 0.6 mmol), and 1,4-dioxane instead of ^{t}BuOH/HFIP. After purification, **4ka** was obtained as colorless liquid (54.3 mg, 47% yield); **^{1}H NMR** (500 MHz, $CDCl_3$) δ 8.86 (d, *J* = 8.7 Hz, 1H), 8.15 (d, *J* = 8.7 Hz, 1H), 8.10 (d, *J* = 7.2 Hz, 1H), 7.71 (d, *J* = 7.3 Hz, 1H), 7.61 (t, *J* = 8.0 Hz, 1H), 7.47 (t, *J* = 8.0 Hz, 1H), 5.49 (d, *J* = 8.7 Hz, 1H), 4.45 (dd, *J* = 8.7, 5.2 Hz, 1H), 4.00 (s, 3H), 3.51 (s, 3H), 1.95 – 1.87 (m, 1H), 1.77 (d, *J* = 3.2 Hz, 6H), 0.61 (d, *J* = 6.9 Hz, 3H), 0.47 (d, *J* = 6.9 Hz, 3H); **^{13}C NMR** (125 MHz, $CDCl_3$) δ 178.2 (C_q), 171.7 (C_q), 168.1 (C_q), 140.4 (C_q), 132.3 (C_q), 131.3 (C_q), 130.1 (CH), 129.2 (CH), 128.4 (CH), 126.9 (C_q), 126.0 (CH), 124.4 (CH), 124.3 (CH), 57.2 (CH), 52.1 (CH_3), 51.7 (CH_3), 47.3 (C_q), 30.7 (CH), 28.0 (CH_3), 27.7 (CH_3), 18.7 (CH_3), 17.4 (CH_3); **ESI-HRMS** (m/z): $[M+H]^+$calcd for $C_{22}H_{28}NO_5$, 386.1962, found: 386.1959.

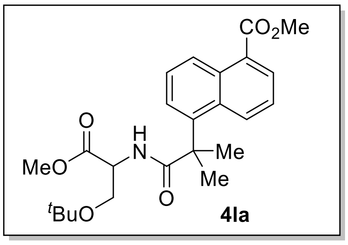

4la

Methyl-5-(1-((3-(tert-butoxy)-1-methoxy-1-oxopropan-2-yl)amino)-2-methyl-1-oxopropan-2-yl)-1-naphthoate (4la)

Compound **4la** was prepared following **Procedure E**, starting from methyl-*N*-(2-bromo-2-methylpropanoyl)-O-(tert-butyl)serinate (96.9 mg, 0.3 mmol) and 1-naphthoic acid (103 mg, 0.6 mmol). and 1,4-dioxane instead of ^{t}BuOH/HFIP. After purification, **4la** was obtained as colorless liquid (45 mg, 35% yield); **^{1}H NMR** (500 MHz, $CDCl_3$) δ 8.81 (d, *J* = 8.7 Hz, 1H), 8.16 (d, *J* = 8.7 Hz, 1H), 8.08 (dt, *J* = 6.6, 2.9 Hz, 1H), 7.77 – 7.68 (m, 1H), 7.60 (ddd, *J* = 8.6, 6.8, 4.1 Hz, 1H), 7.48 (ddt, *J* = 11.2, 6.4, 2.8 Hz, 1H), 5.90 (d, *J* = 8.4 Hz, 1H), 4.65 (dt, *J* = 8.9, 3.1 Hz, 1H), 3.99 (dd, *J* = 5.3, 2.7 Hz, 3H), 3.60 (dd, *J* = 4.7, 2.5 Hz, 3H), 3.56 (dt, *J* = 8.8, 3.3 Hz, 1H), 3.18 – 2.97 (m, 1H), 1.83 (s, 3H), 1.74 (s, 3H), 0.64 (s, 9H); **^{13}C NMR** (125 MHz, $CDCl_3$) δ 177.7 (C_q), 170.7 (C_q), 168.10 (C_q), 140.6 (C_q), 132.4 (C_q), 131.5 (C_q), 130.0 (CH), 129.0 (CH), 128.6 (CH), 126.8 (C_q), 125.9 (CH), 124.4 (CH), 124.2 (CH), 72.7 (C_q), 61.3 (CH_2), 52.2 (CH), 52.1 (CH_3), 52.0 (CH_3), 47.0 (C_q), 28.2 (CH_3), 27.3 (CH_3), 26.5 (CH_3); **ESI-HRMS** (m/z): $[M+H]^+$calcd for $C_{24}H_{32}NO_6$, 430.2224, found: 430.2222.

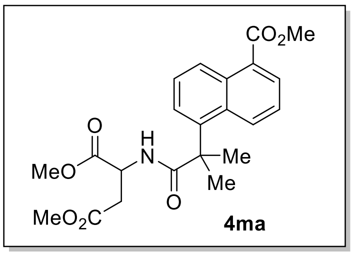

4ma

Dimethyl-(2-(5-(methoxycarbonyl)naphthalen-1-yl)-2-methylpropanoyl)aspartate (4ma)

Compound **4ma** was prepared following **Procedure E**, starting from dimethyl-(2-bromo-2-methylpropanoyl)aspartate (92.7 mg, 0.3 mmol) and 1-naphthoic acid (103 mg, 0.6 mmol). and 1,4-dioxane instead of ^{t}BuOH/HFIP. After purification, **4ma** was obtained as colorless liquid (84.7 mg, 68% yield); **^{1}H NMR** (500 MHz, $CDCl_3$) δ 8.80 (d, *J* = 8.6 Hz, 1H), 8.15 – 8.00 (m, 2H), 7.69 (d, *J* = 7.2 Hz, 1H), 7.65 – 7.53 (m, 1H), 7.46 (dd, *J* = 8.7, 7.2 Hz, 1H), 6.02 (d, *J* = 8.3 Hz, 1H), 4.76 (dt, *J* = 8.8, 4.6 Hz, 1H), 4.00 (s, 3H), 3.56 (s, 3H), 3.25 (s, 3H), 2.80 (dd, *J* = 17.1, 4.6 Hz, 1H), 2.60 (dd, *J* = 17.1, 4.7 Hz, 1H), 1.79 (s, 3H), 1.73 (s, 3H); **^{13}C NMR** (125 MHz, $CDCl_3$) δ 177.9 (C_q), 170.76 (C_q), 170.66 (C_q), 168.2 (C_q), 140.3 (C_q), 132.3 (C_q), 131.4 (C_q), 129.6 (CH), 129.0 (CH), 128.8 (CH), 127.0 (C_q), 126.0 (CH), 124.5 (CH), 124.4 (CH), 52.5 (CH), 52.3 (CH_3), 51.6 (CH_3), 48.5 (CH_3), 47.1 (C_q), 35.4 (CH_2), 28.1 (CH_3), 27.5 (CH_3); **ESI-HRMS** (m/z): $[M+H]^+$calcd for $C_{22}H_{26}NO_7$, 416.1704, found: 416.1700.

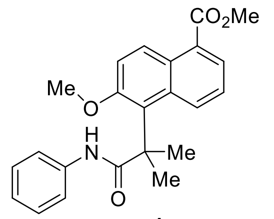

4na

Methyl-6-methoxy-5-(2-methyl-1-oxo-1-(phenylamino)propan-2-yl)-1-naphthoate (4na)

Compound **4na** was prepared following **Procedure E**, starting from 2-bromo-2-methyl-*N*-phenylpropanamide (72 mg, 0.3 mmol) and 6-methoxy-1-naphthoic acid (121 mg, 0.6 mmol). After purification, **4na** was obtained as yellow solid (73.5 mg, 65% yield); **^{1}H NMR** (400 MHz, $CDCl_3$) δ 8.88 (dd, *J* = 9.6, 0.9 Hz, 1H), 8.36 – 8.19 (m, 1H), 7.96 (dd, *J* = 7.2, 1.0 Hz, 1H), 7.51 – 7.38 (m, 2H), 7.25 – 7.18 (m, 4H), 7.03 (tt, *J* = 4.9, 3.5 Hz, 1H), 6.83 (s, 1H), 4.01 (s, 3H), 3.96 (s, 3H), 1.95 (s, 6H); **^{13}C NMR** (100 MHz, $CDCl_3$) δ 178.2 (C_q), 168.3 (C_q), 156.8 (C_q), 138.0 (C_q), 133.7 (C_q), 129.1 (CH), 128.7 (CH), 128.2 (CH), 127.8 (CH), 127.7 (C_q), 127.3 (C_q), 125.2 (CH), 124.1 (CH), 124.0 (CH), 120.0 (CH), 116.4 (C_q), 56.5 (CH_3), 52.3 (CH_3), 48.8 (C_q), 28.6 (CH_3); **ESI-HRMS** (m/z): $[M+H]^+$calcd for $C_{23}H_{24}NO_4$, 378.1699, found: 378.1696.

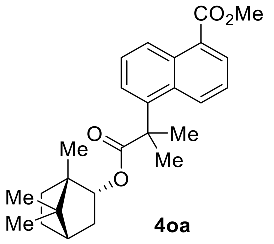

4oa

Methyl-5-(2-methyl-1-oxo-1-(((1*S*,2*R*,4*S*)-1,7,7-trimethylbicyclo[2.2.1]heptan-2-yl)oxy)propan-2-yl)-1-naphthoate (4oa)

Compound **4oa** was prepared following **Procedure E**, starting from (1*S*, 2*R*, 4*S*)-1,7,7-trimethylbicyclo[2.2.1]heptan-2-yl-2-bromo-2-methylpropanoate (90.6 mg, 0.3 mmol) and 1-naphthoic acid (103.2 mg, 0.6 mmol). After purification, **4oa** was obtained as colorless liquid (86.9 mg, 71% yield); **^{1}H NMR** (400 MHz, $CDCl_3$) δ 8.81 (d, *J* = 8.1 Hz, 1H), 8.21 – 8.05 (m, 2H), 7.68 – 7.54 (m, 2H), 7.48 (dd, *J* = 8.7, 7.2 Hz, 1H), 4.76 (ddd, *J* = 9.8, 3.3, 2.1 Hz, 1H), 4.00 (s, 3H), 2.31 – 2.19 (m, 1H), 1.79 (d, *J* = 1.7 Hz, 6H), 1.51 (t, *J* = 4.5 Hz, 1H), 1.48 – 1.39 (m, 1H), 1.37 – 1.22 (m, 1H), 1.11 – 0.99 (m, 1H), 0.91 – 0.83 (m, 1H), 0.82 (s, 3H), 0.72 (s, 3H), 0.64 – 0.55 (m, 1H), 0.46 (s, 3H); **^{13}C NMR** (125 MHz, $CDCl_3$) δ 178.7 (C_q), 168.3 (C_q), 141.3 (C_q), 132.1 (C_q), 131.5 (C_q), 129.3 (CH), 128.9 (CH), 128.5 (CH), 126.9 (C_q), 125.0 (CH), 124.1 (CH), 123.1 (CH), 80.5 (CH), 52.2 (CH_3), 48.4 (C_q), 47.4 (C_q), 46.6 (C_q), 44.6 (CH), 36.0 (CH_2), 27.7 (CH_3), 27.5 (CH_2), 26.4 (CH_2), 19.4 (CH_3), 18.7 (CH_3), 13.0 (CH_3); **ESI-HRMS (m/z)**: $[M]^+$calcd for $C_{26}H_{32}O_4$, 408.2300, found: 408.2290.

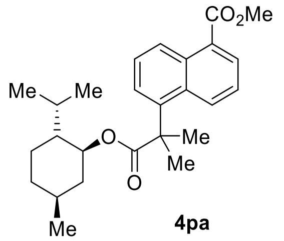

4pa

Methyl-5-(1-(((1*S*,2*S*,5*S*)-2-isopropyl-5-methylcyclohexyl)oxy)-2-methyl-1-oxopropan-2-yl)-1-naphthoate (4pa)

Compound **4pa** was prepared following **Procedure E**, starting from (2*S*,5*S*)-5-isopropyl-2-methylcyclohexyl-2-bromo-2-methylpropanoate (91 mg, 0.3 mmol) and 1-naphthoic acid (103 mg, 0.6 mmol). After purification, **4pa** was obtained as yellow liquid (78.7 mg, 64% yield); **^{1}H NMR** (500 MHz, $CDCl_3$) δ 8.56 (d, *J* = 8.1 Hz, 1H), 7.94 (d, *J* = 8.7 Hz, 1H), 7.86 (d, *J* = 7.2 Hz, 1H), 7.36 (q, *J* = 7.5 Hz, 2H), 7.28 – 7.21 (m, 1H), 4.39 (td, *J* = 10.8, 4.3 Hz, 1H), 3.79 (s, 3H), 1.71 (d, *J* = 11.9 Hz, 1H), 1.55 (d, *J* = 6.6 Hz, 6H), 1.39 – 1.27 (m, 2H), 0.97 (dt, *J* = 7.5, 6.1 Hz, 1H), 0.88 – 0.81 (m, 1H), 0.77 – 0.66 (m, 2H), 0.61 (d, *J* = 6.5 Hz, 3H), 0.51 – 0.41 (m, 2H), 0.35 (d, *J* = 7.0 Hz, 3H), 0.29 (d, *J* = 6.9 Hz, 3H); **^{13}C NMR** (125 MHz, $CDCl_3$) δ 178.0 (C_q), 168.3 (C_q), 141.2 (C_q), 132.1 (C_q), 131.6 (C_q), 129.4 (CH), 128.8 (CH), 128.6 (CH), 126.9 (C_q), 125.0 (CH), 124.1 (CH), 123.2 (CH), 74.9 (CH), 52.1 (CH_3), 46.7 (C_q), 46.5 (CH), 40.0 (CH), 34.0 (CH_2), 31.2 (CH), 27.8 (CH_2), 27.4 (CH_3), 25.4 (CH_3), 22.9 (CH_2), 21.9 (CH_3), 20.5 (CH_3), 15.7 (CH_3); **ESI-HRMS**

(m/z): [M-H]$^+$calcd for $C_{26}H_{33}O_4$, 409.2384, found: 409.2382.

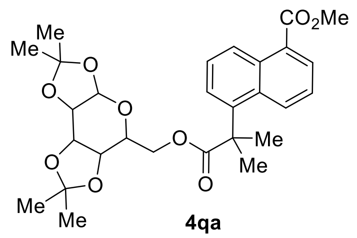

4qa

Methyl-5-(2-methyl-1-oxo-1-((2,2,7,7-tetramethyltetrahydro-5H-bis([1,3]dioxolo)[4,5-b:4',5'-d]pyran-5-yl)methoxy)propan-2-yl)-1-naphthoate (4qa)

Compound **4qa** was prepared following **Procedure E**, starting from (2,2,7,7-tetramethyltetrahydro-5H-bis([1,3]dioxolo)[4,5-b:4',5'-d]pyran-5-yl)methyl 2-bromo-2-methylpropanoate (122 mg, 0.3 mmol) and 1-naphthoic acid (103 mg, 0.6 mmol). After purification, **4qa** was obtained as yellow liquid (112.6 mg, 73% yield); **^{1}H NMR** (500 MHz, $CDCl_3$) δ 8.66 (d, *J* = 8.5 Hz, 1H), 8.01 – 7.95 (m, 2H), 7.52 (d, *J* = 7.2 Hz, 1H), 7.47 (t, *J* = 7.9 Hz, 1H), 7.41 – 7.36 (m, 1H), 5.33 (d, *J* = 4.9 Hz, 1H), 4.30 (dd, *J* = 7.9, 2.1 Hz, 1H), 4.18 – 4.10 (m, 2H), 4.04 (dd, *J* = 11.3, 5.1 Hz, 1H), 3.90 (s, 3H), 3.69 (t, *J* = 6.1 Hz, 1H), 3.60 (d, *J* = 8.0 Hz, 1H), 1.70 (s, 6H), 1.26 (d, *J* = 1.7 Hz, 6H), 1.19 (s, 3H), 1.08 (s, 3H); **^{13}C NMR** (125 MHz, $CDCl_3$) δ 178.2 (C_q), 168.3 (C_q), 140.9 (C_q), 132.1 (C_q), 131.5 (C_q), 128.8 (CH), 128.8 (CH), 128.7 (CH), 126.9 (C_q), 125.2 (CH), 124.4 (CH), 123.4 (C_q), 109.3 (C_q), 108.5 (CH), 96.0 (CH), 70.5 (CH), 70.4 (CH), 65.5 (CH), 63.5 (CH), 52.1 (CH_2), 46.5 (CH_3), 27.6 (C_q), 25.9 (CH_3), 25.7 (CH_3), 24.9 (CH_3), 24.0 (CH_3); **ESI-HRMS** (m/z): [M+H]$^+$calcd for $C_{28}H_{35}O_9$, 515.2276, found: 515.2267.

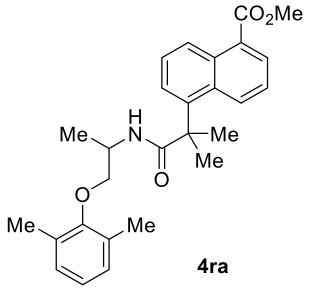

4ra

Methyl-5-(1-((1-(2,6-dimethylphenoxy)propan-2-yl)amino)-2-methyl-1-oxopropan-2-yl)-1-naphthoate (4ra)

Compound **4ra** was prepared following **Procedure E**, starting from 2-bromo-*N*-(1-(2,6-dimethylphenoxy)propan-2-yl)-2-methylpropanamide (98 mg, 0.3 mmol) and 1-naphthoic acid (103 mg, 0.6 mmol). After purification, **4ra** was obtained as yellow liquid (67.5 mg, 52% yield); **^{1}H NMR** (500 MHz, $CDCl_3$) δ 8.82 (d, *J* = 8.7 Hz, 1H), 8.19 (d, *J* = 8.7 Hz, 1H), 8.11 – 8.06 (m, 1H), 7.68 (d, *J* = 6.9 Hz, 1H), 7.58 (dd, *J* = 8.5, 7.4 Hz, 1H), 7.49 (dd, *J* = 8.6, 7.3 Hz, 1H), 6.89 – 6.79 (m, 3H), 5.68 (d, *J* = 8.4 Hz, 1H), 4.30 (ddd, *J* = 11.2, 6.7, 3.4 Hz, 1H), 4.01 (s, 3H), 3.51 (dd, *J* = 9.0, 2.9 Hz, 1H), 3.36 (dd, *J* = 9.0, 3.7 Hz, 1H), 1.77 (s, 3H), 1.76 (s, 9H), 1.15 (d, *J* = 6.8 Hz, 3H); **^{13}C NMR** (125 MHz, $CDCl_3$) δ 177.6 (C_q), 168.2 (C_q), 154.3 (C_q), 140.8 (C_q), 132.5 (C_q), 131.4 (C_q), 130.4 (CH), 130.0 (C_q), 129.3 (CH), 128.73 (CH), 128.66 (CH), 126.9 (C_q), 126.0 (CH), 124.4 (CH), 124.3 (CH), 123.9 (CH), 73.5 (CH_2), 52.3 (CH_3), 47.1 (CH), 45.4 (C_q), 28.1 (CH_3), 27.9 (CH_3), 17.3 (CH_3), 15.6 (CH_3); **ESI-HRMS** (m/z): [M+H]$^+$calcd for $C_{27}H_{32}NO_4$, 434.2326, found: 434.2318.

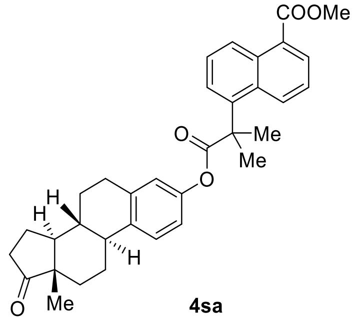

4sa

Methyl-5-(2-methyl-1-(((8*R*,9*S*,13*S*,14*S*)-13-methyl-17-oxo-7,8,9,11,12,13,14,15,16,17-decahydro-6H-cyclopenta[a]phenanthren-3-yl)oxy)-1-oxopropan-2-yl)-1-naphthoate (4sa)

Compound **4sa** was prepared following **Procedure E**, starting from (8*S*,9*R*,13*R*,14*R*)-13-methyl-17-oxo-7,8,9,11,12,13,14,15,16,17-

decahydro-6H-cyclopenta[a]phenanthren-3-yl-2-bromo-2-methylpropanoate (125 mg, 0.3 mmol) and 1-naphthoic acid (103 mg, 0.6 mmol). After purification, **4sa** was obtained as brown liquid (119.5 mg, 76% yield); **^{1}H NMR** (500 MHz, $CDCl_3$) δ 8.80 (d, *J* = 8.6 Hz, 1H), 8.27 (d, *J* = 8.7 Hz, 1H), 8.12 (d, *J* = 7.2 Hz, 1H), 7.67 (d, *J* = 7.2 Hz, 1H), 7.62 – 7.58 (m, 1H), 7.57 – 7.53 (m, 1H), 7.14 (d, *J* = 8.5 Hz, 1H), 6.53 (dd, *J* = 8.5, 2.2 Hz, 1H), 6.49 (d, *J* = 1.9 Hz, 1H), 4.00 (s, 3H), 2.83 – 2.76 (m, 2H), 2.47 (dd, *J* = 19.1, 8.7 Hz, 1H), 2.20 (d, *J* = 10.2 Hz, 1H), 2.15 – 2.06 (m, 1H), 2.04 – 1.93 (m, 2H), 1.89 (s, 6H), 1.63 – 1.51 (m, 2H), 1.51 – 1.39 (m, 4H), 1.39 – 1.26 (m, 2H), 0.86 (s, 3H); **^{13}C NMR** (125 MHz, $CDCl_3$) δ 177.6 (C_q), 168.3 (C_q), 148.5 (C_q), 140.5 (C_q), 137.7 (C_q), 137.2 (C_q), 132.2 (C_q), 131.5 (C_q), 128.9 (CH), 128.7 (CH), 127.0 (CH), 126.1 (C_q), 125.4 (CH), 124.5 (CH), 123.4 (CH), 121.1 (CH), 118.3 (CH), 52.2 (CH_3), 50.3 (C_q), 47.8 (C_q), 46.6 (C_q), 43.9 (C_q), 37.8 (C_q), 35.7 (CH_2), 31.4 (CH_2), 29.2 (CH_2), 27.6 (CH_3), 26.2 (CH_2), 25.6 (CH_2), 21.4 (CH_2), 13.7 (CH_3);

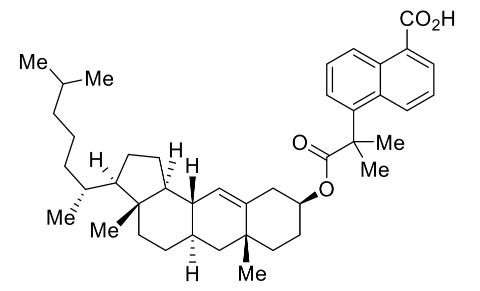

4ta

5-(1-(((3*R*,3a*R*,5a*R*,6a*R*,9*S*,11a*S*,11b*S*)-3a,6a-dimethyl-3-((*R*)-6-methylheptan-2-yl)-2,3,3a,4,5,5a,6,6a,7,8,9,10,11a,11b-tetradecahydro-1H-cyclopenta[a]anthracen-9-yl)oxy)-2-methyl-1-oxopropan-2-yl)-1-naphthoic acid (4ta)

Compound **4ta** was prepared following **Procedure E**, starting from (3*R*,8*R*,9*R*,10*S*,13*S*,14*R*,17*S*)-10,13-dimethyl-17-((*S*)-6-methylheptan-2-yl)-2, 3, 4, 7, 8, 9, 10, 11, 12, 13, 14, 15,16,17-tetradecahydro-1H-cyclopenta[a]phenanthren-3-yl-2-bromo-2-methylpropanoate (160 mg, 0.3 mmol) and 1-naphthoic acid (103 mg, 0.6 mmol). After purification, **4ta** was obtained as white solid (86.4 mg, 45% yield); **MP** = 196 – 198 ºC; **^{1}H NMR** (500 MHz, $CDCl_3$) δ 8.68 (dd, *J* = 7.1, 2.3 Hz, 1H), 8.00 (dd, *J* = 10.7, 8.0 Hz, 2H), 7.50 (q, *J* = 4.8 Hz, 2H), 7.39 (dd, *J* = 8.6, 7.3 Hz, 1H), 5.24 – 5.18 (m, 1H), 4.60 – 4.49 (m, 1H), 3.93 (s, 3H), 2.02 – 1.97 (m, 1H), 1.91 – 1.86 (m, 2H), 1.86 – 1.80 (m, 1H), 1.73 (dd, *J* = 8.5, 4.7 Hz, 1H), 1.67 (d, *J* = 0.9 Hz, 6H), 1.45 (ddd, *J* = 19.4, 11.8, 4.4 Hz, 4H), 1.35 (d, *J* = 8.5 Hz, 1H), 1.30 – 1.24 (m, 4H), 1.14 (dd, *J* = 20.0, 9.4 Hz, 4H), 1.03 (ddd, *J* = 30.9, 14.4, 8.6 Hz, 6H), 0.97 – 0.87 (m, 4H), 0.82 (d, *J* = 6.5 Hz, 3H), 0.78 (dd, *J* = 6.6, 2.4 Hz, 6H), 0.76 (s, 3H), 0.56 (s, 3H); **^{13}C NMR** (125 MHz, $CDCl_3$) δ 178.0 (C_q), 168.5 (C_q), 141.3 (C_q), 139.5 (C_q), 132.1 (C_q), 131.5 (C_q), 129.2 (CH), 128.9 (CH), 128.7 (CH), 126.9 (CH), 125.1 (C_q), 124.1 (CH), 123.3 (CH), 122.5 (CH), 74.5 (CH), 56.6 (CH), 56.1 (CH), 52.3 (CH_3), 49.9 (CH), 46.5 (C_q), 42.2 (C_q), 39.7 (CH_2), 39.5 (CH_2), 37.6 (CH_2), 36.8 (CH_2), 36.5 (C_q), 36.1 (C_q), 35.7 (CH), 31.8 (CH_2), 31.8 (CH), 28.2 (CH_2), 28.0 (CH_2), 27.7 (CH), 27.2 (CH_2), 24.2 (CH_2), 23.8 (CH_2), 22.8 (CH_3), 22.5 (CH_3), 20.9 (CH_2), 19.2 (CH_3), 18.7 (CH_3), 11.8 (CH_3); **ESI-HRMS** (m/z): $[M+H]^+$calcd for $C_{43}H_{61}O_4$, 641.4564, found: 641.4565.

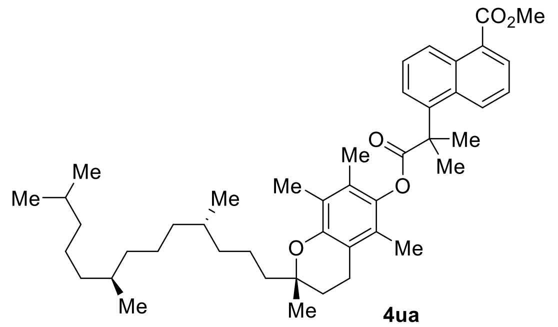

4ua

Methyl-5-(2-methyl-1-oxo-1-(((S)-2,5,7,8-tetramethyl-2-((4S,8S)-4,8,12-trimethyltridecyl)chroman-6-yl)oxy)propan-2-yl)-1-naphthoate (4ua)

Compound **4ua** was prepared following **Procedure E**, starting from (*S*)-2,5,7,8-tetramethyl-2-((4*S*,8*S*)-4,8,12-trimethyltridecyl)chroman-6-yl-2-bromo-2-methylpropanoate (173 mg, 0.3 mmol) and 1-naphthoic acid (103 mg, 0.6 mmol). After purification, **4ua** was obtained as white solid (104.6 mg, 51% yield); **MP** = 88 – 90 ºC; **^{1}H NMR** (400 MHz, $CDCl_3$) δ 8.90 (d, *J* = 8.6 Hz, 1H), 8.54 (d, *J* = 8.7 Hz, 1H), 8.17 (d, *J* = 7.2 Hz, 1H), 7.79 (d, *J* = 7.3 Hz, 1H), 7.72 – 7.62 (m, 1H), 7.58 – 7.47 (m, 1H), 4.05 (s, 3H),

2.50 (t, *J* = 7.0 Hz, 2H), 2.04 (s, 9H), 1.77 (dq, *J* = 13.8, 6.9 Hz, 2H), 1.64 (s, 3H), 1.58 (s, 3H), 1.56 – 1.50 (m, 2H), 1.47 – 1.37 (m, 4H), 1.35 – 1.26 (m, 8H), 1.26 – 1.17 (m, 6H), 1.11 (p, *J* = 6.6 Hz, 4H), 0.93 (s, 3H), 0.91 (s, 3H), 0.89 (d, *J* = 6.2 Hz, 6H); **^{13}C NMR** (100 MHz, $CDCl_3$) δ 176.2 (C_q), 168.3 (C_q), 149.2 (C_q), 140.3 (C_q), 140.2 (C_q), 132.3 (C_q), 131.9 (C_q), 130.2 (CH), 129.1 (CH), 128.6 (CH), 127.1 (C_q), 126.8 (C_q), 125.6 (C_q), 125.0 (C_q), 124.3 (CH), 123.8 (CH), 122.9 (CH), 117.2 (C_q), 74.9 (C_q), 52.2 (CH_3), 47.0 (C_q), 39.3 (CH_2), 37.4 (CH_2), 37.2 (CH_2), 32.7 (CH_2), 32.6 (CH_2), 28.1 (CH), 27.9 (CH_2), 24.7 (CH_3), 24.4 (CH), 22.7 (CH_3), 22.6 (CH_2), 20.9 (CH_3), 20.5 (CH_2), 19.7 (CH_3), 19.6 (CH_2), 12.8 (CH_3), 11.9 (CH_3), 11.7 (CH_3); **ESI-HRMS** (m/z): $[M+H]^+$calcd for $C_{45}H_{65}O_5$, 685.4827, found: 685.4816.

1.7. Control and Mechanistic Studies

A) No reaction without *ortho* C–H

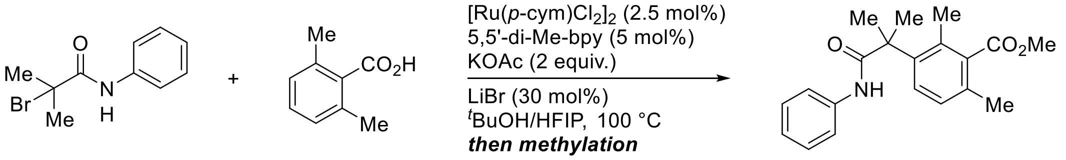

A 4 mL oven dried vessel was filled with 2-bromo-2-methyl-*N*-phenylpropanamide **1a** (0.3 mmol, 72.3 mg), 2,6-dimethylbenzoic acid **2tb** (0.6 mmol, 90.0 mg), [Ru(*p*-cym)Cl_2]$_2$ (4.6 mg, 2.5 mol%), 5,5'-di-Me-bpy (2.8 mg, 5 mol%), KOAc (58.8 mg, 2.0 equiv.), LiBr (7.8 mg, 30 mol%). The tube was flushed three times with N_2 and then ^{t}BuOH (1.8 mL) and HFIP (0.2 mL) were added. The mixture was stirred at 100 °C for 12 h. The solution was then cooled to room temperature, and MeI (5 equiv.), K_2CO_3 (2.0 equiv.) and NMP (2.0 mL) were added, the mixture was stirred at 60 °C for a further 2 h. After cooling to room temperature, and the product **3tb** was not determined by GC using tetradecane as internal standard, which confirms that the presence of *ortho* C–H bonds is vital for the *meta*-alkylation of aromatic carboxylic acids.

B) H/D exchange experiment

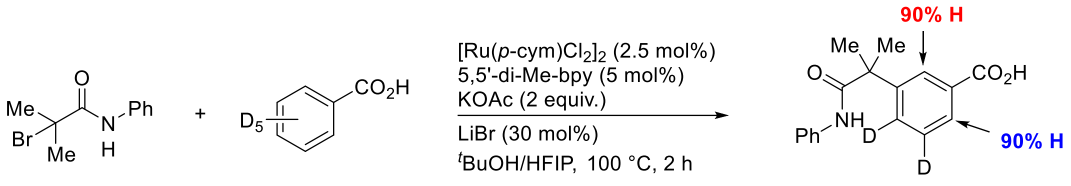

A 4 mL oven dried vessel was filled with 2-bromo-2-methyl-*N*-phenylpropanamide **1a** (0.1 mmol, 24.1 mg), deuterobenzoic acid **2a-[D₅]** (0.2 mmol, 25.4 mg), [Ru(*p*-cym)Cl_2]$_2$ (1.6 mg, 2.5 mol%), 5,5'-di-Me-bpy (0.9 mg, 5 mol%), KOAc (19.6 mg, 2.0 equiv.), LiBr (2.6 mg, 30 mol%). The tube was flushed three times with N_2 and then ^{t}BuOH (0.9 mL) and HFIP (0.1 mL) were added. The mixture was stirred at 100 °C for 2 h. After cooling to room temperature, the mixture was purified by silica gel column chromatography to afford the corresponding **3aa-[Dn]** (9.6 mg, 32% yield). This indicates that the *ortho* C–H metalation step takes place rapidly and that it is reversible.

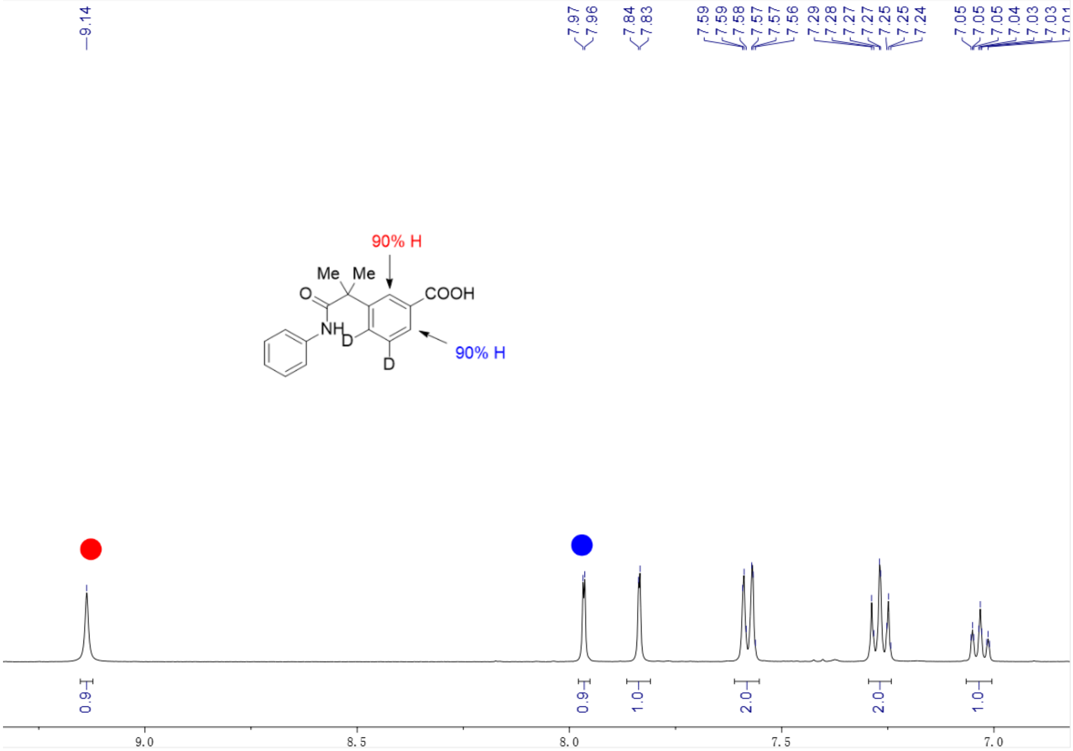

C) Kinetic isotopic effect study

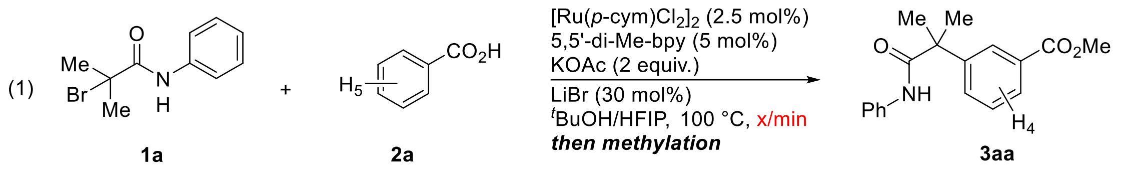

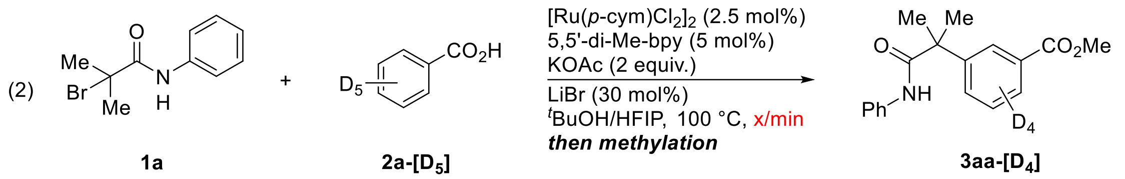

KIE by two parallel reactions: A mixture of 2-bromo-2-methyl-*N*-phenylpropanamide **1a** (0.1 mmol, 24.1 mg), **2a** (0.2 mmol, 24.4 mg), or deuterobenzoic acid **2a-[D$_5$]** (0.2 mmol, 25.4 mg), [Ru(*p*-cym)Cl$_2$]$_2$ (1.6 mg, 2.5 mol%), 5,5'-di-Me-bpy (0.9 mg, 5 mol%), KOAc (19.6 mg, 2.0 equiv.), LiBr (2.6 mg, 30 mol%). The tube was flushed three times with N_2 and then ^{t}BuOH (0.9 mL) and HFIP (0.1 mL) were added. The mixture was stirred at 100 °C for x min. 100 uL were taken into the reaction flask, MeI (5 equiv.), K_2CO_3 (2.0 equiv.) and NMP (1.0 mL) were added, the mixture was stirred at 60 °C for 2 h. After cooling to room temperature, the yields of **3aa or 3aa-[D$_4$]** were determined by GC using tetradecane as internal standard. A kinetic isotope effect value (KIE) of 1.1 was observed, which suggests that the reversible *ortho*-C−H bond insertion is in no way rate-limiting.

Time / min	**3aa / (yield) %**	**3aa-[D$_4$] / (yield) %**
0	0	0
15	6	6
30	10	9
45	15	13
60	16	15

Supplementary Table 8. Monitor the yield of product **3aa** and **3aa-[D$_4$]**.

Based on the above data, the graph is shown below:

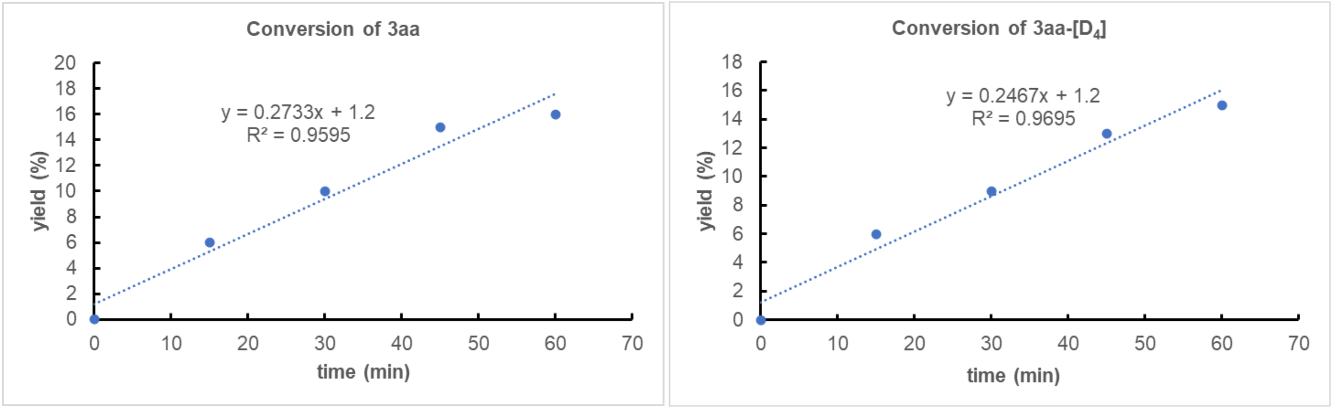

Supplementary Figure 8. Monitor the yield of product **3aa** and **3aa-[D$_4$].**

H/D exchange experiment (to indicate whether the ligand promotes C–H activation)

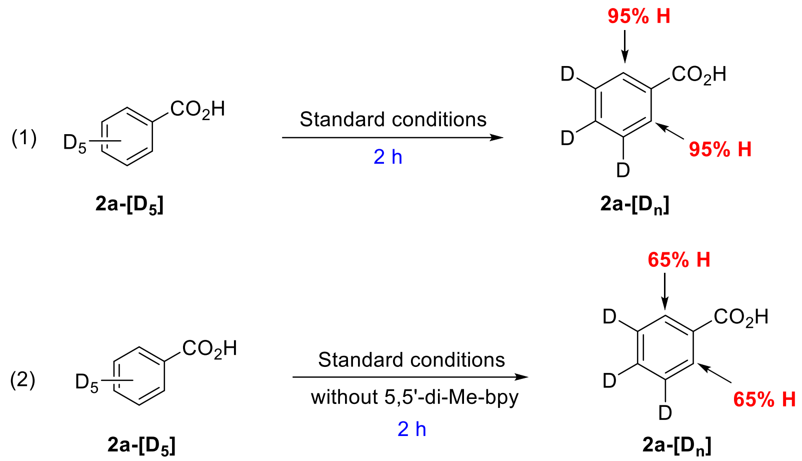

Two parallel reactions: a 4 mL oven dried vessel was filled with deuterobenzoic acid **2a-[D5]** (0.1 mmol, 12.7 mg), [Ru(*p*-cym)Cl_2]$_2$ (1.6 mg, 2.5 mol%), 5,5'-di-Me-bpy (0.9 mg, 5 mol%) or without 5,5'-di-Me-bpy (0.9 mg, 5 mol%), KOAc (19.6 mg, 2.0 equiv.), LiBr (2.6 mg, 30 mol%). The tube was flushed three times with N_2 and then ^{t}BuOH (0.9 mL) and HFIP (0.1 mL) were added. The mixture was stirred at 100 °C for 2 h. After cooling to room temperature, the mixture was purified by silica gel column chromatography to afford the corresponding **2a-[Dn]**. A substantial D/H scrambling was observed in both *ortho* position. When the Ligand 5,5'-di-Me-bpy is added, the kinetic isotope effect increases to 95% H in a one pot competitive experiment. When the Ligand 5,5'-di-Me-bpy is left out, the kinetic isotope effect reduced to 65% H in a one pot competitive experiment, indicating that one vital function of the bipyridine ligands is to accelerate the *ortho* C–H activation step.

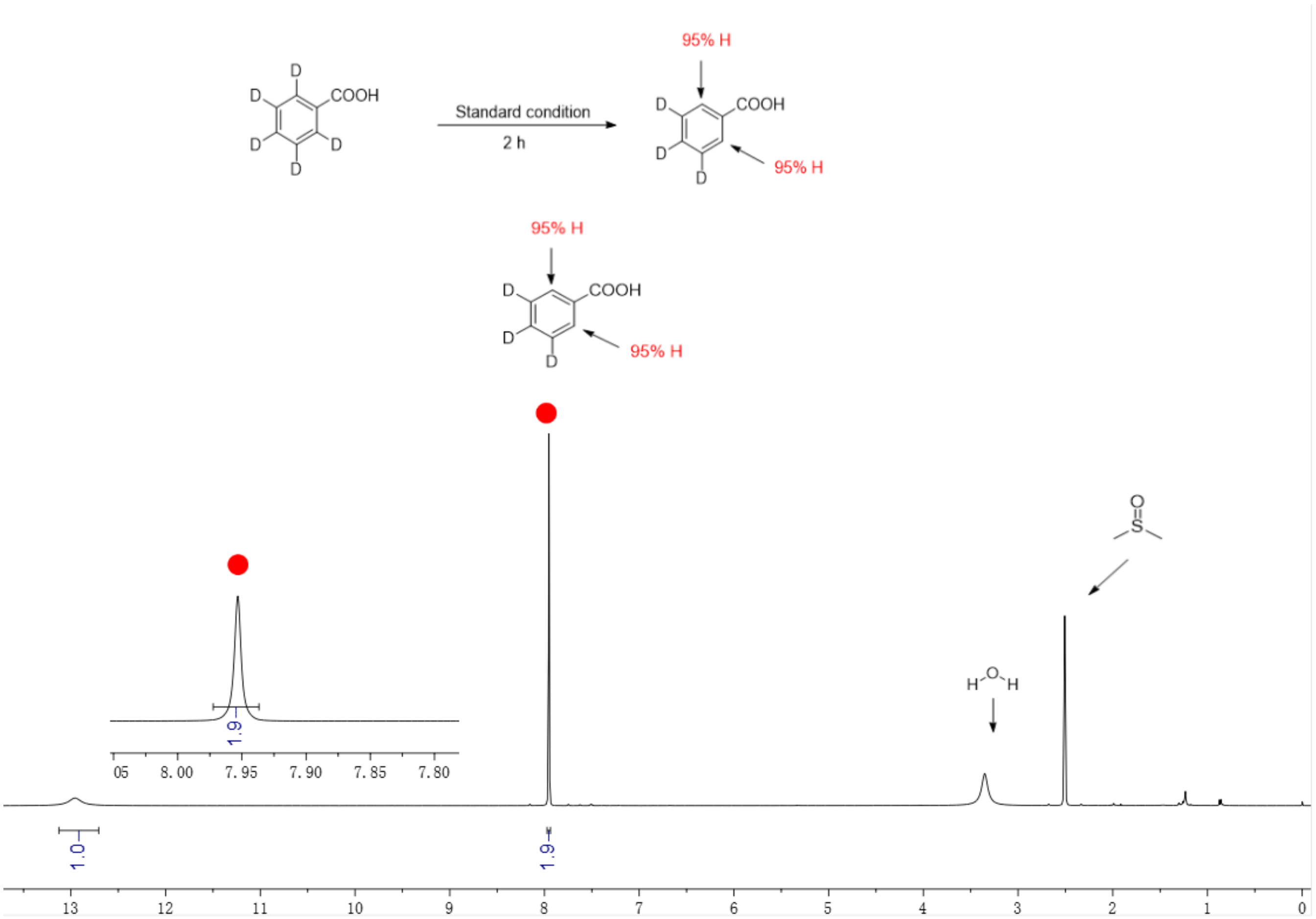

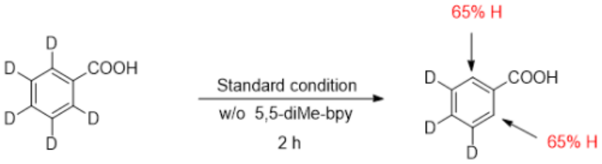

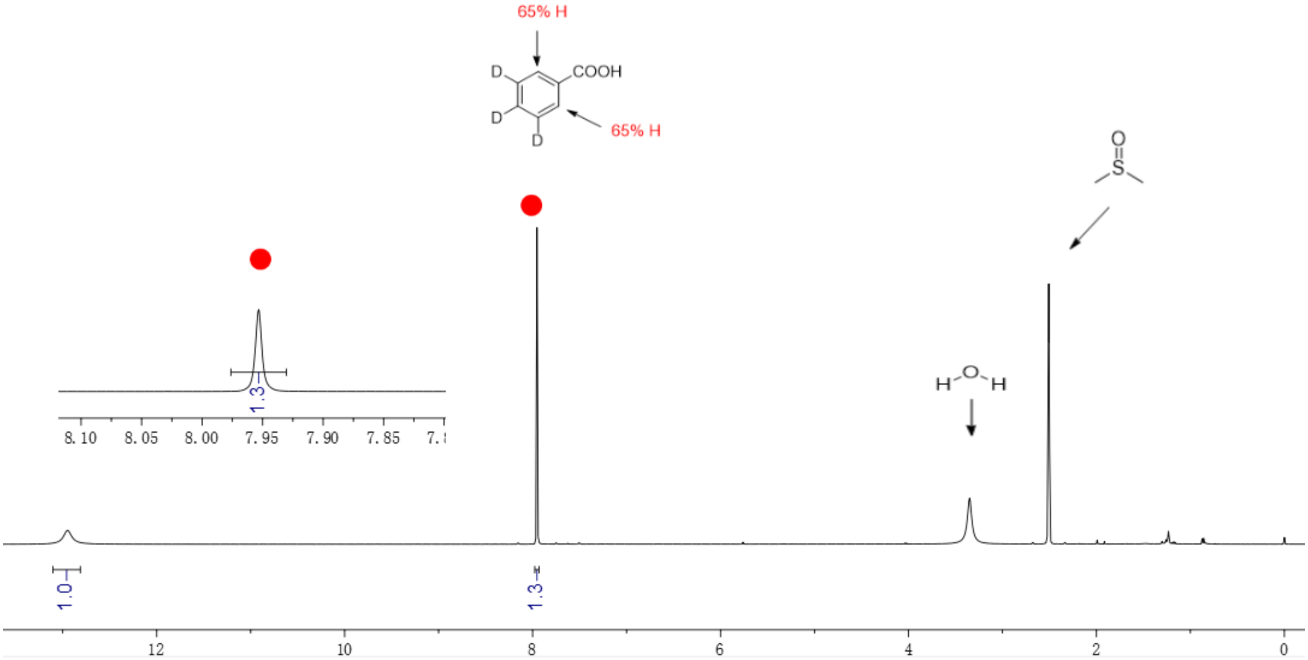

Synthesis of ruthenacycles (Ru-A) via C–H metallation

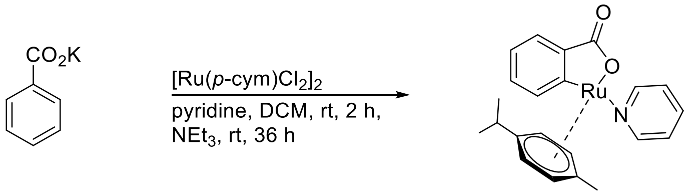

A solution of $[RuCl_2(p\text{-cym})]_2$ (612 mg, 1.0 mmol, 1.0 equiv.) and pyridine (163.0 µL, 2.0 equiv.) in dry DCM (30.0 mL) was stirred at 23 °C for 2 h. Subsequently potassium benzoate (800.0 mg, 5.0 mmol, 5.0 equiv.) and NEt_3 (2.0 mL) were added and the suspension stirred at 23 °C for further 48 h. Filtration over celite and purification by column chromatography (DCM/MeOH: 40/1 → 20/1) yielded the ruthenapentacycle complex (432 mg, 50% yield); **Physical state**: yellow solid; **^{1}H NMR** (400 MHz, $CDCl_3$) δ 8.52 – 8.44 (m, 2H), 7.99 (dd, *J* = 7.3, 1.1 Hz, 1H), 7.37 (dd, *J* = 7.5, 1.6 Hz, 1H), 7.31 (dt, *J* = 7.6, 1.6 Hz, 1H), 7.21 (td, *J* = 7.3, 1.6 Hz, 1H), 6.89 (tdd, *J* = 6.5, 3.5, 1.2 Hz, 3H), 5.48 (d, *J* = 5.6 Hz, 1H), 5.43 (d, *J* = 5.8 Hz, 1H), 5.20 (d, *J* = 5.9 Hz, 1H), 4.78 (d, *J* = 5.6 Hz, 1H), 2.34 (p, *J* = 6.9 Hz, 1H), 1.67 (s, 3H), 0.94 (t, *J* = 7.0 Hz, 6H); **^{13}C NMR** (100 MHz, $CDCl_3$) δ 181.2 (Cq), 176.0 (CH), 153.6 (CH), 138.5 (Cq), 136.7 (CH), 136.5 (Cq), 130.8 (Cq), 128.3 (CH), 124.2 (CH), 122.8 (CH), 102.3 (CH), 97.9 (CH), 87.7(CH), 86.4 (CH), 84.1 (CH), 79.9 (CH), 30.6 (CH), 22.4 (CH_3), 22.0 (CH_3), 17.8 (CH_3); The ruthenacycles **(Ru-A)** was prepared according the reported procedures[16].

D) Stoichiometric and catalytic reaction of Ru-A

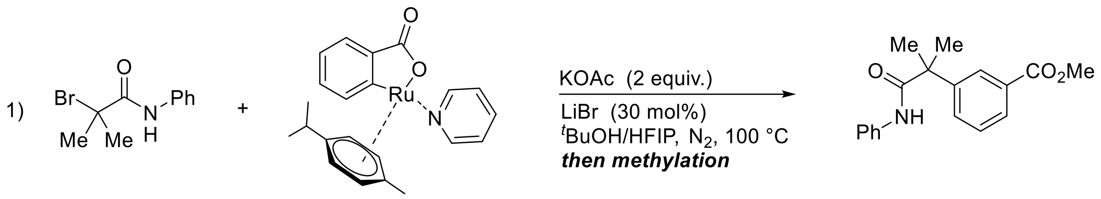

1a (0.1 mmol) **Ru-A** (0.2 mmol) **3aa**, n.d.

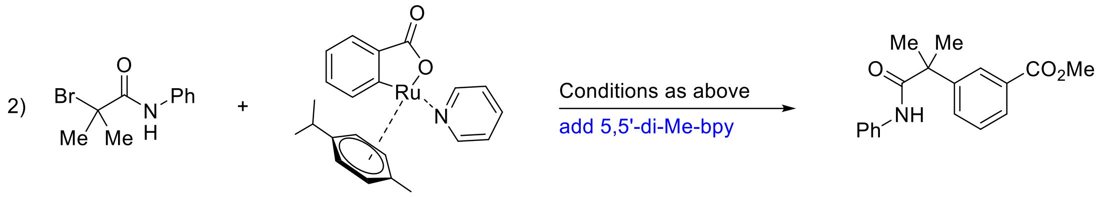

1a (0.1 mmol) **Ru-A** (0.2 mmol) **3aa**, 49%

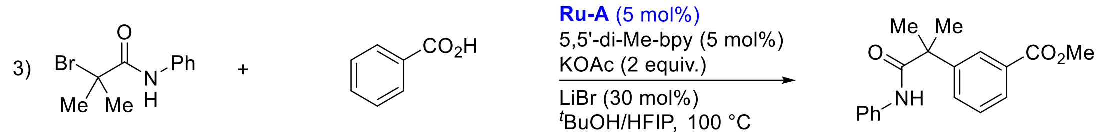

1a (0.1 mmol) **2a** (0.2 mmol) ***then methylation*** **3aa**, 76%

As shown in experiment 1): a mixture of 2-bromo-2-methyl-*N*-phenylpropanamide **1a** (24.1 mg, 0.1 mmol), **Ru-A** (87 mg, 0.2 mmol), KOAc (19.6 mg, 2.0 equiv.), LiBr (2.6 mg, 30 mol%). The tube was flushed three times with N_2, then ^{t}BuOH (0.9 mL), HFIP (0.1 mL) were added and the mixture was stirred at 100 °C for 12 hours. The solution was then cooled to room temperature, MeI (5 equiv.), K_2CO_3 (2.0 equiv.) and NMP (2 mL) were added and the mixture stirred at 60 °C for a further 2 h. After cooling to room temperature, **3aa** was not determined by GC using tetradecane as internal standard.

As shown in experiment 2): a mixture of 2-bromo-2-methyl-*N*-phenylpropanamide **1a** (24.1 mg, 0.1 mmol), **Ru-A** (87 mg, 0.2 mmol), 5,5-di-Me-bpy (18.4 mg, 0.1 mmol), LiBr (2.6 mg, 30 mol%). The tube was flushed three times with N_2, then ^{t}BuOH (0.9 mL), HFIP (0.1 mL) were added and the mixture was stirred at 100 °C for 12 hours. The solution was then cooled to room temperature, MeI (5 equiv.), K_2CO_3 (2.0 equiv.) and NMP (2 mL) were added and the mixture stirred at 60 °C for a further 2 h. After cooling to room temperature, **3aa** (49% yield) was determined by GC using tetradecane as internal standard.

As shown in experiment 3): a mixture of 2-bromo-2-methyl-*N*-phenylpropanamide **1a** (24.1 mg, 0.1 mmol), benzoic acid **2a** (24.4 mg, 0.2 mmol), **Ru-A** (2.2 mg, 5 mol%), 5,5-di-Me-bpy (0.9 mg, 5 mol%), KOAc (19.6 mg, 2.0 equiv.), LiBr (2.6 mg, 30 mol%). The tube was flushed three times with N_2, then ^{t}BuOH (0.9 mL), HFIP (0.1 mL) were added and the mixture was stirred at 100 °C for 12 hours. The solution was then cooled to room temperature and MeI (5 equiv.), K_2CO_3 (2.0 equiv.) and NMP (2.0 mL) were added, the mixture was stirred at 60 °C for a further 2 h. After cooling to room temperature, **3aa** (76% yield) was determined by GC using tetradecane as the internal standard. Combining the above experiments 1) and 2), when subjecting **1a** along with a pre-formed cyclometallated carboxylate complex **Ru-A** to the reaction conditions, no conversion was observed. Only when ligand 5,5-di-Me-bpy was added, the product **3aa** was formed in significant amounts. In combination with 5,5-di-Me-bpy, **Ru-A** has a comparably high catalytic activity as $[RuCl_2(p\text{-cym})]_2$. These findings support the intermediacy of *ortho*-benzoate ruthenacycles in the reaction and underline the vital importance of the bipyridine ligand 5,5-di-Me-bpy also for the steps following the *ortho*-metalation.

E) Trapping of intermediate with 1,1-diphenylethylene or BHT

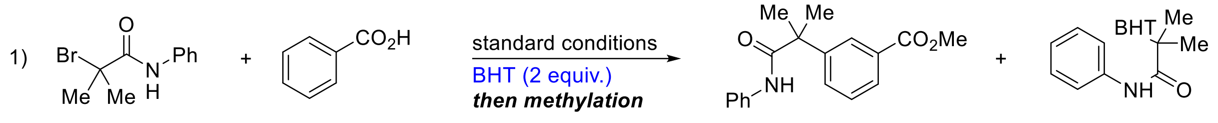

1a **2a** **3aa**, 15% **5aa**, 25%

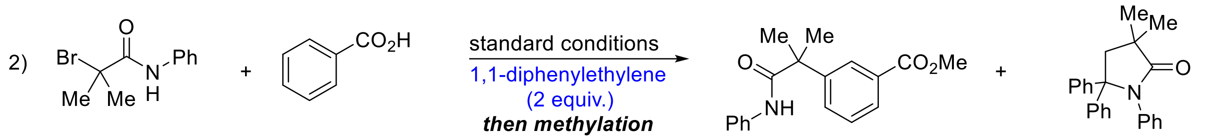

1a **2a** **3aa**, 14% **5ab**, 66%

A mixture of 2-bromo-2-methyl-*N*-phenylpropanamide **1a** (24.1 mg, 0.1 mmol), benzoic acid **2a** (24.4 mg, 0.2 mmol), Ru(*p*-cym)$Cl_2]_2$ (1.6 mg, 2.5 mol%), 5,5'-di-Me-bpy (0.9 mg, 5 mol%), KOAc (19.6 mg, 2.0 equiv.), LiBr (2.6 mg, 30 mol%) and butylated hydroxytoluene (BHT) (44 mg, 2 equiv.), the tube was flushed three times with N_2 and then ^{t}BuOH (0.9 mL), HFIP (0.1 mL) were added and the mixture was stirred at 100 °C for 12 hours. The solution was then cooled to room temperature and MeI (5 equiv.), K_2CO_3 (2.0 equiv.) and NMP (2 mL) were added, the mixture was stirred at 60 °C for a further 2 h. After cooling to room temperature, the yields of **3aa** (15% yield) were determined by GC using tetradecane as internal standard. And **5aa** (9.5 mg, 25% yield) was purified by column chromatography on silica gel. **^{1}H NMR** (500 MHz, $CDCl_3$) δ 7.42 – 7.38 (m, 2H), 7.27 – 7.24 (m, 2H), 7.15 (s, 1H), 7.05 (tt, *J* = 7.3, 1.2 Hz, 1H), 6.53 (s, 2H), 1.26 (s, 3H), 1.20 (s, 6H), 1.16 (s, 18H); **^{13}C NMR** (125 MHz, $CDCl_3$) δ 186.2 (C_q), 173.0 (C_q), 147.6 (C_q), 143.4 (C_q), 137.2 (CH), 129.0 (CH), 128.8 (CH), 124.6 (C_q), 120.1 (CH), 49.1 (C_q), 43.4 (C_q), 34.9 (CH), 30.2 (CH), 29.5 (CH), 21.9 (CH), 21.5 (CH); **ESI-HRMS** (m/z): $[M-H]^+$calcd for $C_{25}H_{34}NO_2$, 380.2595, found: 380.2597.

A mixture of 2-bromo-2-methyl-*N*-phenylpropanamide **1a** (24.1 mg, 0.1 mmol), benzoic acid **2a** (24.4 mg, 0.2 mmol), Ru(*p*-cym)$Cl_2]_2$ (1.6 mg, 2.5 mol%), 5,5'-di-Me-bpy (0.9 mg, 5 mol%), KOAc (19.6 mg, 2.0 equiv.), LiBr (2.6 mg, 30 mol%) and 1,1-diphenylethylene (36 mg, 2 equiv.), the tube was flushed three times with N_2, and then ^{t}BuOH (0.9 mL), HFIP (0.1 mL) were added, the mixture was stirred at 100 °C for 12 hours. The solution was then cooled to room temperature and MeI (5 equiv.), K_2CO_3 (2.0 equiv.) and NMP (2 mL) were added, the mixture was stirred at 60 °C for a further 2 h. After cooling to room temperature, **3aa** (14% yield) was determined by GC using tetradecane as internal standard. **5ab** (22.5 mg, 66% yield) was purified by column chromatography on silica gel. **^{1}H NMR** (500 MHz, $CDCl_3$) δ 7.43 – 7.38 (m, 6H), 7.33 (dd, *J* = 13.2, 5.7 Hz, 4H), 7.24 (t, *J* = 7.6 Hz, 4H), 7.15 (t, *J* = 7.4 Hz, 1H), 2.92 (s, 2H), 1.26 (s, 6H); **^{13}C NMR** (125 MHz, $CDCl_3$) δ 167.3 (C_q), 147.5 (C_q), 145.3 (C_q), 128.6 (CH), 128.5 (CH), 127.2 (CH), 124.9 (CH), 123.4 (CH), 122.6 (CH), 88.2 (C_q), 50.9 (CH_2), 41.5 (C_q), 27.6 (CH_3).

Combining the above experiments, when conducting the reaction in the presence of the radical scavenger BHT or 1,1-diphenylethylene, *meta*-alkylation was retarded, and alkyl radical-capture product was detected. This finding supports the proposed radical mechanism.

F) The rate of product 5ab formation w/wo 5,5'-di-Me-bpy

A comparative experiment: a mixture of 2-bromo-2-methyl-*N*-phenylpropanamide **1a** (24.1 mg, 0.1 mmol), 1,1-diphenylethylene **4va** (36.0 mg, 2 equiv.), Ru(*p*-cym)$Cl_2]_2$ (1.6 mg, 2.5 mol%), 5,5-di-Me-bpy (0.9 mg, 5 mol%) or without 5,5-di-Me-bpy (0.9 mg, 5 mol%), KOAc (19.6 mg, 2.0 equiv.), LiBr (2.6 mg, 30 mol%) and the tube was flushed three times with N_2 and then ^{t}BuOH (0.9 mL), HFIP (0.1 mL) were added, the resulting mixture was stirred at 100 °C for 0.5 hours, 1.0 hours, 1.5 hours, 2.0 hours, 5.0 hours and 12.0 hours. And take 100 uL into the reaction vessel, the GC yield of **5ab** was determined using tetradecane as the internal standard.

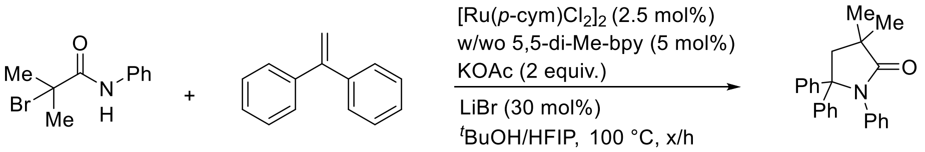

1a **4va** **5ab**

Time / h	With 5,5-di-Me-bpy, **5ab** / (yield)%	Without 5,5-di-Me-bpy, **5ab** / (yield) %
0.5	19	13
1	29	19

1.5	35	23
2.0	44	30
5.0	58	43
12.0	75	51

Supplementary Table 9. Monitor the yield of product **5ab** w/wo 5,5-di-Me-bpy.

A comparative experiment: a mixture of 2-bromo-2-methyl-*N*-phenylpropanamide **1a** (24.1 mg, 0.1 mmol), 1,1-diphenylethylene **4va** (36.0 mg, 2 equiv.), **Ru-A** (2.4 mg, 5 mol%), 5,5-di-Me-bpy (0.9 mg, 5 mol%) or without 5,5-di-Me-bpy (0.9 mg, 5 mol%), KOAc (19.6 mg, 2.0 equiv.), LiBr (2.6 mg, 30 mol%) and the tube was flushed three times with N_2 and then *t*BuOH (0.9 mL), HFIP (0.1 mL) were added, the resulting mixture was stirred at 100 °C for 0.5 hours, 1.0 hours, 1.5 hours, 2.0 hours, 5.0 hours and 12.0 hours. And take 100 uL into the reaction vessel, the GC yield of **5ab** was determined using tetradecane as the internal standard.

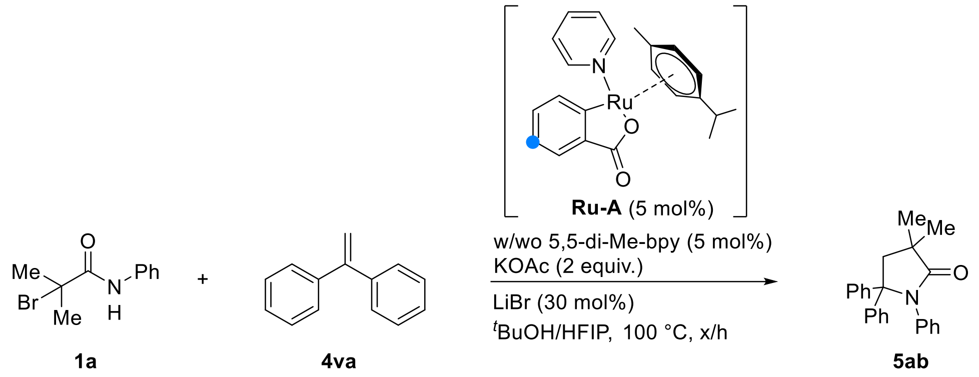

Time / h	With 5,5-di-Me-bpy, **5ab** / (yield)%	Without 5,5-di-Me-bpy, **5ab** / (yield) %
0.5	20	16
1	30	21
1.5	38	25
2.0	41	29
5.0	58	43
12.0	79	59

Supplementary Table 10. Monitor the yield of product **5ab** w/wo 5,5-di-Me-bpy.

The data processing is shown below: when the rate of the diphenylethylene adduct formation was monitored by in situ GC spectroscopy using a Ru-catalyst with and without ligand 5,5-di-Me-bpy, it was found that ligand 5,5-di-Me-bpy accelerates this reaction. The same observations were made when employing the cyclometallated complex **Ru-A** as a catalyst.

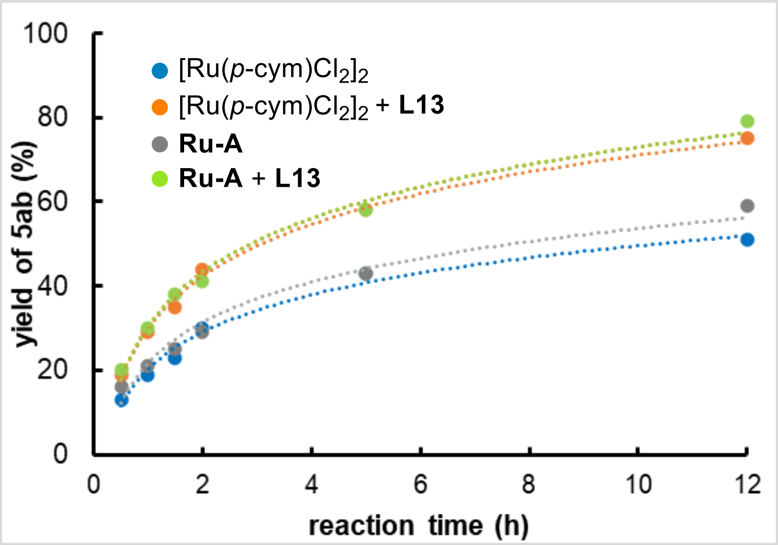

Supplementary Figure 9. Monitor the yield of product **5ab** When the [Ru(p-cym)Cl_2]$_2$ + **L13** or [Ru(p-cym)Cl_2]$_2$ or **Ru-A**+ **L13** or **Ru-A** was added, respectively.

G) Cyclic voltammograms for 1a, [Ru(p-cym)Cl_2]$_2$ + w/wo 5,5-di-Me-bpy and Ru-A+ w/wo 5,5-di-Me-bpy

Cyclic voltammetry was tested using a standard three-electrode system on Corrtest-CS310M electrochemical workstation at room temperature. Glassy carbon electrode served as the working electrode. Ag/AgCl (saturated KCl aqueous solution) electrode was severed as the reference electrode, and a platinum sheet served as the counter electrode. 0.1 M tetrabutylammonium perchlorate (TBAP) in ^{t}BuOH/HFIP was used as the electrolyte and the concentration of tested substances (**1a**, [Ru(p-cym)Cl_2]$_2$, [Ru(p-cym)Cl_2]$_2$ with 5,5-di-Me-bpy, **Ru-A**, **Ru-A** with 5,5-di-Me-bpy) was 1 mM. The scan rate was 100 mV s^{-1} and testing potential range was 0 to -2.5 V (vs. Ag/AgCl). It is implied by the experimental results that the reduction potential of the alkyl halide **1a** was determined as -1.01 V, and that of the [RuCl_2(p-cym)]$_2$ as -1.12 V. Thus, the [RuCl_2(p-cym)]$_2$ should already be able to reduce the alkyl halide to **1a**. However, addition of ligand **L13** should facilitate this step, as it increases the reduction potential to -1.17 V. The preformed pyridine-stabilized, cyclometallated complex **Ru-A** has an even higher reduction potential of -1.22 V.

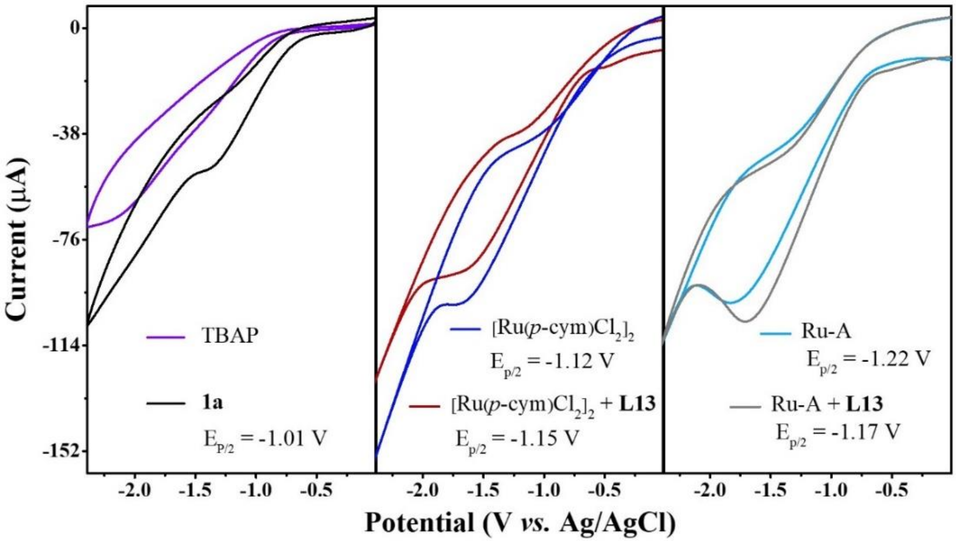

Supplementary Figure 10. Cyclic voltammograms (CV) data for **1a**, [Ru(p-cym)Cl_2]$_2$+ w/wo 5,5-di-Me-bpy, **Ru-A** + w/wo 5,5-di-Me-bpy.

(H) Cyclometalated Ruthenium complex (Ru-B) as catalyst

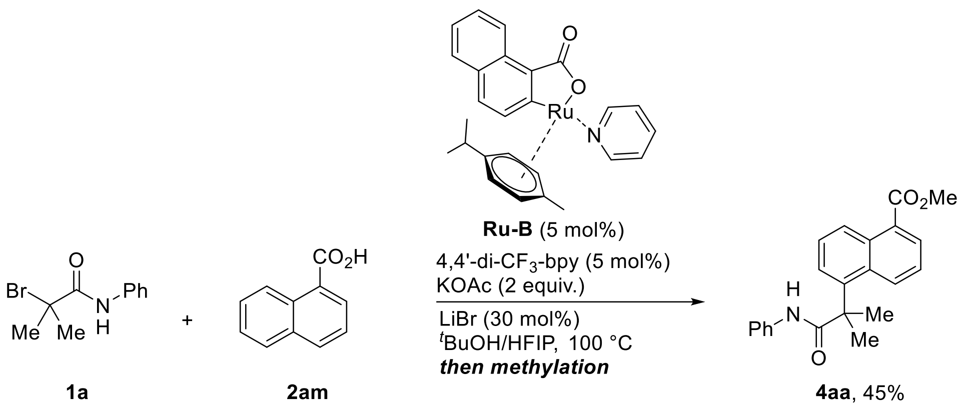

A mixture of 2-bromo-2-methyl-*N*-phenylpropanamide **1a** (24.1 mg, 0.1 mmol), 1-naphthoic acid **2am** (34.4 mg, 0.2 mmol), **Ru-B** (2.4 mg, 5 mol%), 4,4'-di-CF_3-bpy (1.5 mg, 5 mol%), KOAc (19.6 mg, 2.0 equiv.), LiBr (2.6 mg, 30 mol%). The tube was flushed three times with N_2, then ^{t}BuOH (0.9 mL), HFIP (0.1 mL) were added and the mixture was stirred at 100 °C for 12 hours. The solution was then cooled to room temperature and MeI (5 equiv.), K_2CO_3 (2.0 equiv.) and NMP (2.0 mL) were added, the mixture was stirred at 60 °C for a further 2 h. After cooling to room temperature, **4aa** (45% yield) was determined by GC using tetradecane as the internal standard. This implies that **Ru-B** has a comparably high catalytic activity as $[RuCl_2(p\text{-cym})]_2$.

(I) H/D exchange experiment in the presence of D_2O

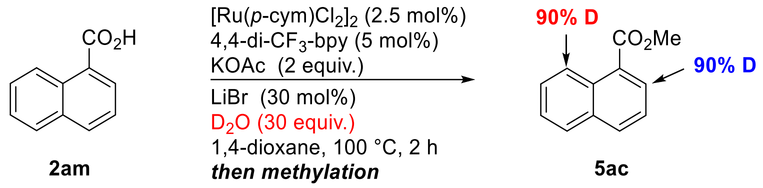

A 4 mL oven dried vessel was filled with 1-naphthoic acid **2am** (17.2 mg, 0.1 mmol), $[Ru(p\text{-cym})Cl_2]_2$ (1.6 mg, 2.5 mol%), 4,4'-di-CF_3-bpy (1.5 mg, 5 mol%), KOAc (19.6 mg, 2.0 equiv.), LiBr (2.6 mg, 30 mol%). The tube was flushed three times with N_2, then 1,4-dioxane (2.0 mL) and D_2O (30 equiv.) were added and the mixture was stirred at 100 °C for 2 h. The solution was then cooled to room temperature and MeI (5 equiv.), K_2CO_3 (2.0 equiv.) and NMP (2.0 mL) were added, the mixture was stirred at 60 °C for a further 2 h. After cooling to room temperature, the mixture was purified by silica gel column chromatography to give the corresponding **5ac**.

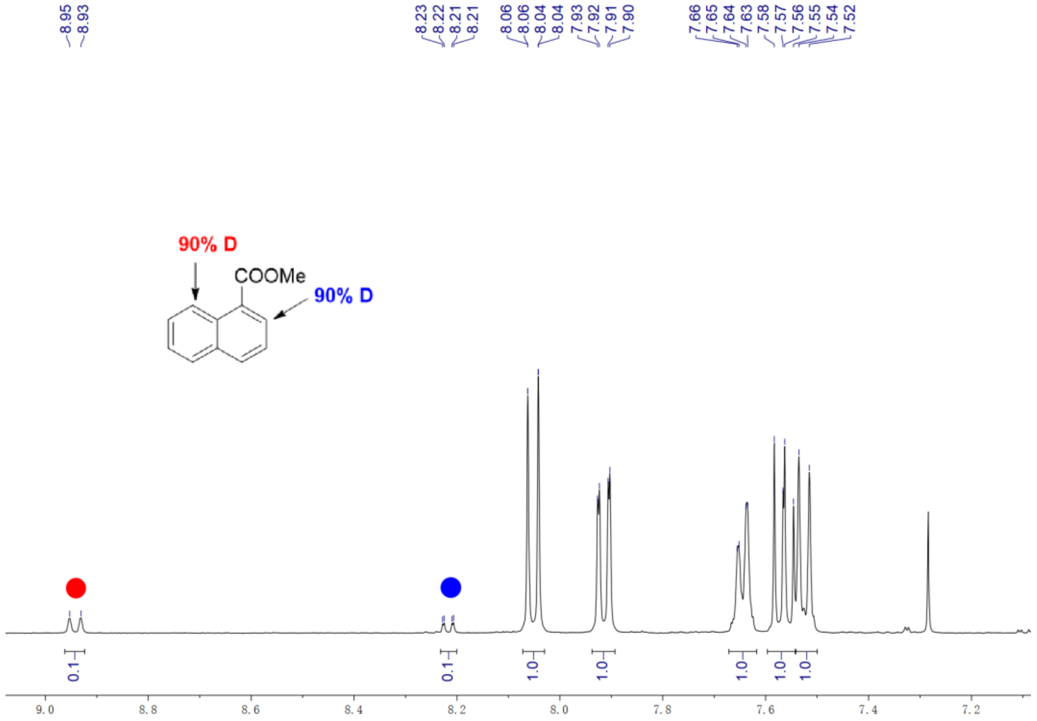

A 4 mL oven dried vessel was filled with 1-naphthoic acid **2am** (17.2 mg, 0.1 mmol), [Ru(*p*-cym)Cl_2]$_2$ (1.6 mg, 2.5 mol%), KOAc (19.6 mg, 2.0 equiv.), LiBr (2.6 mg, 30 mol%). The tube was flushed three times with N_2 and then 1,4-dioxane (1.0 mL) and D_2O (30 equiv.) were added, the mixture was stirred at 100 °C for 2 h. The solution was then cooled to room temperature and MeI (5 equiv.), K_2CO_3 (2.0 equiv.) and NMP (2.0 mL) were added and the mixture was stirred at 60 °C for a further 2 h. After cooling to room temperature, the mixture was purified by silica gel column chromatography to give the corresponding **5ac.**

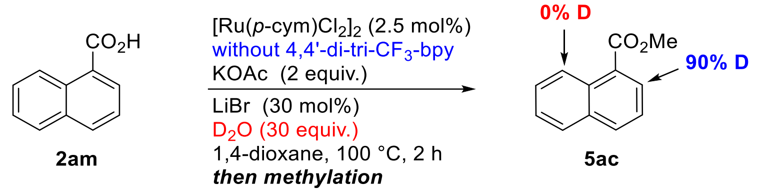

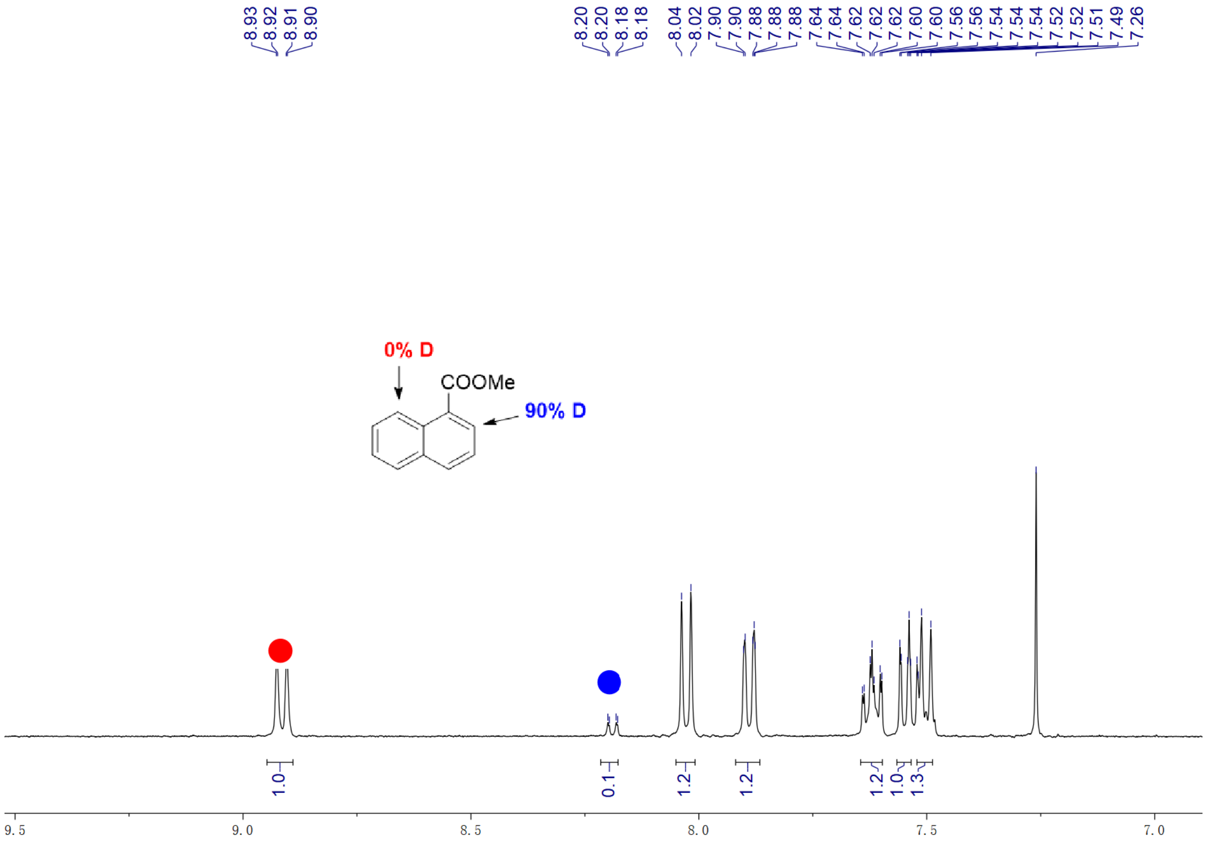

A 4 mL oven dried vessel was filled with 1-naphthoic acid **2am** (17.2 mg, 0.1 mmol), $RuCl_3$ (2.2 mg, 10 mol%), 4,4'-di-CF_3-bpy (2.9 mg, 10 mol%), KOAc (19.6 mg, 2.0 equiv.), LiBr (2.6 mg, 30 mol%). The tube was flushed three times with N_2 and the 1,4-dioxane (1 mL) and D_2O (30 equiv.), the mixture was stirred at 100 °C for 2 h. The solution was then cooled to room temperature and added MeI (5 equiv.), K_2CO_3 (2.0 equiv.), NMP (2.0 mL), the mixture was stirred at 60 °C for a further 2 h. After cooling to room temperature, the mixture was purified by silica gel column chromatography to give the corresponding **5ac**.

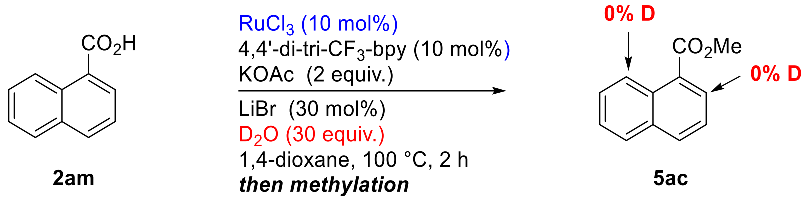

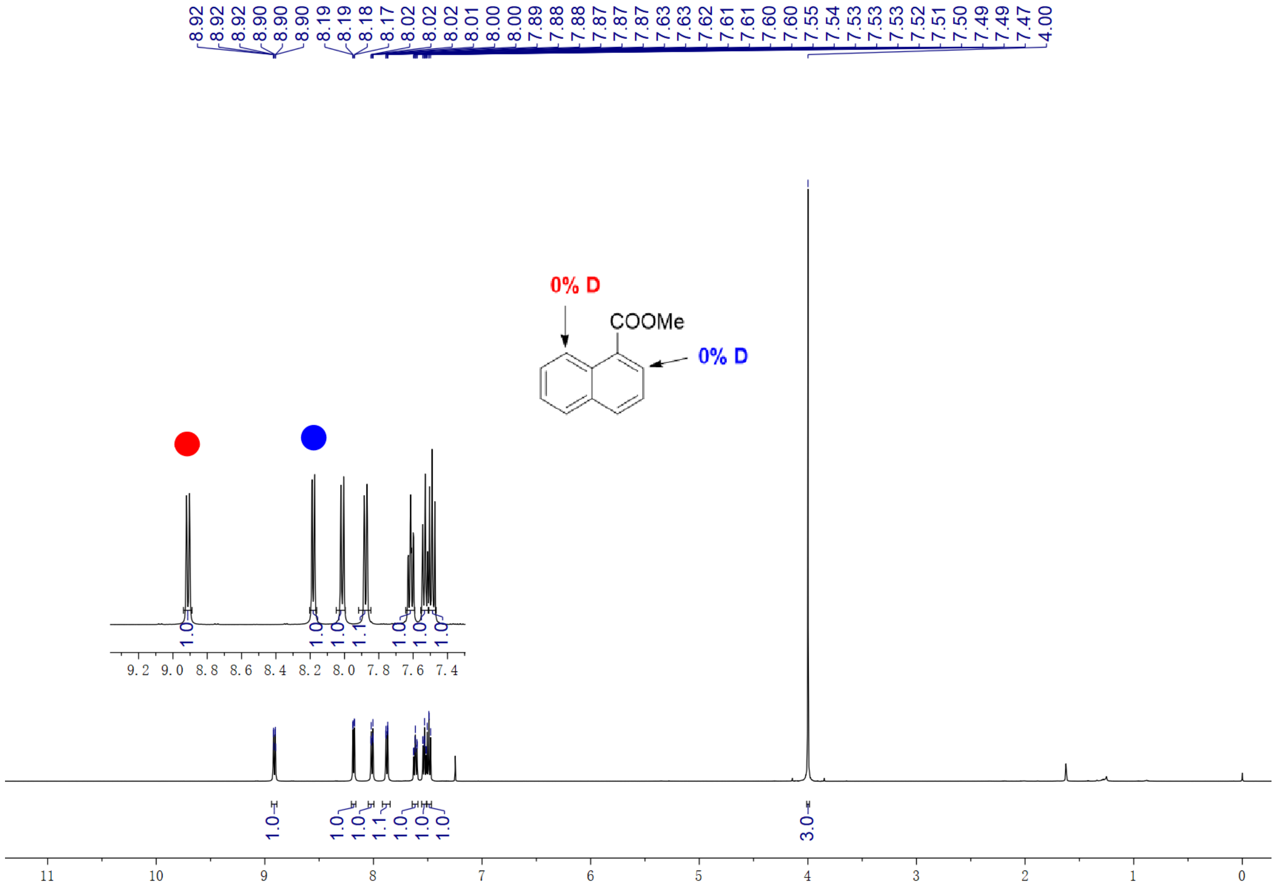

(J) Detection of free *p*-cymene (7)

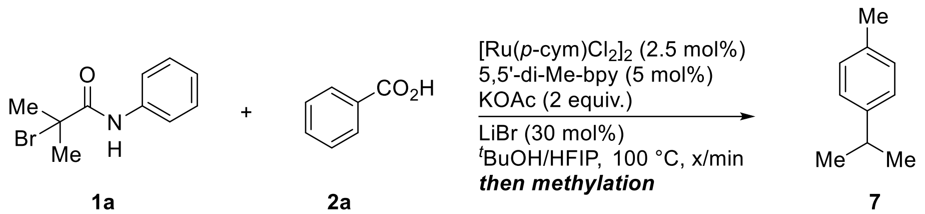

A mixture of 2-bromo-2-methyl-*N*-phenylpropanamide **1a** (72.3 mg, 0.3 mmol), benzoic acid **2a** (73.2 mg, 0.6 mmol), [Ru(*p*-cym)Cl_2]$_2$ (4.6 mg, 2.5 mol%), 5,5-di-Me-bpy (2.7 mg, 5 mol%), KOAc (58.8 mg, 2.0 equiv.), LiBr (7.8 mg, 30 mol%). The tube was flushed three times with N_2, then ^{t}BuOH (1.8 mL), HFIP (0.2 mL) were added and the mixture was stirred at 100 °C for x hours. During the course of the reaction, an aliquot of 100 µL was removed via syringe after 0.25 h, 0.5 h, 1.0 h, 3.0 h, 5.0 h, 8.0 h and 12.0 h. After cooling to room temperature, the yields of **7** was determined by GC using tetradecane as the internal standard.

The data are as follows:

Time / h	Yield of **7 / (%)**
0.25	27
0.5	33
1.0	44
3.0	66
5.0	80
8.0	95

12.0	95

Supplementary Table 11. Detection yield of free *p*-cymene.

Based on the data, the figure is shown below, GC monitoring of the reaction revealed that *p*-cymene is liberated within the first minutes. which indicating the displacement of this ligand with 5,5-di-Me-bpy during catalyst activation.

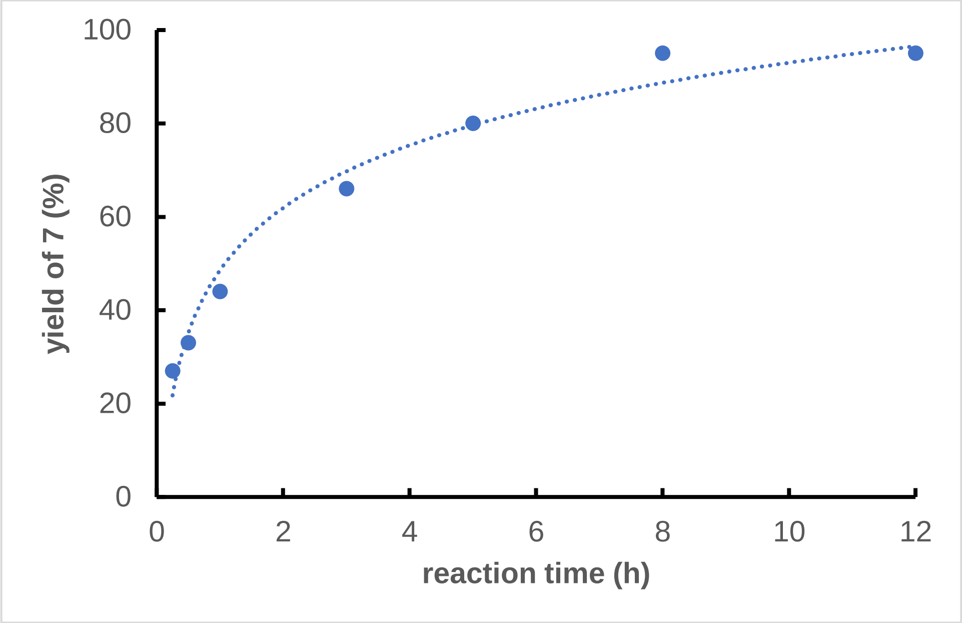

Supplementary Figure 11. Detection yield of free *p*-cymene.

1.8. Gram-Scale Reaction and Synthetic Application

Gram-scale reaction

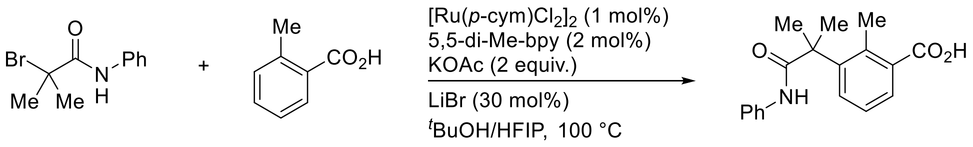

1a, 6 mmol **2a**, 12 mmol **3aa**, 1.21 g, 68%

2-bromo-2-methyl-*N*-phenylpropanamide **1a** (1.5 g, 6 mmol), 2-methylbenzoic acid **2a** (1.6 g, 12 mmol), [Ru(*p*-cym)Cl_2]$_2$ (36.8 mg, 1 mol%), 5,5-di-Me-bpy (22.1 mg, 2 mol%), KOAc (1.18 g, 2.0 equiv.), LiBr (234 mg, 30 mol%) were added to a 60 mL Schlenk tube. The tube was flushed three times with N_2, then ^{t}BuOH (36.0 mL) and HFIP (4.0 mL) was added. The mixture was stirred at 100 °C for 12 h. The solution was then cooled to room temperature and purified by column chromatography (PE/EA/HCOOH=80/20/1) on silica gel to give the pure products **3aa** (1.21 g, 68% yield).

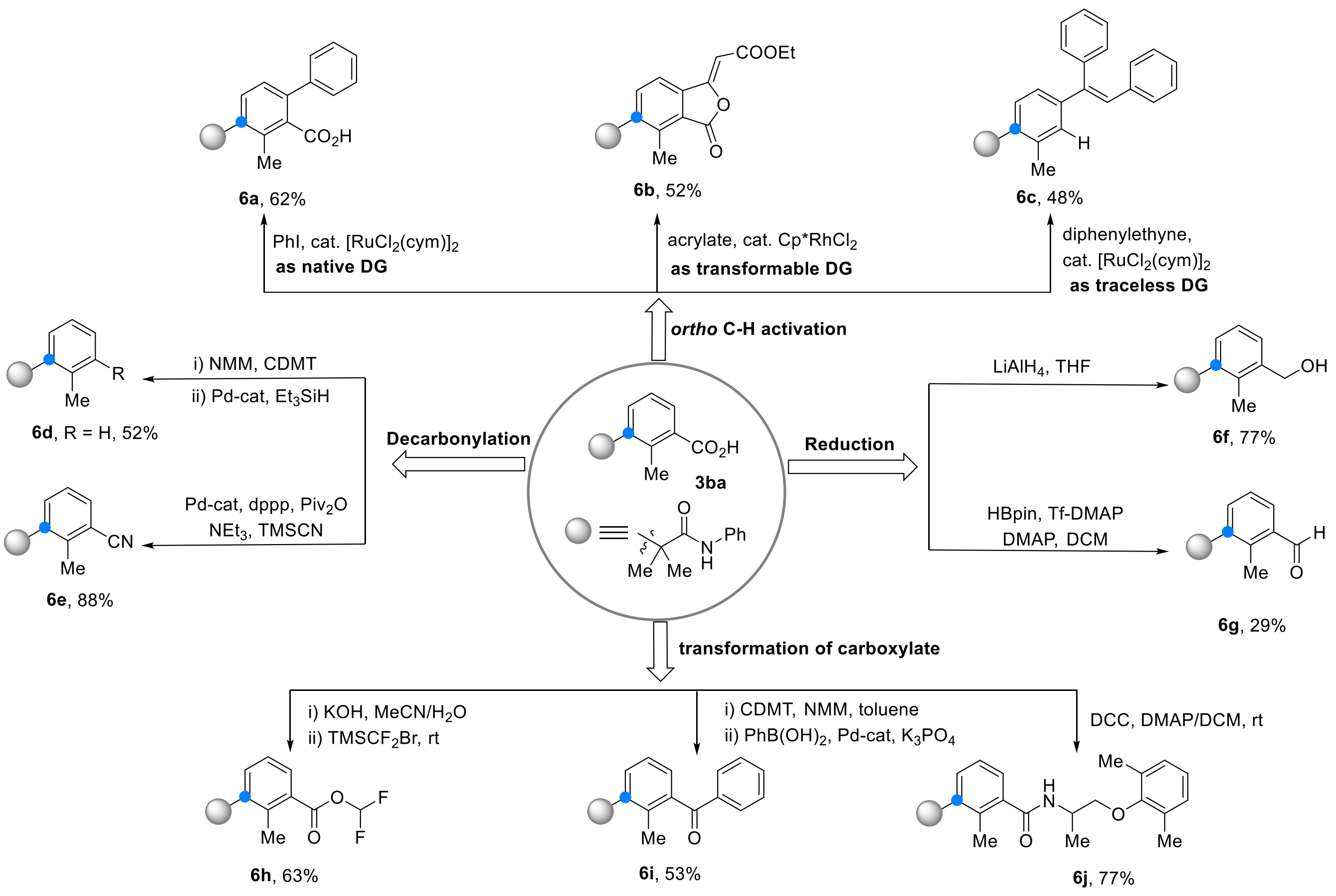

Supplementary Figure 12. Synthetic applications.

Application 1: carboxylic acid directed *ortho*-C(sp^2)-H arylation

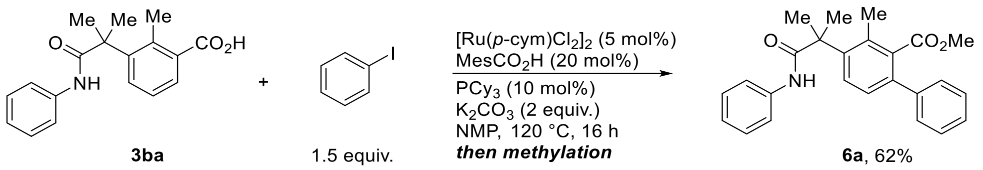

Based on references[17]: a 4 mL oven dried vessel was filled with $[Ru(p\text{-}cym)Cl_2]_2$ (9.2 mg, 5 mol%), $MesCO_2H$ (9.6 mg, 20 mol%), PCy_3 (8.4 mg, 10 mol%), K_2CO_3 (81.6 mg, 0.6 mmol), carboxylic acids **3ba** (89.1 mg, 0.30 mmol) and iodobenzene (91.8 mg, 0.45 mmol) in NMP (2.0 mL) were stirred under N_2 at 120 ºC for 16 h. The solution was then cooled to room temperature, K_2CO_3 (81.6 mg, 0.6 mmol) and MeI (62 uL, 1.5 mmol) were added and the mixture stirred at 60 ºC for a further 2 h. The mixture was then diluted with EA and washed with H_2O. The organic phase was dried over Na_2SO_4, and concentrated under vacuo. The residue was purified by column chromatography on silica gel (n-hexane/EtOAc) to give **6a** (72.0 mg, 62% yield). **Physical state**: white solid; **MP** = 127 – 129 ºC; **^{1}H NMR** (500 MHz, $CDCl_3$) δ 7.54 (d, J = 8.2 Hz, 1H), 7.36 – 7.27 (m, 7H), 7.23 (q, J = 8.3, 7.9 Hz, 3H), 7.01 (t, J = 7.4 Hz, 1H), 6.83 (s, 1H), 3.50 (s, 3H), 2.24 (s, 3H), 1.62 (s, 6H); **^{13}C NMR** (125 MHz, $CDCl_3$) δ 176.0 (C_q), 170.2 (C_q), 141.6 (C_q), 140.0 (C_q), 139.2 (C_q), 137.8 (C_q), 136.2 (C_q), 134.2 (CH), 128.9 (CH), 128.4 (CH), 128.2 (CH), 127.7 (CH, 2C), 127.2 (C_q), 124.4 (CH), 120.2 (CH), 52.0 (CH_3), 48.0 (C_q), 27.3 (CH_3), 17.0 (CH_3); **ESI-HRMS** (m/z): $[M\text{-}H]^+$calcd for $C_{25}H_{24}NO_3$, 386.1762, found: 386.1766.

Application 2: carboxylic acid directed *ortho*-C(sp^2)-H alkenyl cyclization to synthesis of (*E*)-3-ylidenephthalides

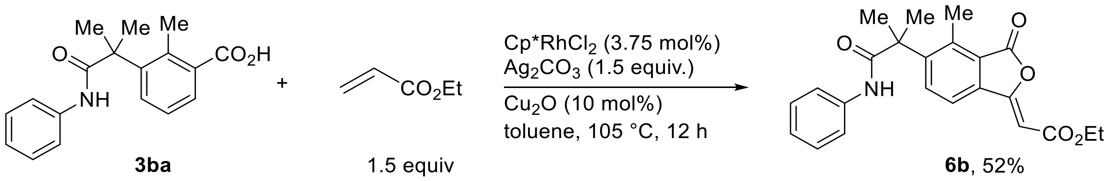

Based on references[18]: a 25 mL Schlenk tube was filled with $[Cp^*RhCl_2]_2$ (6.9 mg, 3.75 mol%), Ag_2CO_3 (124 mg, 0.45 mmol), carboxylic acids **3ba** (89 mg, 0.30 mmol), acrylates (45 mg, 0.45 mmol), Cu_2O (4.2 mg, 10 mol%) and toluene (1.0 mL). The mixture was stirred at 105 °C for 12 h. When the reaction was complete, the resulting mixture was cooled to room temperature and filtered through a short silica gel pad. The mixture was then concentrated in vacuo to give a residue which was purified by column chromatography on silica gel to give the corresponding product **6b** (61.0 mg, 52% yield). **Physical state**: yellow solid; **MP** = 175 – 177 ºC; **^{1}H NMR** (500 MHz, $CDCl_3$) δ 8.87 (d, J = 8.4 Hz, 1H), 7.87 (d, J = 8.4 Hz, 1H), 7.40 (d, J = 8.0 Hz, 2H), 7.33 – 7.22 (m, 2H), 7.10 (d, J = 7.4 Hz, 1H), 7.00 (s, 1H), 6.05 (s, 1H), 4.29 (q, J = 7.1 Hz, 2H), 2.69 (s, 3H), 1.71 (s, 6H), 1.37 (t, J = 7.1 Hz, 3H); **^{13}C NMR** (125 MHz, $CDCl_3$) δ 174.9 (C_q), 165.6 (C_q), 165.5 (C_q), 156.6 (C_q), 148.1 (C_q), 139.7 (C_q), 137.7 (C_q), 135.4 (C_q), 132.8 (CH), 129.0 (C_q), 125.9 (CH), 125.4 (CH), 124.6 (CH), 120.1 (CH), 101.9 (CH), 60.9 (CH_2), 48.2 (C_q), 27.3 (CH_3), 14.9 (CH_3), 14.2 (CH_3); **ESI-HRMS** (m/z): $[M-H]^+$calcd for $C_{23}H_{22}NO_5$, 392.1503, found: 392.1504.

Application 3: Carboxylic acid directed *ortho*-C(sp^2)-H react with internal alkynes for decarboxylative hydro-arylation

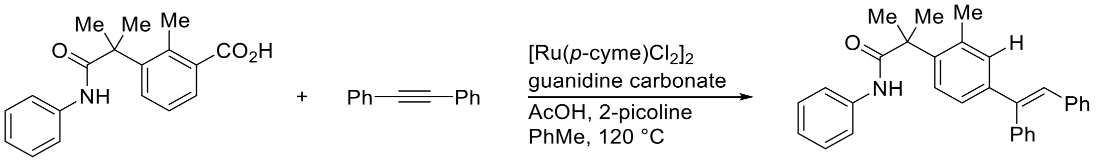

3ba

6c, 48%

Based on references[19]: $[Ru(p\text{-cym})Cl_2]_2$ (7 mg, 4 mol%), guanidine carbonate (11 mg, 20 mol%), carboxylic acids **3ba** (89 mg, 0.3 mmol) and diphenylacetylene (53 mg, 0.3 mmol) were added to a 25 mL Schlenk tube. After rinsing the vessel with 3 alternating cycles of vacuum and nitrogen, degassed toluene (2 mL), HOAc (17 μL, 0.3 mmol) and 2-picoline (6 μL, 20 mol%) were added by syringe. The resulting mixture was stirred at 120 °C for 24 h. When the reaction was complete, the mixture was cooled to RT, brine (20 mL) was added and the resulting mixture was extracted with ethyl acetate (3 × 20 mL). The combined organic layers were dried over $MgSO_4$, filtered and the volatile components were removed under reduced pressure. The residue was purified by column chromatography to give the hydroarylation product **6c** (62.0 mg, 48% yield). **Physical state**: white solid; **MP** = 132 – 135 ºC; **^{1}H NMR** (500 MHz, $CDCl_3$) δ 7.45 – 7.41 (m, 1H), 7.40 – 7.33 (m, 5H),7.30 – 7.26 (m, 2H), 7.25 – 7.17 (m, 3H), 7.14 – 7.11 (m, 3H), 7.09 – 7.06 (m, 2H), 7.04 – 6.99 (m, 3H), 6.93 (s, 1H), 2.29 (s, 3H), 1.65 (s, 6H); **^{13}C NMR** (125 MHz, $CDCl_3$) δ 176.3 (C_q), 142.4 (C_q), 141.6 (C_q), 141.3 (C_q), 140.0 (C_q), 138.0 (C_q), 137.1 (C_q), 131.2 (CH), 130.2 (CH), 129.5 (CH), 128.9 (CH), 128.7 (CH), 128.3 (CH), 127.9 (CH), 127.5 (CH), 126.8 (CH), 126.0 (CH), 125.5 (CH), 124.2 (CH), 119.8 (CH), 47.7 (C_q), 26.9 (CH_3), 20.4 (CH_3); **ESI-HRMS** (m/z): $[M-H]^+$calcd for $C_{31}H_{28}NO$, 430.2176, found: 430.2174.

Application 4: Decarboxylation of carboxylic acid

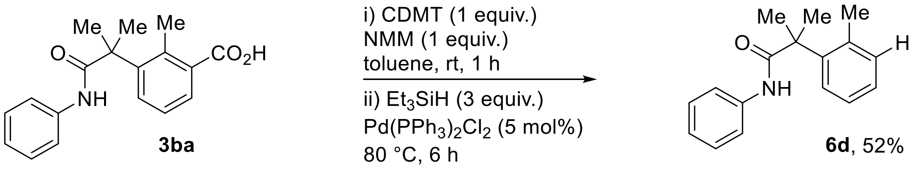

3ba

6d, 52%

Based on references[20], we made slight adjustments to the reaction conditions: a 25 mL Schlenk tube was filled with 2-chloro-4,6-dimethoxy1,3,5-triazine (CDMT) (53 mg, 0.3 mmol), carboxylic acids **3ba** (89 mg, 0.3 mmol), and 4-methylmorpholine (30 mg, 0.3 mmol) in toluene (3 mL) at room temperature for 1 h. After checking the completion of the rection by TLC, the flask was charged with $Pd(PPh_3)_2Cl_2$ (11 mg, 5 mol%) and $(CH_3CH_2)_3SiH$ (104 mg, 0.9 mmol). The mixture was stirred and heated at 80 °C for 6 h. After cooling to room temperature, the mixture was purified by silica gel column chromatography to afford the aldehydes and the yields were calculated: **6d** (39.5 mg, 52% yield). **Physical state**: white solid; **MP** = 98 – 100 ºC; **^{1}H NMR** (500 MHz, $CDCl_3$) δ 7.50 (dd, J = 7.4, 1.9 Hz, 1H), 7.34 (dd, J = 8.7, 1.2 Hz, 2H), 7.30 – 7.26 (m, 2H), 7.27 – 7.22 (m, 2H), 7.20 (dd, J = 7.3, 2.0 Hz, 1H), 7.07 –7.04 (m, 1H), 6.87 (s, 1H), 2.31 (s, 3H), 1.65 (s, 6H); **^{13}C NMR** (125 MHz, $CDCl_3$) δ 176.4 (C_q), 142.0 (C_q), 138.0 (C_q), 137.2 (C_q), 132.4 (C_q), 128.9 (CH), 127.6 (CH), 126.4 (CH), 126.1 (CH), 124.1 (CH), 119.7 (CH), 47.9 (C_q), 26.9 (CH_3), 20.3 (CH_3); **ESI-HRMS** (m/z): $[M+H]^+$calcd for $C_{17}H_{20}NO$, 254.1539, found: 254.1535.

Application 5: Conversion of carboxylic acid to cyano group

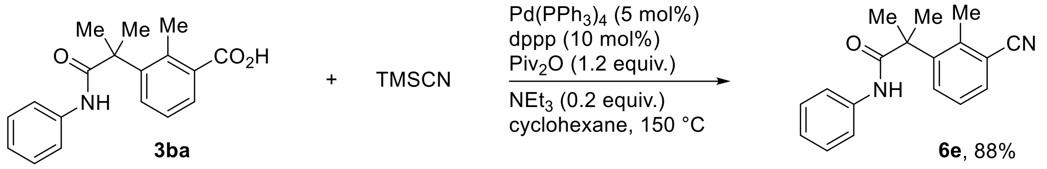

Based on references[21]: carboxylic acids **3ba** (89 mg, 0.3 mmol), TMSCN (45 mg, 0.45 mmol), $Pd(PPh_3)_4$ (17.4 mg, 5 mol%), dppp (12.3 mg, 10 mol%), NEt_3 (9 uL, 0.06 mmol), Piv_2O (67 mg, 0.36 mmol) and cyclohexane (2 mL) were added to a 25 mL Schlenk tube. The reaction mixture was heated at 120 °C for 12 h, then cooled to room temperature and concentrated in vacuo. The desired product was isolated and purified by column chromatography to give the corresponding **6e** (73 mg, 88% yield) as a yellow solid. **Physical state**: yellow solid; **MP** = 104 – 106 ºC; **^{1}H NMR** (500 MHz, $CDCl_3$) δ 7.74 (d, *J* = 8.0 Hz, 1H), 7.58 (d, *J* = 7.6 Hz, 1H), 7.36 (dd, *J* = 16.0, 7.9 Hz, 3H), 7.28 (t, *J* = 7.9 Hz, 2H), 7.09 (t, *J* = 7.3 Hz, 1H), 6.88 (s, 1H), 2.51 (s, 3H), 1.66 (s, 6H); **^{13}C NMR** (125 MHz, $CDCl_3$) δ 175.1 (C_q), 143.8 (C_q), 141.2 (C_q), 137.5 (C_q), 131.9 (CH), 130.5 (CH), 128.9 (CH), 126.9 (CH), 124.6 (CH), 120.1 (CH), 118.0 (C_q), 115.4 (C_q), 48.0 (C_q), 26.9 (CH_3), 18.5 (CH_3); **ESI-HRMS** (m/z): $[M+H]^+$calcd for $C_{18}H_{19}N_2O$, 279.1492, found: 279.1487.

Application 6: Reduction of carboxylic acid to benzyl alcohol

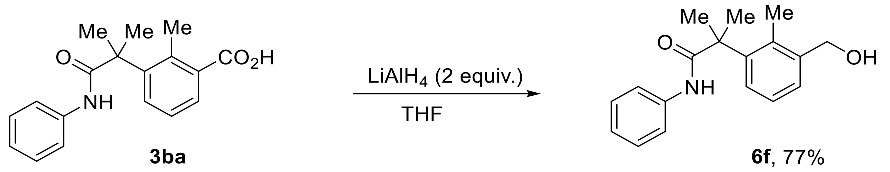

Based on references[22]: carboxylic acids **3ba** (89 mg, 0.3 mmol), THF (3 mL) was added to a 25 mL Schlenk tube under nitrogen and cooled to 0 °C, $LiAlH_4$ (0.6 mmol, 1mol/L) was slowly added to the solution, the reaction was allowed to warm to room temperature and stirred overnight. After completion of the reaction, it was quenched with aqueous NaOH solution (1.85 g H_2O + 1.85 g 15% NaOH + 1.85 g × 3 H_2O) and filtered through Celite, then rinsed twice with diethyl ether. The filtrate was concentrated in vacuo and purified by column chromatography to give the corresponding benzyl alcohol **6f** (66 mg, 77% yield) as a white solid. **Physical state**: white solid; **MP** = 110 – 113 ºC; **^{1}H NMR** (500 MHz, $CDCl_3$) δ 7.45 (d, *J* = 7.9 Hz, 1H), 7.41 (d, *J* = 7.4 Hz, 1H), 7.29 (t, *J* = 8.0 Hz, 3H), 7.25 – 7.20 (m, 2H), 7.04 (t, *J* = 7.4 Hz, 1H), 6.93 (s, 1H), 4.63 (s, 2H), 2.20 (s, 3H), 1.63 (s, 6H); **^{13}C NMR** (125 MHz, $CDCl_3$) δ 176.9 (C_q), 142.3 (C_q), 140.9 (C_q), 137.8 (C_q), 135.0 (C_q), 128.8 (CH), 126.8 (CH), 126.3 (CH), 125.5 (CH), 124.2 (CH), 119.9 (CH), 63.3 (CH_2), 48.0 (C_q), 27.4 (CH_3), 15.3 (CH_3); **ESI-HRMS** (m/z): $[M-H]^+$calcd for $C_{18}H_{20}NO_2$, 282.1499, found: 282.1499.

Application 7: Conversion of carboxylic acid to aldehyde

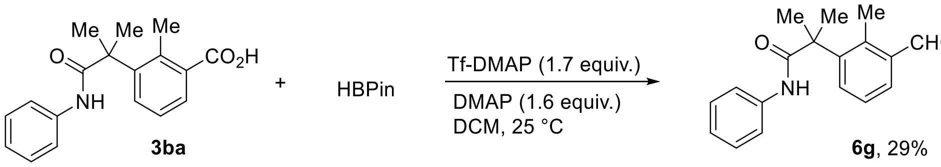

Based on references[23]: carboxylic acid **3ba** (89 mg, 0.3 mmol) was added to a 25 mL Schlenk tube, DMAP (1.6 equiv) and DCM (3 mL) were added under a dry nitrogen atmosphere. Tf-DMAP (1.7 equiv) and HBpin (69 mg, 1.8 equiv) were then added to the reaction mixture. After stirring for 4 h, the crude mixture was quenched with H_2O and extracted with DCM (3 x 3.0 mL). The combined organic layers were dried over anhydrous Na_2SO_4. After removal of the solvent under reduced pressure, the residue was purified by flash column chromatography on silica gel to give the desired aldehydes **6g** (25 mg, 29% yield). **Physical state**: colorless liquid; **^{1}H NMR** (500 MHz, $CDCl_3$) δ 10.37 (s, 1H), 7.83 (d, *J* = 7.7 Hz, 1H), 7.78 (d, *J* = 7.2 Hz, 1H), 7.47 (t, *J* = 7.8 Hz, 1H), 7.34 (d, *J* = 8.3 Hz, 2H), 7.28 (d, *J* = 7.5 Hz, 2H), 7.09 (t, *J* = 7.3 Hz, 1H), 6.80 (s, 1H), 2.63 (s, 3H), 1.70 (s, 6H); **^{13}C NMR** (125 MHz, $CDCl_3$) δ 192.7 (CH), 175.9 (C_q), 143.9 (C_q), 140.1 (C_q), 137.7 (C_q), 135.9 (C_q), 131.6 (CH), 130.9 (CH), 129.0 (CH), 126.8 (CH), 124.5 (CH), 119.9 (CH), 48.0 (C_q), 27.5 (CH_3), 16.0 (CH_3); **ESI-HRMS** (m/z): $[M-H]^+$calcd for $C_{18}H_{18}NO_2$, 280.1343, found: 280.1343.

Application 8: Reaction of carboxylic acid with $TMSCF_2Br$ access to difluoromethyl esters

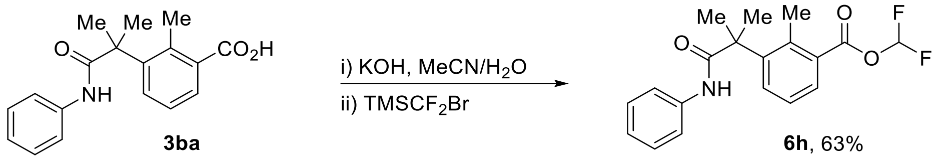

Based on references[24]: KOH (37 mg, 2.20 equiv, 0.66 mmol) and carboxylic acids **3ba** (89 mg, 0.3 mmol) were added to a 25 mL Schlenk tube under an air atmosphere. The vial was then sealed with a crimp top septum cap. MeCN (1 mL) was added at r.t., followed quickly by water (0.16 mL), and the mixture was stirred for 30 min. $TMSCF_2Br$ (120 mg, 2.00 equiv, 0.6 mmol) was then added and the reaction mixture stirred at r.t. for 2 hours. The vial was then opened, the reaction mixture was diluted with DCM (5 mL) and water (8 mL) was added. The organic layer was removed, the aqueous layer was extracted twice more with DCM (2 x 5 mL) and the organic extracts were washed separately with brine (10 mL). The combined organics were dried with Na_2SO_4, decanted and concentrated under vacuum. The crude product was dried on silica gel and purified by flash column chromatography to give the corresponding product **6h** (65 mg, 63% yield). Note: Due to its moderate volatility, care must be taken during evaporation of the solvent to avoid loss of product. **Physical state**: white solid; **MP** = 196 – 198 ºC; **^{1}H NMR** (500 MHz, $CDCl_3$) δ 7.92 (d, *J* = 7.8 Hz, 1H), 7.76 (d, *J* = 7.9 Hz, 1H), 7.41 – 7.36 (m, 1H), 7.31 (d, *J* = 7.9 Hz, 2H), 7.25 – 7.21 (m, 2H), 7.10 – 7.05 (m, 1H), 6.80 (s, 1H), 2.50 (s, 3H), 1.66 (s, 6H); **^{13}C NMR** (125 MHz, $CDCl_3$) δ 175.8 (C_q), 163.1 (C_q), 144.2 (C_q), 140.7 (C_q), 137.6 (C_q), 131.5 (CH), 130.4 (CH), 129.2 (CH), 128.9 (CH), 125.5 (d, *J* = 247.5 Hz, C_q), 120.1 (CH), 114.8 (CH), 112.8 (C_q), 110.7 (CH), 48.2 (C_q), 27.4 (CH_3), 17.9 (CH_3); **^{19}F NMR** (471 MHz, $CDCl_3$) δ -91.5, -91.7; **ESI-HRMS** (m/z): $[M-H]^+$calcd for $C_{19}H_{18}F_2NO_3$, 346.1260, found: 346.1258.

Application 9: Conversion of carboxylic acid to ketone and decarboxylation followed by arylation

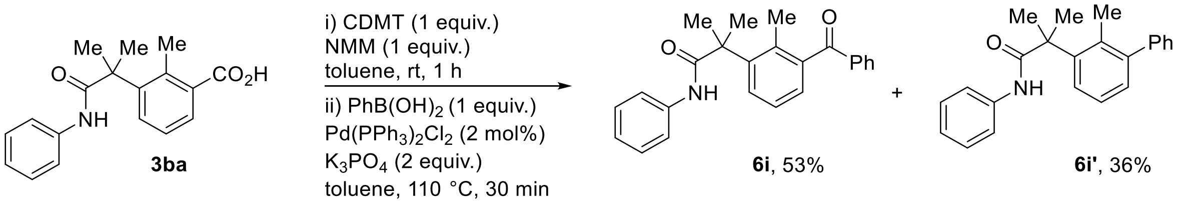

Based on references[25]: a 25 mL Schlenk tube was filled with 2-chloro-4,6-dimethoxy-1,3,5-triazine (CDMT) (53 mg, 0.3 mmol), carboxylic acids **3ba** (89 mg, 0.3 mmol) and 4-methylmorpholine (30 mg, 0.3 mmol) in toluene (3 mL) and stirred for 1 h at room temperature. After monitoring the completion of the reaction by TLC, the flask was charged with $Pd(PPh_3)_2Cl_2$ (4.4 mg, 0.02 mmol), K_3PO_4 (127 mg, 0.6 mmol) and phenylboronic acid (36.6 mg, 0.3 mmol) before standard cycles of evacuation and refilling with dry nitrogen. The mixture was stirred and heated at 110 °C for 30 min. After cooling to room temperature, the mixture was filtered through a short pad of silica gel, the silica gel was washed with DCM (3 × 15 mL) and the organic phases were combined. After removal of the solvent, the crude product was purified by silica gel column chromatography to give the corresponding ketone derivatives **6i** (56.8 mg, 53% yield) and **6i’** (35.5 mg, 36% yield).

2-(3-Benzoyl-2-methylphenyl)-2-methyl-*N*-phenylpropanamide (6i)

Physical state: white solid; **MP** = 122 – 124 ºC; **^{1}H NMR** (500 MHz, $CDCl_3$) δ 7.81 – 7.74 (m, 2H), 7.66 (d, *J* = 7.4 Hz, 1H), 7.58 (t, *J* = 7.4 Hz, 1H), 7.43 (t, *J* = 7.8 Hz, 2H), 7.39 – 7.34 (m, 3H), 7.31 – 7.23 (m, 3H), 7.08 (t, *J* = 7.4 Hz, 1H), 6.92 (s, 1H), 2.18 (s, 3H), 1.71 (s, 6H); **^{13}C NMR** (125 MHz, $CDCl_3$) δ 199.0 (C_q), 176.1 (C_q), 143.3 (C_q), 142.0 (C_q), 137.8 (C_q), 136.9 (C_q), 134.8 (C_q), 133.7 (CH), 129.9 (CH), 128.9 (CH), 128.6 (CH), 127.6 (CH), 126.7 (CH), 126.2 (CH), 124.4 (CH), 119.9 (CH), 48.1 (C_q), 27.3 (CH_3), 17.3 (CH_3); **ESI-HRMS** (m/z): $[M+H]^+$calcd for $C_{24}H_{24}NO_2$, 358.1802, found: 358.1793;

2-Methyl-2-(2-methyl-[1,1'-biphenyl]-3-yl)-*N*-phenylpropanamide (6i’)

Physical state: white solid; **MP** = 174 – 177 ºC; **^{1}H NMR** (500 MHz, $CDCl_3$) δ 7.54 (d, *J* = 7.9 Hz, 1H), 7.42 – 7.35 (m, 4H), 7.35 – 7.30 (m, 2H), 7.28 (t, *J* = 7.9 Hz, 2H), 7.25 – 7.20 (m, 3H), 7.07 (t, *J* = 7.4 Hz, 1H), 7.00 (s, 1H), 2.13 (s, 3H), 1.70 (s, 6H); **^{13}C NMR** (125 MHz, $CDCl_3$) δ 176.7 (C_q), 144.7 (C_q), 142.6 (C_q), 142.2 (C_q), 138.0 (C_q), 134.9 (CH), 129.5 (CH), 129.1 (CH), 128.9 (C_q), 128.1 (CH), 126.9 (CH), 125.9 (CH), 125.4 (CH), 124.2 (CH), 119.8 (CH), 48.3 (C_q), 27.4 (CH_3), 18.1 (CH_3); **ESI-HRMS** (m/z): $[M+H]^+$calcd for $C_{23}H_{24}NO$, 330.1852, found: 330.1854.

Application 10: Conversion of carboxylic acid to amide

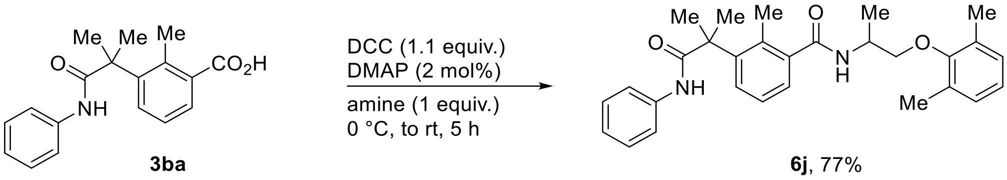

3ba 6j, 77%

Based on references[26]: carboxylic acid **3ba** (89 mg, 0.3 mmol) was added to a 25 mL Schlenk tube in DCM (5 ml), to which DCC (68 mg, 0.33 mmol), DMAP (0.7 mg, 0.006 mmol) and 1-(2,6-dimethylphenoxy)propan-2-amine (54 mg, 0.3 mmol) were added at 0° C. The reaction mixture was stirred at room temperature for 5 h and washed with brine. The organic layer was dried over Na_2SO_4, filtered and evaporated under reduced pressure to give the crude product, which was purified by column chromatography on silica gel to give the corresponding amide **6j** (105.8 mg, 77% yield). **Physical state**: white solid; **^{1}H NMR** (500 MHz, $CDCl_3$) δ 7.54 (d, J = 7.4 Hz, 1H), 7.37 – 7.27 (m, 4H), 7.24 – 7.19 (m, 2H), 7.03 (t, J = 7.4 Hz, 1H), 6.94 (d, J = 7.3 Hz, 2H), 6.90 – 6.87 (m, 2H), 6.28 (d, J = 8.4 Hz, 1H), 4.59 – 4.36 (m, 1H), 3.87 (dd, J = 9.2, 4.0 Hz, 1H), 3.74 (dd, J = 9.2, 3.2 Hz, 1H), 2.35 (s, 3H), 2.18 (s, 6H), 1.62 (d, J = 5.2 Hz, 6H), 1.45 (d, J = 6.8 Hz, 3H); **^{13}C NMR** (125 MHz, $CDCl_3$) δ 176.0 (C_q), 169.8 (C_q), 154.6 (C_q), 143.2 (C_q), 139.8 (C_q), 137.7 (C_q), 134.5 (C_q), 130.5 (C_q), 128.9 (CH), 128.8 (CH), 127.4 (CH), 126.4 (CH), 125.8 (CH), 124.3 (CH), 124.1 (CH), 120.0 (CH), 73.6 (CH_2), 48.0 (CH), 45.6 (C_q), 27.3 (CH_3), 27.1 (CH_3), 17.8 (CH_3), 16.7 (CH_3), 16.1 (CH_3); **ESI-HRMS** (m/z): $[M+H]^+$calcd for $C_{29}H_{35}N_2O_3$, 459.2642, found: 459.2633.

Application 11: Conversion of carboxylic acid to corresponding esters

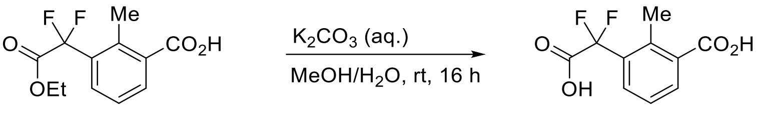

3bb 6k, 75%

Based on references[6]: K_2CO_3 (1 mL, 1N) was added at 23 °C to a solution of **3bb** (77.4 mg, 0.3 mmol) in MeOH (3 mL). The mixture was stirred overnight at 23 °C, poured into 5% HCl (3.0 mL) and successively extracted with EtOAc (10 mL × 3). The combined organic phase was washed with brine and dried over anhydrous Na_2SO_4. After removal of the solvents in vacuo, the residue was purified by column chromatography (CH_2Cl_2/MeOH = 5/1) to give **6k** (51.8 mg, 75% yield) as a colorless solid; **^{1}H NMR** (500 MHz, $CDCl_3$) δ 7.00 (d, J = 7.7 Hz, 1H), 6.89 (d, J = 7.9 Hz, 1H), 6.60 (t, J = 7.8 Hz, 1H), 1.62 (s, 3H); **^{13}C NMR** (125 MHz, $CDCl_3$) δ 174.1 (C_q), 170.0 (t, $J_{C\text{-}F}$ = 32.5 Hz, C_q), 140.7 (C_q), 139.6 (C_q), 137.9 (t, $J_{C\text{-}F}$ = 22.5 Hz, C_q), 136.9 (CH), 133.4 (t, $J_{C\text{-}F}$ = 8.8 Hz, CH), 131.3 (CH), 119.2 (t, $J_{C\text{-}F}$ = 250 Hz, C_q), 21.4 (CH_3); **^{19}F NMR** (471 MHz, $CDCl_3$) δ -94.9 (s); **ESI-HRMS** (m/z): $[M-H]^+$calcd for $C_{10}H_7F_2O_4$, 229.0318, found: 229.0315.

1.9. Single Crystal Structure and Data

X-ray structure of **3ba (CCDC 2245724)**

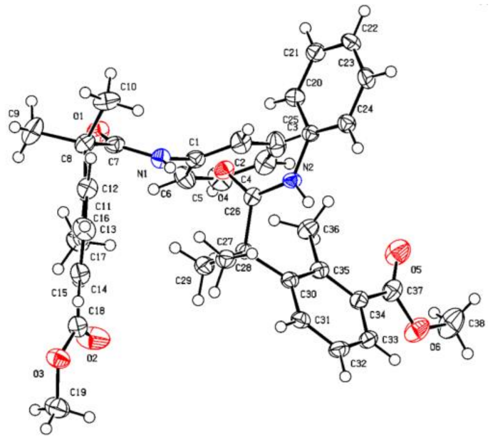

Supplementary Table 11. Crystal data and structure refinement for 3ba.

Identification code	**3ba**
Empirical formula	$C_{19}H_{21}NO_3$
Formula weight	311.37
Temperature/K	150.0
Crystal system	monoclinic
Space group	$P2_1/c$
a/Å	10.3226(5)
b/Å	14.5478(9)
c/Å	22.2334(12)
α/°	90
β/°	95.813(2)
γ/°	90
Volume/Å^3	3321.6(3)
Z	8
ρ_{calc}g/cm^3	1.245
μ/mm^{-1}	0.084
F(000)	1328.0
Crystal size/mm^3	0.12 × 0.06 × 0.05
Radiation	MoKα (λ = 0.71073)
2Θ range for data collection/°	3.966 to 50.81
Index ranges	-11 ≤ h ≤ 12, -15 ≤ k ≤ 17, -26 ≤ l ≤ 26
Reflections collected	22989
Independent reflections	6098 [R_{int} = 0.0679, R_{sigma} = 0.0679]
Data/restraints/parameters	6098/0/423
Goodness-of-fit on F^2	1.025
Final R indexes [I>=2σ (I)]	R_1 = 0.0504, wR_2 = 0.1047
Final R indexes [all data]	R_1 = 0.0926, wR_2 = 0.1288
Largest diff. peak/hole / e Å^{-3}	0.18/-0.21

Supplementary Table 12. Fractional Atomic Coordinates (×10^4) and Equivalent Isotropic Displacement Parameters (Å^2×10^3) for 3ba. U_{eq} is defined as 1/3 of of the trace of the orthogonalised U_{IJ} tensor.

Atom	*x*	*y*	*z*	U(eq)
O4	7390.6(13)	7446.2(10)	7168.6(6)	27.5(4)
O1	2404.0(13)	7291.8(11)	7270.5(7)	35.2(4)
O3	6313.5(15)	4704.9(11)	9665.3(7)	37.3(4)
N1	4515.3(16)	7056.3(12)	7114.9(7)	27.2(4)
N2	9450.6(16)	7472.3(12)	6885.0(8)	24.5(4)
O6	9886.4(17)	3450.4(12)	5555.3(8)	48.0(5)
O5	8807.7(17)	4749.9(13)	5297.7(8)	50.6(5)
O2	5279(2)	4353.2(12)	8767.2(8)	58.7(6)
C25	9376.8(19)	8227.1(14)	6483.5(9)	21.9(5)
C26	8480.3(19)	7101.1(14)	7177.8(9)	22.9(5)
C35	9145.3(19)	5263.1(15)	6591.9(9)	25.0(5)
C7	3569(2)	7364.9(15)	7441.8(10)	28.0(5)
C31	10669(2)	5073.4(15)	7485.4(10)	27.4(5)
C28	8865.7(19)	6234.8(14)	7549.8(9)	23.9(5)
C11	5036.2(19)	7316.1(16)	8455.0(9)	26.6(5)
C15	5790(2)	5937.6(15)	9001.6(9)	26.7(5)
C1	4289(2)	6620.9(15)	6537.4(9)	27.3(5)
C20	8202(2)	8607.9(15)	6239.0(9)	28.3(5)
C24	10543(2)	8582.8(15)	6321.2(9)	27.1(5)
C30	9588.4(19)	5524.8(14)	7192.2(9)	24.4(5)
C33	10905(2)	4141.7(15)	6623.9(10)	29.3(5)
C34	9837(2)	4576.7(15)	6312.3(10)	27.1(5)
C12	6076(2)	7789.6(17)	8769.0(10)	33.0(6)
C16	4880.1(19)	6373.2(16)	8575.8(9)	26.5(5)
C36	7936(2)	5690.8(16)	6263.0(10)	32.6(6)
C32	11319(2)	4387.9(15)	7208.7(10)	30.4(5)
C21	8212(2)	9331.6(16)	5836.8(9)	31.3(6)
C18	5737(2)	4930.4(17)	9114.4(10)	32.3(6)
C27	9701(2)	6594.4(16)	8114.1(9)	31.5(5)
C13	6939(2)	7356.5(17)	9195.3(10)	34.8(6)
C29	7638(2)	5789.4(16)	7760.5(11)	33.3(6)
C8	4056(2)	7872.0(16)	8031.7(10)	30.4(5)
C23	10528(2)	9315.8(16)	5923.3(10)	33.6(6)
C6	3350(2)	5948.3(16)	6421.4(10)	36.0(6)
C37	9440(2)	4293.3(17)	5676.9(11)	34.7(6)
C14	6803(2)	6437.0(17)	9311.2(10)	31.6(6)
C2	5063(2)	6866.0(17)	6091.9(10)	34.8(6)
C22	9361(2)	9698.2(16)	5679.6(10)	34.4(6)
C17	3760(2)	5828.0(16)	8262.9(10)	35.4(6)
C5	3192(2)	5530.4(18)	5862.1(11)	41.8(6)
C3	4911(2)	6433.2(18)	5532.1(11)	41.1(6)
C4	3970(2)	5766.5(18)	5417.8(11)	40.8(6)
C9	2894(2)	8120.4(19)	8380.9(11)	45.6(7)
C19	6343(3)	3729.2(17)	9803.7(11)	45.0(7)

C10	4641(2)	8779.2(16)	7811.7(11)	41.1(6)
C38	9573(3)	3135(2)	4938.2(12)	59.1(8)

Supplementary Table 13. Anisotropic Displacement Parameters ($Å^2 \times 10^3$) for 3ba. The Anisotropic displacement factor exponent takes the form: $-2\pi^2[h^2a^{*2}U_{11}+2hka^*b^*U_{12}+\cdots]$.

Atom	U_{11}	U_{22}	U_{33}	U_{23}	U_{13}	U_{12}
O4	21.3(8)	26.2(9)	34.9(8)	-0.6(7)	2.0(6)	0.0(6)
O1	19.3(8)	43.0(11)	41.9(9)	1.3(8)	-4.1(7)	3.5(7)
O3	46.0(10)	33.3(10)	31.1(9)	3.6(7)	-3.0(8)	-1.5(8)
N1	20.0(9)	33.3(12)	27.1(10)	-1.3(8)	-2.8(8)	0.2(8)
N2	18.4(9)	21.8(10)	33.4(10)	6.6(8)	2.5(8)	1.2(7)
O6	55.4(11)	37.4(11)	50.4(11)	-17.2(9)	1.6(9)	6.2(9)
O5	55.9(11)	54.9(13)	39.1(10)	-12.2(9)	-5.0(9)	12.9(10)
O2	88.8(15)	34.9(11)	46.5(11)	-4.7(9)	-21.8(10)	-5.6(10)
C25	24.3(11)	19.6(12)	21.5(11)	-1.7(9)	1.4(9)	-0.5(9)
C26	20.5(11)	23.4(13)	24.4(11)	-4.9(9)	0.4(9)	-2.0(9)
C35	21.4(11)	22.2(13)	31.3(12)	1.8(10)	2.0(9)	-2.2(9)
C7	22.8(11)	26.0(13)	34.5(12)	4.0(10)	0.1(10)	2.3(9)
C31	27.2(12)	25.2(13)	29.7(12)	4.8(10)	2.6(10)	-0.9(10)
C28	24.1(11)	20.8(12)	27.1(11)	2.5(9)	3.7(9)	-0.2(9)
C11	22.2(11)	32.4(14)	25.6(11)	-4.1(10)	4.5(9)	-0.4(10)
C15	26.5(11)	31.8(14)	22.6(11)	-0.5(10)	6.3(9)	-2.3(10)
C1	21.9(11)	30.3(14)	28.6(12)	3.5(10)	-2.7(9)	3.9(10)
C20	26.9(12)	29.1(14)	28.0(12)	0.8(10)	-1.1(10)	0.1(10)
C24	25.1(11)	29.3(14)	27.1(11)	1.6(10)	3.0(9)	-0.4(10)
C30	21.1(10)	21.4(12)	31.2(12)	3.8(10)	5.0(9)	-1.6(9)
C33	26.4(11)	22.1(13)	40.7(13)	1.2(10)	8.9(10)	1.8(10)
C34	26.8(11)	21.0(13)	34.2(12)	-1.7(10)	7.3(10)	-3.1(9)
C12	30.5(12)	32.7(14)	35.8(13)	-3.6(11)	3.2(10)	-3.2(10)
C16	22.7(11)	33.8(15)	23.5(11)	-2.6(10)	4.5(9)	-3.6(10)
C36	28.5(12)	31.6(14)	36.9(13)	-4.1(11)	-1.2(10)	3.6(10)
C32	25.1(11)	25.8(14)	40.1(13)	6.6(11)	2.7(10)	4.2(10)
C21	34.0(13)	35.7(15)	23.4(11)	1.3(10)	-1.1(10)	8.8(11)
C18	28.9(12)	38.9(16)	29.1(13)	0.1(11)	2.5(10)	-1.2(11)
C27	36.9(13)	28.9(14)	28.0(12)	0.4(10)	0.7(10)	3.6(10)
C13	31.0(13)	40.5(16)	31.2(12)	-2.2(11)	-5.4(10)	-9.8(11)
C29	31.5(12)	27.9(14)	41.9(14)	6.3(11)	11.2(11)	-0.3(10)
C8	25.7(11)	29.7(14)	35.2(13)	-3.5(10)	-0.2(10)	2.7(10)
C23	35.7(13)	34.7(15)	32.3(12)	3.2(11)	13.2(11)	-3.2(11)
C6	36.3(13)	38.5(16)	33.3(13)	2.1(11)	3.8(11)	-10.5(11)
C37	31.4(13)	32.2(15)	40.8(14)	-6.9(12)	5.6(11)	-0.5(11)
C14	27.0(12)	40.2(16)	27.1(12)	0.4(11)	0.5(10)	-2.3(11)
C2	23.4(11)	42.6(16)	37.4(13)	-0.4(12)	-1.0(10)	-4.2(11)
C22	49.5(15)	29.8(14)	24.9(12)	8.1(10)	8.2(11)	5.8(11)

C17	33.2(13)	36.4(15)	35.7(13)	4.5(11)	-1.7(11)	-8.9(11)
C5	43.1(15)	42.6(17)	38.6(14)	-1.6(12)	-1.4(12)	-11.4(12)
C3	29.7(13)	59.1(18)	34.7(13)	-0.6(13)	4.9(11)	-0.1(12)
C4	35.8(14)	50.4(18)	34.6(13)	-9.7(12)	-3.3(11)	3.2(12)
C9	32.2(13)	54.9(18)	49.9(16)	-17.3(13)	4.6(12)	8.9(12)
C19	56.6(17)	35.9(17)	42.3(15)	7.1(12)	4.4(12)	-1.2(13)
C10	44.3(14)	25.7(14)	50.9(15)	0.1(12)	-6.7(12)	3.6(11)
C38	63.8(19)	60(2)	52.2(17)	-31.9(15)	-1.3(15)	6.5(15)

Supplementary Table 14. Bond Lengths for 3ba.

Atom	Atom	Length/Å	Atom	Atom	Length/Å
O4	C26	1.230(2)	C11	C12	1.401(3)
O1	C7	1.229(2)	C11	C16	1.410(3)
O3	C18	1.347(3)	C11	C8	1.540(3)
O3	C19	1.452(3)	C15	C16	1.414(3)
N1	C7	1.352(3)	C15	C18	1.489(3)
N1	C1	1.430(3)	C15	C14	1.396(3)
N2	C25	1.412(3)	C1	C6	1.383(3)
N2	C26	1.360(3)	C1	C2	1.381(3)
O6	C37	1.347(3)	C20	C21	1.382(3)
O6	C38	1.452(3)	C24	C23	1.385(3)
O5	C37	1.211(3)	C33	C34	1.393(3)
O2	C18	1.204(3)	C33	C32	1.375(3)
C25	C20	1.392(3)	C34	C37	1.489(3)
C25	C24	1.391(3)	C12	C13	1.385(3)
C26	C28	1.538(3)	C16	C17	1.512(3)
C35	C30	1.418(3)	C21	C22	1.377(3)
C35	C34	1.408(3)	C13	C14	1.372(3)
C35	C36	1.515(3)	C8	C9	1.536(3)
C7	C8	1.544(3)	C8	C10	1.551(3)
C31	C30	1.398(3)	C23	C22	1.387(3)
C31	C32	1.381(3)	C6	C5	1.379(3)
C28	C30	1.541(3)	C2	C3	1.390(3)
C28	C27	1.540(3)	C5	C4	1.378(3)
C28	C29	1.538(3)	C3	C4	1.378(3)

Supplementary Table 15. Bond Angles for 3ba.

Atom	Atom	Atom	Angle/°	Atom	Atom	Atom	Angle/°
C18	O3	C19	115.38(18)	C35	C30	C28	122.58(17)
C7	N1	C1	124.67(17)	C31	C30	C35	118.6(2)
C26	N2	C25	128.02(17)	C31	C30	C28	118.65(18)
C37	O6	C38	115.4(2)	C32	C33	C34	120.5(2)
C20	C25	N2	123.04(19)	C35	C34	C37	120.79(19)
C24	C25	N2	117.41(18)	C33	C34	C35	120.9(2)
C24	C25	C20	119.5(2)	C33	C34	C37	118.3(2)

O4	C26	N2	123.12(19)	C13	C12	C11	121.7(2)
O4	C26	C28	122.00(18)	C11	C16	C15	118.85(18)
N2	C26	C28	114.82(17)	C11	C16	C17	121.29(19)
C30	C35	C36	121.02(19)	C15	C16	C17	119.9(2)
C34	C35	C30	118.43(18)	C33	C32	C31	119.4(2)
C34	C35	C36	120.53(19)	C22	C21	C20	121.4(2)
O1	C7	N1	122.8(2)	O3	C18	C15	111.87(19)
O1	C7	C8	121.9(2)	O2	C18	O3	121.4(2)
N1	C7	C8	115.13(17)	O2	C18	C15	126.7(2)
C32	C31	C30	122.2(2)	C14	C13	C12	120.0(2)
C26	C28	C30	112.60(17)	C7	C8	C10	104.03(18)
C26	C28	C27	104.64(17)	C11	C8	C7	113.92(18)
C27	C28	C30	112.80(17)	C11	C8	C10	112.77(18)
C29	C28	C26	109.45(17)	C9	C8	C7	109.76(18)
C29	C28	C30	109.36(17)	C9	C8	C11	108.24(18)
C29	C28	C27	107.79(17)	C9	C8	C10	107.9(2)
C12	C11	C16	118.69(19)	C24	C23	C22	120.8(2)
C12	C11	C8	118.0(2)	C5	C6	C1	119.8(2)
C16	C11	C8	123.14(18)	O6	C37	C34	112.1(2)
C16	C15	C18	121.39(19)	O5	C37	O6	122.1(2)
C14	C15	C16	120.7(2)	O5	C37	C34	125.9(2)
C14	C15	C18	117.87(19)	C13	C14	C15	120.0(2)
C6	C1	N1	121.9(2)	C1	C2	C3	120.3(2)
C2	C1	N1	118.43(19)	C21	C22	C23	118.8(2)
C2	C1	C6	119.6(2)	C4	C5	C6	120.8(2)
C21	C20	C25	119.5(2)	C4	C3	C2	119.8(2)
C23	C24	C25	119.9(2)	C5	C4	C3	119.6(2)

Supplementary Table 16. Hydrogen Atom Coordinates (Å×10^4) and Isotropic Displacement Parameters (Å^2×10^3) for 3ba.

Atom	*x*	*y*	*z*	U(eq)
H1	5328.55	7128.68	7268.56	33
H2	10219.14	7209.62	6954.28	29
H31	10966.45	5243.79	7888.36	33
H20	7400.31	8371.91	6347.88	34
H24	11348.64	8323.3	6483.1	33
H33	11349.2	3671.45	6430.91	35
H12	6191.08	8423.79	8687.68	40
H36A	7400.45	5958.5	6557.88	49
H36B	7435	5217.36	6027.84	49
H36C	8190.09	6173.2	5990.59	49
H32	12046.69	4089.3	7420.6	36
H21	7407.84	9581.75	5664.9	38
H27A	10535.67	6818.88	7998.04	47
H27B	9857.22	6095.38	8408.61	47
H27C	9241.99	7097.67	8294.64	47

H13	7626.63	7696.24	9407.51	42
H29A	7189.39	6235.28	7996.62	50
H29B	7887.47	5252.14	8011.97	50
H29C	7056.45	5597.02	7407.35	50
H23	11326.84	9559.52	5815.89	40
H6	2816.41	5774.61	6726.22	43
H14	7397.9	6139.97	9602.31	38
H2A	5702.74	7332.58	6168.56	42
H22	9353.39	10204.12	5409.13	41
H17A	3014.85	6236.23	8162.09	53
H17B	3514.11	5338.82	8532.16	53
H17C	4026.8	5555.52	7891.5	53
H5	2538.08	5074.41	5782.02	50
H3	5454.6	6596.48	5228.8	49
H4	3857.7	5471.3	5034.95	49
H9A	2251.32	8466.08	8116.32	68
H9B	3196.58	8497.43	8732.64	68
H9C	2494.17	7556.22	8516.42	68
H19A	6718.49	3393.37	9481.05	67
H19B	5454.18	3510.37	9835.35	67
H19C	6874.79	3625.38	10188.28	67
H10A	5474.96	8649.76	7654.25	62
H10B	4778.81	9210.61	8150.69	62
H10C	4038.48	9050.46	7491.23	62
H38A	9932.19	3564.89	4659.34	89
H38B	8624.83	3102.71	4846.84	89
H38C	9949.13	2523.5	4891.9	89

Experimental

Single crystals of $C_{19}H_{21}NO_3$ [**3ba**] were []. A suitable crystal was selected and [] on a 'D8 VENTURE ' diffractometer. The crystal was kept at 150.0 K during data collection. Using Olex2 [1], the structure was solved with the ShelXT [2] structure solution program using Intrinsic Phasing and refined with the ShelXL [3] refinement package using Least Squares minimisation.

1. Dolomanov, O.V., Bourhis, L.J., Gildea, R.J, Howard, J.A.K. & Puschmann, H. (2009), J. Appl. Cryst. 42, 339-341.
2. Sheldrick, G.M. (2015). Acta Cryst. A71, 3-8.
3. Sheldrick, G.M. (2015). Acta Cryst. C71, 3-8.

Crystal structure determination of [3ba]

Crystal Data for $C_{19}H_{21}NO_3$ (M =311.37 g/mol): monoclinic, space group P2$_1$/c (no. 14), a = 10.3226(5) Å, b = 14.5478(9) Å, c = 22.2334(12) Å, β = 95.813(2)°, V = 3321.6(3) Å^3, Z = 8, T = 150.0 K, μ(MoKα) = 0.084 mm^{-1}, $Dcalc$ = 1.245 g/cm^3, 22989 reflections measured (3.966° ⩽ 2Θ ⩽ 50.81°), 6098 unique (R_{int} = 0.0679, R_{sigma} = 0.0679) which were used in all calculations. The final R_1 was 0.0504 (I > 2σ(I)) and wR_2 was 0.1288 (all data).

X-ray structure of **3kb (CCDC 2284237)**

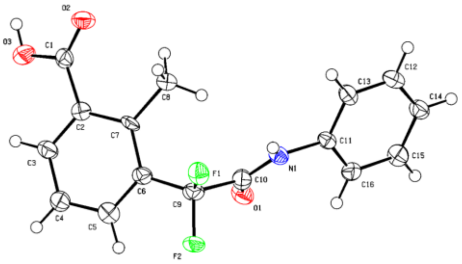

Supplementary Table 17. Crystal data and structure refinement for 3kb.

Identification code	**3kb**
Empirical formula	$C_{16}H_{13}F_2NO_3$
Formula weight	305.27
Temperature/K	100
Crystal system	monoclinic
Space group	$P2_1/n$
a/Å	5.2730(14)
b/Å	5.5058(14)
c/Å	45.503(12)
α/°	90
β/°	90.925(8)
γ/°	90
Volume/Å^3	1320.9(6)
Z	4
ρ_{calc}g/cm^3	1.535
μ/mm^{-1}	0.125
F(000)	632.0
Crystal size/mm^3	0.06 × 0.04 × 0.02
Radiation	MoKα (λ = 0.71073)
2Θ range for data collection/°	5.372 to 50.052
Index ranges	-6 ≤ h ≤ 6, 0 ≤ k ≤ 6, 0 ≤ l ≤ 54
Reflections collected	2255
Independent reflections	2255 [R_{int} = ?, R_{sigma} = 0.1212]
Data/restraints/parameters	2255/0/202
Goodness-of-fit on F^2	1.105
Final R indexes [I>=2σ (I)]	R_1 = 0.1130, wR_2 = 0.2587
Final R indexes [all data]	R_1 = 0.1633, wR_2 = 0.2856
Largest diff. peak/hole / e Å^{-3}	0.45/-0.54

Supplementary Table 18. Fractional Atomic Coordinates (×10^4) and Equivalent Isotropic Displacement Parameters (Å^2×10^3) for 3kb. U_{eq} is defined as 1/3 of the trace of the ortho gonalised U_{IJ} tensor.

**Atom**	***x***	***y***	***z***	**U(eq)**
F1	7414(6)	2714(5)	3751.4(6)	32.2(8)
F2	3873(6)	1008(5)	3607.5(6)	31.0(8)
O2	6275(7)	8674(7)	4704.9(8)	33.9(10)

O1	2025(7)	5676(7)	3455.2(8)	32.9(10)
O3	2645(8)	7682(7)	4932.7(8)	38.0(11)
N1	6297(9)	6016(8)	3362.4(9)	26.7(11)
C10	4258(12)	5133(10)	3497.8(11)	30.8(15)
C7	4666(10)	5340(9)	4218.7(11)	22.9(13)
C11	6202(10)	7881(9)	3137.5(10)	21.8(12)
C8	6693(10)	7105(10)	4123.7(11)	28.1(14)
C2	3565(10)	5522(9)	4505.0(10)	23.2(13)
C9	4852(11)	3123(10)	3729.8(11)	30.1(15)
C1	4367(11)	7398(10)	4714.9(11)	27.0(14)
C12	8100(10)	11368(10)	2923.9(11)	29.1(14)
C3	1748(11)	3853(10)	4591.5(12)	32.0(15)
C15	4192(11)	9799(10)	2719.2(12)	29.7(14)
C13	8166(11)	9551(10)	3131.4(11)	28.2(14)
C6	3752(10)	3455(9)	4035.7(11)	25.4(13)
C4	935(12)	1966(10)	4408.1(11)	32.2(15)
C14	6154(10)	11491(10)	2713.8(11)	29.2(14)
C16	4205(12)	7954(10)	2931.4(11)	31.1(15)
C5	1952(10)	1841(10)	4125.5(11)	27.8(14)

Supplementary Table 19. Anisotropic Displacement Parameters (Å^2×10^3) for 3kb. The Anisotropic displacement factor exponent takes the form: $-2\pi^2[h^2a^{*2}U_{11}+2hka^*b^*U_{12}+\cdots]$.

Atom	U_{11}	U_{22}	U_{33}	U_{23}	U_{13}	U_{12}
F1	37.9(17)	24.9(15)	34.0(14)	2.6(13)	4.1(14)	6.6(15)
F2	39.8(17)	19.0(15)	34.4(14)	-7.2(13)	3.7(14)	-6.3(15)
O2	36(2)	27(2)	39.2(19)	-9.1(18)	4.7(18)	-6.6(19)
O1	28.3(19)	31(2)	39.5(19)	6.9(17)	-4.5(18)	-4.6(19)
O3	46(2)	35(2)	33.0(18)	-10.6(17)	6.7(19)	-7(2)
N1	30(2)	19(2)	31(2)	2.8(19)	0(2)	0(2)
C10	46(3)	19(3)	27(2)	-1(2)	6(3)	4(3)
C7	26(3)	12(2)	30(2)	5(2)	-2(2)	-6(2)
C11	20(2)	19(3)	27(2)	0(2)	3(2)	0(2)
C8	31(3)	25(3)	29(2)	-2(2)	-1(2)	-6(3)
C2	22(3)	20(2)	27(2)	0(2)	-2(2)	5(2)
C9	39(3)	19(3)	32(3)	-1(2)	-5(3)	-1(3)
C1	36(3)	20(3)	25(2)	4(2)	1(2)	3(3)
C12	26(3)	22(3)	39(3)	-3(2)	6(2)	-4(3)
C3	39(3)	23(3)	34(3)	-2(2)	7(3)	-7(3)
C15	35(3)	22(3)	33(3)	4(2)	-2(3)	9(3)
C13	32(3)	22(3)	31(2)	2(2)	9(2)	0(3)
C6	31(3)	16(3)	29(2)	1(2)	-4(2)	2(2)
C4	47(3)	19(3)	30(2)	2(2)	1(3)	3(3)
C14	34(3)	22(3)	32(2)	1(2)	4(2)	-2(3)
C16	45(3)	23(3)	26(2)	-2(2)	-1(3)	-4(3)
C5	24(3)	23(3)	36(3)	0(2)	4(2)	8(2)

Supplementary Table 20. Bond Lengths for 3kb.

Atom	Atom	Length/Å	Atom	Atom	Length/Å
F1	C9	1.371(7)	C11	C16	1.399(7)
F2	C9	1.387(6)	C2	C1	1.464(7)
O2	C1	1.228(7)	C2	C3	1.389(8)
O1	C10	1.227(7)	C9	C6	1.528(7)
O3	C1	1.364(6)	C12	C13	1.375(7)
N1	C10	1.339(7)	C12	C14	1.393(8)
N1	C11	1.450(6)	C3	C4	1.396(8)
C10	C9	1.558(8)	C15	C14	1.393(8)
C7	C8	1.513(7)	C15	C16	1.401(8)
C7	C2	1.438(7)	C6	C5	1.368(8)
C7	C6	1.410(7)	C4	C5	1.403(7)
C11	C13	1.386(7)			

Supplementary Table 21. Bond Angles for 3kb.

Atom	Atom	Atom	Angle/˚	Atom	Atom	Atom	Angle/˚
C10	N1	C11	124.3(5)	F2	C9	C6	108.8(4)
O1	C10	N1	127.9(5)	C6	C9	C10	117.2(5)
O1	C10	C9	117.5(5)	O2	C1	O3	121.1(5)
N1	C10	C9	114.5(5)	O2	C1	C2	127.4(5)
C2	C7	C8	120.7(4)	O3	C1	C2	111.5(5)
C6	C7	C8	122.7(4)	C13	C12	C14	121.1(5)
C6	C7	C2	116.6(5)	C2	C3	C4	122.0(5)
C13	C11	N1	117.8(4)	C14	C15	C16	120.1(5)
C13	C11	C16	121.5(5)	C12	C13	C11	119.1(5)
C16	C11	N1	120.7(5)	C7	C6	C9	119.7(5)
C7	C2	C1	121.5(5)	C5	C6	C7	122.3(5)
C3	C2	C7	120.1(5)	C5	C6	C9	118.0(5)
C3	C2	C1	118.4(4)	C3	C4	C5	117.8(5)
F1	C9	F2	104.5(4)	C15	C14	C12	119.6(5)
F1	C9	C10	110.6(5)	C11	C16	C15	118.6(5)
F1	C9	C6	110.0(4)	C6	C5	C4	121.3(5)
F2	C9	C10	104.8(4)				

Supplementary Table 22. Torsion Angles for 3kb.

A	B	C	D	Angle/˚	A	B	C	D	Angle/˚
F1	C9	C6	C7	59.2(6)	C11	N1	C10	C9	179.5(4)
F1	C9	C6	C5	-118.7(5)	C8	C7	C2	C1	-0.4(7)
F2	C9	C6	C7	173.2(4)	C8	C7	C2	C3	-178.5(5)
F2	C9	C6	C5	-4.8(7)	C8	C7	C6	C9	0.9(8)
O1	C10	C9	F1	179.7(5)	C8	C7	C6	C5	178.8(5)
O1	C10	C9	F2	67.6(6)	C2	C7	C6	C9	-179.2(5)
O1	C10	C9	C6	-53.1(7)	C2	C7	C6	C5	-1.3(8)
N1	C10	C9	F1	1.4(6)	C2	C3	C4	C5	-2.2(8)
N1	C10	C9	F2	-110.7(5)	C9	C6	C5	C4	177.2(5)

N1	C10	C9	C6	128.7(5)	C1	C2	C3	C4	-178.1(5)
N1	C11	C13	C12	-178.8(5)	C3	C2	C1	O2	163.2(5)
N1	C11	C16	C15	179.7(5)	C3	C2	C1	O3	-19.0(7)
C10	N1	C11	C13	144.3(5)	C3	C4	C5	C6	2.5(8)
C10	N1	C11	C16	-36.7(7)	C13	C11	C16	C15	-1.3(8)
C10	C9	C6	C7	-68.3(7)	C13	C12	C14	C15	2.2(8)
C10	C9	C6	C5	113.7(6)	C6	C7	C2	C1	179.7(5)
C7	C2	C1	O2	-15.0(8)	C6	C7	C2	C3	1.6(7)
C7	C2	C1	O3	162.9(5)	C14	C12	C13	C11	-2.7(8)
C7	C2	C3	C4	0.1(8)	C14	C15	C16	C11	0.8(8)
C7	C6	C5	C4	-0.7(8)	C16	C11	C13	C12	2.2(8)
C11	N1	C10	O1	1.5(9)	C16	C15	C14	C12	-1.3(8)

Supplementary Table 23. Hydrogen Atom Coordinates (Å×10^4) and Isotropic Displacement Parameters (Å^2×10^3) for 3kb.

**Atom**	***x***	***y***	***z***	**U(eq)**
H3	3105.2	8824.99	5043.6	57
H1	7791.4	5425.39	3413.17	32
H8A	6300.69	8729.53	4198.32	42
H8B	6747.67	7148.55	3908.61	42
H8C	8343.83	6579.97	4202.73	42
H12	9403.73	12558.33	2923.96	35
H3A	1037.21	4000.87	4781.13	38
H15	2843.01	9895.12	2578.81	36
H13	9539.04	9442.28	3268.53	34
H4	-266.55	804.86	4472.53	39
H14	6164.17	12723.18	2567.69	35
H16	2887.14	6779.03	2935.26	37
H5	1378.89	611.11	3993.96	33

Experimental

Single crystals of $C_{16}H_{13}F_2NO_3$ **3kb** were **[]**. A suitable crystal was selected and **[]** on a **D8 VENTURE** diffractometer. The crystal was kept at 100 K during data collection. Using Olex2 [1], the structure was solved with the SHELXT [2] structure solution program using Intrinsic Phasing and refined with the SHELXL [3] refinement package using Least Squares minimisation.

1. Dolomanov, O.V., Bourhis, L.J., Gildea, R.J, Howard, J.A.K. & Puschmann, H. (2009), J. Appl. Cryst. 42, 339-341.
2. Sheldrick, G.M. (2015). Acta Cryst. A71, 3-8.
3. Sheldrick, G.M. (2015). Acta Cryst. C71, 3-8.

Crystal structure determination of 3kb

Crystal Data for $C_{16}H_{13}F_2NO_3$ (M =305.27 g/mol): monoclinic, space group P2$_1$/n (no. 14), a = 5.2730(14) Å, b = 5.5058(14) Å, c = 45.503(12) Å, β = 90.925(8), V = 1320.9(6) Å^3, Z = 4, T = 100 K, μ (MoKα) = 0.125 mm^{-1}, $Dcalc$ = 1.535 g/cm^3, 2255 reflections measured (5.372° ≤ 2Θ ≤ 50.052), 2255 unique (R_{int} = ?, R_{sigma} = 0.1212) which were used in all calculations. The final R_1 was 0.1130 (I > 2σ(I)) and wR_2 was 0.2856 (all data).

X-ray structure of **4aa (CCDC 2245725)**

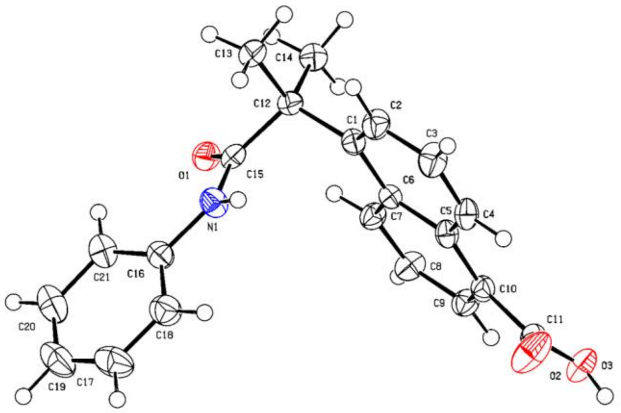

Supplementary Table 24. Crystal data and structure refinement for 4aa.

Identification code	**4aa**
Empirical formula	$C_{21}H_{19}NO_3$
Formula weight	333.37
Temperature/K	170
Crystal system	monoclinic
Space group	$P2_1/n$
a/Å	10.5739(10)
b/Å	10.8913(9)
c/Å	14.6081(12)
α/°	90
β/°	100.498(3)
γ/°	90
Volume/Å^3	1654.2(2)
Z	4
ρ_{calc}g/cm^3	1.339
μ/mm^{-1}	0.090
F(000)	704.0
Crystal size/mm^3	0.09 × 0.05 × 0.04
Radiation	MoKα (λ = 0.71073)
2Θ range for data collection/°	4.398 to 52.776
Index ranges	-13 ≤ h ≤ 13, -13 ≤ k ≤ 13, -18 ≤ l ≤ 17
Reflections collected	11762
Independent reflections	3374 [R_{int} = 0.0766, R_{sigma} = 0.0783]
Data/restraints/parameters	3374/0/229
Goodness-of-fit on F^2	1.085
Final R indexes [I>=2σ (I)]	R_1 = 0.0578, wR_2 = 0.1088
Final R indexes [all data]	R_1 = 0.1110, wR_2 = 0.1361
Largest diff. peak/hole / e Å^{-3}	0.23/-0.24

Supplementary Table 25. Fractional Atomic Coordinates (×10^4) and Equivalent Isotropic Displacement Parameters (Å^2×10^3) for 4aa. U_{eq} is defined as 1/3 of of the trace of the ortho gonalised U_{IJ} tensor.

Atom	*x*	*y*	*z*	U(eq)
O1	8477.3(15)	3969.3(16)	7637.0(12)	29.4(4)
O3	958.2(16)	3278.5(16)	8315.9(14)	35.8(5)
N1	7254.5(19)	5606(2)	7060.2(14)	29.3(5)
O2	1218.2(17)	5205.7(18)	7910.8(16)	51.3(6)
C10	3088(2)	3968(2)	8328.5(16)	24.1(6)
C6	5349(2)	4648(2)	8557.4(16)	22.5(5)
C16	7252(2)	5499(2)	6091.4(17)	27.6(6)
C9	3461(2)	2800(2)	8139.3(17)	28.0(6)
C11	1683(2)	4232(2)	8166.7(17)	27.5(6)
C7	5659(2)	3439(2)	8313.0(17)	26.0(6)
C15	7854(2)	4892(2)	7765.8(17)	24.6(6)
C8	4757(2)	2537(2)	8131.8(17)	29.6(6)
C12	7726(2)	5350(2)	8741.5(16)	25.0(6)
C4	3698(2)	6116(2)	8832.4(17)	27.6(6)
C5	4027(2)	4923(2)	8567.0(16)	24.1(6)
C2	5924(2)	6714(2)	9057.5(18)	31.6(6)
C1	6306(2)	5591(2)	8788.1(16)	24.3(6)
C13	8553(2)	6533(2)	8891.4(19)	32.1(6)
C14	8333(2)	4433(3)	9488.0(17)	31.6(6)
C3	4630(2)	6971(2)	9086.3(19)	34.8(7)
C21	8219(3)	4903(2)	5745.3(18)	34.4(7)
C18	6255(3)	6057(3)	5489.7(19)	37.9(7)
C20	8155(3)	4838(3)	4788.8(19)	43.2(8)
C17	6218(3)	6002(3)	4541(2)	47.8(8)
C19	7155(3)	5378(3)	4185(2)	48.6(8)

Supplementary Table 26. Anisotropic Displacement Parameters (Å^2×10^3) for 4aa. The Anisotropic displacement factor exponent takes the form: $-2\pi^2[h^2a^{*2}U_{11}+2hka^*b^*U_{12}+\cdots]$.

Atom	U_{11}	U_{22}	U_{33}	U_{23}	U_{13}	U_{12}
O1	25.4(9)	30.9(10)	32.1(10)	-1.2(8)	5.8(8)	4.3(8)
O3	21.4(9)	33.9(11)	52.0(12)	8.0(9)	6.8(9)	-1.1(8)
N1	30.8(12)	30.3(13)	27.5(12)	0.4(10)	7.3(9)	8.8(10)
O2	26.9(10)	37.1(13)	86.9(17)	25.2(12)	2.3(10)	4.9(9)
C10	23.8(13)	26.5(14)	22.4(13)	3.8(11)	5.4(10)	0.8(11)
C6	23.0(13)	26.2(14)	18.6(12)	1.3(11)	4.3(10)	-0.9(11)
C16	33.3(14)	24.6(14)	25.4(13)	-0.5(11)	6.6(11)	-3.5(12)
C9	27.0(13)	26.6(15)	30.2(15)	0.2(12)	4.7(11)	-4.5(11)
C11	26.3(14)	29.3(15)	27.2(14)	4.1(12)	5.5(11)	-2.3(12)

C7	22.5(13)	25.7(14)	30.1(14)	-2.9(11)	5.8(11)	0.8(11)
C15	17.1(12)	27.3(14)	28.9(14)	0.0(11)	3.3(10)	-2.6(11)
C8	27.0(14)	22.0(14)	39.4(16)	-4.4(12)	4.9(11)	1.0(11)
C12	22.7(13)	27.6(14)	24.6(13)	-3.4(11)	3.7(10)	-3.2(11)
C4	27.5(13)	26.0(14)	32.0(14)	2.2(12)	12.8(11)	4.2(11)
C5	27.2(13)	22.9(14)	23.8(13)	4.7(11)	8.5(10)	0.6(11)
C2	33.6(15)	26.2(15)	36.8(16)	-4.8(12)	11.2(12)	-7.0(12)
C1	26.2(13)	24.9(14)	22.7(13)	0.2(11)	7.2(10)	-1.2(11)
C13	26.3(14)	36.8(16)	33.3(15)	-3.2(13)	5.7(11)	-7.6(12)
C14	28.2(14)	37.5(16)	28.6(14)	1.5(12)	4.2(11)	-0.7(12)
C3	37.2(15)	24.7(15)	46.0(18)	-6.9(13)	17.3(13)	0.6(12)
C21	41.2(16)	31.1(16)	33.2(15)	1.7(12)	12.5(13)	2.2(13)
C18	38.7(16)	40.5(18)	33.7(16)	1.3(14)	4.9(12)	1.6(14)
C20	62(2)	36.7(17)	35.1(17)	-0.9(14)	21.2(15)	-3.2(15)
C17	59(2)	49(2)	30.9(16)	9.0(15)	-3.6(14)	0.9(16)
C19	74(2)	46(2)	26.5(16)	2.6(14)	11.7(16)	-9.5(18)

Supplementary Table 27. Bond Lengths for 4aa.

Atom	Atom	Length/Å	Atom	Atom	Length/Å
O1	C15	1.235(3)	C7	C8	1.362(3)
O3	C11	1.332(3)	C15	C12	1.539(3)
N1	C16	1.420(3)	C12	C1	1.538(3)
N1	C15	1.353(3)	C12	C13	1.550(3)
O2	C11	1.200(3)	C12	C14	1.530(3)
C10	C9	1.375(3)	C4	C5	1.417(3)
C10	C11	1.489(3)	C4	C3	1.357(3)
C10	C5	1.436(3)	C2	C1	1.368(3)
C6	C7	1.419(3)	C2	C3	1.404(3)
C6	C5	1.432(3)	C21	C20	1.388(4)
C6	C1	1.438(3)	C18	C17	1.381(4)
C16	C21	1.382(3)	C20	C19	1.380(4)
C16	C18	1.384(4)	C17	C19	1.378(4)
C9	C8	1.402(3)			

Supplementary Table 28. Bond Angles for 4aa.

Atom	Atom	Atom	Angle/°	Atom	Atom	Atom	Angle/°
C15	N1	C16	128.9(2)	C1	C12	C15	110.34(19)
C9	C10	C11	117.4(2)	C1	C12	C13	112.4(2)
C9	C10	C5	120.5(2)	C14	C12	C15	110.6(2)
C5	C10	C11	121.6(2)	C14	C12	C1	111.45(19)
C7	C6	C5	117.9(2)	C14	C12	C13	106.8(2)

C7	C6	C1	122.5(2)	C3	C4	C5	120.1(2)
C5	C6	C1	119.6(2)	C6	C5	C10	118.6(2)
C21	C16	N1	122.4(2)	C4	C5	C10	122.5(2)
C21	C16	C18	120.1(2)	C4	C5	C6	118.9(2)
C18	C16	N1	117.4(2)	C1	C2	C3	121.8(2)
C10	C9	C8	120.5(2)	C6	C1	C12	121.2(2)
O3	C11	C10	114.3(2)	C2	C1	C6	118.3(2)
O2	C11	O3	121.6(2)	C2	C1	C12	120.5(2)
O2	C11	C10	124.1(2)	C4	C3	C2	121.2(2)
C8	C7	C6	122.2(2)	C16	C21	C20	119.3(3)
O1	C15	N1	122.7(2)	C17	C18	C16	119.9(3)
O1	C15	C12	123.0(2)	C19	C20	C21	120.8(3)
N1	C15	C12	114.3(2)	C19	C17	C18	120.6(3)
C7	C8	C9	120.2(2)	C17	C19	C20	119.3(3)
C15	C12	C13	104.94(19)				

Supplementary Table 29. Hydrogen Atom Coordinates (Å×10^4) and Isotropic Displacement Parameters (Å^2×10^3) for 4aa.

**Atom**	***x***	***y***	***z***	**U(eq)**
H3	178.18	3459.08	8147.49	54
H1	6804	6220.67	7224.5	35
H9	2835.95	2166.35	8012.75	34
H7	6525.62	3252.38	8274.33	31
H8	5005.86	1727.11	8000.04	36
H4	2824.16	6317.33	8832.67	33
H2	6550.61	7337.11	9229.88	38
H13A	9422.19	6354.55	8782.88	48
H13B	8599.59	6825.28	9531.25	48
H13C	8160.68	7168.09	8454.92	48
H14A	7827.19	3674.06	9425.31	47
H14B	8344.58	4787.52	10106.23	47
H14C	9214.81	4251.93	9410.4	47
H3A	4402.98	7757.22	9286.97	42
H21	8919.52	4542.39	6157.22	41
H18	5598.1	6476.22	5728.85	45
H20	8808.77	4415.29	4547.01	52
H17	5541.37	6397.91	4129.3	57
H19	7111.81	5320.36	3531.14	58

II. ^{1}H NMR, ^{13}C NMR and ^{19}F NMR Spectrum of All Products

2-Bromo-2-methyl-*N*-(4-(trifluoromethyl)phenyl)propenamide, **1c**, ^{1}H NMR (500 MHz, $CDCl_3$), ^{13}C NMR (125 MHz, $CDCl_3$) and ^{19}F NMR (471 MHz, $CDCl_3$)

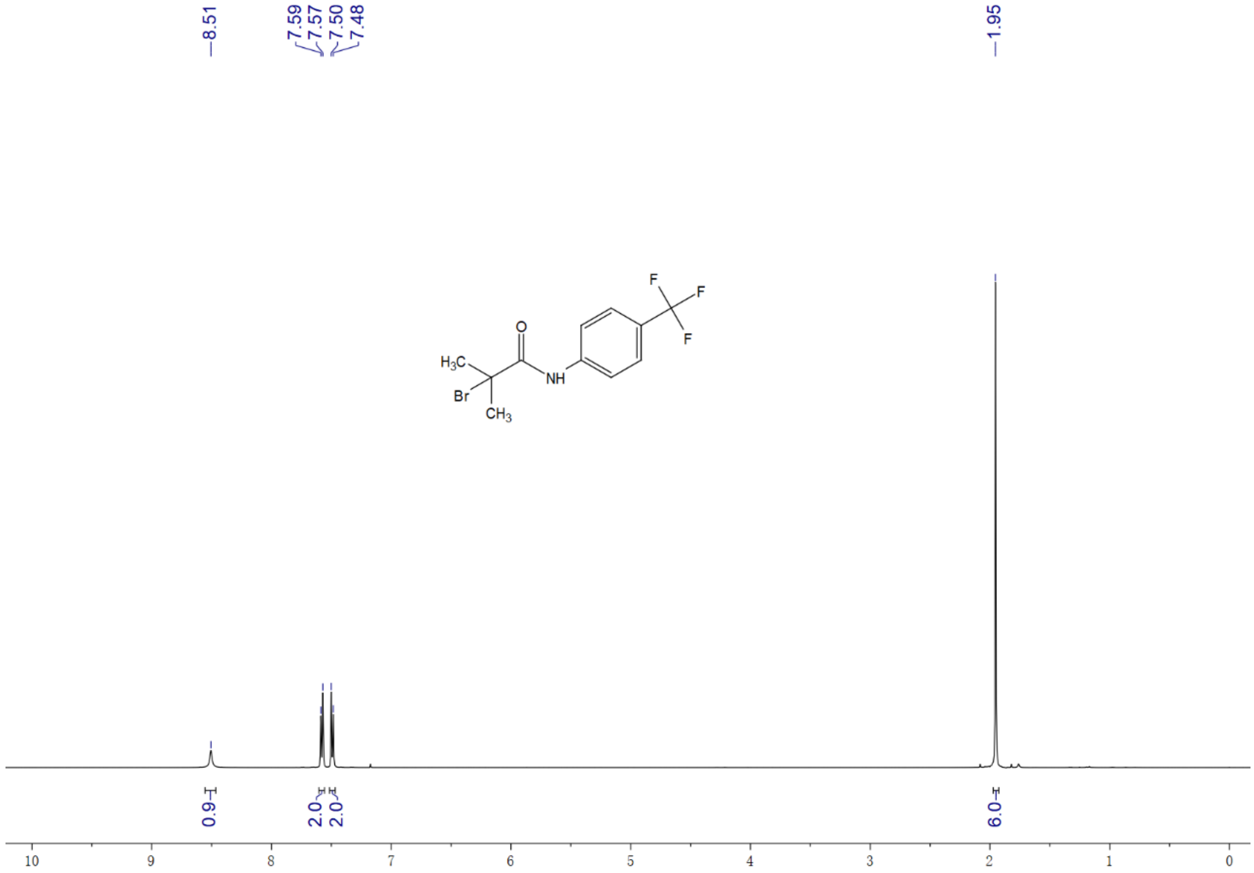

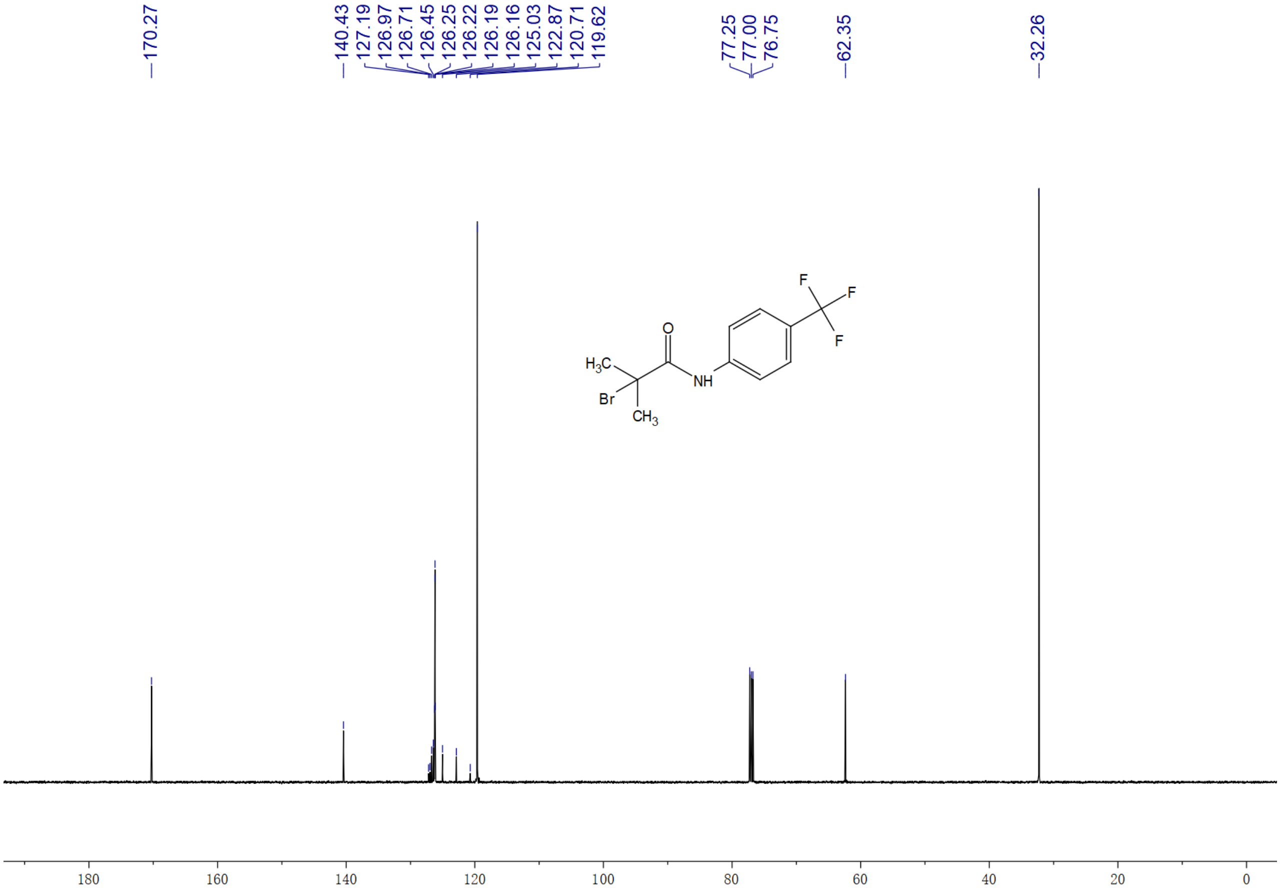

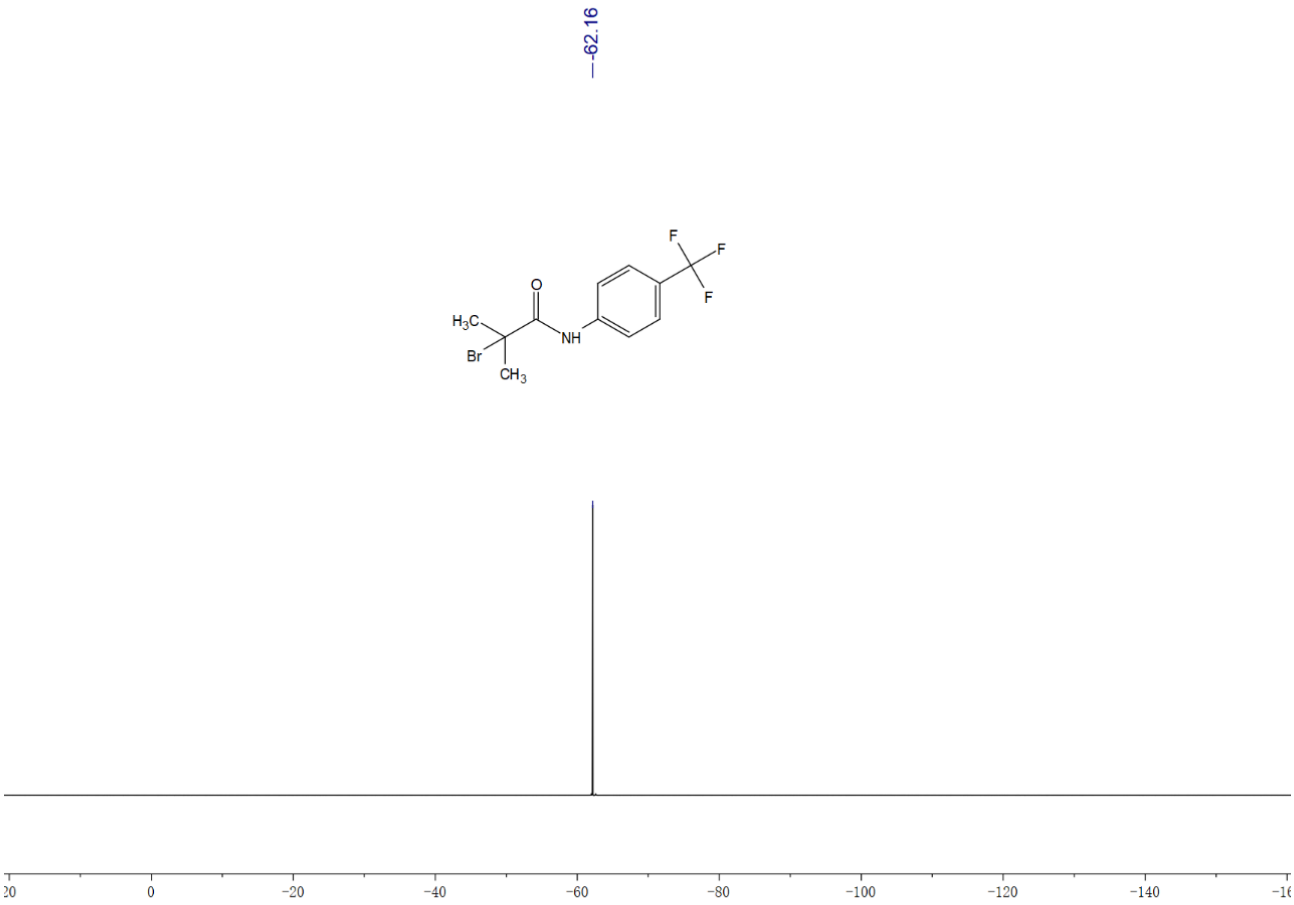

-62.16
H3C
Br
CH3
O
NH
F
F
F
20
0
-20
-40
-60
-80
-100
-120
-140

N-([1,1'-Biphenyl]-4-yl)-2-bromo-2-methylpropanamide, **1d**, ^{1}H NMR (500 MHz, $CDCl_3$) and ^{13}C NMR (125 MHz, $CDCl_3$)

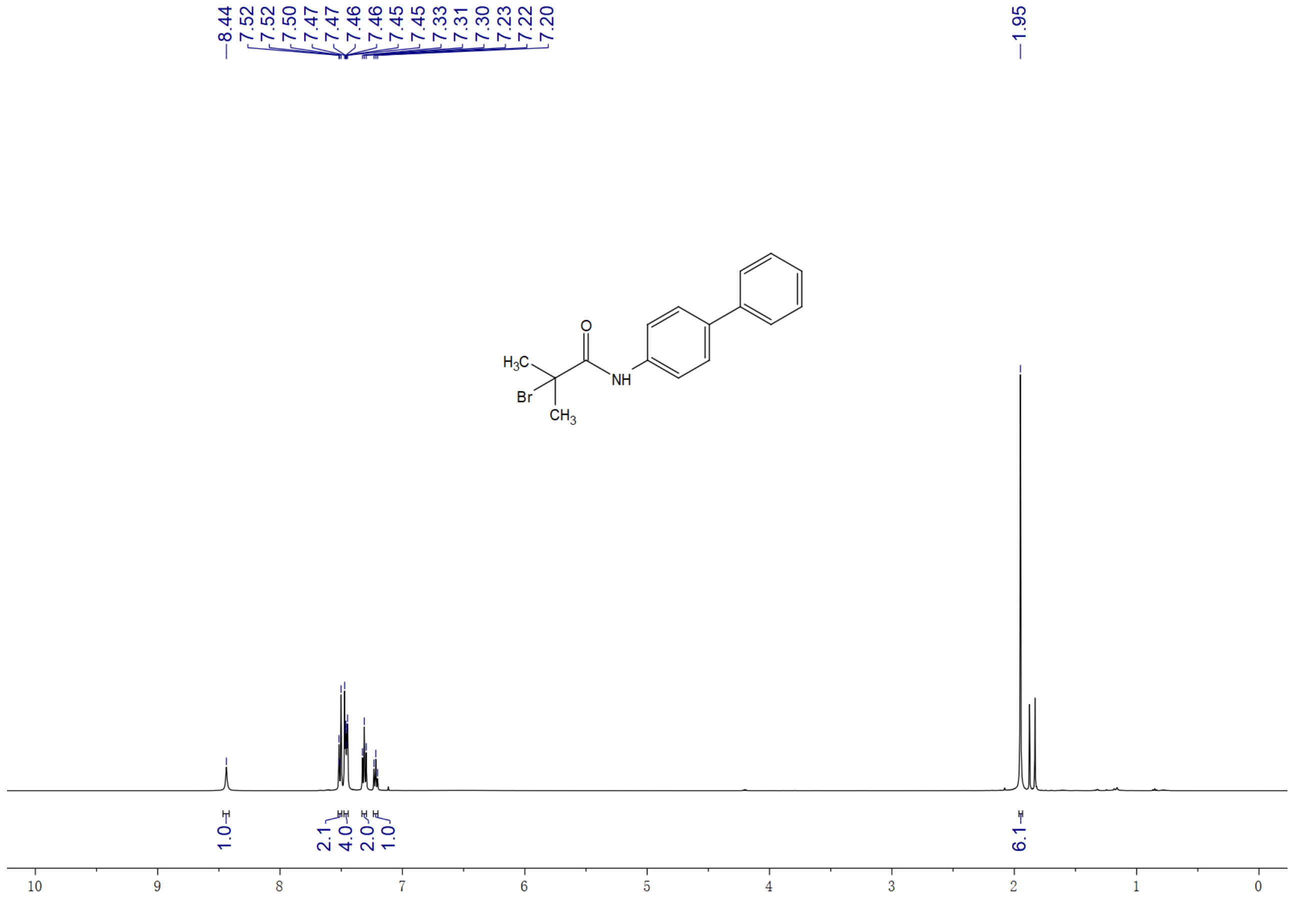

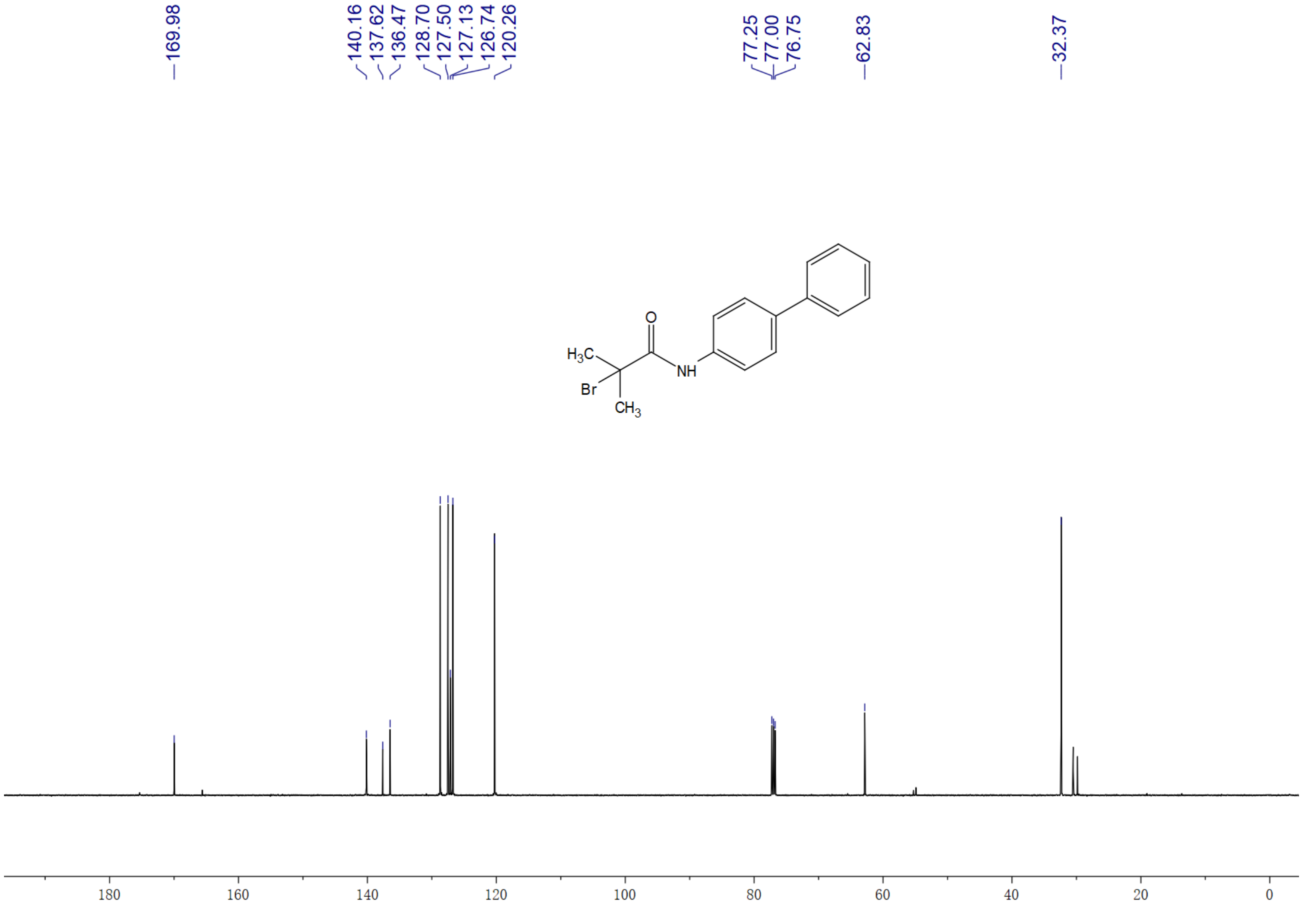

2-Bromo-*N*-(2,6-difluorophenyl)-2-methylpropanamide, **1e**, ^{1}H NMR (500 MHz, $CDCl_3$) and ^{13}C NMR (125 MHz, $CDCl_3$)

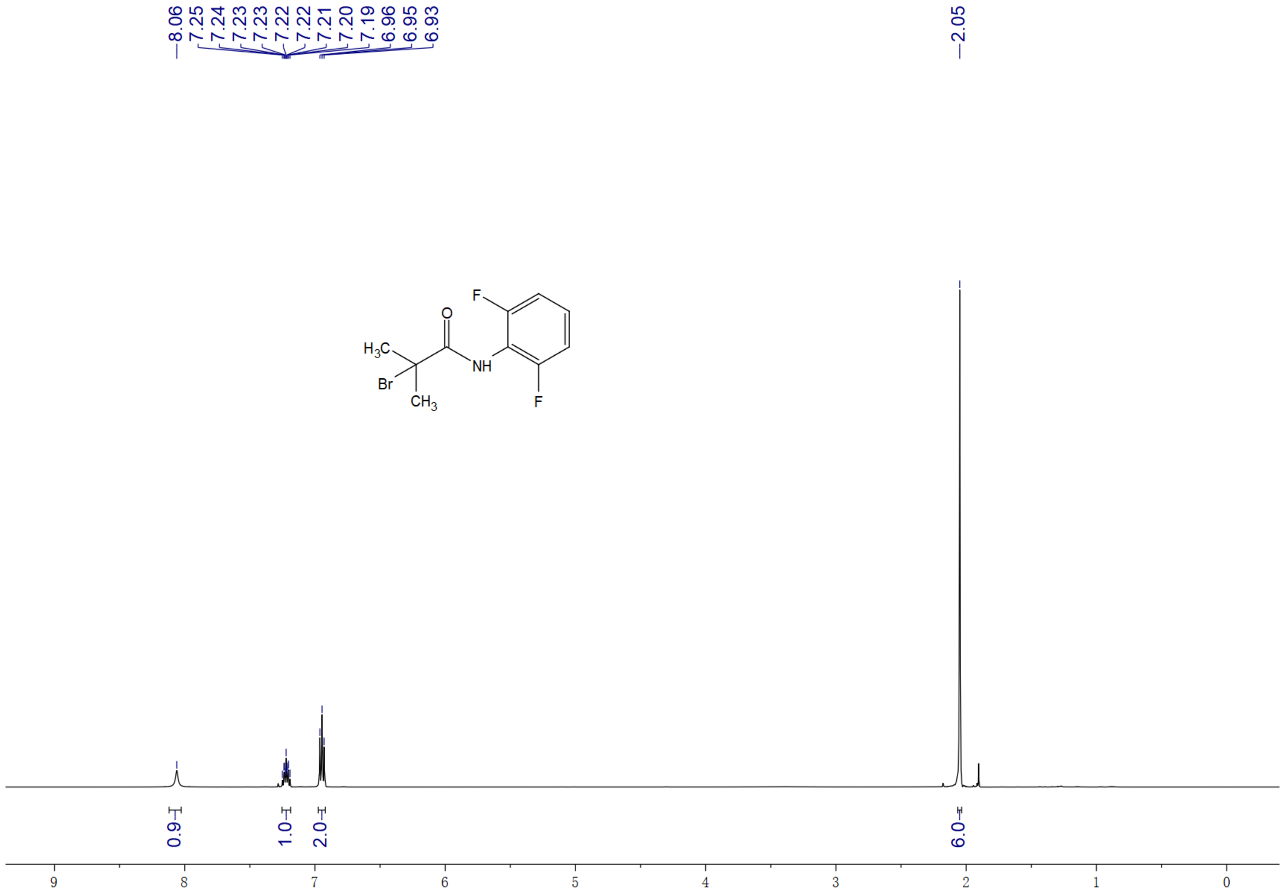

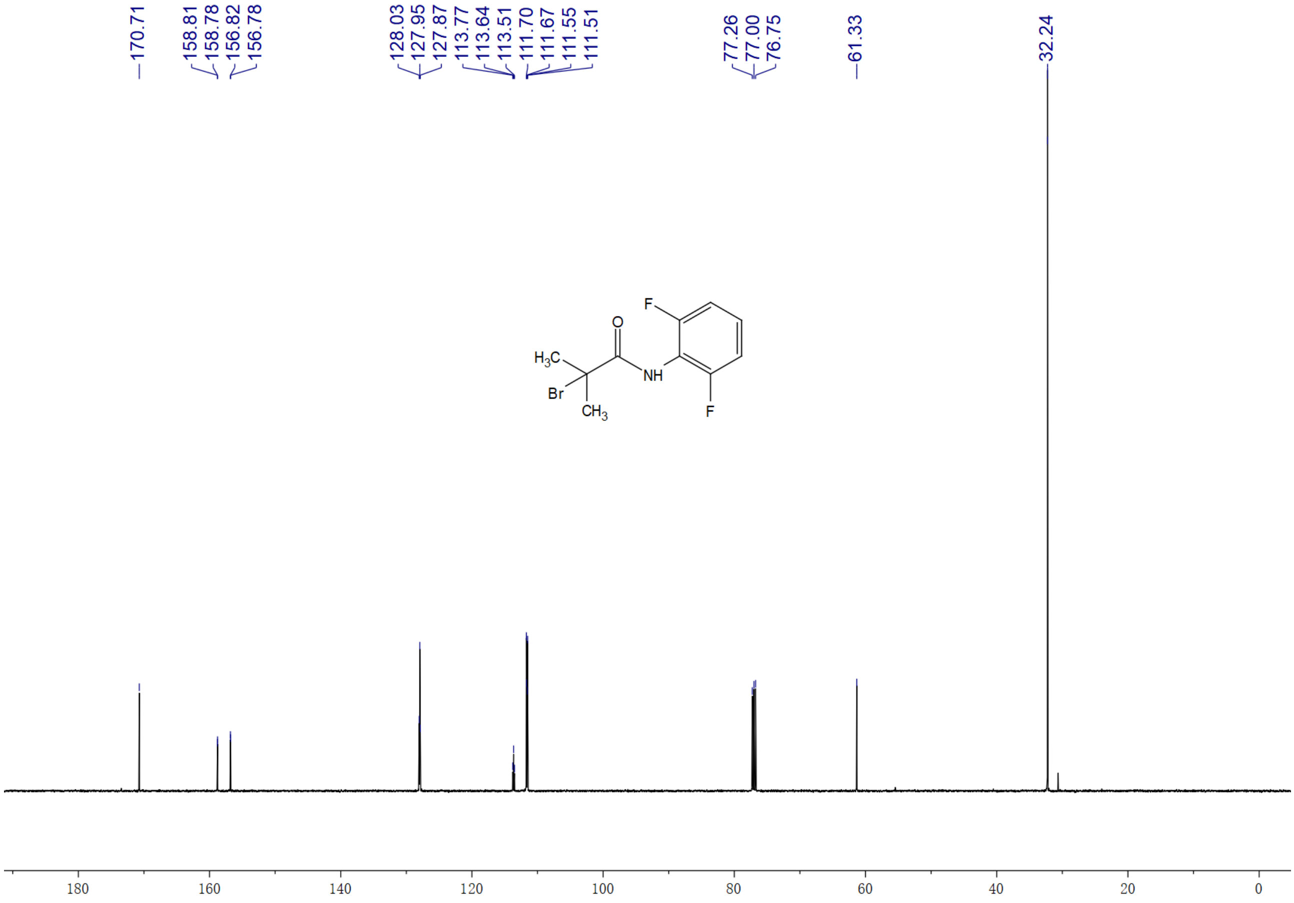

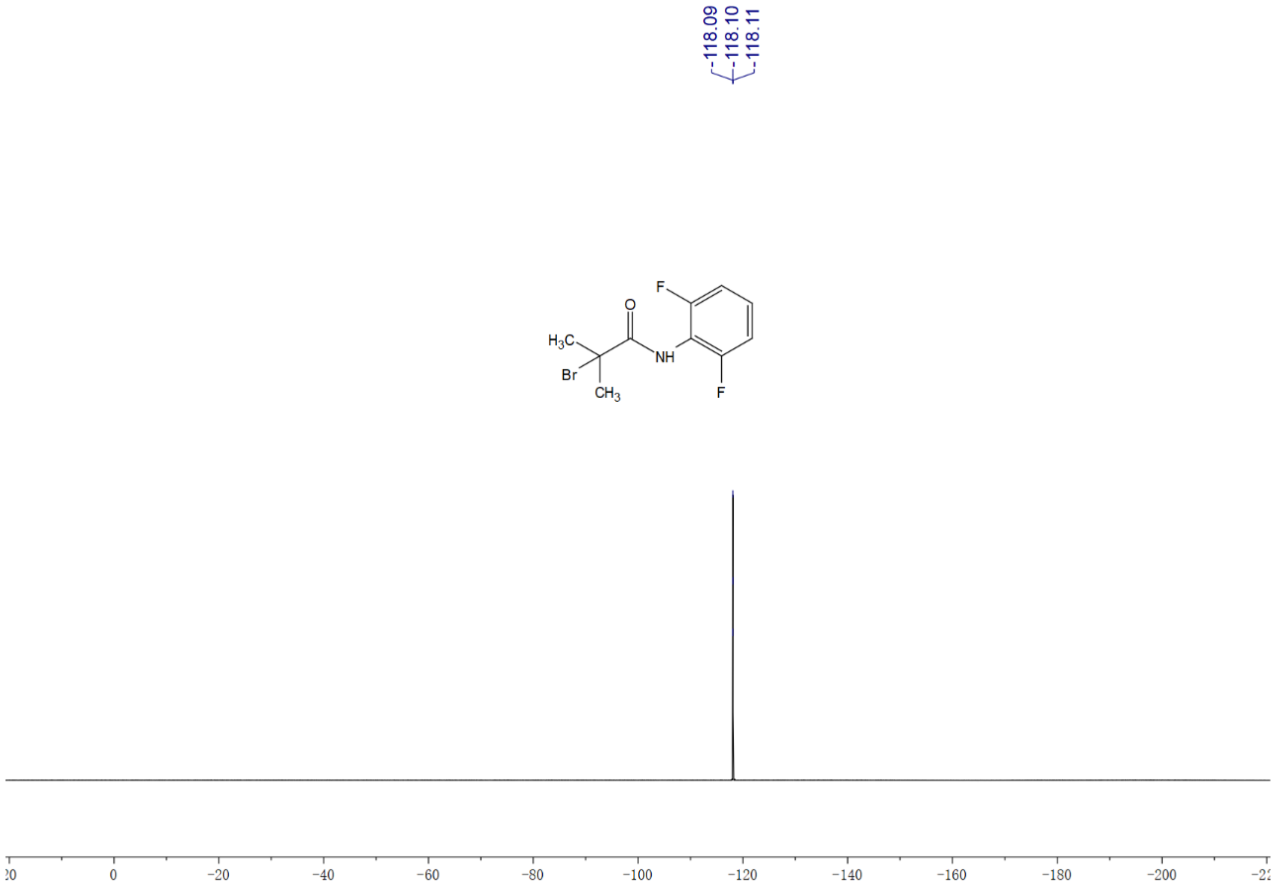
-118.09
-118.10
-118.11
F
O
H3C
NH
Br
CH3
F
0
−20
−40
−60
−80
−100
−120
−140
−160
−180
−200

2-Bromo-2-methyl-*N*-(6-methylpyridin-2-yl)propenamide, **1g**, ^{1}H NMR (500 MHz, $CDCl_3$) and ^{13}C NMR (125 MHz, $CDCl_3$)

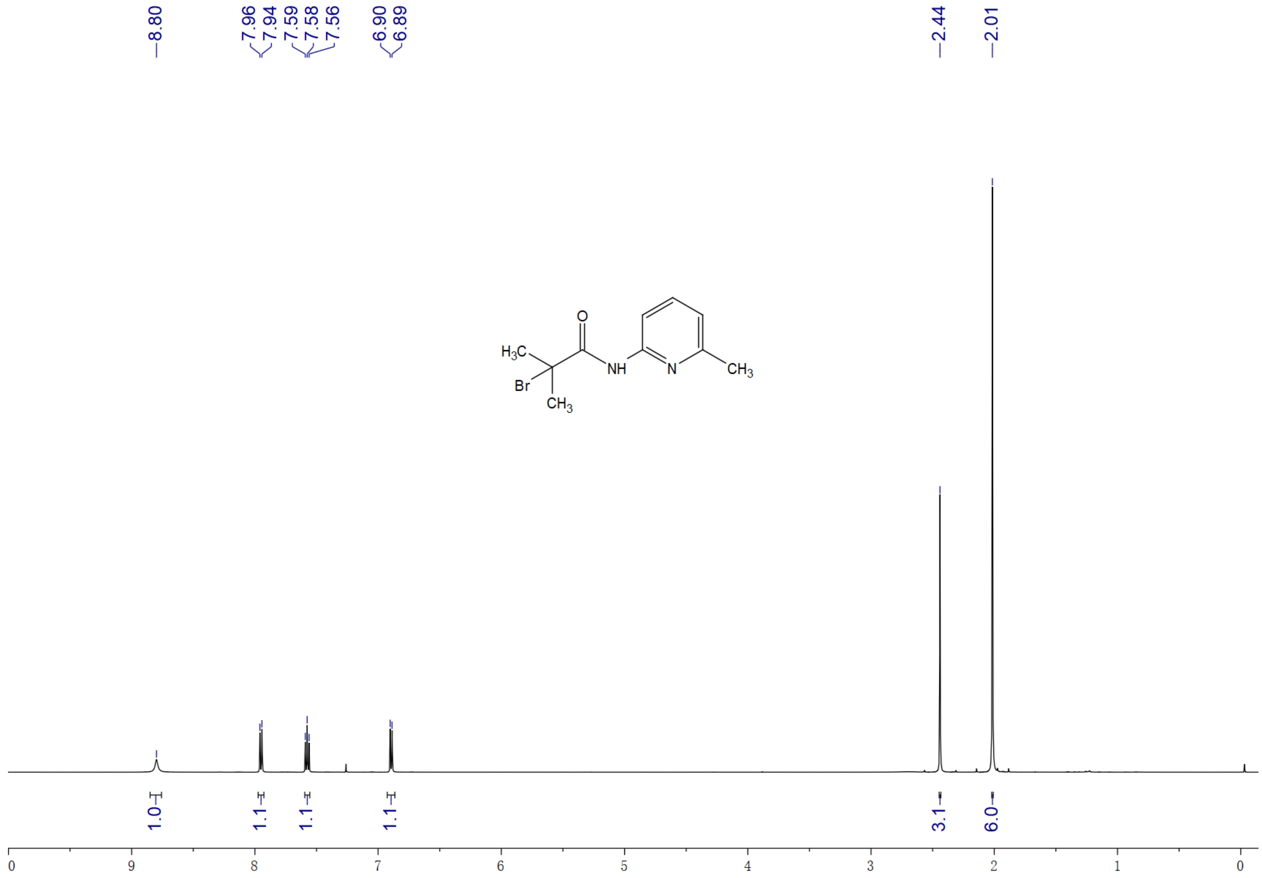

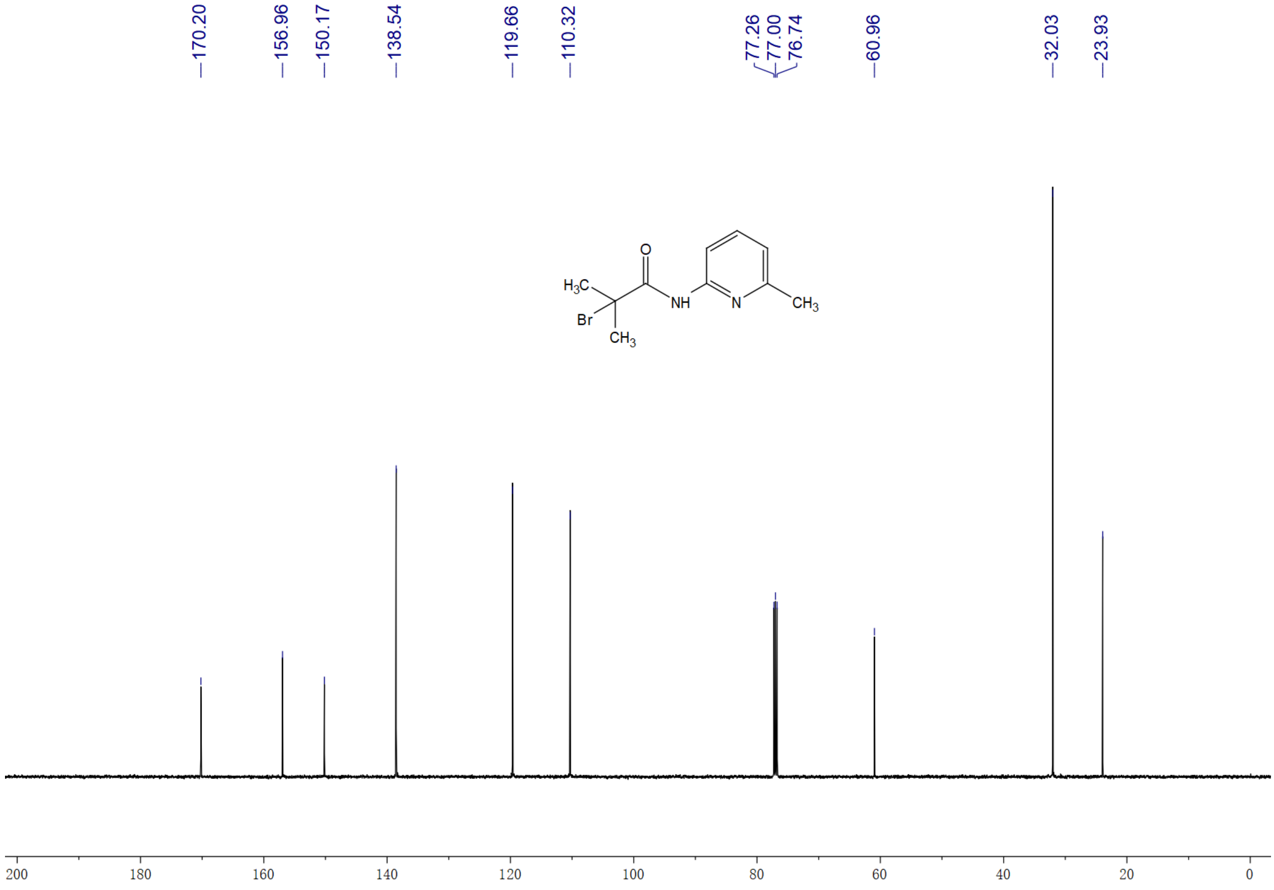

Methyl-(2-bromo-2-methylpropanoyl)phenylalaninate,**1i**, ^{1}H NMR (500 MHz, $CDCl_3$) and ^{13}C NMR (125 MHz, $CDCl_3$)

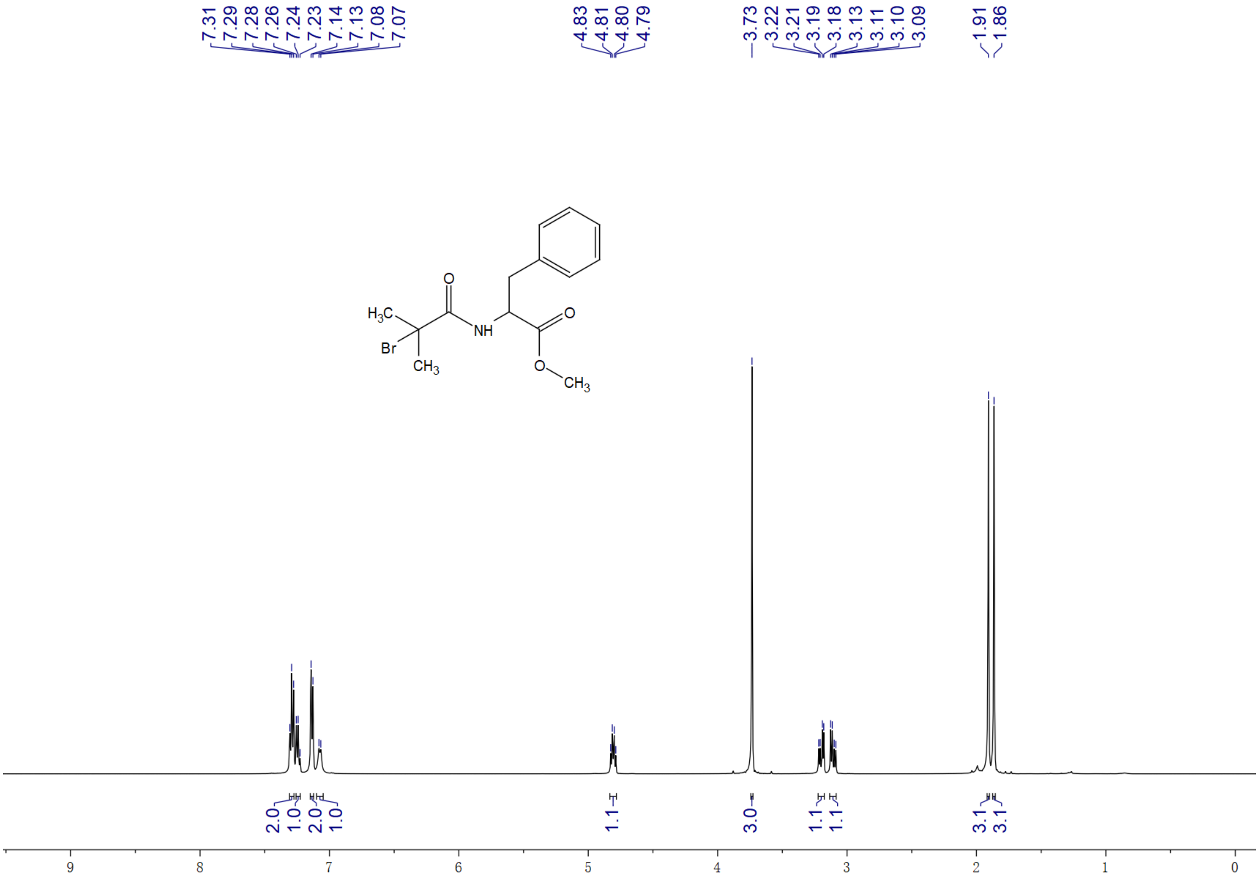

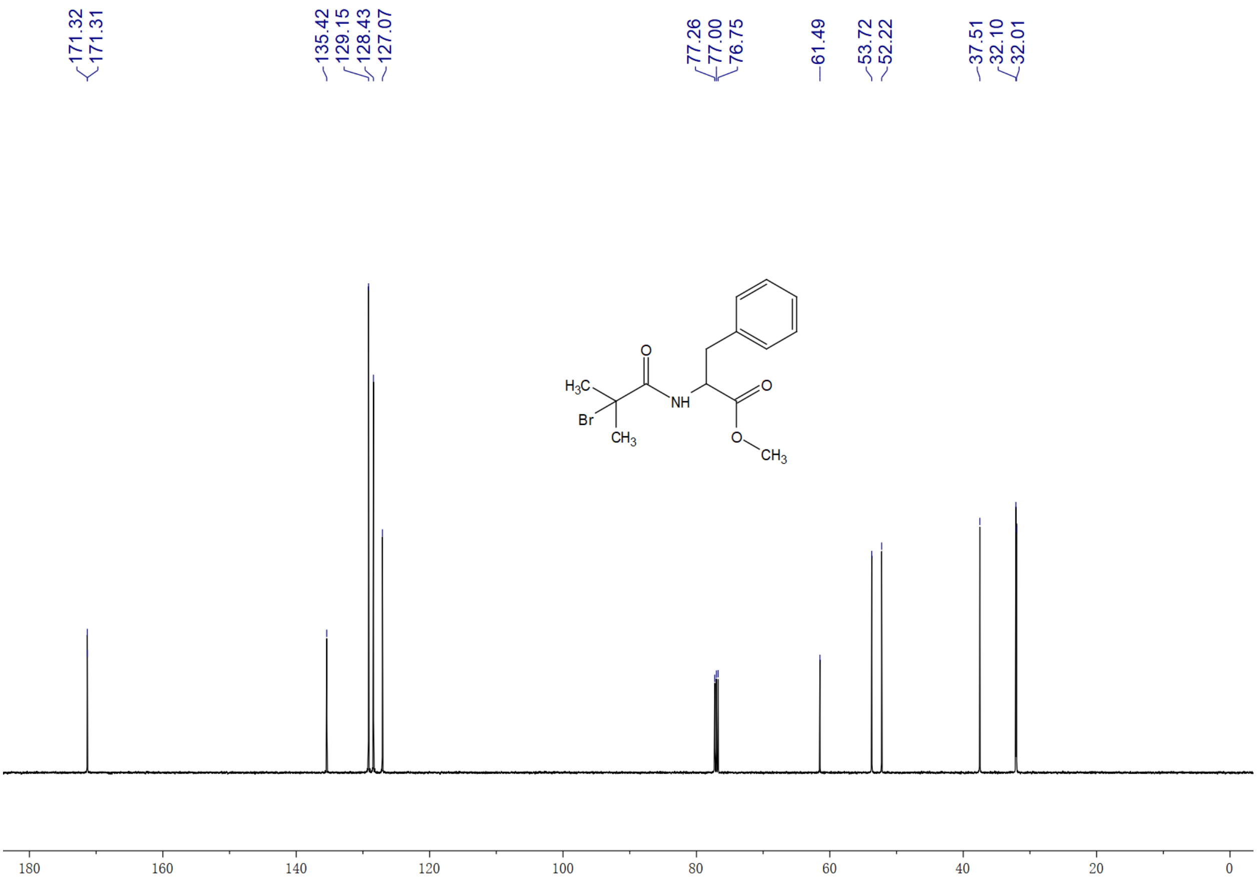

Methyl-(2-bromo-2-methylpropanoyl)glycinate, **1j**, ^1H NMR (500 MHz, $CDCl_3$) and ^{13}C NMR (125 MHz, $CDCl_3$)

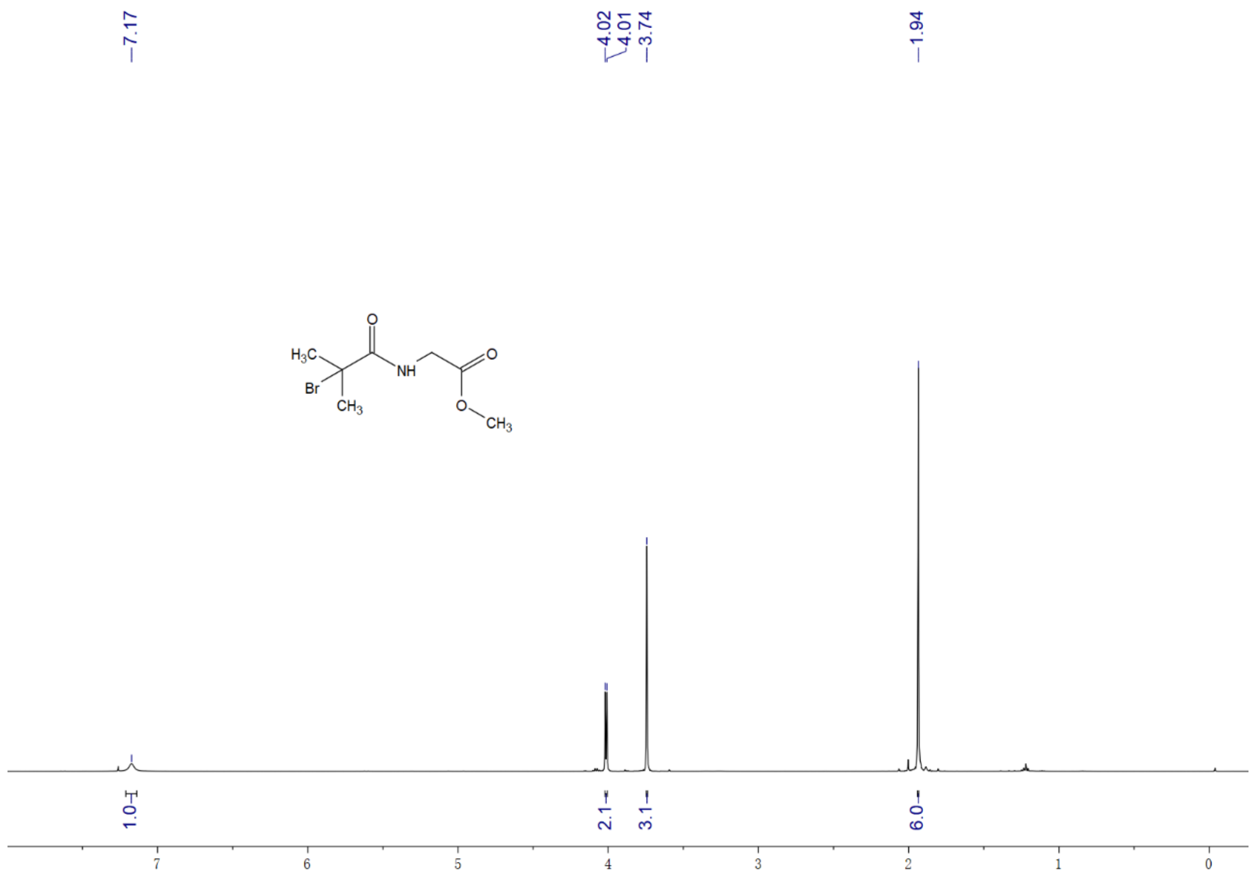

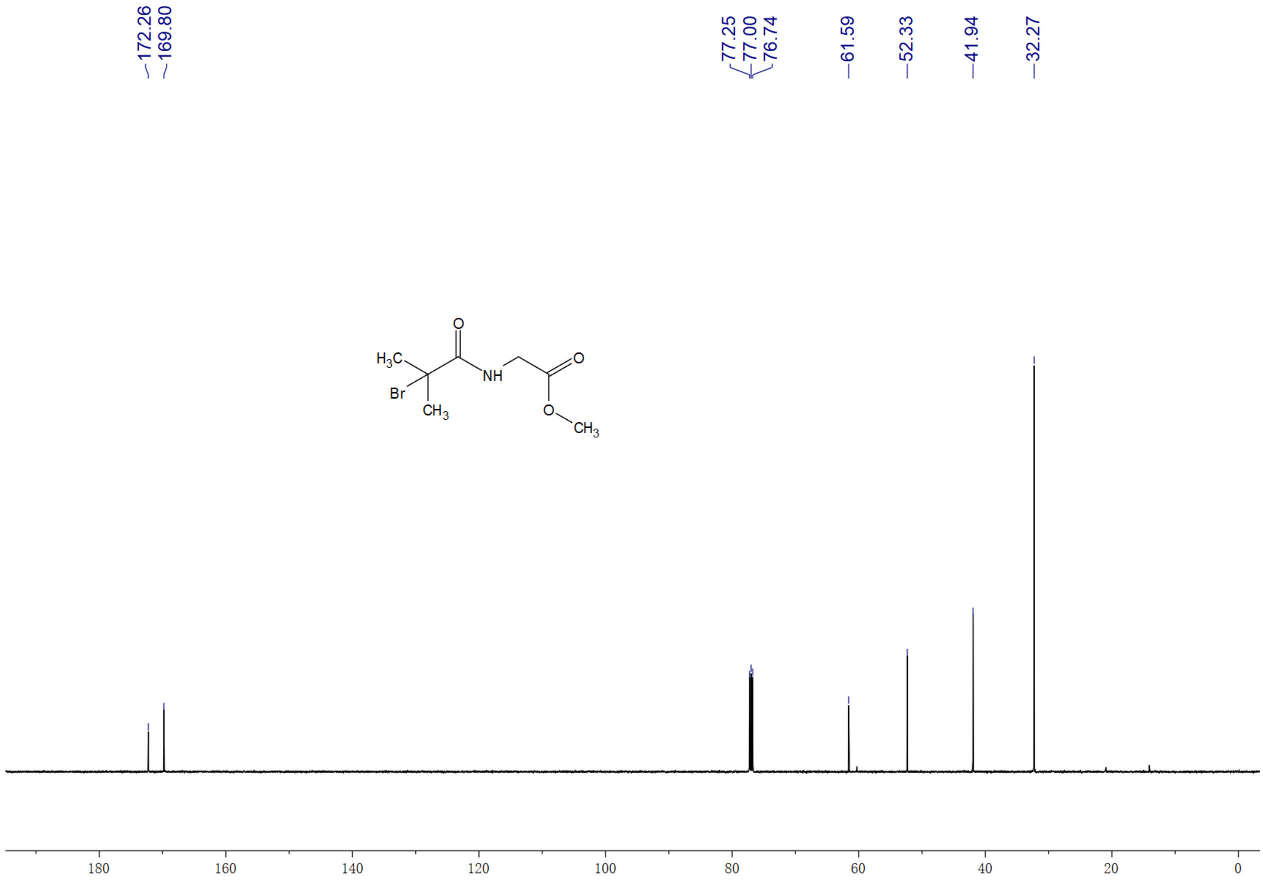

2-Bromo-N-(4-(3-ethyl-2,6-dioxopiperidin-3-yl)phenyl)-2-methylpropanamide, **1aj**, ^{1}H NMR (500 MHz, $CDCl_3$) and ^{13}C NMR (125 MHz, $CDCl_3$)

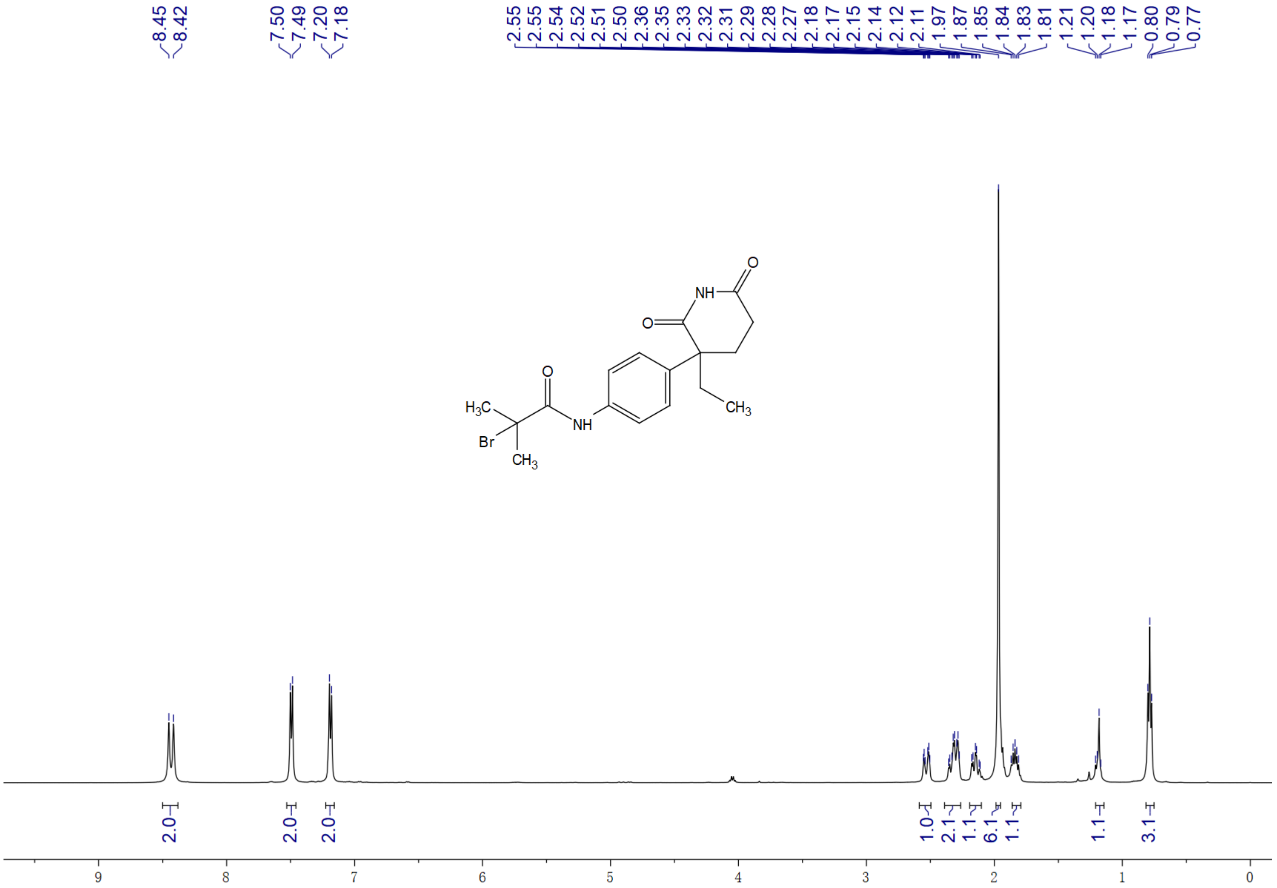

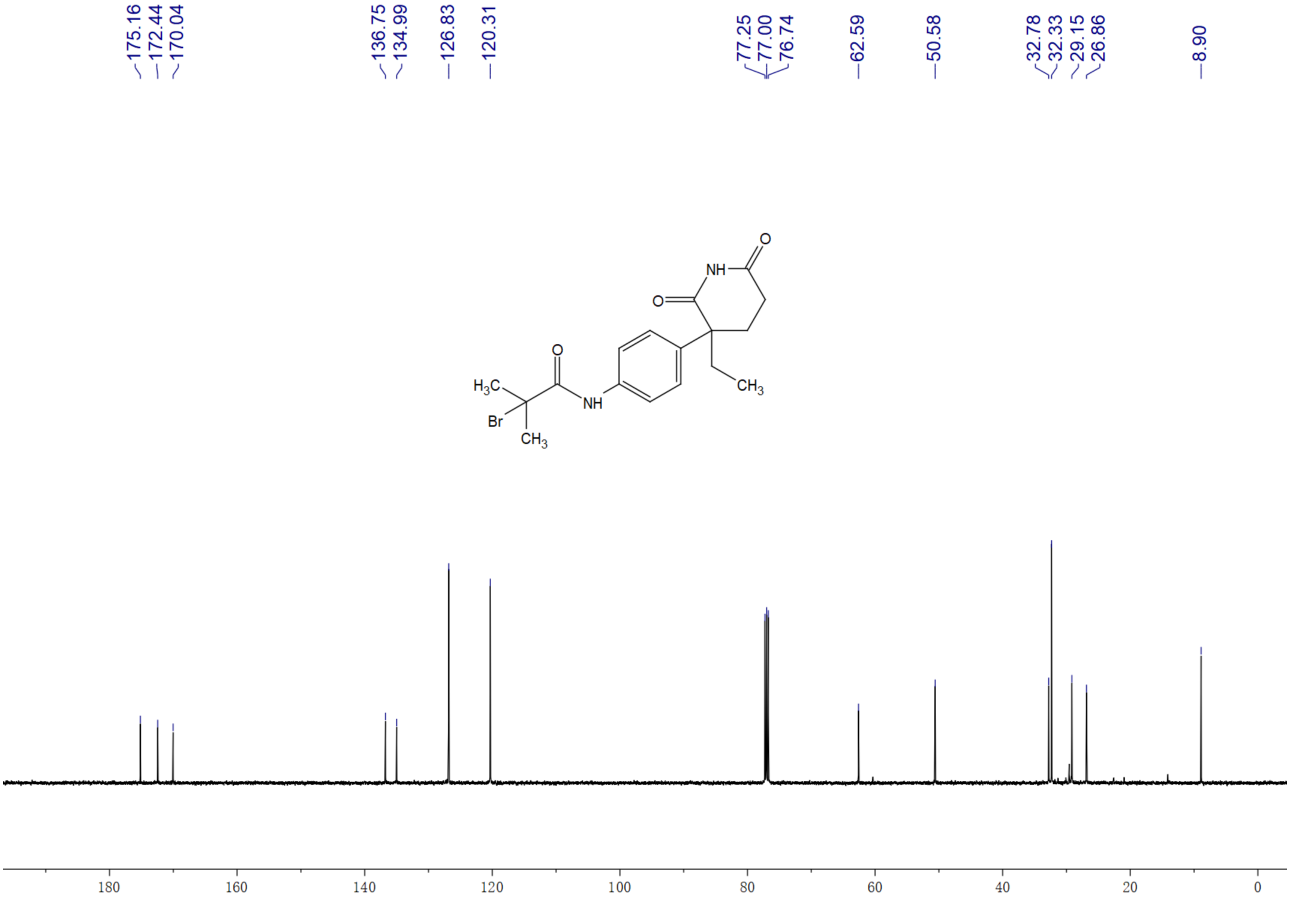

2-Bromo-N-(1-(2,6-dimethylphenoxy)propan-2-yl)-2-methylpropanamide, **1al**, ^{1}H NMR (500 MHz, $CDCl_3$) and ^{13}C NMR (125 MHz, $CDCl_3$)

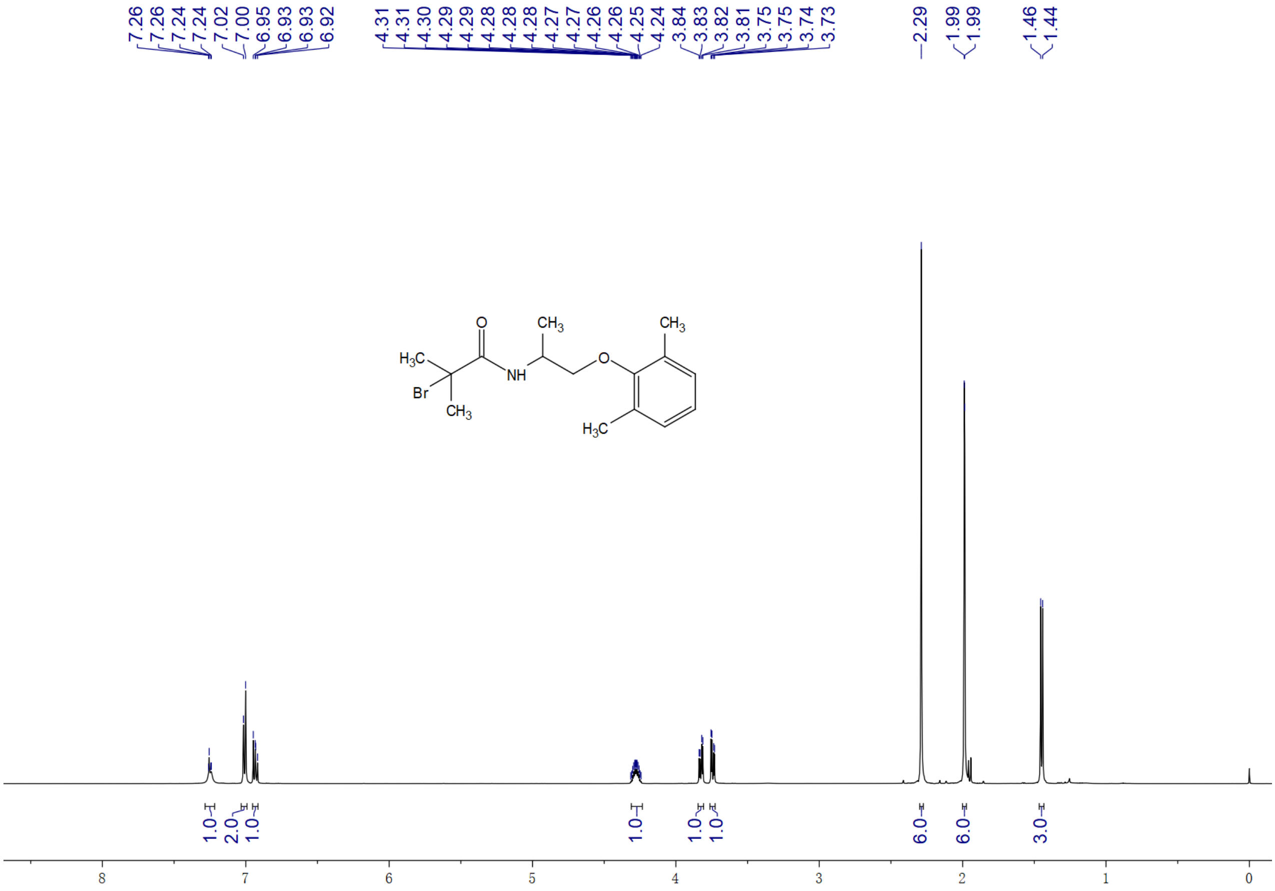

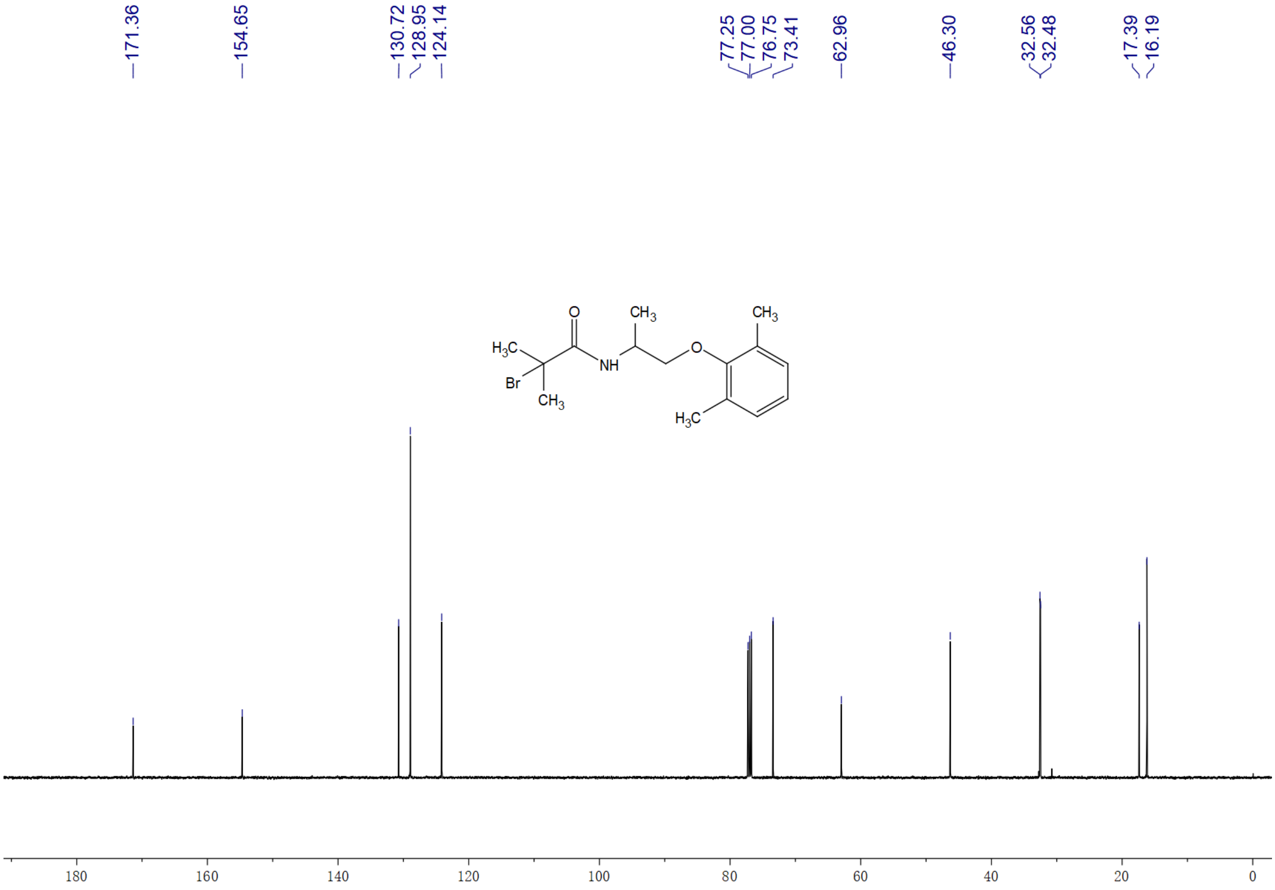

2-(Thiophen-2-yl)ethyl 2-bromo-2-methylpropanoate, **1t**, ^{1}H NMR (400 MHz, $CDCl_3$) and ^{13}C NMR (100 MHz, $CDCl_3$)

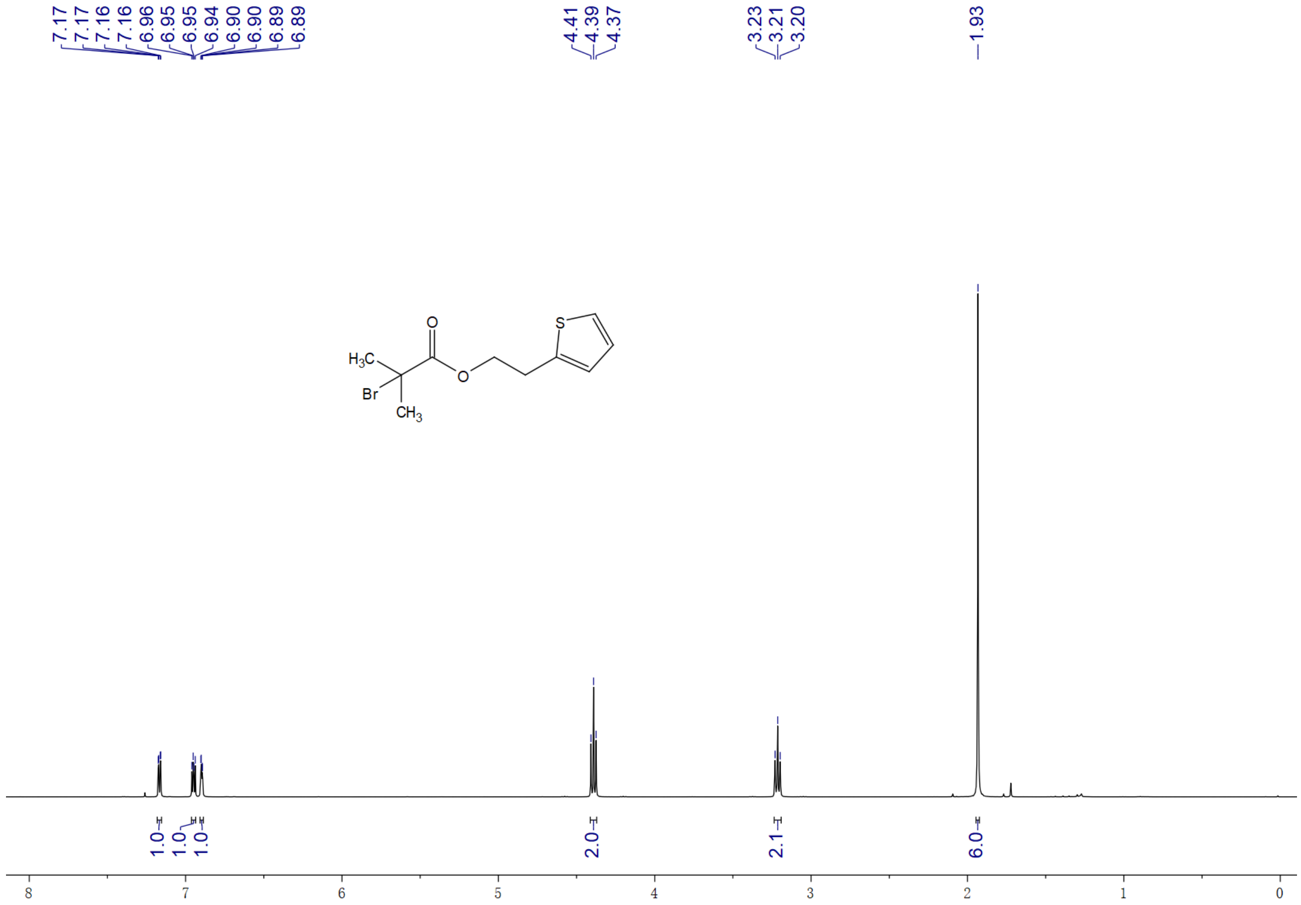

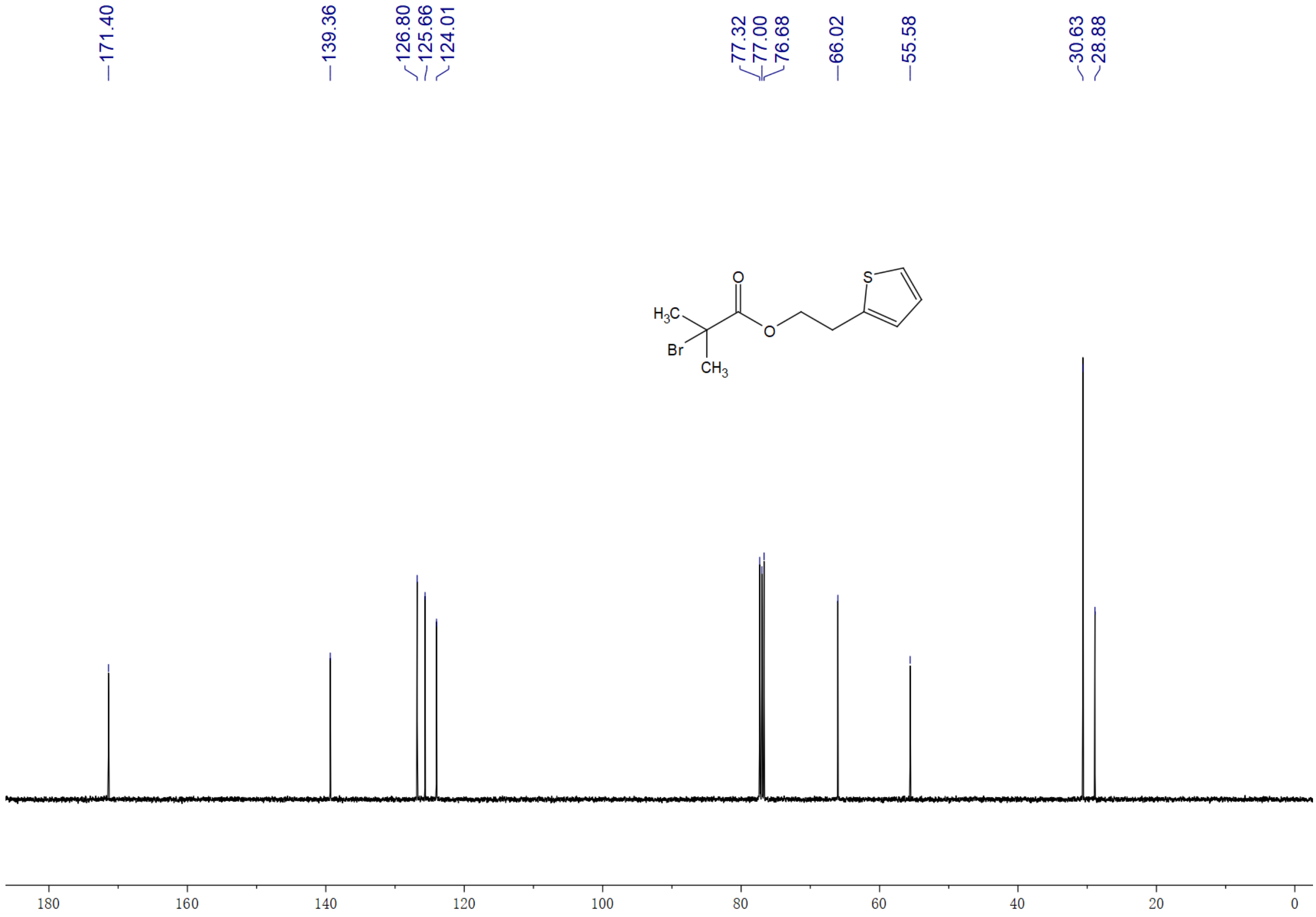

Methyl-(2-bromo-2,2-difluoroacetyl)alaninate, **1aa**, ^{1}H NMR (500 MHz, $CDCl_3$), ^{13}C NMR (125 MHz, $CDCl_3$) and ^{19}F NMR (471 MHz, $CDCl_3$)

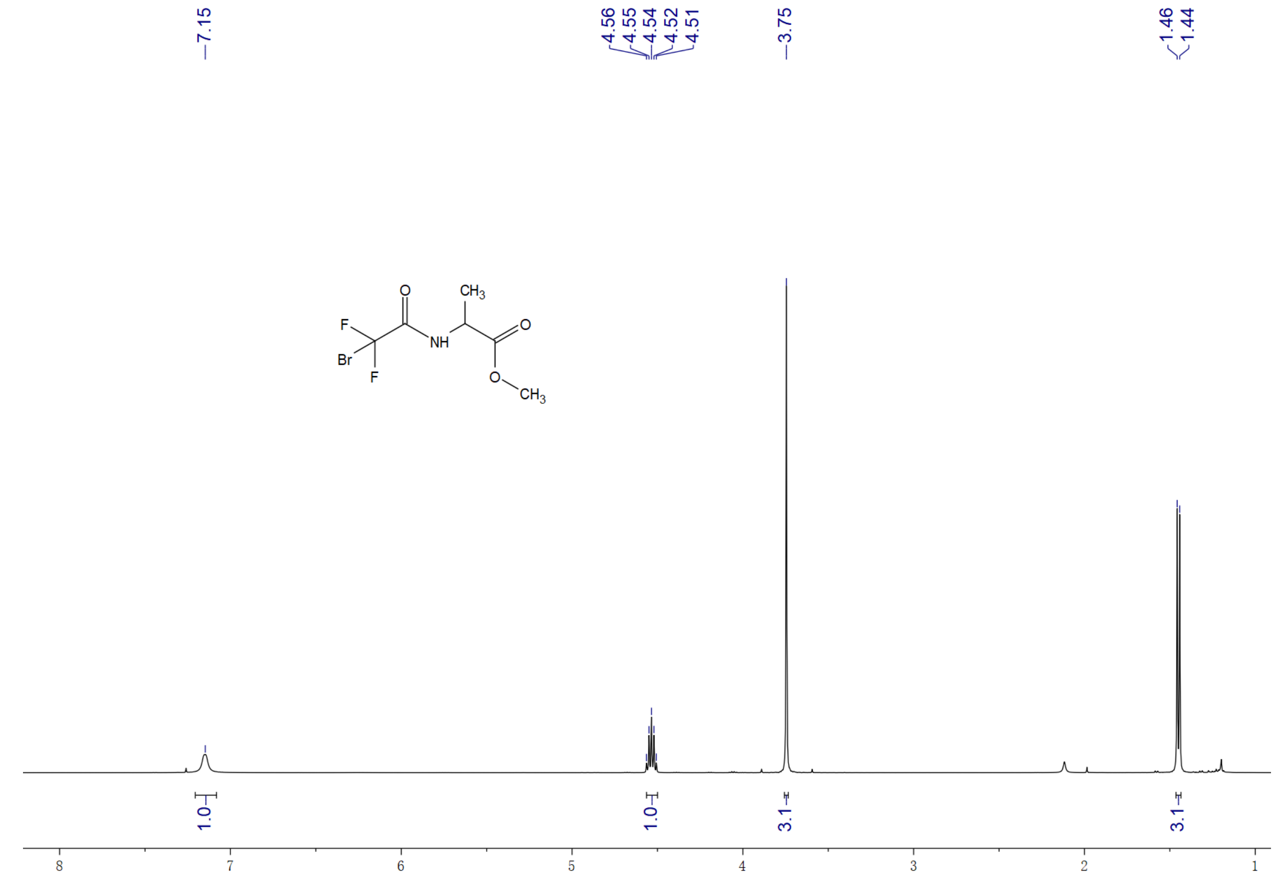

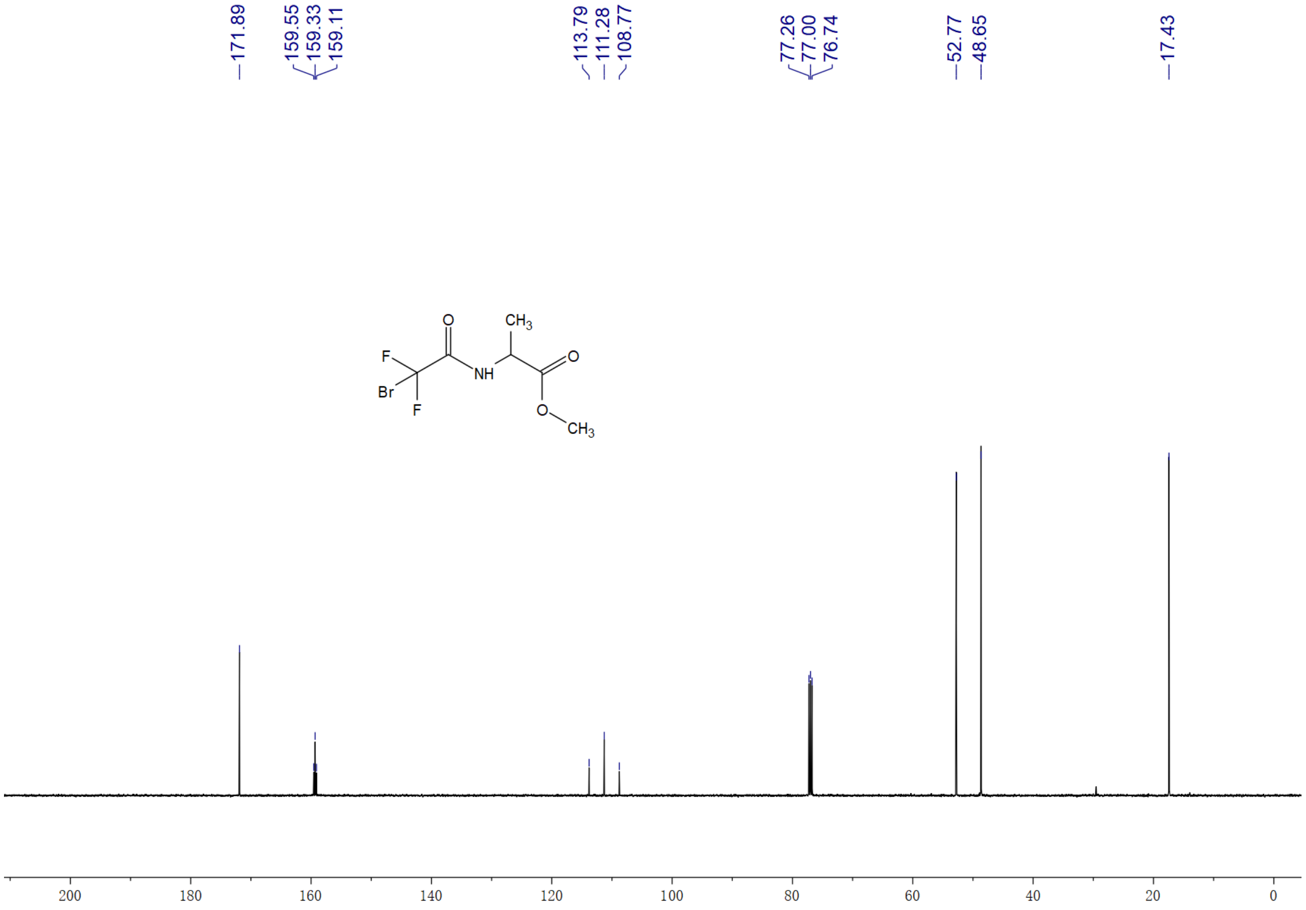

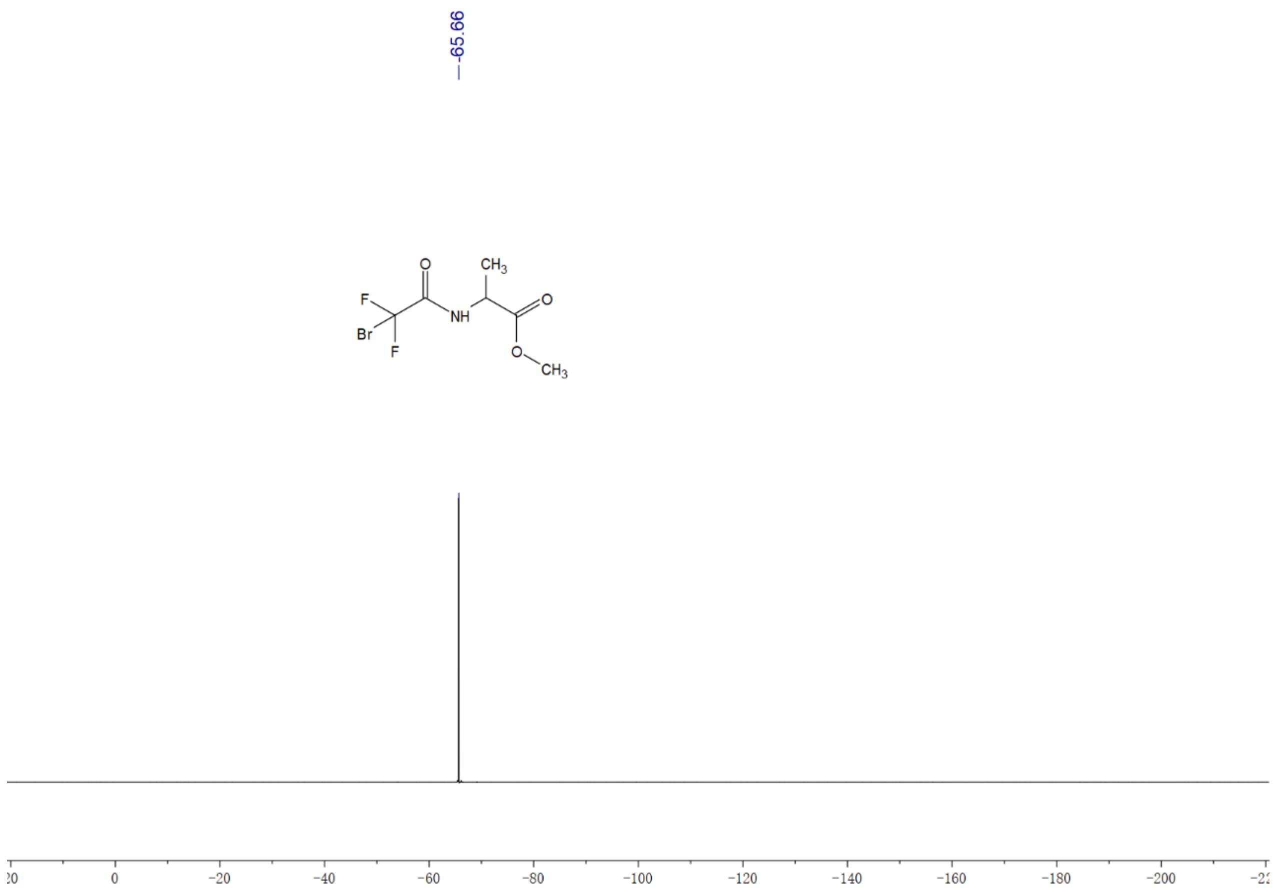
-65.66
O
CH3
F
NH
O
Br
F
O
CH3
20
0
-20
-40
-60
-80
-100
-120
-140
-160
-180
-200

Dimethyl-(2-bromo-2,2-difluoroacetyl)aspartate, **1ac**, ^{1}H NMR (500 MHz, $CDCl_3$), ^{13}C NMR (125 MHz, $CDCl_3$) and ^{19}F NMR (471 MHz, $CDCl_3$)

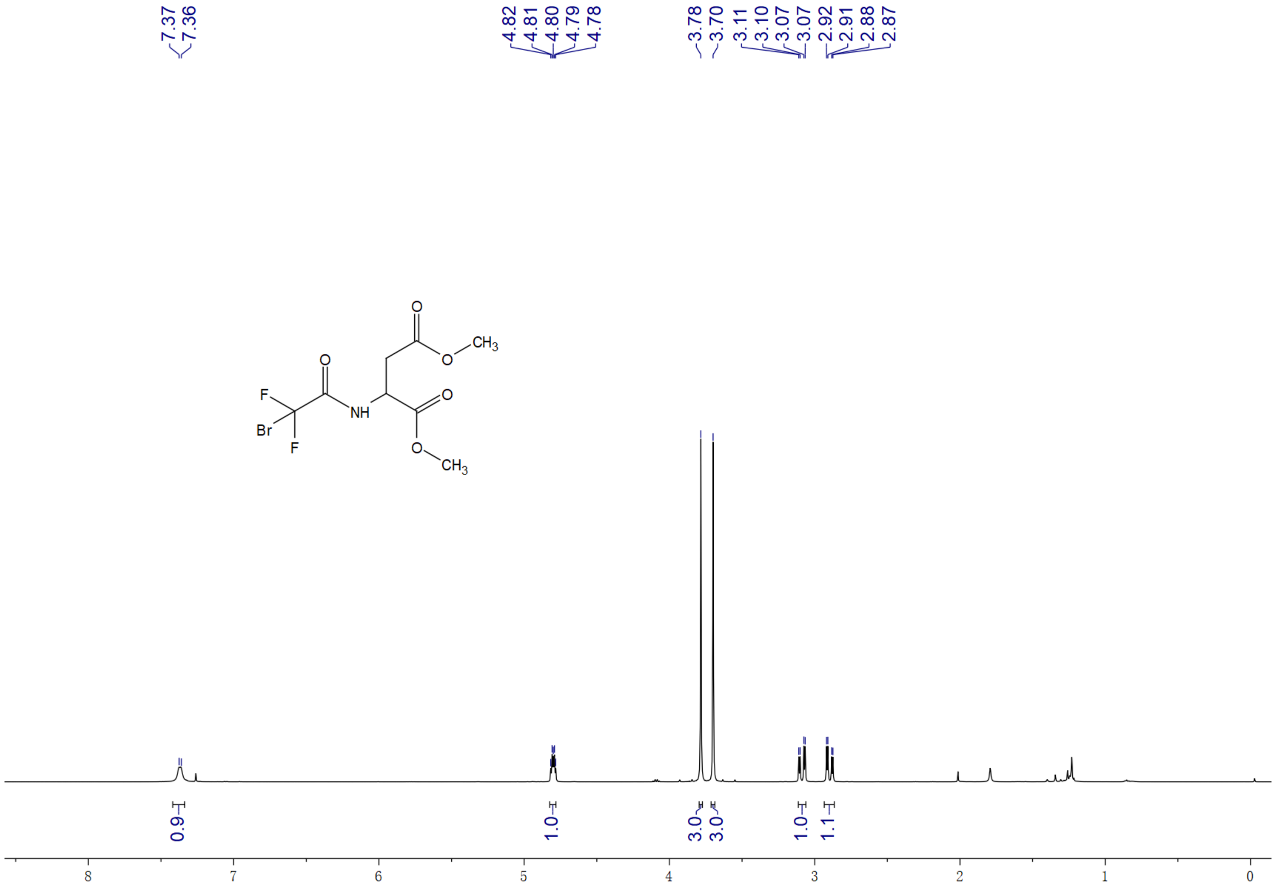

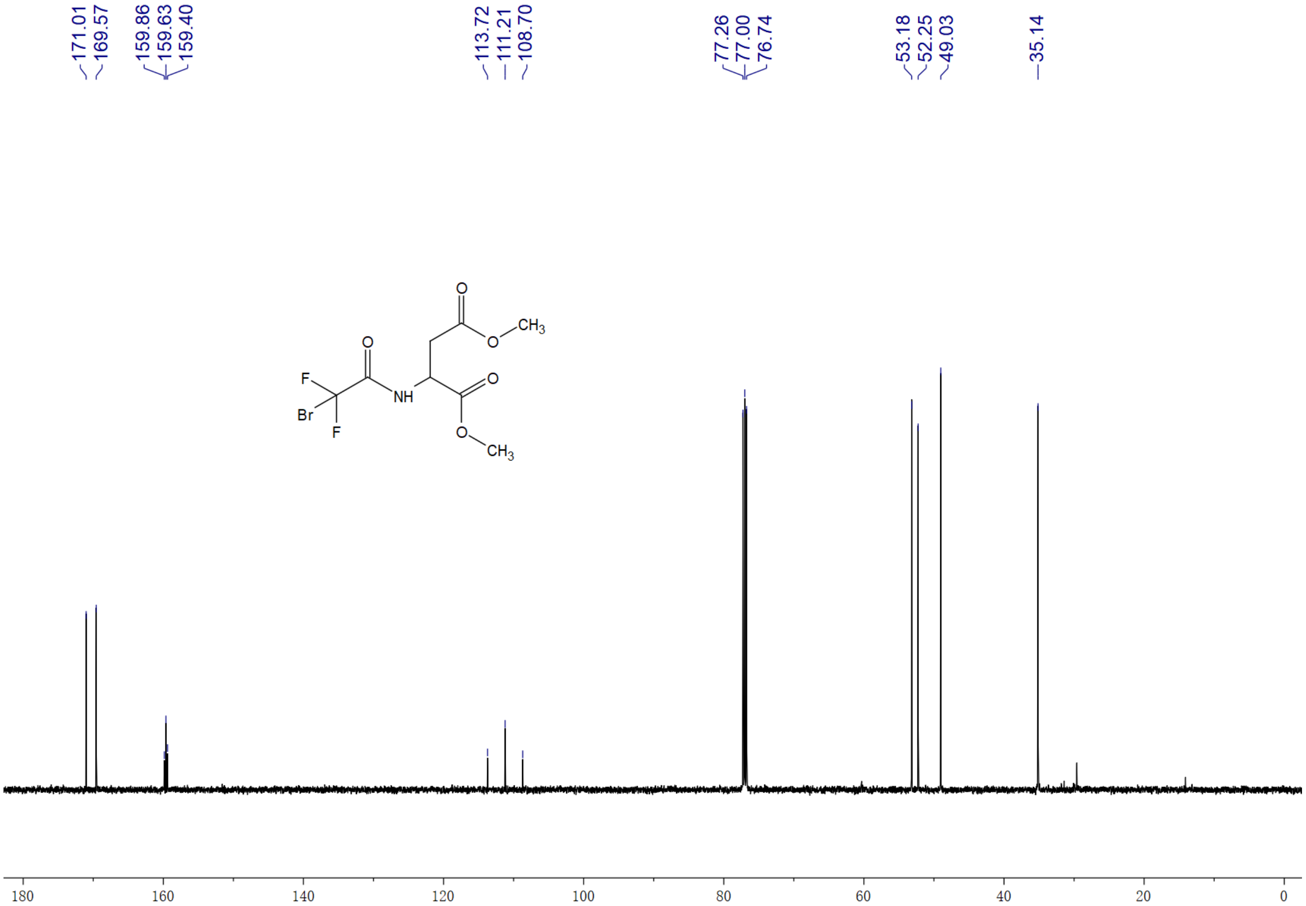

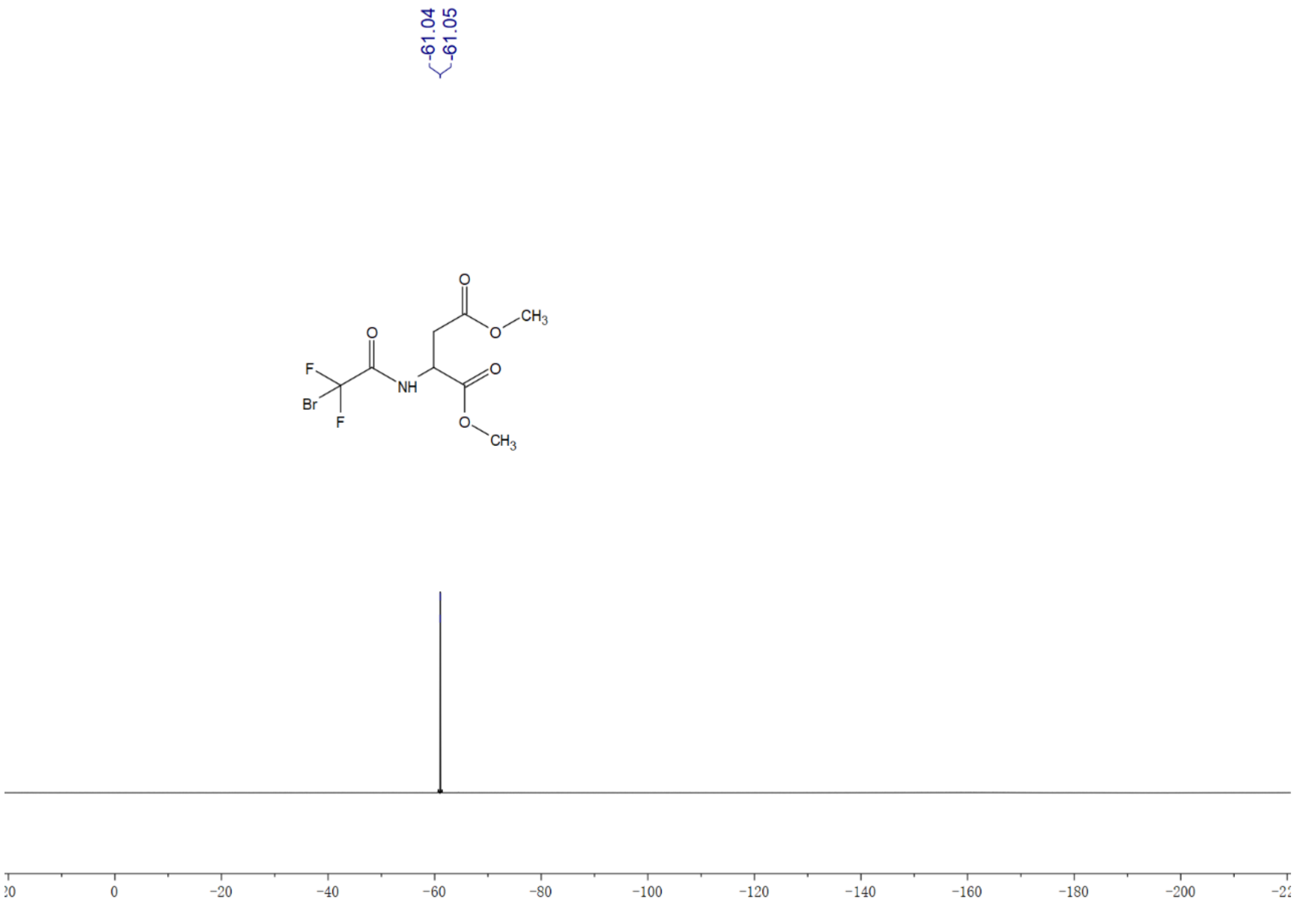
-61.04
-61.05
F
Br
F
O
NH
O
O
CH3
O
O
CH3
20
0
−20
−40
−60
−80
−100
−120
−140
−160
−180
−200

Methyl-*N*-(2-bromo-2,2-difluoroacetyl)-O-(tert-butyl)serinate, **1ad**, ^{1}H NMR (500 MHz, $CDCl_3$), ^{13}C NMR (125 MHz, $CDCl_3$) and ^{19}F NMR (471 MHz, $CDCl_3$)

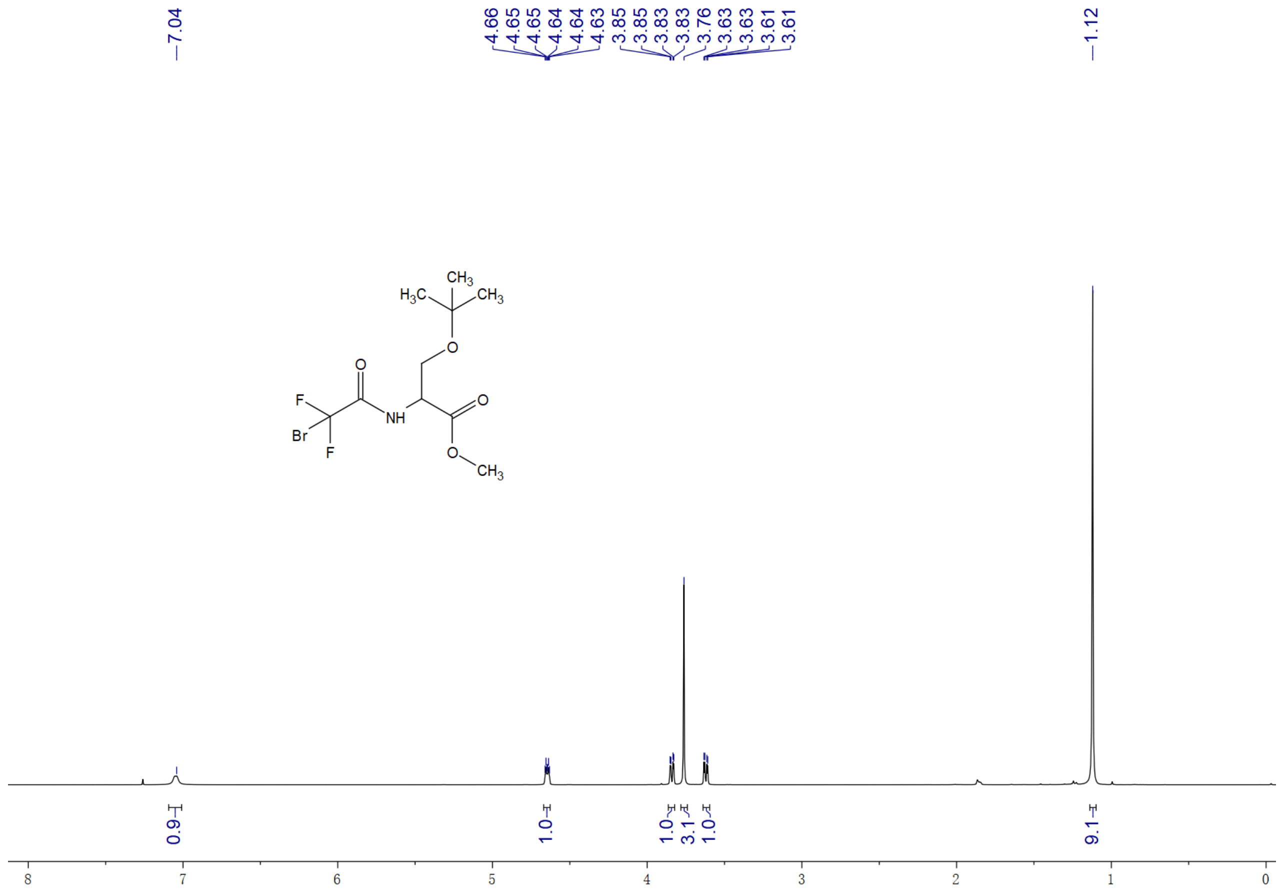

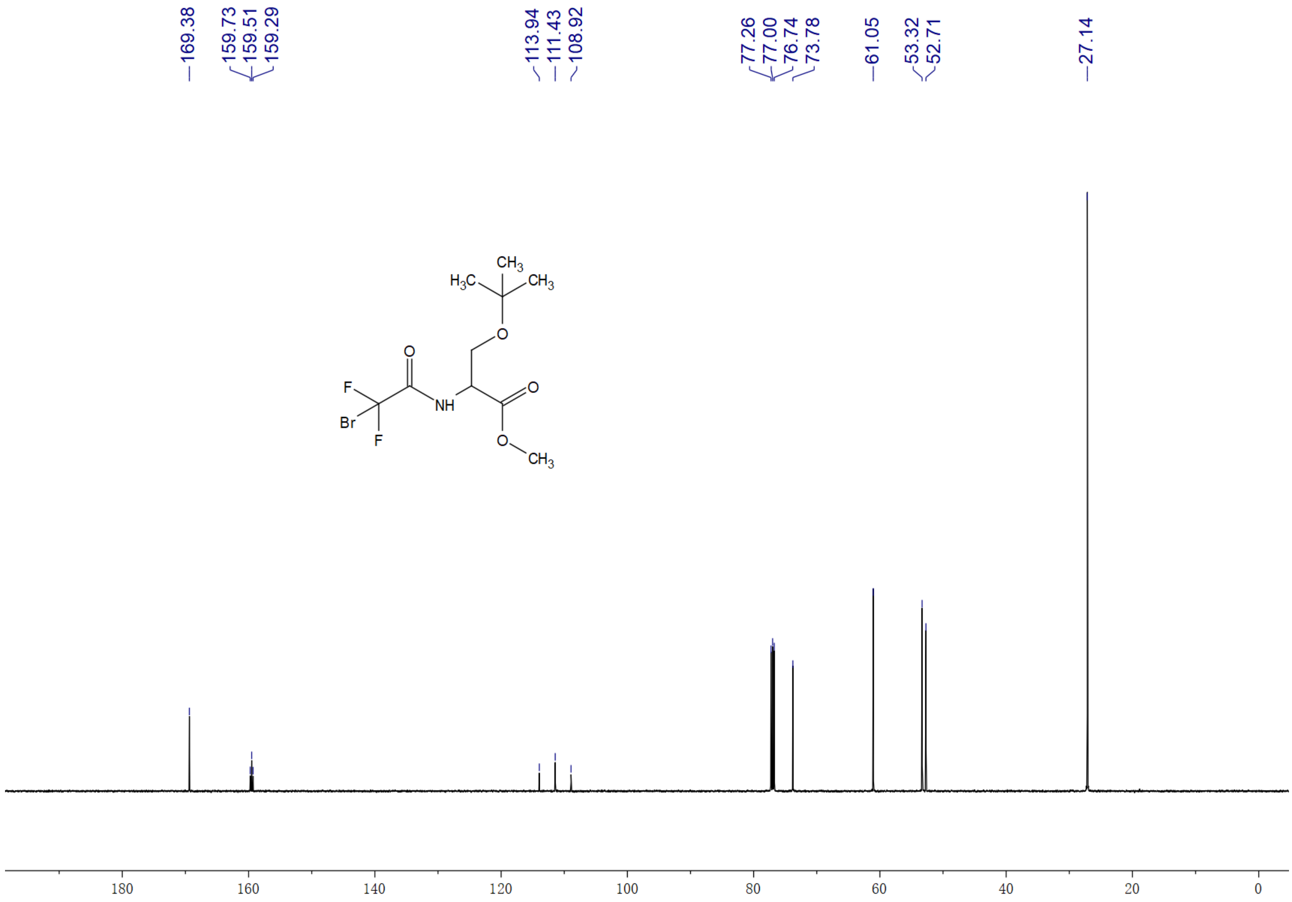

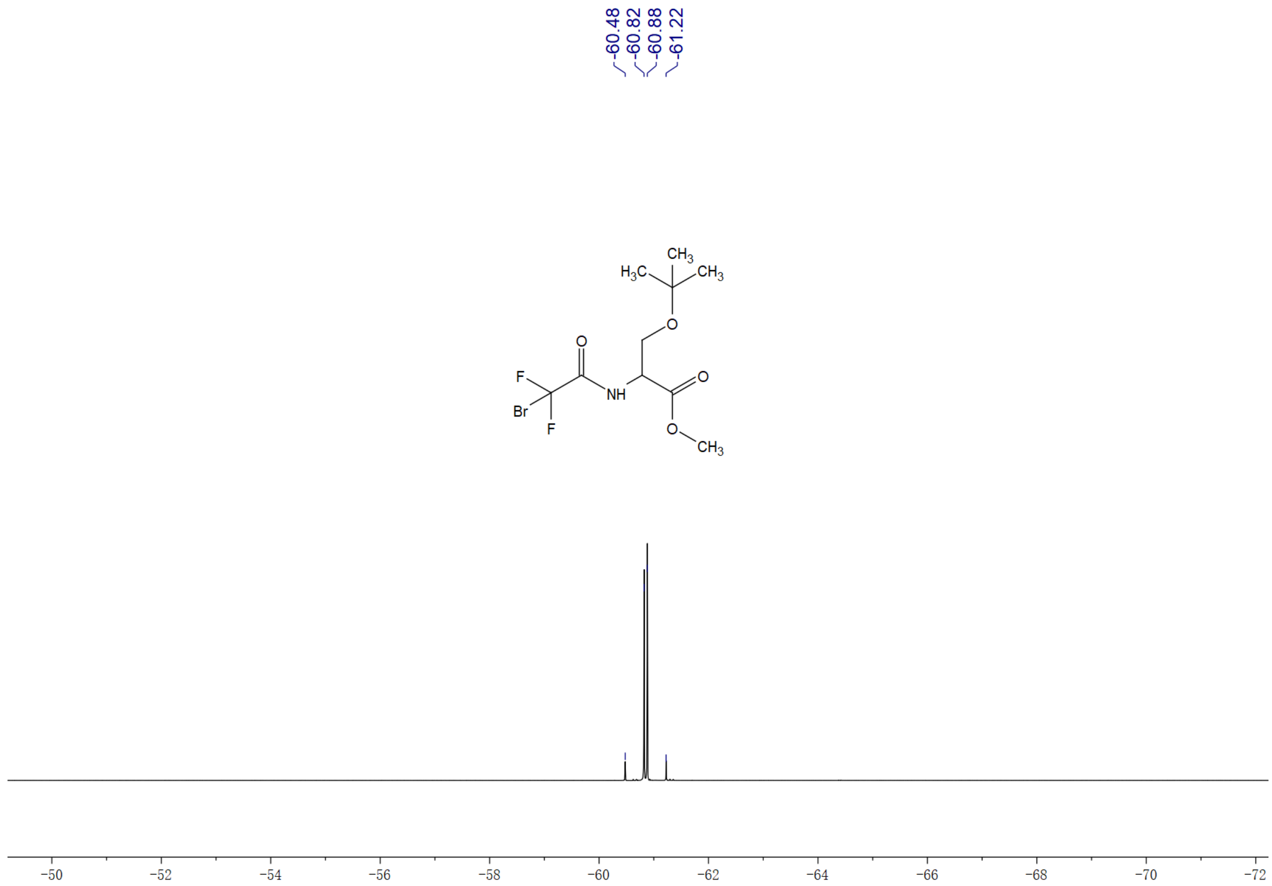

-60.48
-60.82
-60.88
-61.22
CH3
H3C
CH3
O
O
F
NH
O
Br
F
O
CH3
-50
-52
-54
-56
-58
-60
-62
-64
-66
-68
-70
-72

Methyl-3-(2-methyl-1-oxo-1-(phenylamino)propan-2-yl)benzoate, **3aa**, ^{1}H NMR (500 MHz, $CDCl_3$) and ^{13}C NMR (125 MHz, $CDCl_3$)

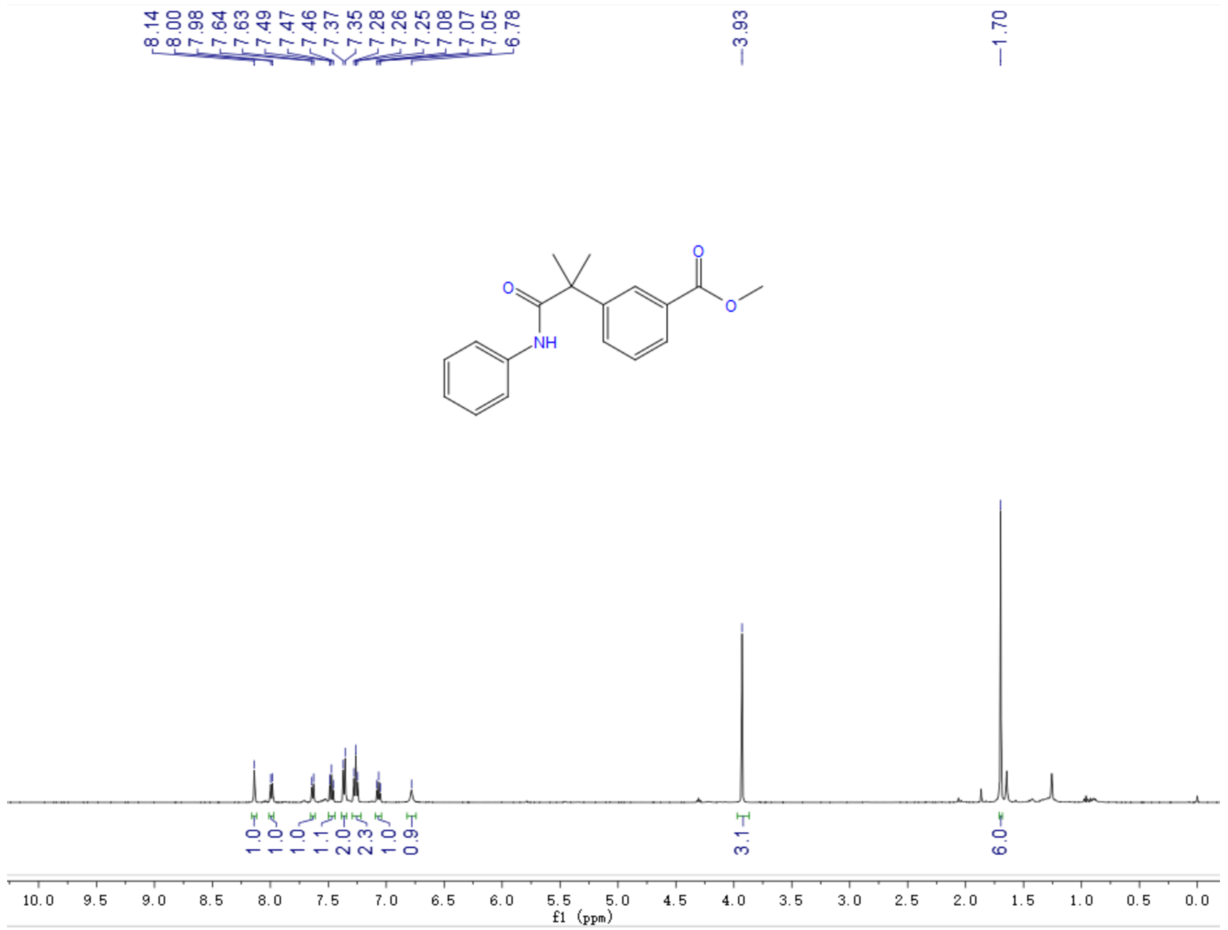

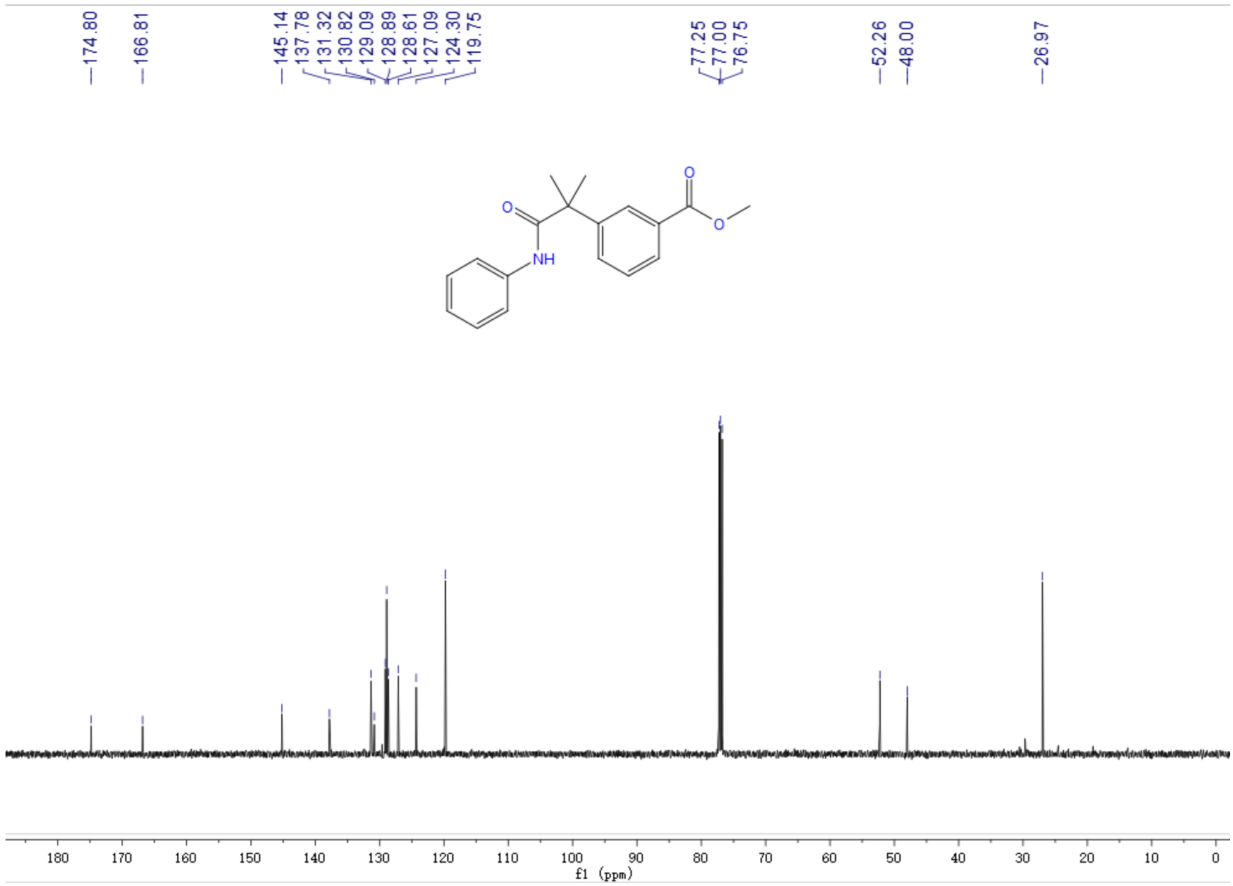

Methyl-2-methyl-3-(2-methyl-1-oxo-1-(phenylamino)propan-2-yl)benzoate, **3ba**, ^{1}H NMR (500 MHz, $CDCl_3$) and ^{13}C NMR (125 MHz, $CDCl_3$)

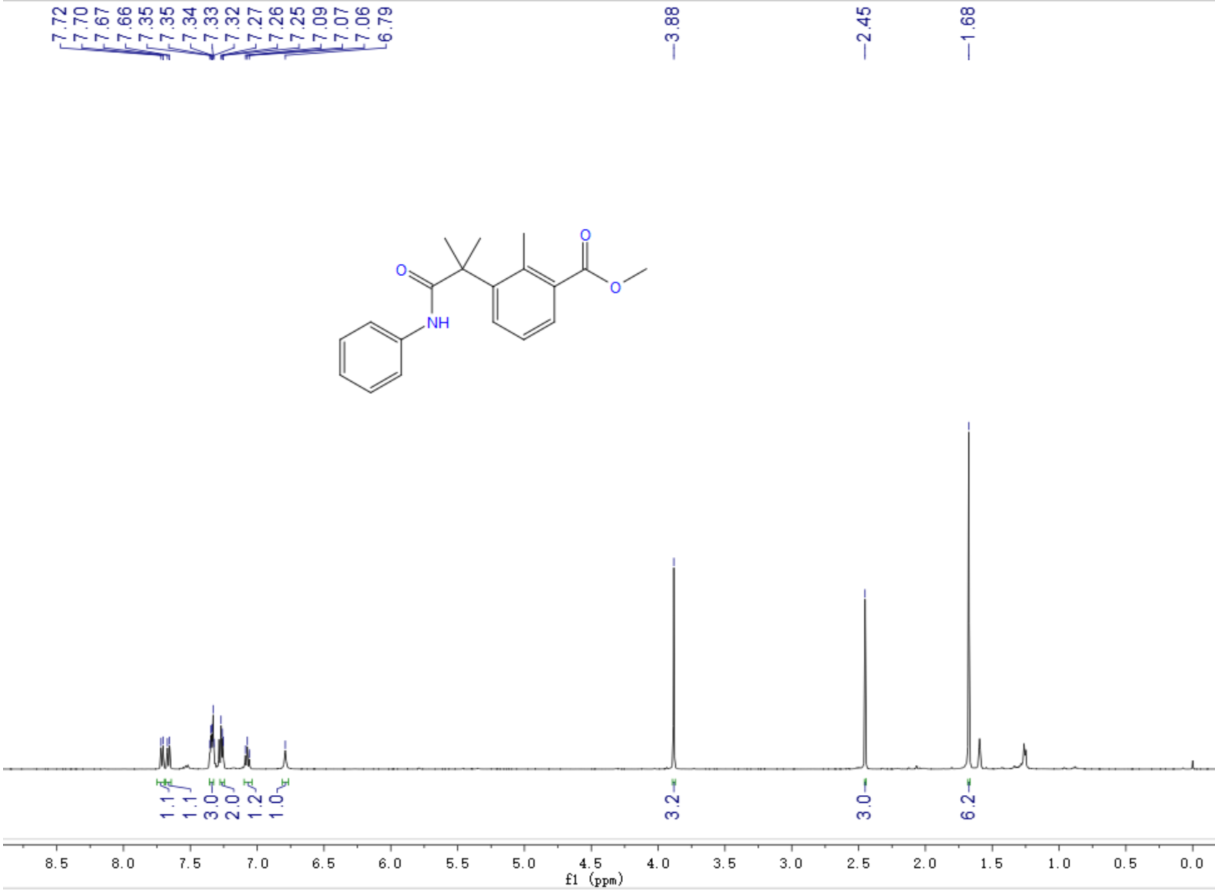

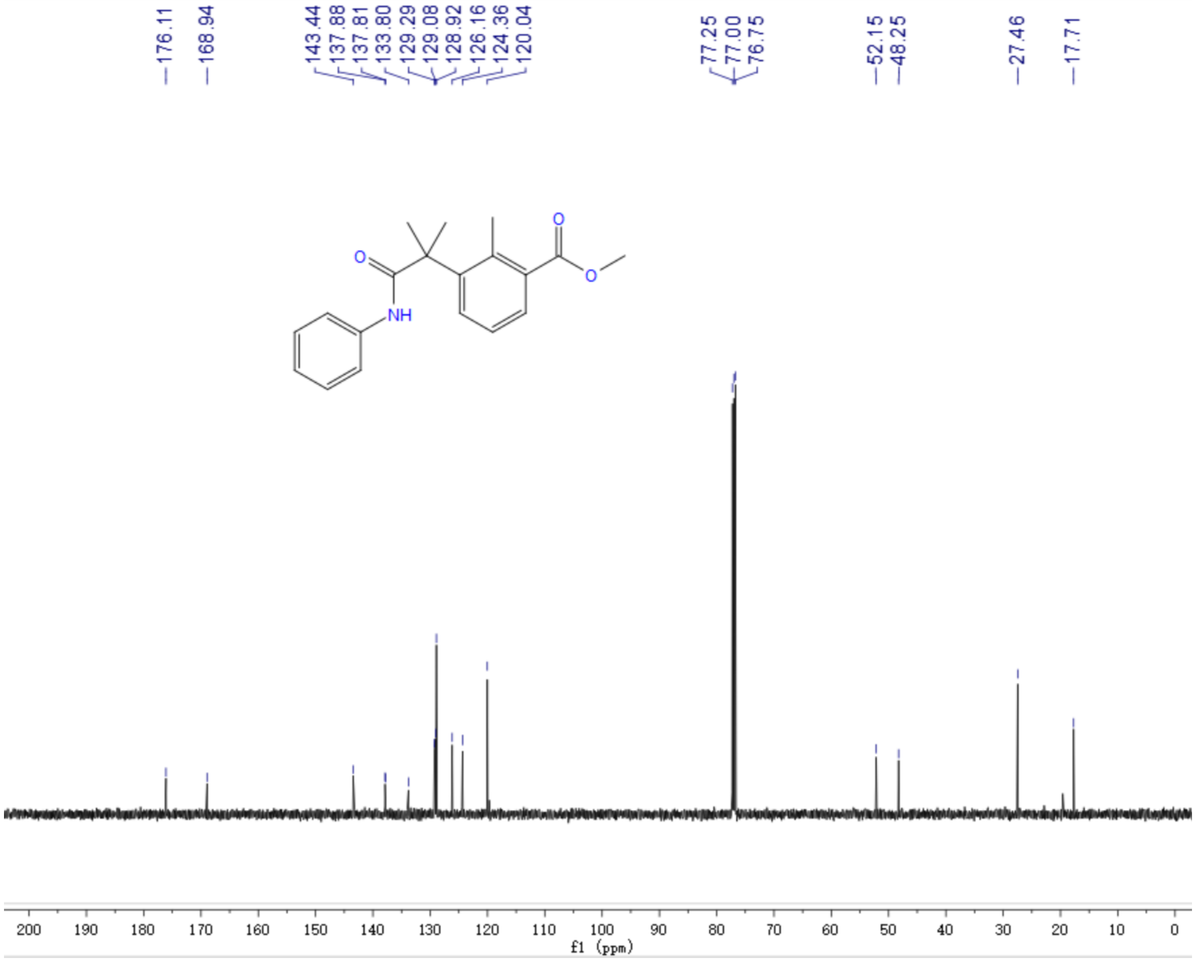

Methyl-2-ethyl-3-(2-methyl-1-oxo-1-(phenylamino)propan-2-yl)benzoate, **3ca**, ^{1}H NMR (500 MHz, $CDCl_3$) and ^{13}C NMR (125 MHz, $CDCl_3$)

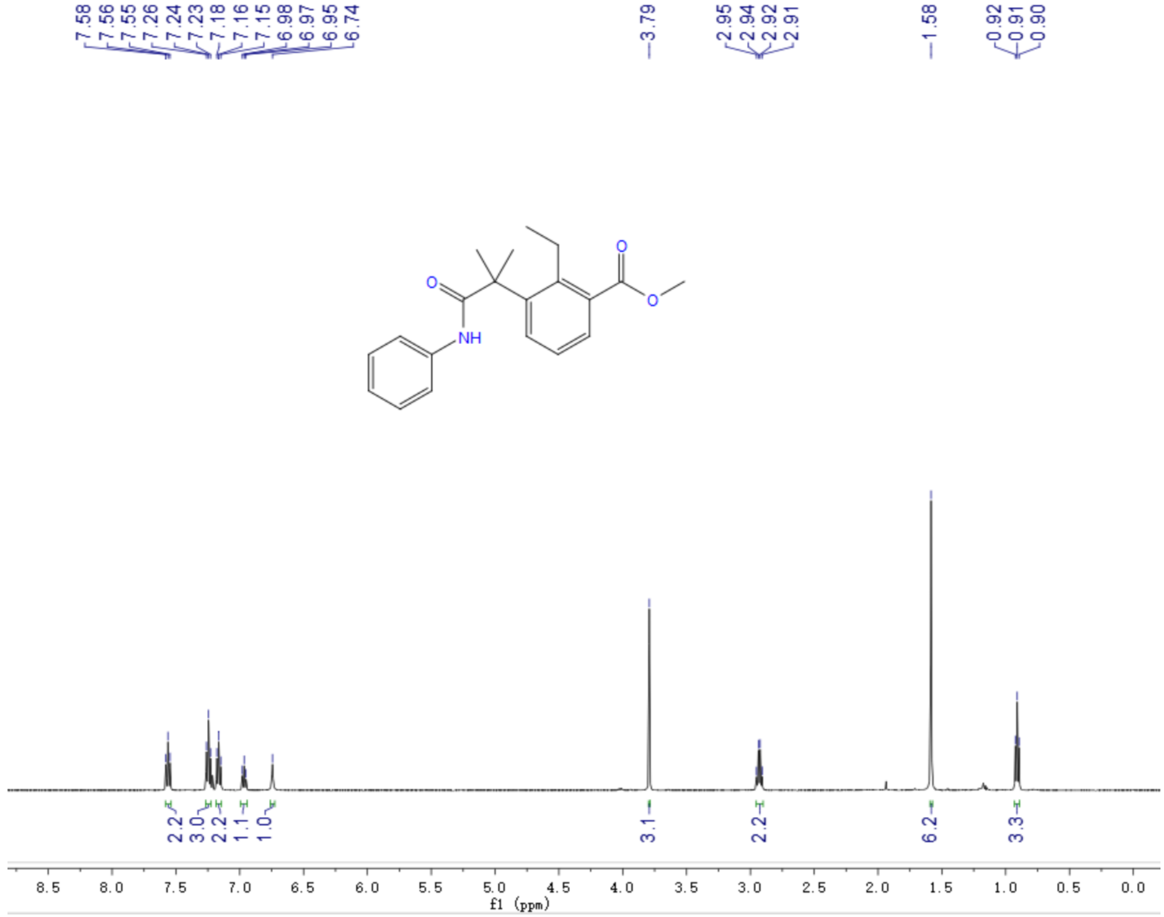

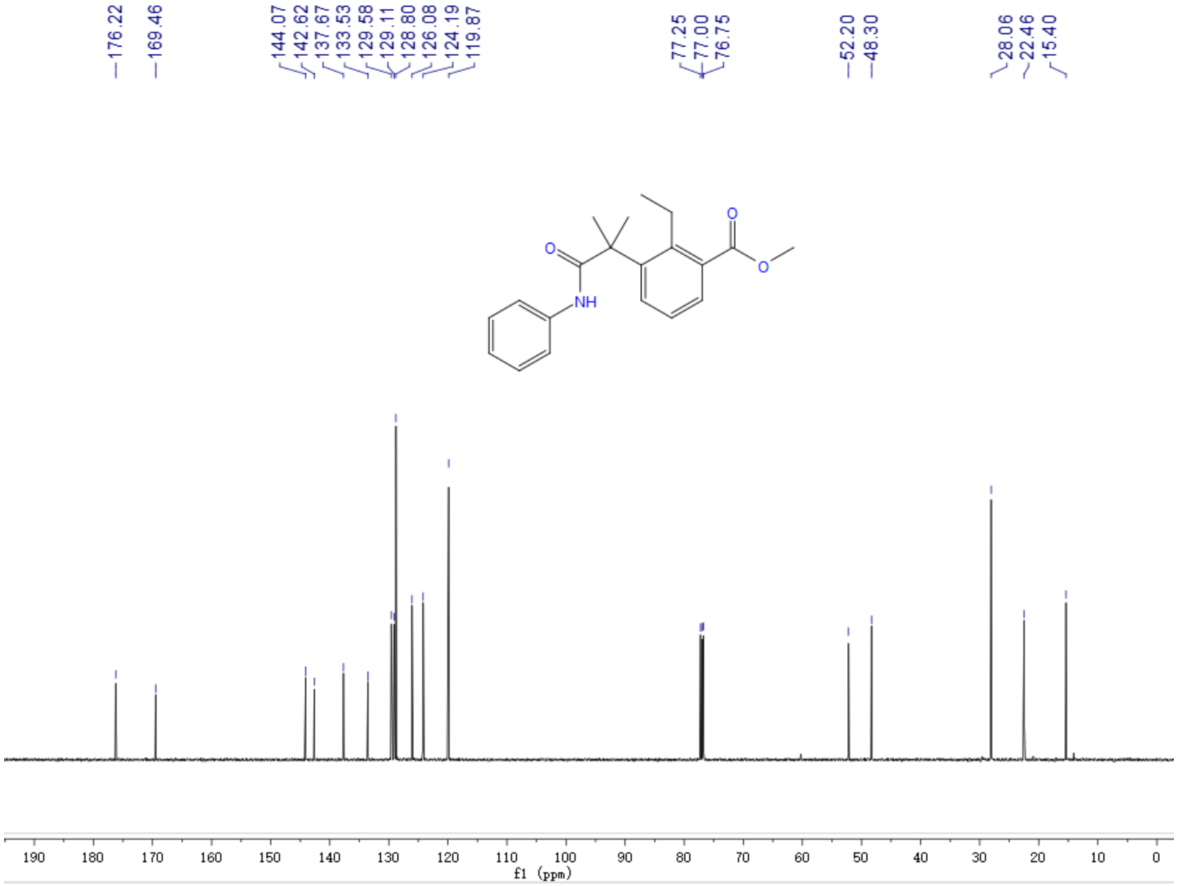

Methyl-2-methoxy-3-(2-methyl-1-oxo-1-(phenylamino)propan-2-yl)benzoate, **3da**, ^1H NMR (400 MHz, $CDCl_3$) and ^{13}C NMR (100 MHz, $CDCl_3$)

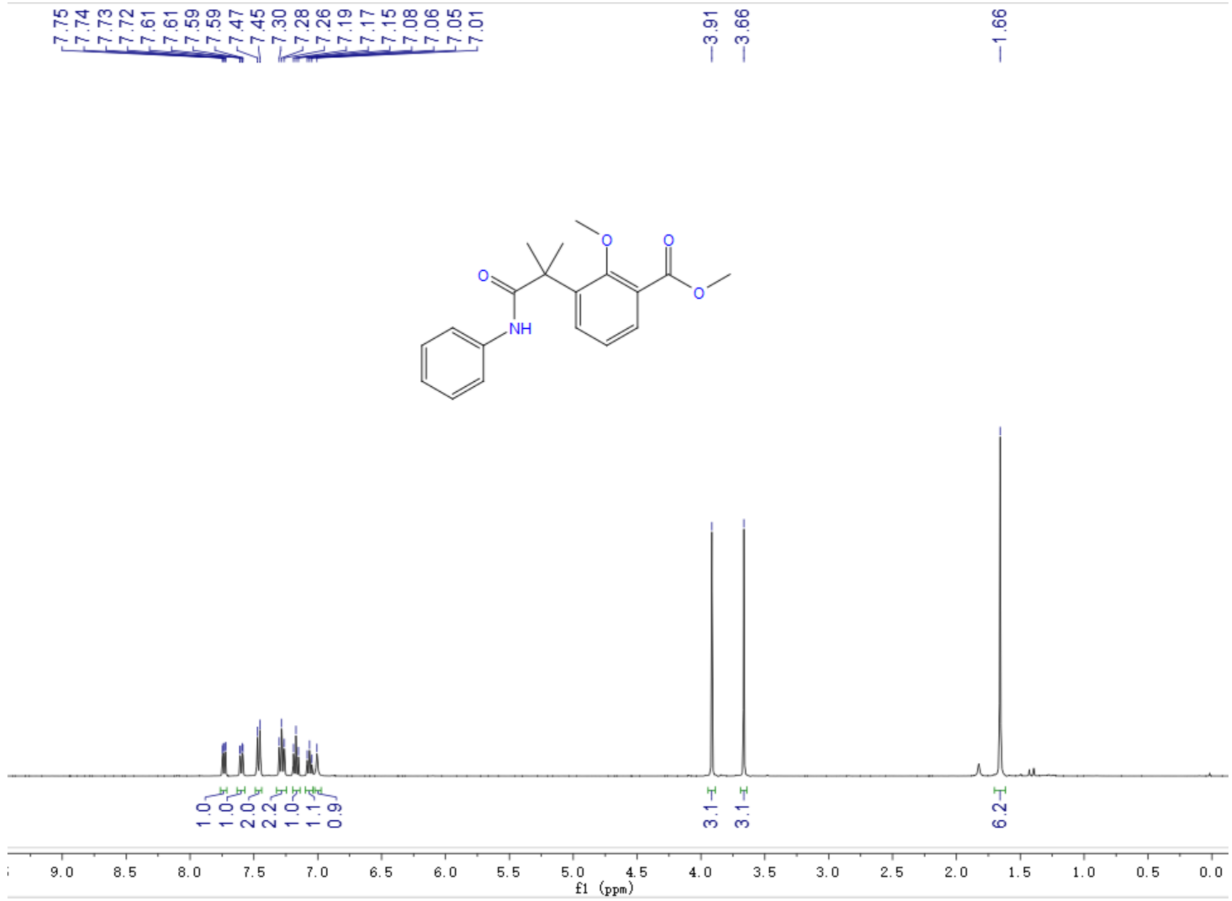

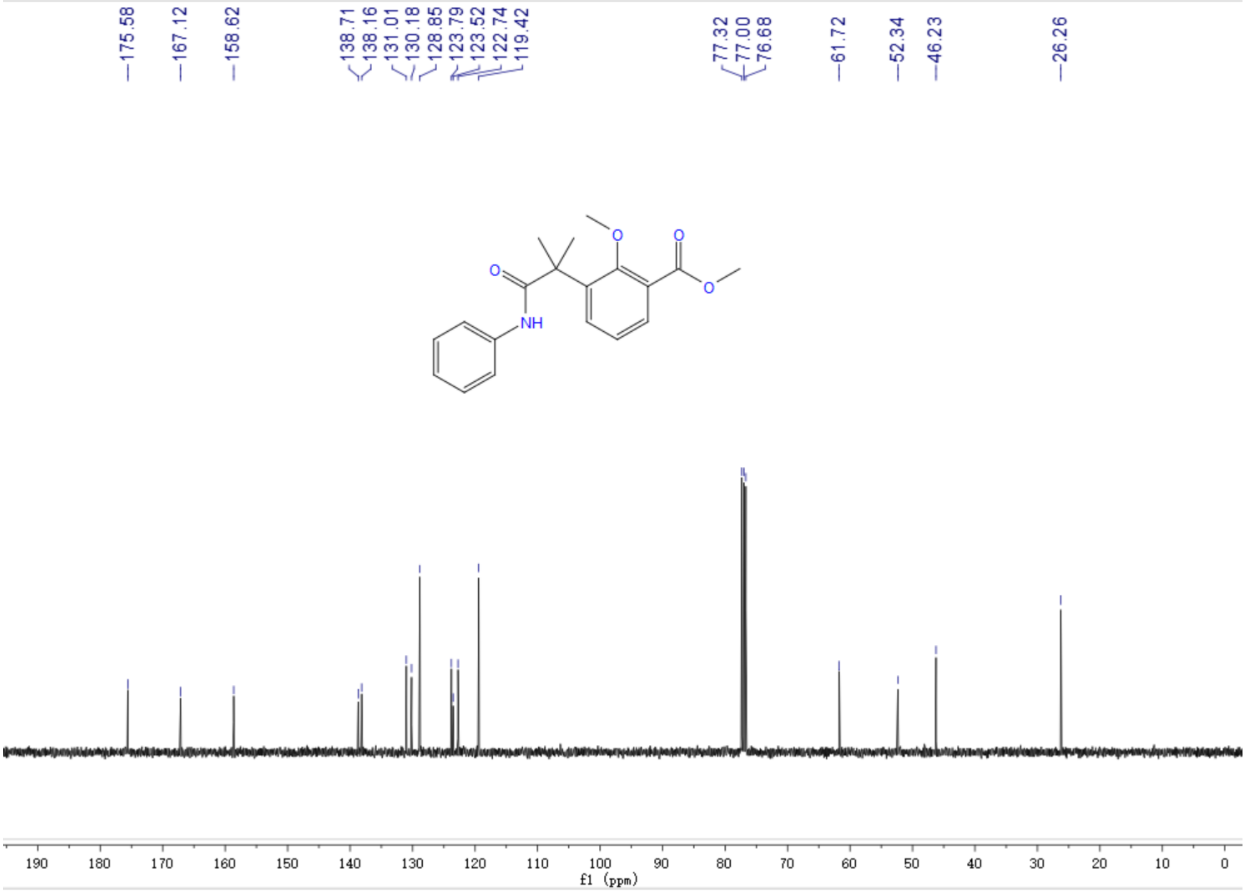

Methyl-2-fluoro-3-(2-methyl-1-oxo-1-(phenylamino)propan-2-yl)benzoate, **3ea**, ^{1}H NMR (500 MHz, $CDCl_3$) and ^{13}C NMR (125 MHz, $CDCl_3$)

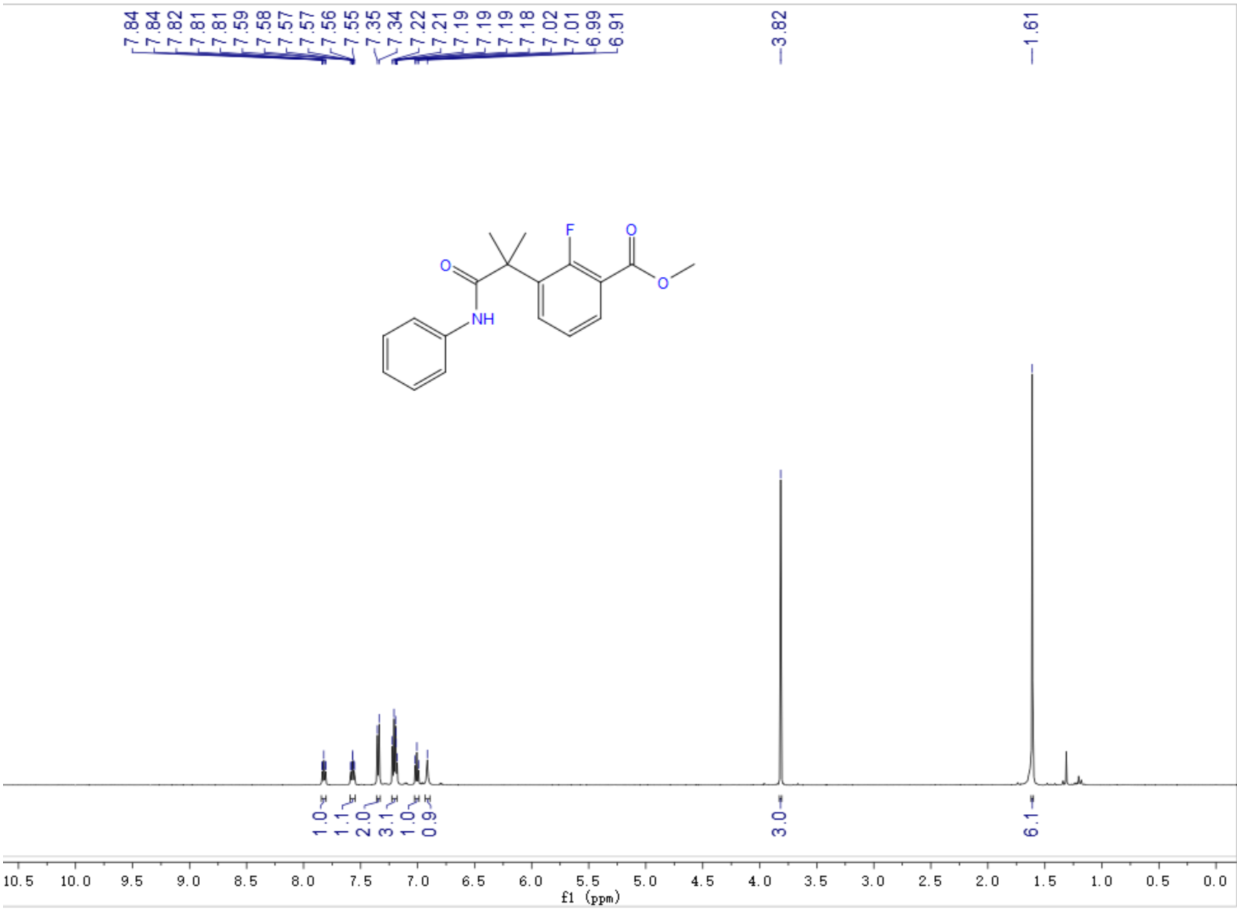

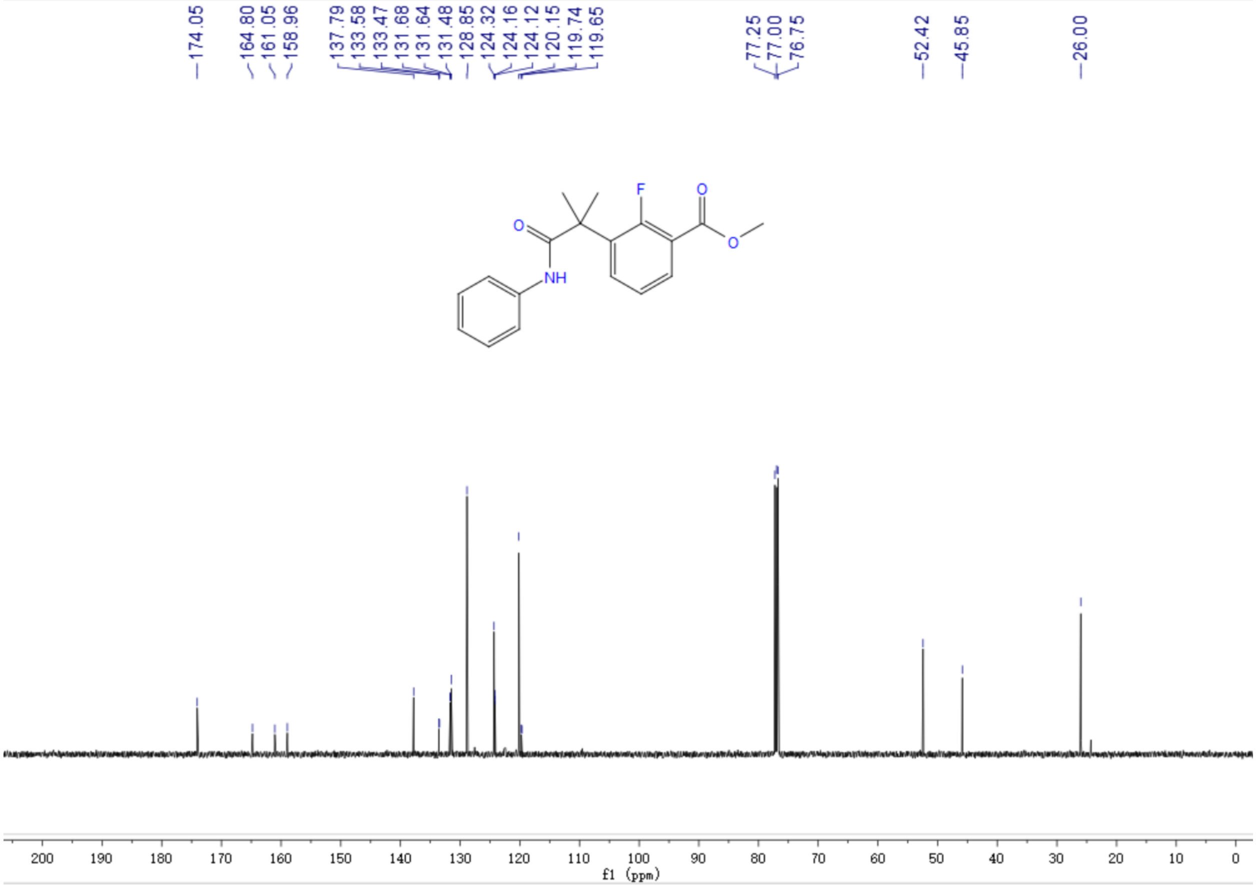

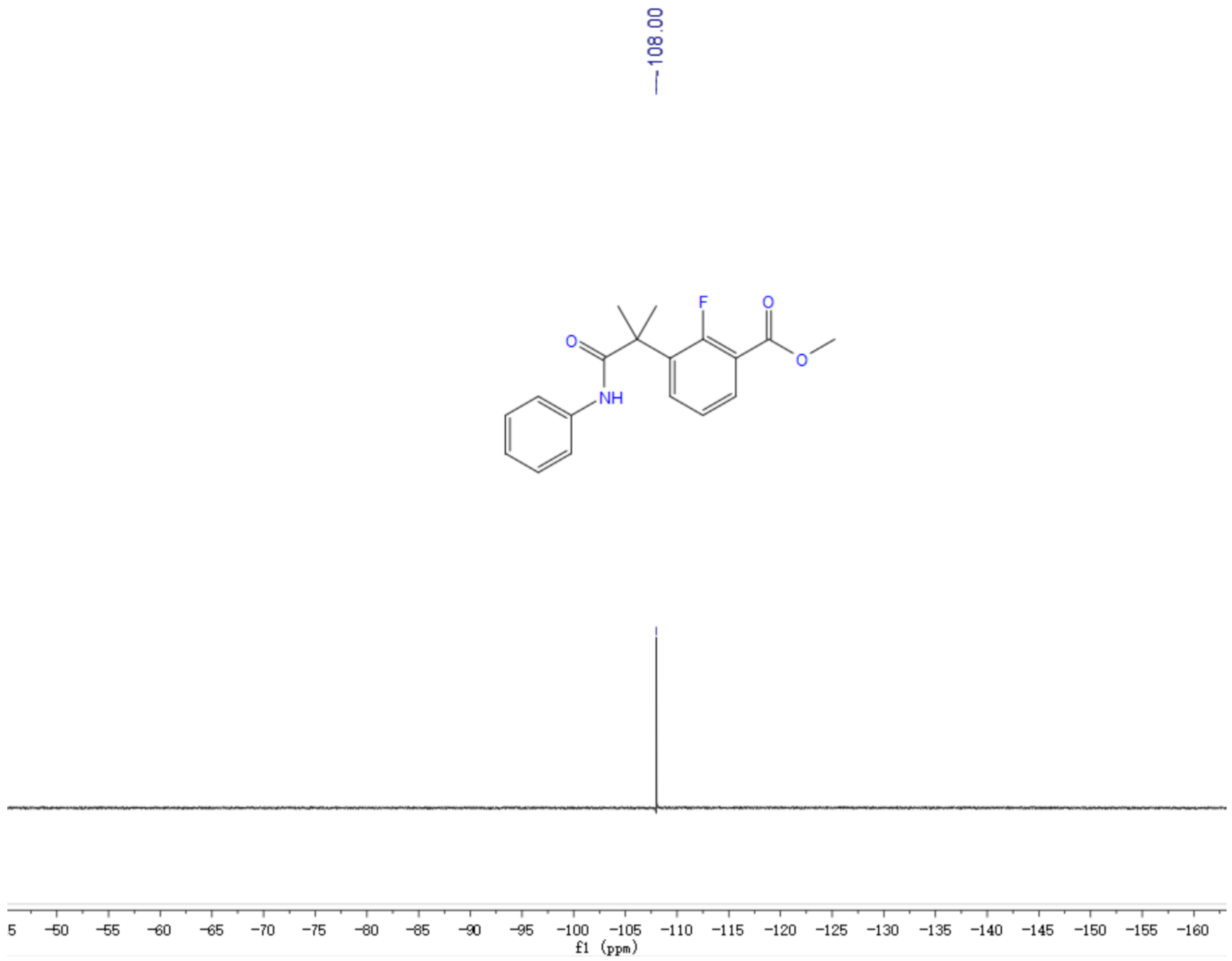
-108.00
F
O
O
O
NH
5 -50 -55 -60 -65 -70 -75 -80 -85 -90 -95 -100 -105 -110 -115 -120 -125 -130 -135 -140 -145 -150 -155 -160
f1 (ppm)

2-Chloro-3-(2-methyl-1-oxo-1-(phenylamino)propan-2-yl)benzoic acid, **3fa**, ^{1}H NMR (500 MHz, DMSO-d$_6$) and ^{13}C NMR (125 MHz, DMSO-d$_6$)

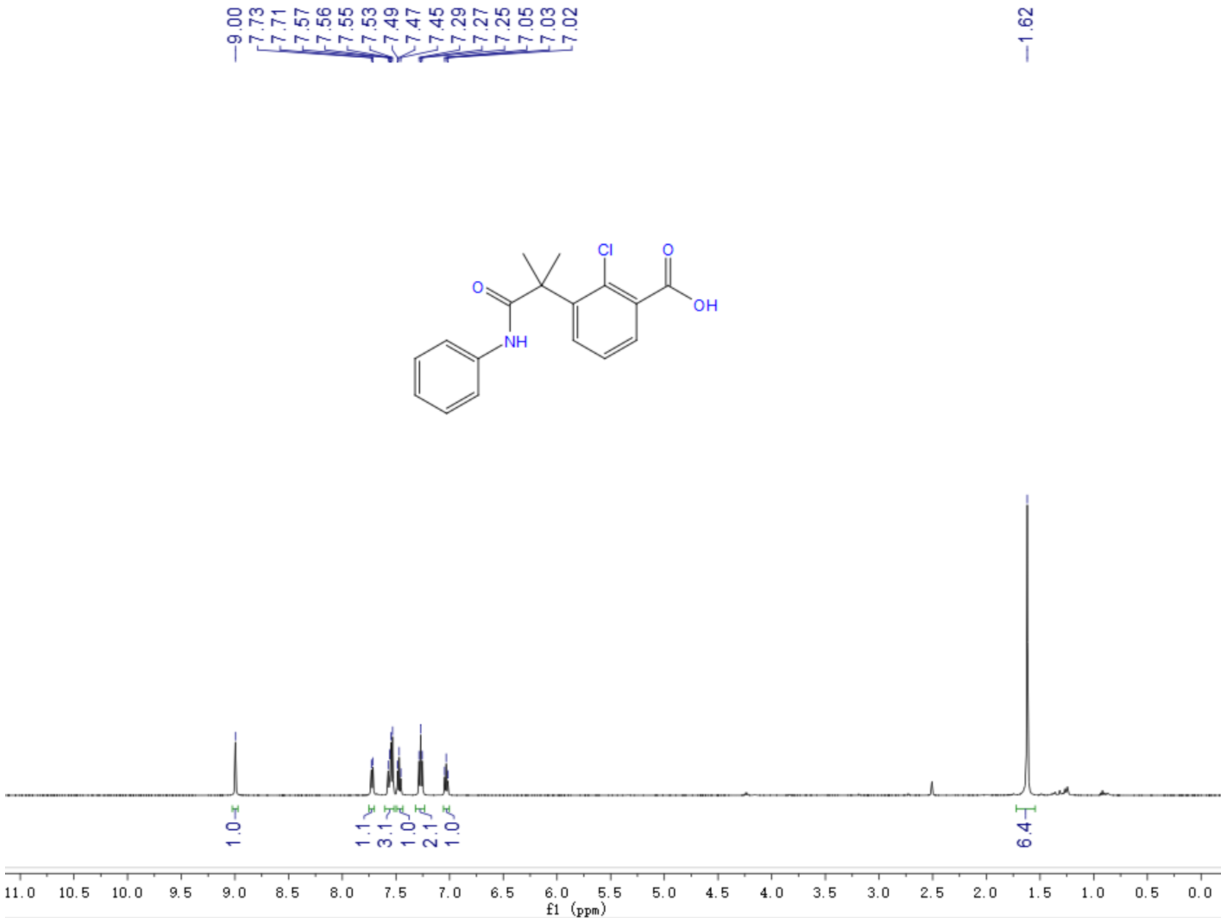

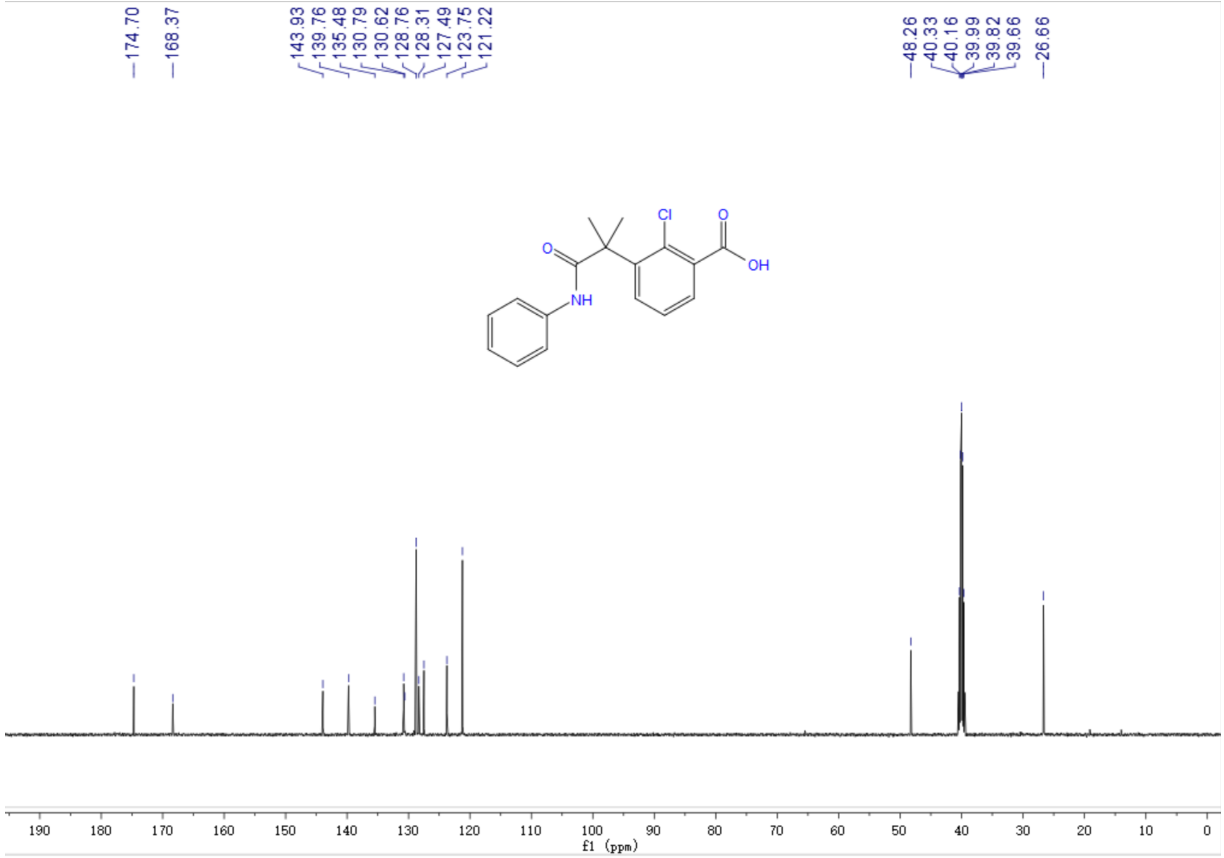

Methyl-3-methoxy-5-(2-methyl-1-oxo-1-(phenylamino)propan-2-yl)benzoate, **3ga**, ^{1}H NMR (500 MHz, $CDCl_3$) and ^{3}C NMR (125 MHz, $CDCl_3$)

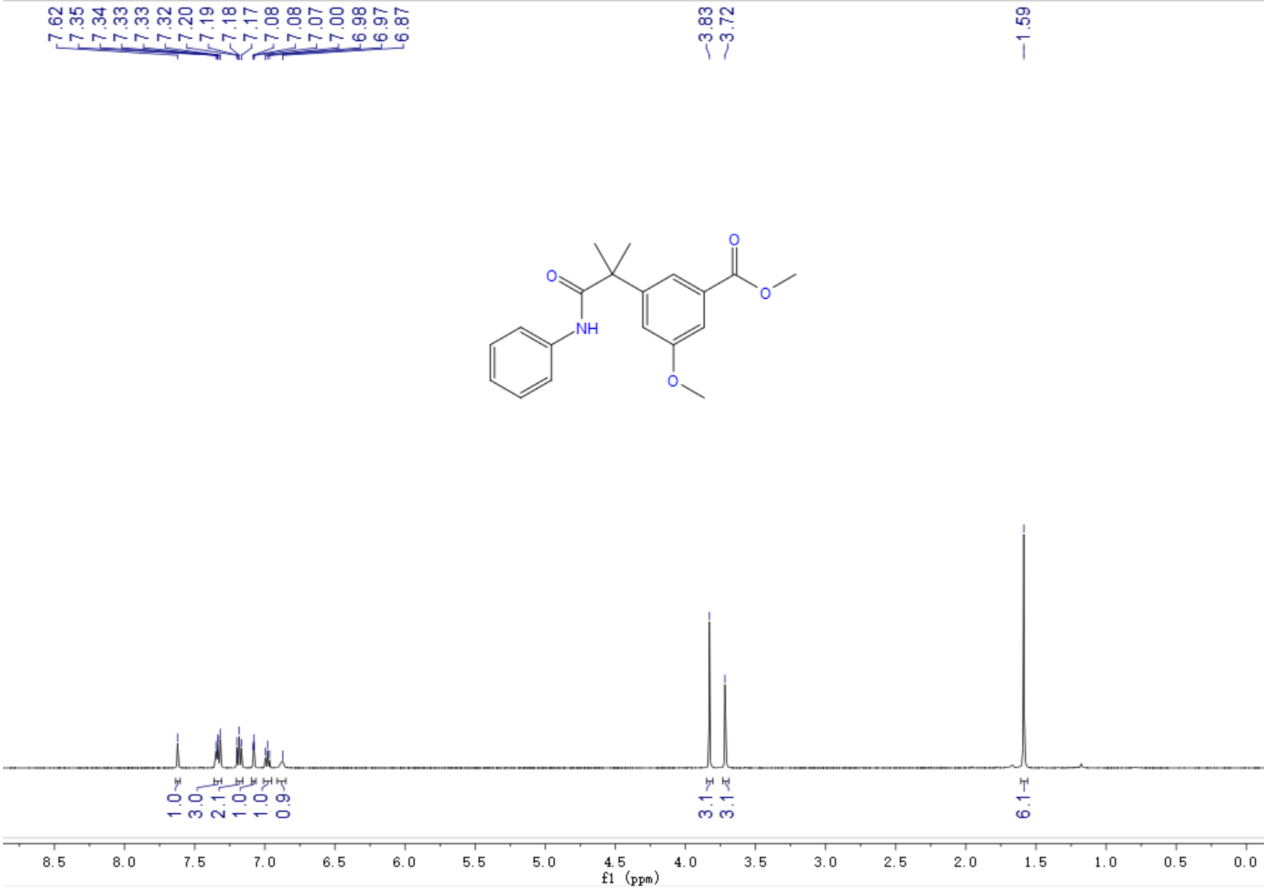

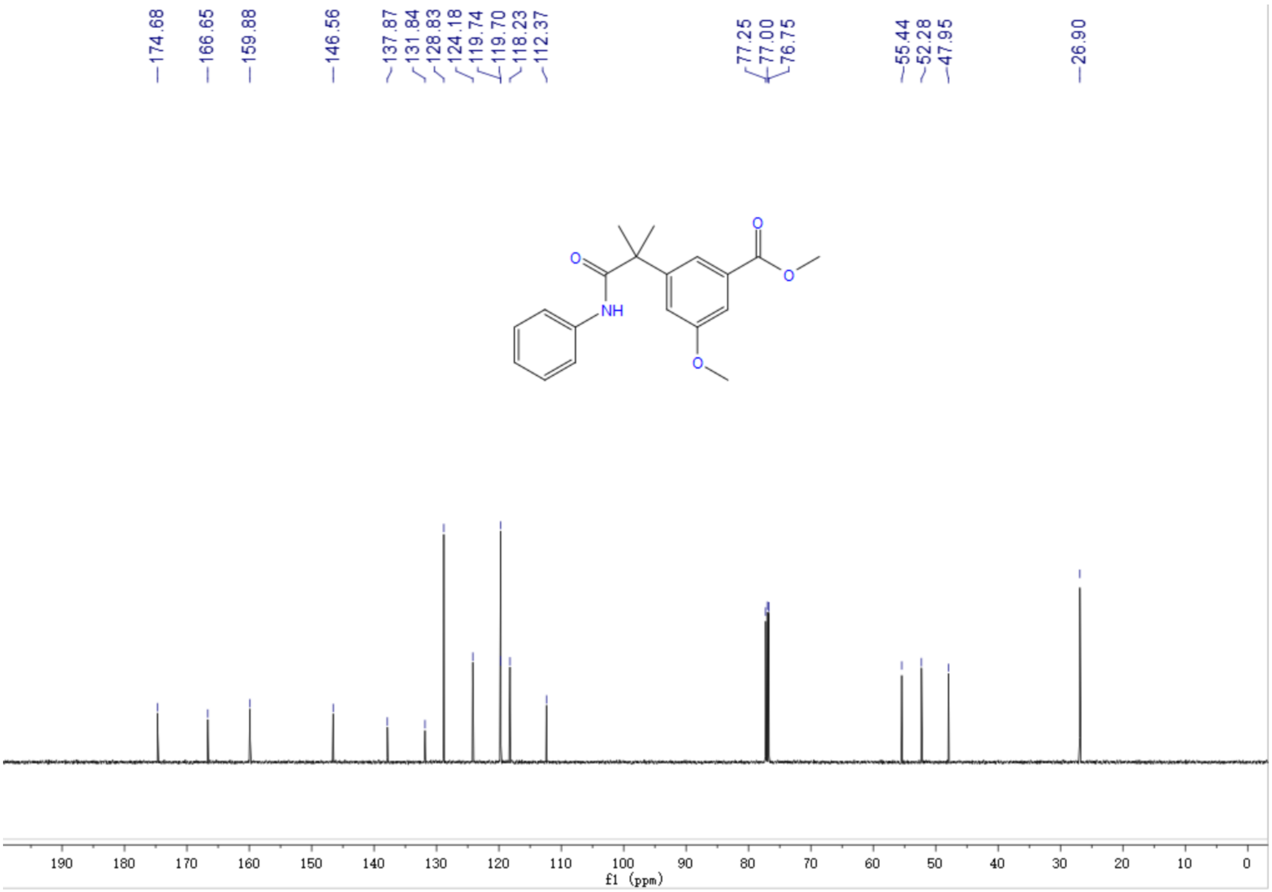

Methyl-3-fluoro-5-(2-methyl-1-oxo-1-(phenylamino)propan-2-yl)benzoate, **3ha**, ^{1}H NMR (500 MHz, $CDCl_3$), ^{13}C NMR (125 MHz, $CDCl_3$) and ^{19}F NMR (471 MHz, $CDCl_3$)

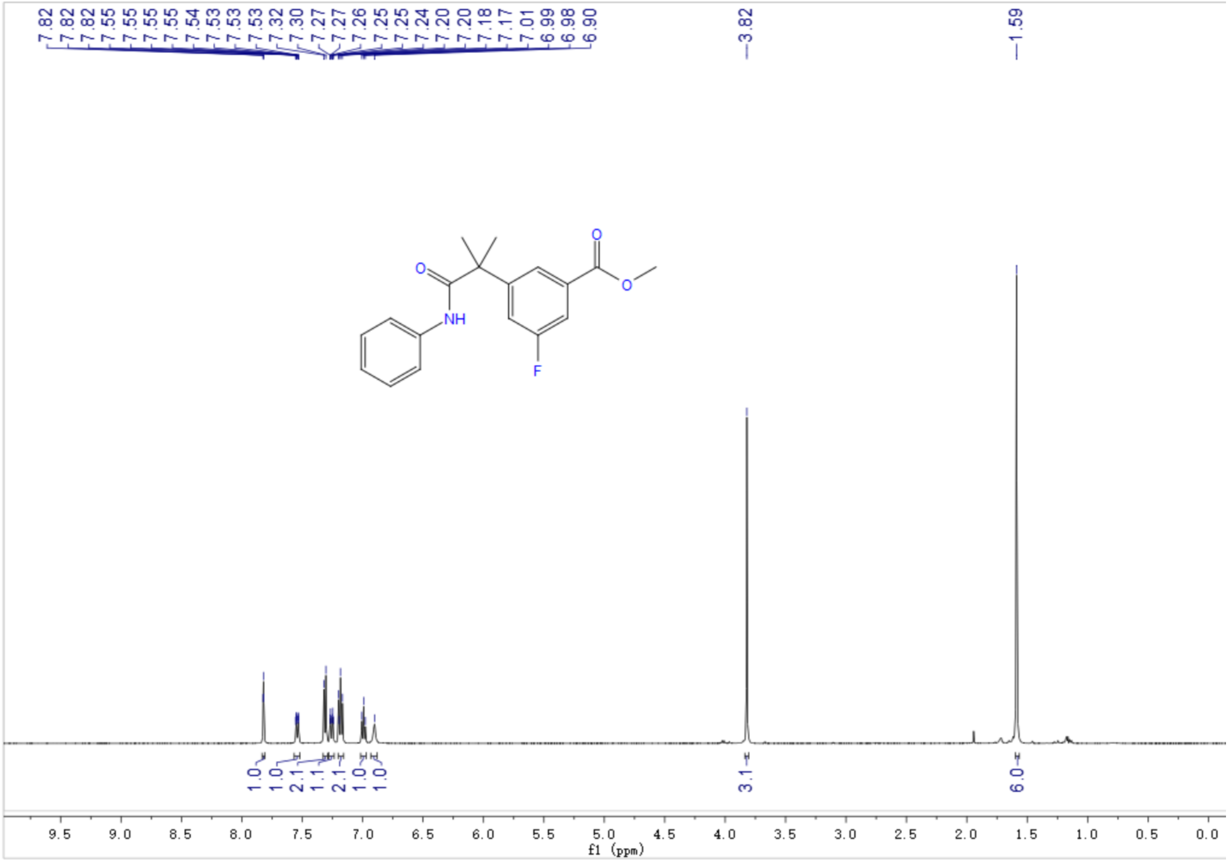

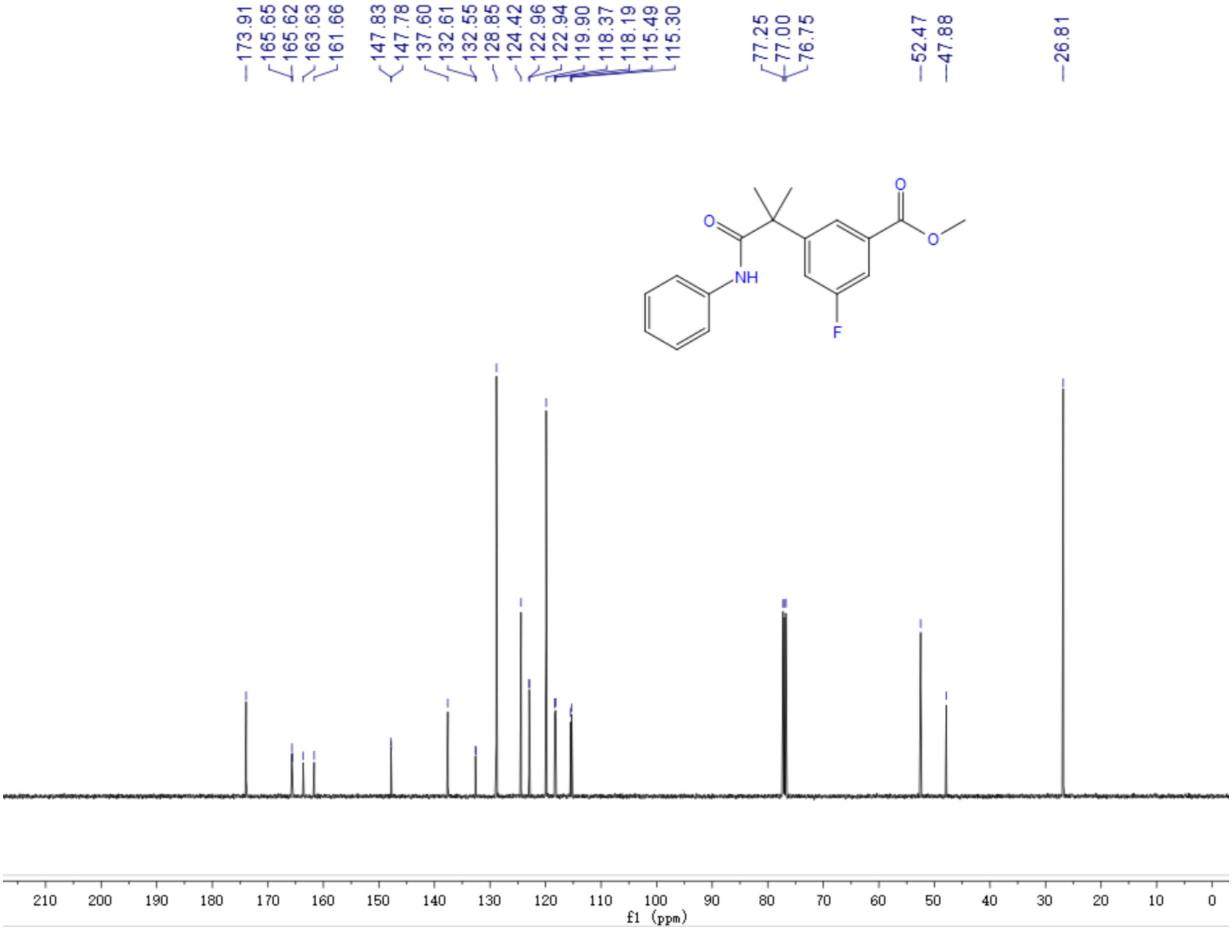

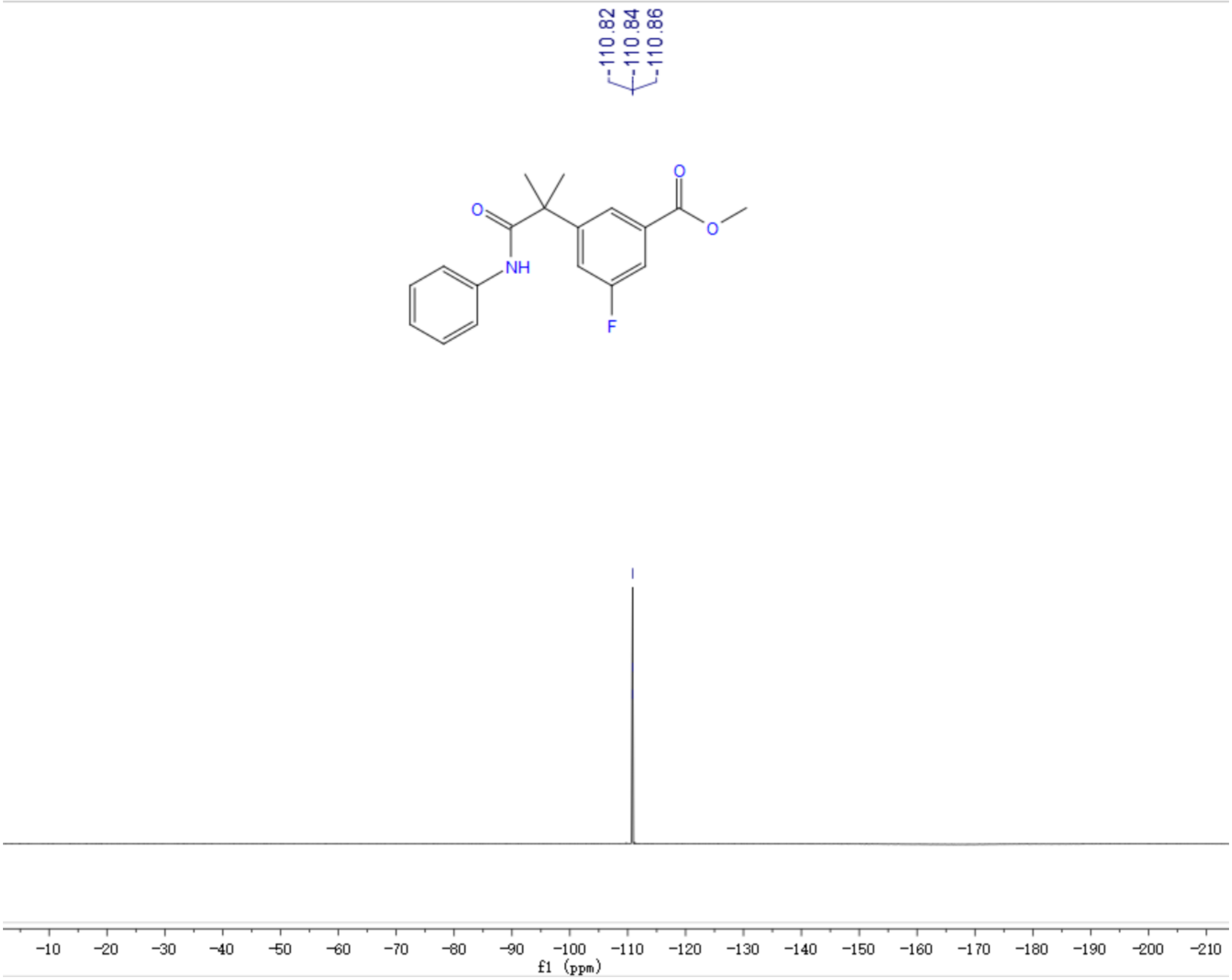

-110.82
-110.84
-110.86
O
NH
O
O
F
−10 −20 −30 −40 −50 −60 −70 −80 −90 −100 −110 −120 −130 −140 −150 −160 −170 −180 −190 −200 −210
f1 (ppm)

3-(2-Methyl-1-oxo-1-(phenylamino)propan-2-yl)-5-((2-methylallyl)oxy)benzoic acid, **3ia**, ^{1}H NMR (400 MHz, DMSO-d_6) and ^{13}C NMR (125 MHz, DMSO-d_6)

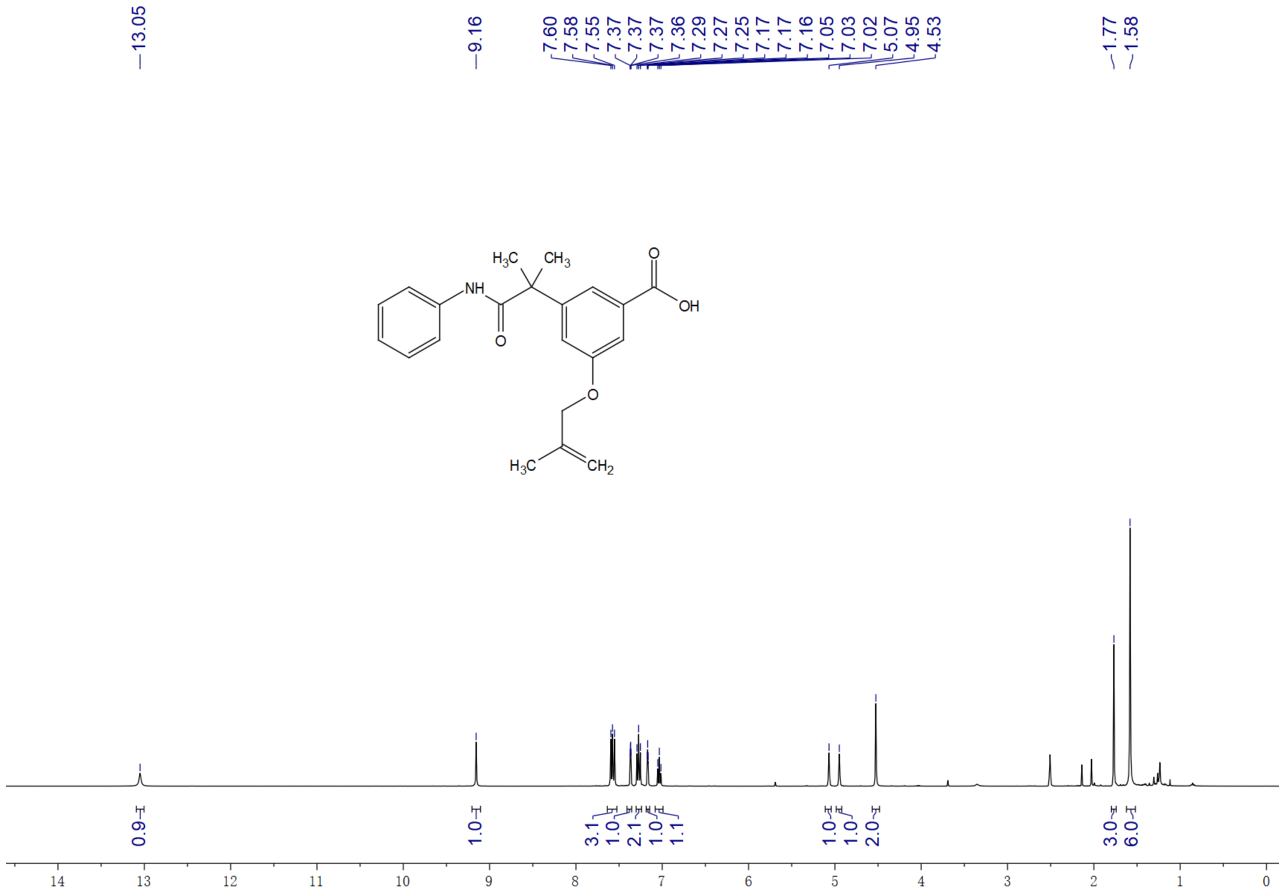

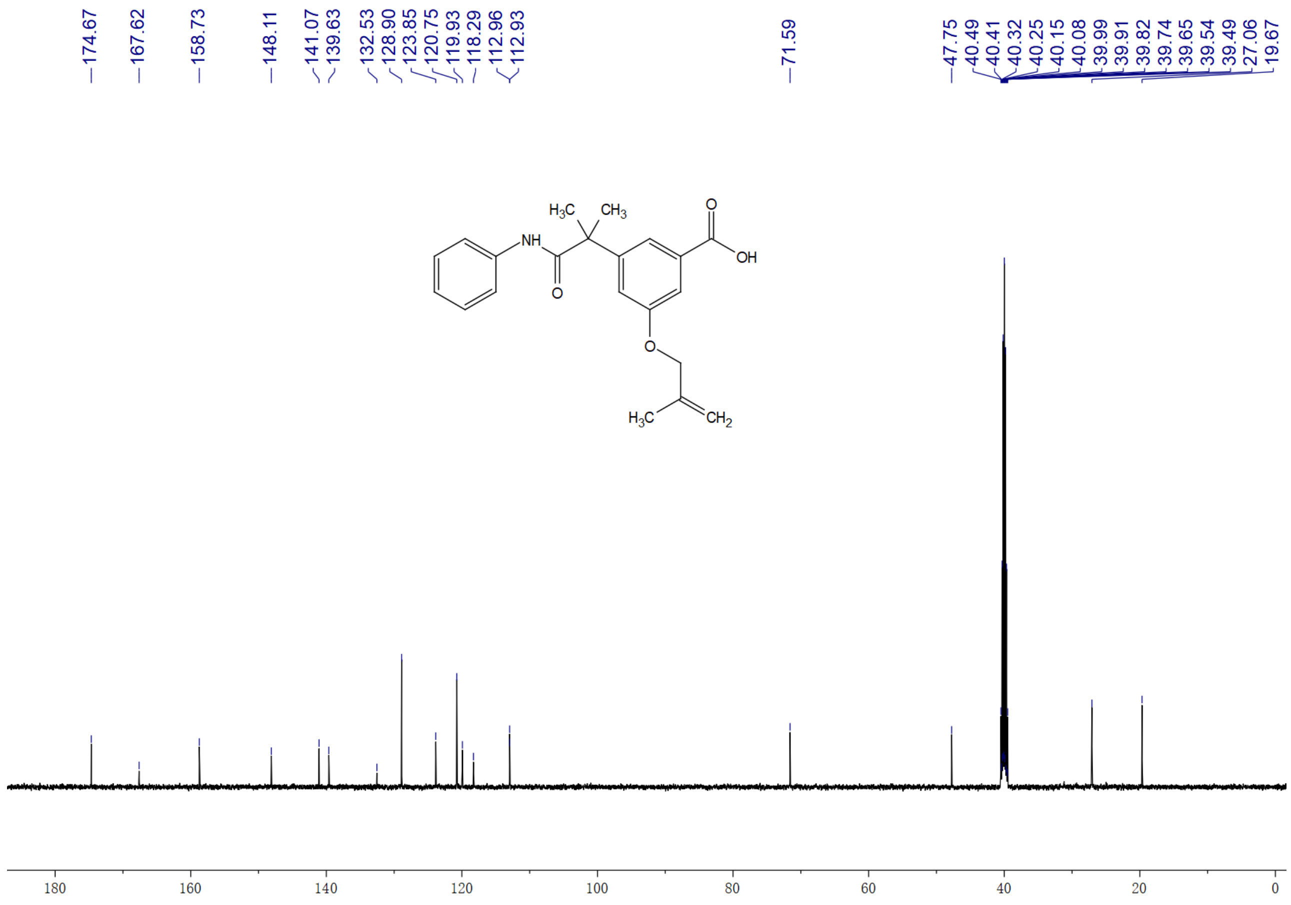

4-(Difluoromethoxy)-3-(2-methyl-1-oxo-1-(phenylamino)propan-2-yl)benzoic acid, **3ja**, ^{1}H NMR (500 MHz, DMSO-d_6) and ^{13}C NMR (125 MHz, DMSO-d_6)

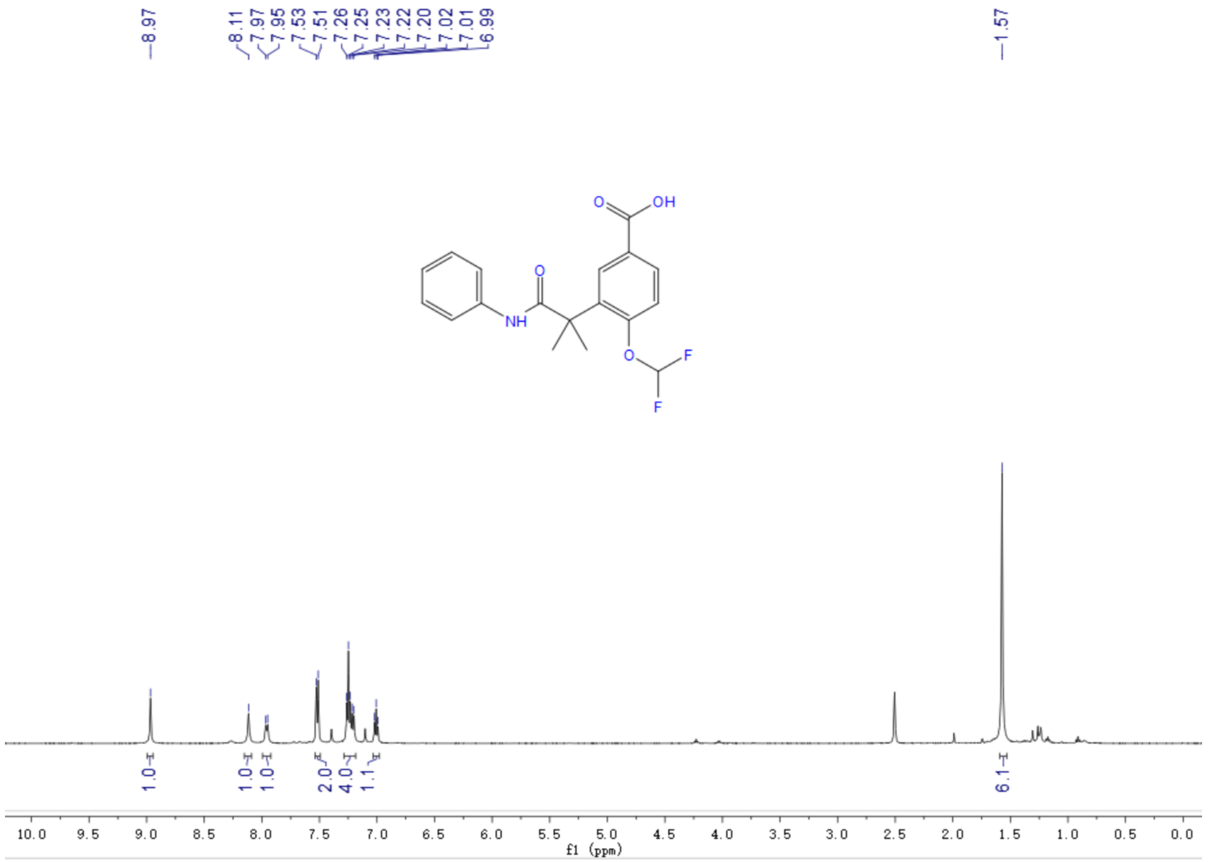

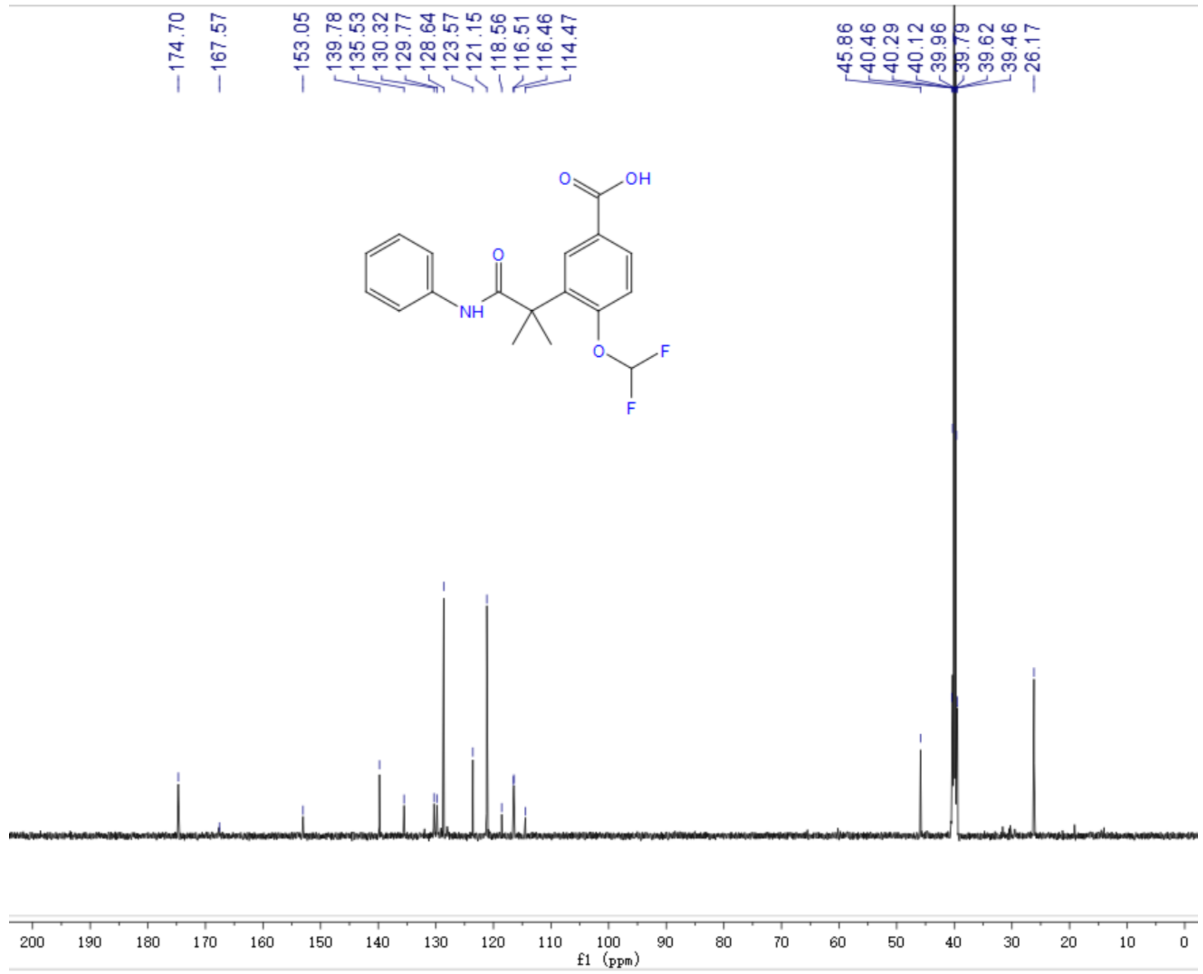

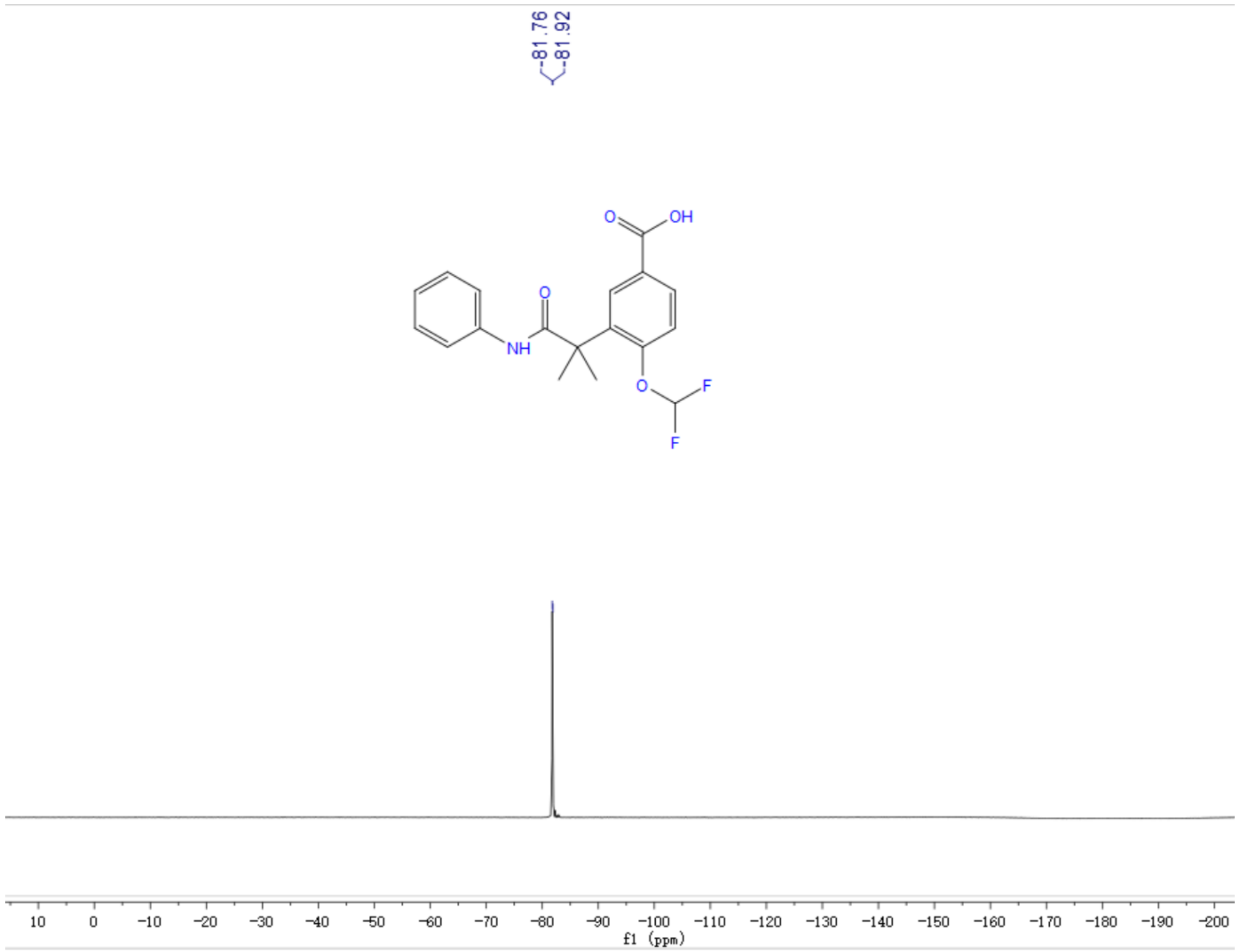
-81.76
-81.92
O
OH
O
NH
O
F
F
10 0 −10 −20 −30 −40 −50 −60 −70 −80 −90 −100 −110 −120 −130 −140 −150 −160 −170 −180 −190 −200
f1 (ppm)

3-(2-Methyl-1-oxo-1-(phenylamino)propan-2-yl)-4-(trifluoromethoxy)benzoic acid, **3ka**, ^{1}H NMR (500 MHz, DMSO-d_6) and ^{13}C NMR (125 MHz, DMSO-d_6)

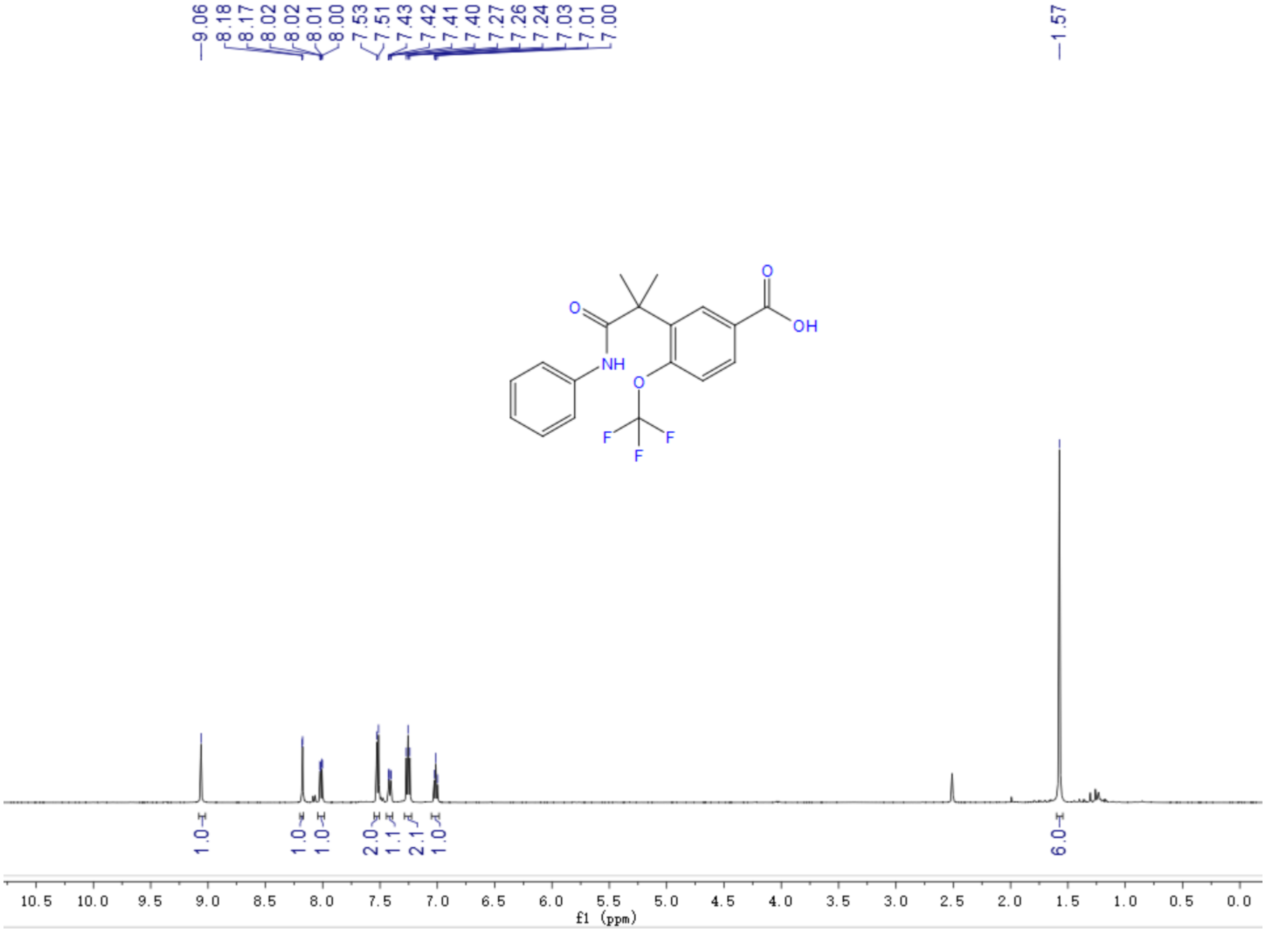

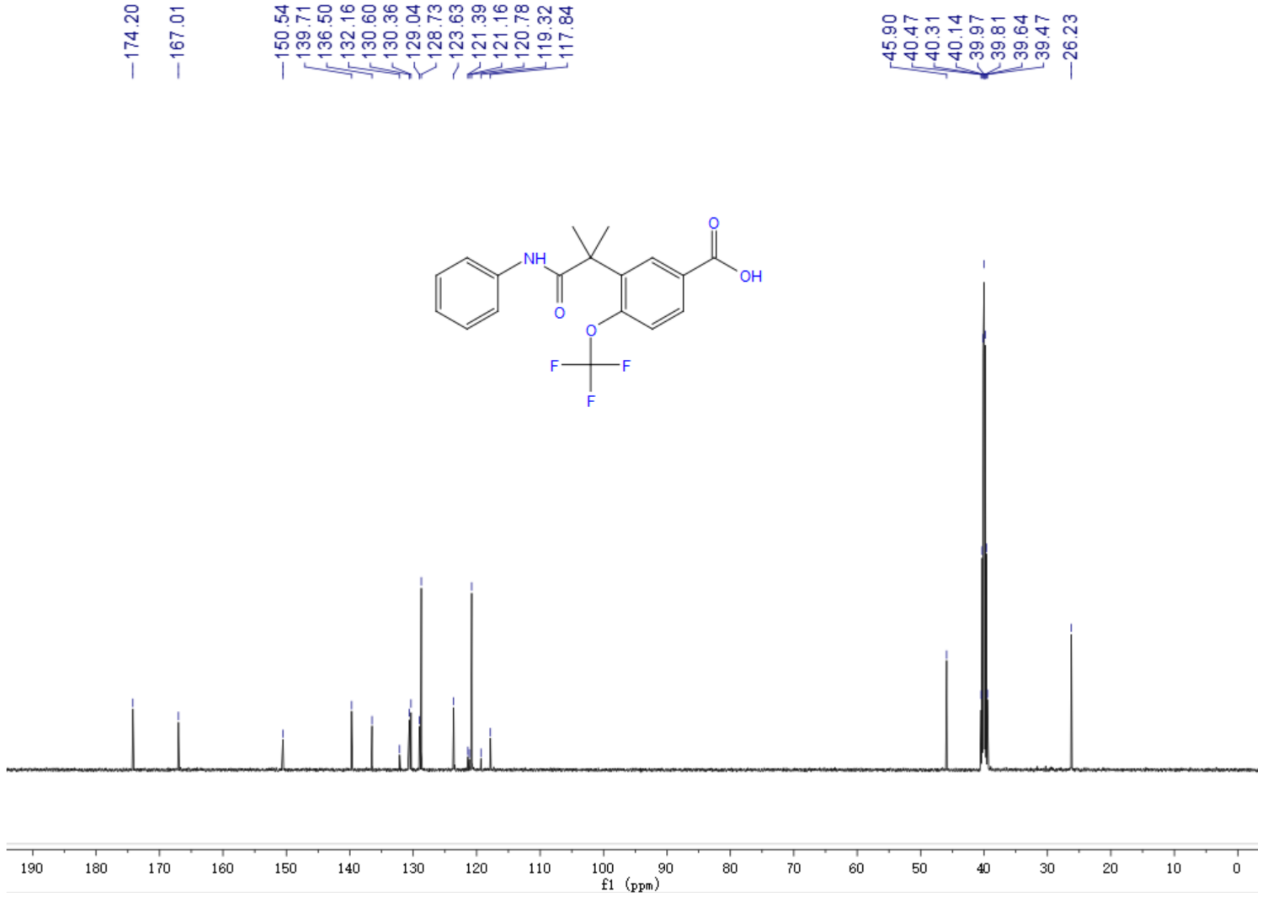

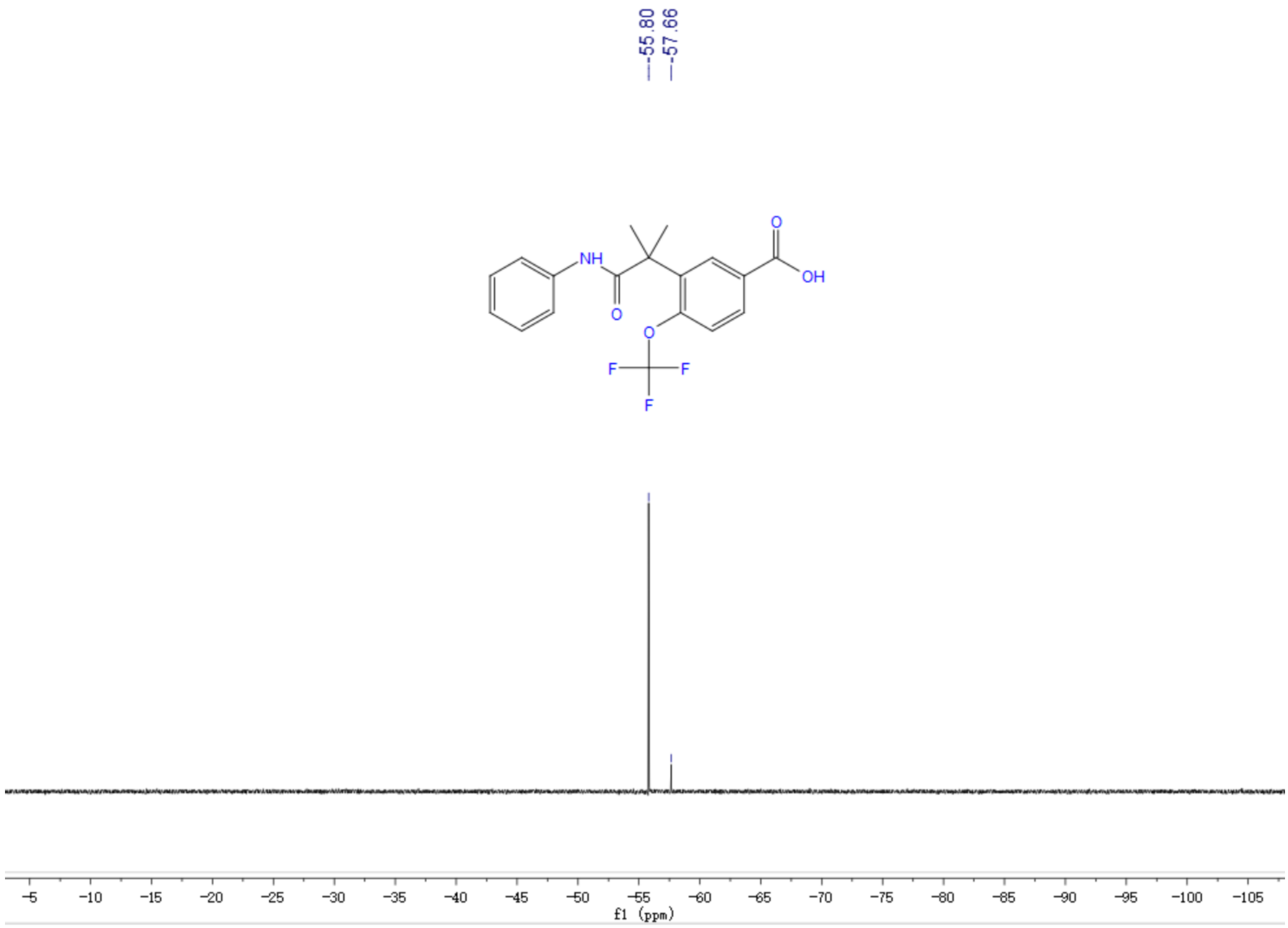

-55.80
-57.66
NH
O
O
OH
O
F
F
F
-5
-10
-15
-20
-25
-30
-35
-40
-45
-50
-55
-60
-65
-70
-75
-80
-85
-90
-95
-100
-105
f1 (ppm)

Methyl-4-methoxy-3-(2-methyl-1-oxo-1-(phenylamino)propan-2-yl)benzoate, **3la**, ^{1}H NMR (500 MHz, $CDCl_3$) and ^{13}C NMR (125 MHz, $CDCl_3$)

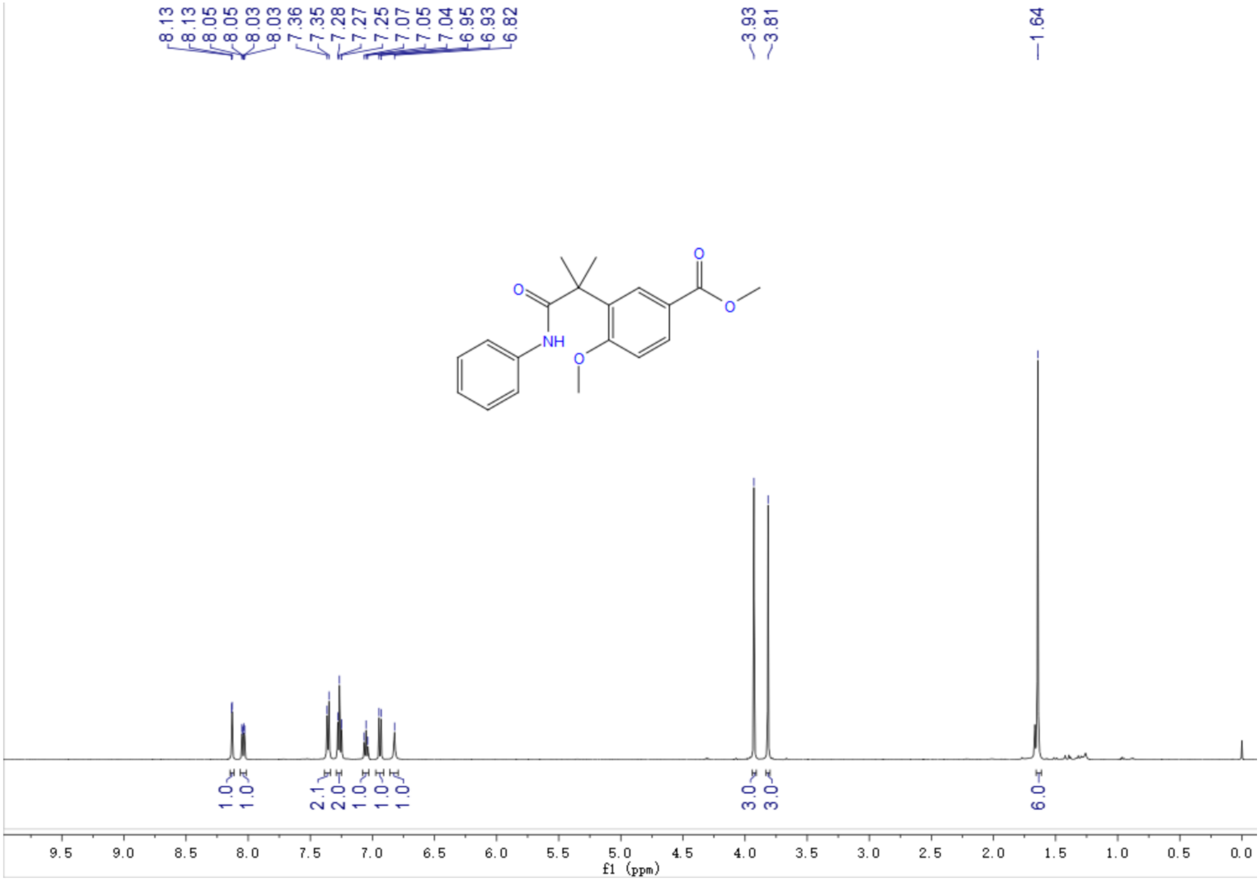

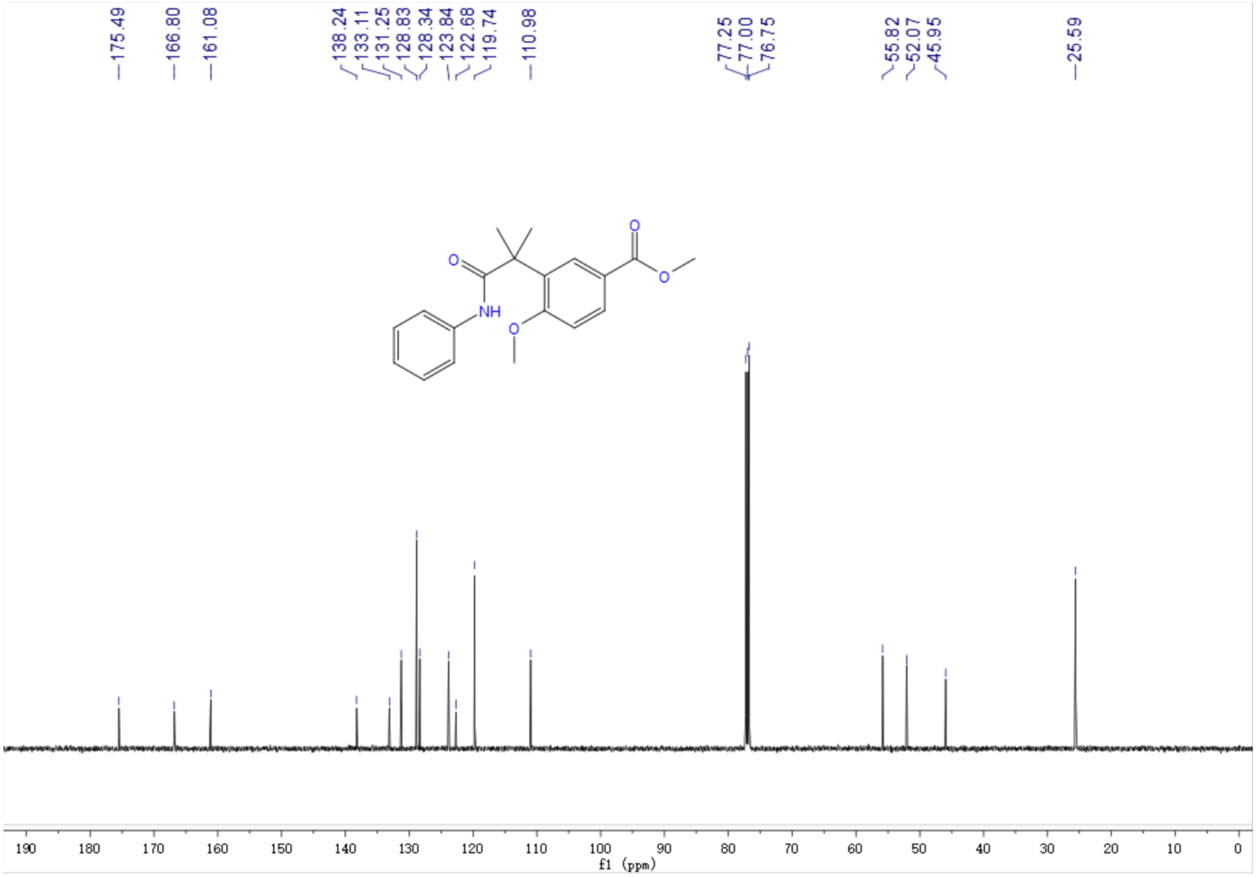

Methyl-3-(2-methyl-1-oxo-1-(phenylamino)propan-2-yl)-4-(methylthio)benzoate, **3ma**, ^{1}H NMR (500 MHz, $CDCl_3$) and ^{13}C NMR (125 MHz, $CDCl_3$)

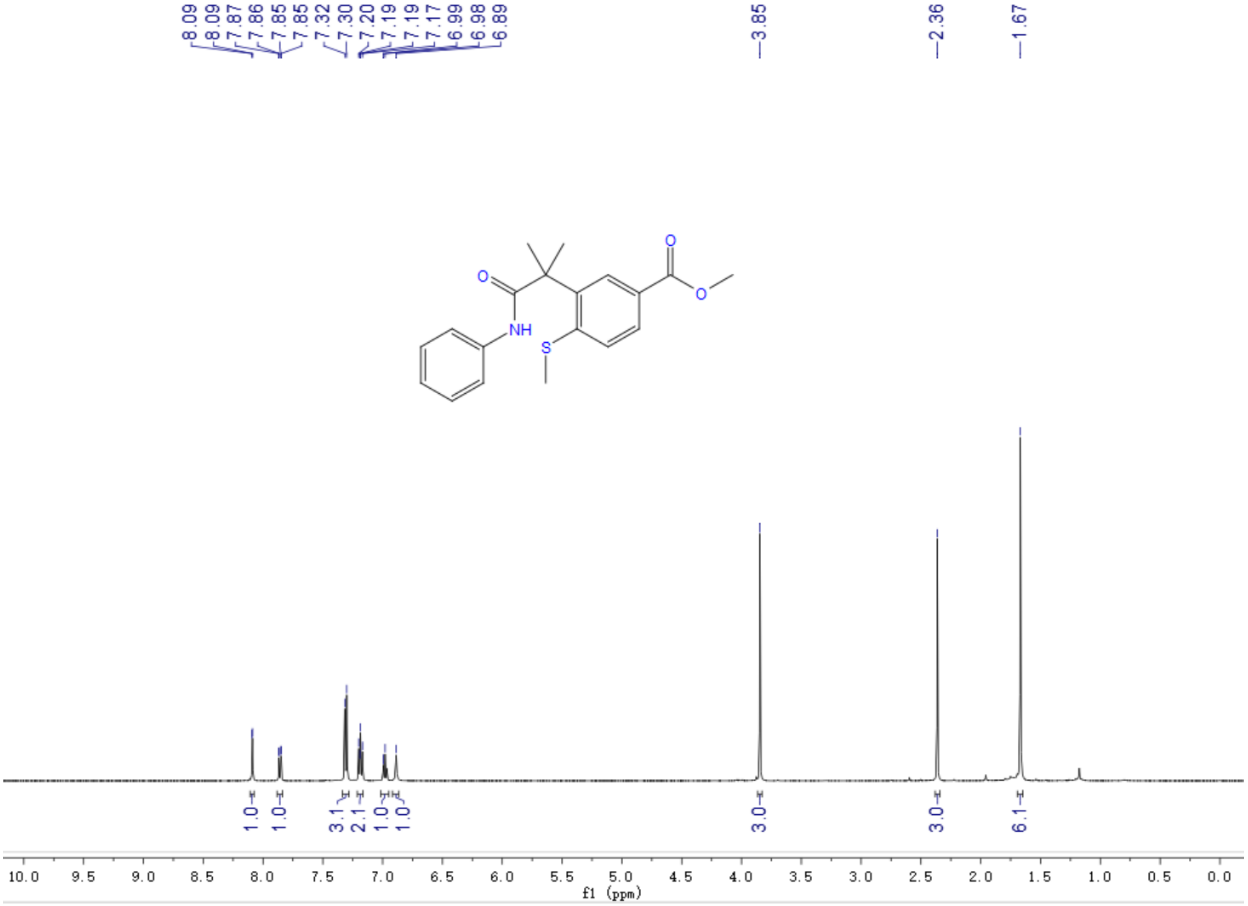

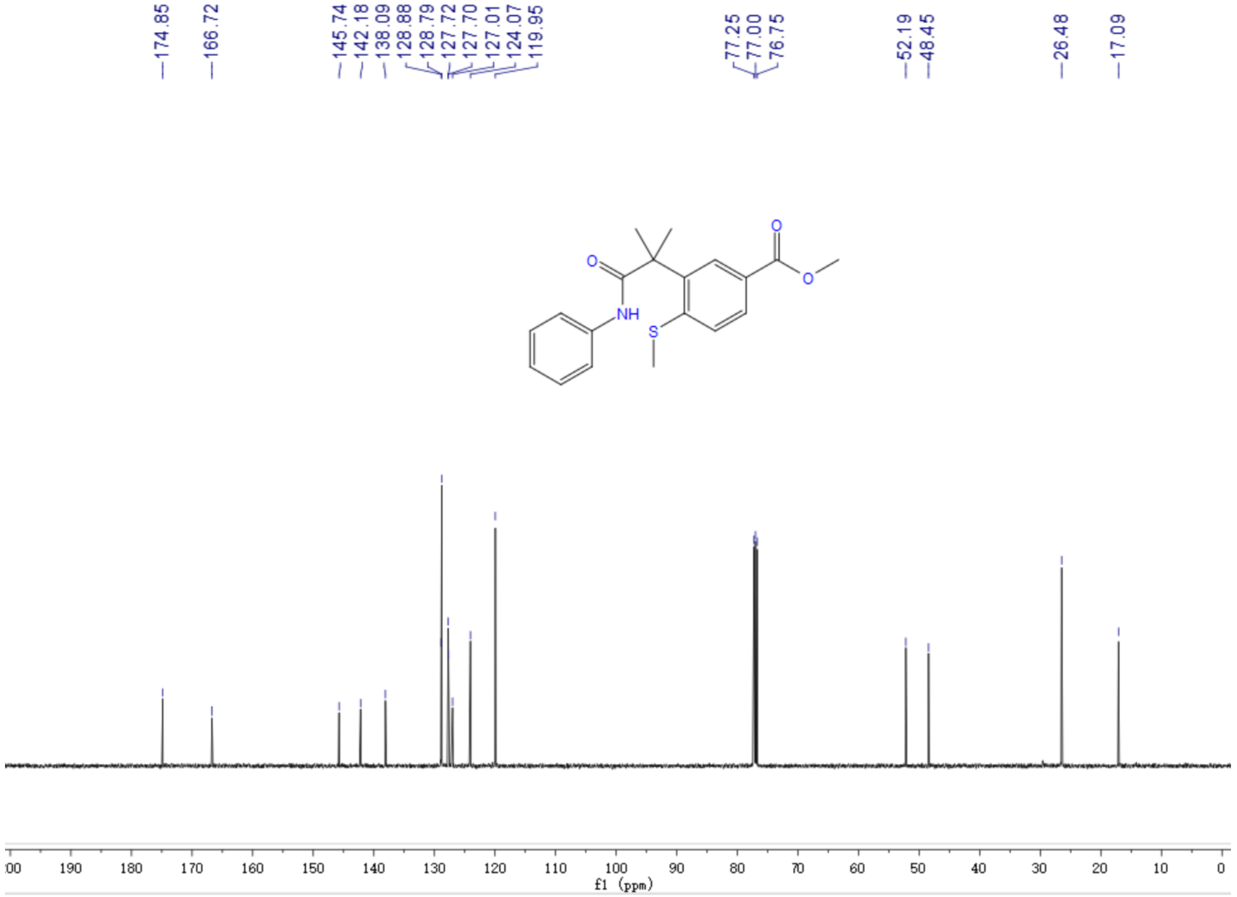

4-Fluoro-3-(2-methyl-1-oxo-1-(phenylamino)propan-2-yl)benzoic acid, **3na**, ^{1}H NMR (500 MHz, DMSO-d_6), ^{13}C NMR (125 MHz, DMSO-d_6) and ^{19}F NMR (375 MHz, $CDCl_3$)

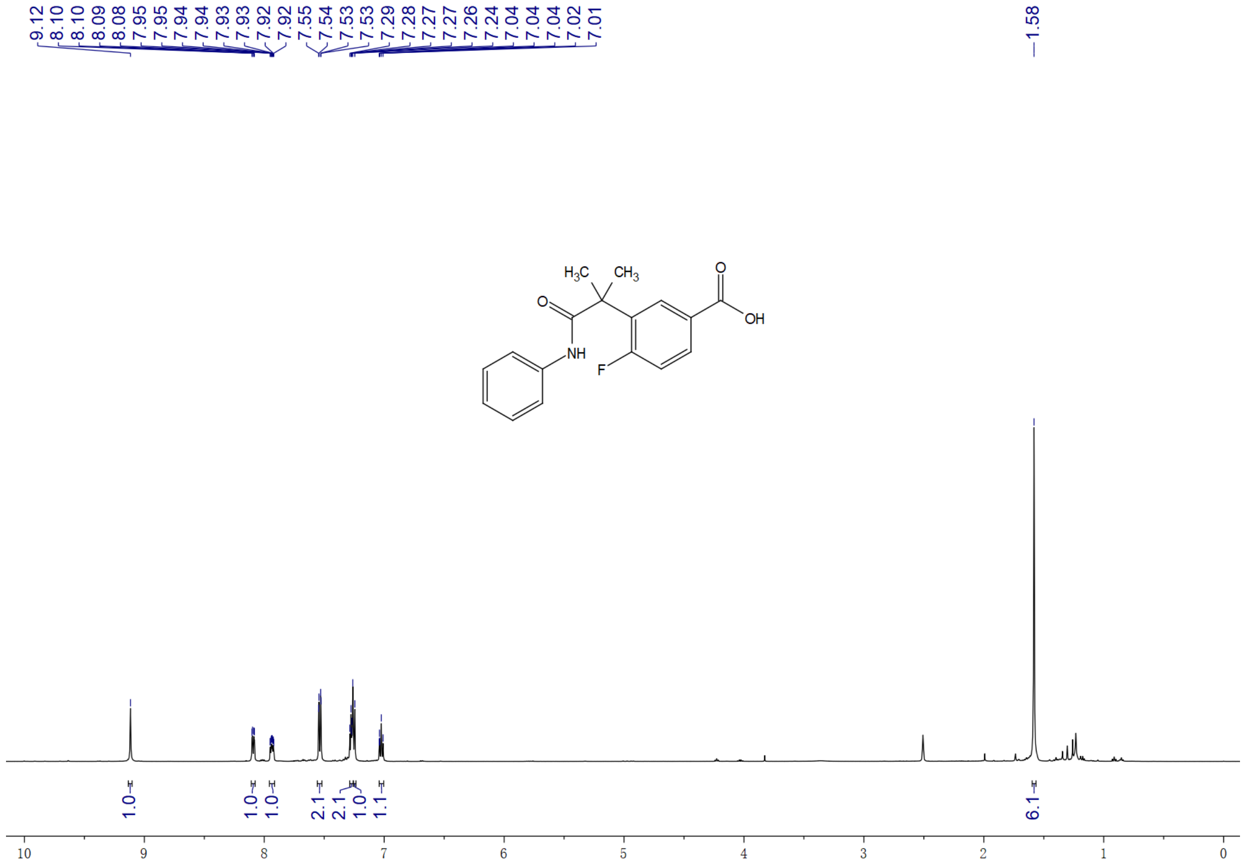

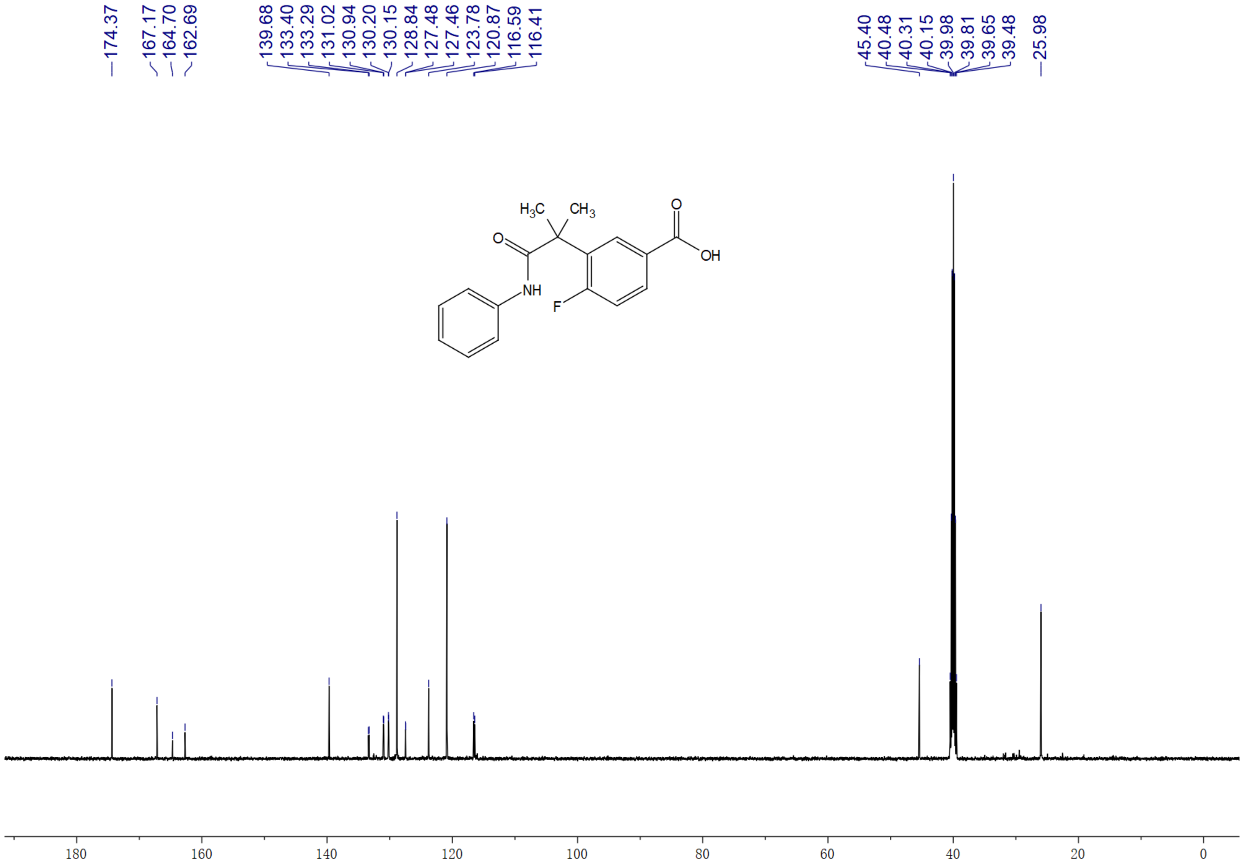

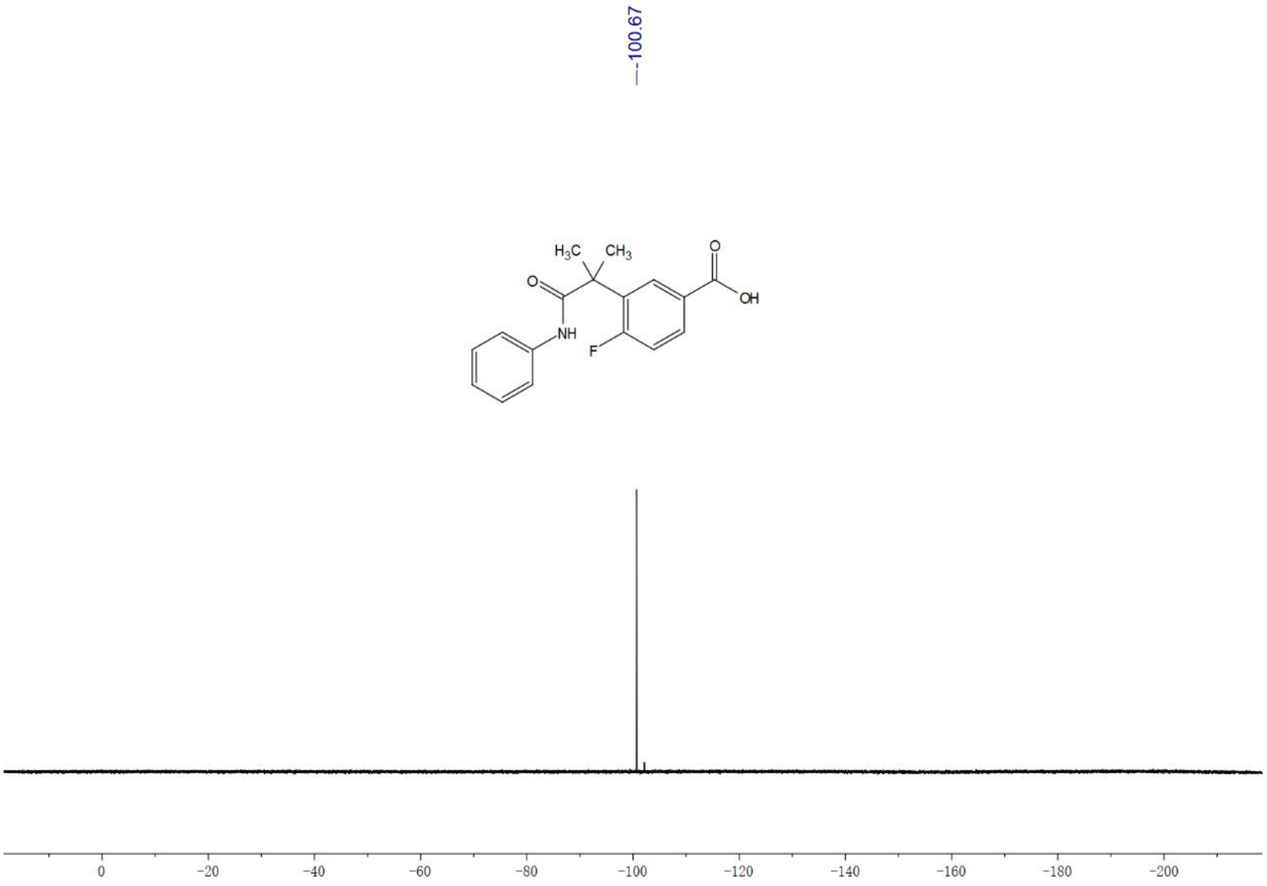
-100.67
H3C
CH3
O
OH
NH
F
0
-20
-40
-60
-80
-100
-120
-140
-160
-180
-200

4-Chloro-3-(2-methyl-1-oxo-1-(phenylamino)propan-2-yl)benzoic acid, **3oa**, ^{1}H NMR (500 MHz, DMSO-d_6) and ^{13}C NMR (125 MHz, DMSO-d_6)

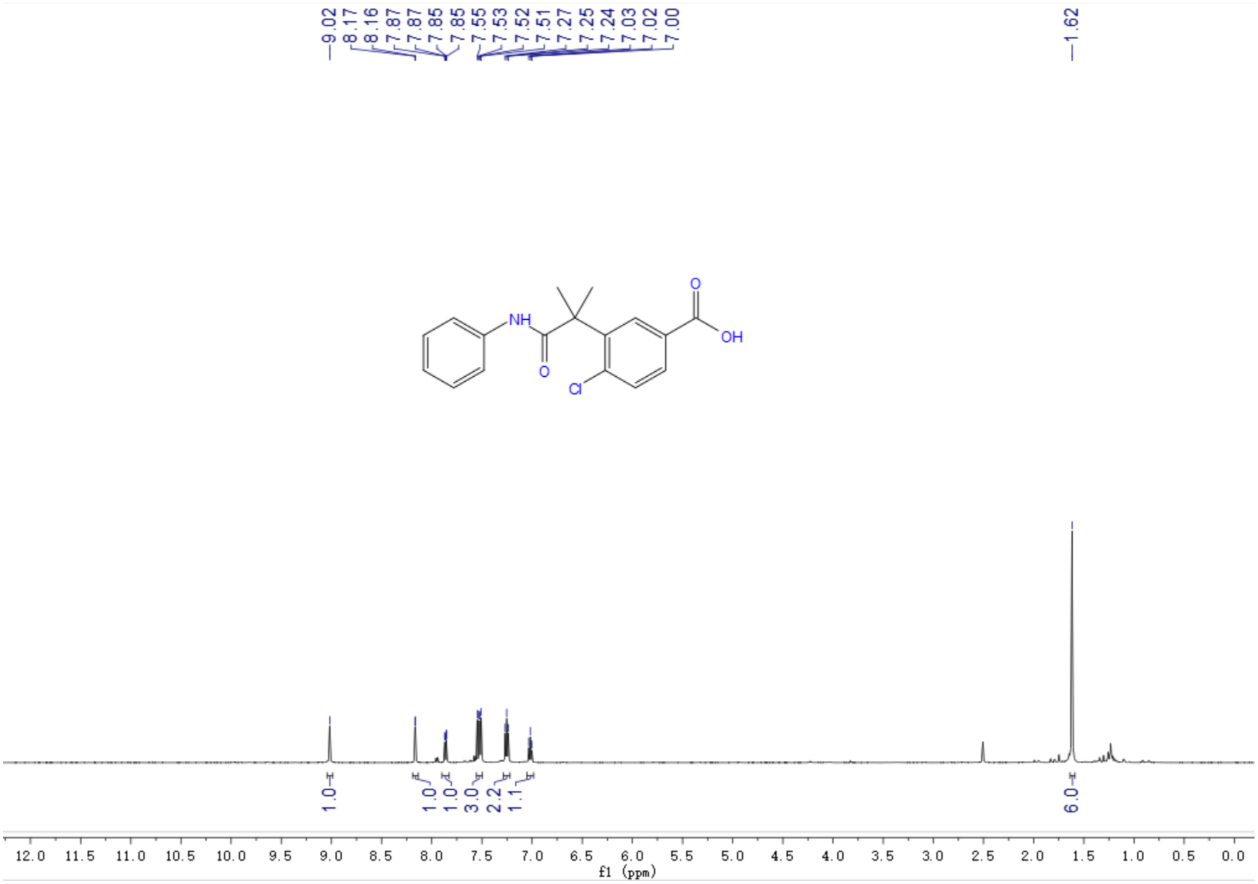

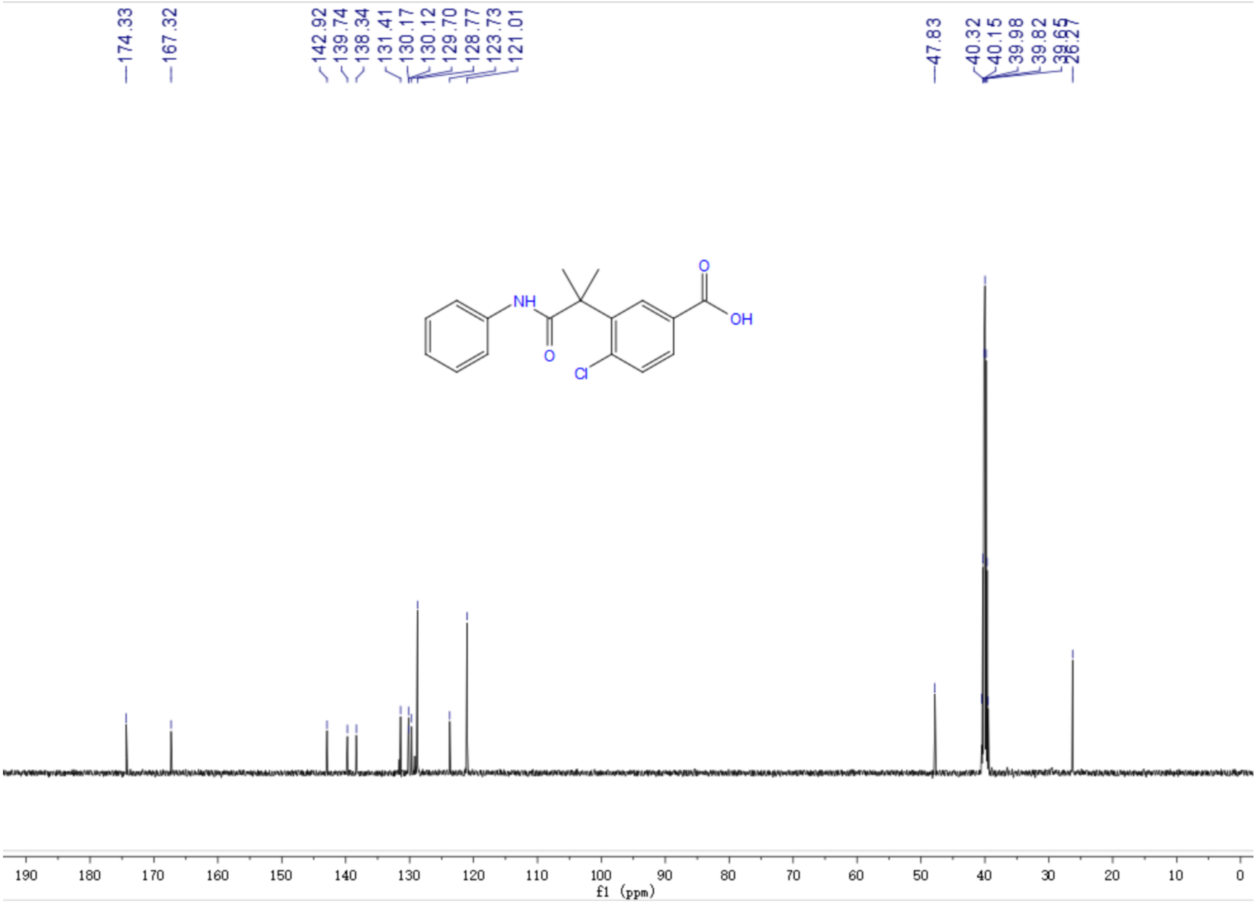

4-Bromo-3-(2-methyl-1-oxo-1-(phenylamino)propan-2-yl)benzoic acid, **3pa**, ^{1}H NMR (500 MHz, DMSO-d_6) and ^{13}C NMR (125 MHz, DMSO-d_6)

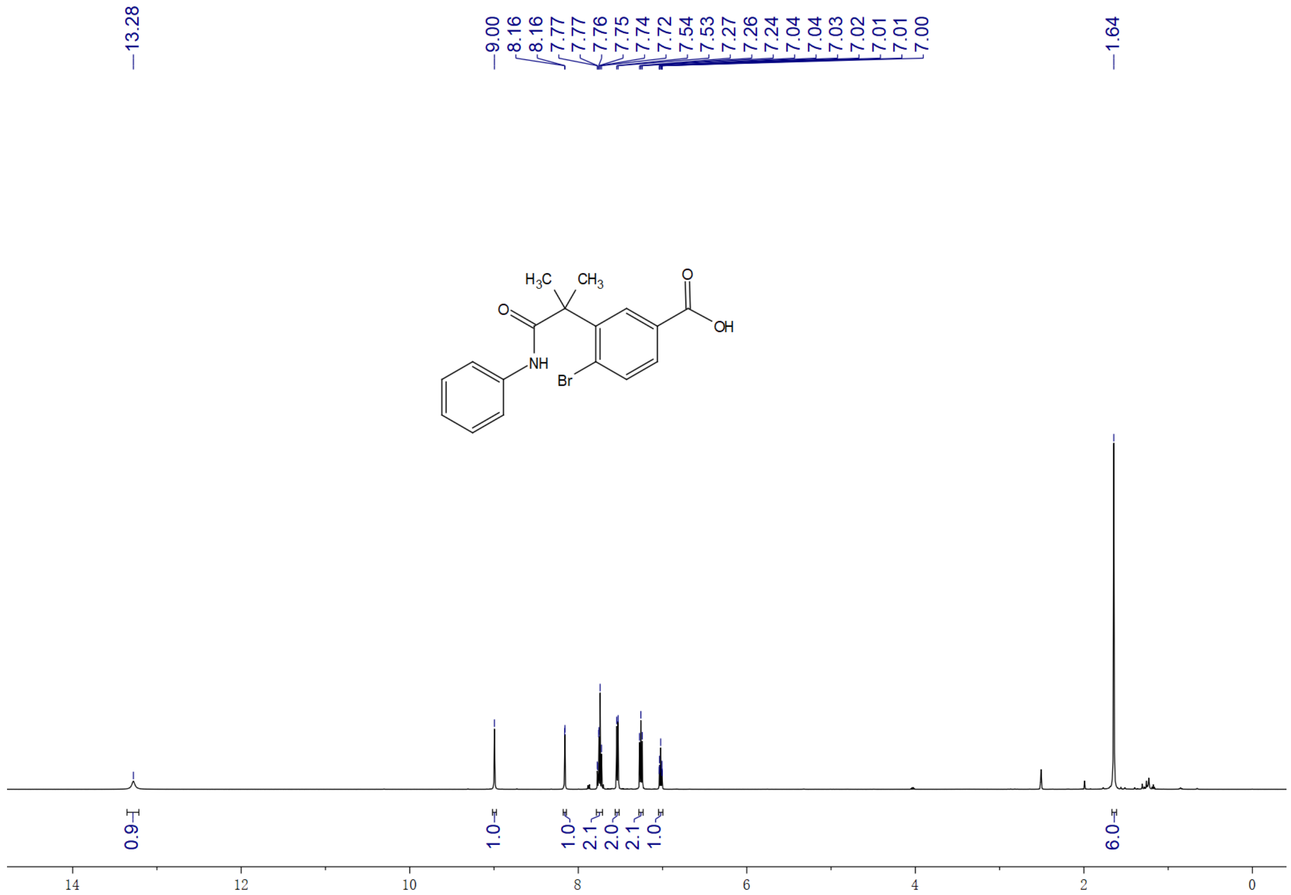

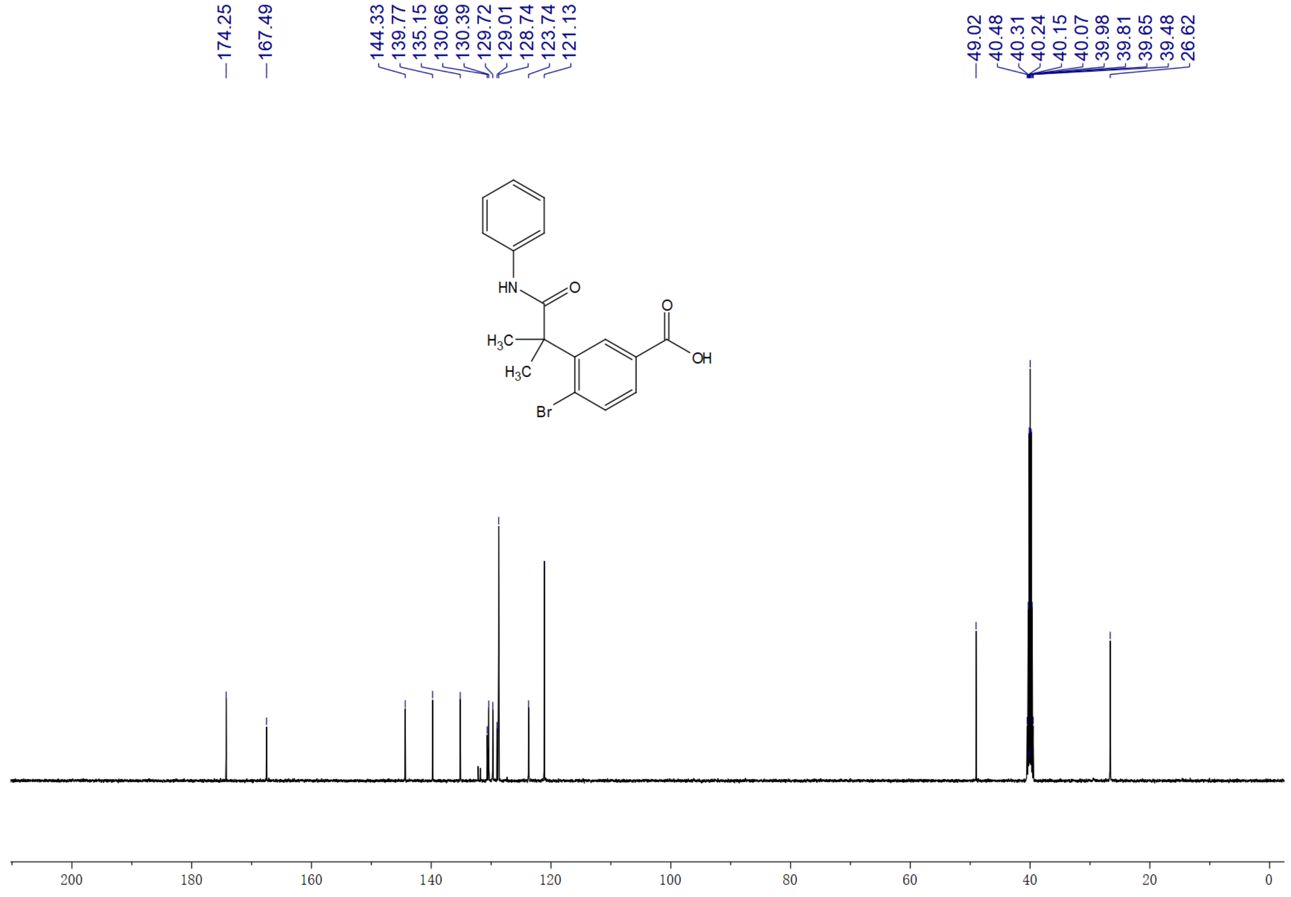

2-Chloro-4-methoxy-3-(2-methyl-1-oxo-1-(phenylamino)propan-2-yl)benzoic acid, **3qa**, ^{1}H NMR (500 MHz, DMSO-d_6) and ^{13}C NMR (125 MHz, DMSO-d_6)

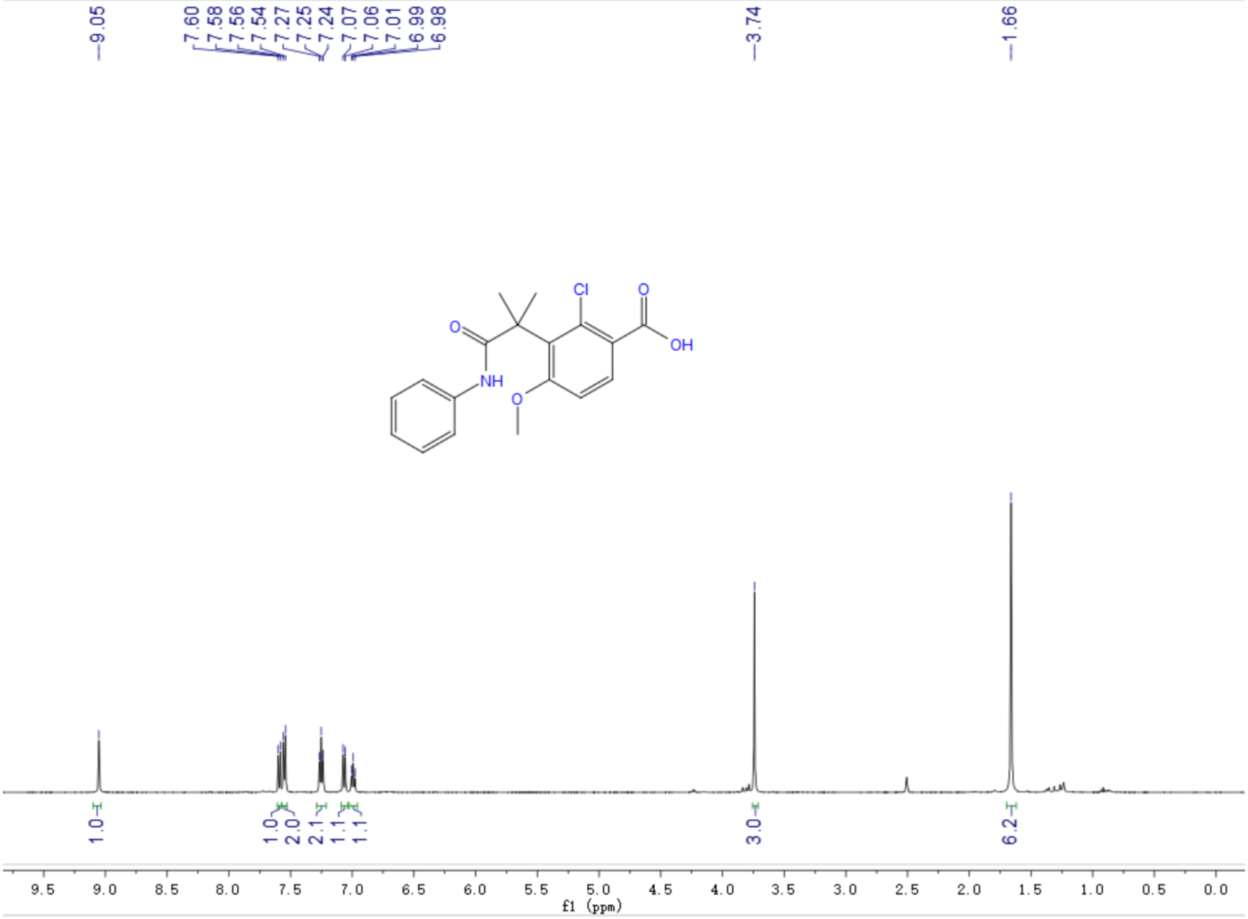

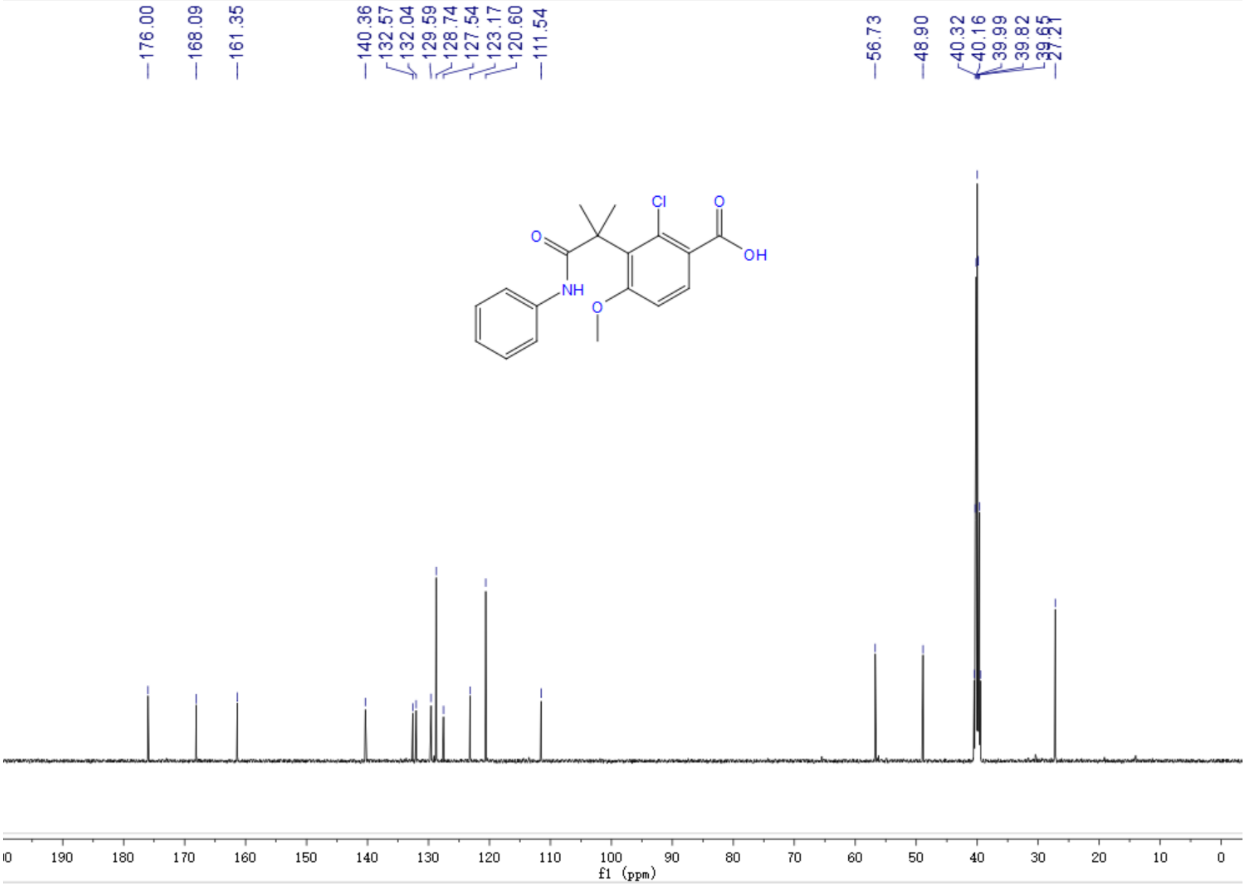

4-Methoxy-2-methyl-3-(2-methyl-1-oxo-1-(phenylamino)propan-2-yl)benzoic acid, **3ra**, ^{1}H NMR (500 MHz, DMSO-d_6) and ^{13}C NMR (125 MHz, DMSO-d_6)

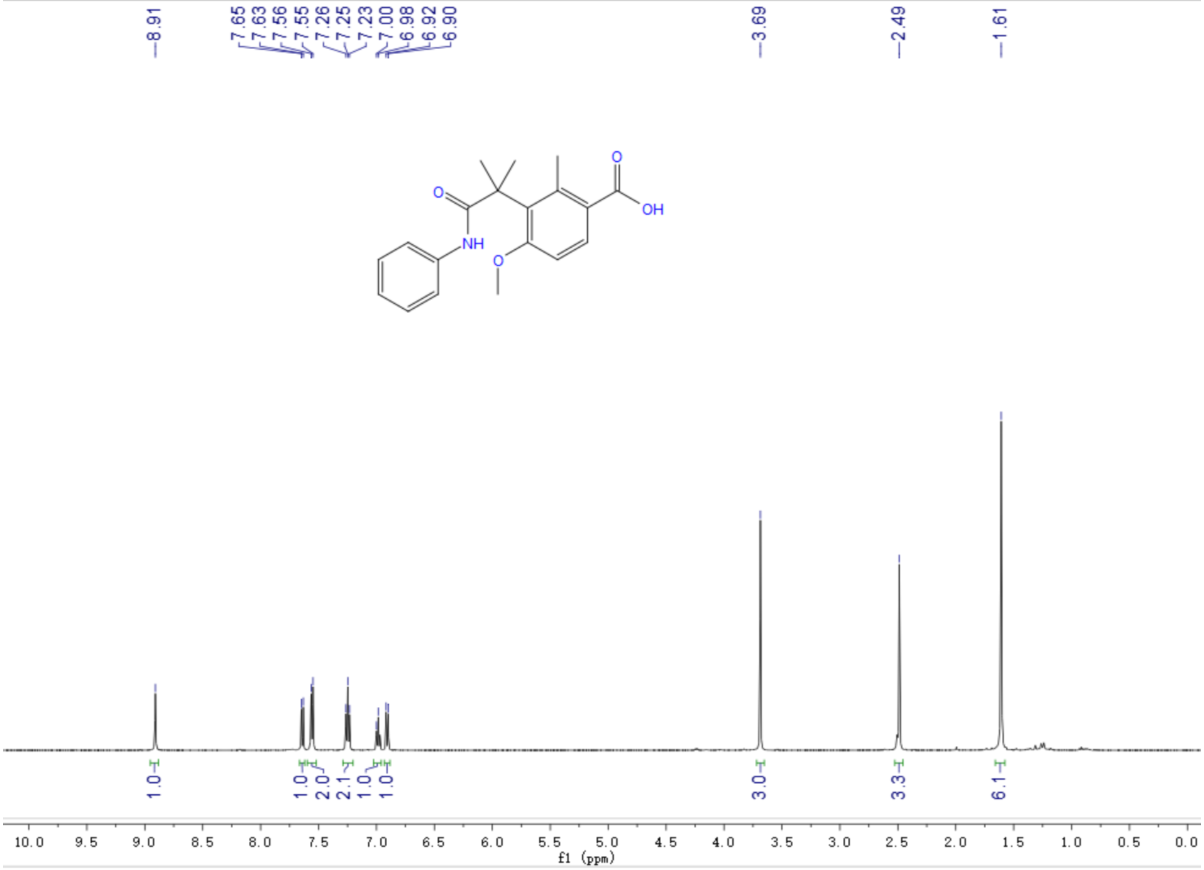

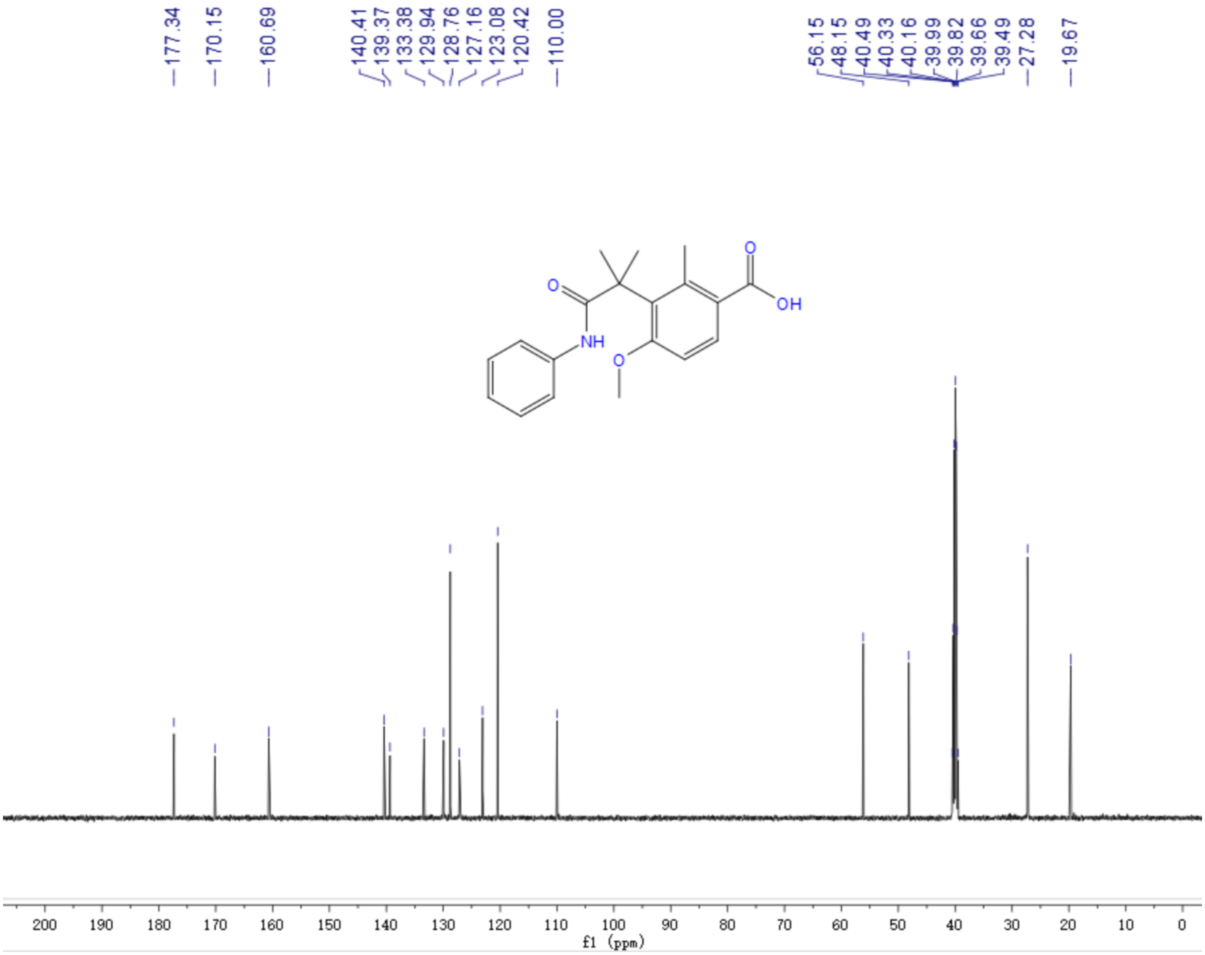

4-Fluoro-2-methyl-3-(2-methyl-1-oxo-1-(phenylamino)propan-2-yl)benzoic acid, **3sa**, ^{1}H NMR (500 MHz, DMSO-d_6), ^{13}C NMR (125 MHz, DMSO-d_6) and ^{19}F NMR (471 MHz, $CDCl_3$)

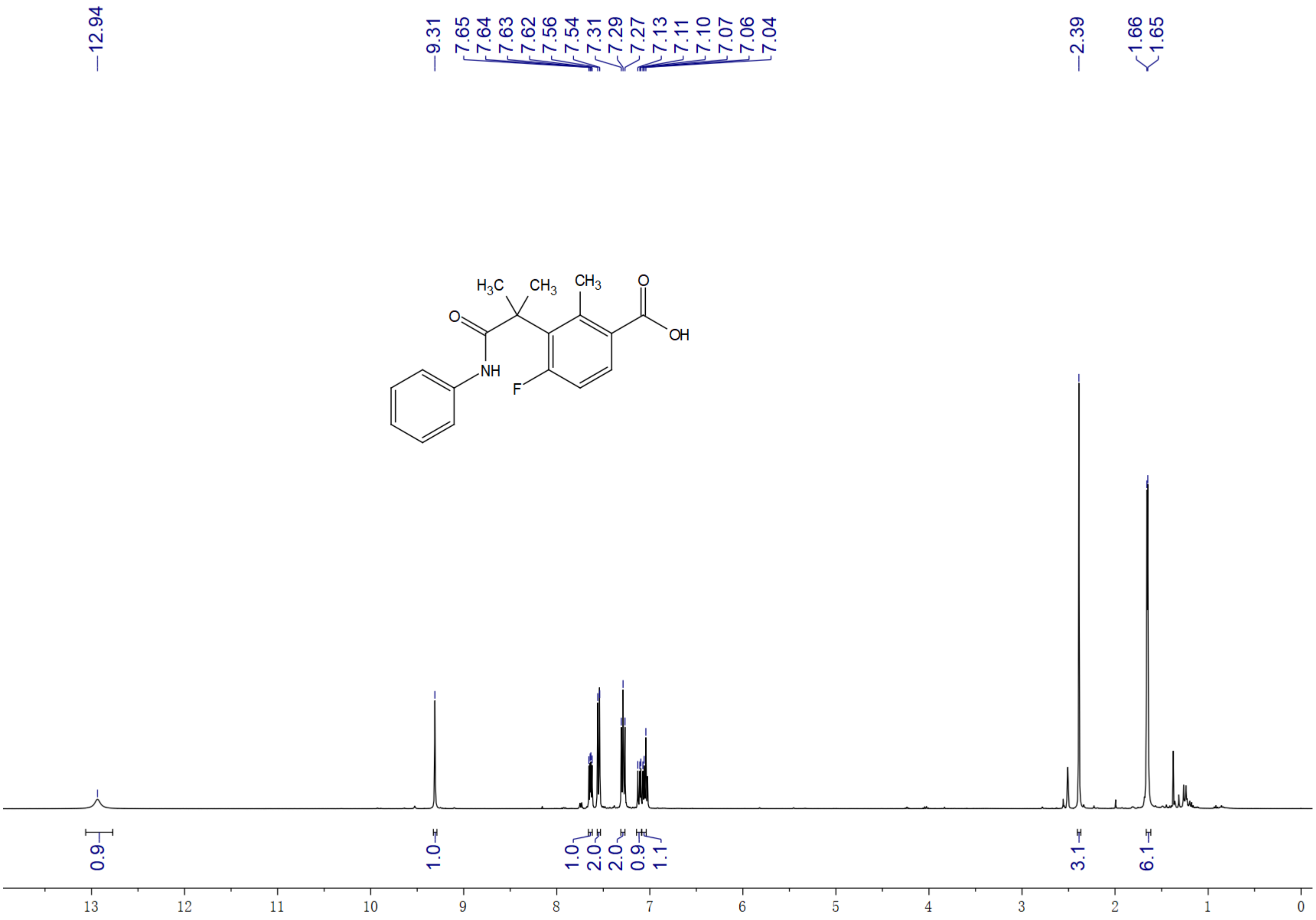

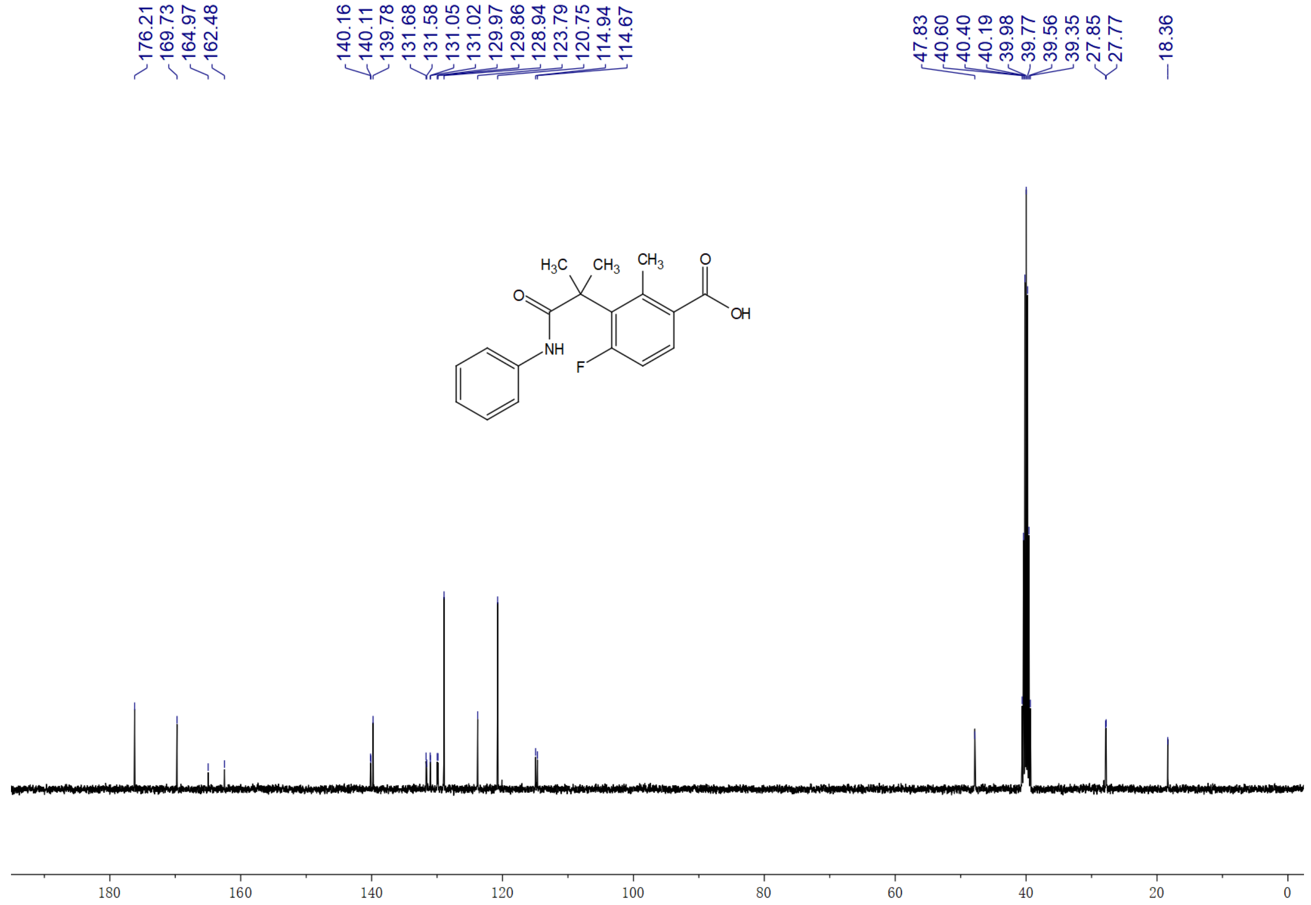

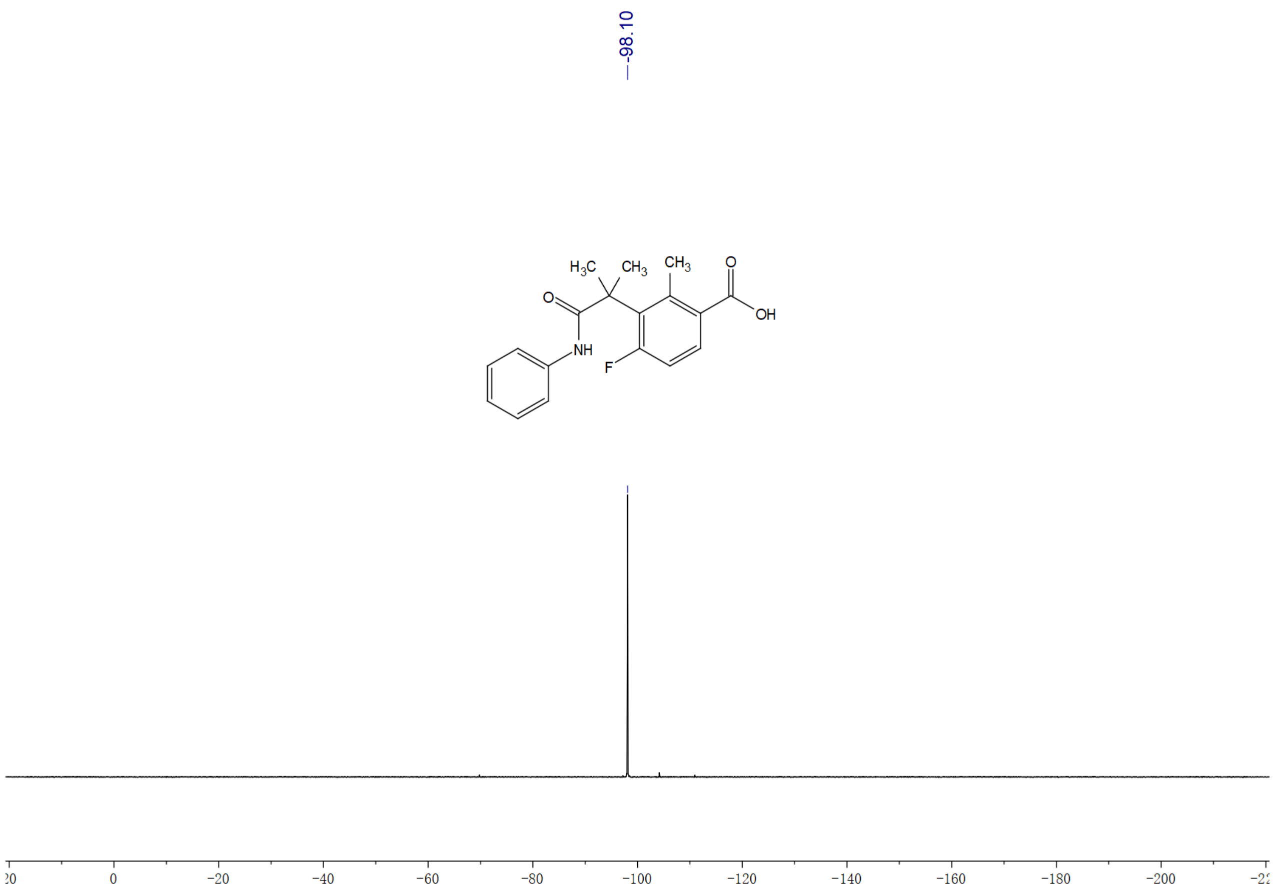
-98.10
H3C
CH3
CH3
O
OH
O
NH
F
20
0
-20
-40
-60
-80
-100
-120
-140
-160
-180
-200

Methyl-4-chloro-2-fluoro-3-(2-methyl-1-oxo-1-(phenylamino)propan-2-yl)benzoate, **3ta**, ^{1}H NMR (400 MHz, CDCl$_3$), ^{13}C NMR (125 MHz, CDCl$_3$) and ^{19}F NMR (375 MHz, CDCl$_3$)

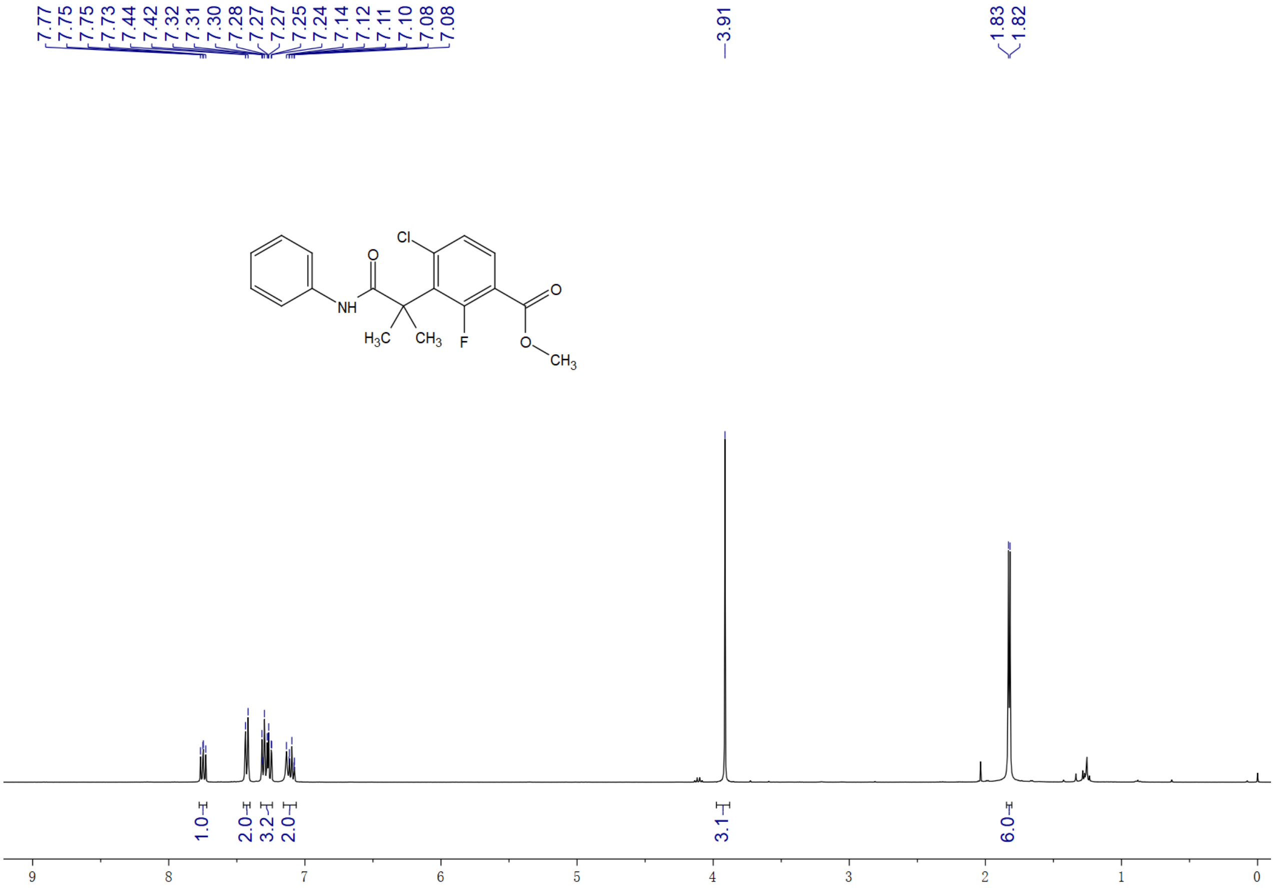

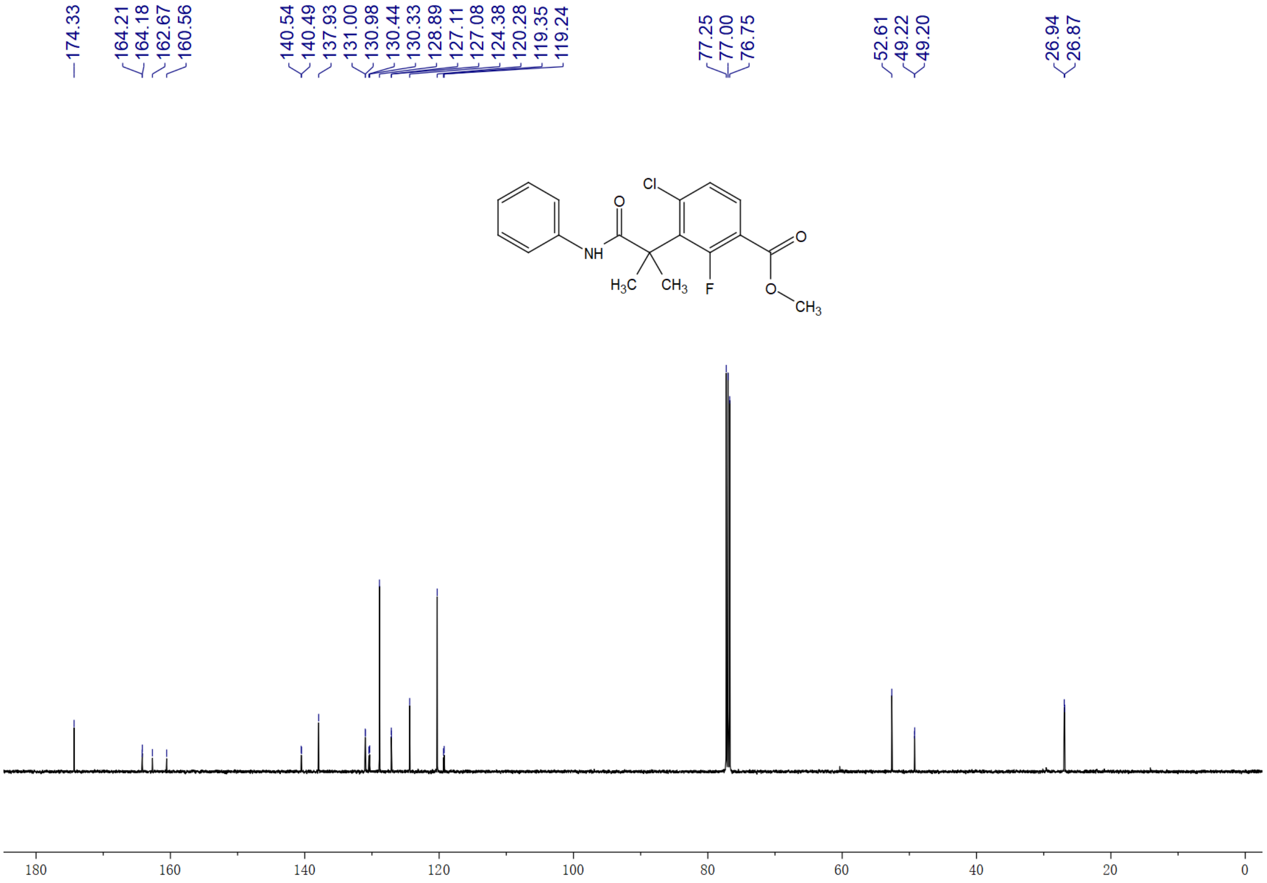

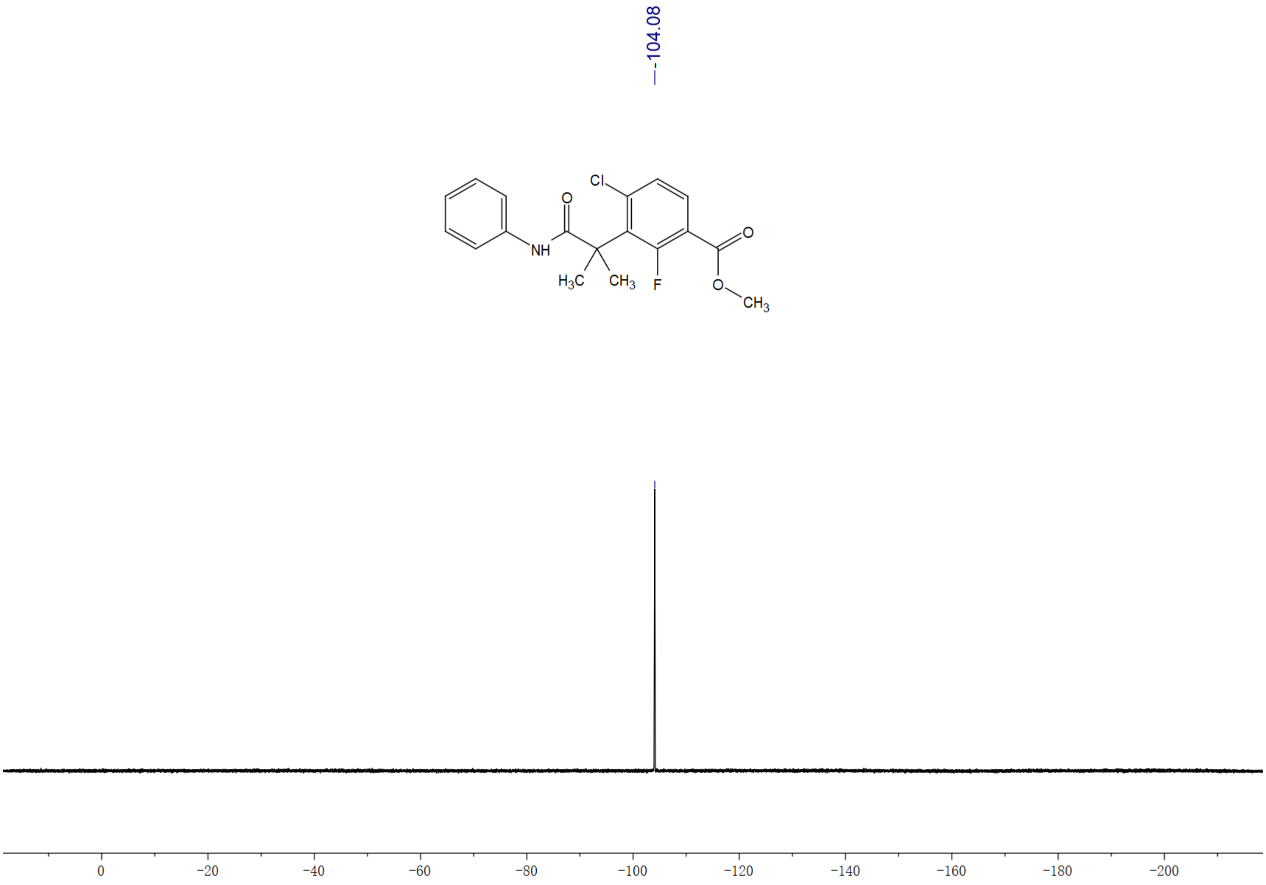

-104.08
Cl
O
NH
H3C
CH3
F
O
O
CH3
0
-20
-40
-60
-80
-100
-120
-140
-160
-180
-200

Methyl-4-bromo-3-fluoro-5-(2-methyl-1-oxo-1-(phenylamino)propan-2-yl)benzoate, **3ua**, ^{1}H NMR (400 MHz, $CDCl_3$), ^{13}C NMR (125 MHz, $CDCl_3$) and ^{19}F NMR (375 MHz, $CDCl_3$)

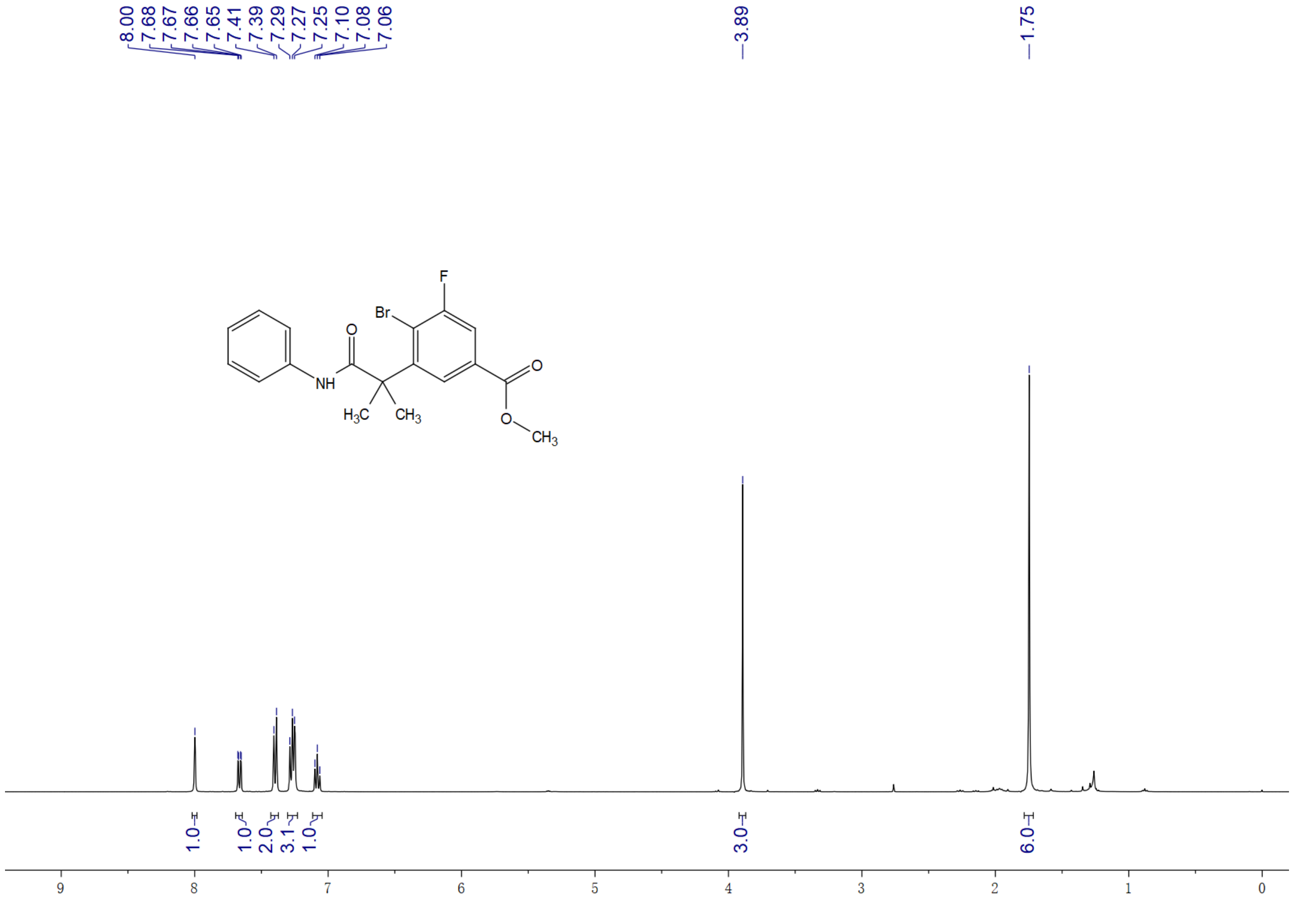

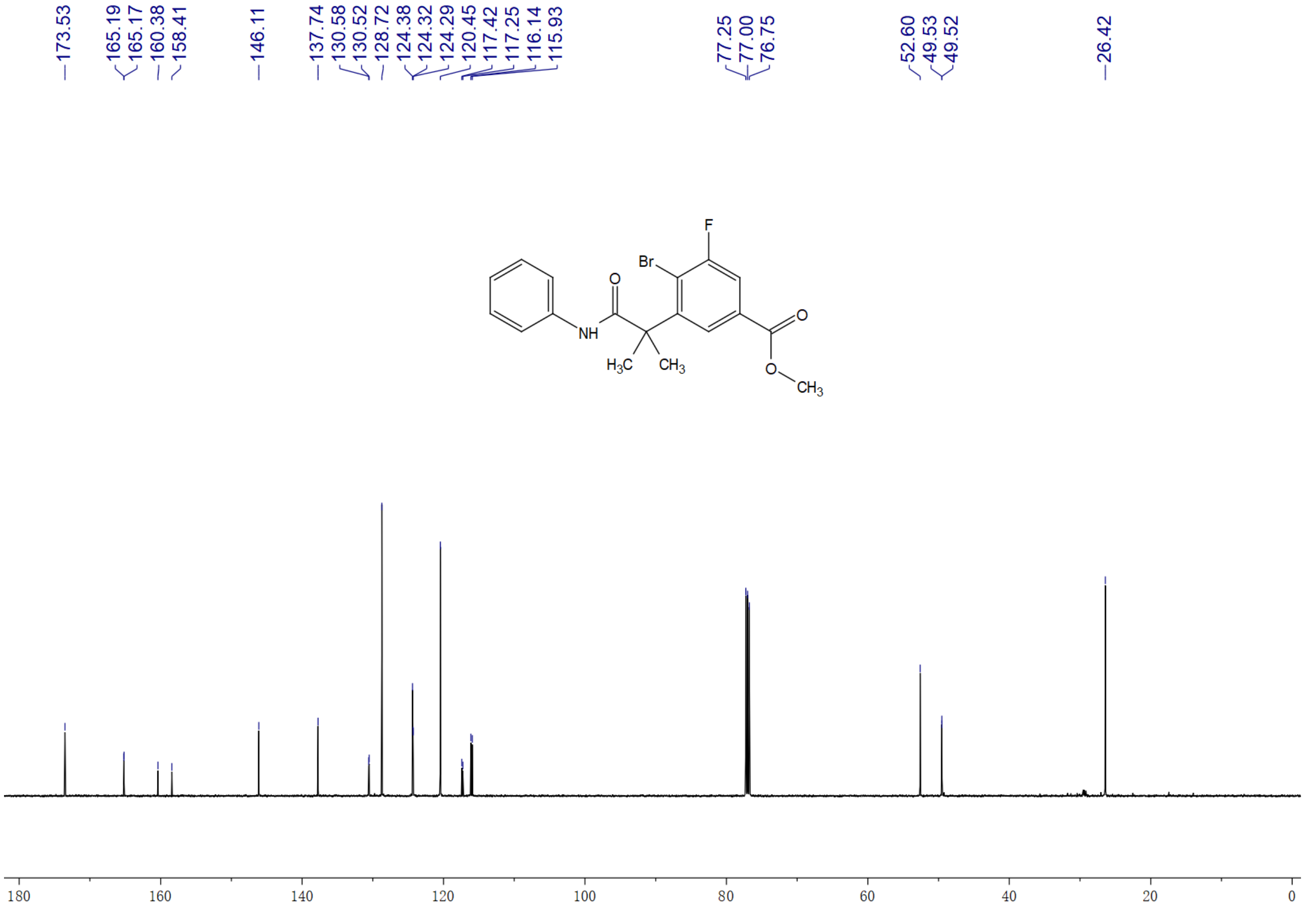

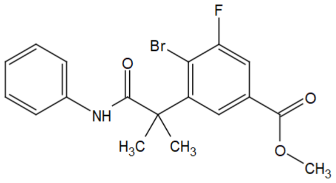

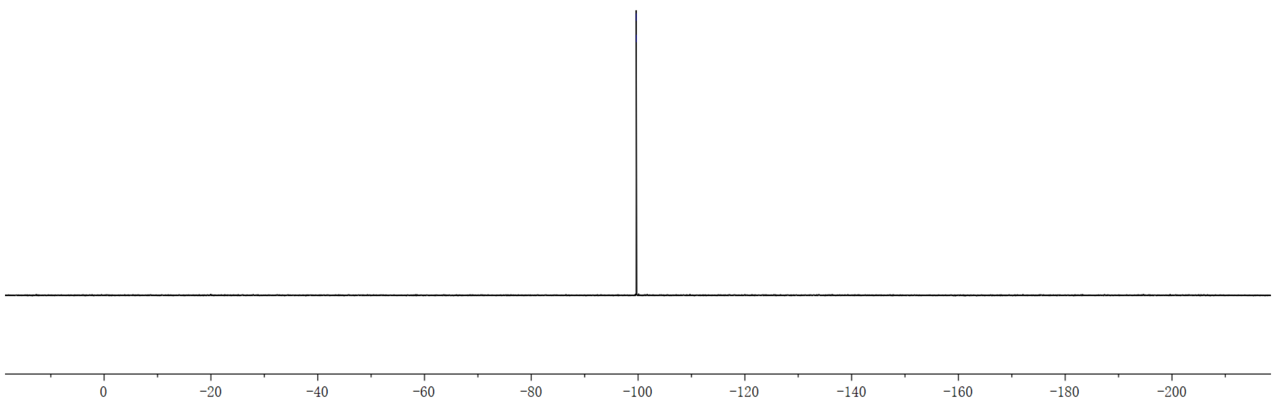

-99.67
-99.69
0
-20
-40
-60
-80
-100
-120
-140
-160
-180
-200

2,5-Dimethoxy-3-(2-methyl-1-oxo-1-(phenylamino)propan-2-yl)benzoic acid, **3va**, ^{1}H NMR (500 MHz, DMSO-d_6) and ^{13}C NMR (125 MHz, DMSO-d_6)

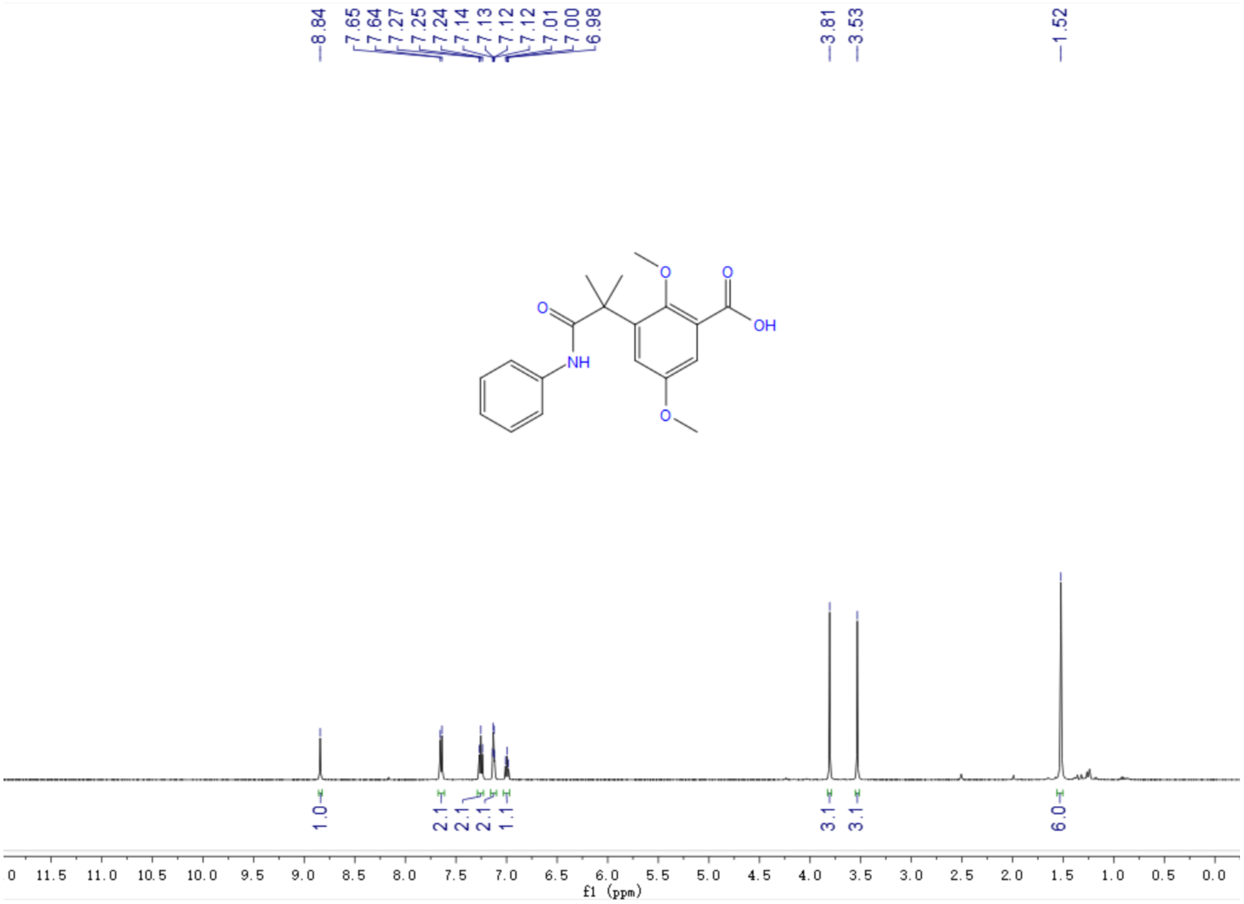

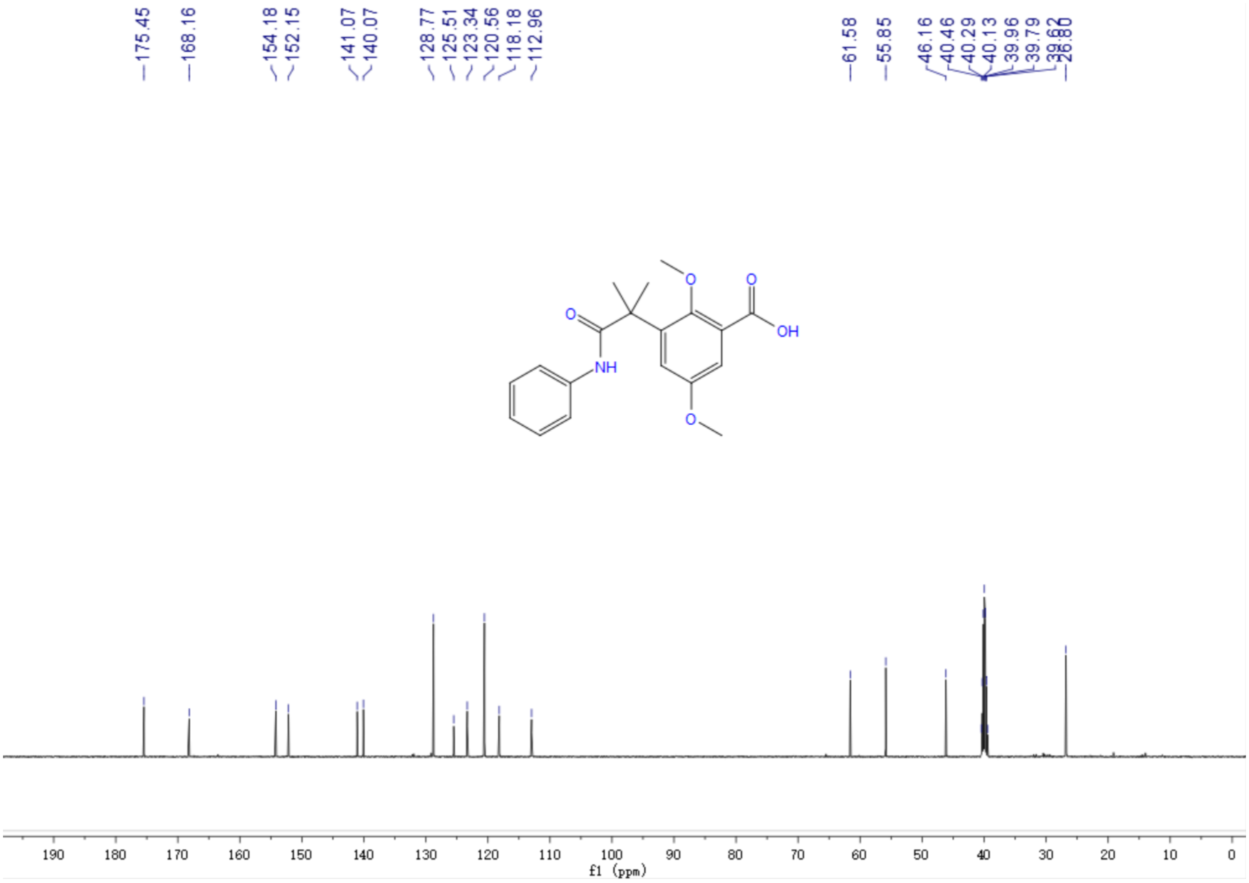

Methyl-4-(2-methyl-1-oxo-1-(phenylamino)propan-2-yl)-2-naphthoate, **3wa**, ^{1}H NMR (500 MHz, $CDCl_3$) and ^{13}C NMR (125 MHz, $CDCl_3$)

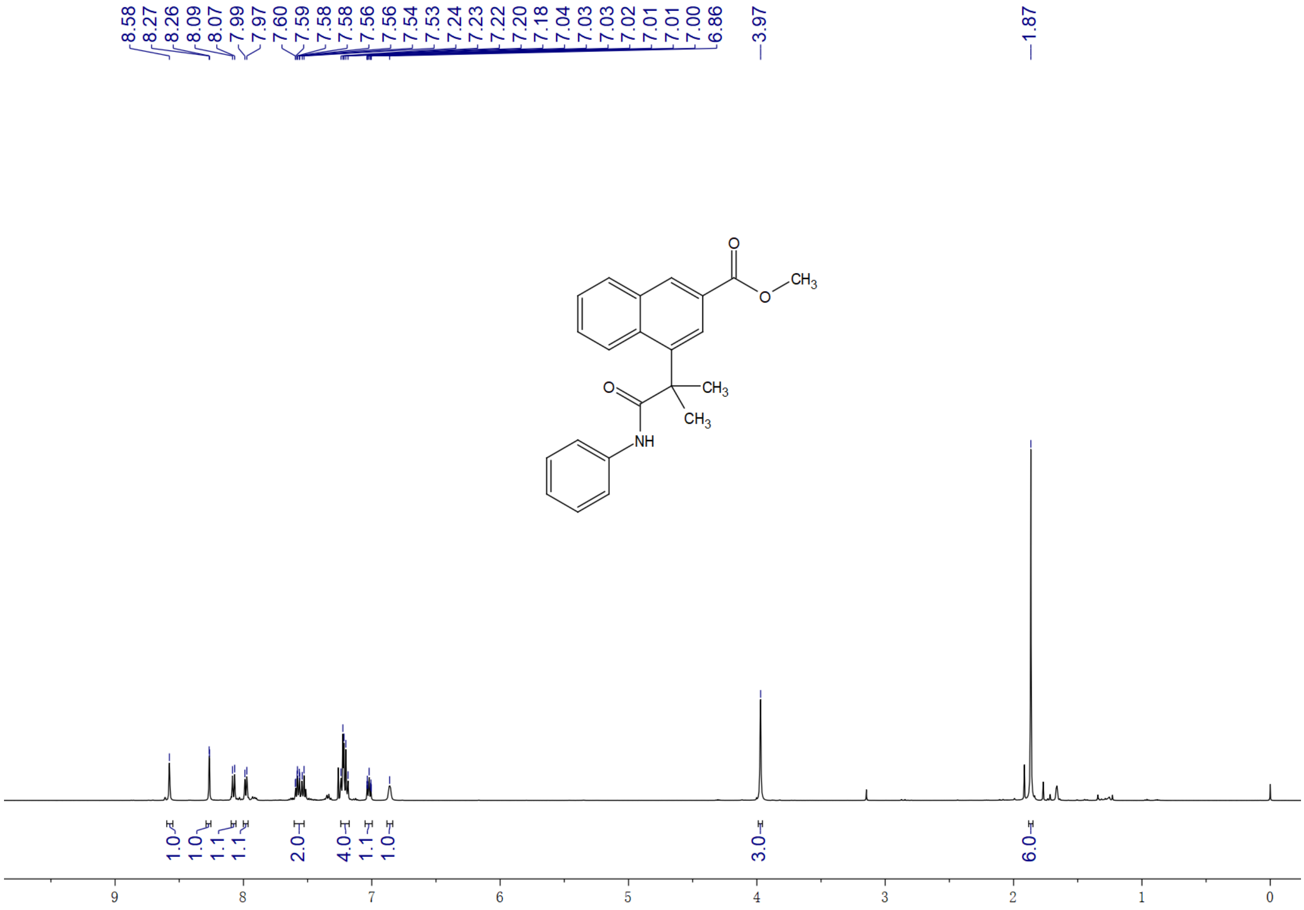

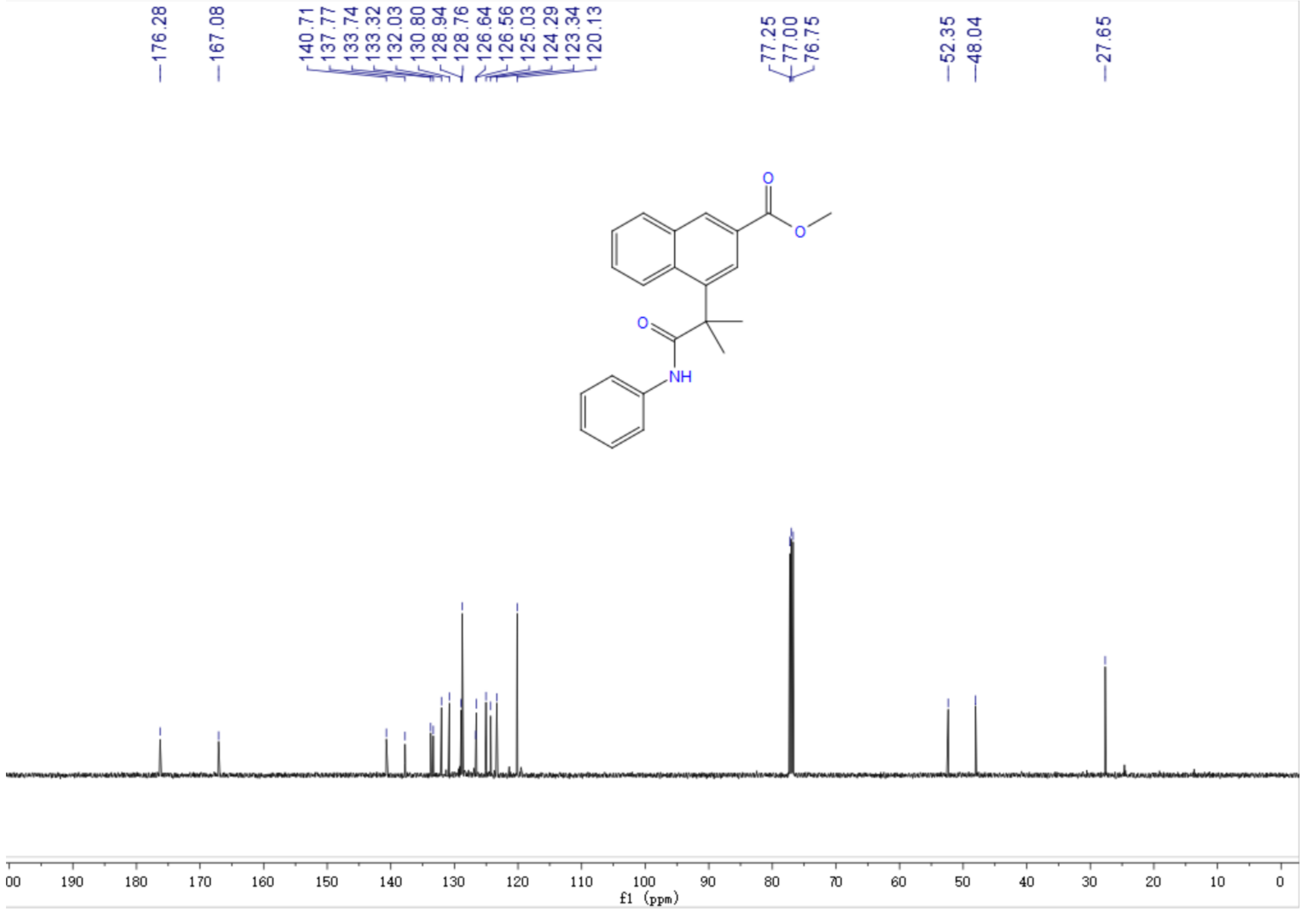

Methyl-7-(2-methyl-1-oxo-1-(phenylamino)propan-2-yl)benzo[d][1,3]dioxole-5-carboxylate, **3xa**, ^{1}H NMR (500 MHz, $CDCl_3$) and ^{13}C NMR (125 MHz, $CDCl_3$)

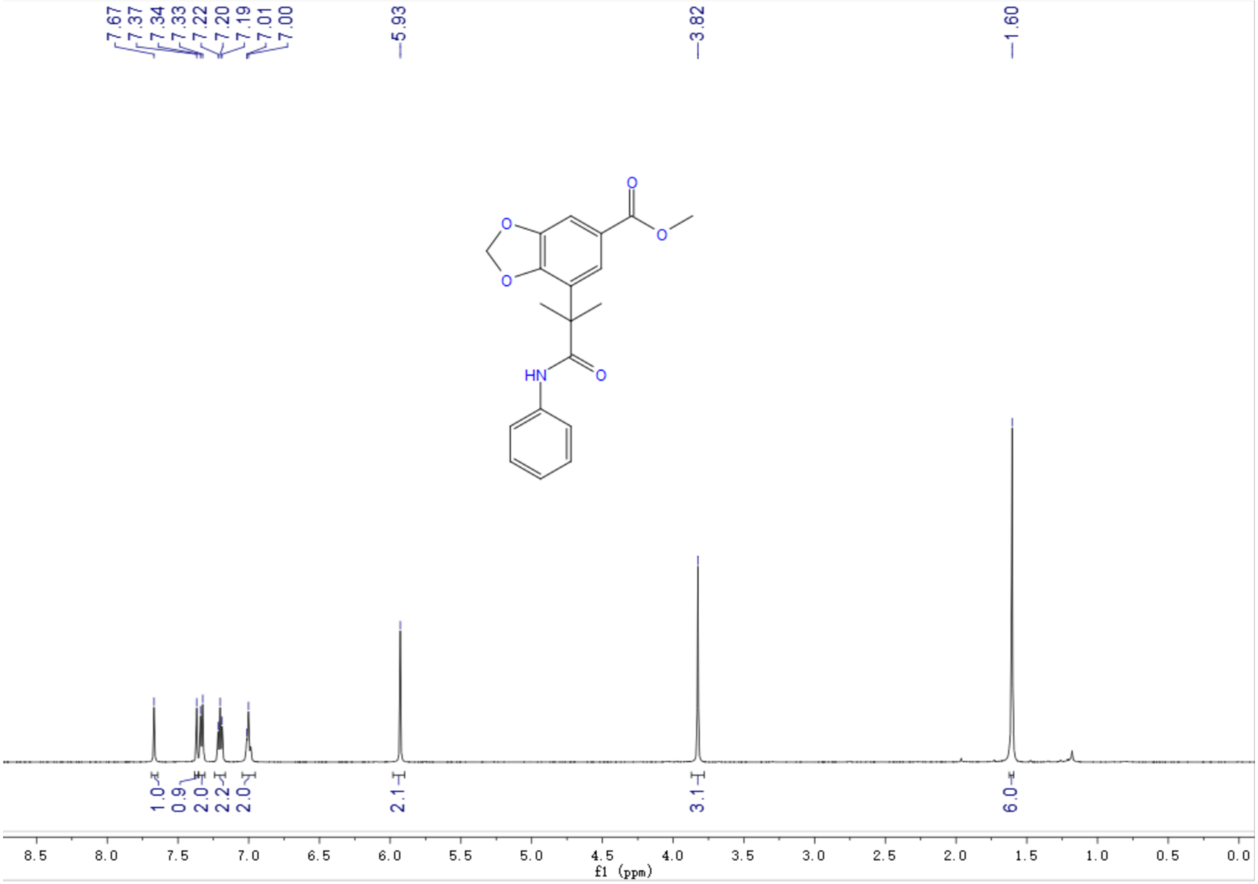

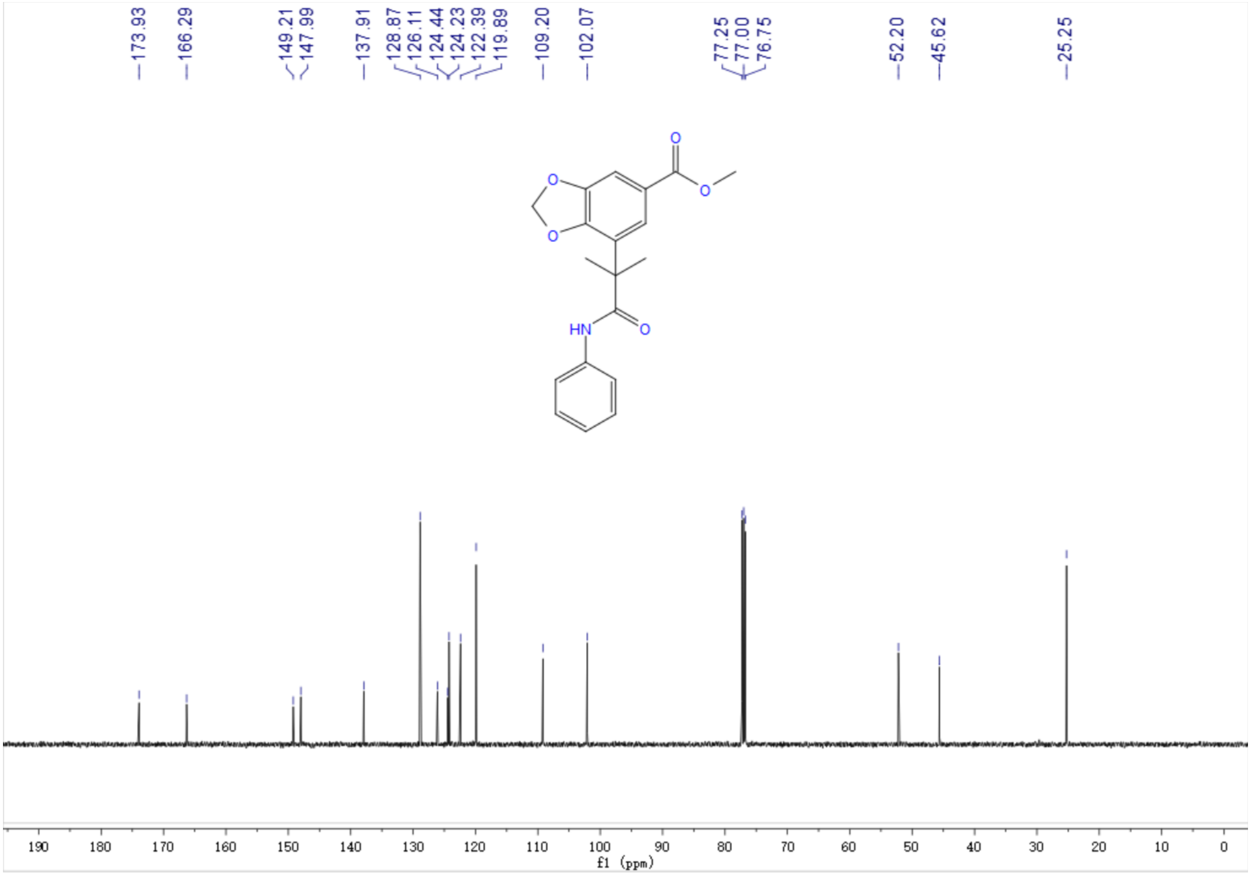

Methyl-2,2-difluoro-7-(2-methyl-1-oxo-1-(phenylamino)propan-2-yl)benzo[d][1,3]dioxole-5-carboxylate, **3ya**, ^{1}H NMR (500 MHz, CDCl$_3$), ^{13}C NMR (125 MHz, CDCl$_3$) and ^{19}F NMR (471 MHz, CDCl$_3$)

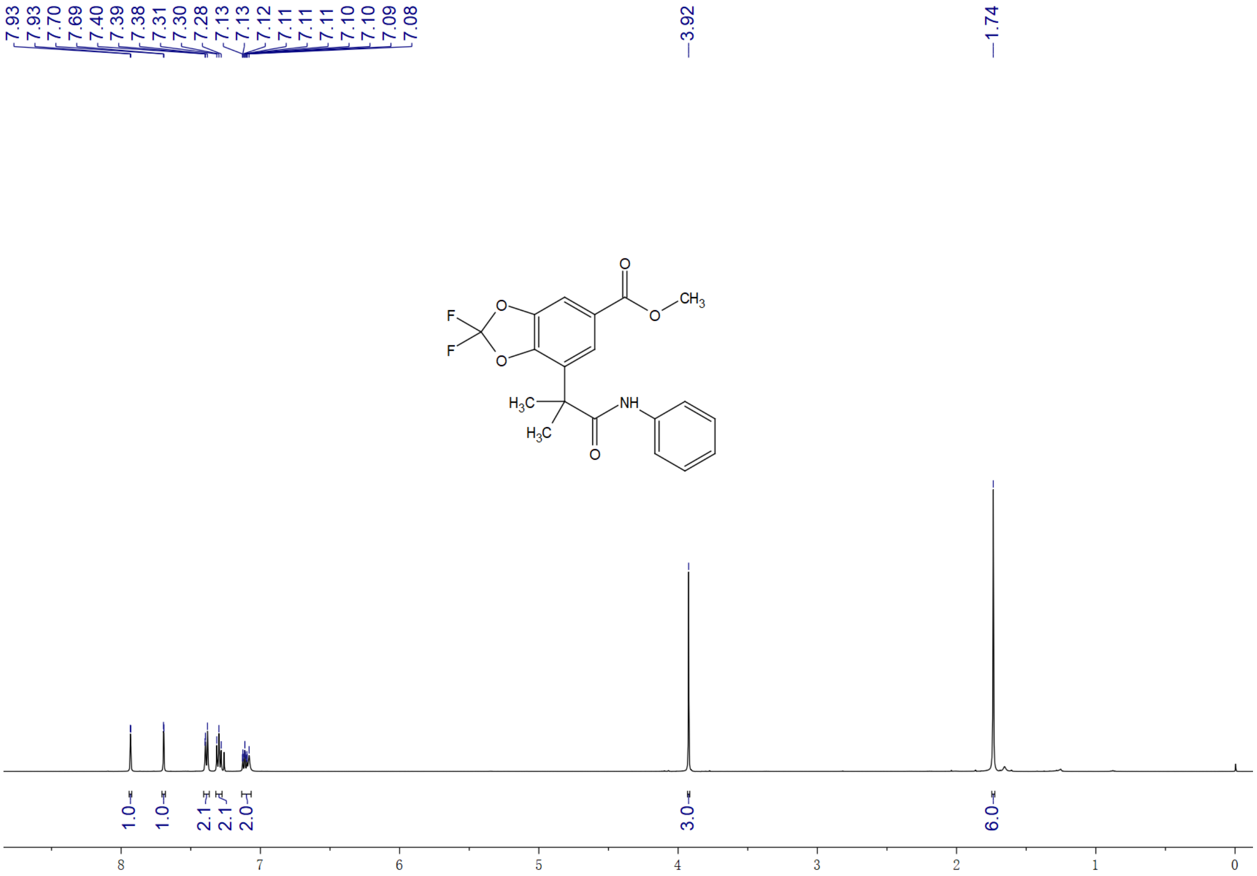

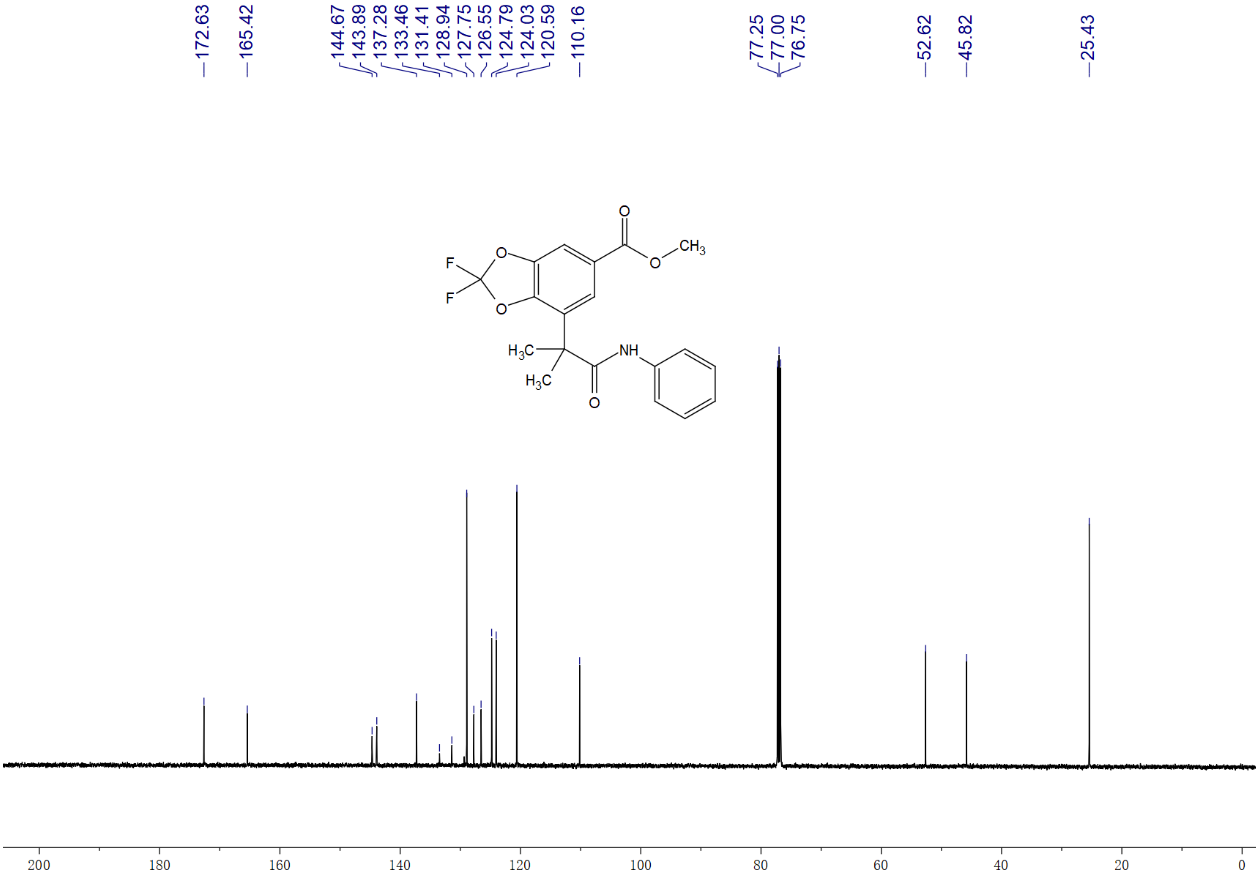

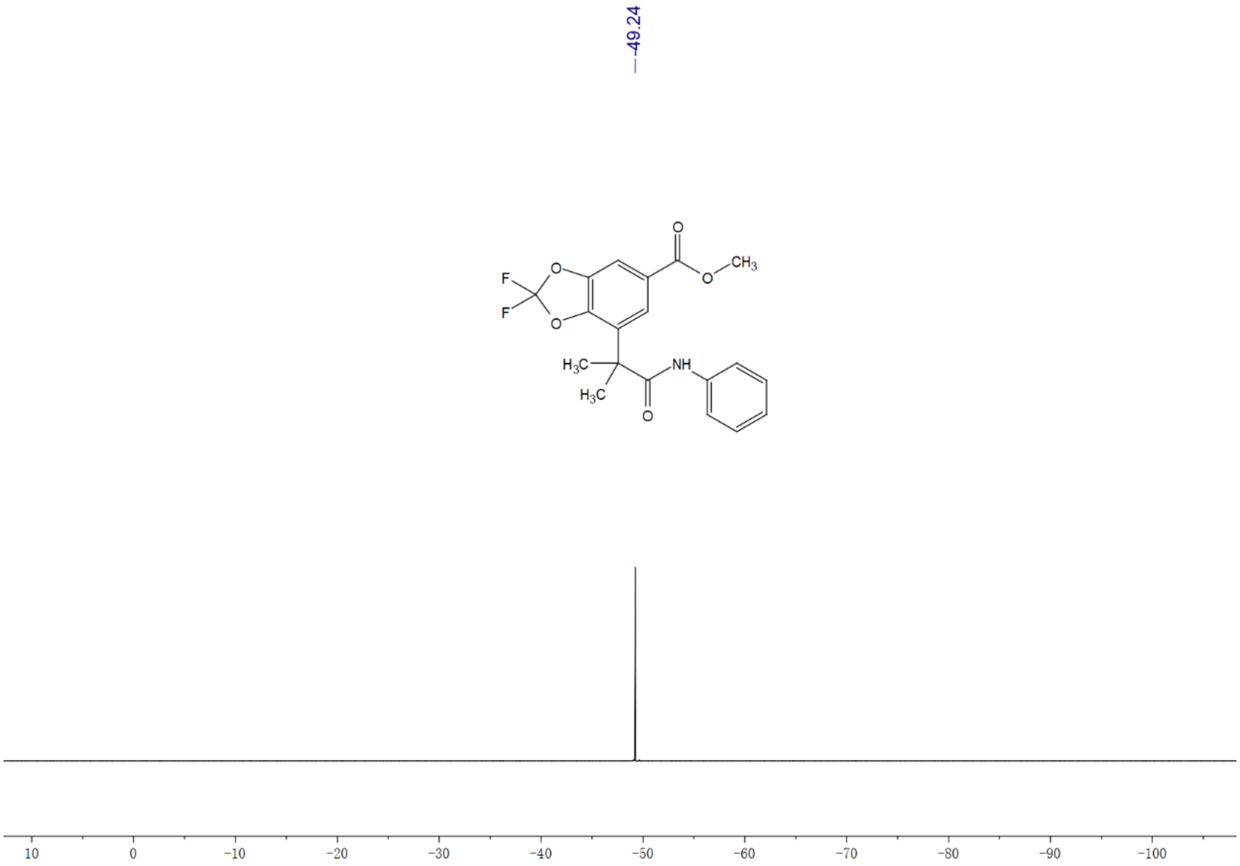

-49.24
F
F
O
O
O
O
CH3
H3C
H3C
NH
O
10
0
-10
-20
-30
-40
-50
-60
-70
-80
-90
-100

8-(2-Methyl-1-oxo-1-(phenylamino)propan-2-yl)-2,3-dihydrobenzo[b][1,4]dioxine-6-carboxylic acid, **3za**, ^{1}H NMR (500 MHz, DMSO-d_6) and ^{13}C NMR (125 MHz, DMSO-d_6)

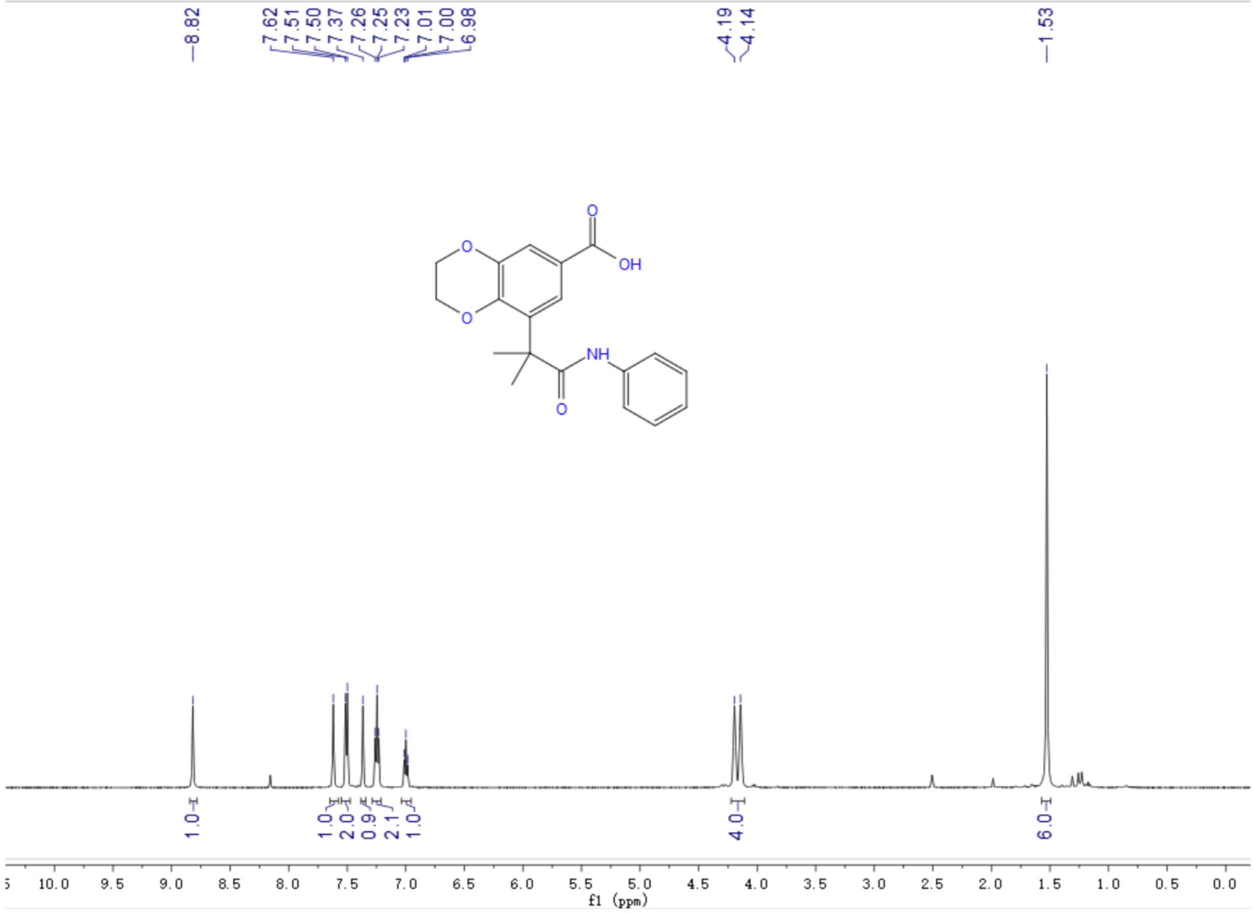

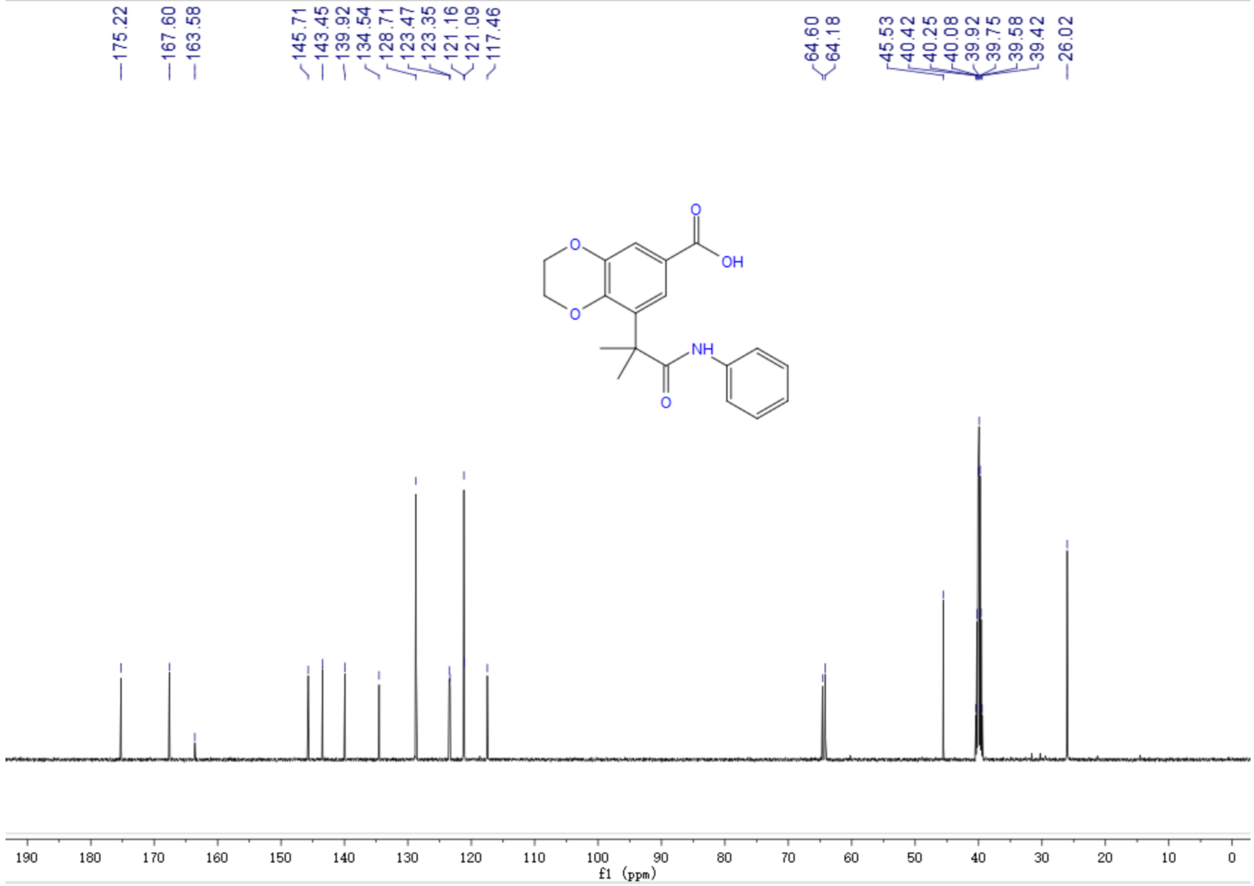

Methyl-1-methyl-7-(2-methyl-1-oxo-1-(phenylamino)propan-2-yl)-1H-indole-5-carboxylate, **3ab**, ^{1}H NMR (400 MHz, $CDCl_3$) and ^{13}C NMR (100 MHz, $CDCl_3$)

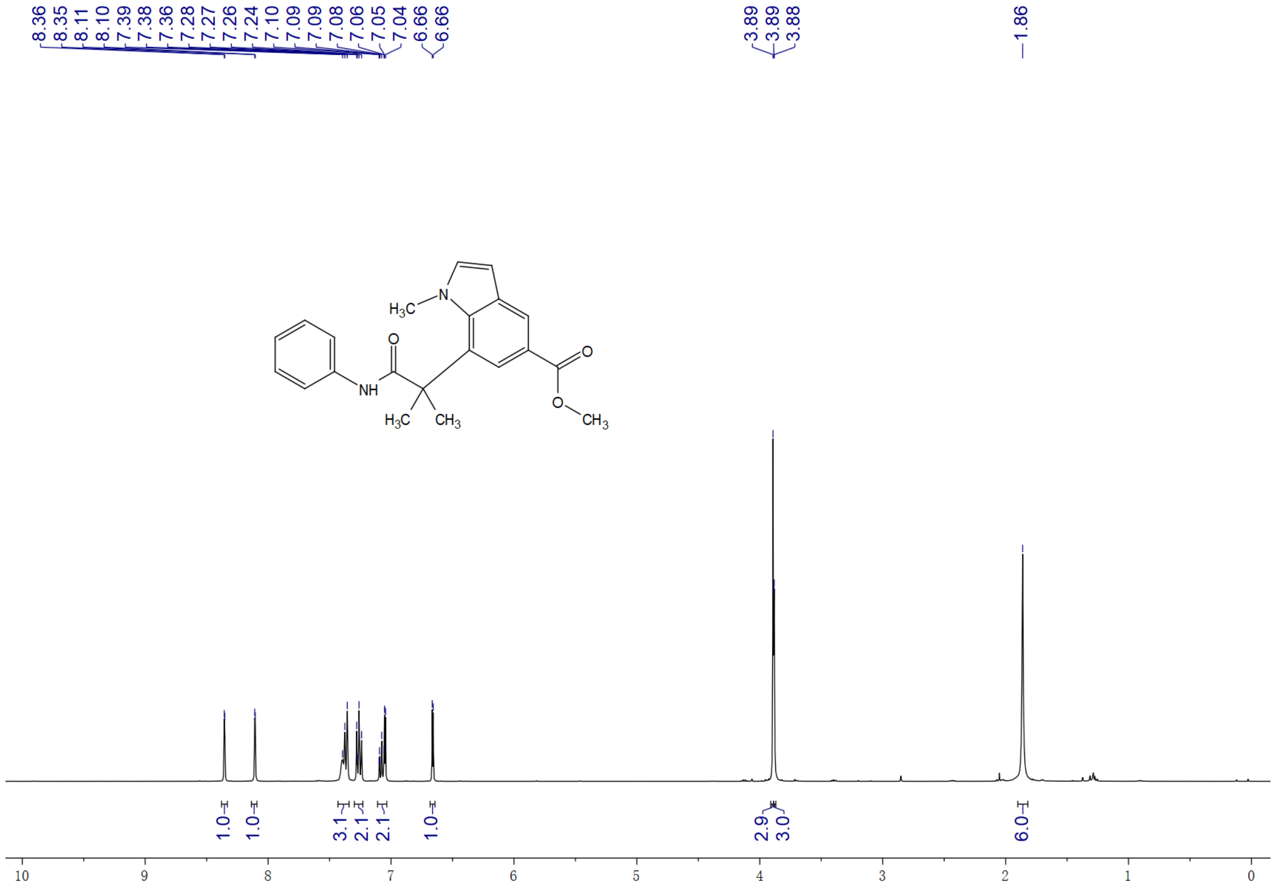

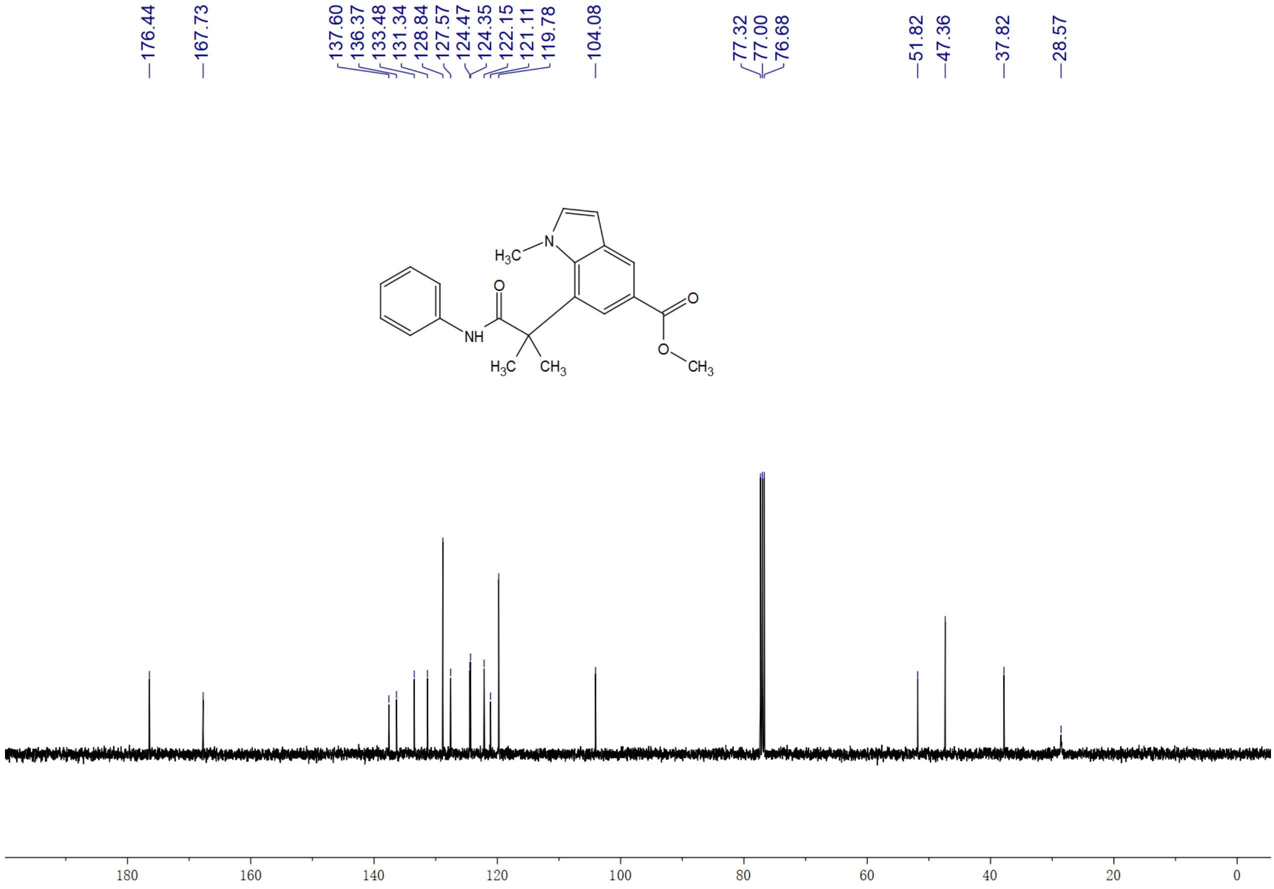

Methyl-7-(2-methyl-1-oxo-1-(phenylamino)propan-2-yl)benzofuran-5-carboxylate, **3ac**, ^{1}H NMR (400 MHz, $CDCl_3$) and ^{13}C NMR (100 MHz, $CDCl_3$)

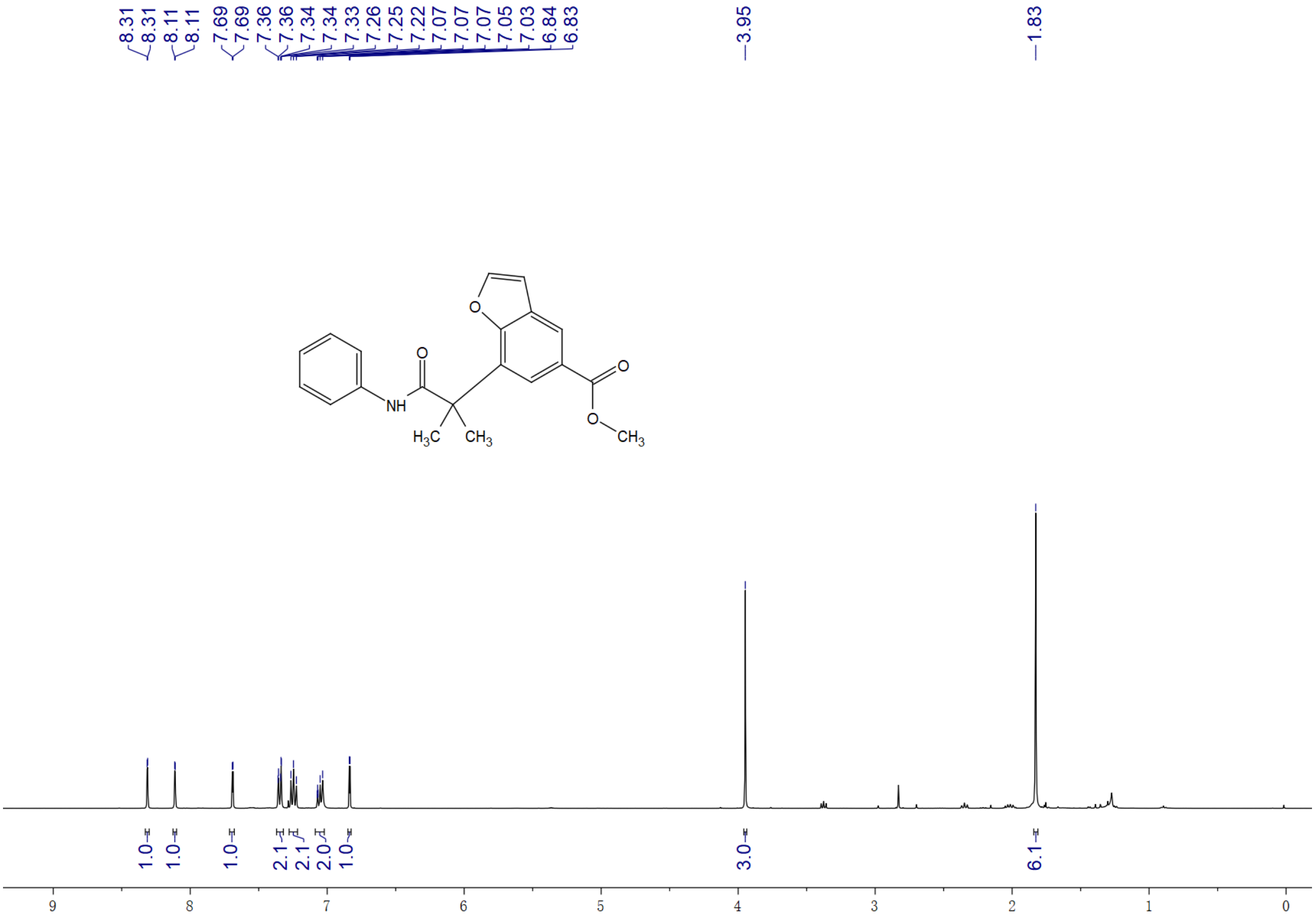

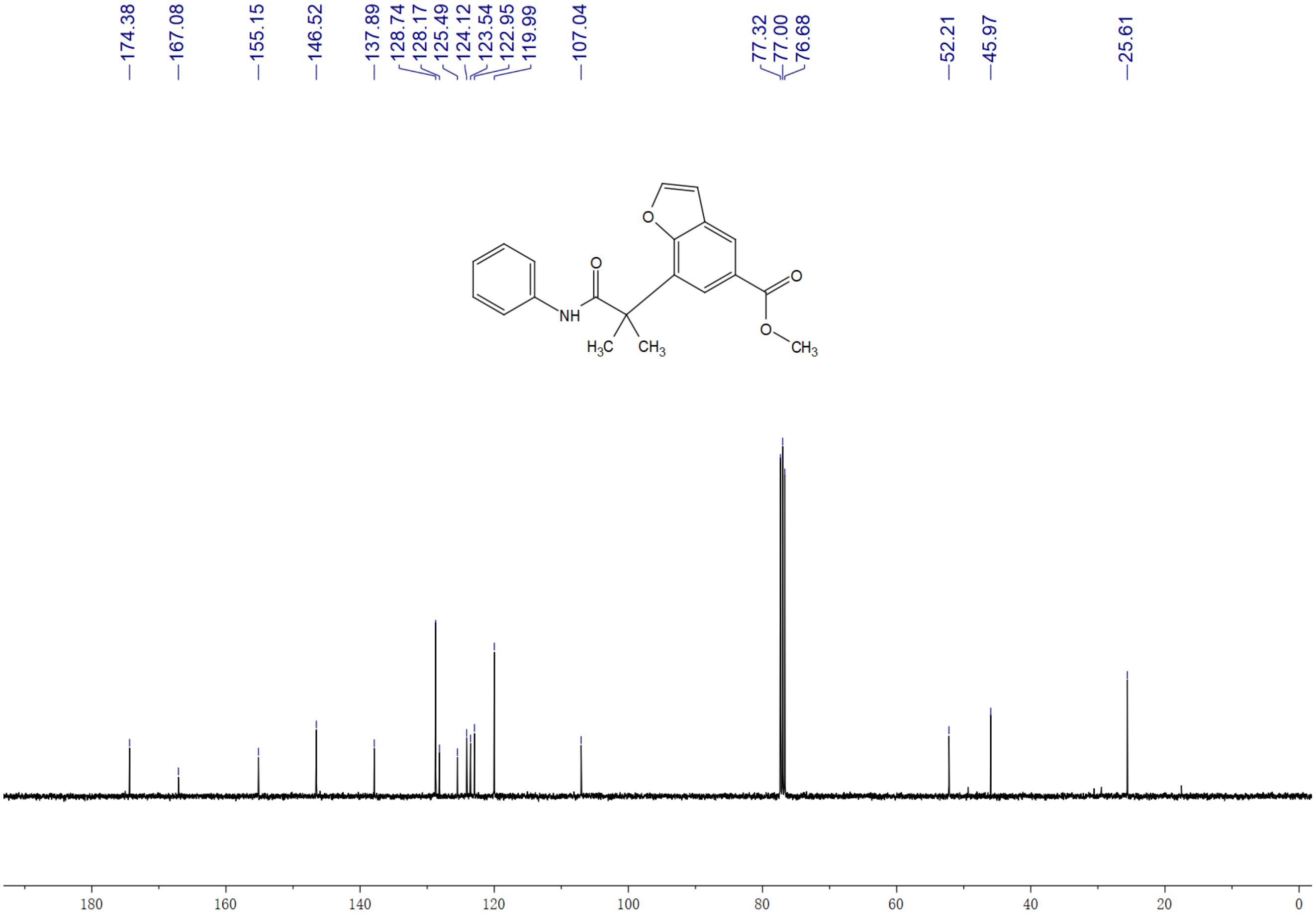

Methyl-7-(2-methyl-1-oxo-1-(phenylamino)propan-2-yl)benzo[b]thiophene-5-carboxylate, **3ad**, ^{1}H NMR (400 MHz, $CDCl_3$) and ^{13}C NMR (100 MHz, $CDCl_3$)

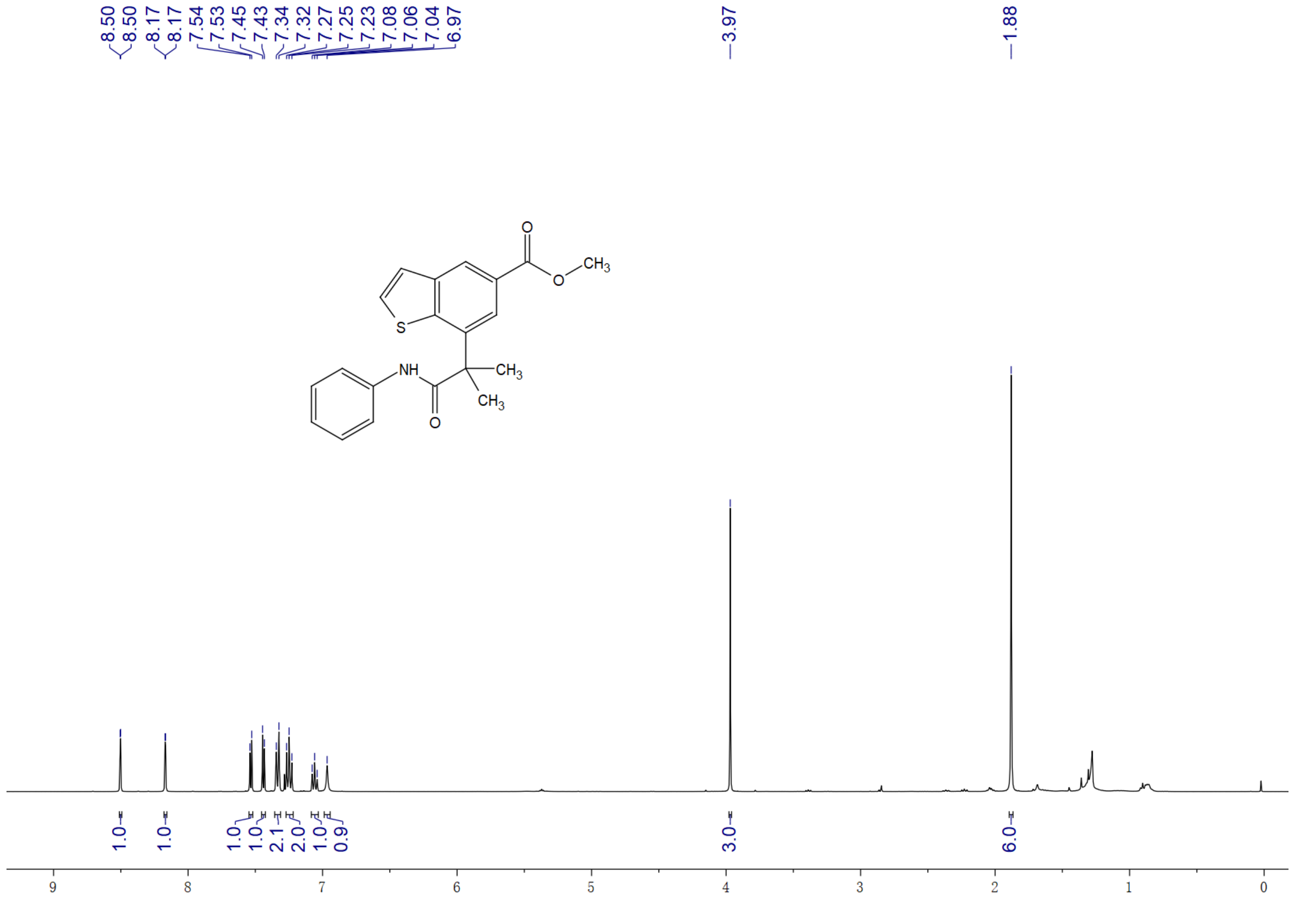

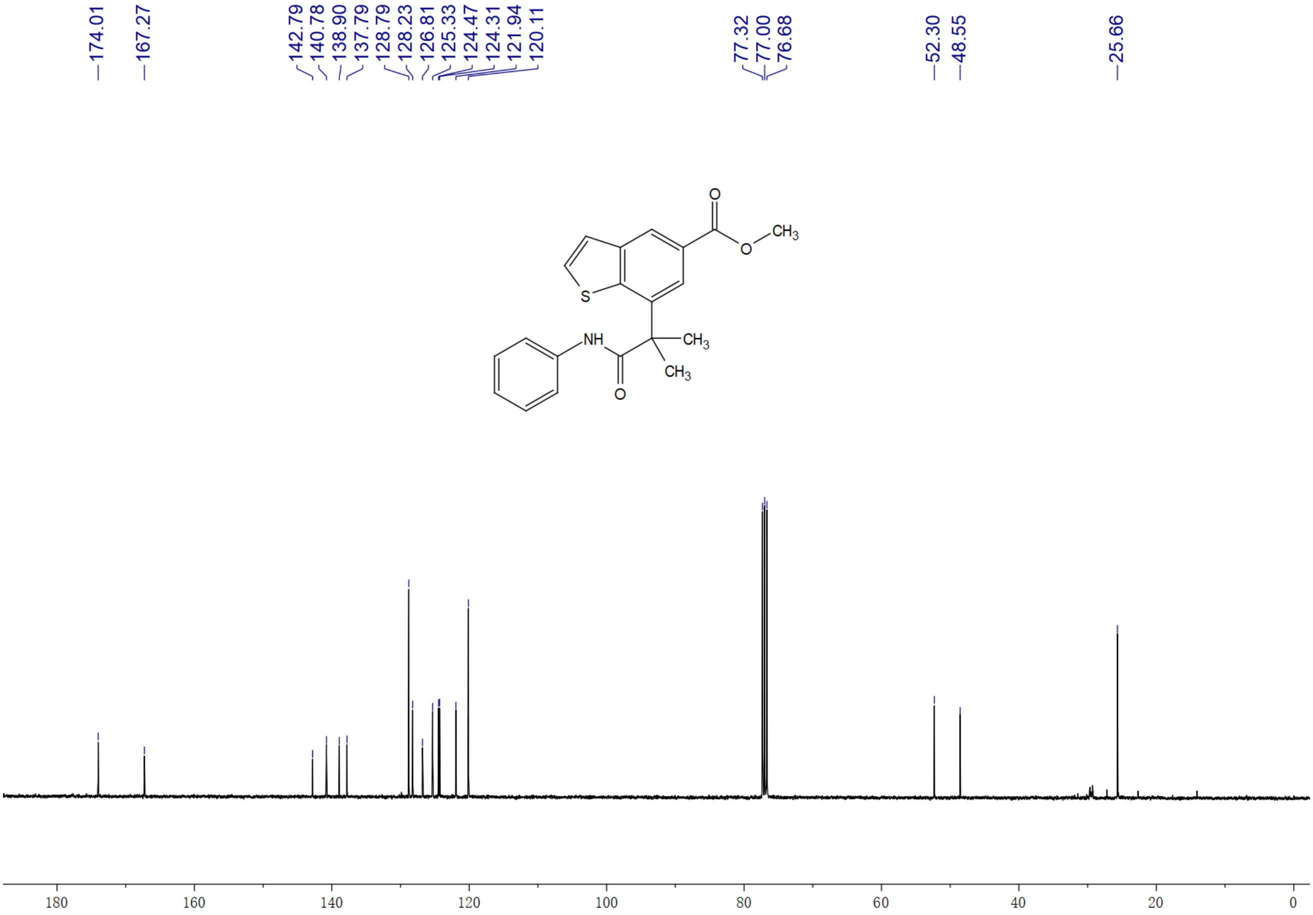

3-(1-((4-Methoxyphenyl)amino)-2-methyl-1-oxopropan-2-yl)-2-methylbenzoic acid, **3ae**, ^{1}H NMR (500 MHz, DMSO-d_6) and ^{13}C NMR (125 MHz, DMSO-d_6)

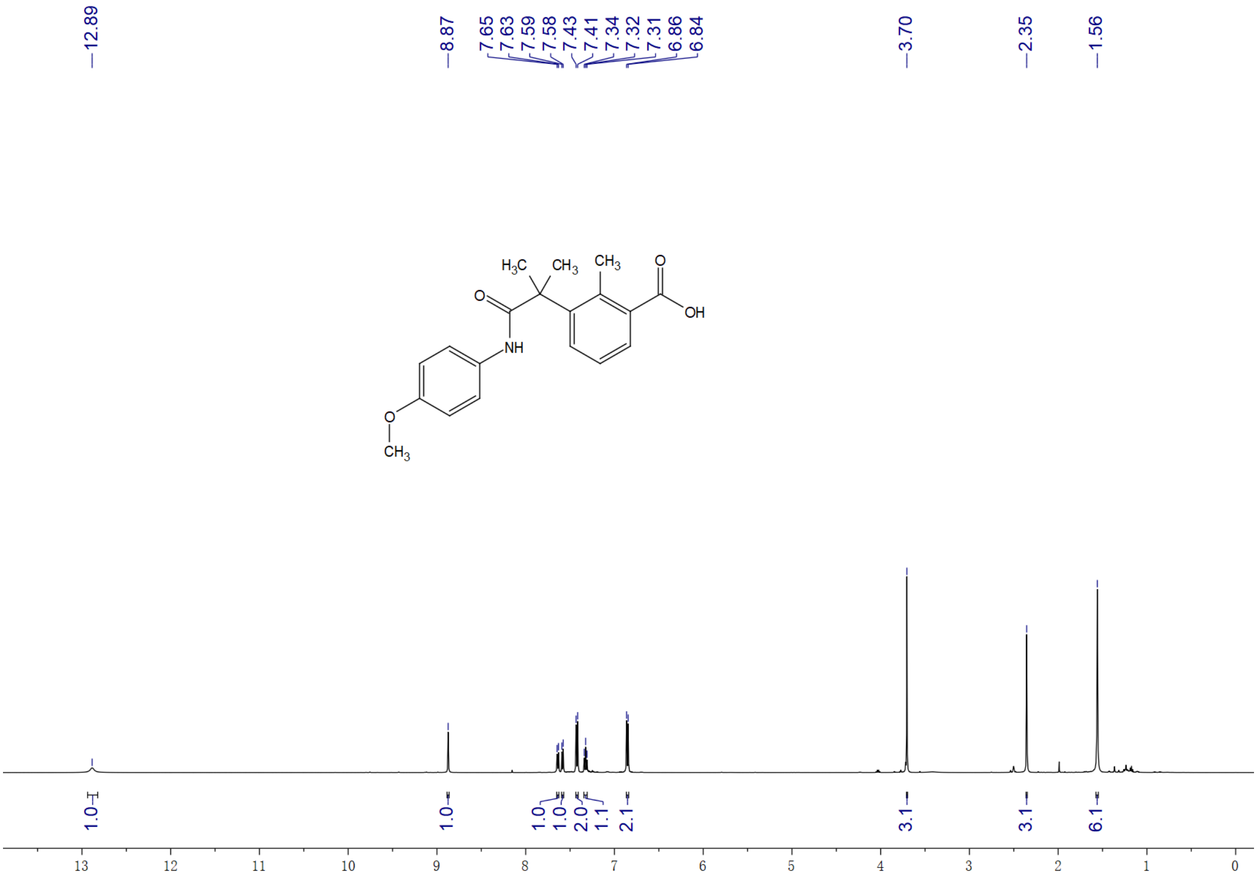

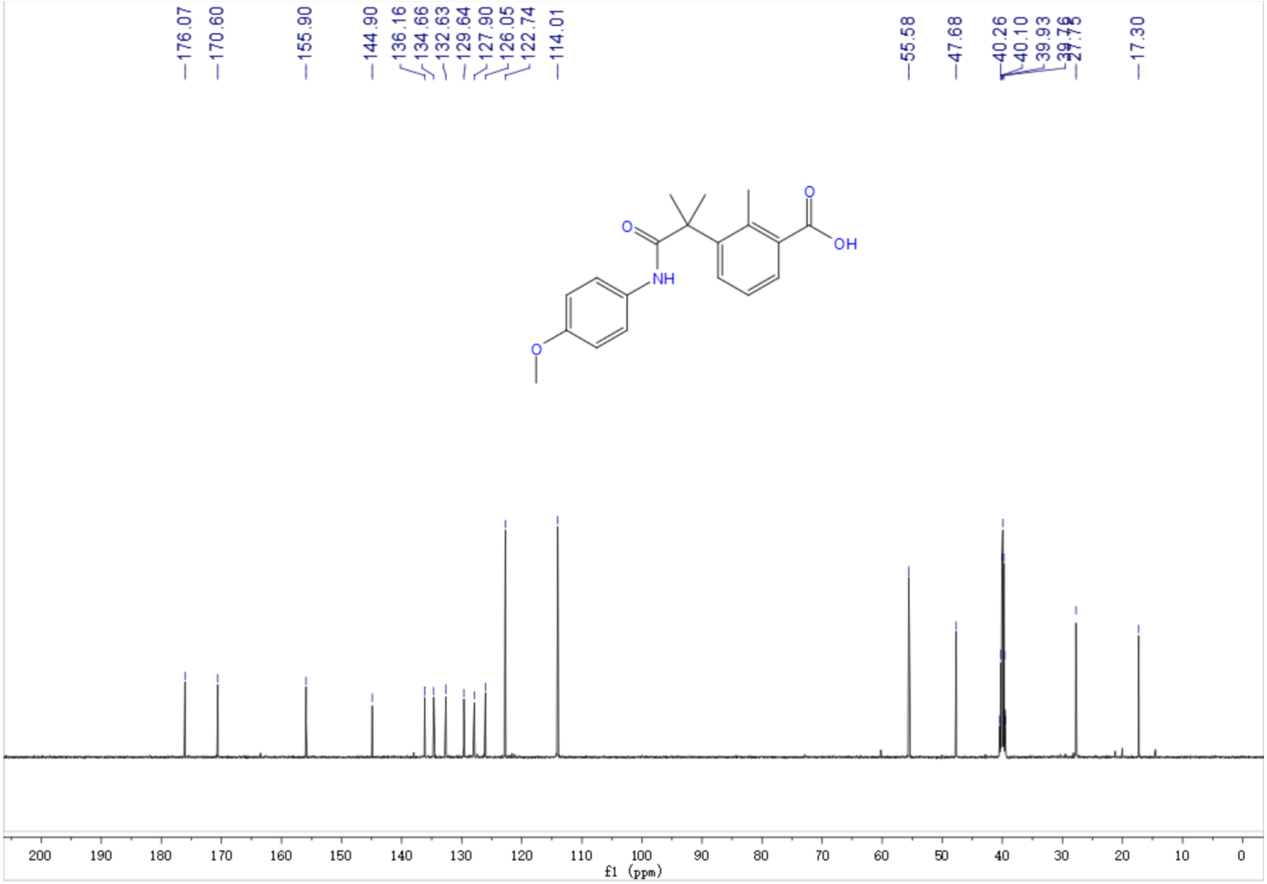

2-Methyl-3-(2-methyl-1-oxo-1-((4-(trifluoromethyl)phenyl)amino)propan-2-yl)benzoic acid, **3af**, ^{1}H NMR (500 MHz, DMSO-d_6), ^{13}C NMR (125 MHz, DMSO-d_6) and ^{19}F NMR (376 MHz, $CDCl_3$)

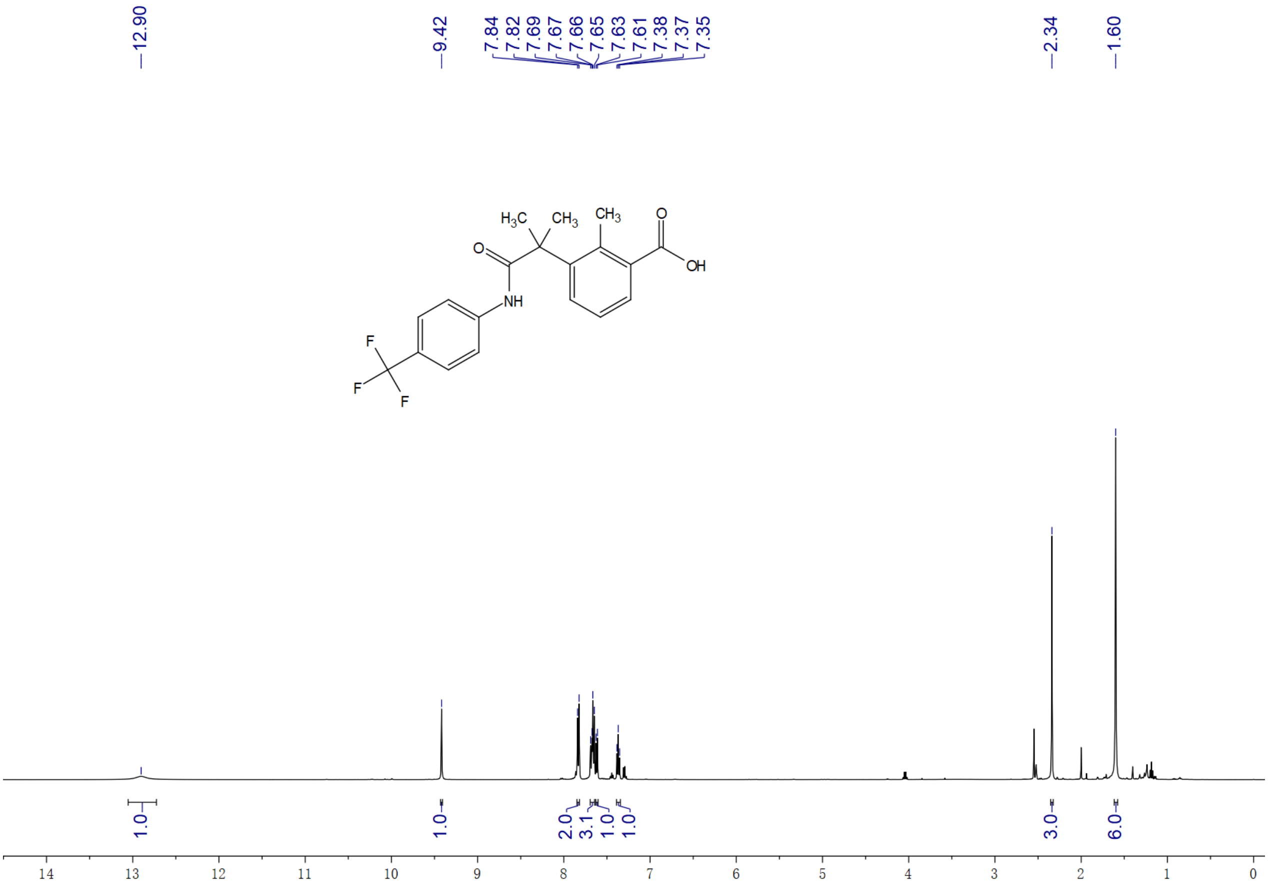

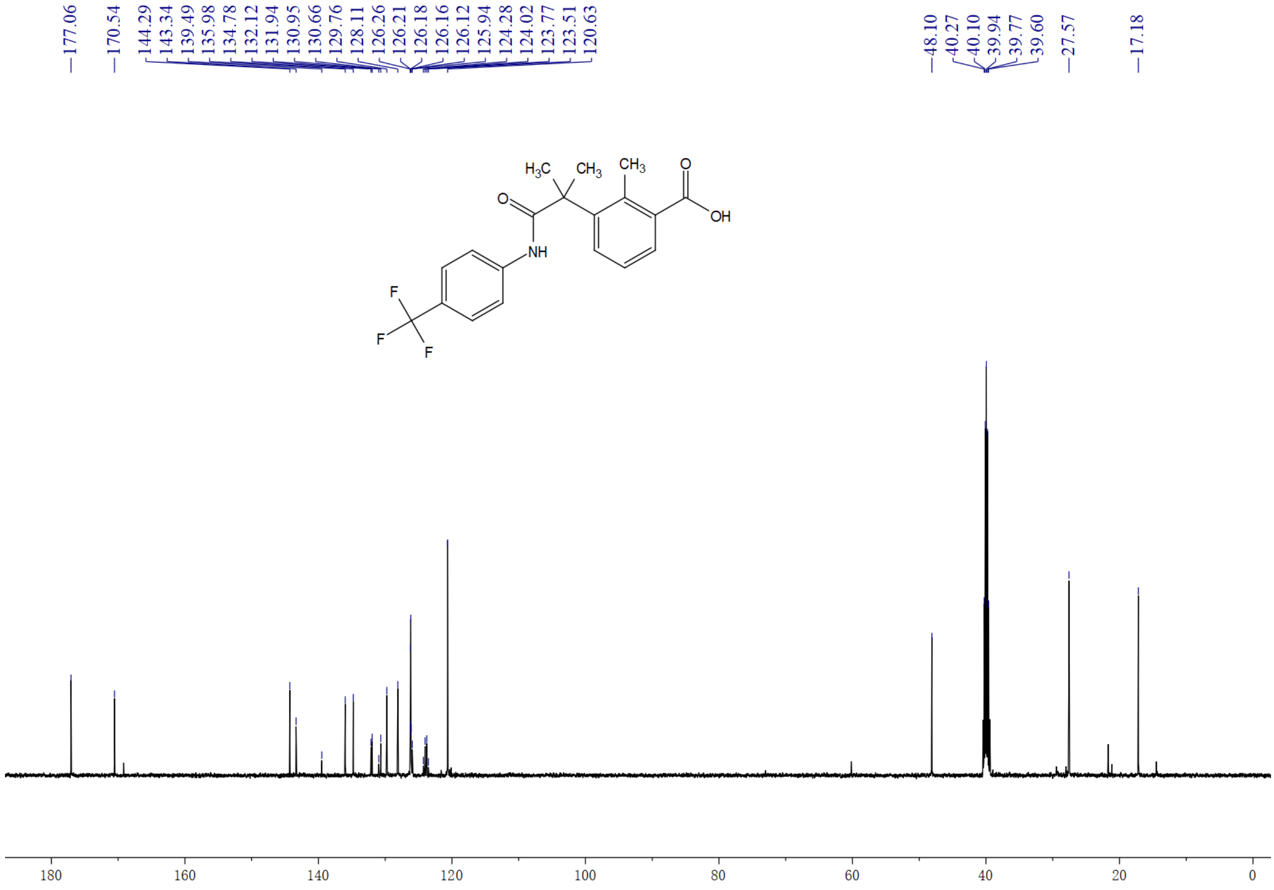

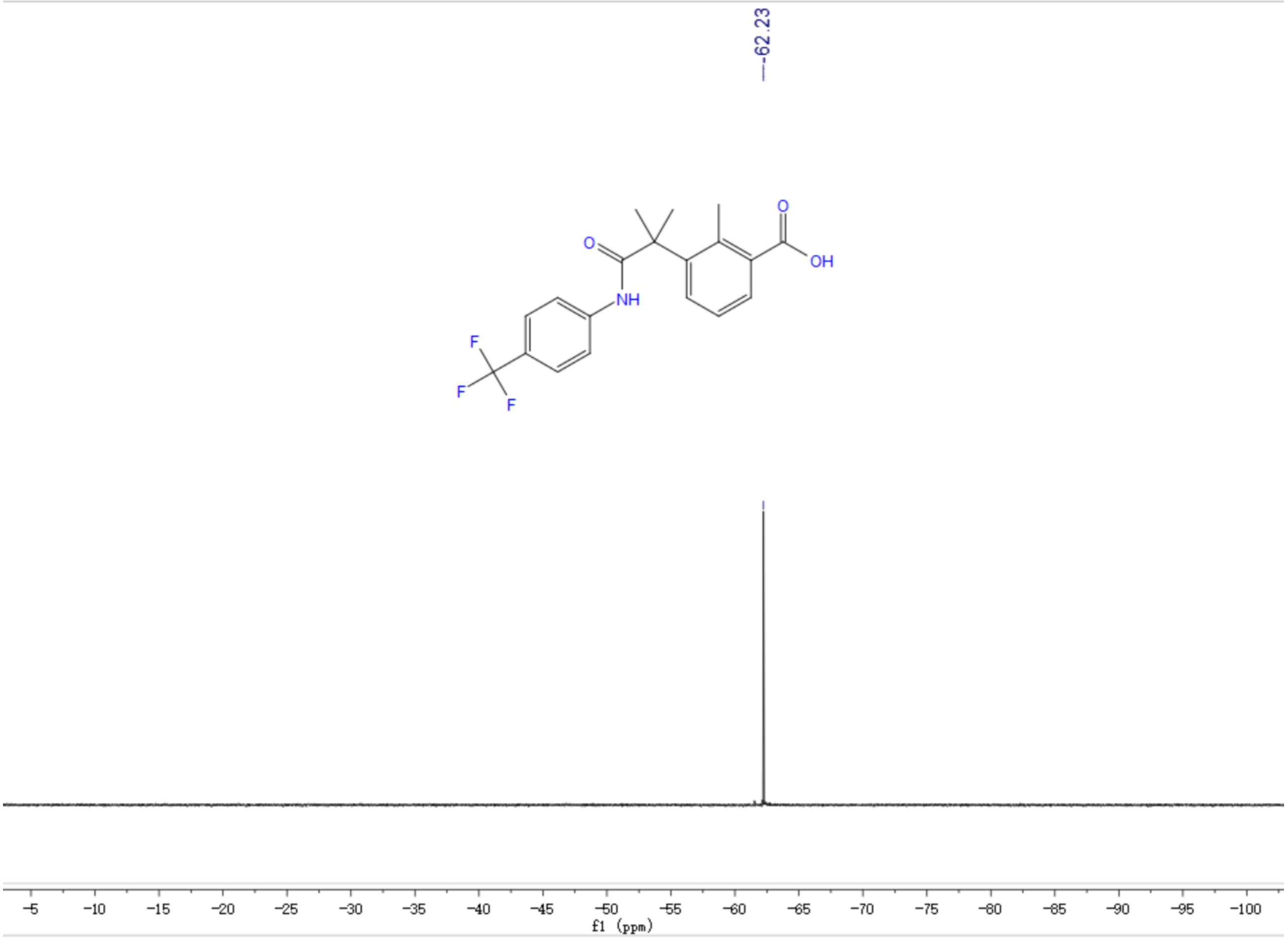

-62.23
O
OH
O
NH
F
F
F
f1 (ppm)

Methyl-3-(1-([1,1'-biphenyl]-4-ylamino)-2-methyl-1-oxopropan-2-yl)-2-methylbenzoate, **3ag**, ^{1}H NMR (500 MHz, $CDCl_3$) and ^{13}C NMR (125 MHz, $CDCl_3$)

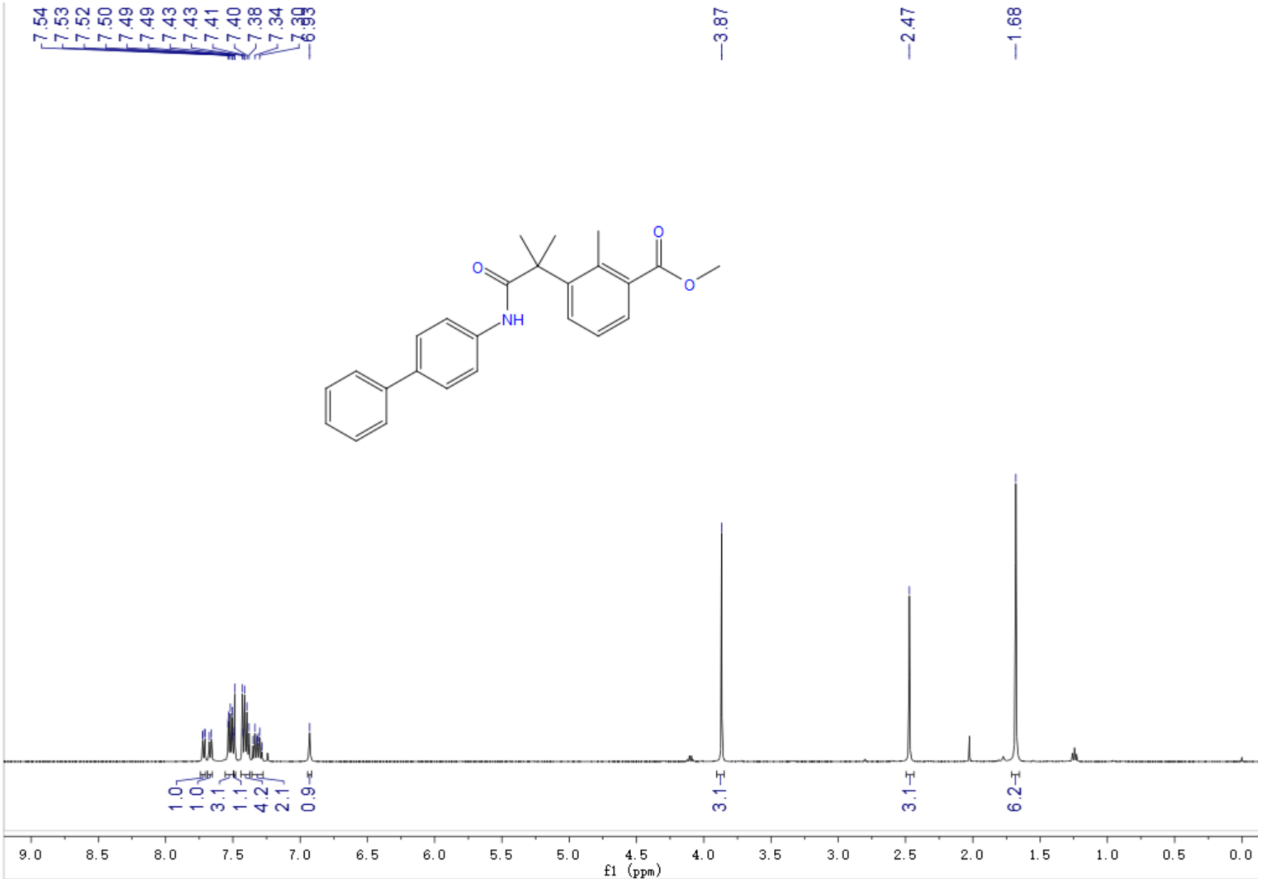

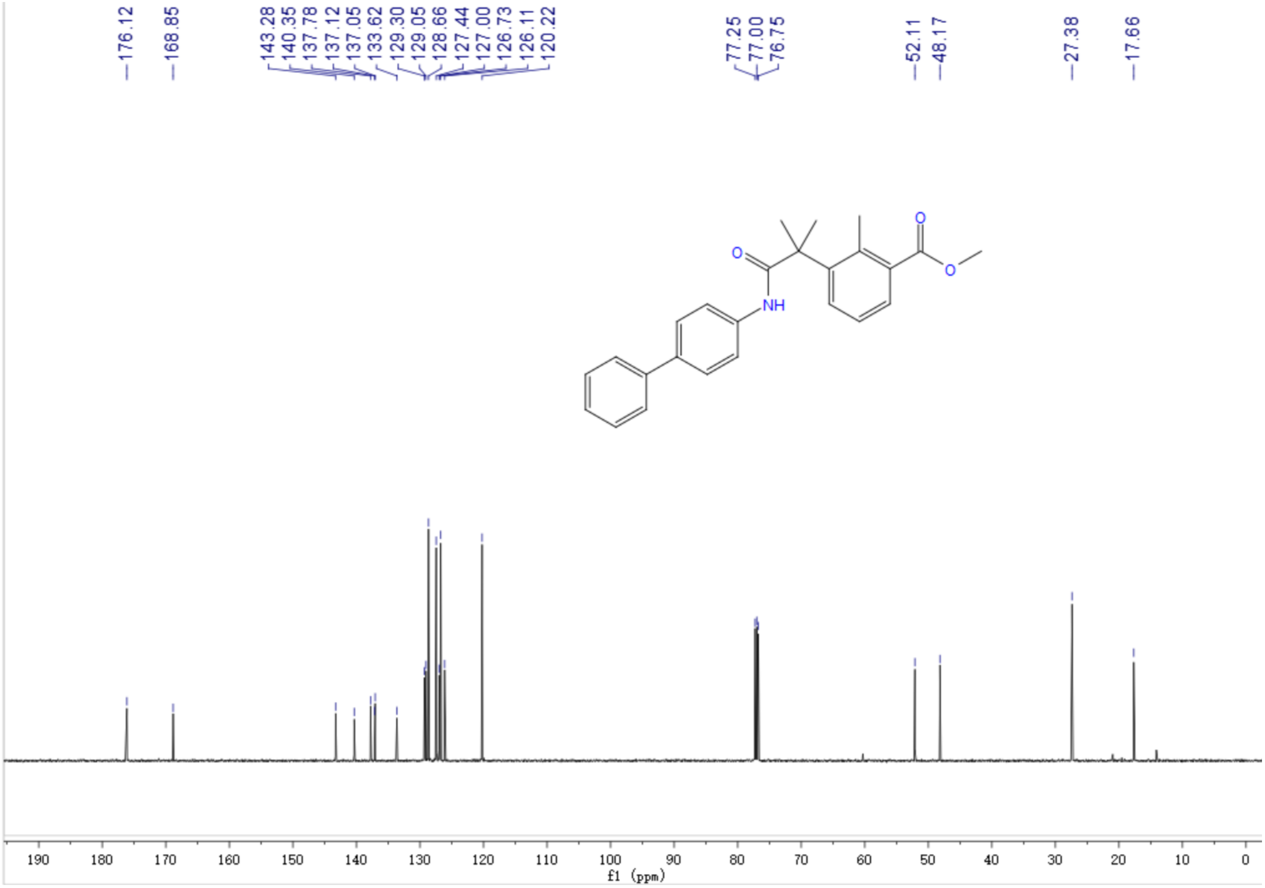

3-(1-((2,6-Difluorophenyl)amino)-2-methyl-1-oxopropan-2-yl)-2-methylbenzoic acid, **3ah**, ^{1}H NMR (500 MHz, DMSO-d_6) and ^{13}C NMR (125 MHz, DMSO-d_6)

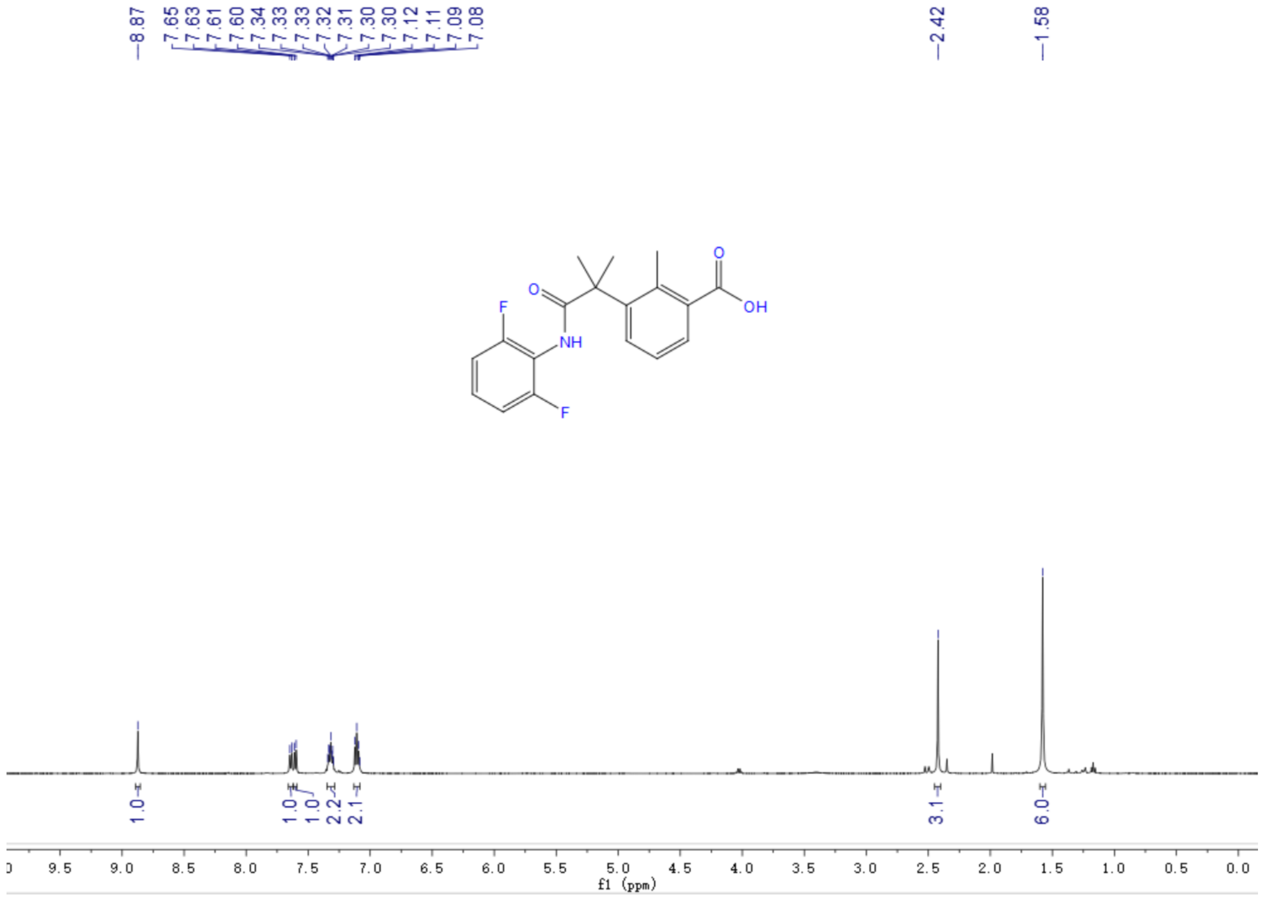

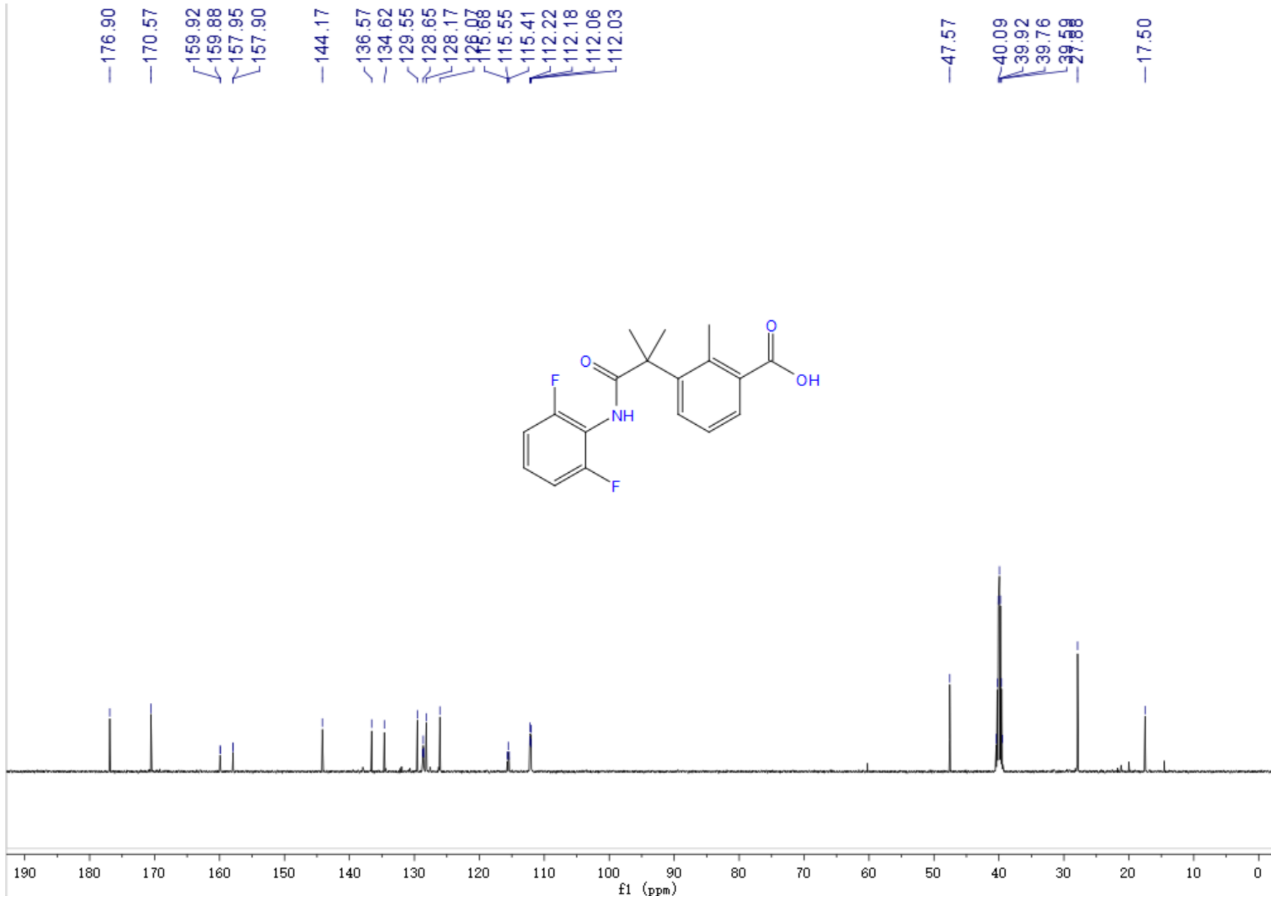

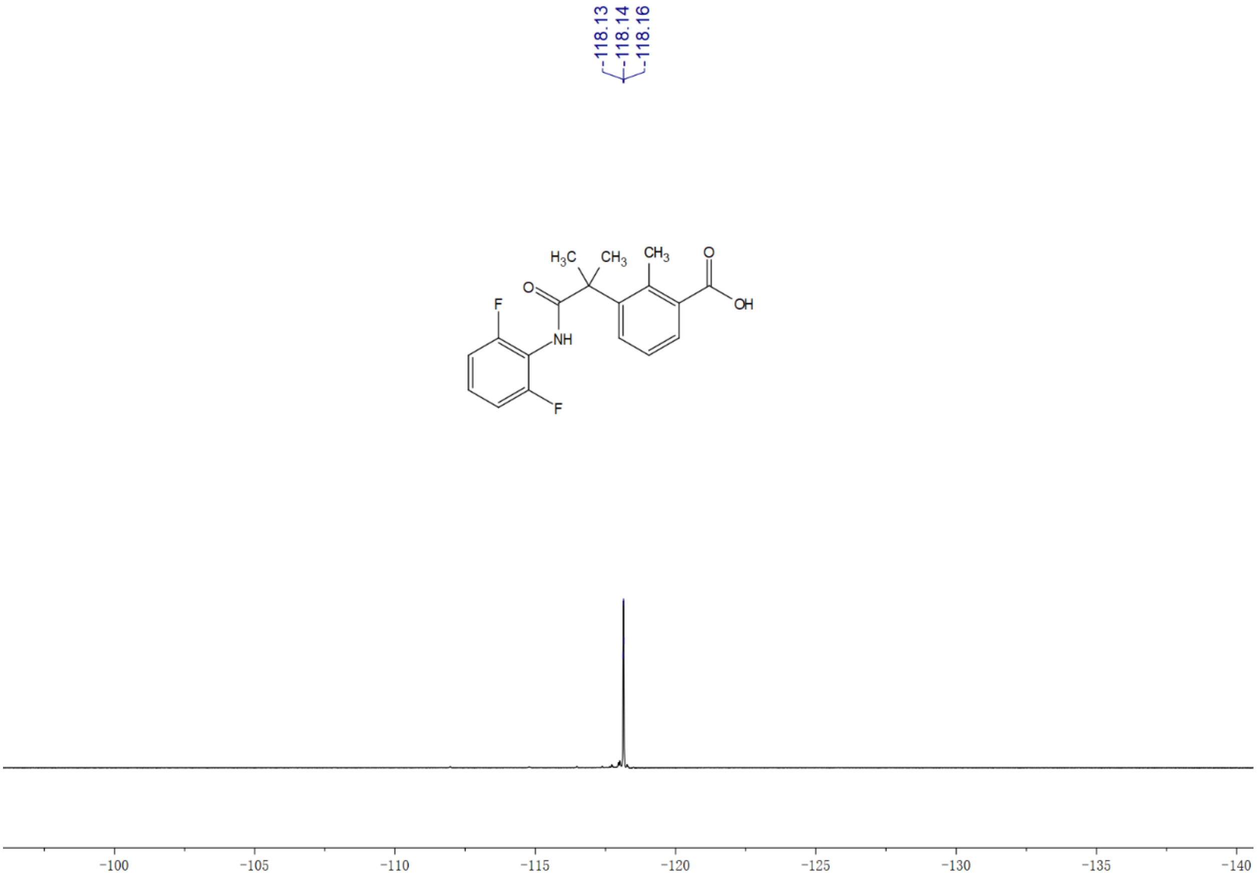
-118.13
-118.14
-118.16
H3C
CH3
CH3
O
OH
O
F
NH
F
-100
-105
-110
-115
-120
-125
-130
-135
-140

3-(1-(Tert-butylamino)-2-methyl-1-oxopropan-2-yl)-2-methylbenzoic acid, **3ai**, ^{1}H NMR (500 MHz, DMSO-d_6) and ^{13}C NMR (125 MHz, DMSO-d_6)

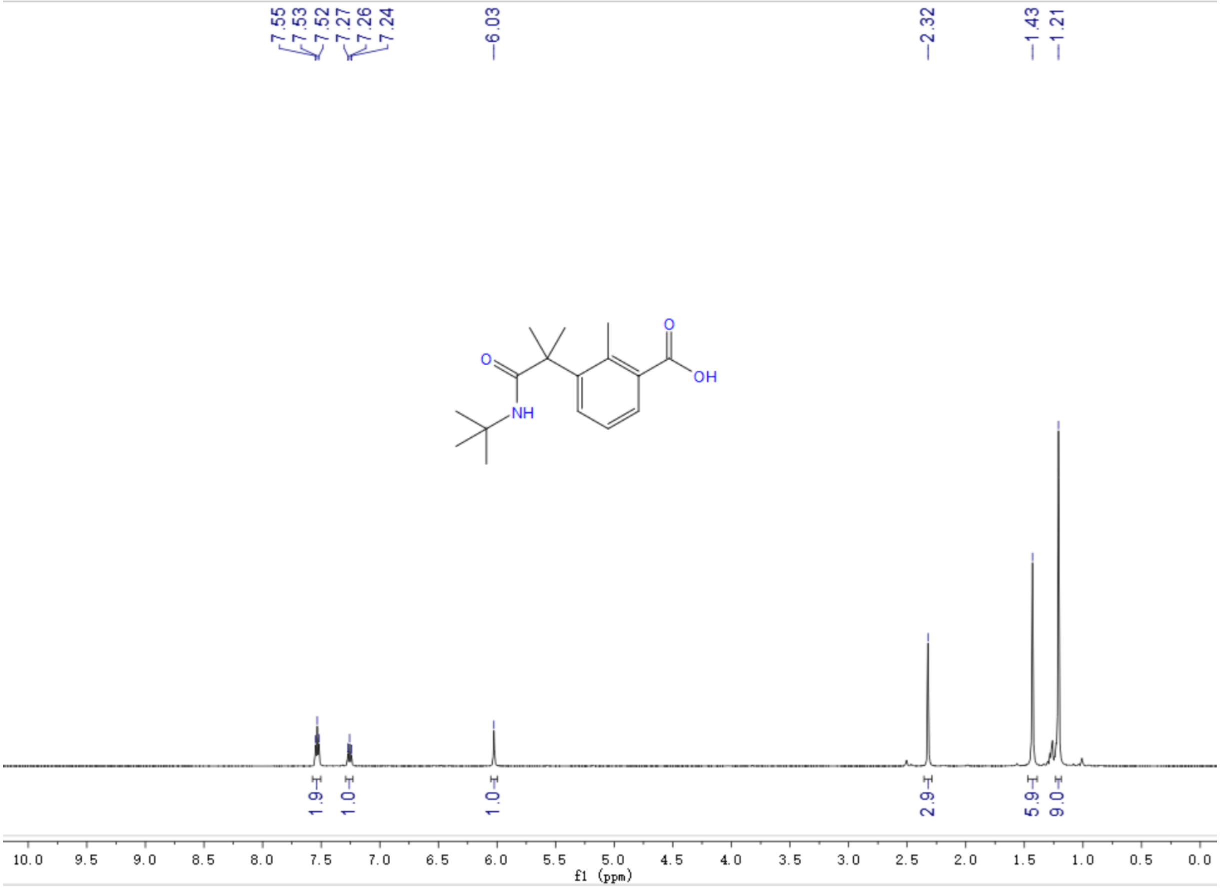

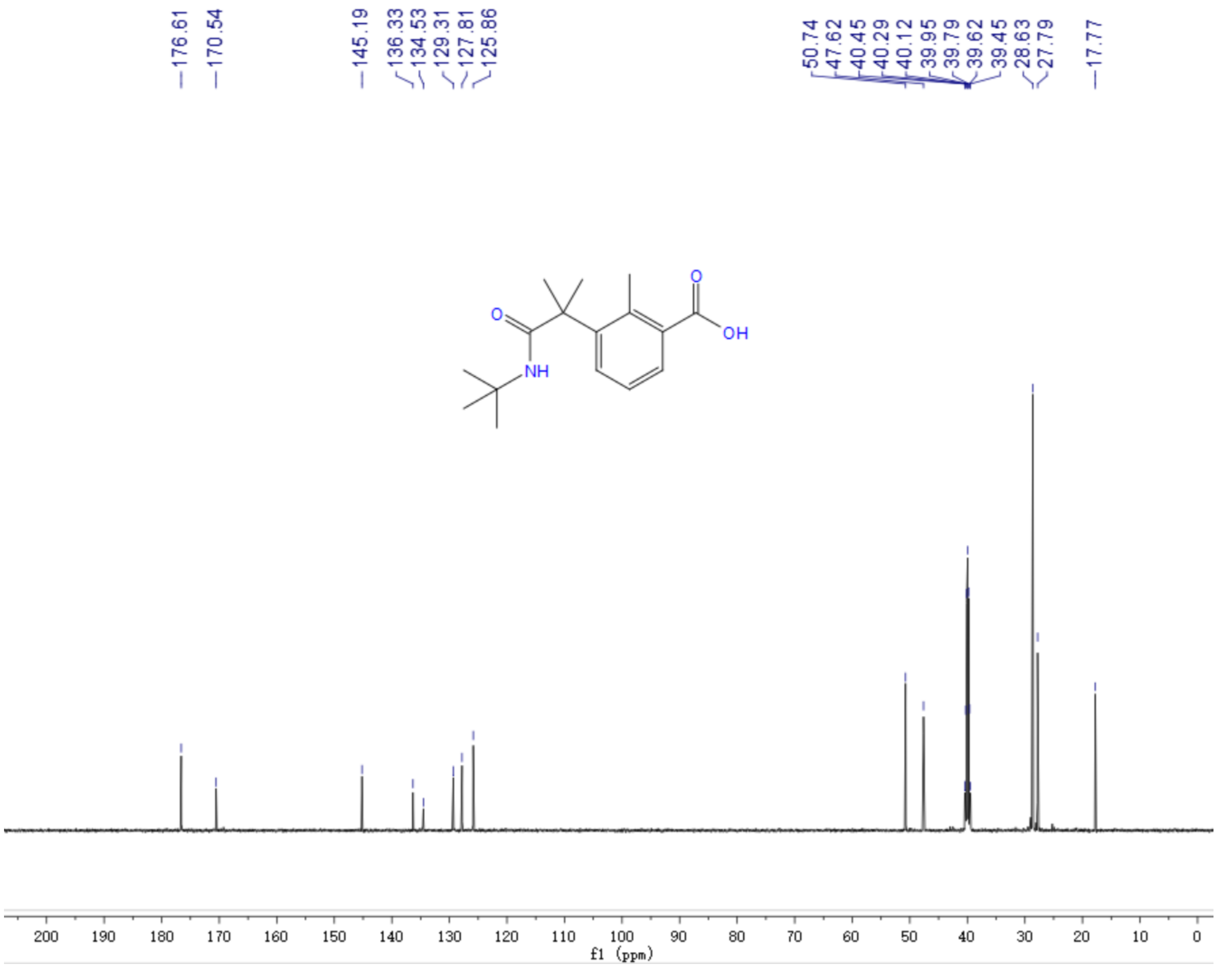

Methyl-3-(1-(methoxy(methyl)amino)-2-methyl-1-oxopropan-2-yl)-2-methylbenzoate, **3aj**, ^{1}H NMR (400 MHz, $CDCl_3$) and ^{13}C NMR (100 MHz, $CDCl_3$)

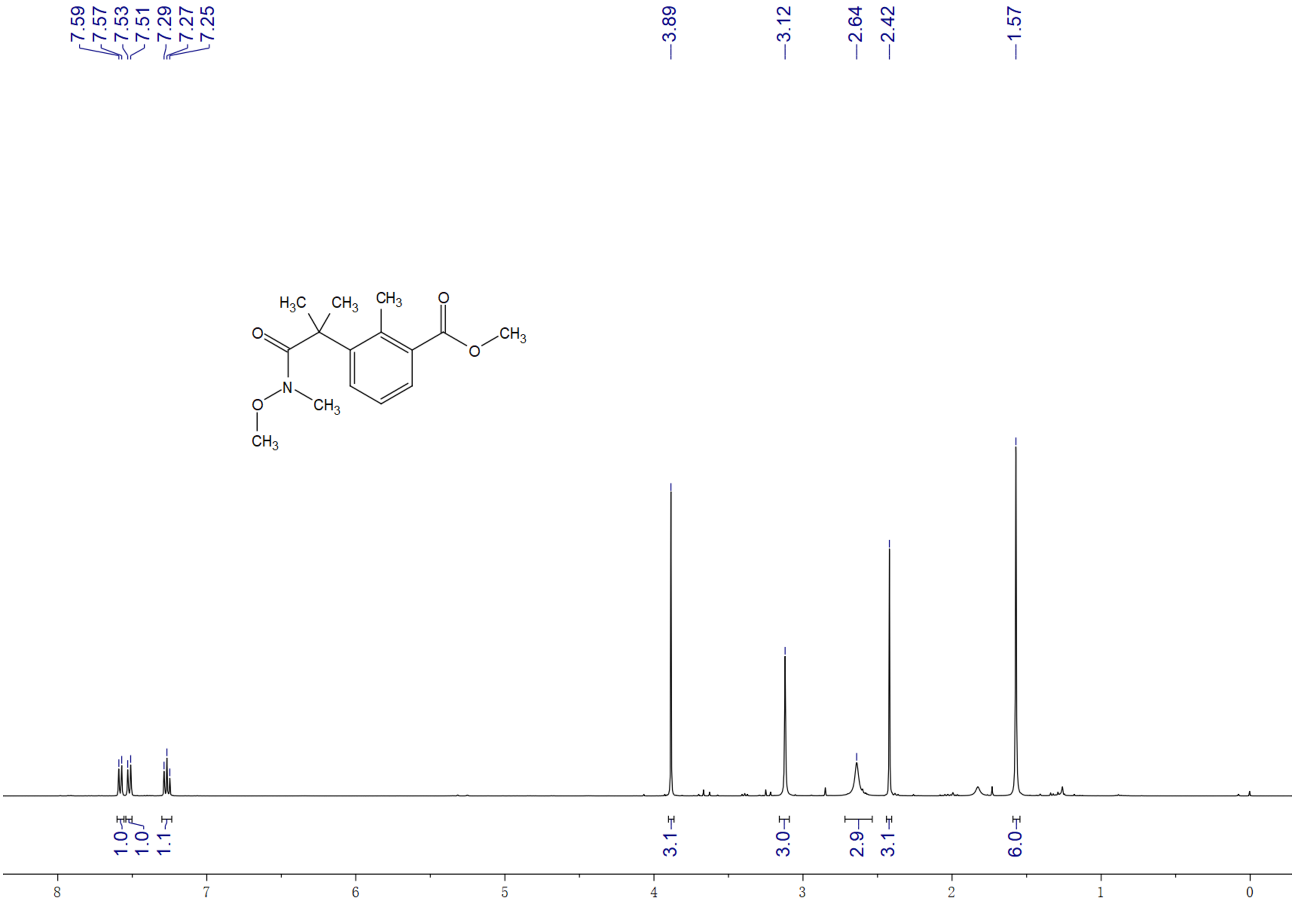

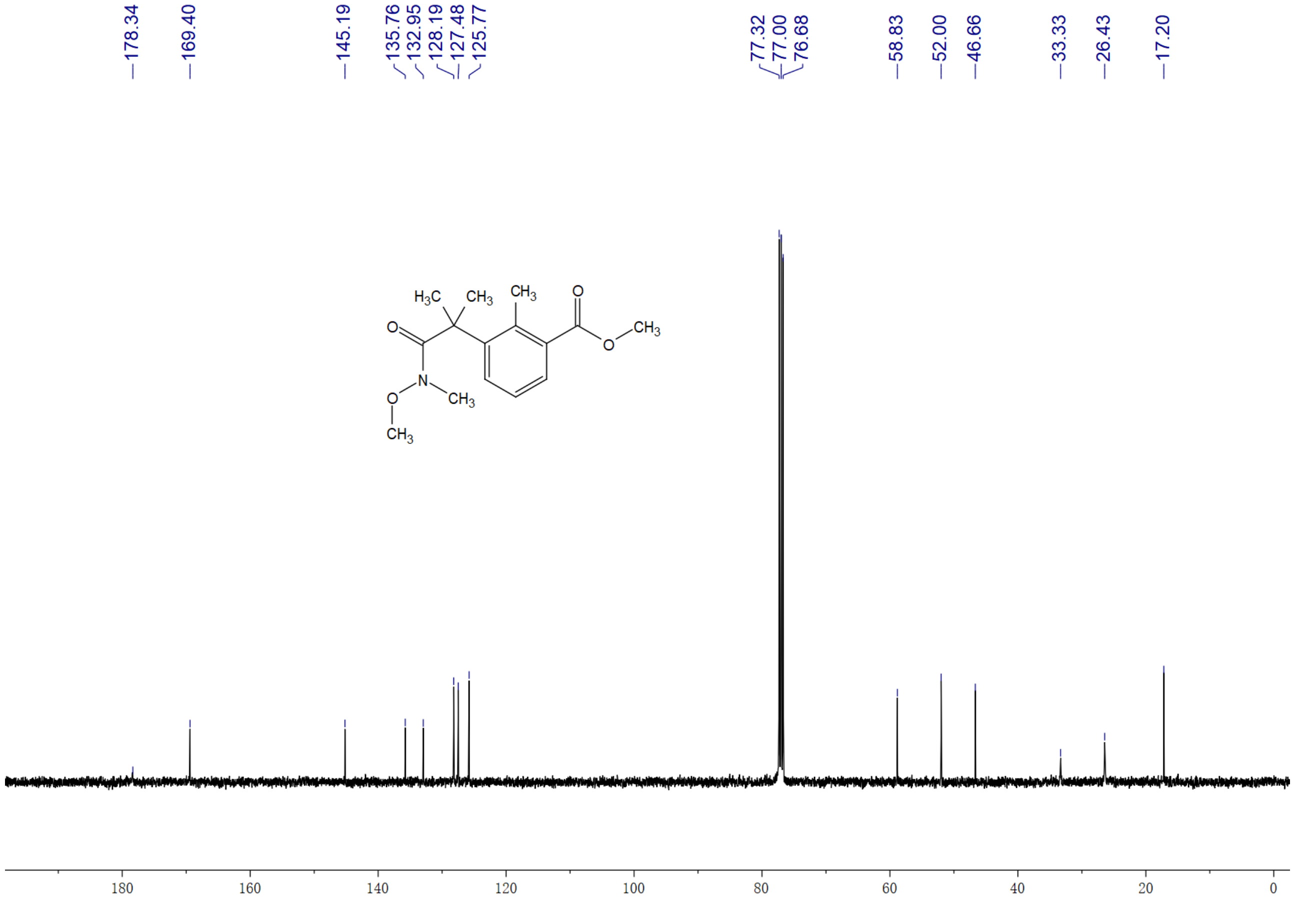

Methyl-3-(1-((2-methoxy-2-oxoethyl)amino)-2-methyl-1-oxopropan-2-yl)-2-methylbenzoate, **3ak**, ^1H NMR (400 MHz, $CDCl_3$) and ^{13}C NMR (100 MHz, $CDCl_3$)

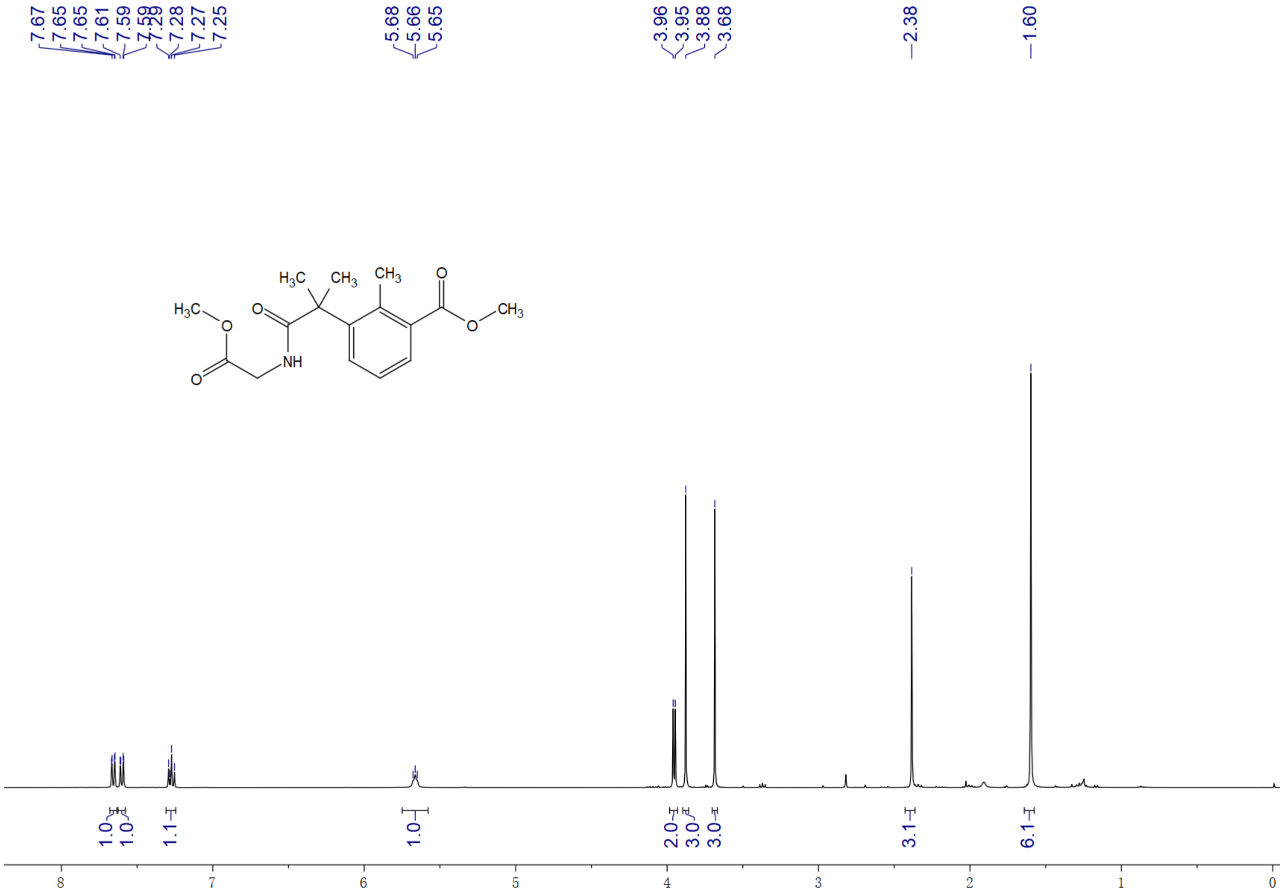

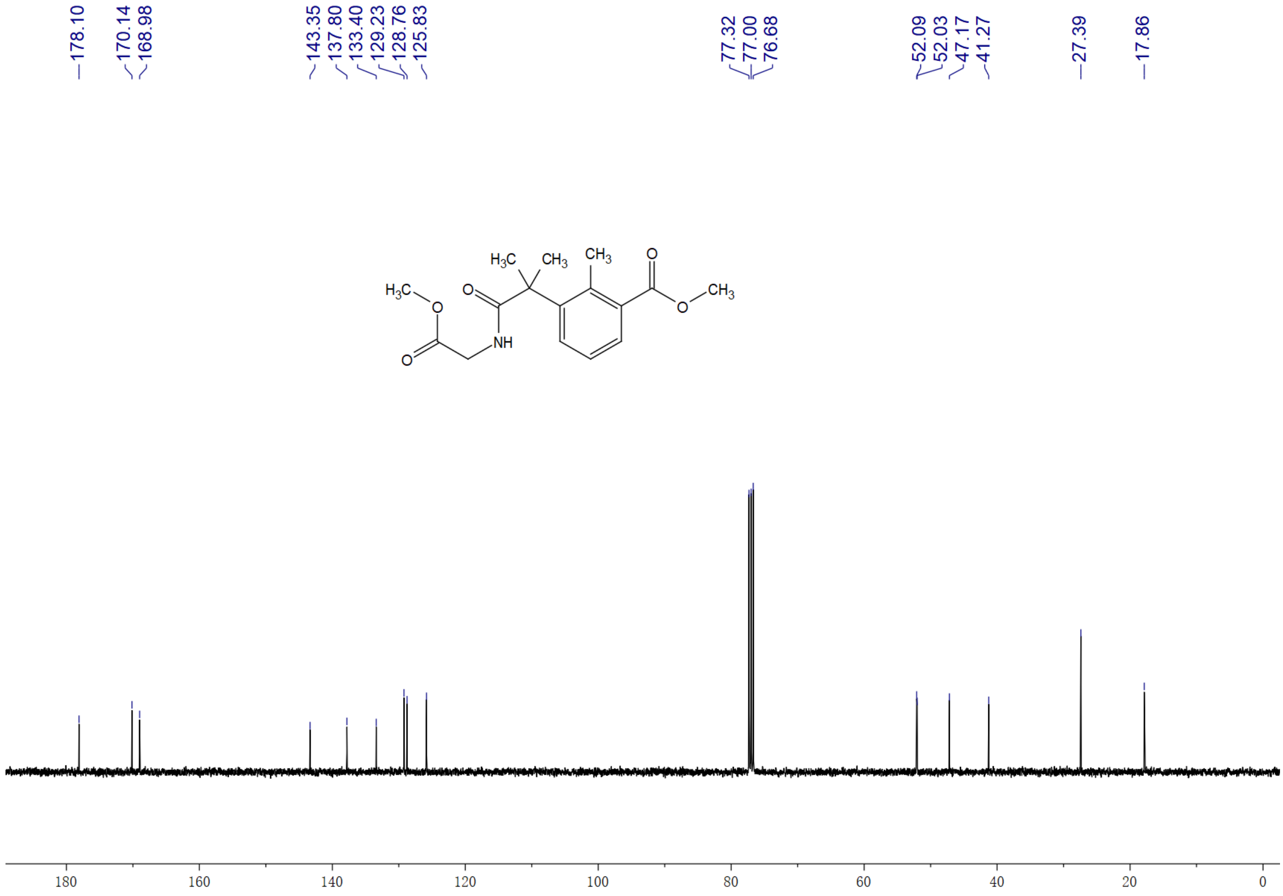

Methyl-3-(1-((1-methoxy-3-methyl-1-oxobutan-2-yl)amino)-2-methyl-1-oxopropan-2-yl)-2-methylbenzoate, **3al**, ^{1}H NMR (400 MHz, $CDCl_3$) and ^{13}C NMR (100 MHz, $CDCl_3$)

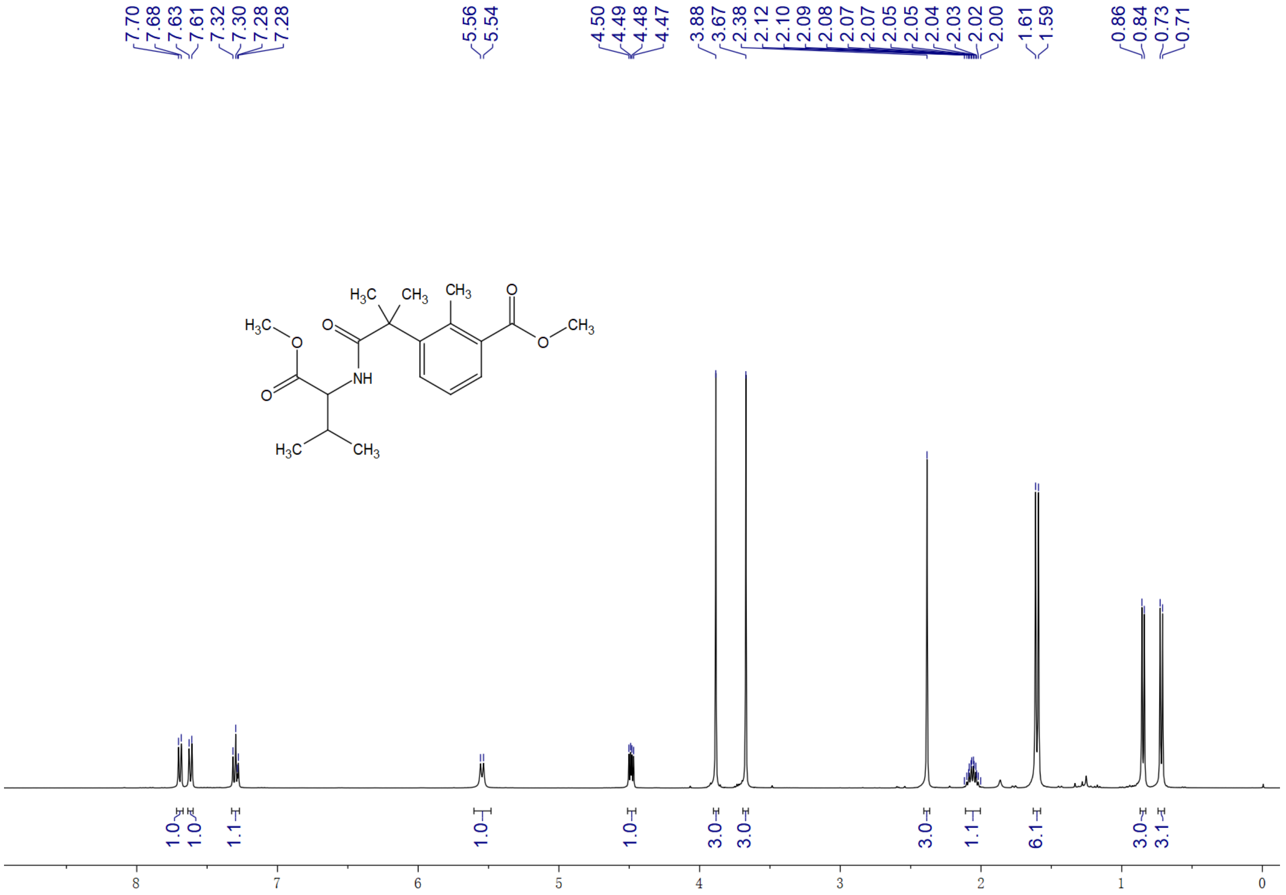

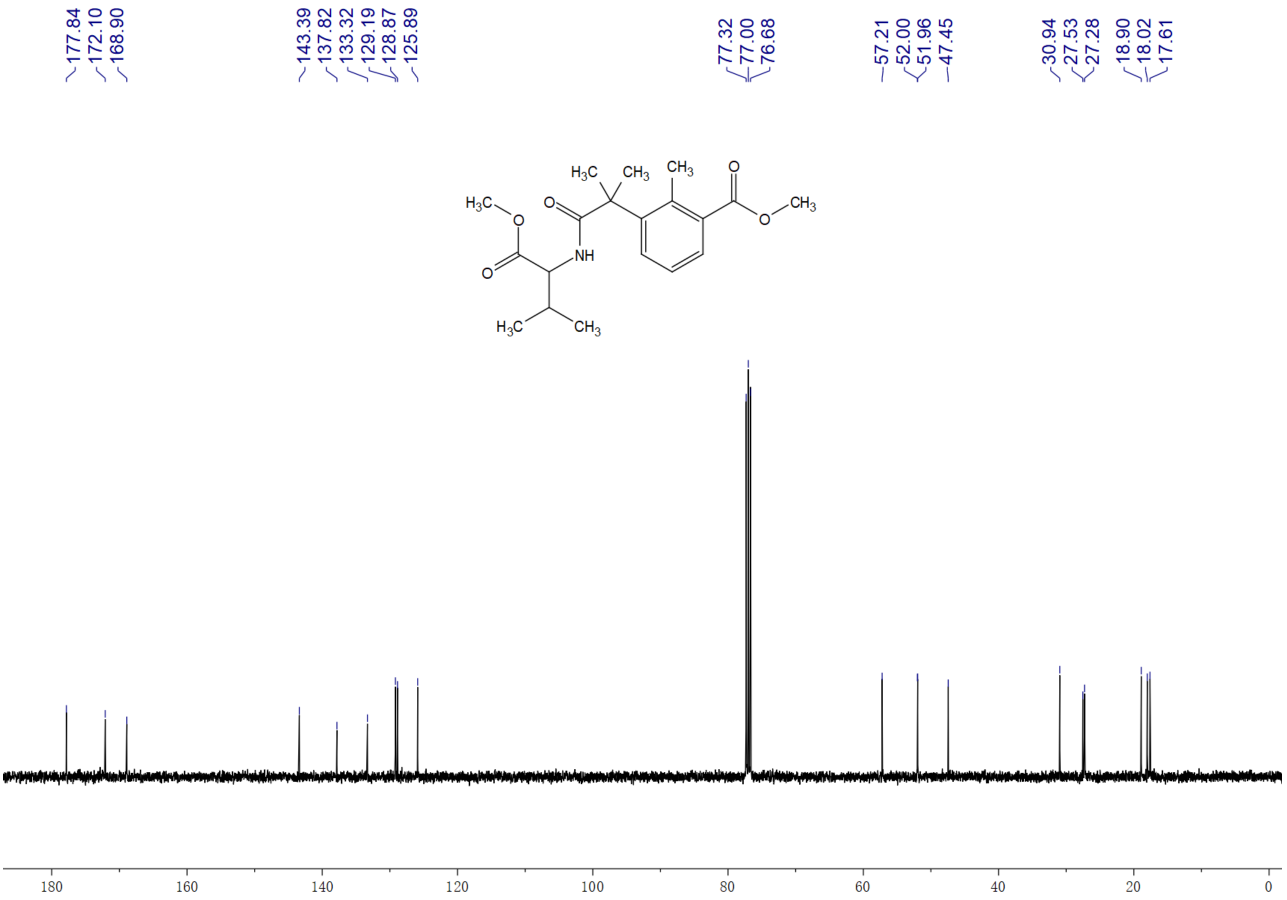

Dimethyl-(2-(3-(methoxycarbonyl)-2-methylphenyl)-2-methylpropanoyl)aspartate, **3am**, ^{1}H NMR (400 MHz, $CDCl_3$) and ^{13}C NMR (125 MHz, $CDCl_3$)

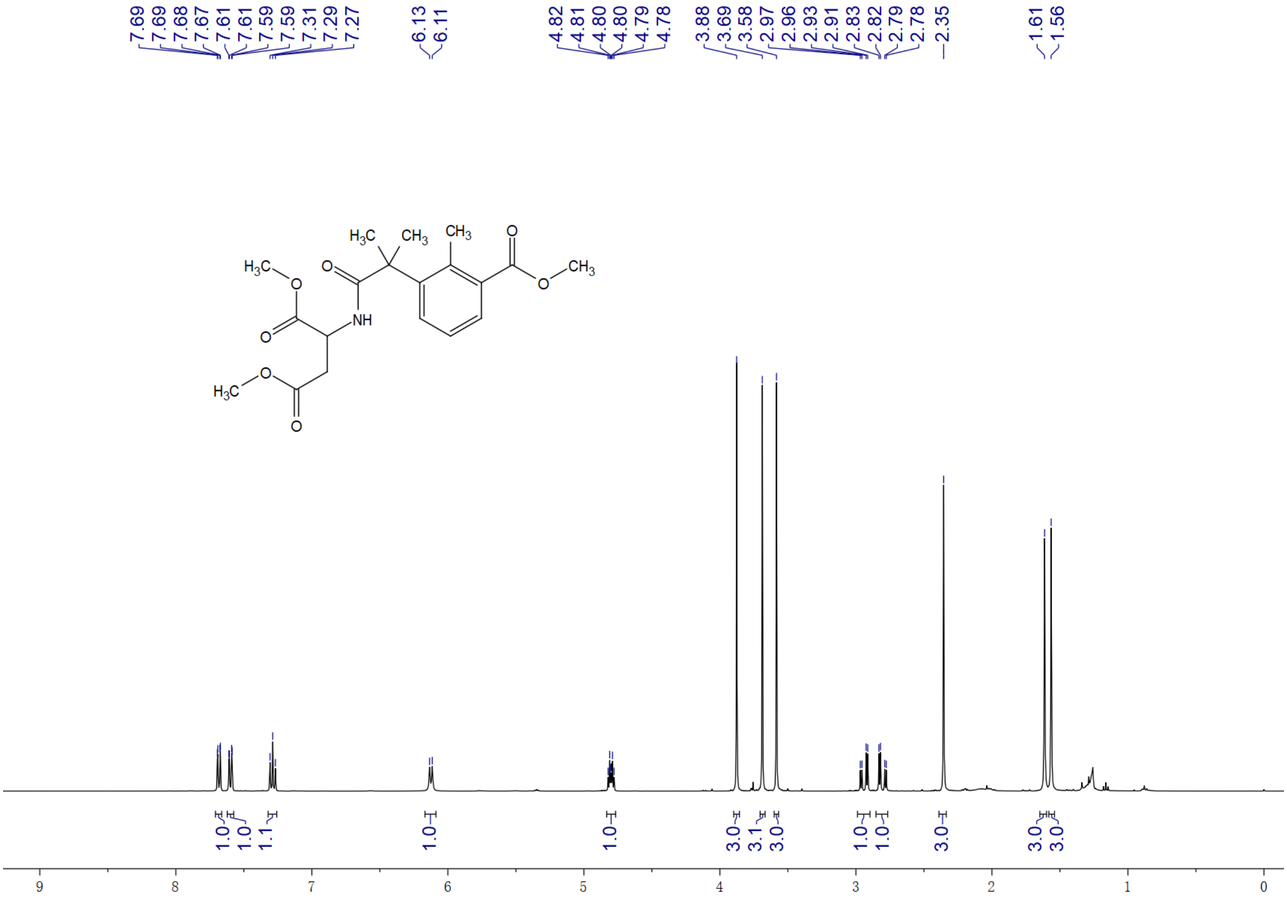

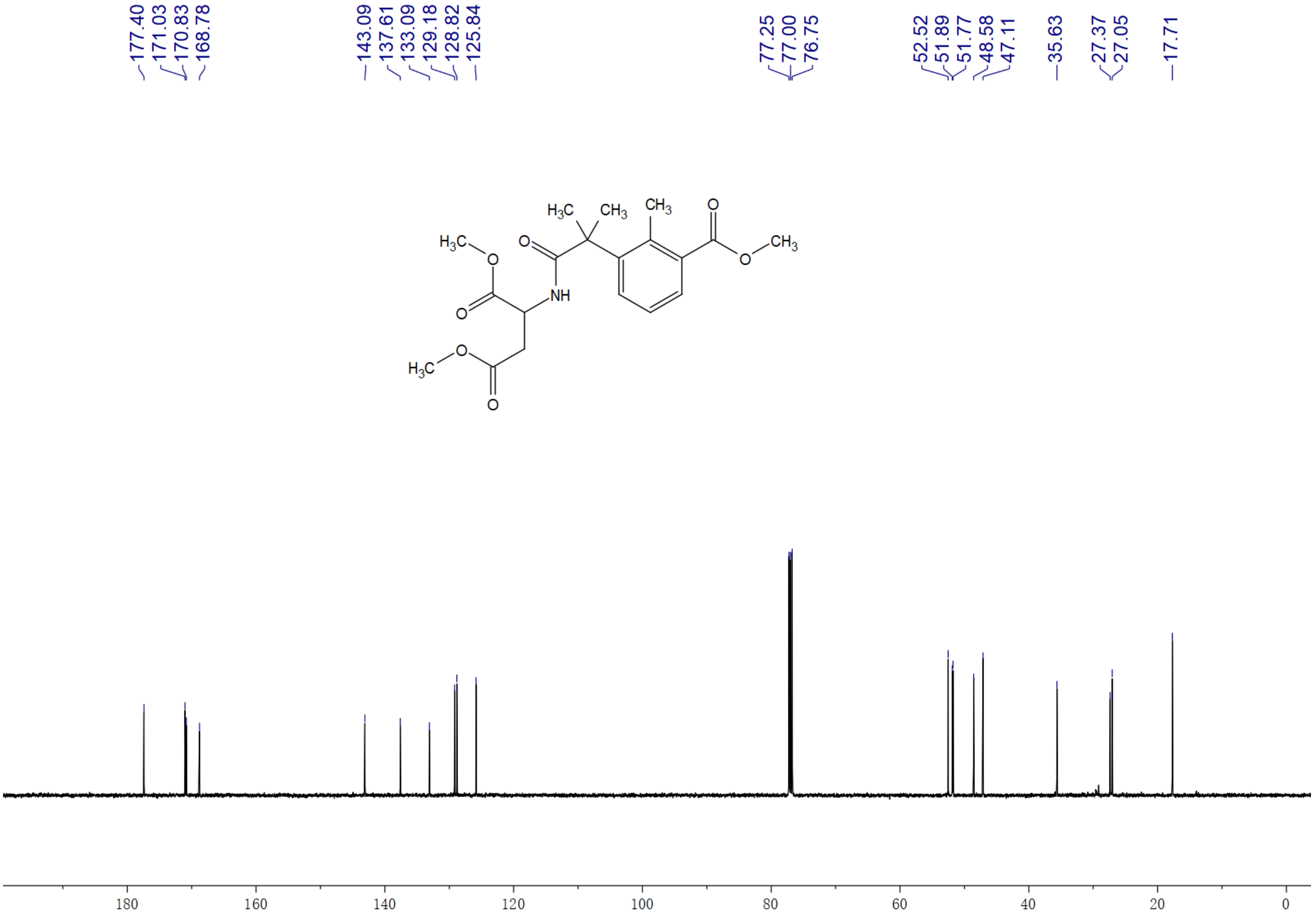

Methyl-3-(1-((3-(tert-butoxy)-1-methoxy-1-oxopropan-2-yl)amino)-2-methyl-1-oxopropan-2-yl)-2-methylbenzoate, **3an**, ^{1}H NMR (400 MHz, $CDCl_3$) and ^{13}C NMR (100 MHz, $CDCl_3$)

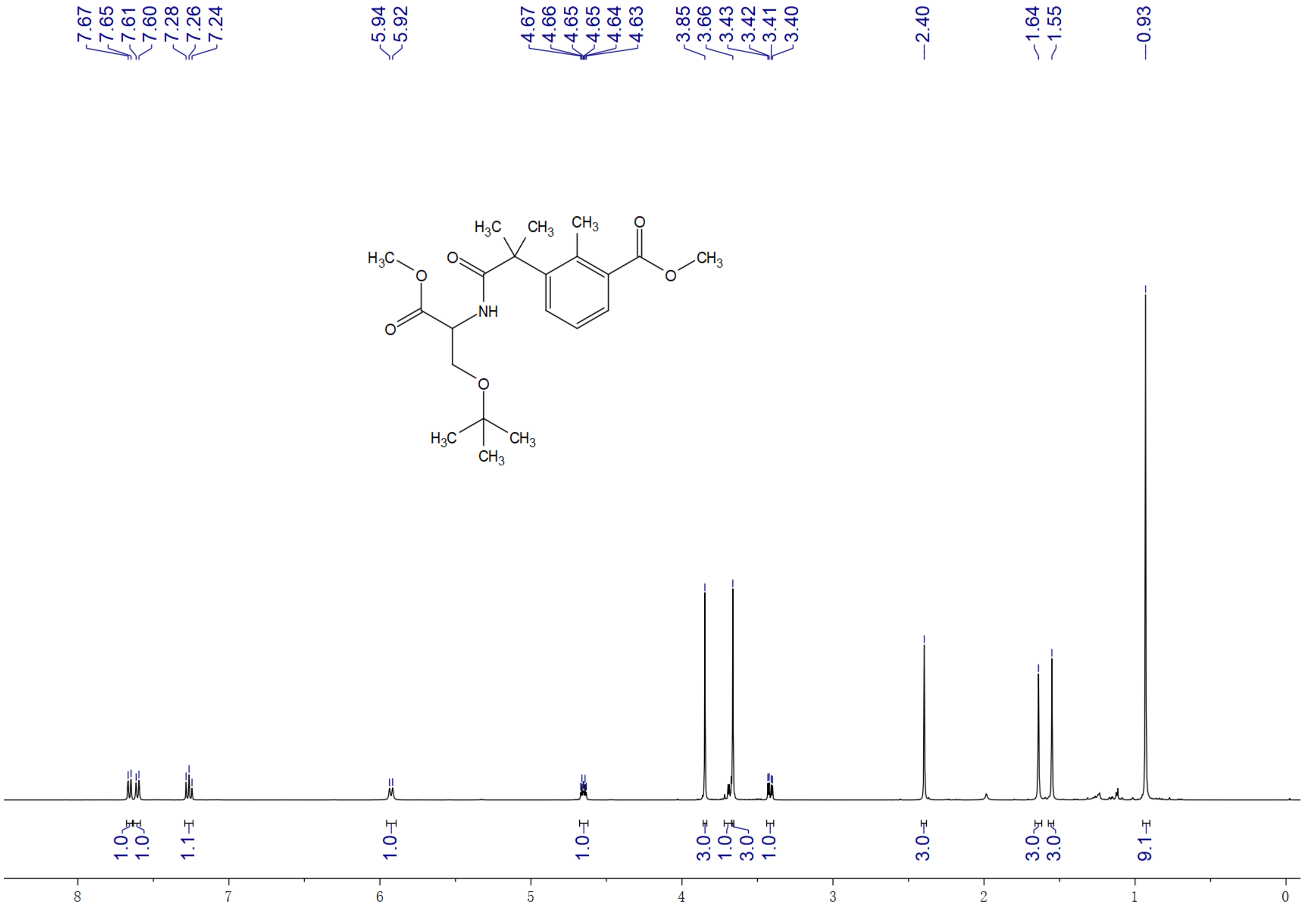

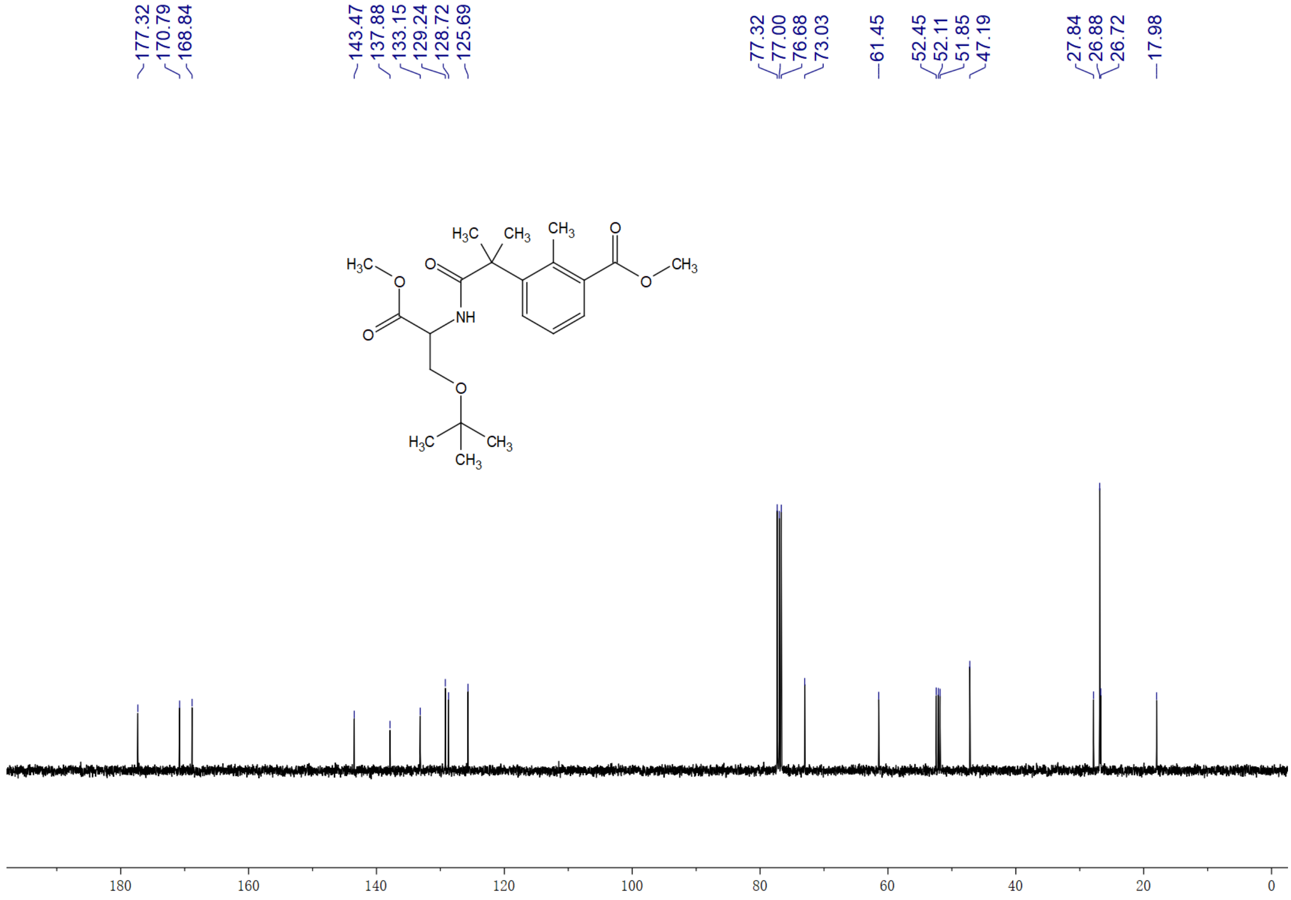

Methyl-3-(1-((1-methoxy-1-oxo-3-phenylpropan-2-yl)amino)-2-methyl-1-oxopropan-2-yl)-2 methylbenzoate, **3ao**, ^{1}H NMR (500 MHz, $CDCl_3$) and ^{13}C NMR (125 MHz, $CDCl_3$)

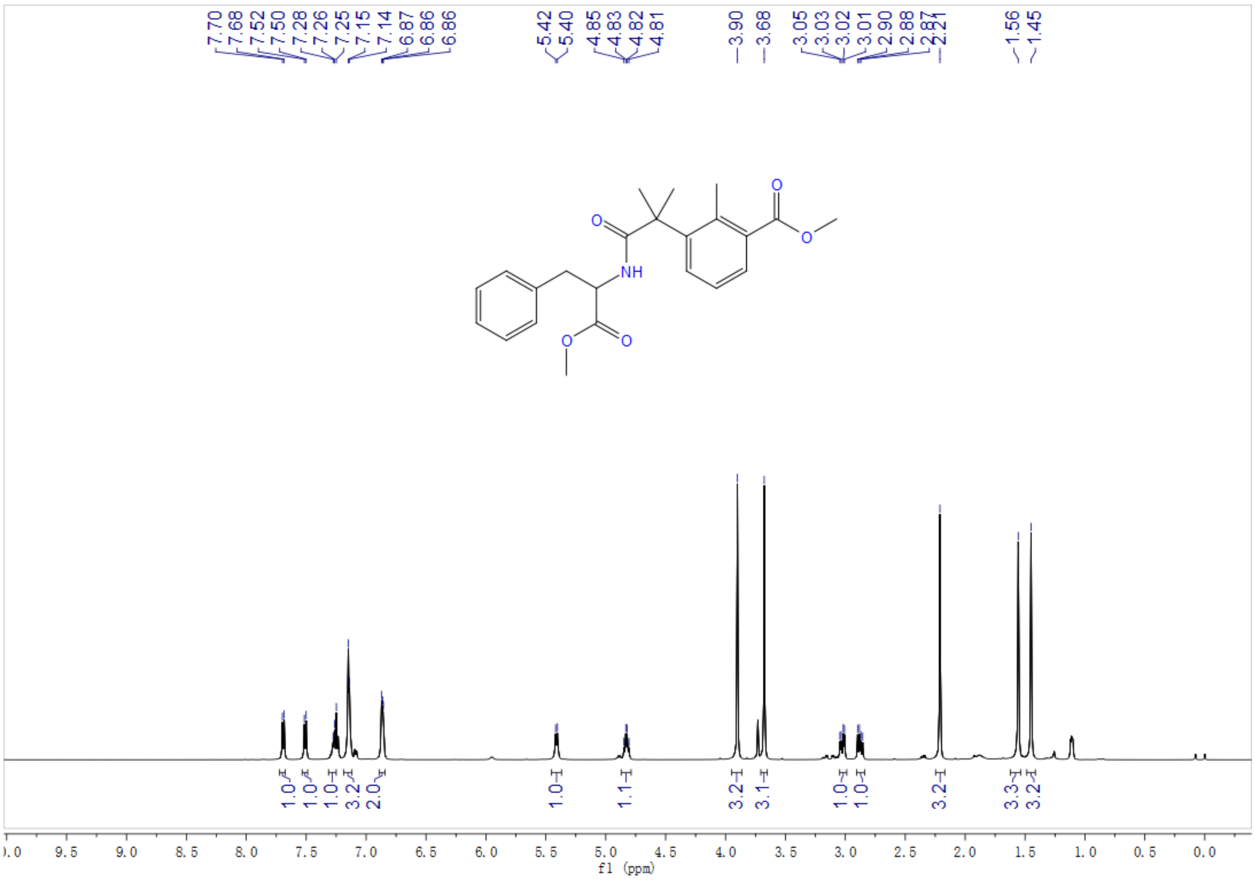

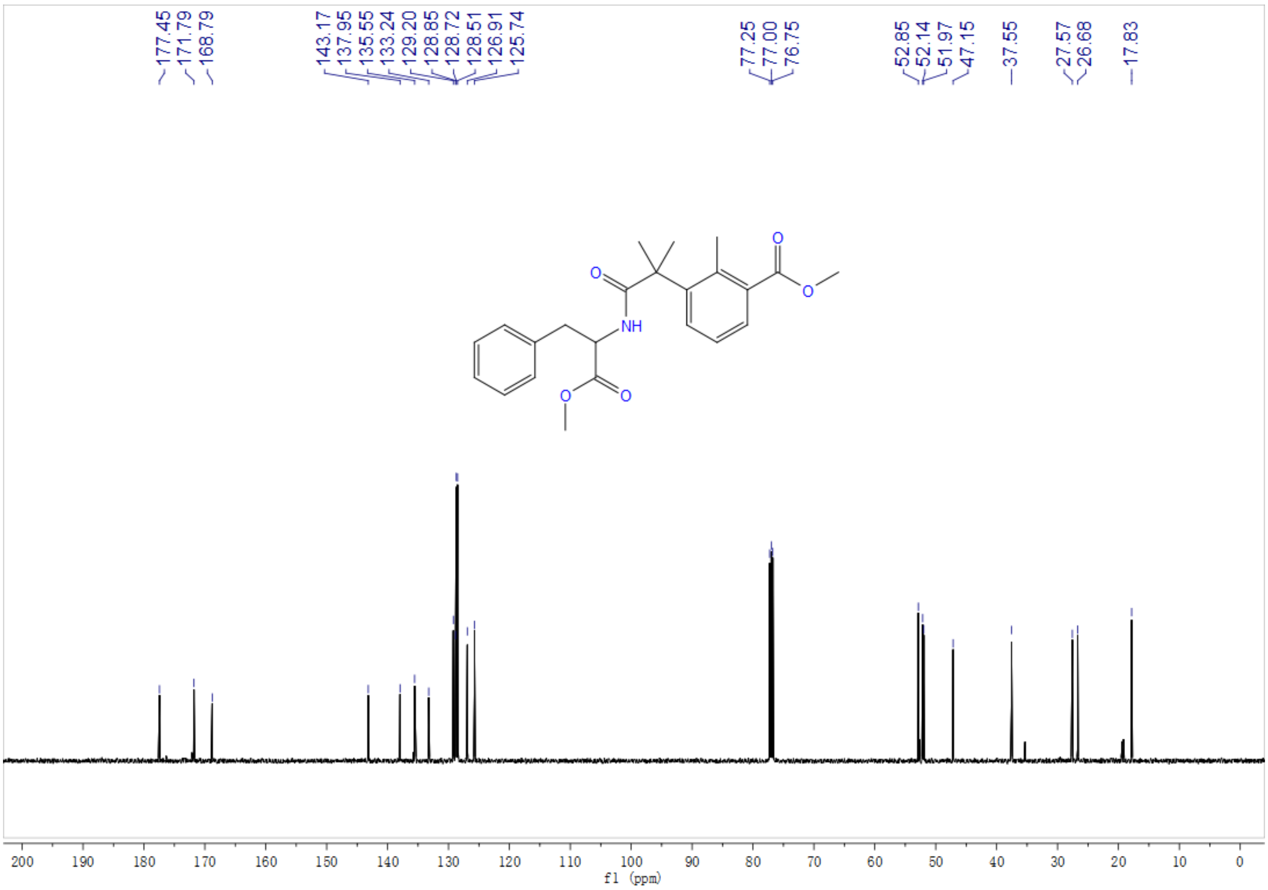

Methyl-3-(1-((1-methoxy-4-(methylthio)-1-oxobutan-2-yl)amino)-2-methyl-1-oxopropan-2-yl)-2-methylbenzoate, **3ap**, ^{1}H NMR (400 MHz, $CDCl_3$) and ^{13}C NMR (125 MHz, $CDCl_3$)

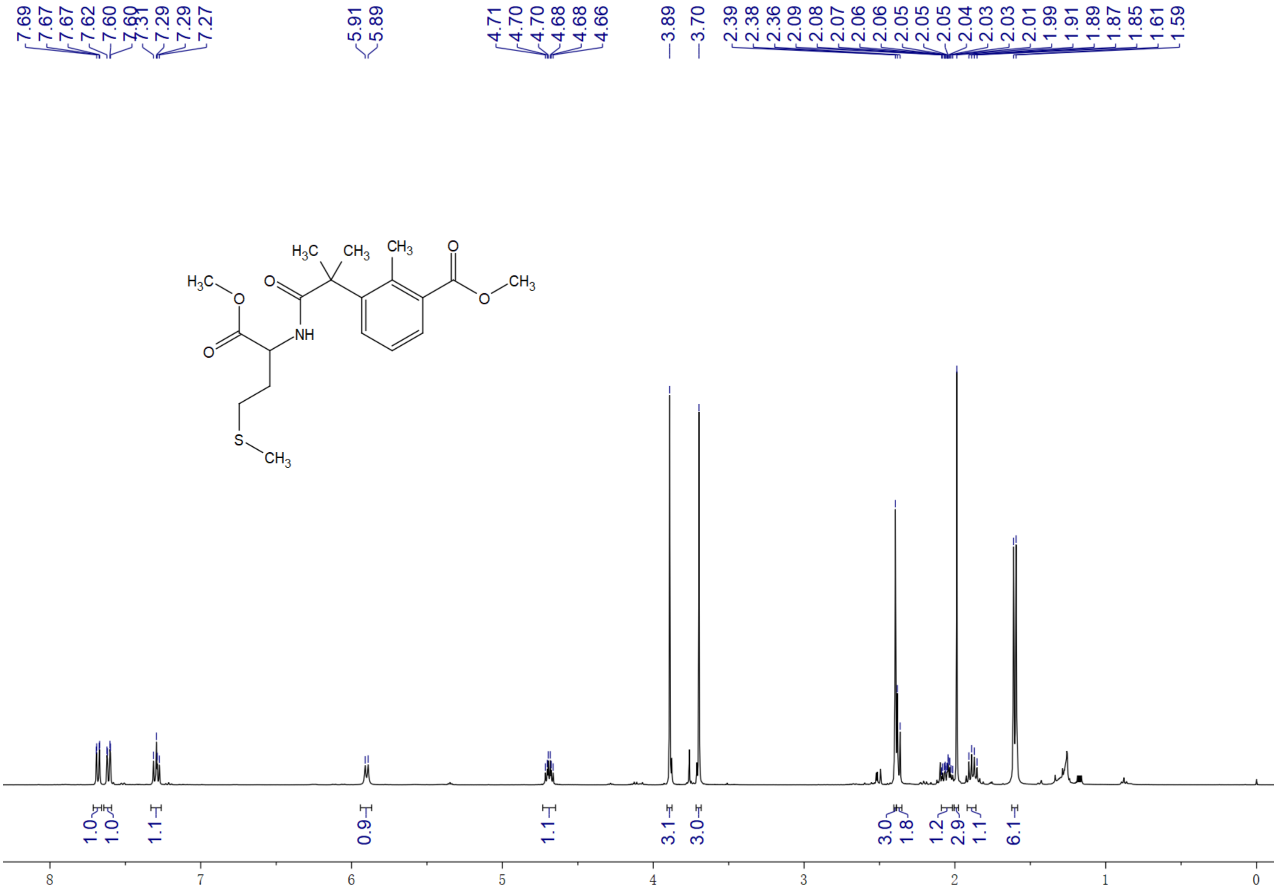

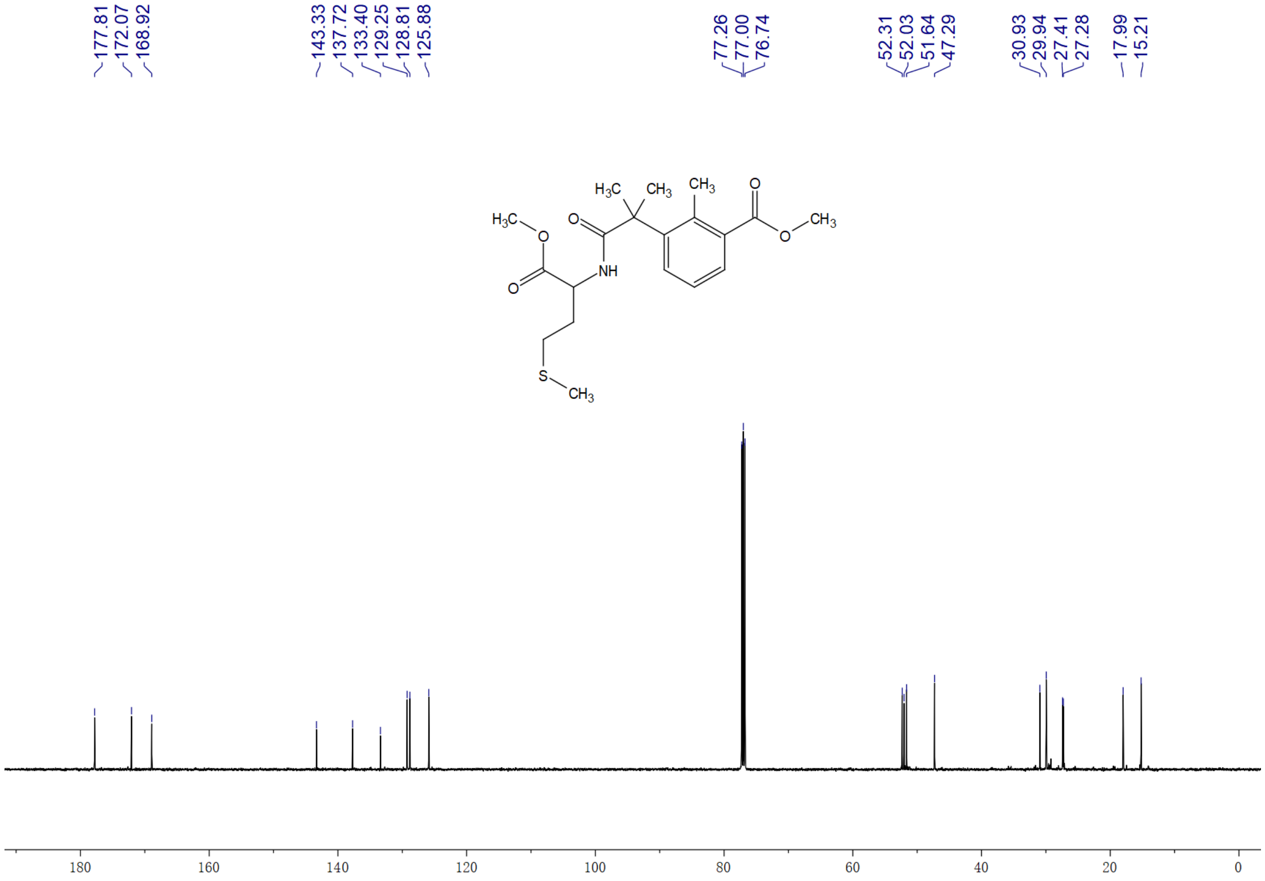

3-(1-Methoxy-2-methyl-1-oxopropan-2-yl)-2-methylbenzoic acid, **3aq**, ^{1}H NMR (500 MHz, DMSO-d_6) and ^{13}C NMR (125 MHz, DMSO-d_6)

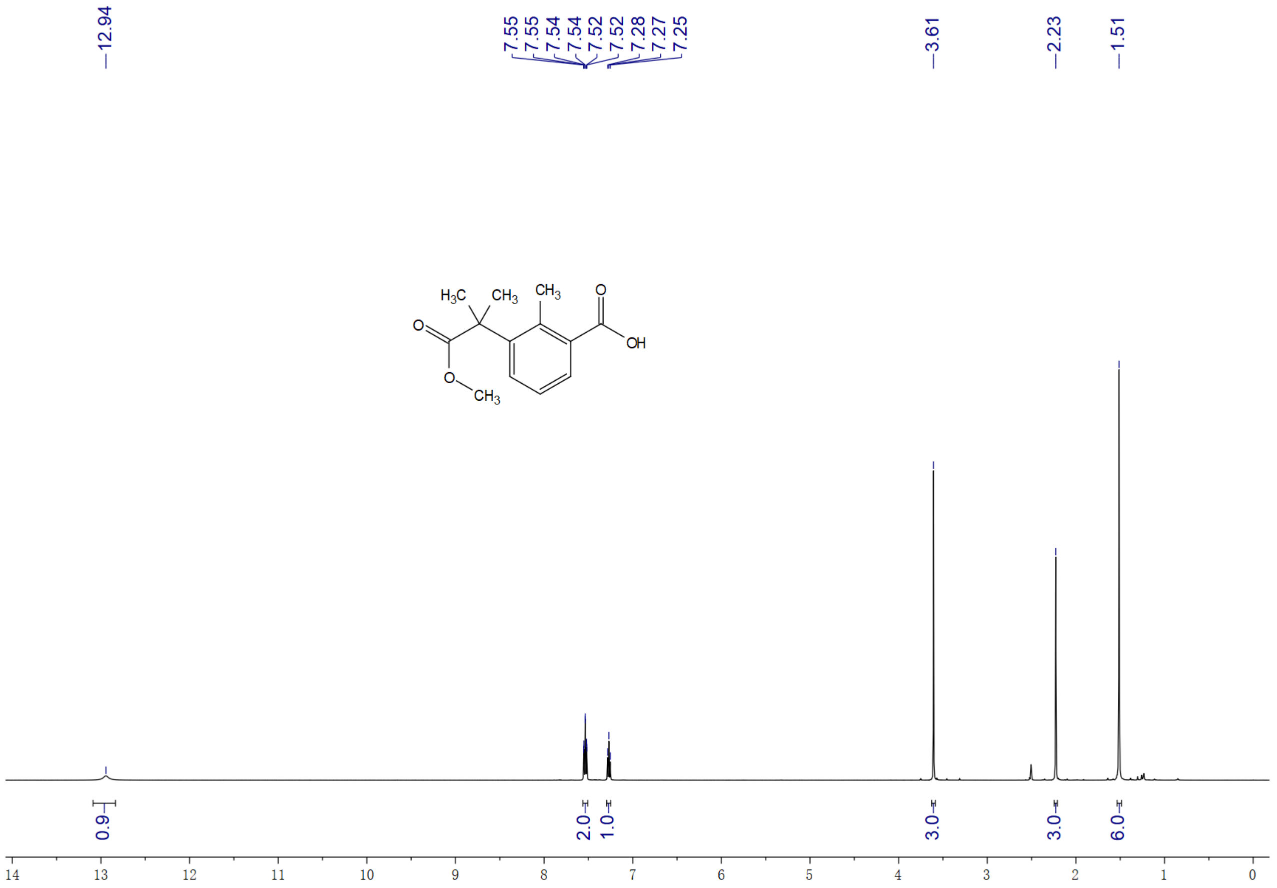

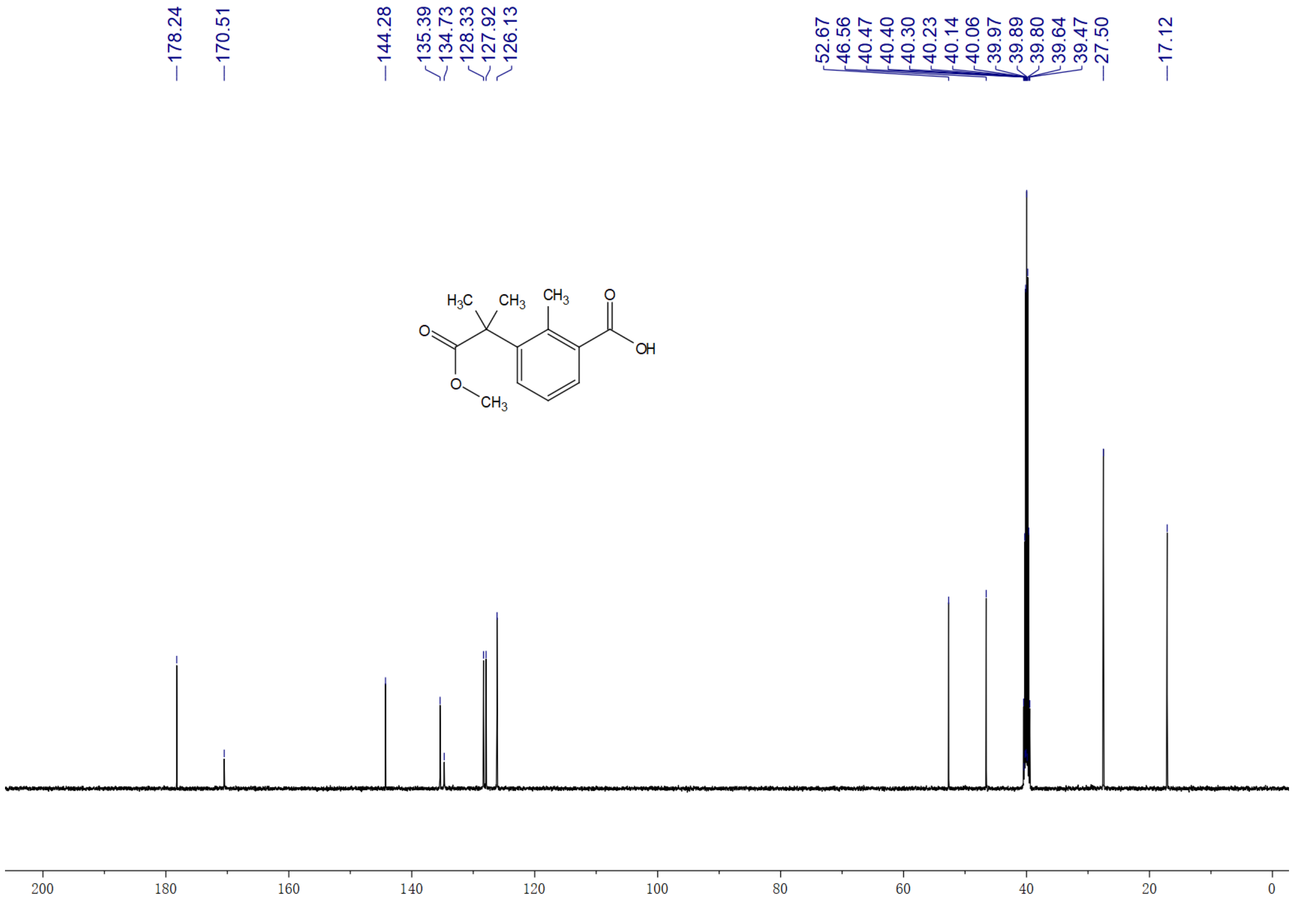

Methyl-3-(1-(tert-butoxy)-2-methyl-1-oxopropan-2-yl)-2-methylbenzoate, **3ar**, ^{1}H NMR (400 MHz, $CDCl_3$) and ^{13}C NMR (100 MHz, $CDCl_3$)

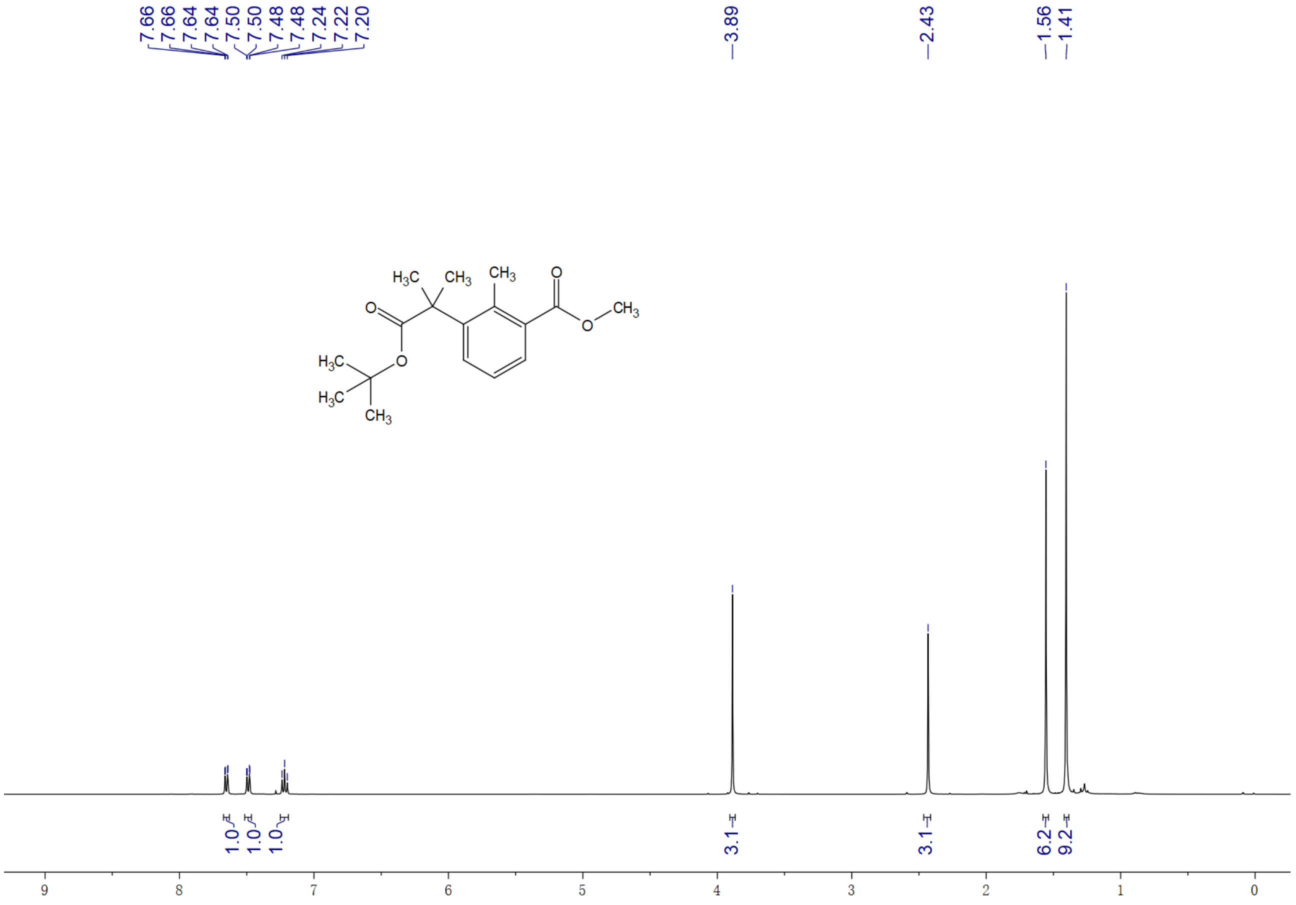

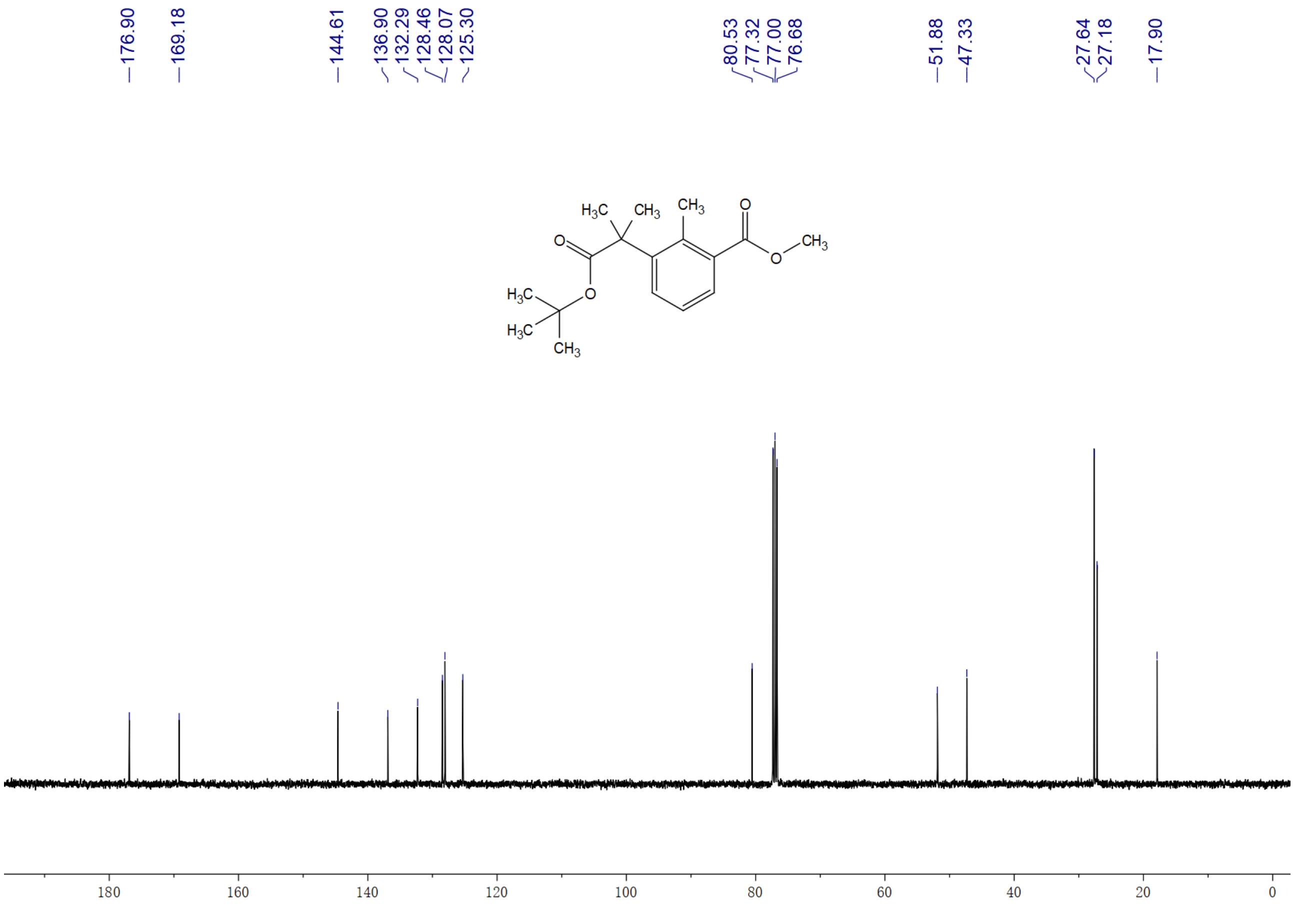

Methyl-2-methyl-3-(2-methyl-1-oxo-1-phenoxypropan-2-yl)benzoate, **3as**, ^{1}H NMR (400 MHz, $CDCl_3$) and ^{13}C NMR (100 MHz, $CDCl_3$)

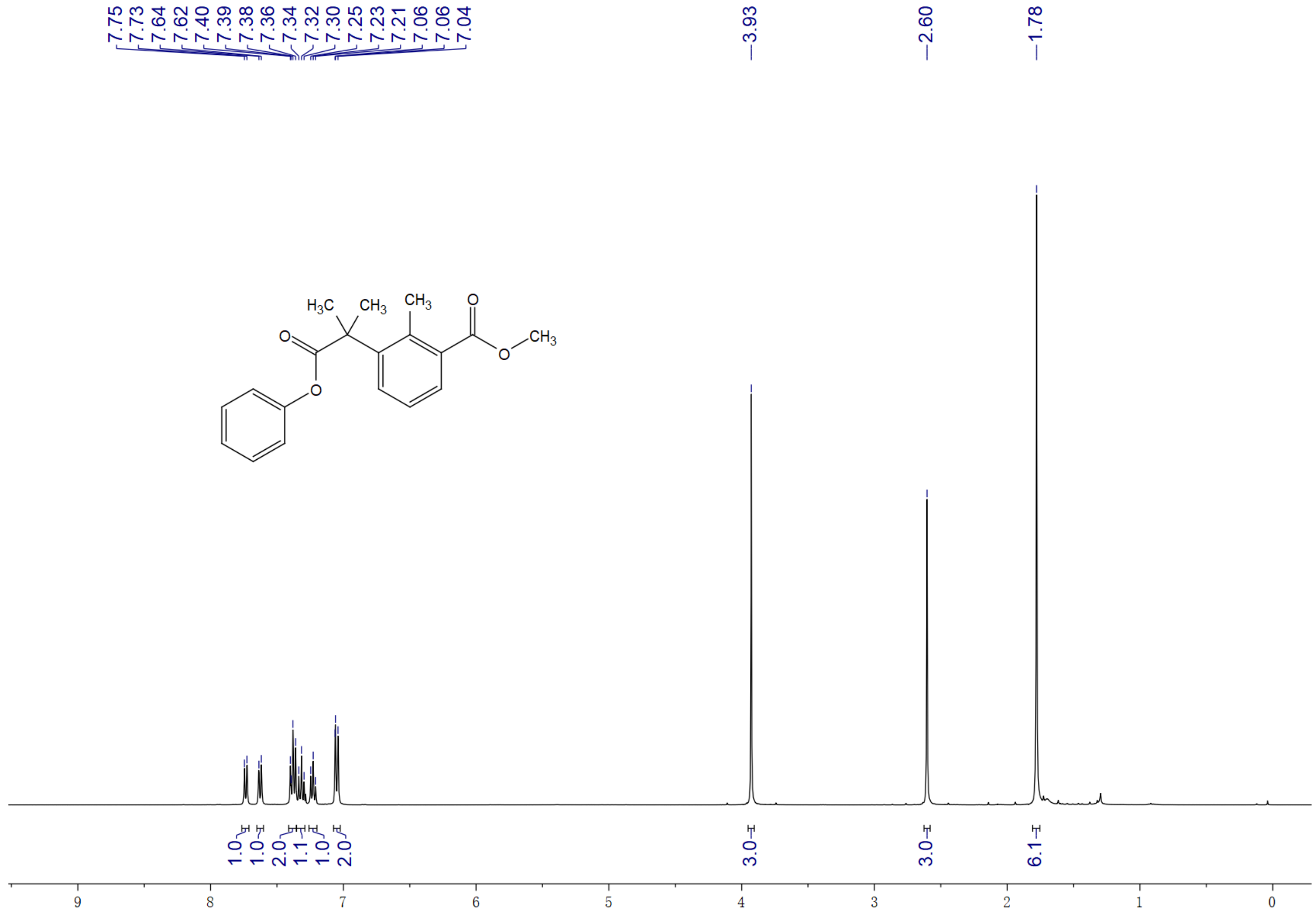

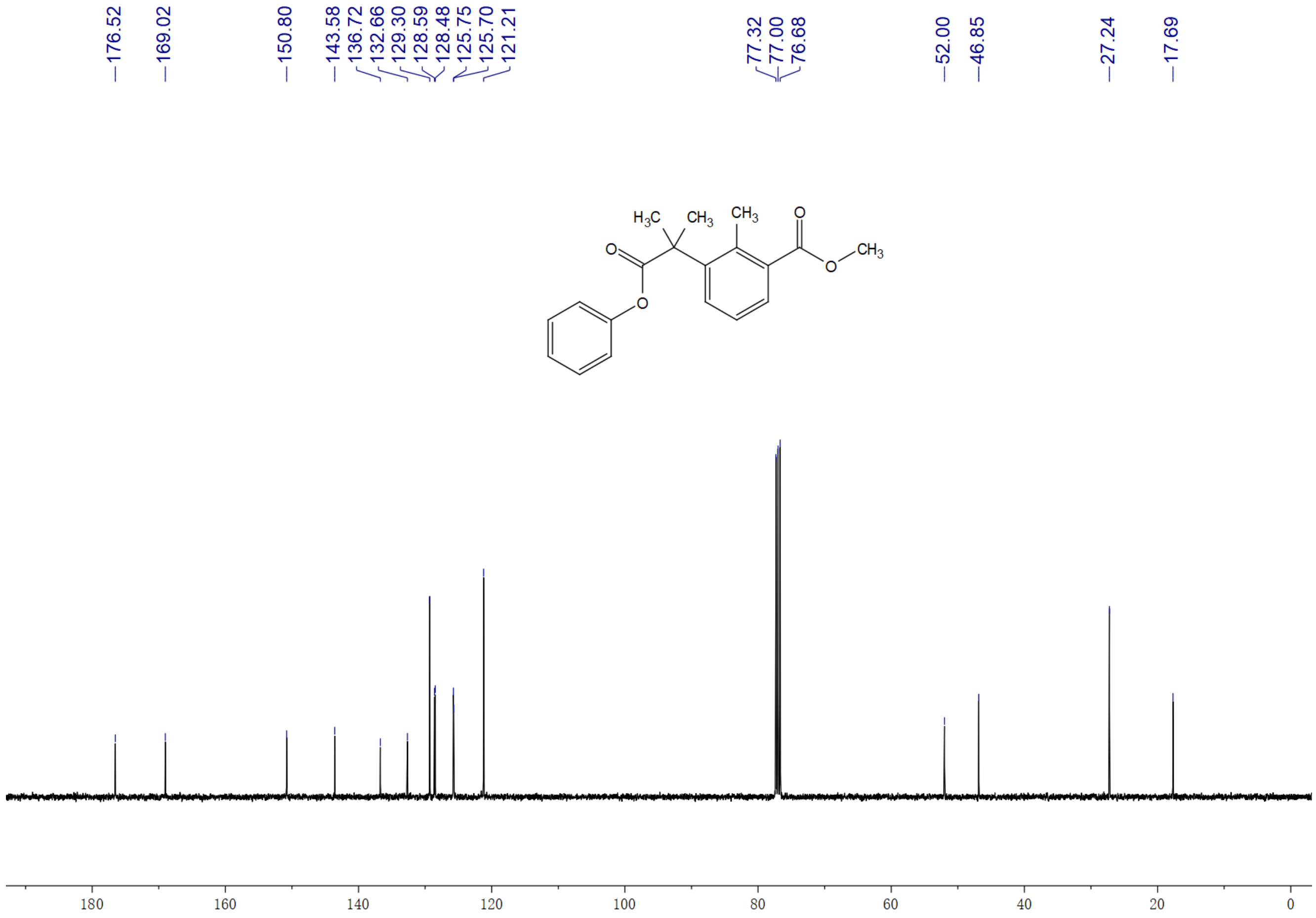

Methyl-3-(1-(benzyloxy)-2-methyl-1-oxopropan-2-yl)-2-methylbenzoate, **3at**, ^{1}H NMR (400 MHz, $CDCl_3$) and ^{13}C NMR (100 MHz, $CDCl_3$)

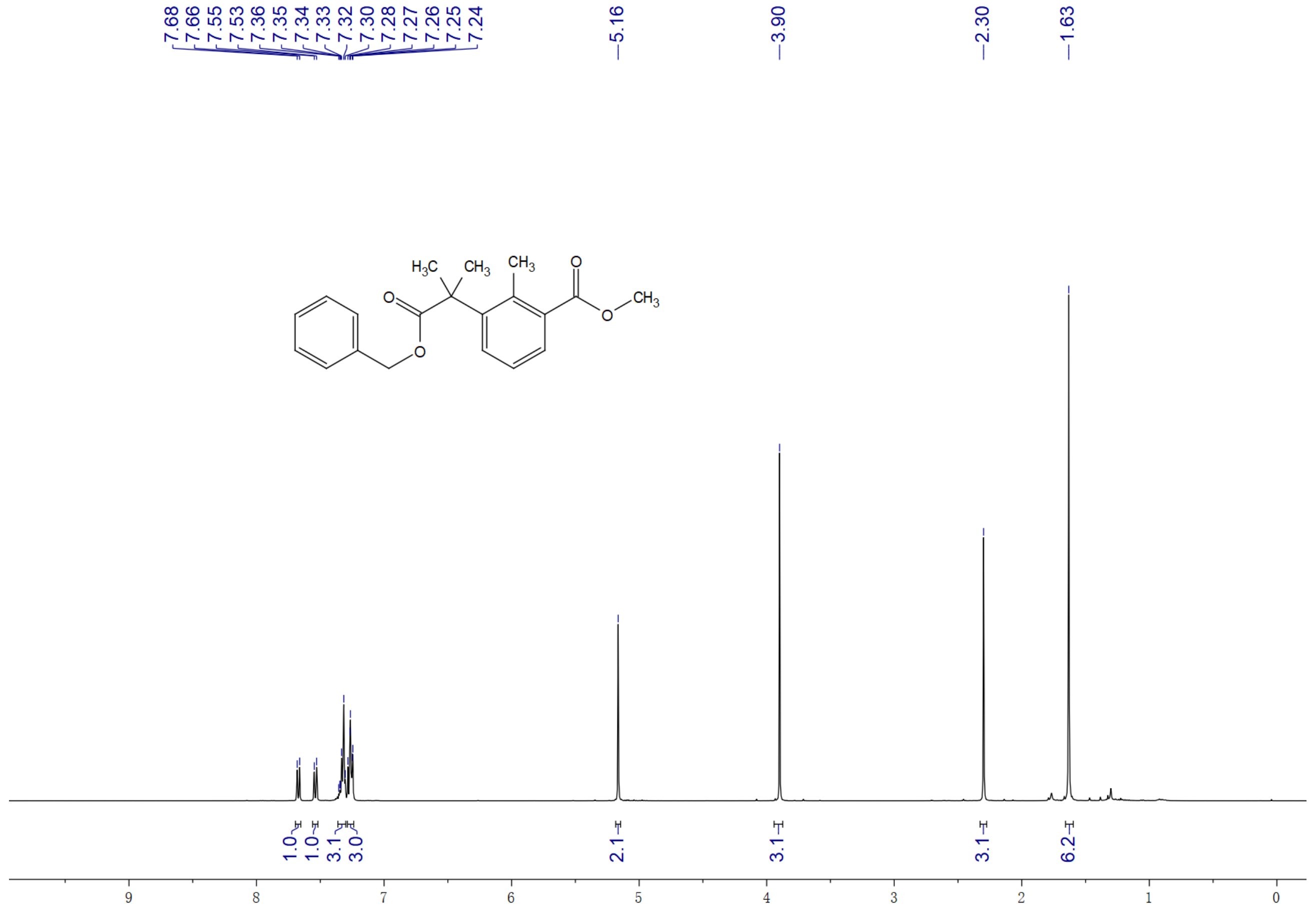

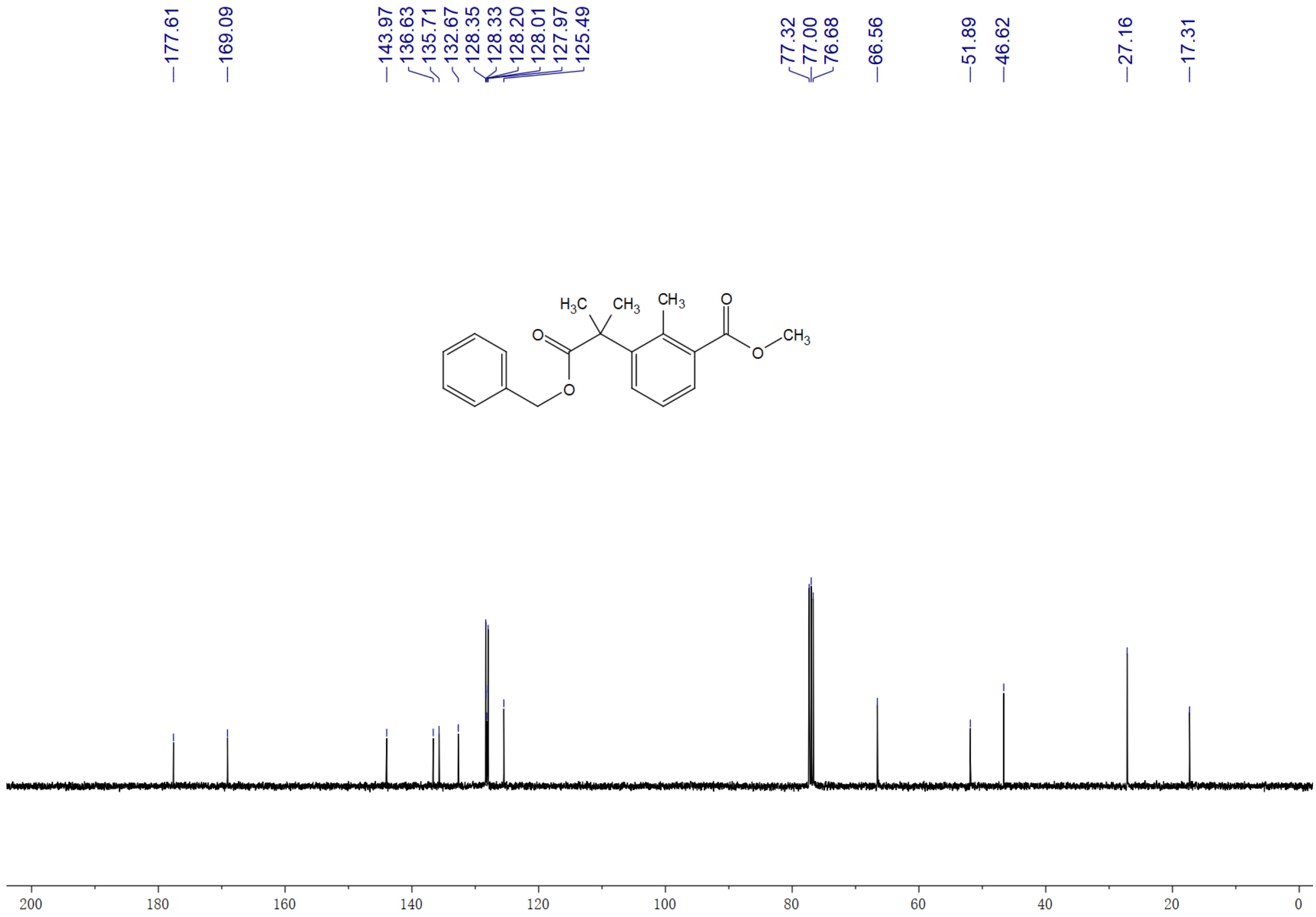

Methyl-2-methyl-3-(2-methyl-1-oxo-1-(2-(thiophen-2-yl)ethoxy)propan-2-yl)benzoate, **3au**, ^{1}H NMR (400 MHz, $CDCl_3$) and ^{13}C NMR (100 MHz, $CDCl_3$)

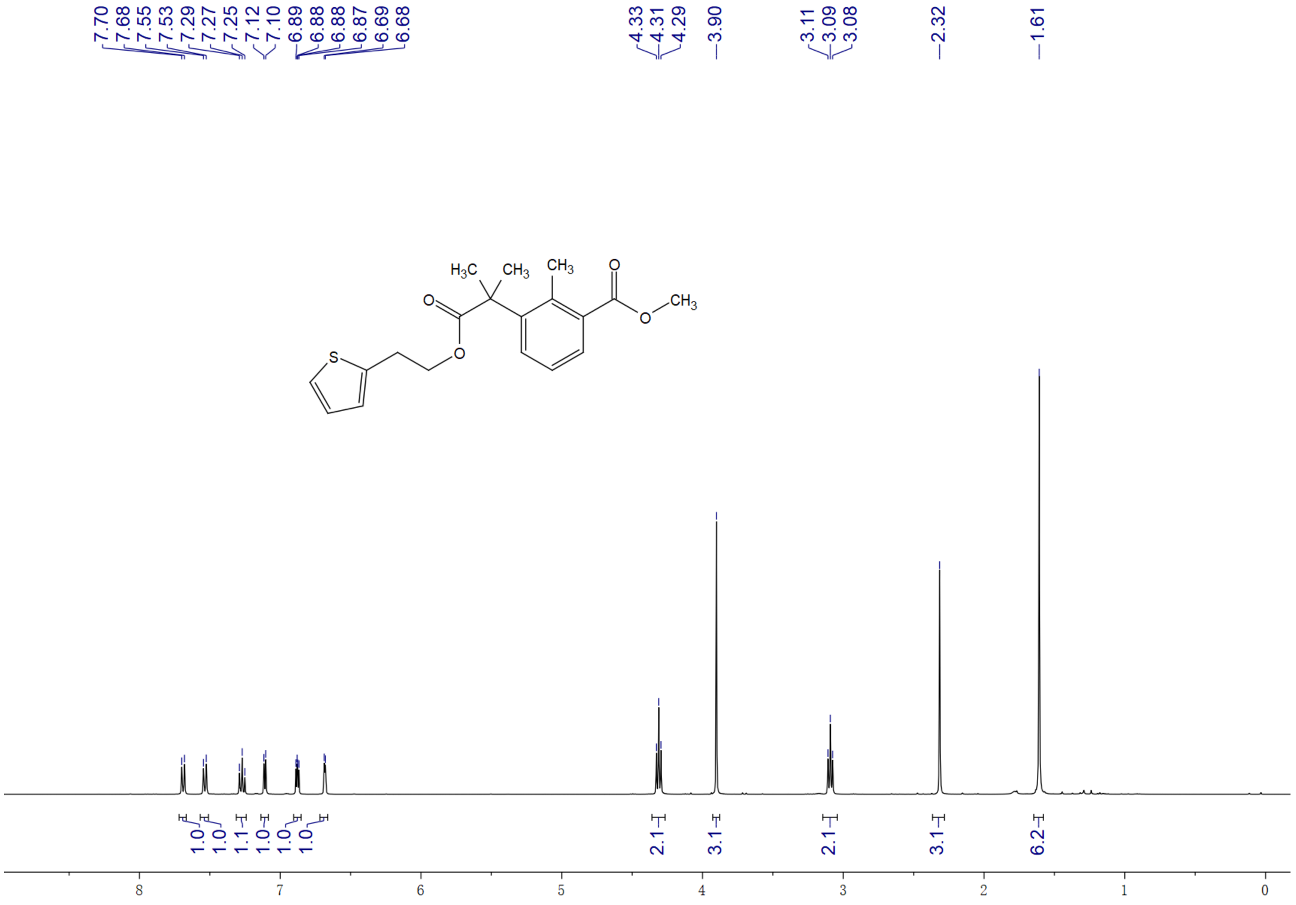

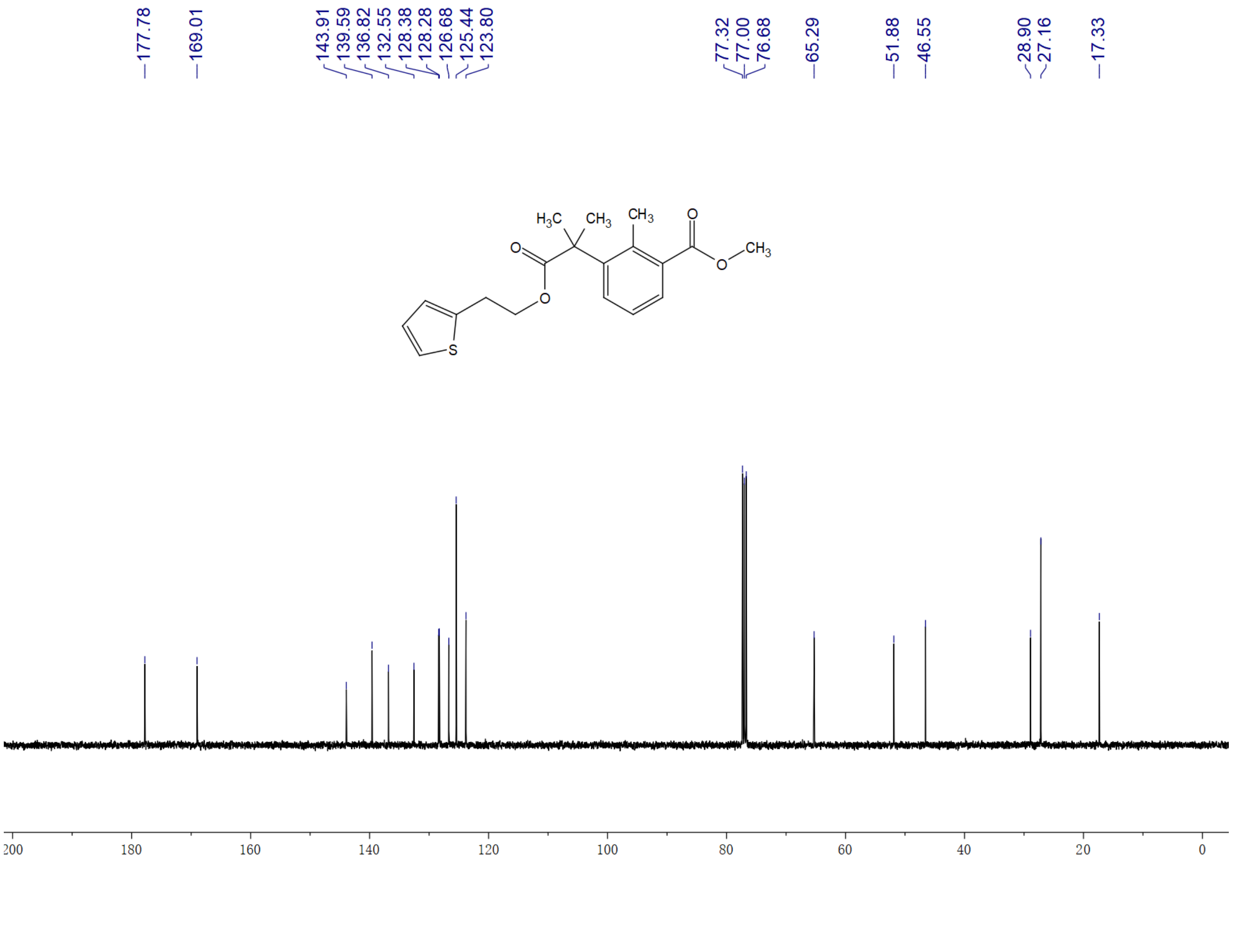

Methyl-3-(1-(ethoxycarbonyl)cyclobutyl)-2-methylbenzoate, **3av**, ^{1}H NMR (400 MHz, $CDCl_3$) and ^{13}C NMR (100 MHz, $CDCl_3$)

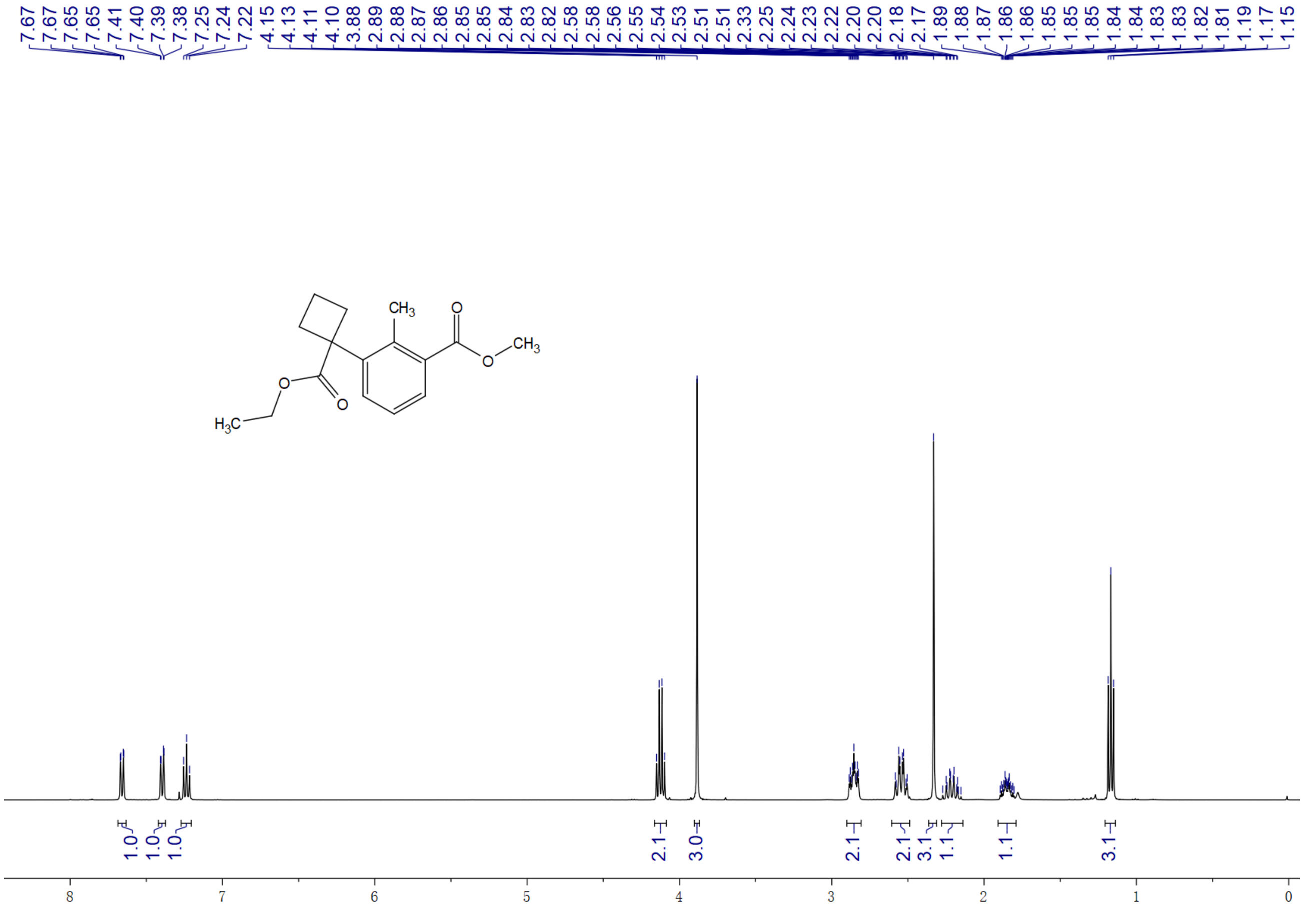

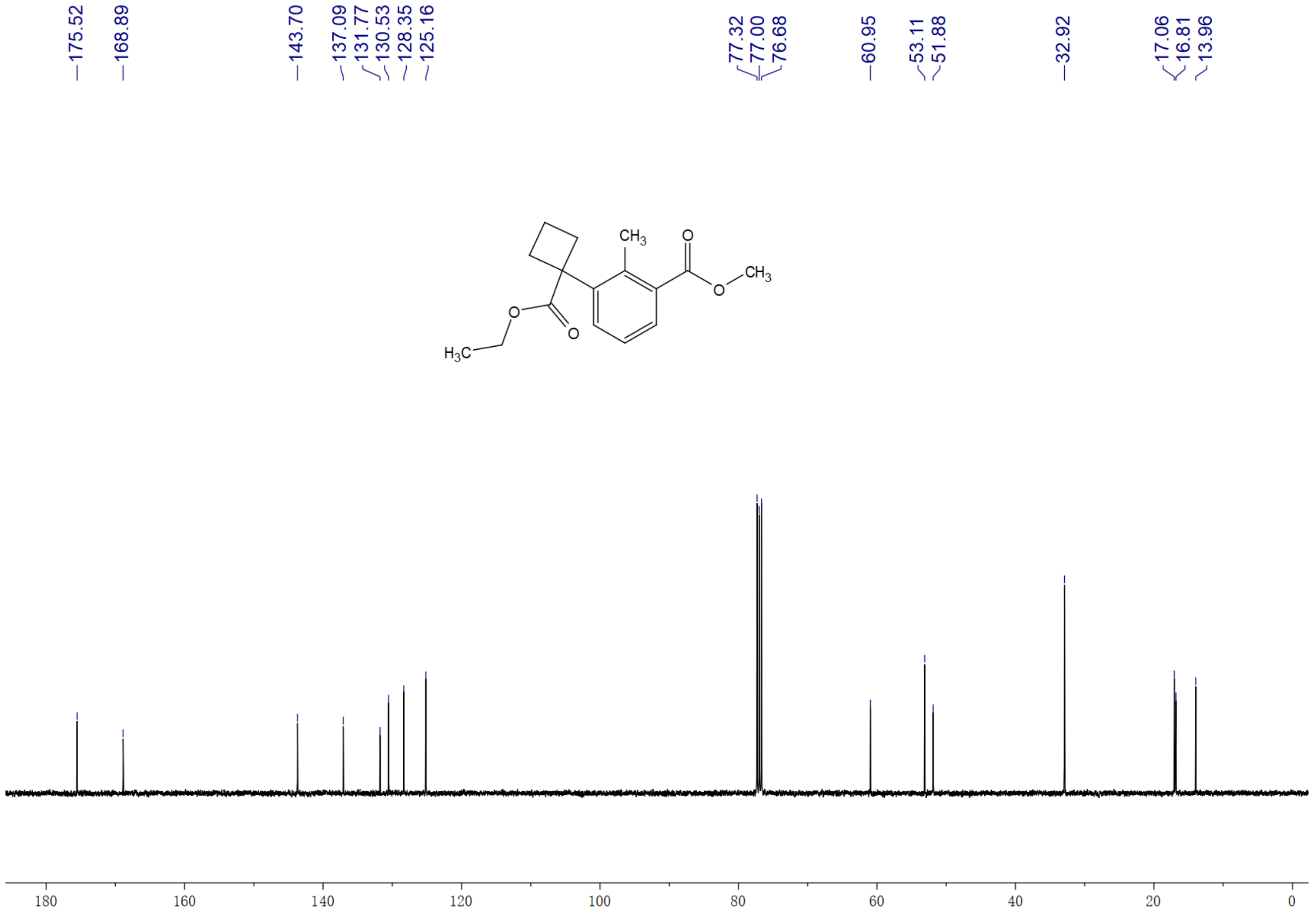

Methyl-2-methyl-3-(2-methyl-3-oxobutan-2-yl)benzoate, **3aw**, ^{1}H NMR (400 MHz, $CDCl_3$) and ^{13}C NMR (100 MHz, $CDCl_3$)

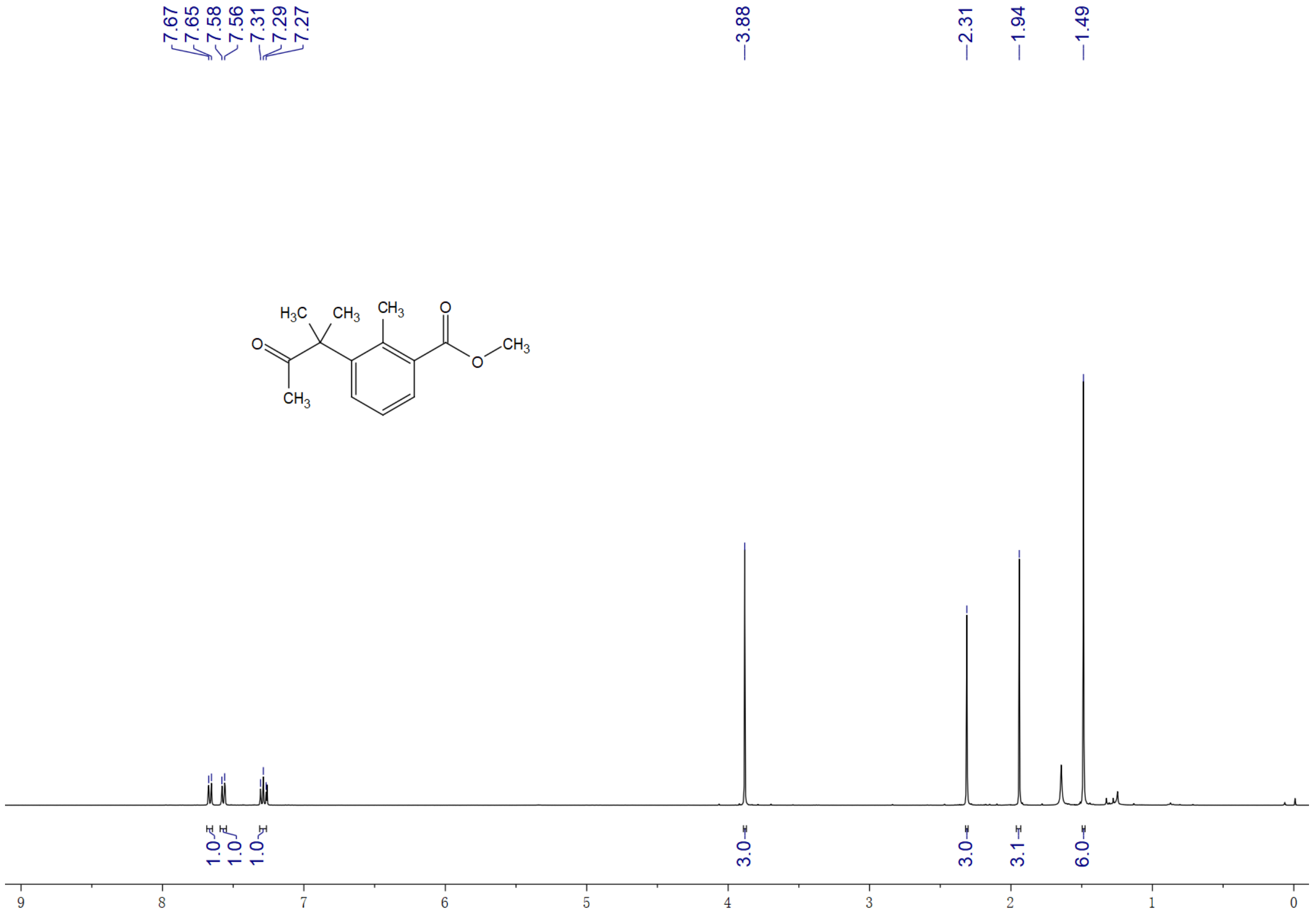

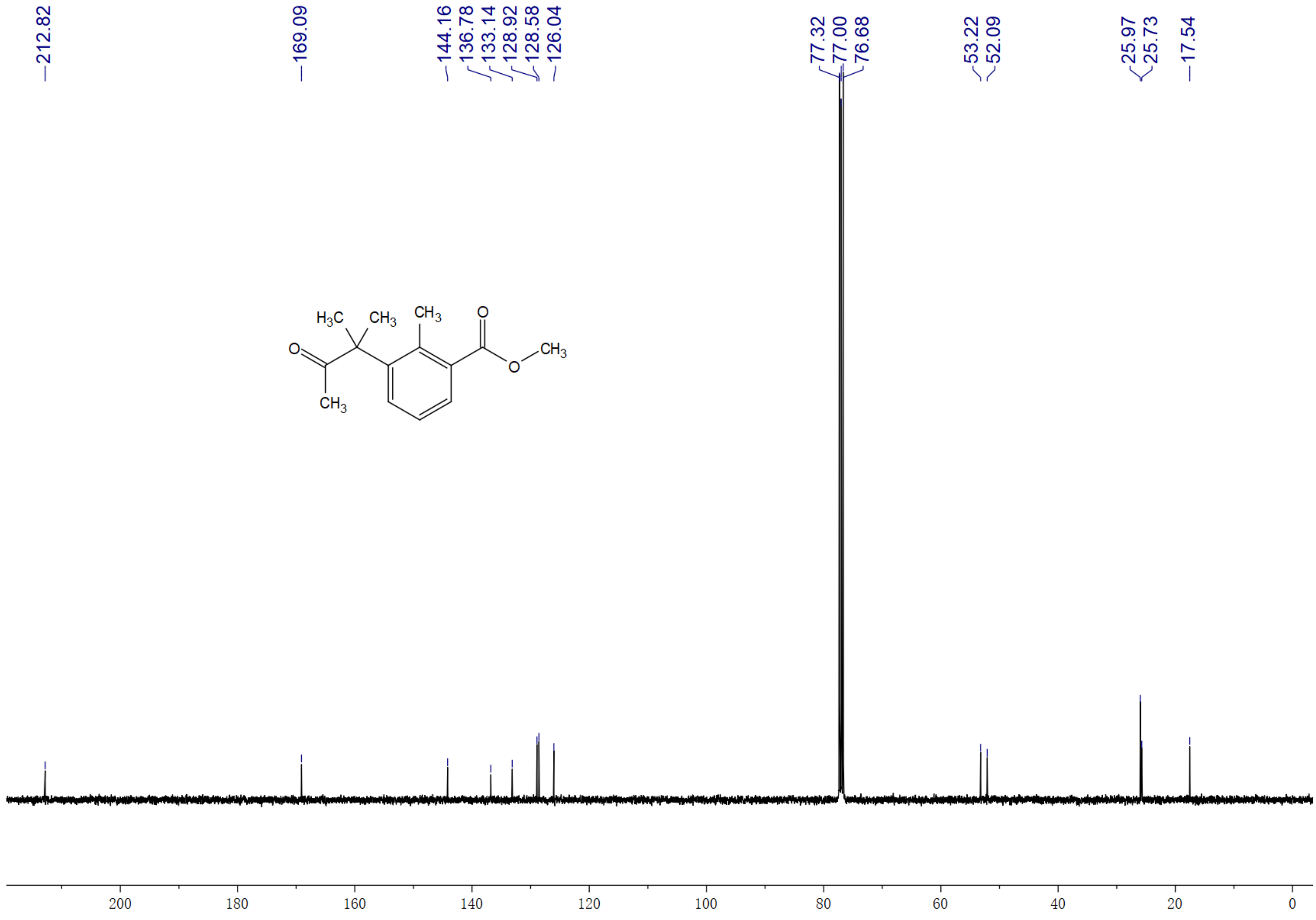

Methyl-3-(1-((2,6-dimethylphenyl)thio)-2-methyl-1-oxopropan-2-yl)-2-methylbenzoate, **3ax**, ^{1}H NMR (400 MHz, $CDCl_3$) and ^{13}C NMR (100 MHz, $CDCl_3$)

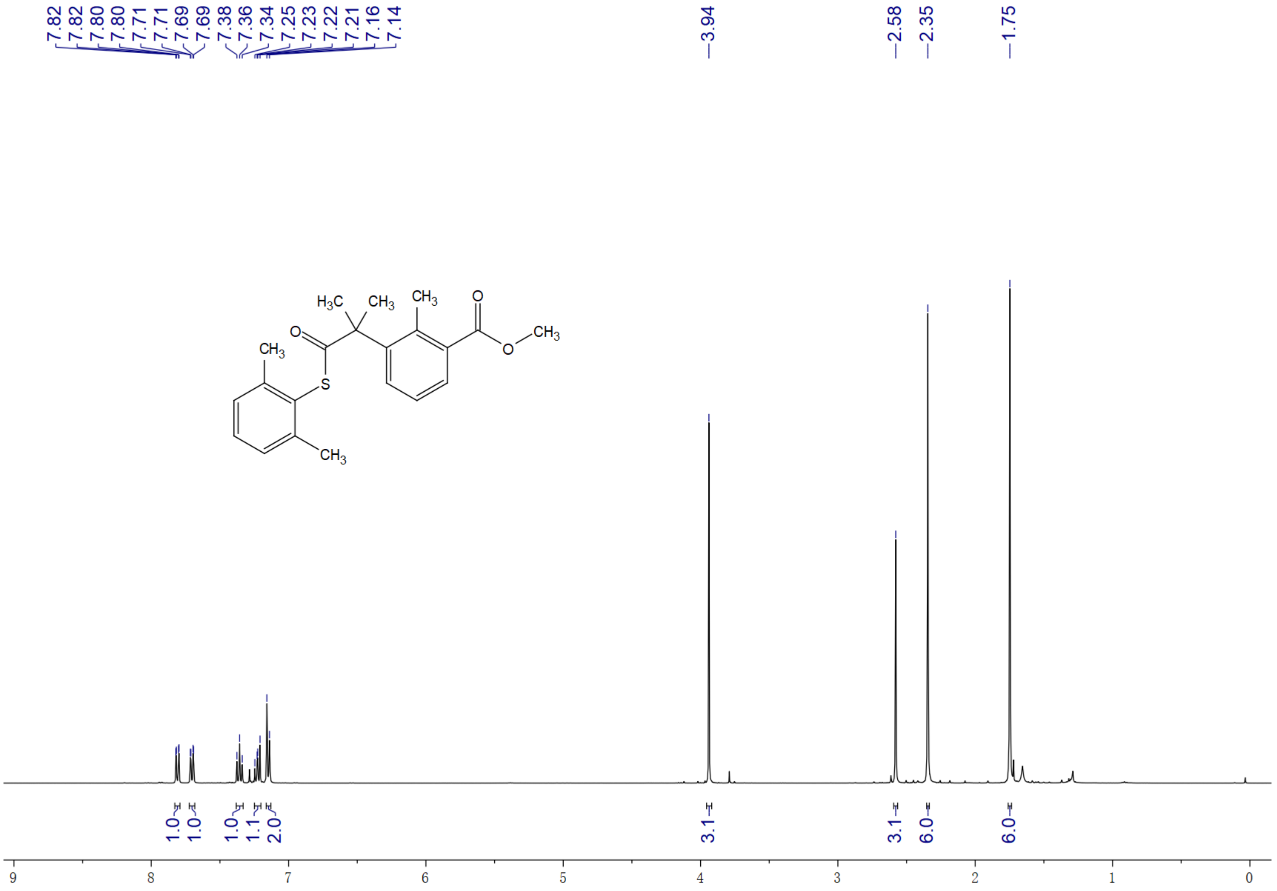

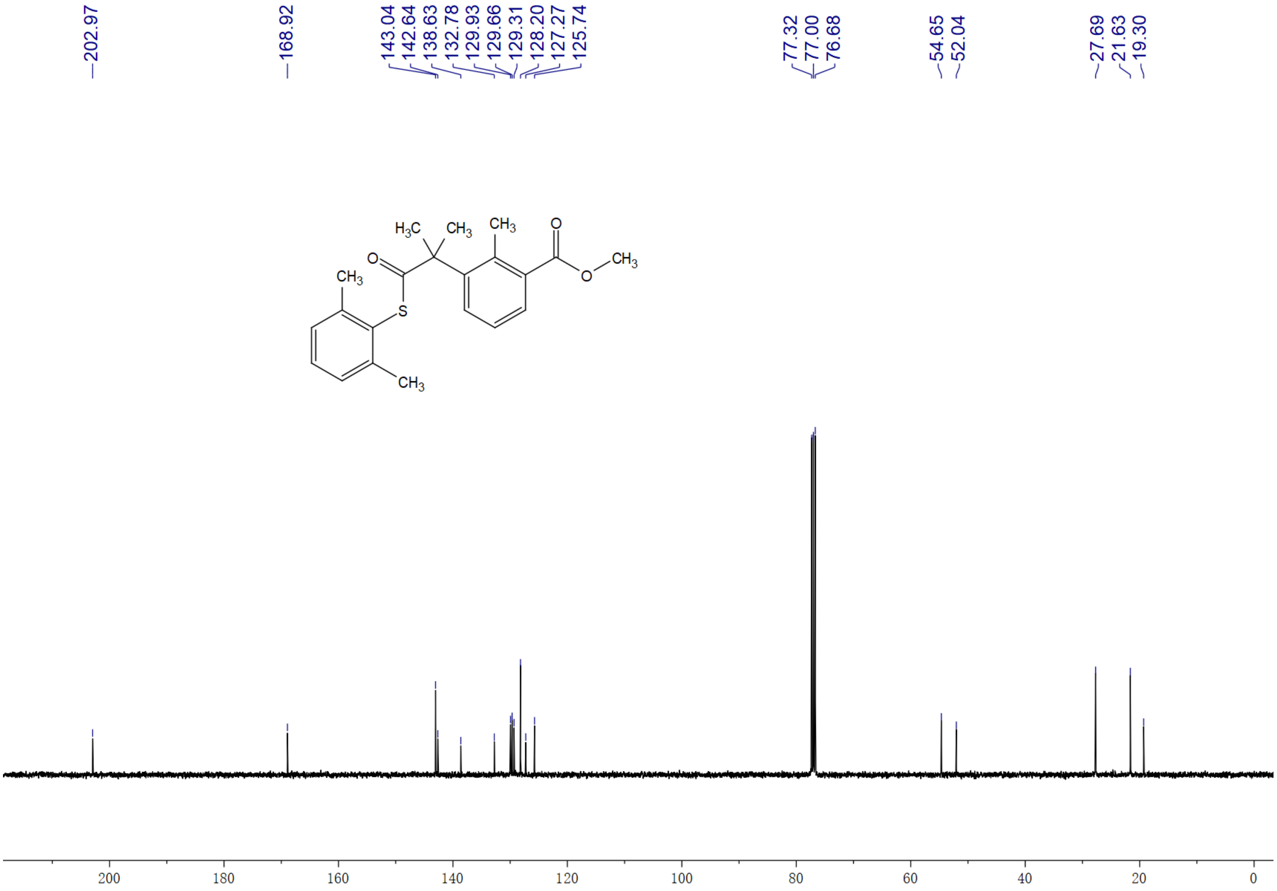

1-Methoxy-1-oxopropan-2-yl 3-(1-methoxy-1-oxopropan-2-yl)-2-methylbenzoate, **3ay**, ^{1}H NMR (500 MHz, $CDCl_3$) and ^{13}C NMR (125 MHz, $CDCl_3$)

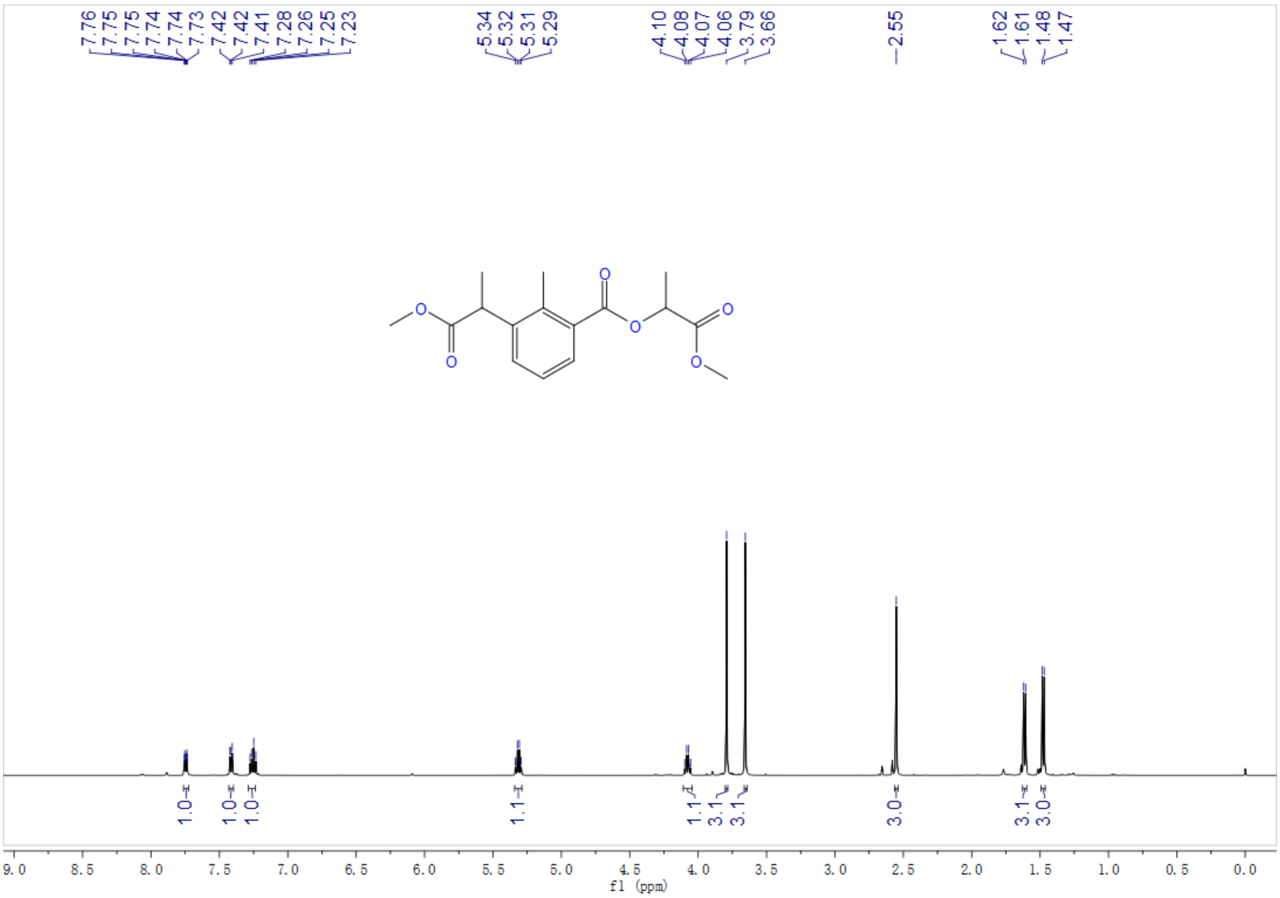

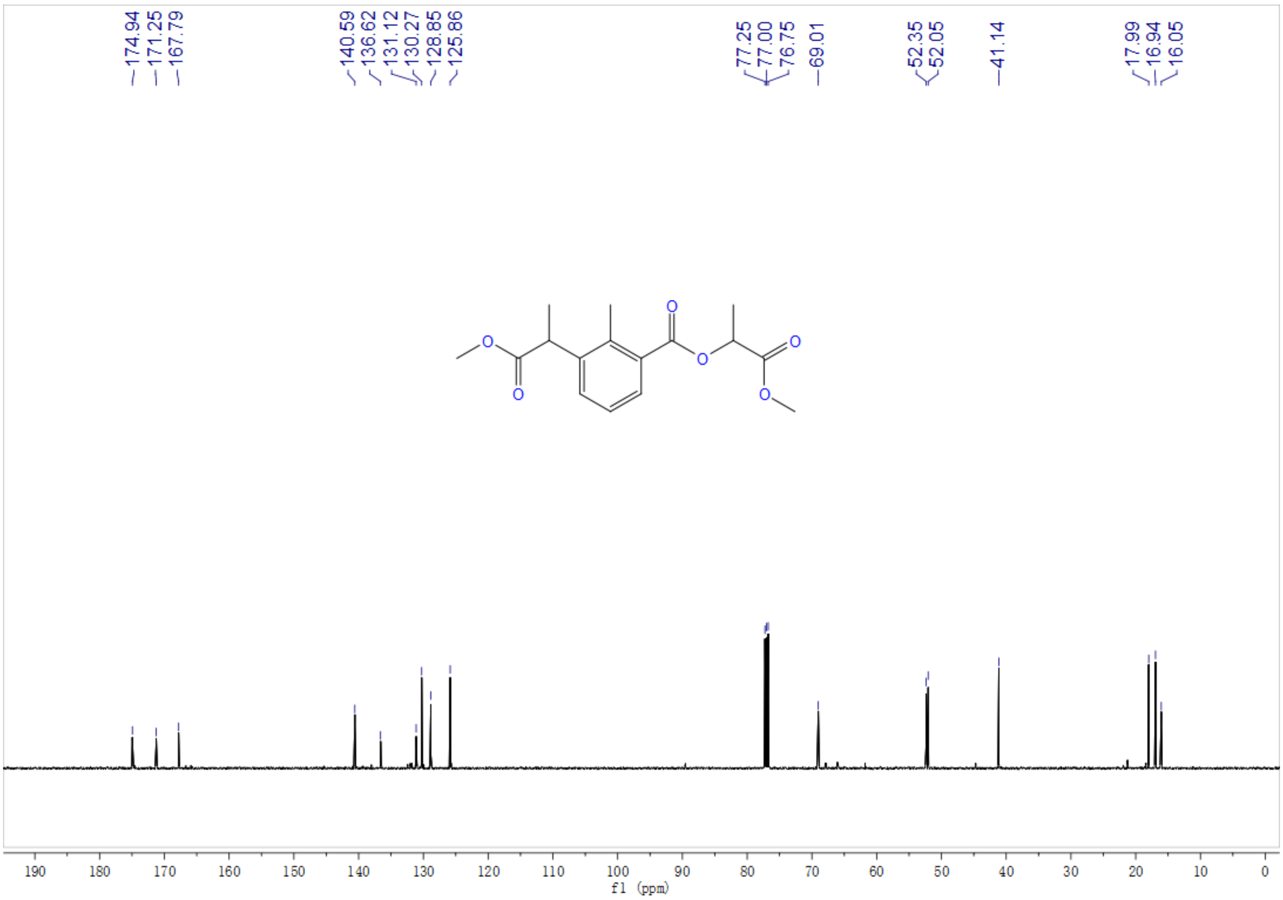

Methoxy-1-oxopropan-2-yl 2-methylbenzoate, **3ay'**, ^{1}H NMR (400 MHz, $CDCl_3$) and ^{13}C NMR (100 MHz, $CDCl_3$)

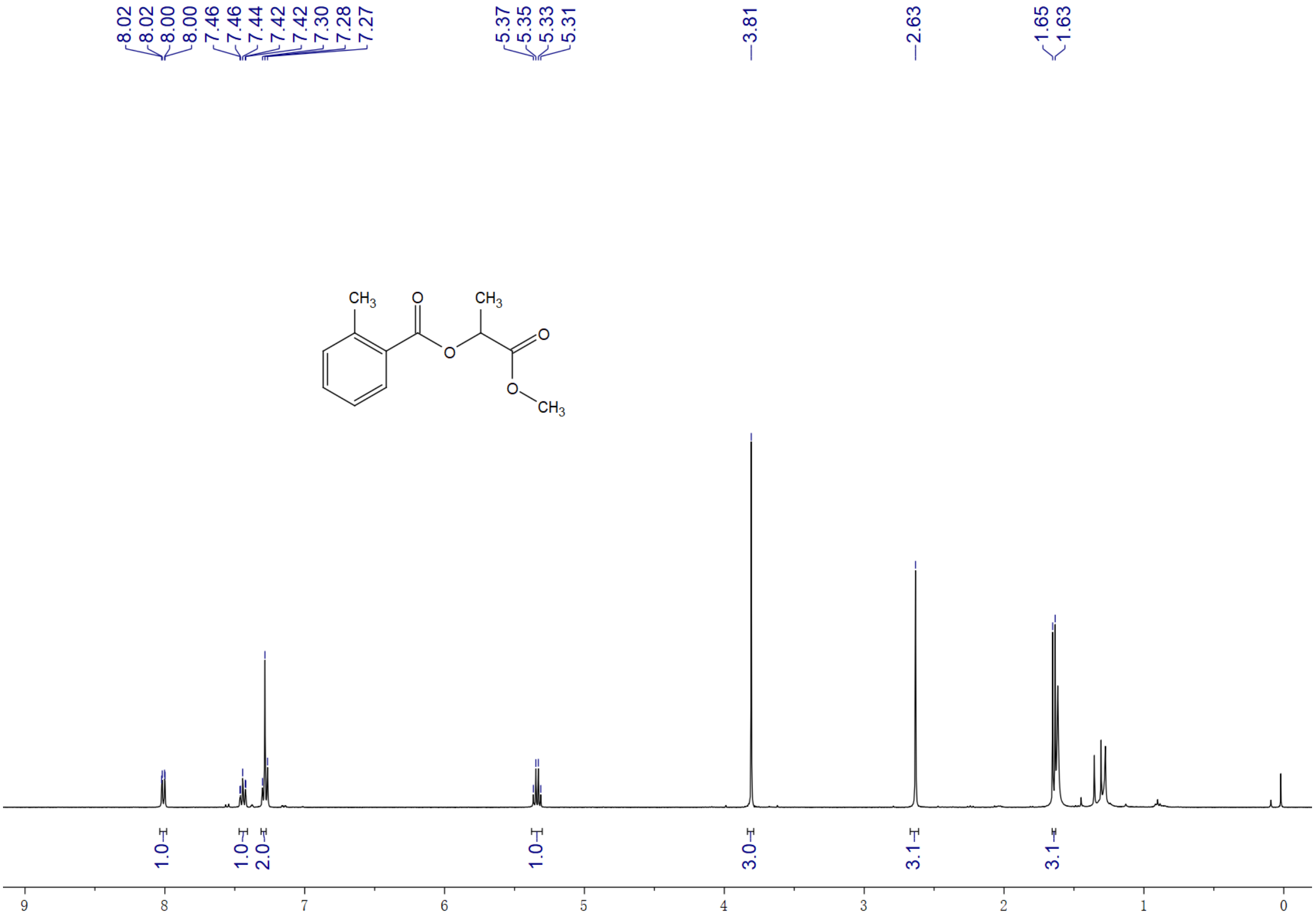

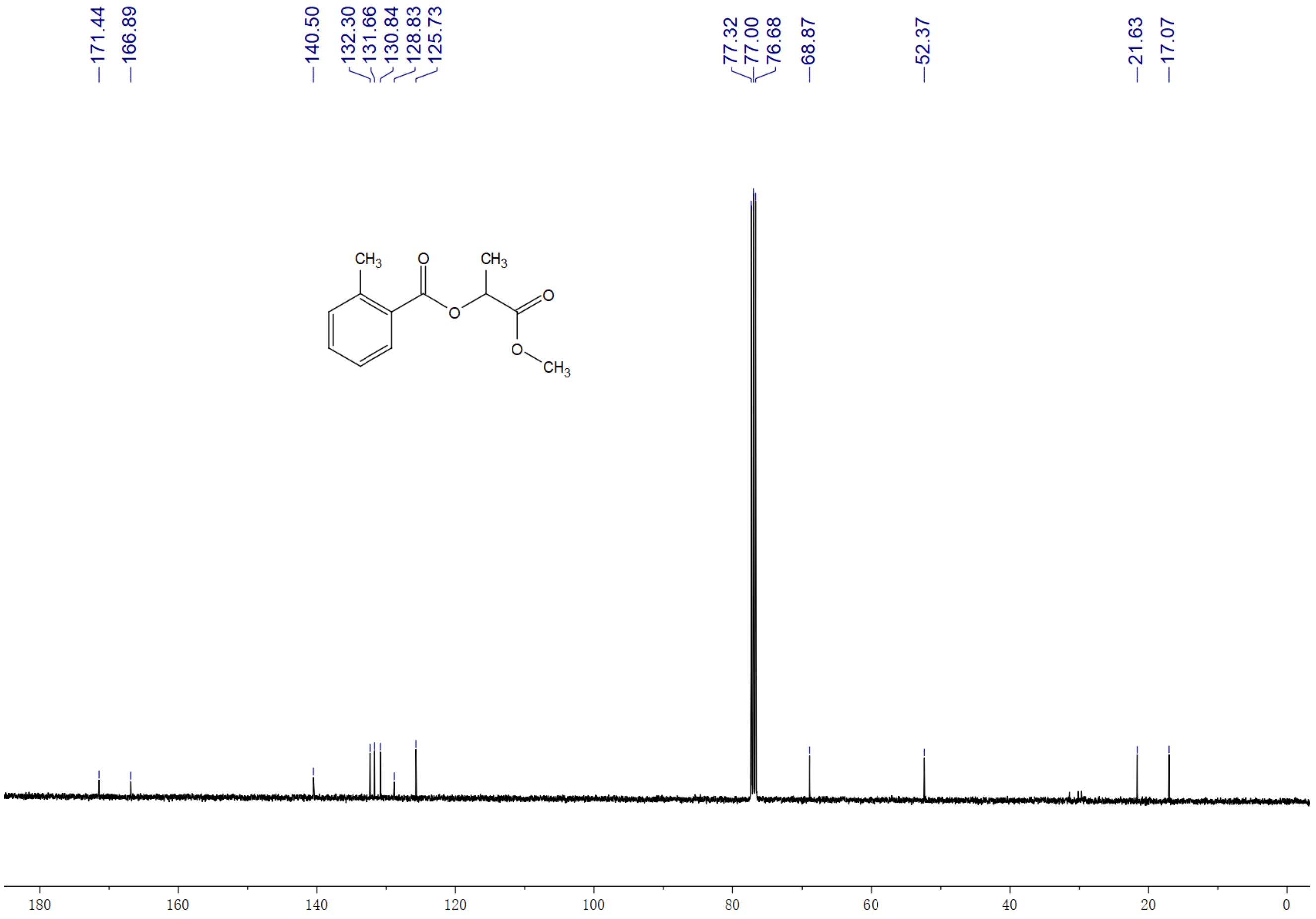

Butyl-2-methylbenzoate, **3az**, ^{1}H NMR (400 MHz, $CDCl_3$) and ^{13}C NMR (100 MHz, $CDCl_3$)

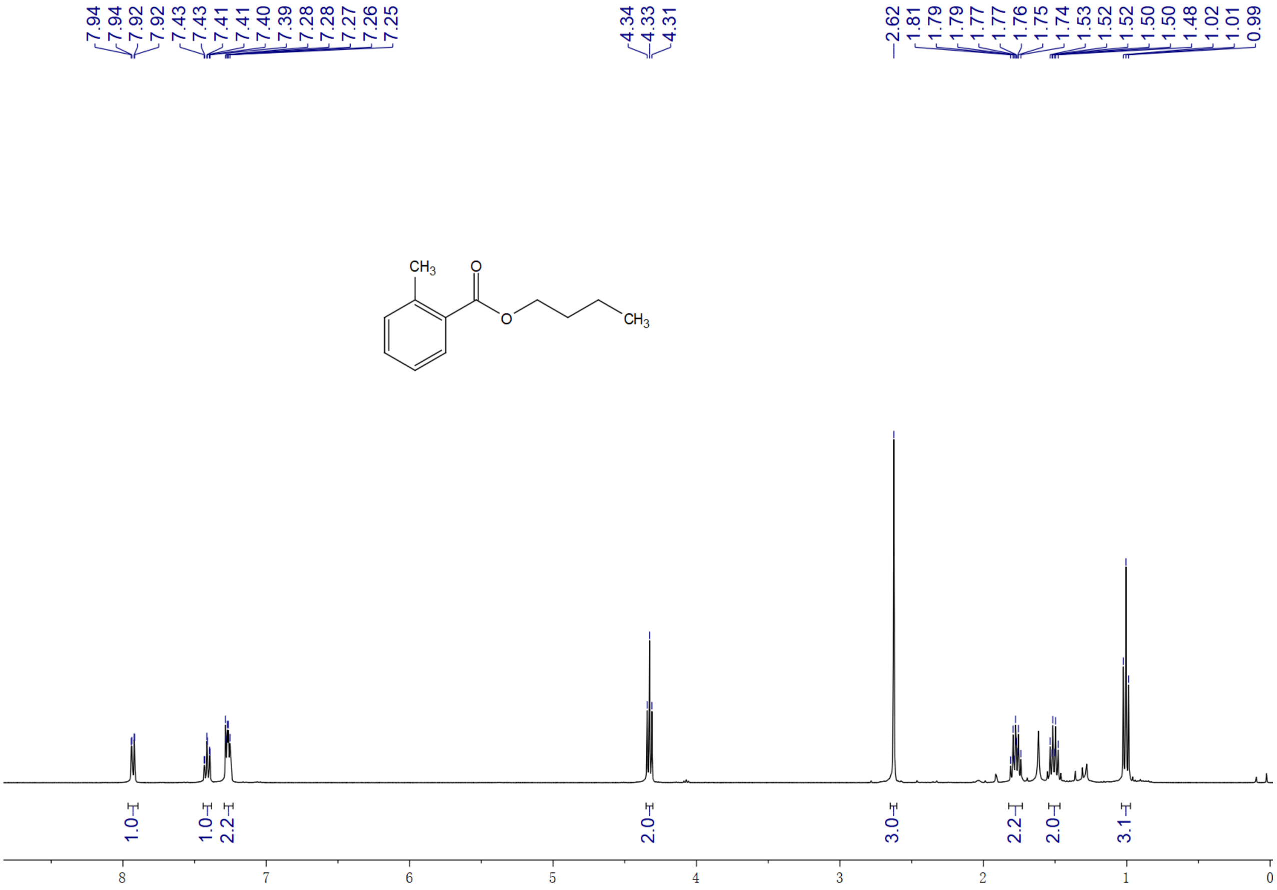

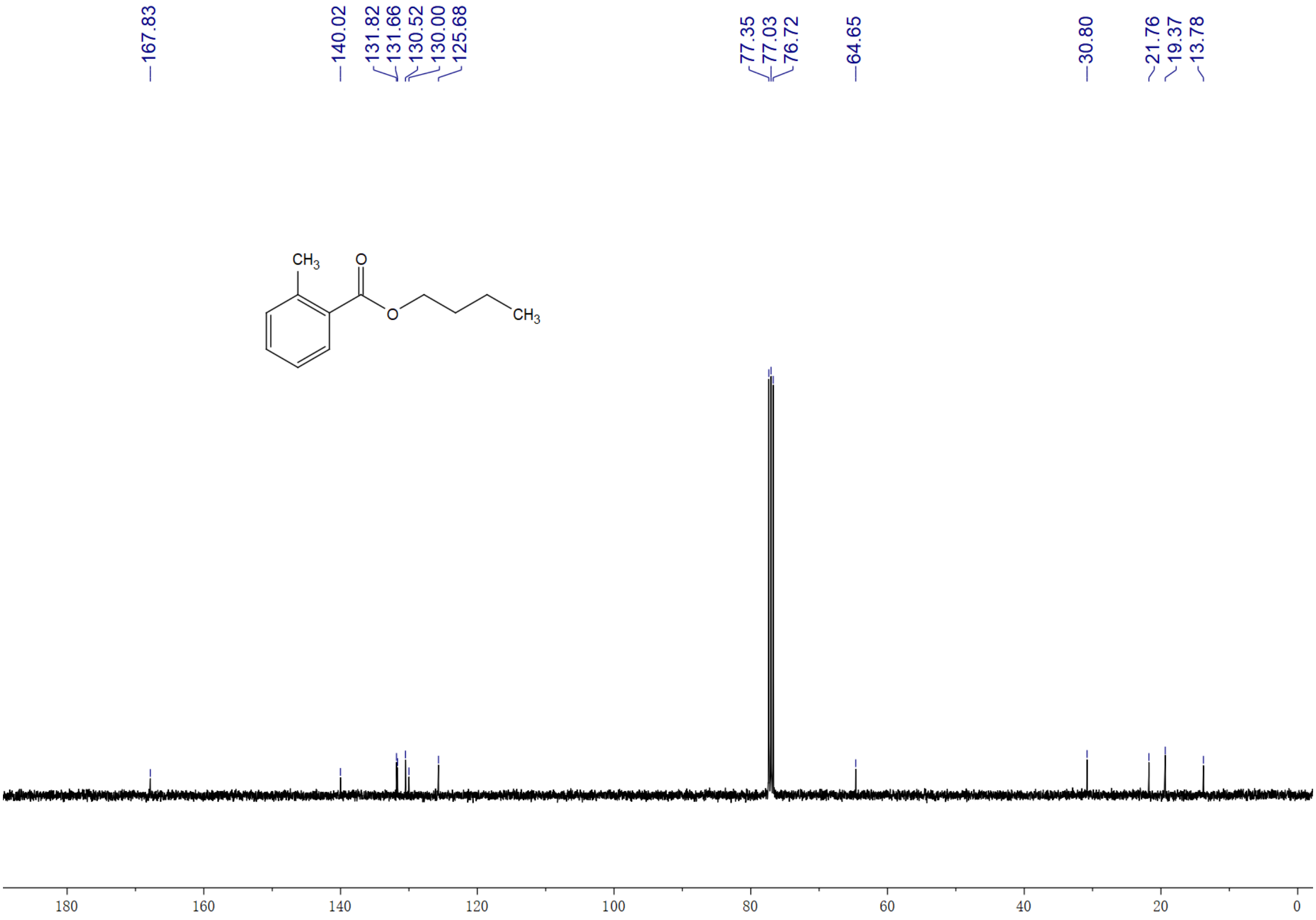

Methyl-3-(2-ethoxy-1,1-difluoro-2-oxoethyl)-2-methylbenzoate, **3bb**, ^{1}H NMR (500 MHz, $CDCl_3$), ^{13}C NMR (125 MHz, $CDCl_3$) and ^{19}F NMR (471 MHz, $CDCl_3$)

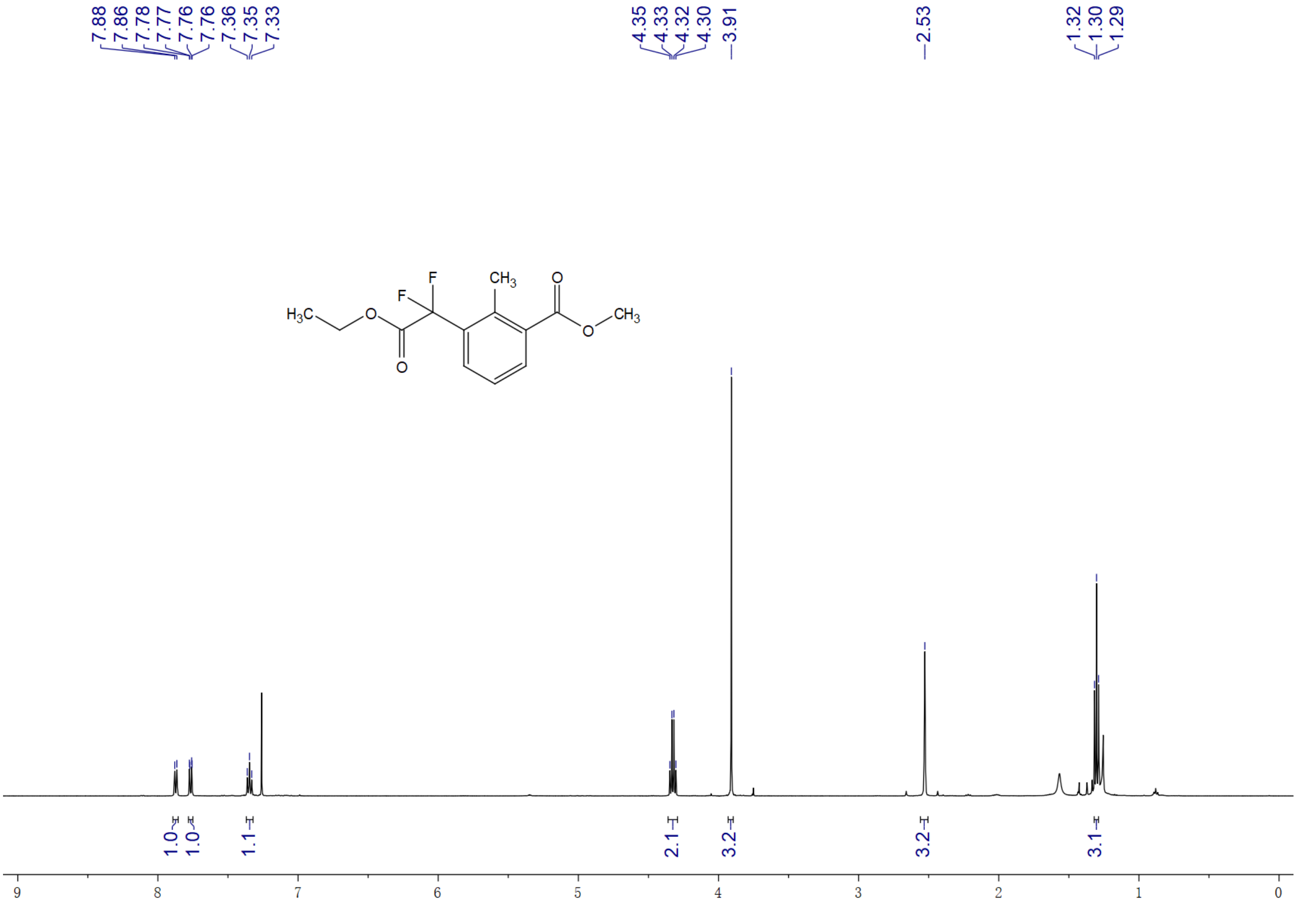

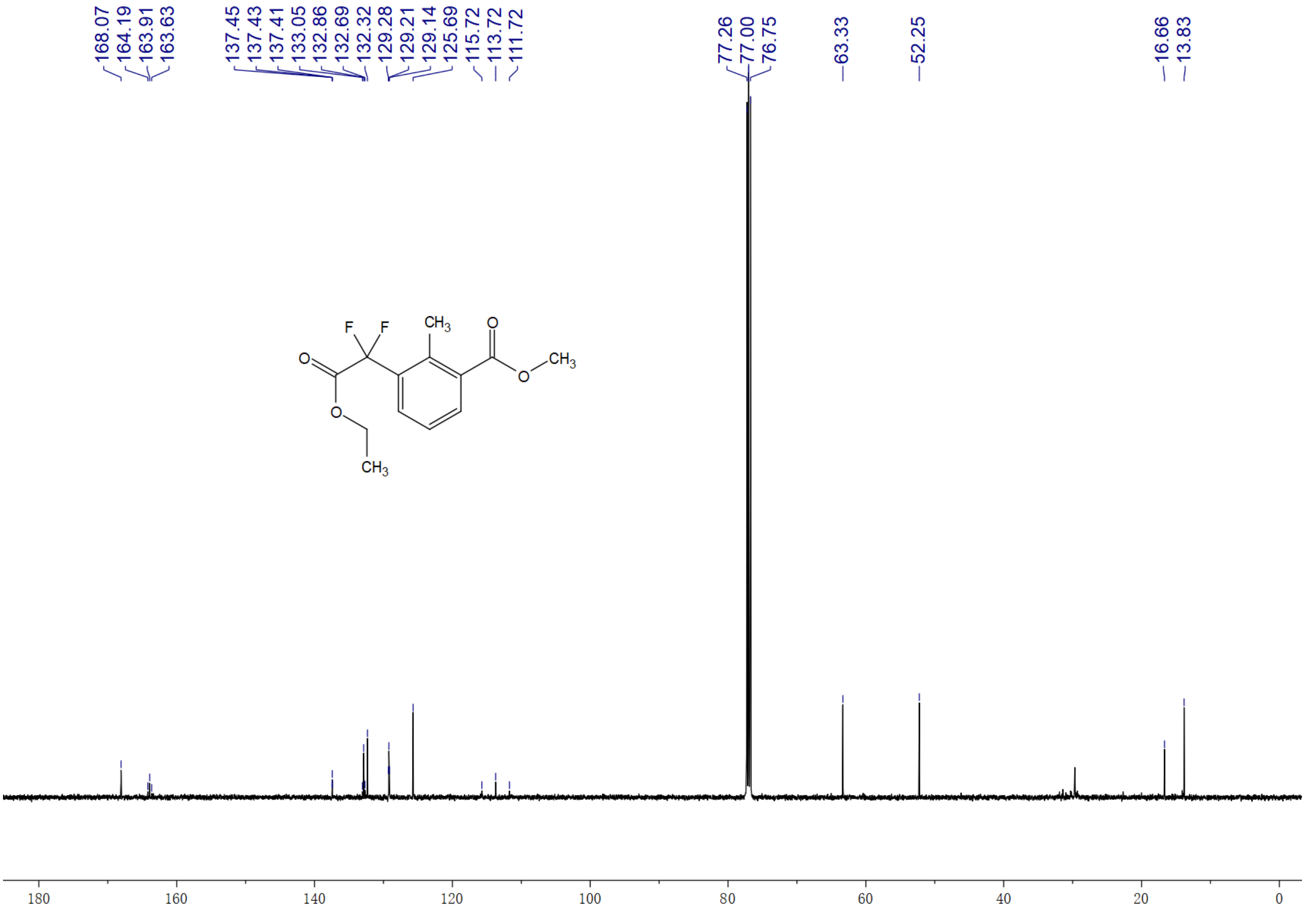

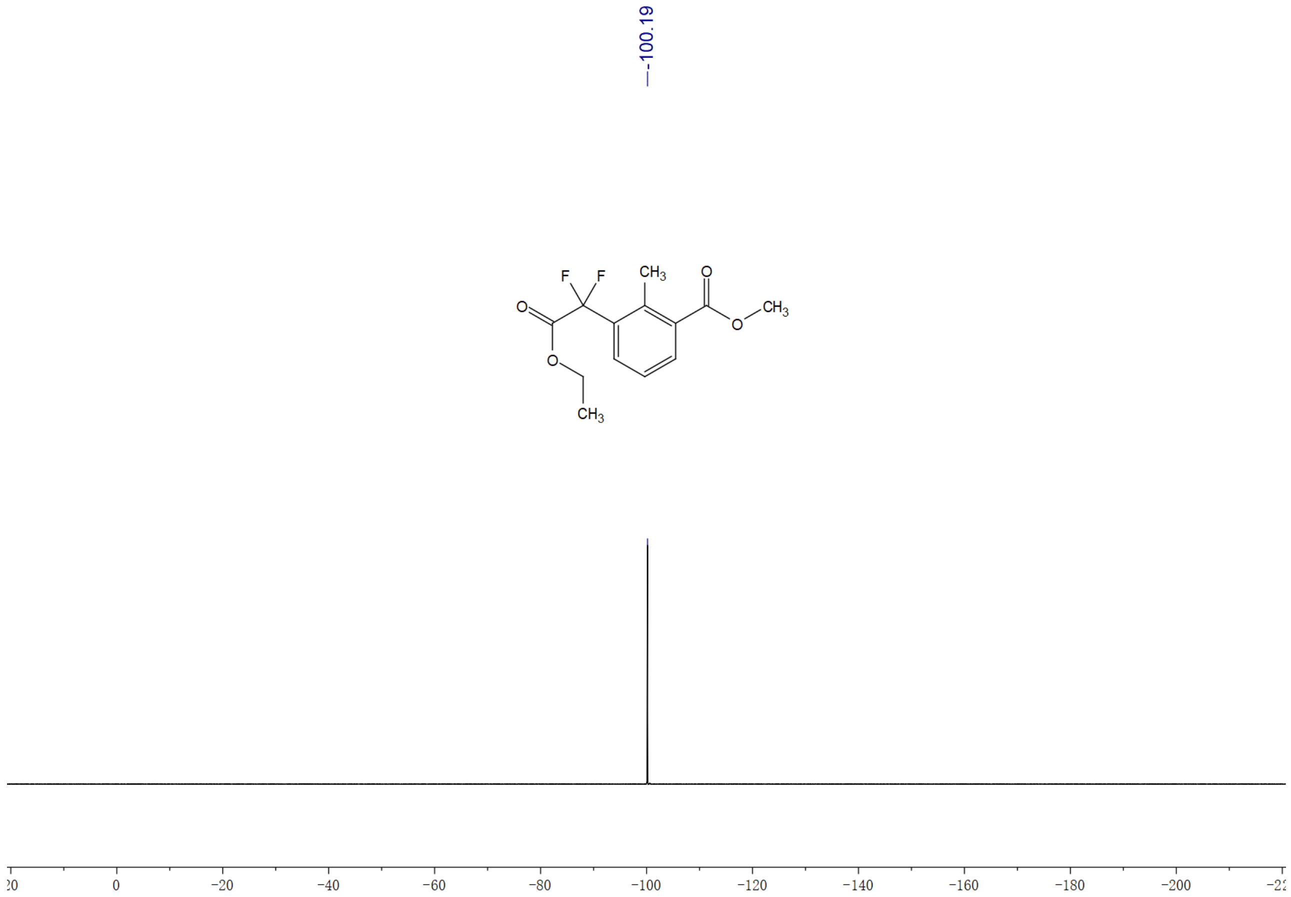
-100.19
F
F
CH3
O
O
CH3
O
O
CH3
20
0
-20
-40
-60
-80
-100
-120
-140
-160
-180
-200

Methyl-3-(2-ethoxy-1,1-difluoro-2-oxoethyl)-2,4-dimethylbenzoate, **3cb**, ^{1}H NMR (500 MHz, $CDCl_3$), ^{13}C NMR (125 MHz, $CDCl_3$) and ^{19}F NMR (471 MHz, $CDCl_3$)

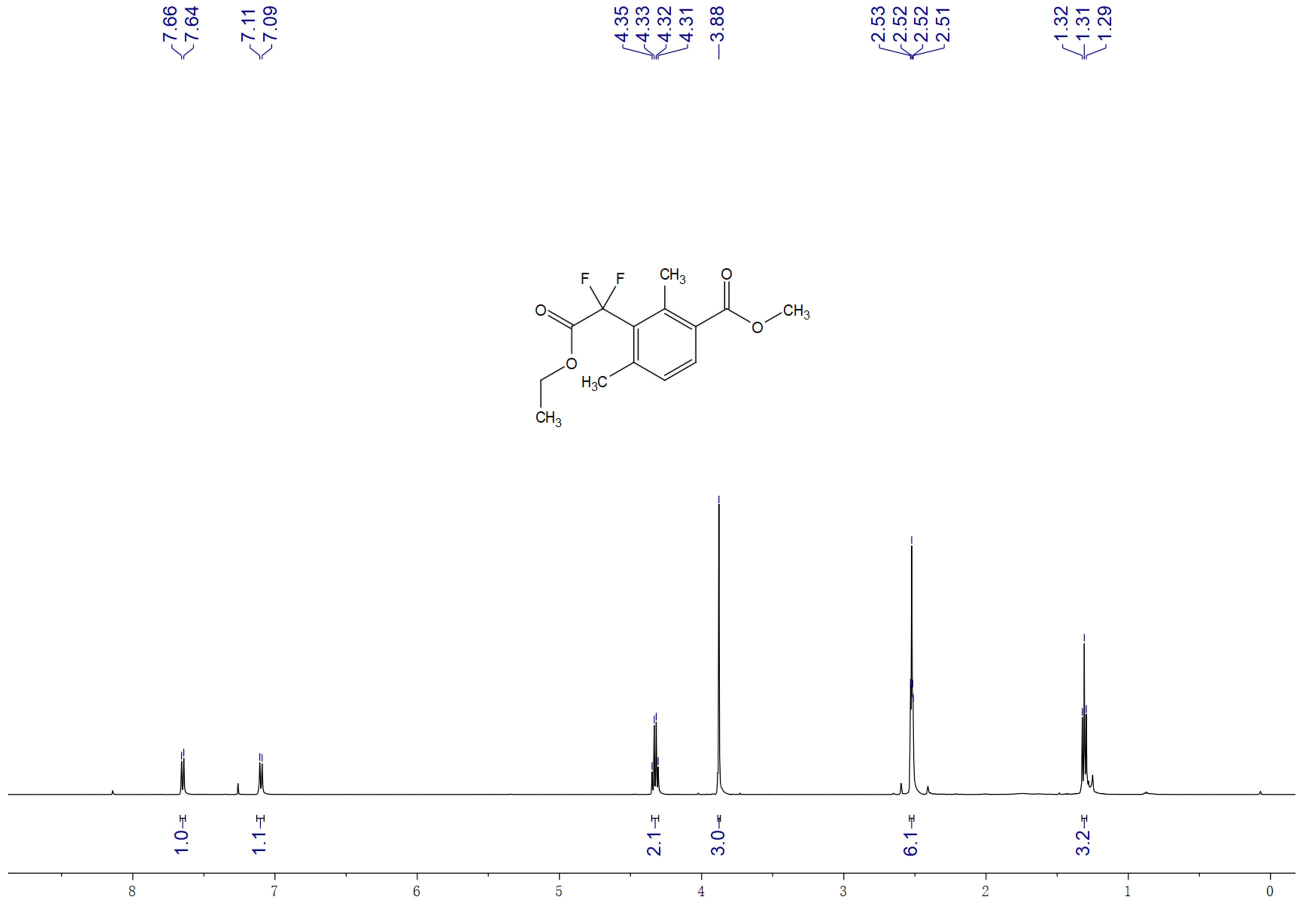

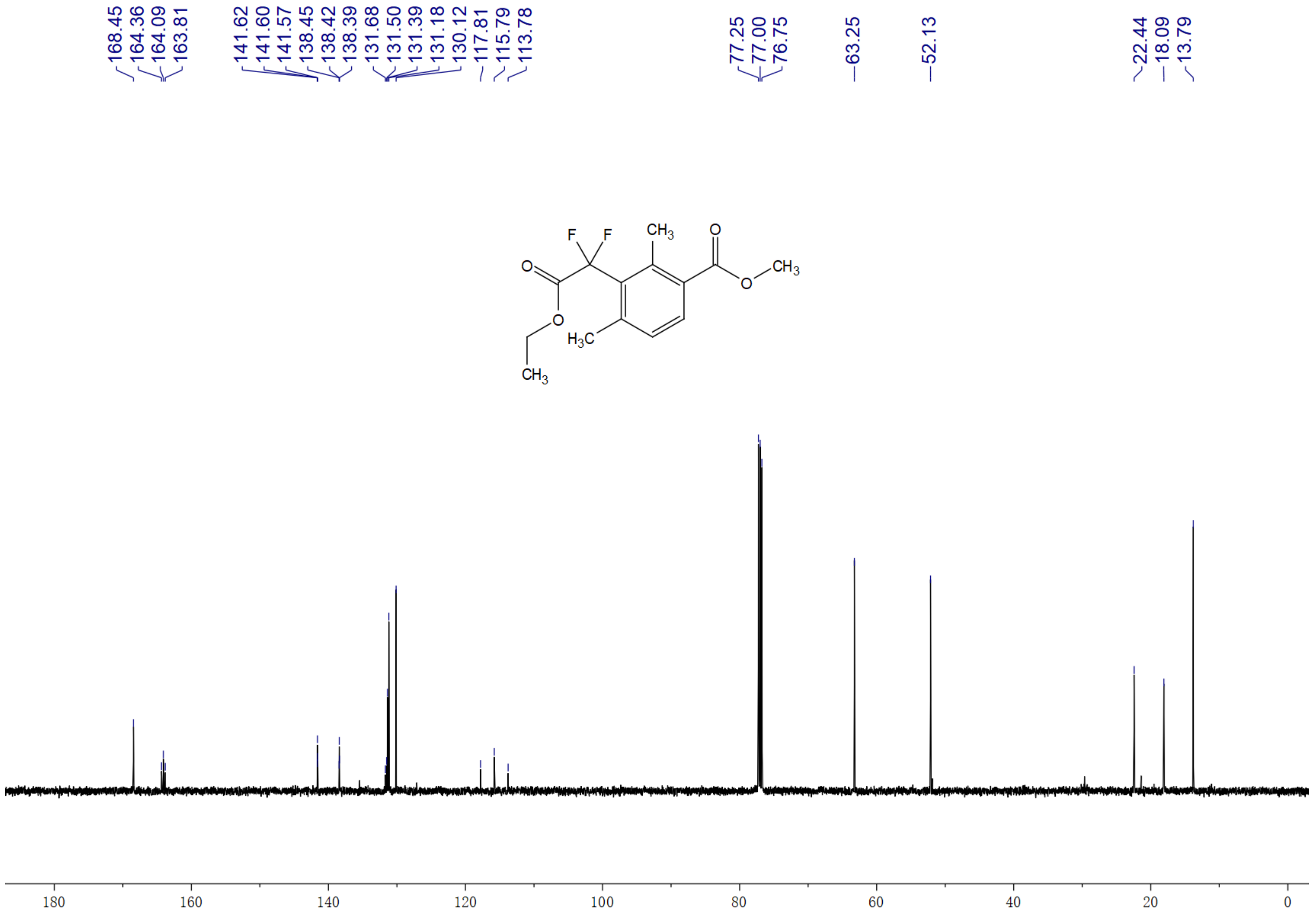

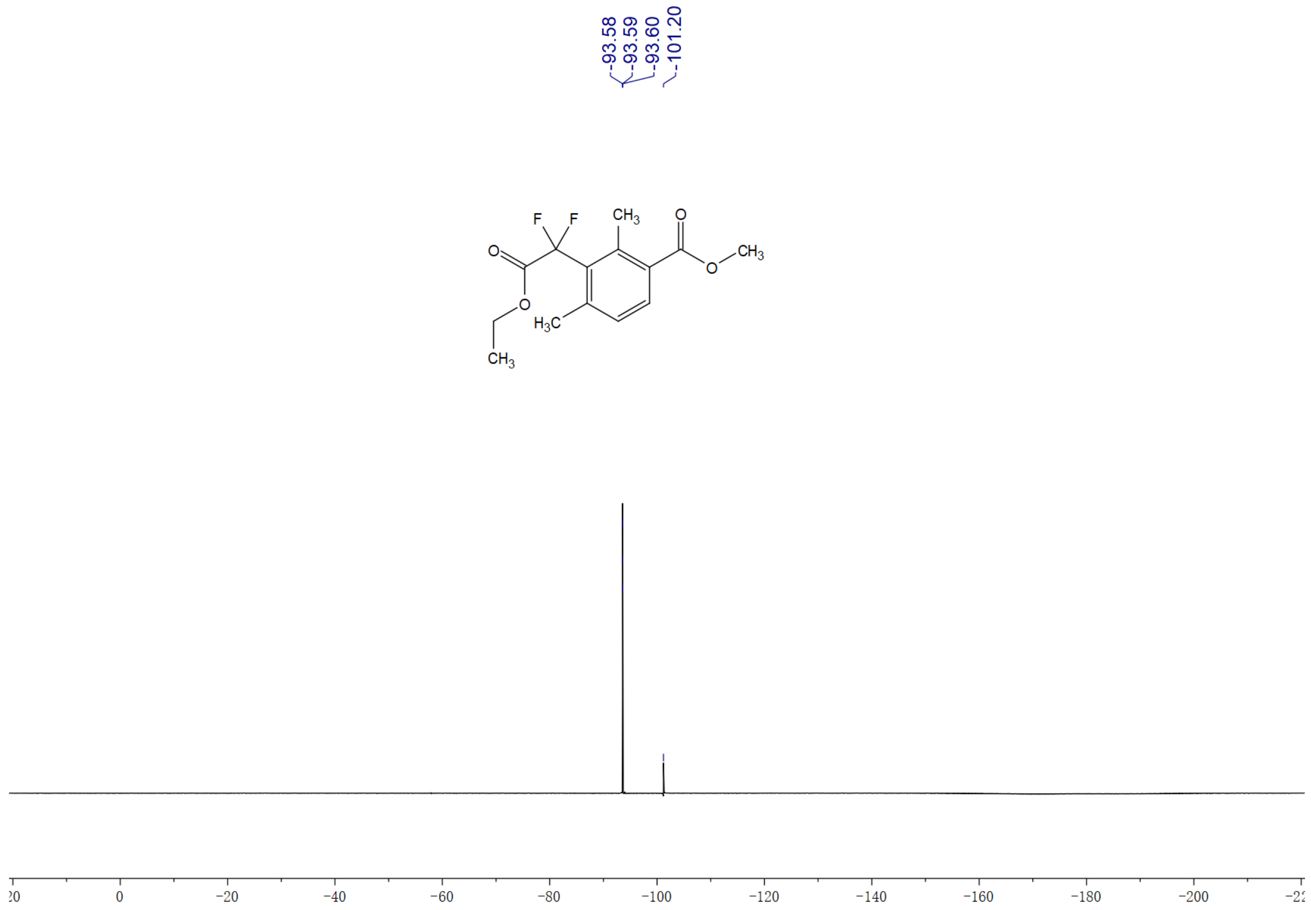

-93.58
-93.59
-93.60
-101.20
F
F
CH3
O
O
CH3
O
O
H3C
CH3
0
-20
-40
-60
-80
-100
-120
-140
-160
-180
-200

Methyl-3-(2-ethoxy-1,1-difluoro-2-oxoethyl)-5-methoxy-2-methylbenzoate, **3db**, ^{1}H NMR (500 MHz, $CDCl_3$), ^{13}C NMR (125 MHz, $CDCl_3$) and ^{19}F NMR (471 MHz, $CDCl_3$)

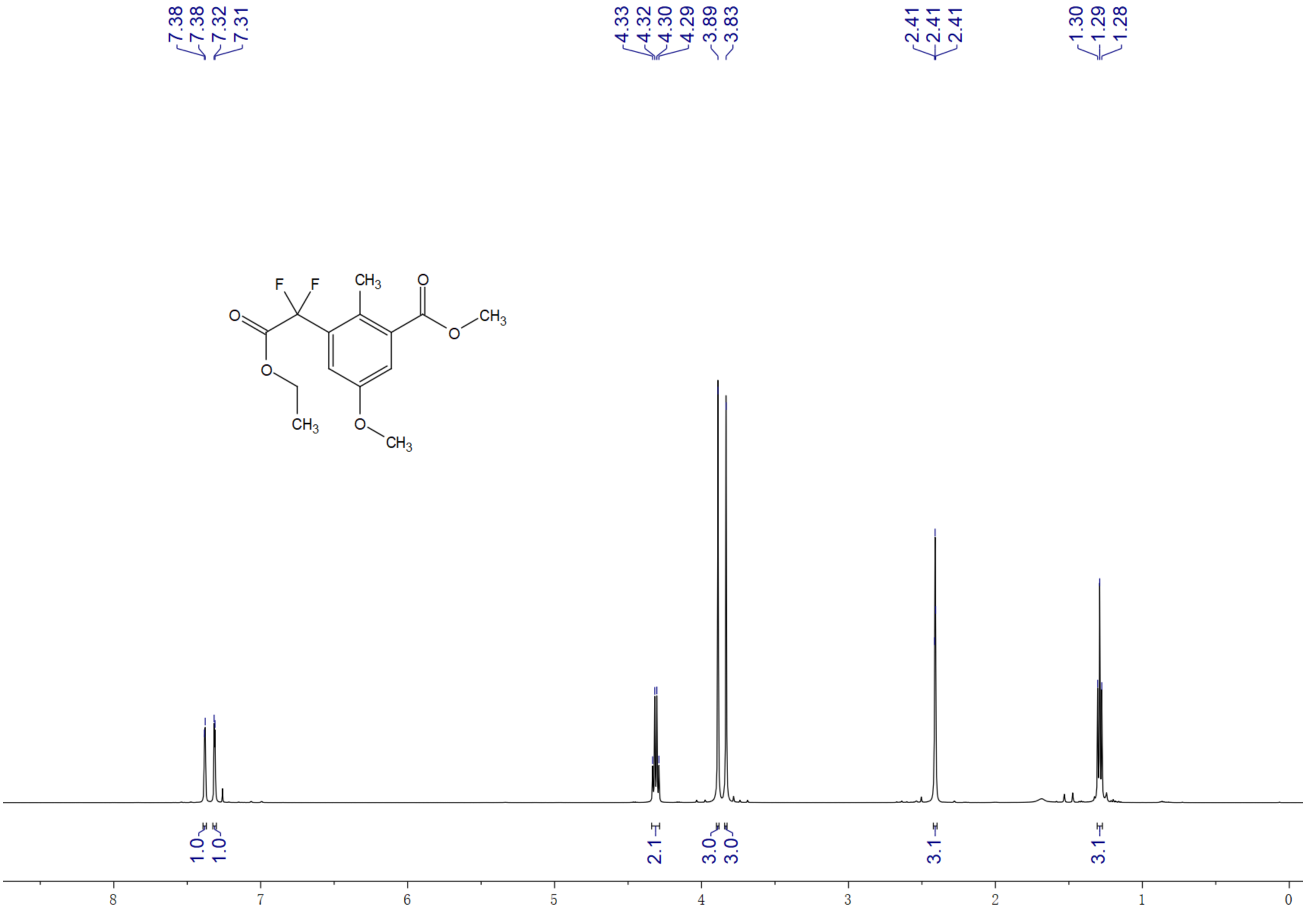

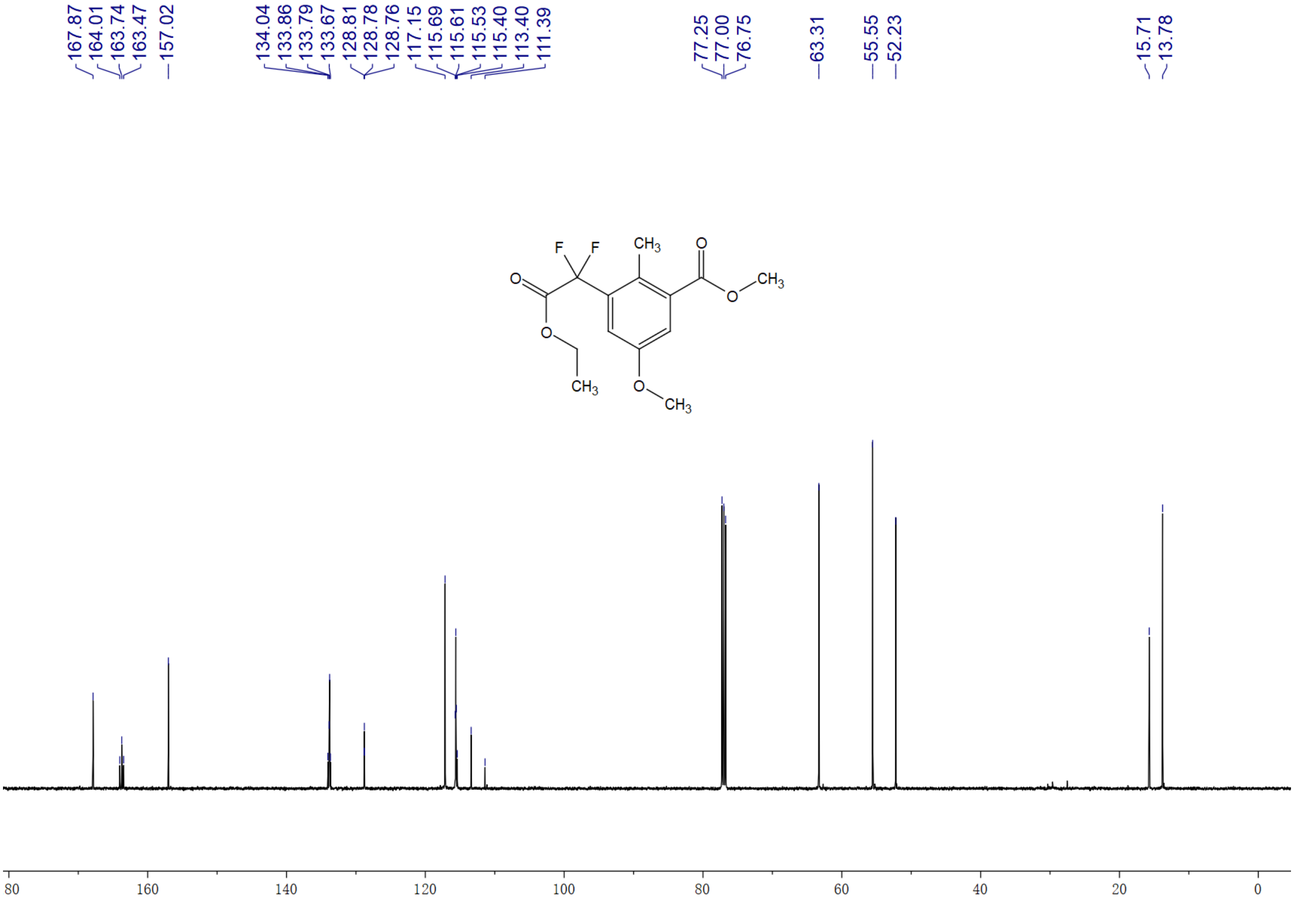

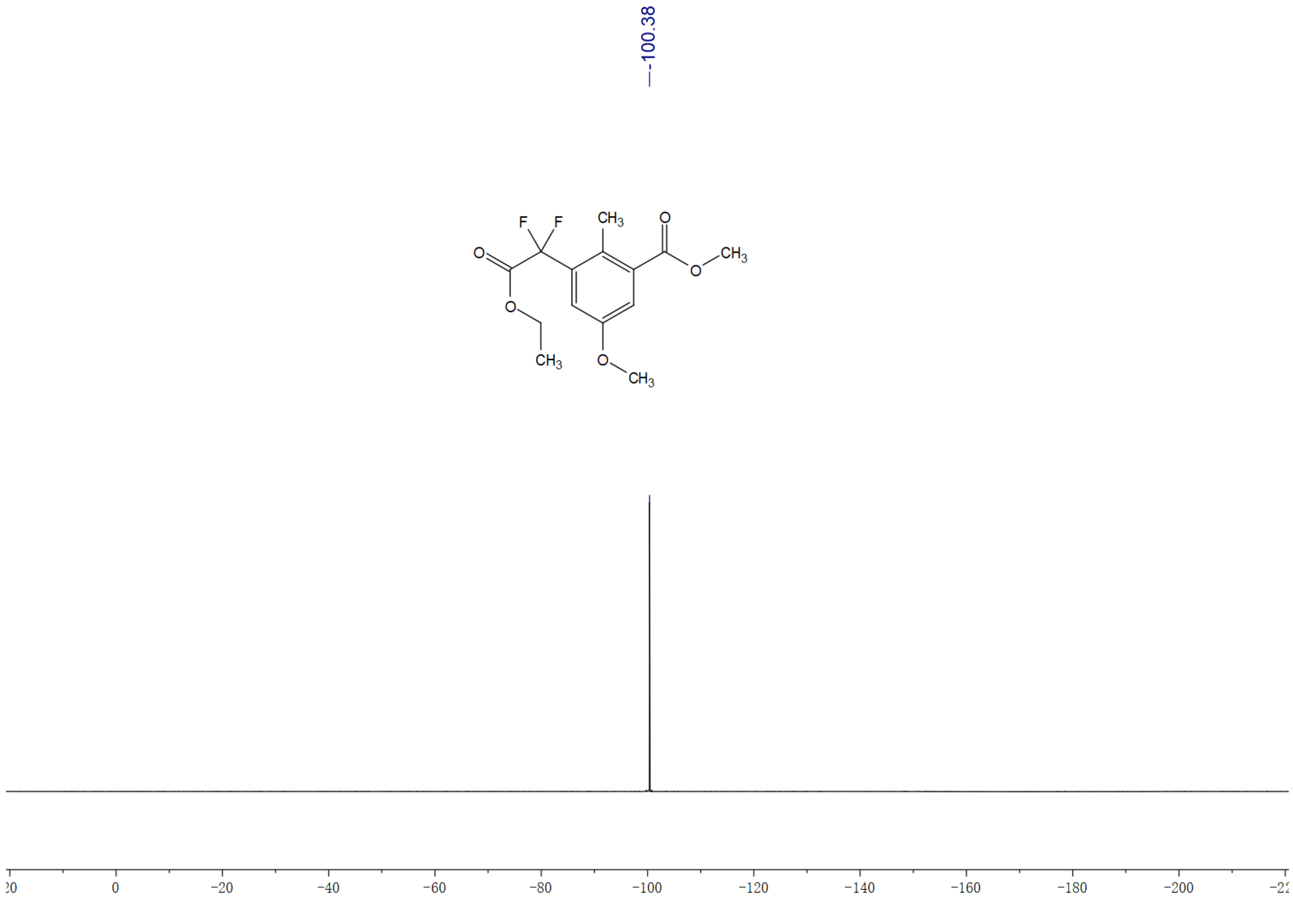

-100.38
F
F
CH3
O
O
O
CH3
O
CH3
O
CH3
20
0
−20
−40
−60
−80
−100
−120
−140
−160
−180
−200
−22

Methyl-4-(dimethylamino)-3-(2-ethoxy-1,1-difluoro-2-oxoethyl)benzoate, **3eb**, ^{1}H NMR (500 MHz, $CDCl_3$), ^{13}C NMR (125 MHz, $CDCl_3$) and ^{19}F NMR (471 MHz, $CDCl_3$)

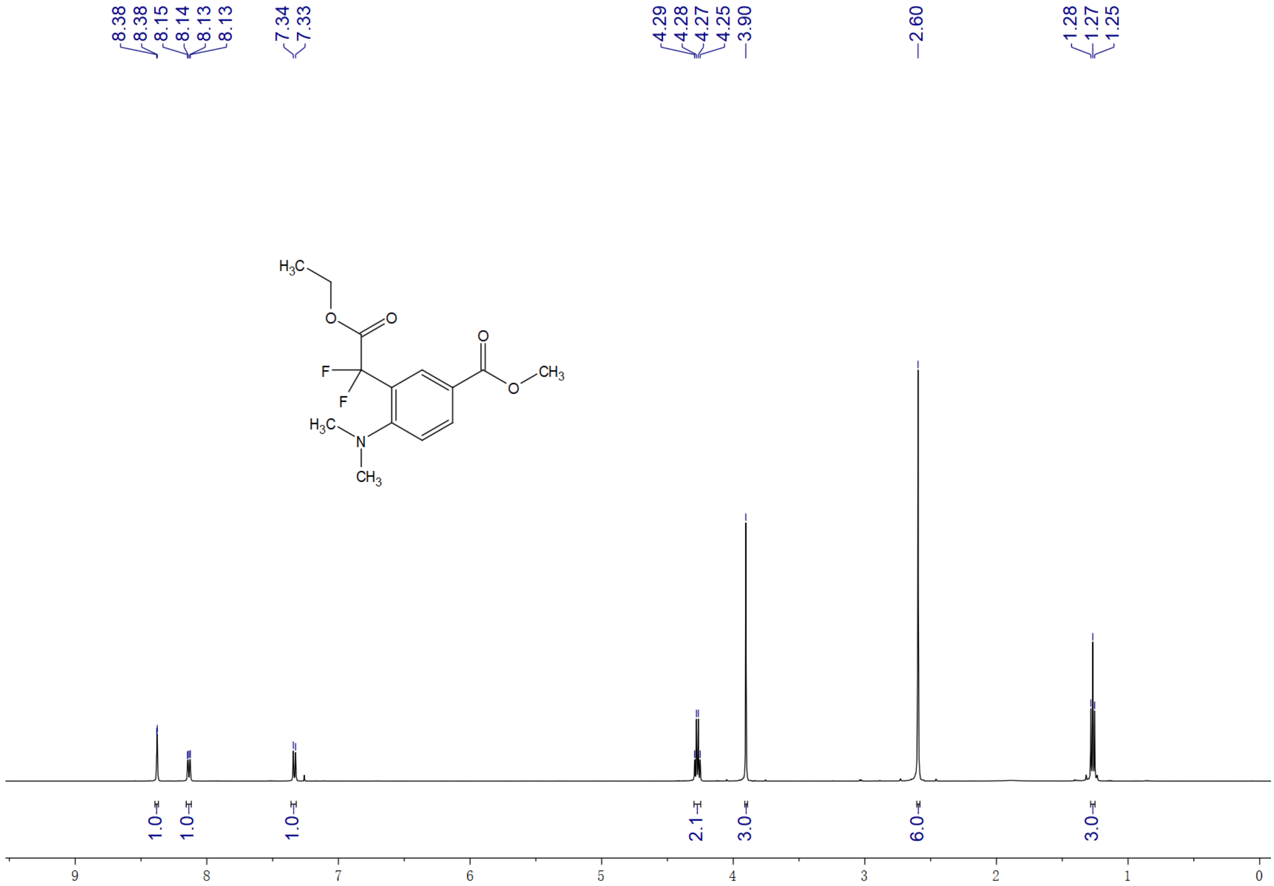

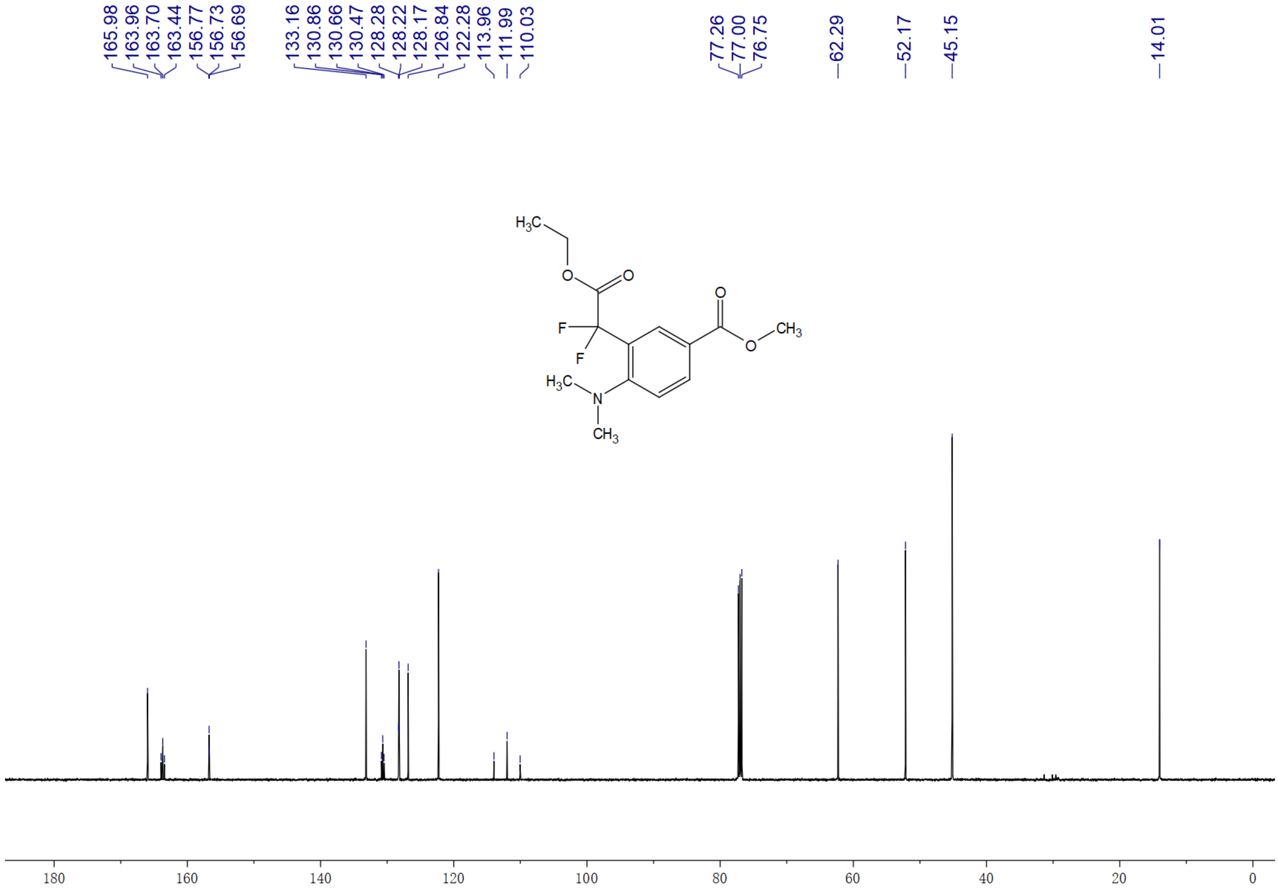

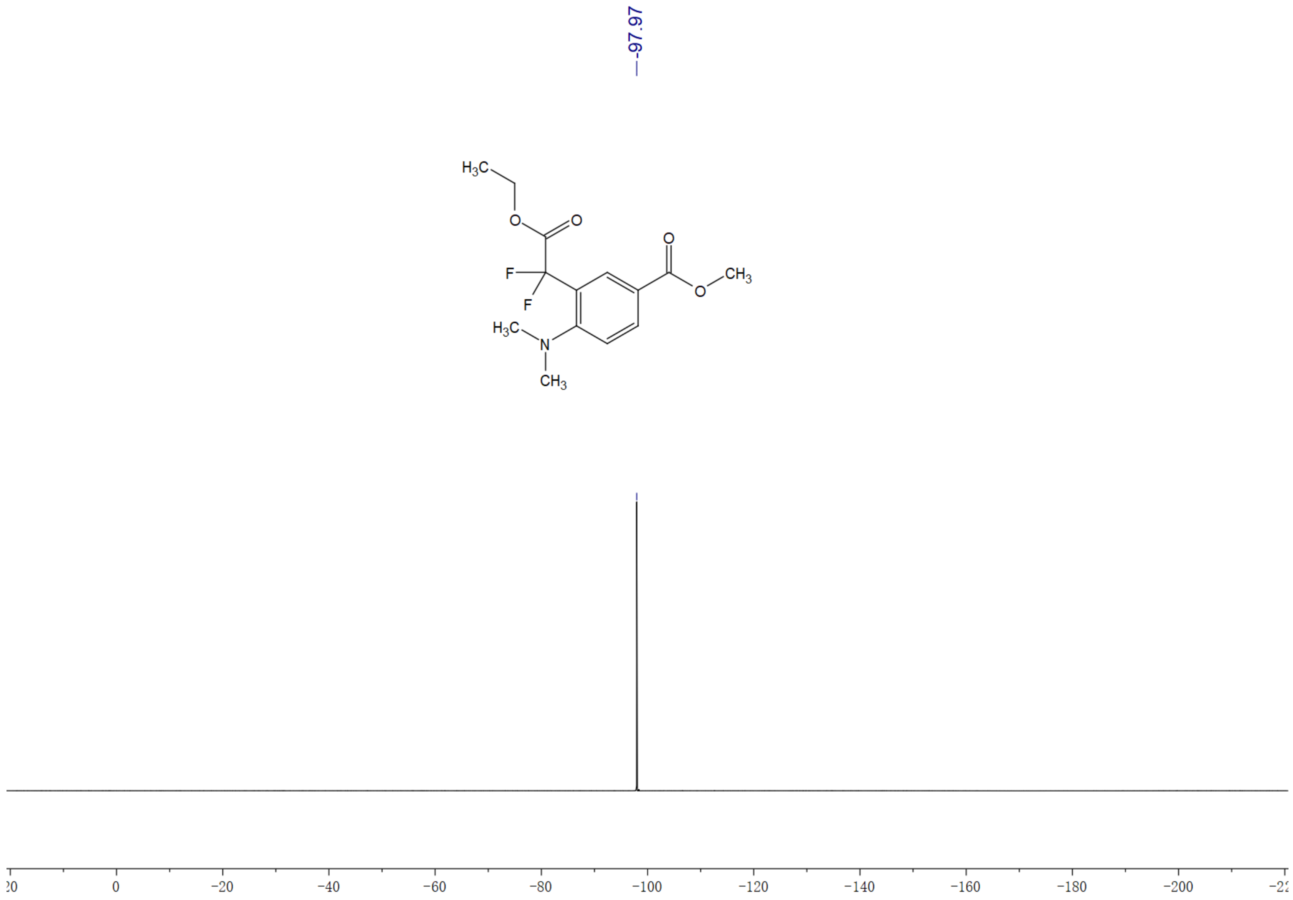
-97.97
H3C
O
O
F
F
O
O
CH3
H3C
N
CH3
20
0
-20
-40
-60
-80
-100
-120
-140
-160
-180
-200
-220

Methyl-8-(2-ethoxy-1,1-difluoro-2-oxoethyl)-2,3-dihydrobenzo[b][1,4]dioxine-6-carboxylate, **3fb**, ^{1}H NMR (500 MHz, $CDCl_3$), ^{13}C NMR (125 MHz, $CDCl_3$) and ^{19}F NMR (471 MHz, $CDCl_3$)

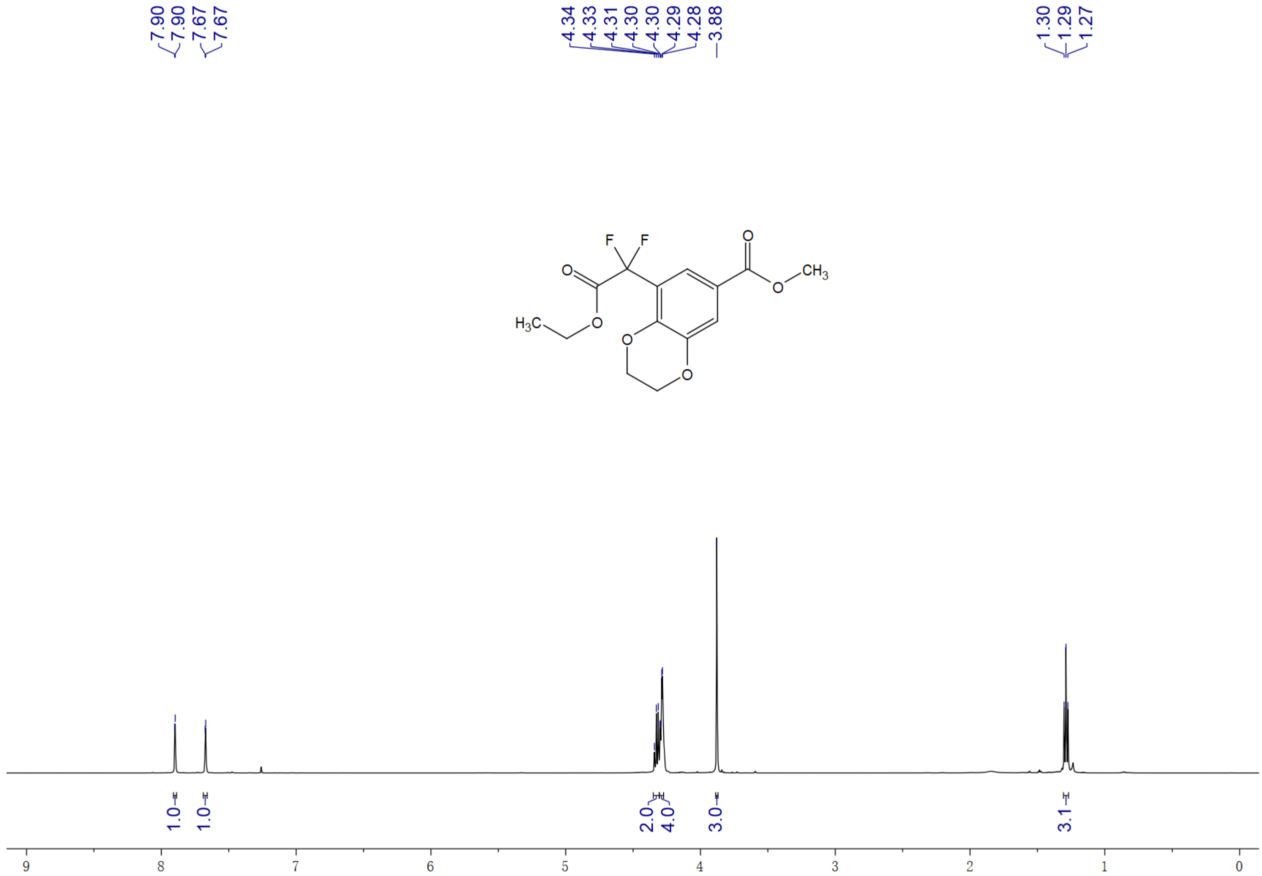

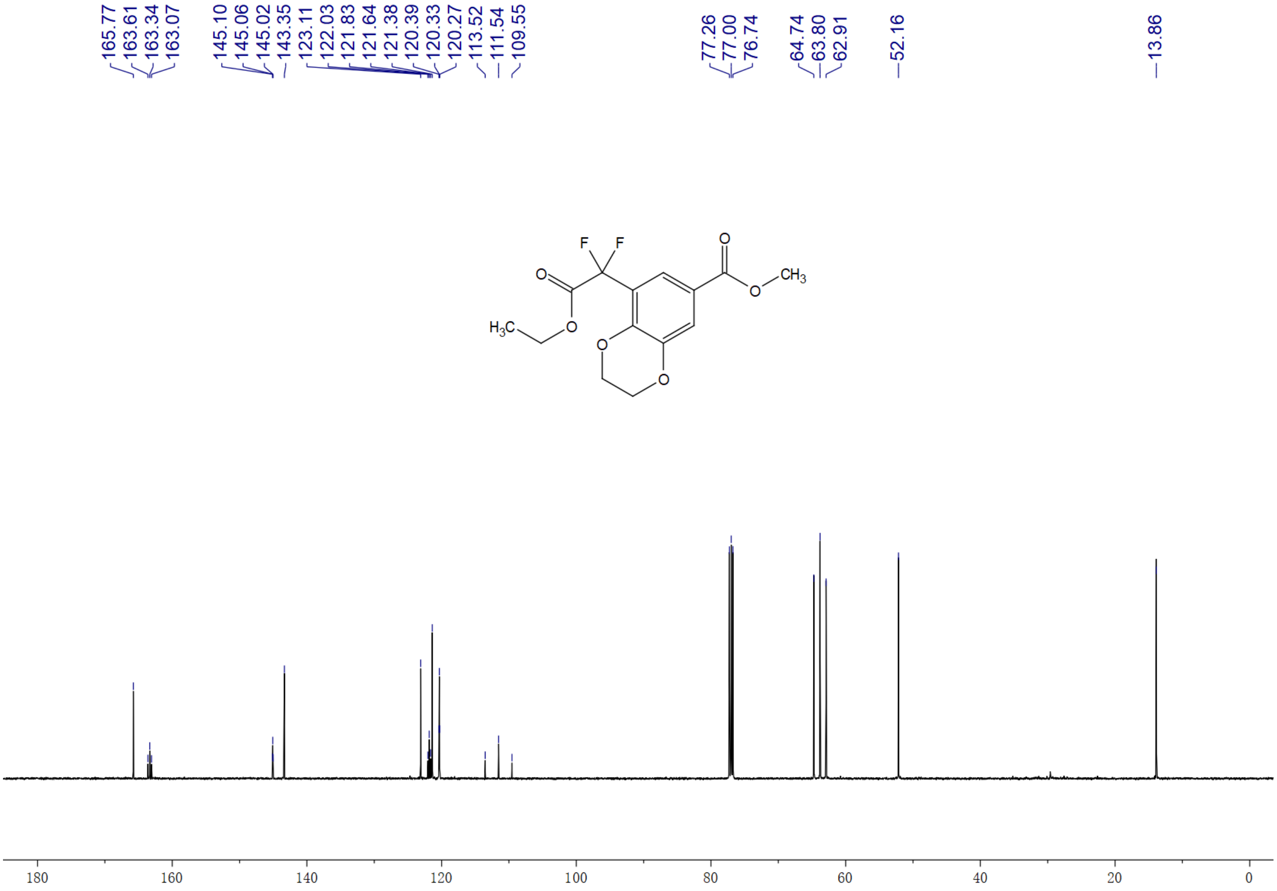

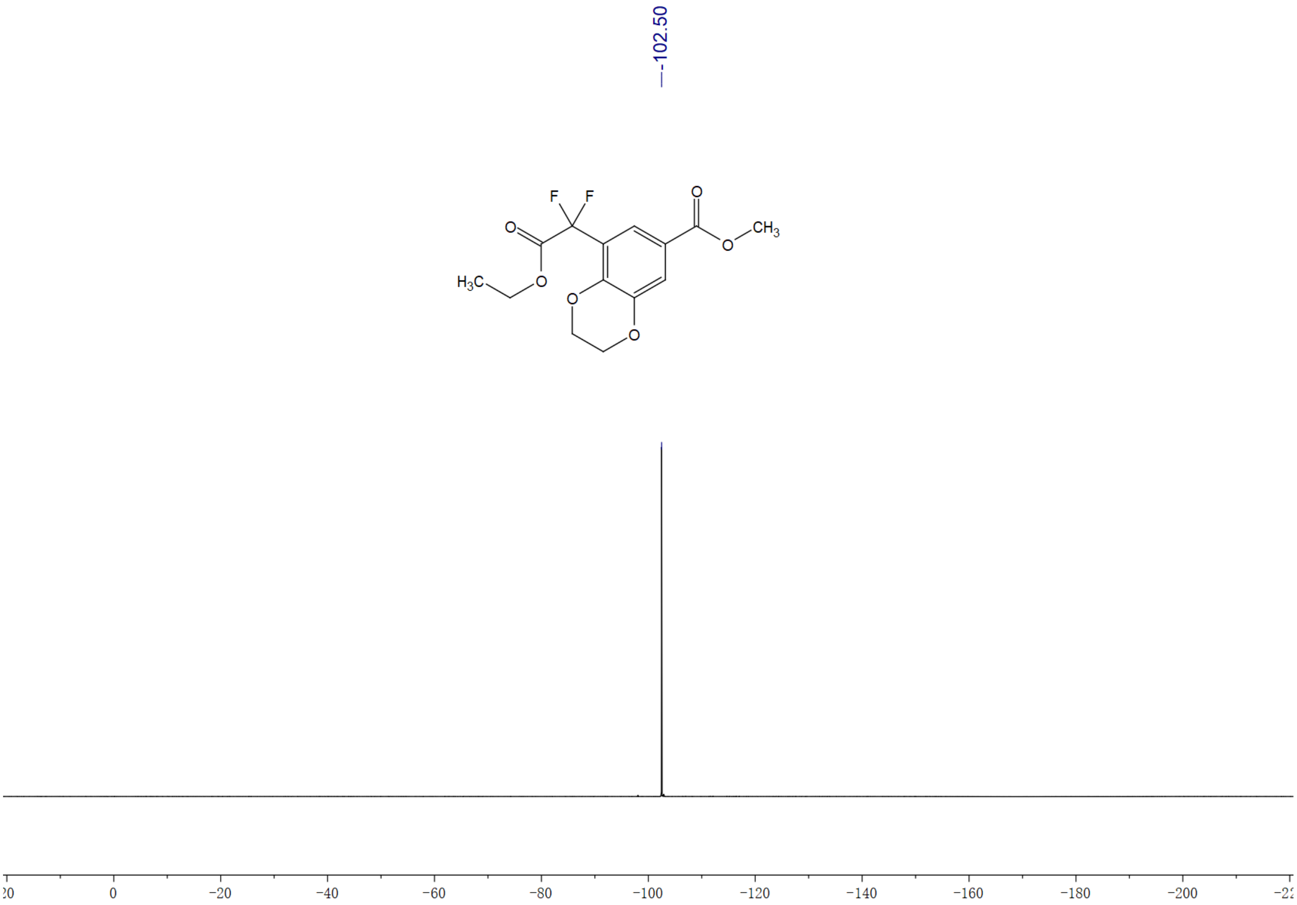

-102.50
F
F
O
O
H3C
O
O
O
O
CH3
20
0
-20
-40
-60
-80
-100
-120
-140
-160
-180
-200

Methyl-3-(1,1-difluoro-2-((1-methoxy-1-oxopropan-2-yl)amino)-2-oxoethyl)-2-methylbenzoate, **3gb**, ^{1}H NMR (500 MHz, $CDCl_3$), ^{13}C NMR (125 MHz, $CDCl_3$) and ^{19}F NMR (471 MHz, $CDCl_3$)

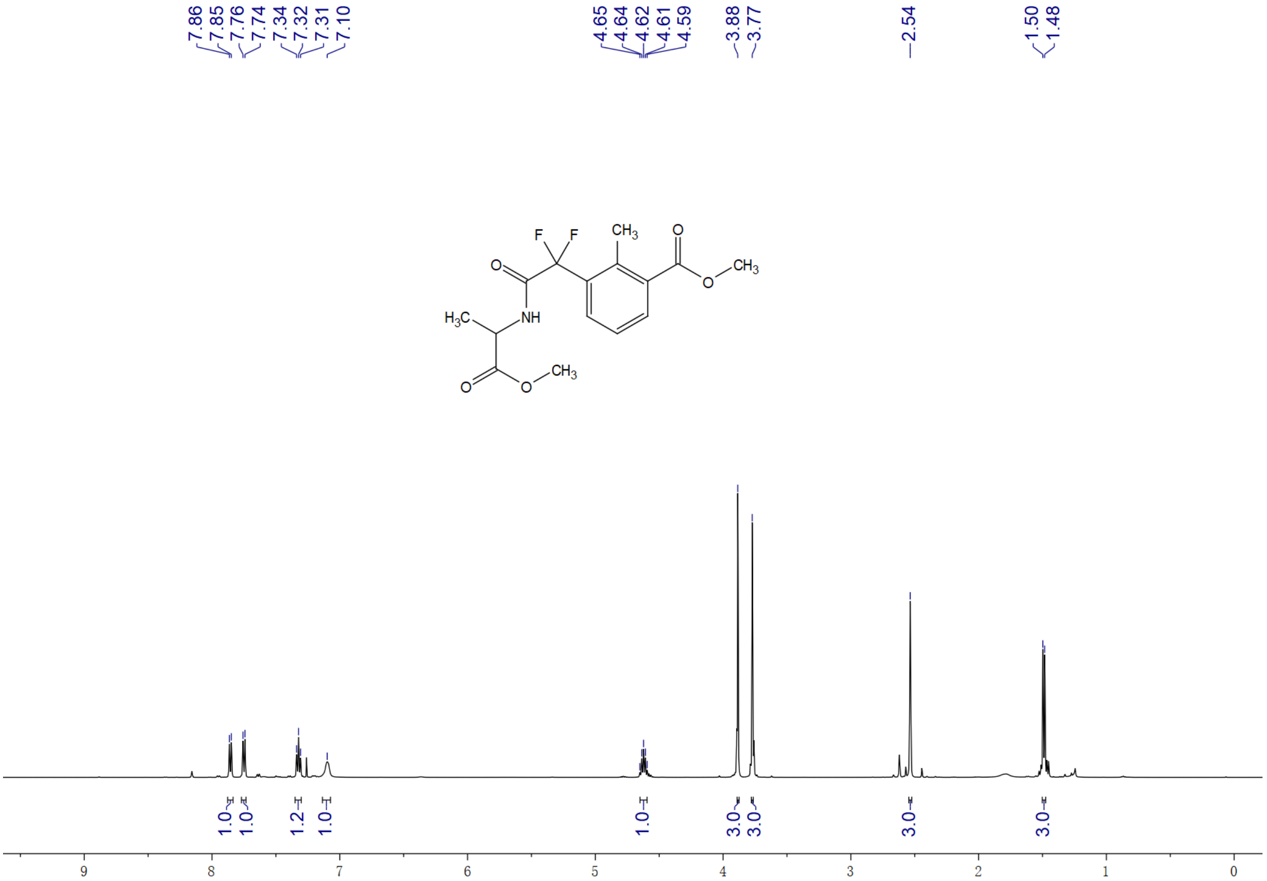

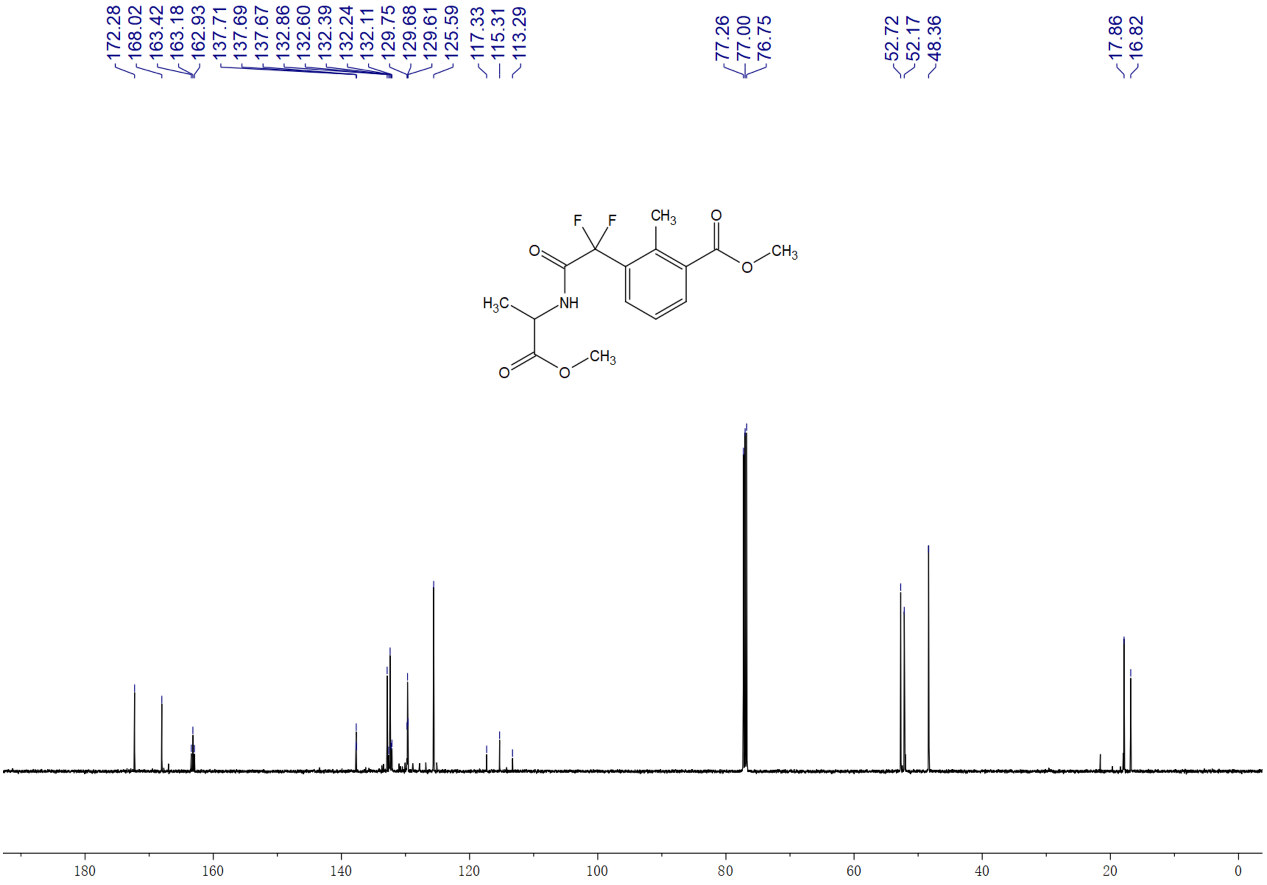

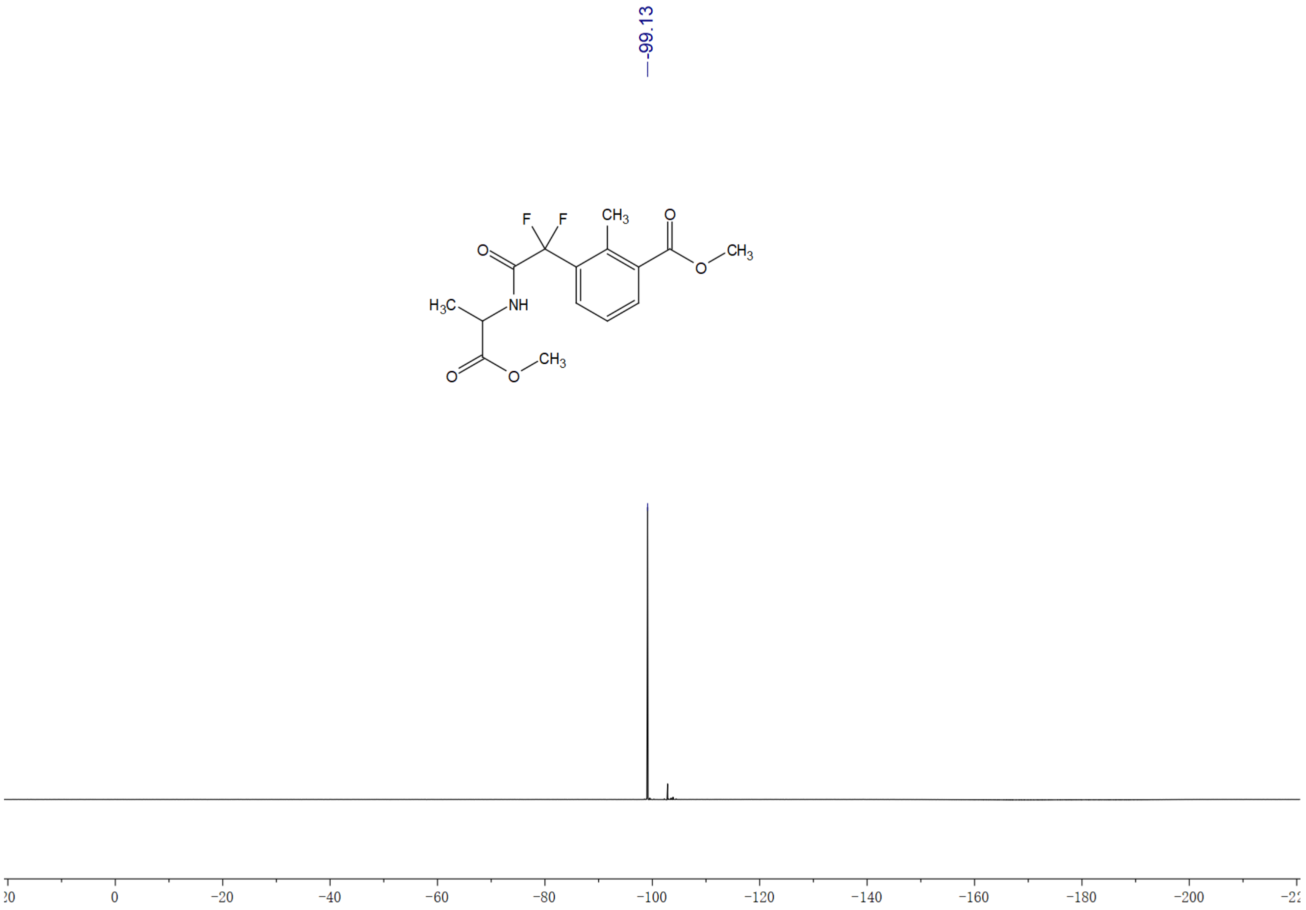

-99.13
F F
CH3
O
O
CH3
O
NH
H3C
O
O
CH3
20 0 −20 −40 −60 −80 −100 −120 −140 −160 −180 −200 −22

Methyl-(2,2-difluoro-2-(3-(methoxycarbonyl)-2-methylphenyl)acetyl)prolinate, **3hb**, ^{1}H NMR (500 MHz, CDCl$_3$), ^{13}C NMR (125 MHz, CDCl$_3$) and ^{19}F NMR (471 MHz, CDCl$_3$)

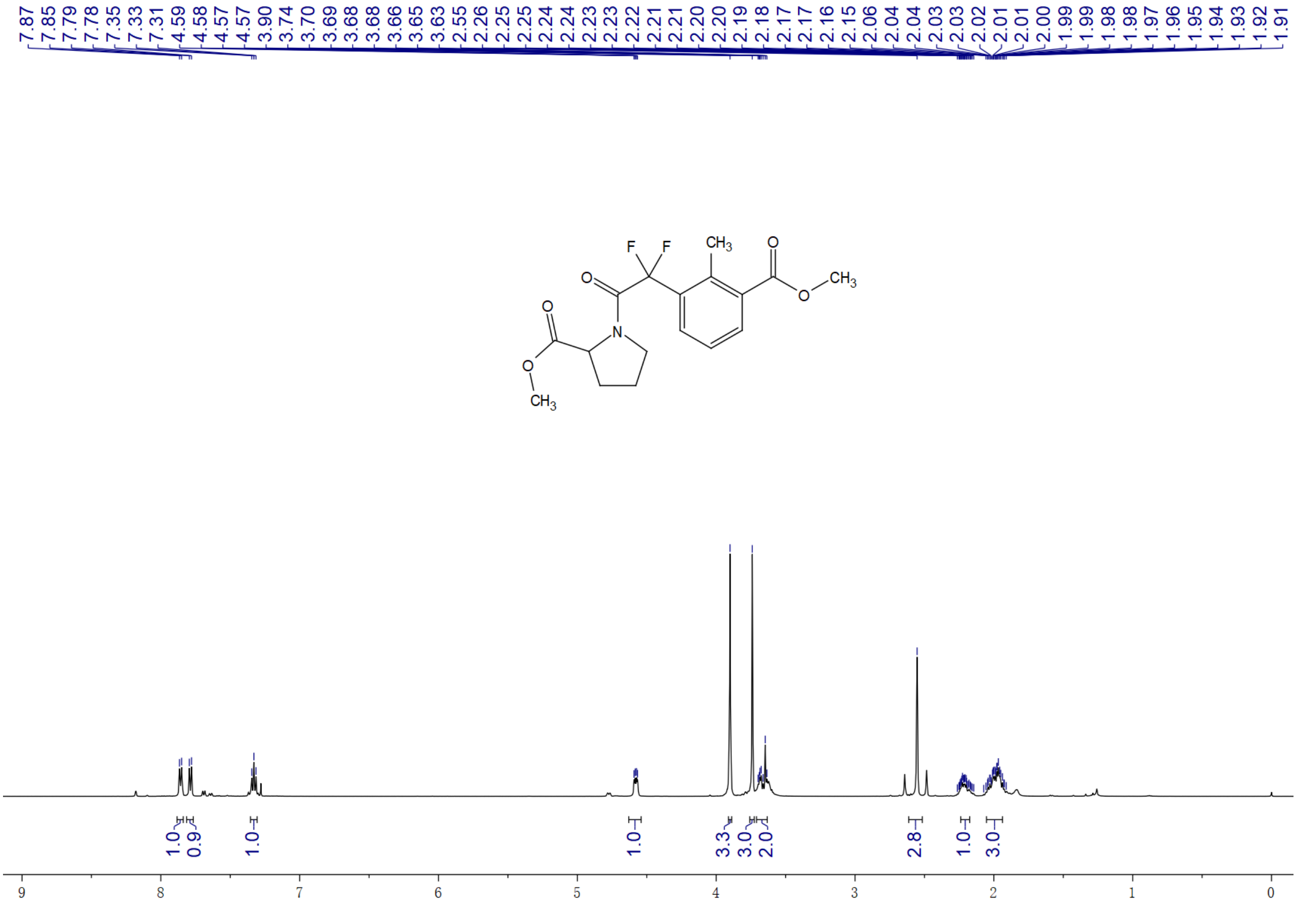

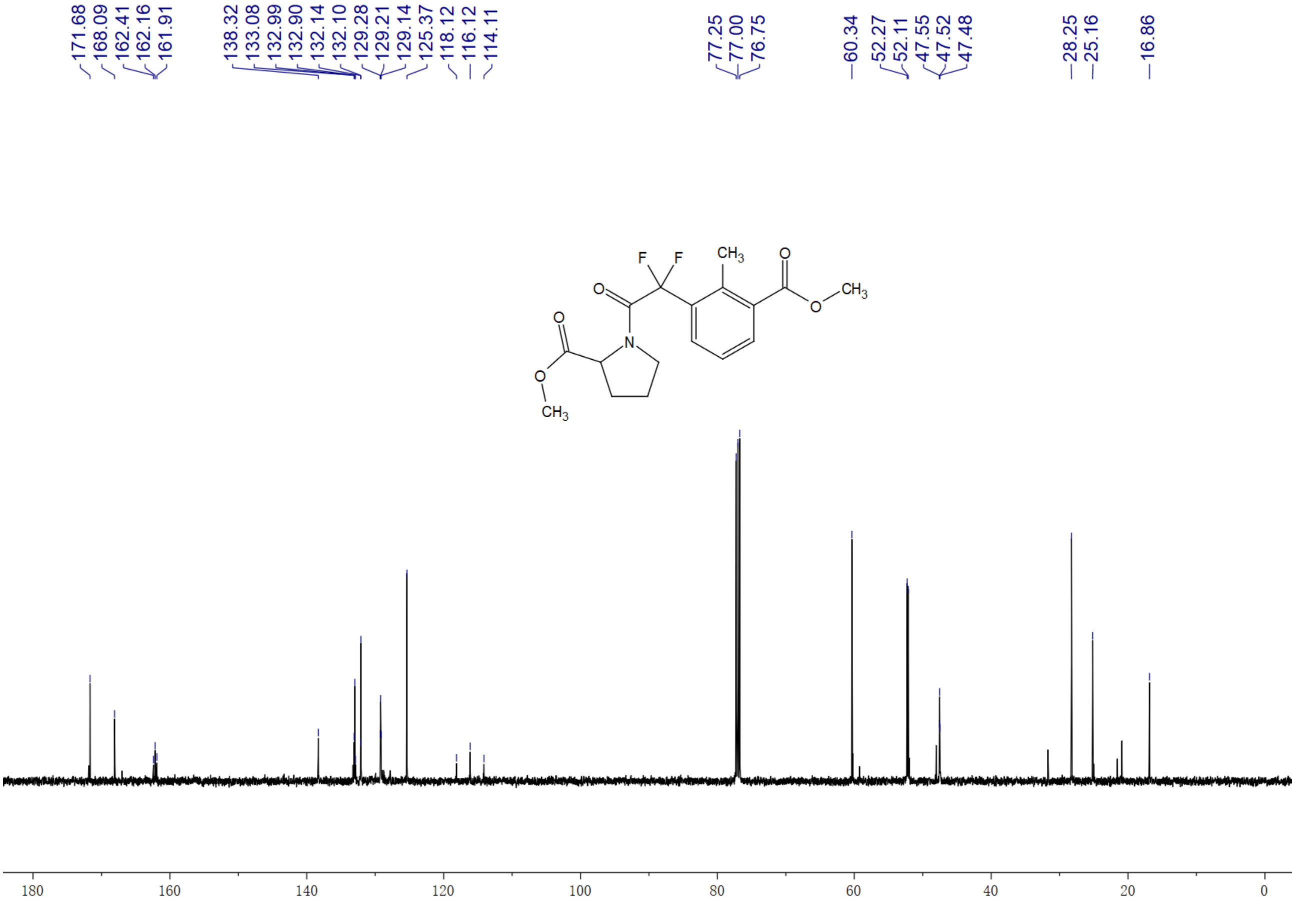

Dimethyl-(2,2-difluoro-2-(3-(methoxycarbonyl)-2-methylphenyl)acetyl)aspartate, **3ib**, ^{1}H NMR (500 MHz, $CDCl_3$), ^{13}C NMR (125 MHz, $CDCl_3$) and ^{19}F NMR (471 MHz, $CDCl_3$)

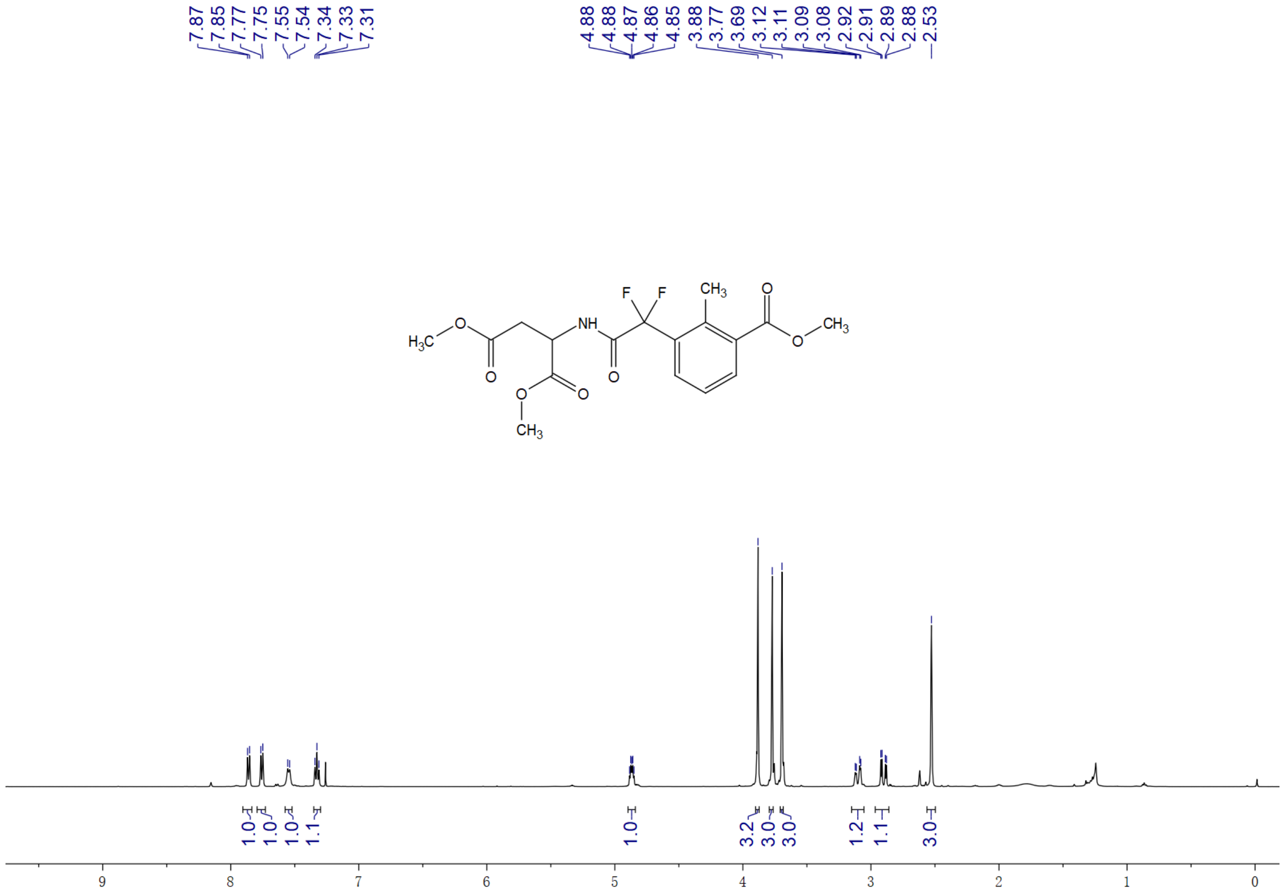

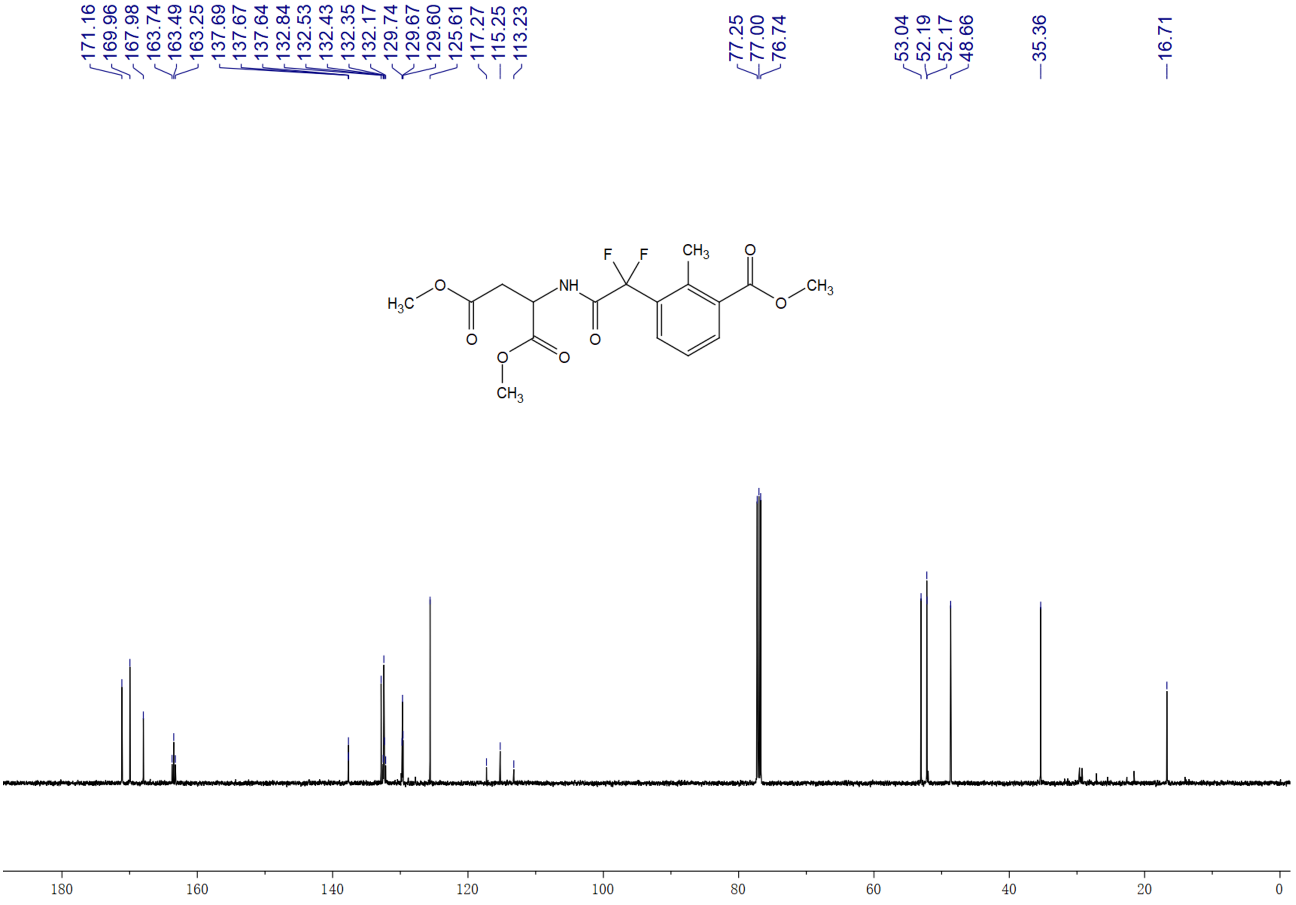

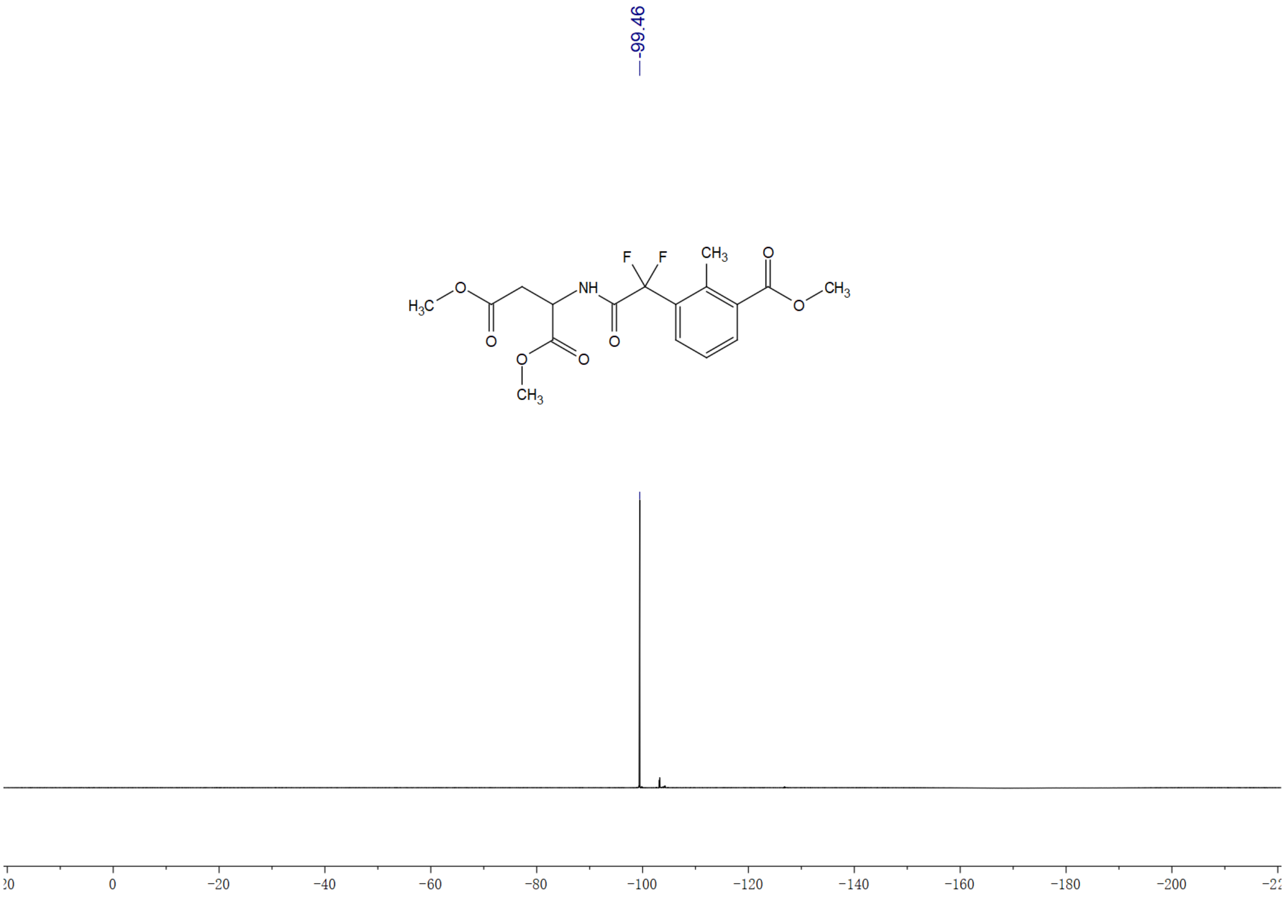
-99.46
F
F
CH3
O
NH
H3C
O
O
O
O
O
CH3
O
CH3
O
20
0
−20
−40
−60
−80
−100
−120
−140
−160
−180
−200

Methyl-3-(2-((3-(tert-butoxy)-1-methoxy-1-oxopropan-2-yl)amino)-1,1-difluoro-2-oxoethyl)-2-methylbenzoate, **3jb**, ^{1}H NMR (500 MHz, $CDCl_3$), ^{13}C NMR (125 MHz, $CDCl_3$) and ^{19}F NMR (471 MHz, $CDCl_3$)

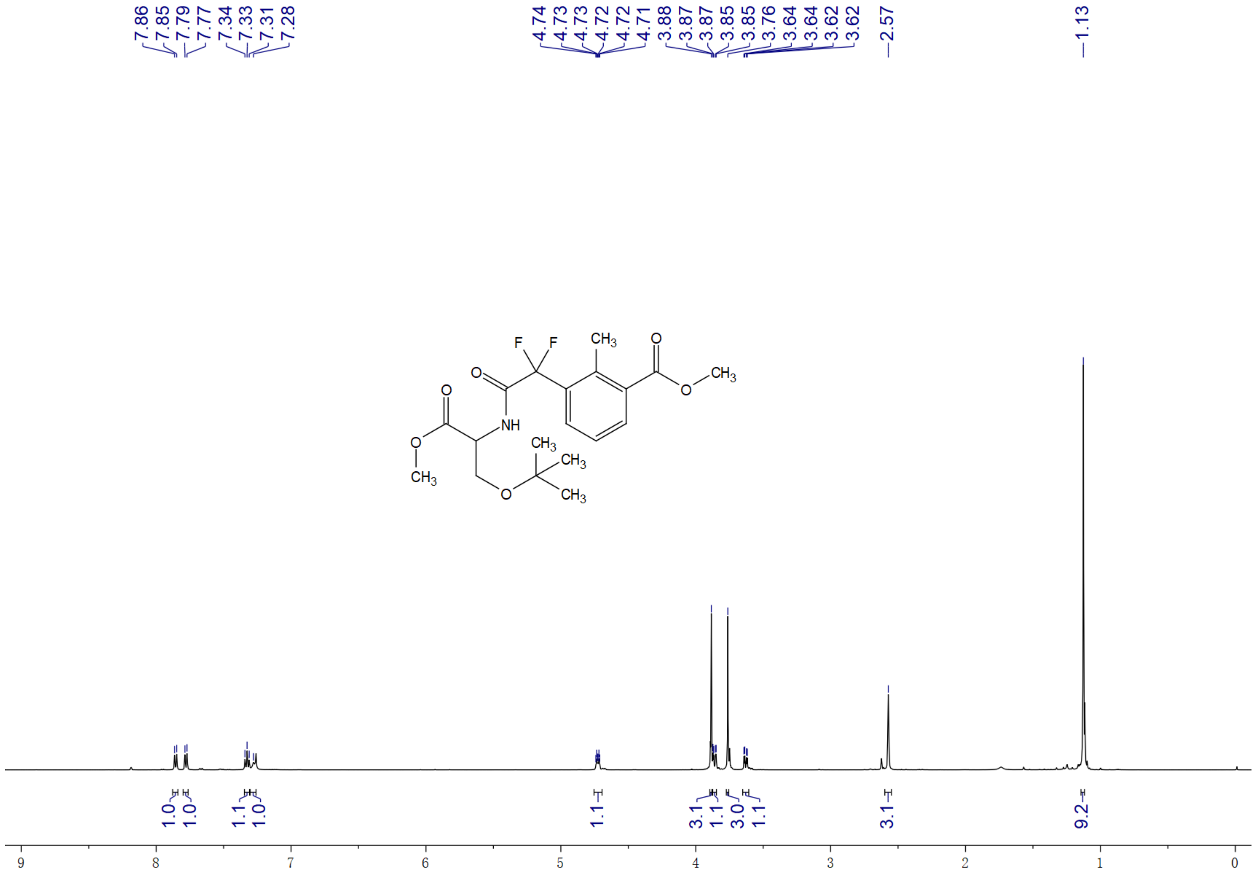

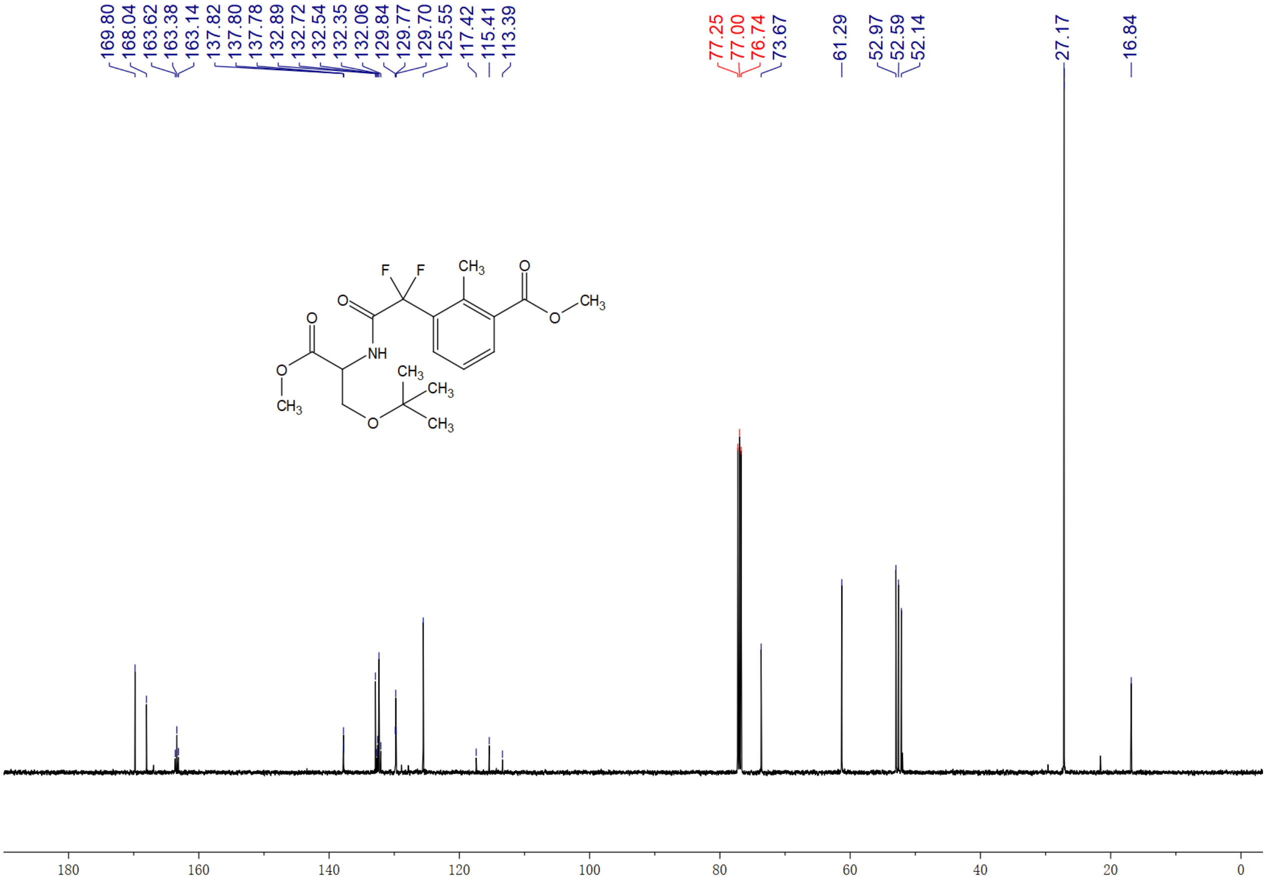

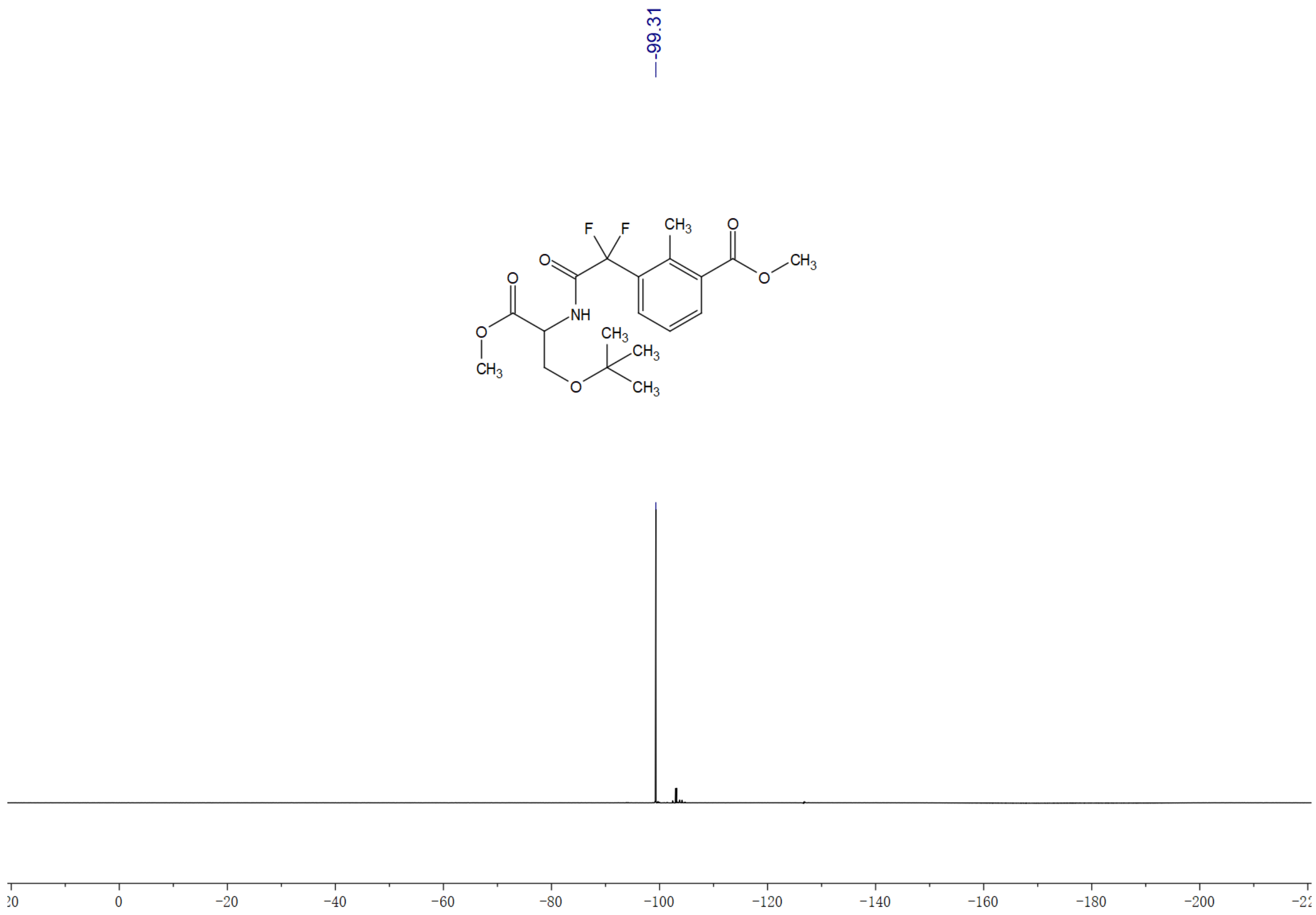

-99.31
F
F
CH3
O
O
CH3
O
O
NH
O
CH3
CH3
CH3
CH3
O
20
0
-20
-40
-60
-80
-100
-120
-140
-160
-180
-200

3-(1,1-Difluoro-2-oxo-2-(phenylamino)ethyl)-2-methylbenzoic acid, **3kb**, ^{1}H NMR (500 MHz, DMSO-d_6), ^{13}C NMR (125 MHz, DMSO-d_6) and ^{19}F NMR (471 MHz, DMSO)

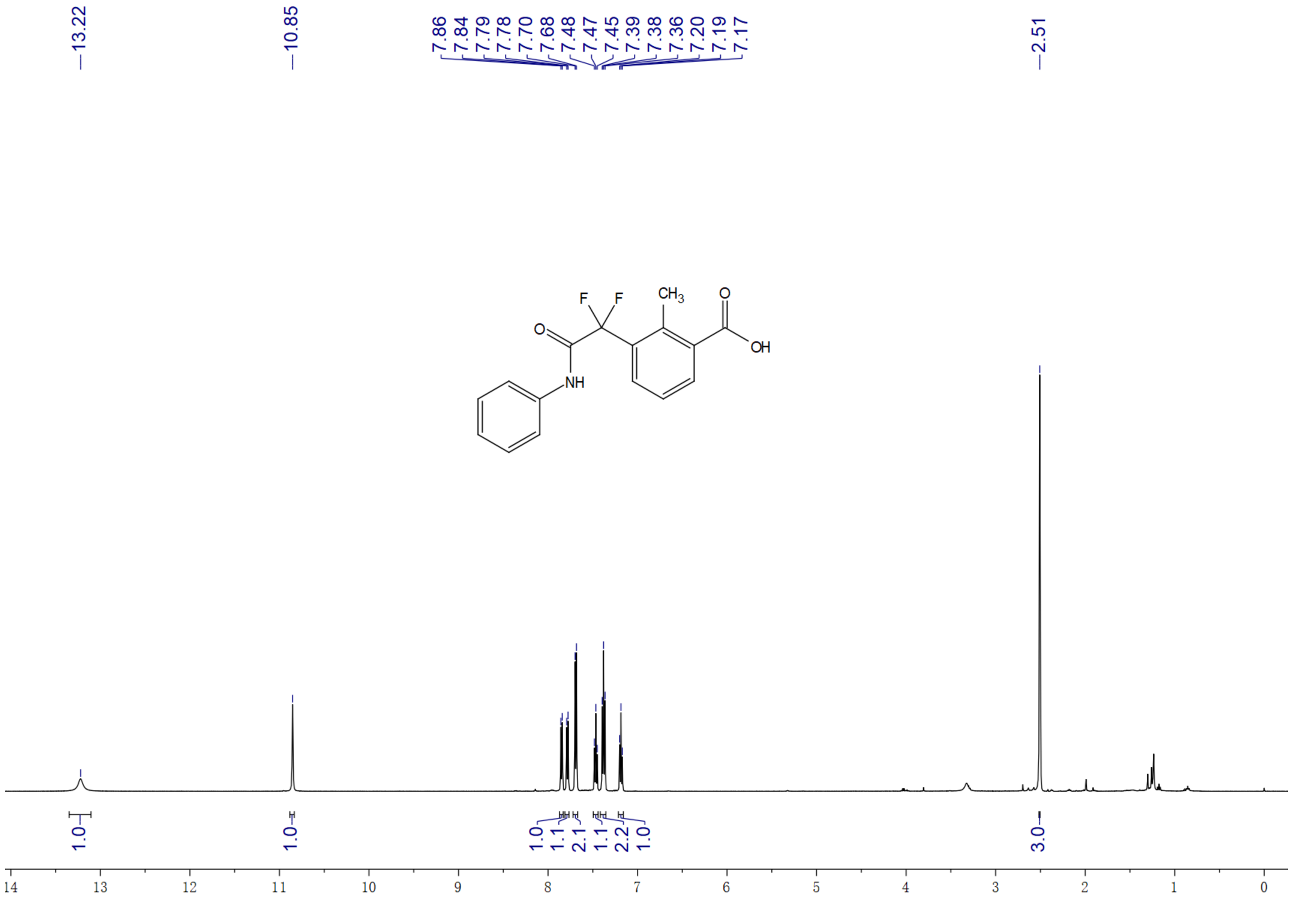

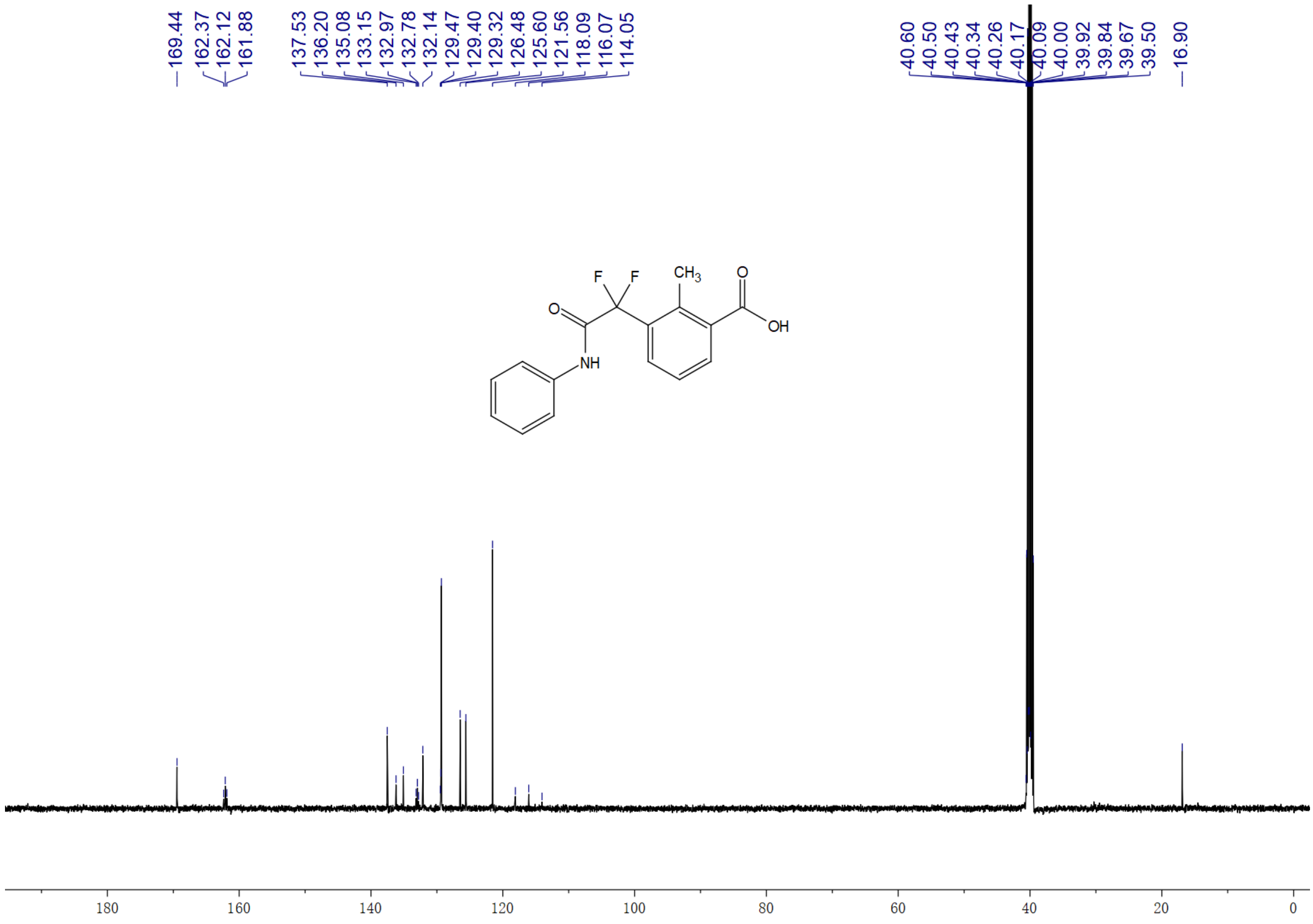

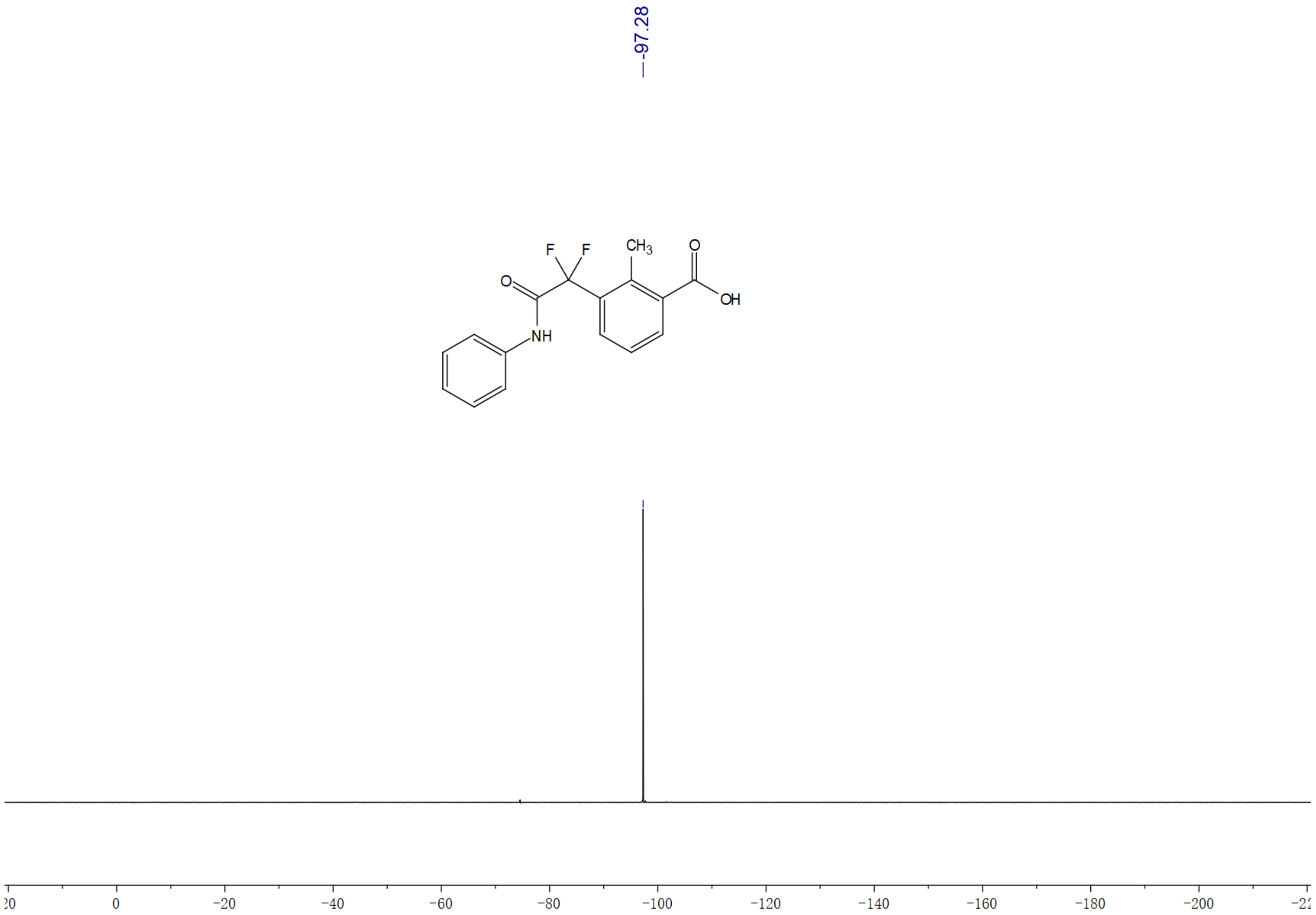

-97.28
F
F
CH3
O
OH
O
NH
20
0
-20
-40
-60
-80
-100
-120
-140
-160
-180
-200

Methyl-3-(1,1-difluoro-2-morpholino-2-oxoethyl)-2-methylbenzoate, **3lb**, ^{1}H NMR (500 MHz, $CDCl_3$), ^{13}C NMR (125 MHz, $CDCl_3$) and ^{19}F NMR (471 MHz, $CDCl_3$)

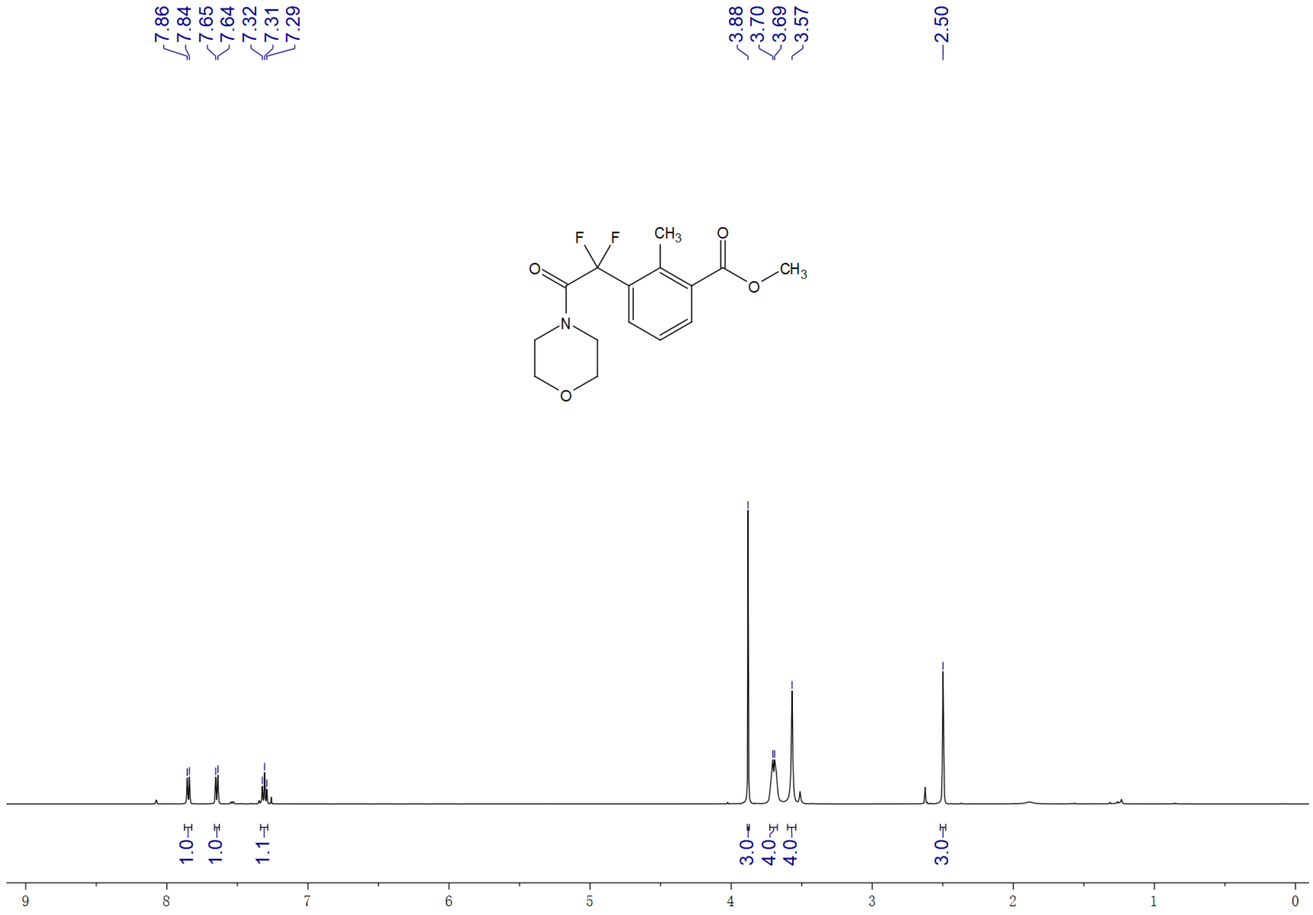

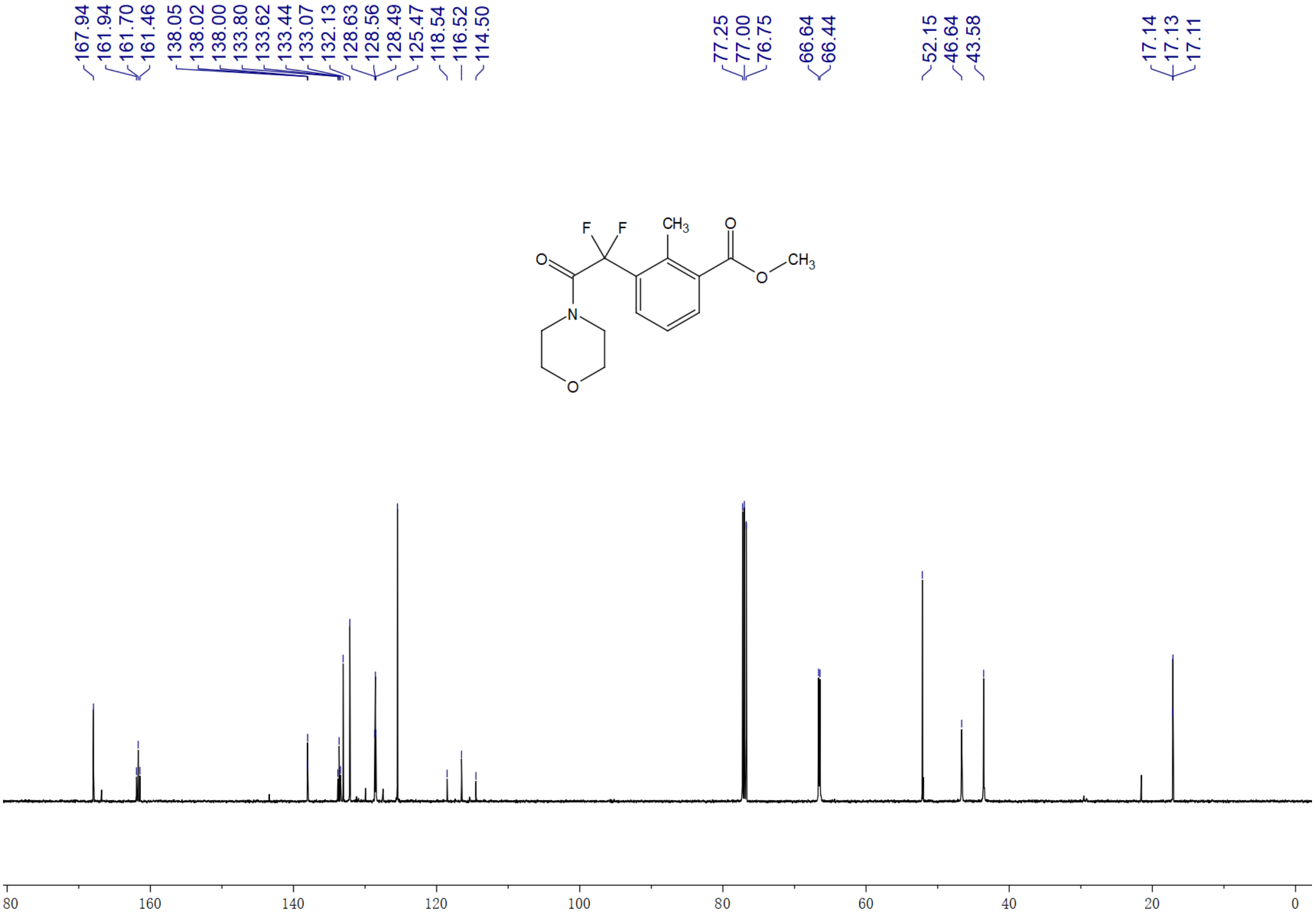

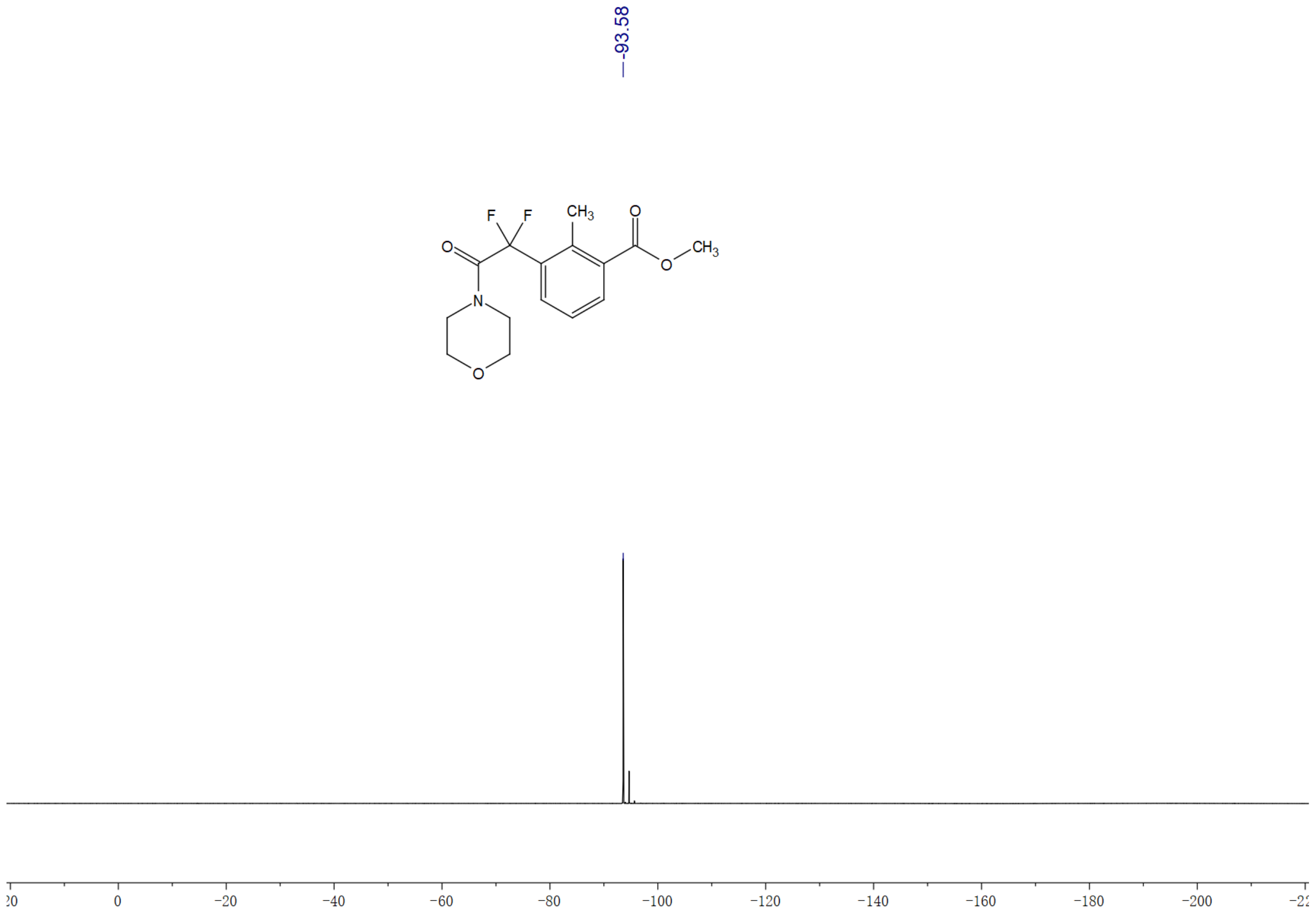
—-93.58
F
F
CH3
O
O
O
CH3
N
O
20
0
−20
−40
−60
−80
−100
−120
−140
−160
−180
−200
−220

Methyl-3-(2-(diethylamino)-1,1-difluoro-2-oxoethyl)-2-methylbenzoate, **3mb**, ^{1}H NMR (500 MHz, $CDCl_3$), ^{13}C NMR (125 MHz, $CDCl_3$) and ^{19}F NMR (471 MHz, $CDCl_3$)

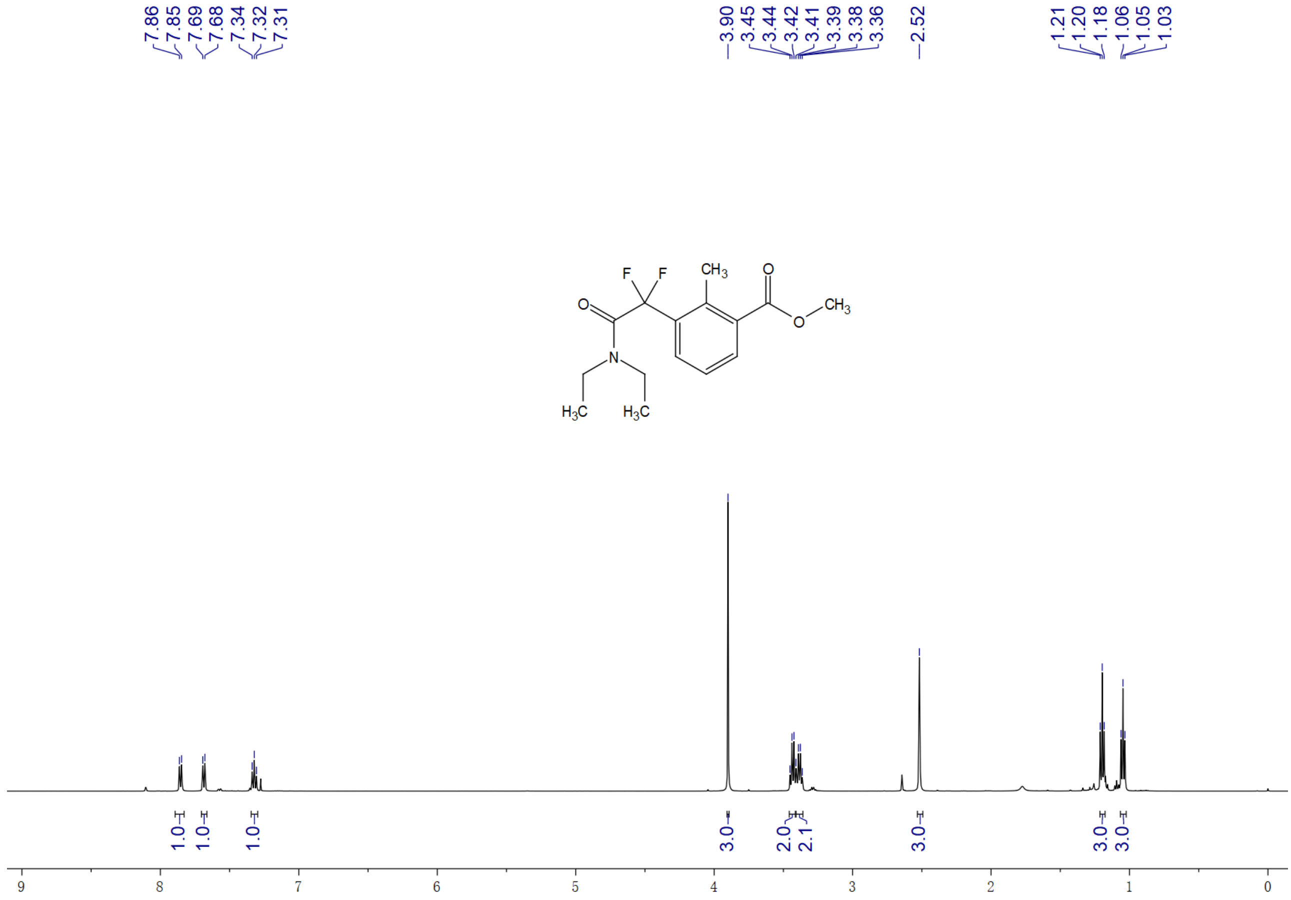

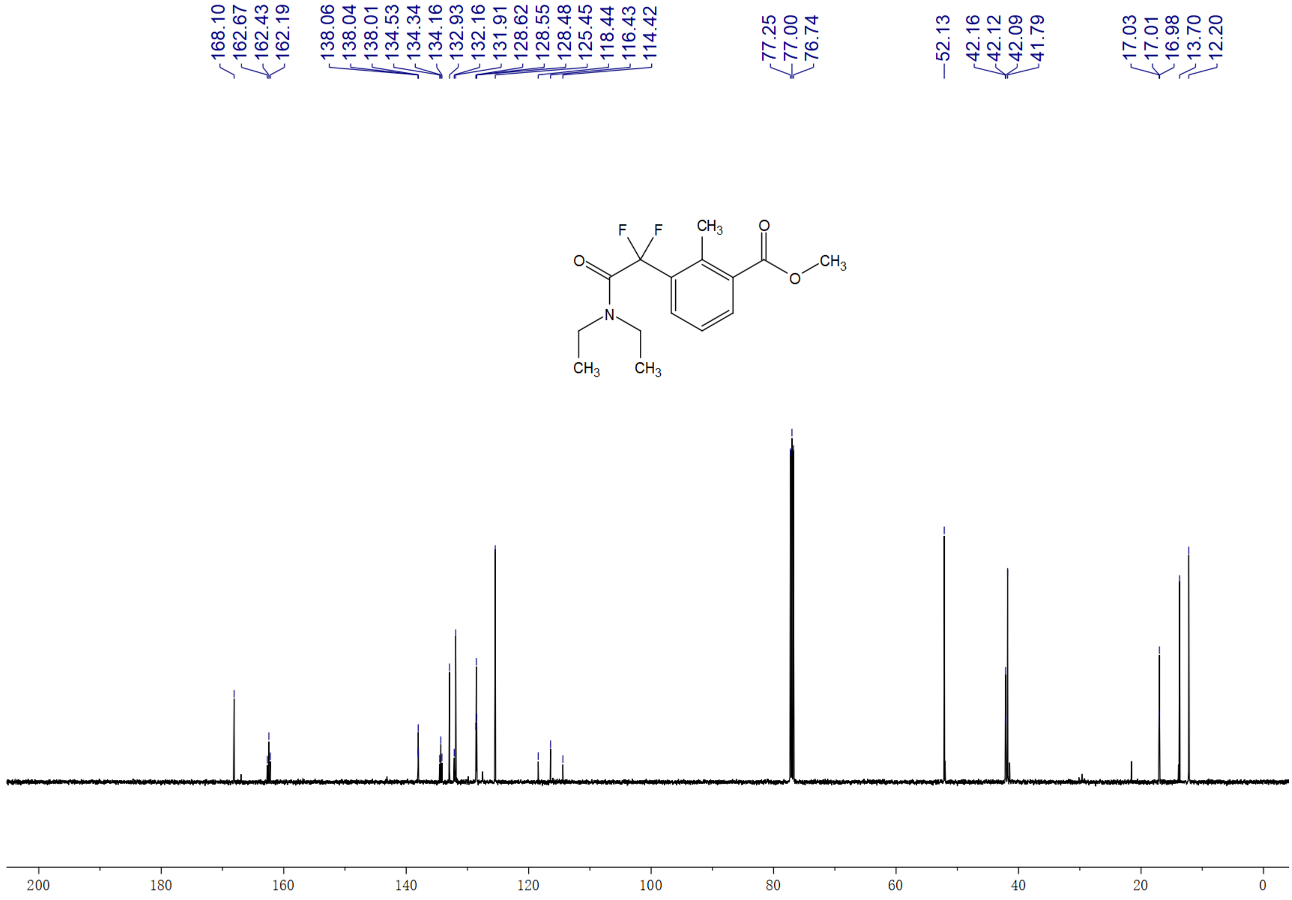

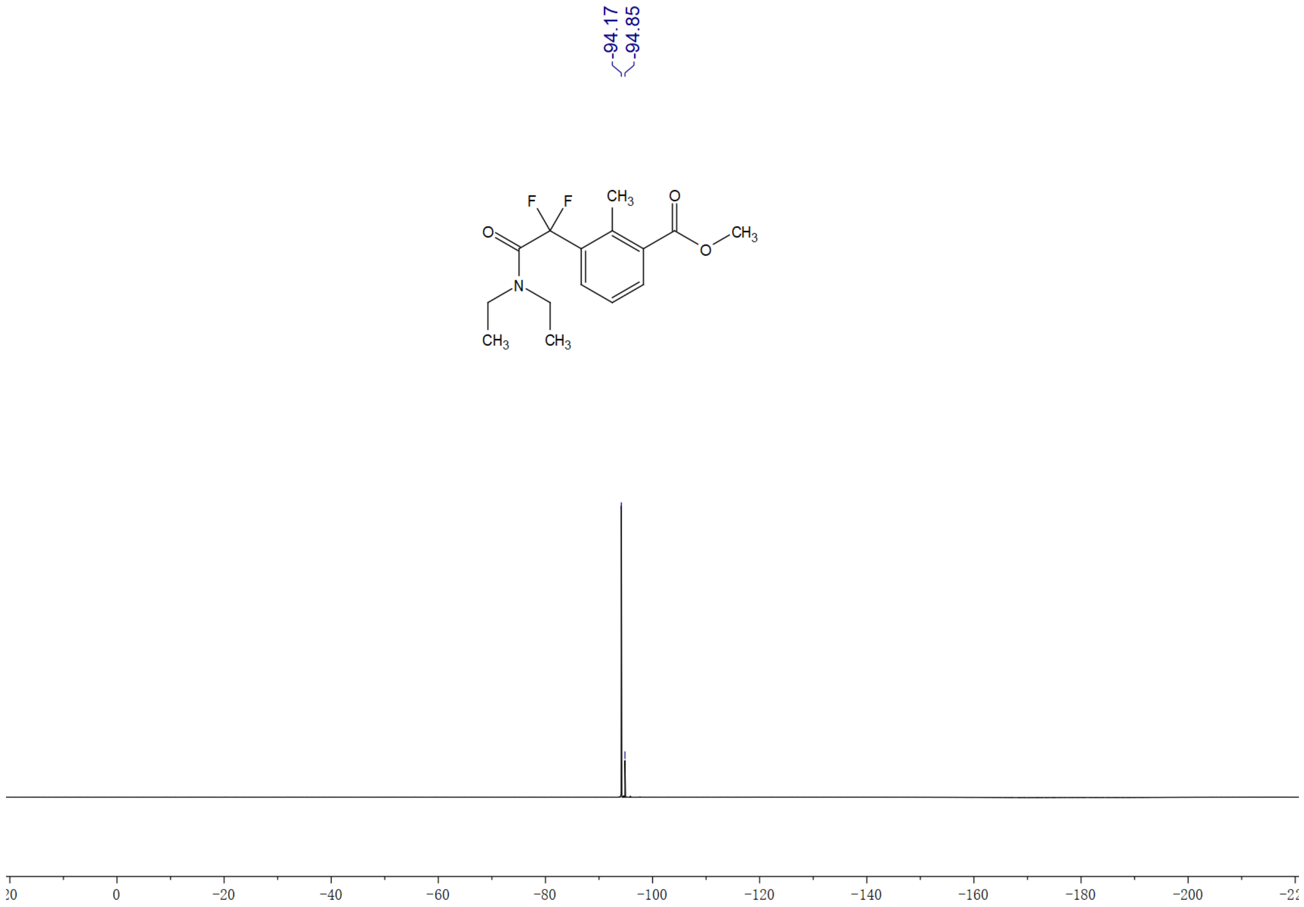

-94.17
-94.85
F
F
CH3
O
O
O
CH3
N
CH3
CH3
0
-20
-40
-60
-80
-100
-120
-140
-160
-180
-200

Methyl-3-(1-((5-isopropyl-2-methylcyclohexyl)oxy)-2-methyl-1-oxopropan-2-yl)-2-methylbenzoate, **3nb**, ^{1}H NMR (400 MHz, $CDCl_3$) and ^{13}C NMR (100 MHz, $CDCl_3$)

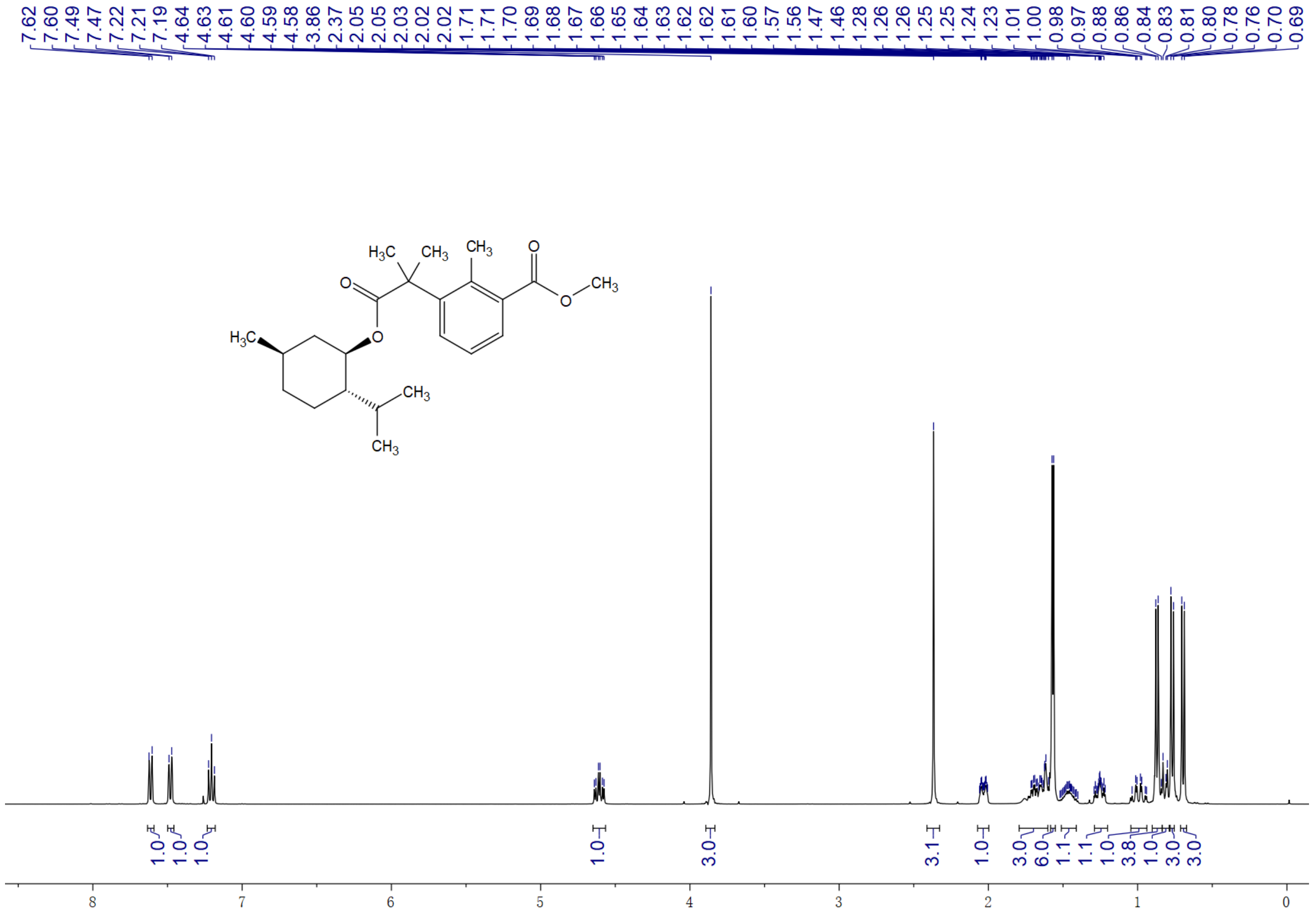

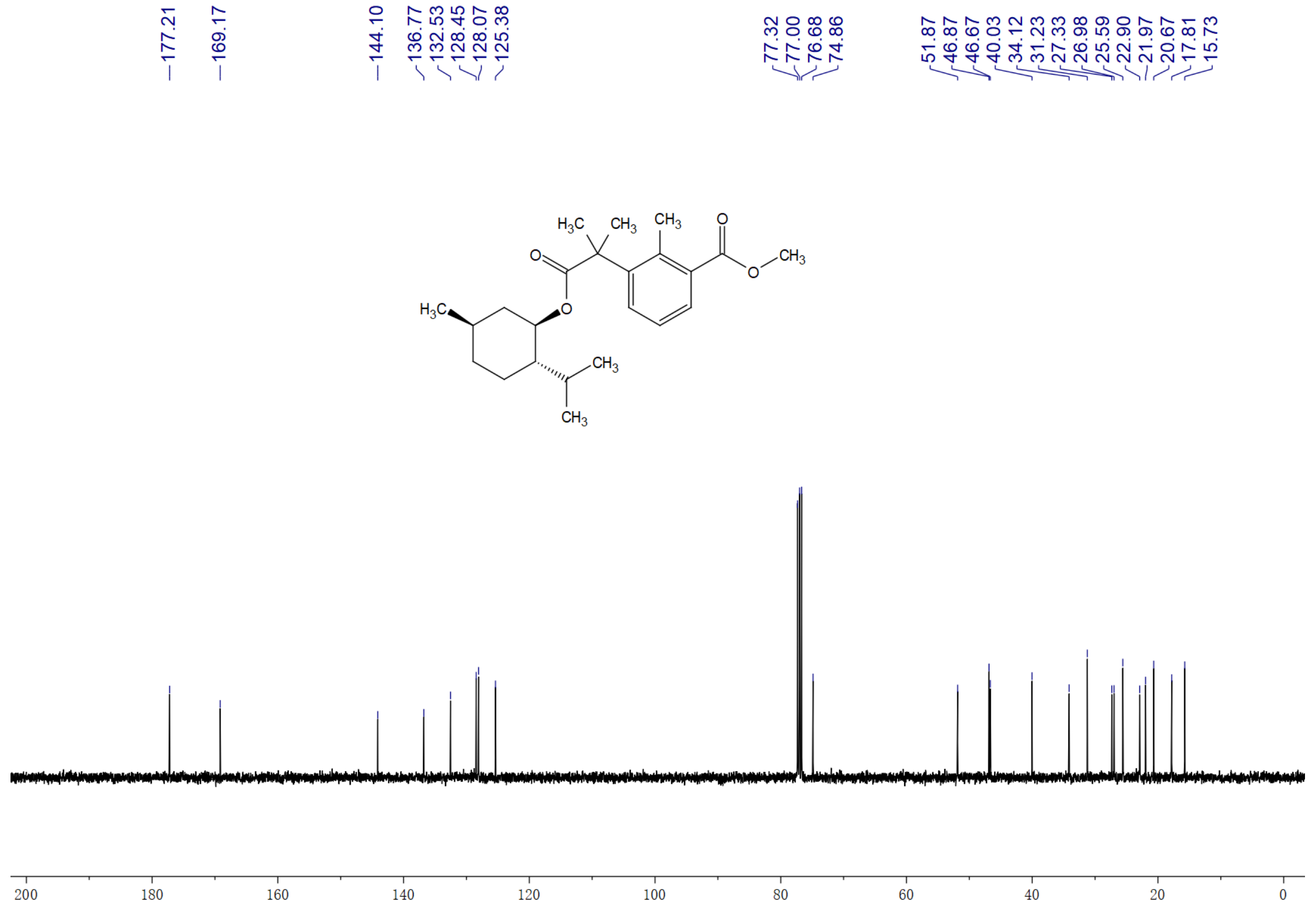

Methyl-2-methyl-3-(2-methyl-1-oxo-1-(((3aS,5S,5aR,8aR,8bS)-2,2,7,7-tetramethyltetrahydro-5H-bis([1,3]dioxolo)[4,5-b:4',5'-d]pyran-5-yl)methoxy)propan-2-yl)benzoate, **3ob**, ^{1}H NMR (400 MHz, $CDCl_3$) and ^{13}C NMR (100 MHz, $CDCl_3$)

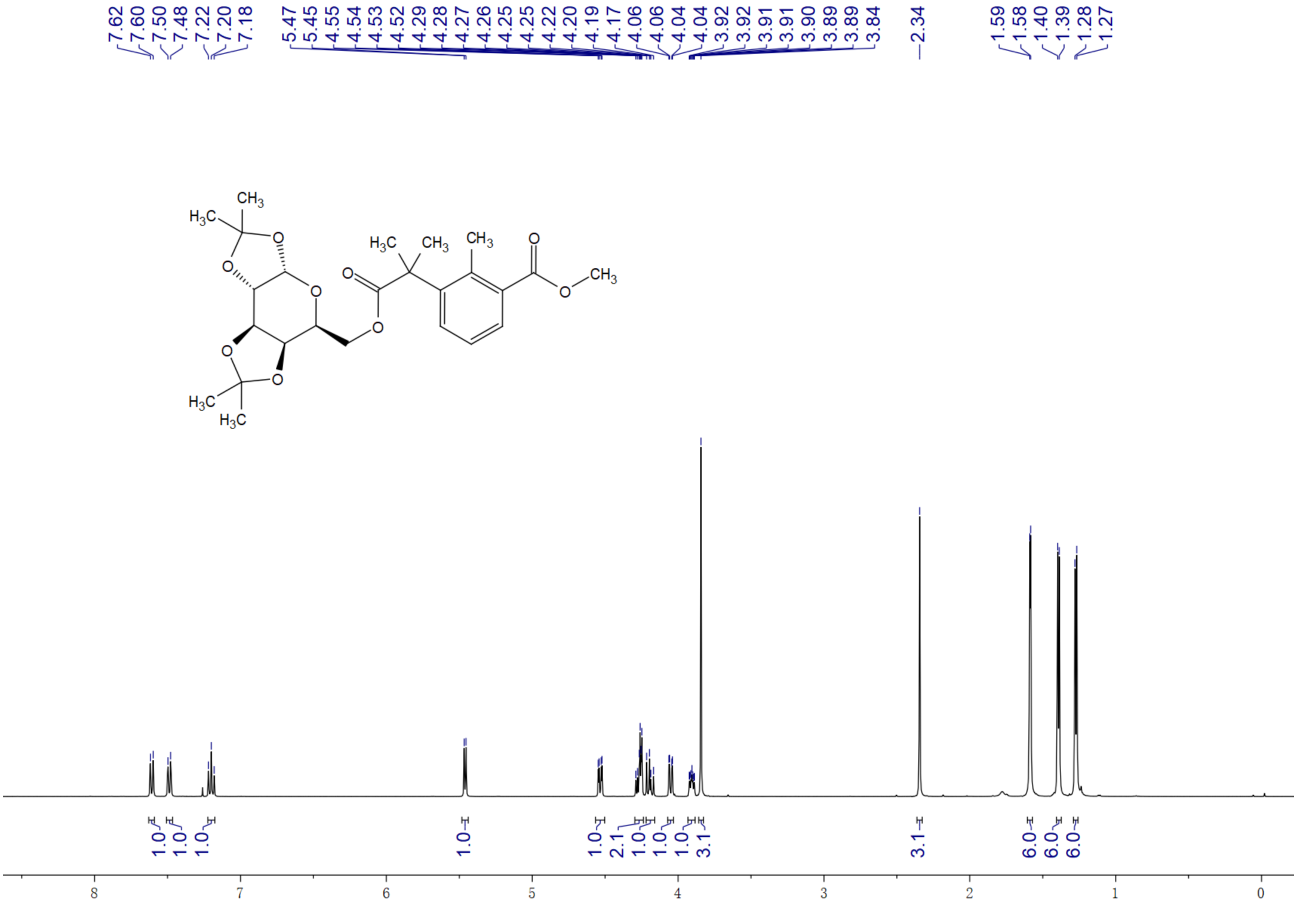

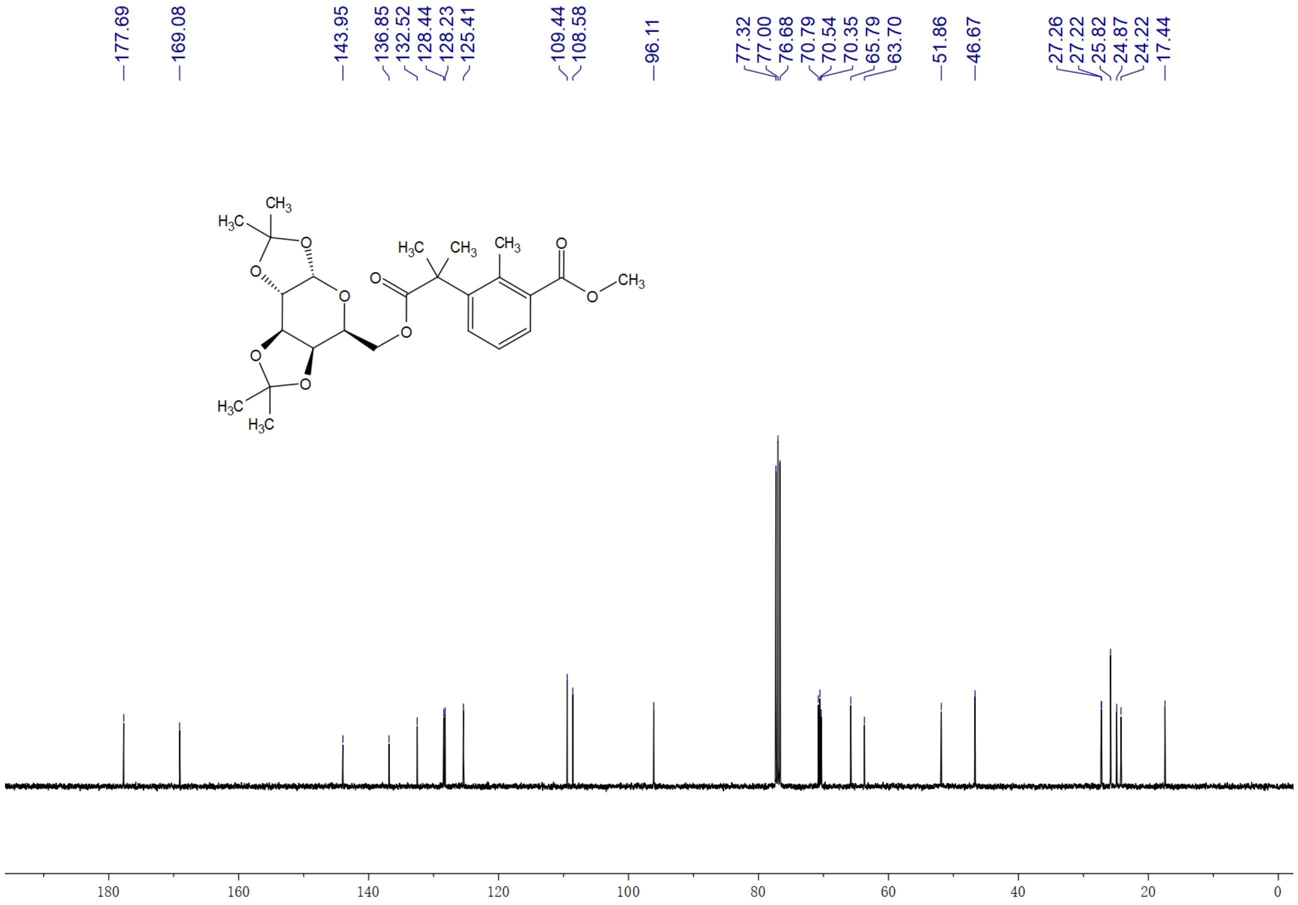

Methyl-2-methyl-3-(2-methyl-1-oxo-1-(((1S,2R,4S)-1,7,7-trimethylbicyclo[2.2.1]heptan-2-yl)oxy)propan-2-yl)benzoate, **3pb**, ^{1}H NMR (400 MHz, $CDCl_3$) and ^{13}C NMR (100 MHz, $CDCl_3$)

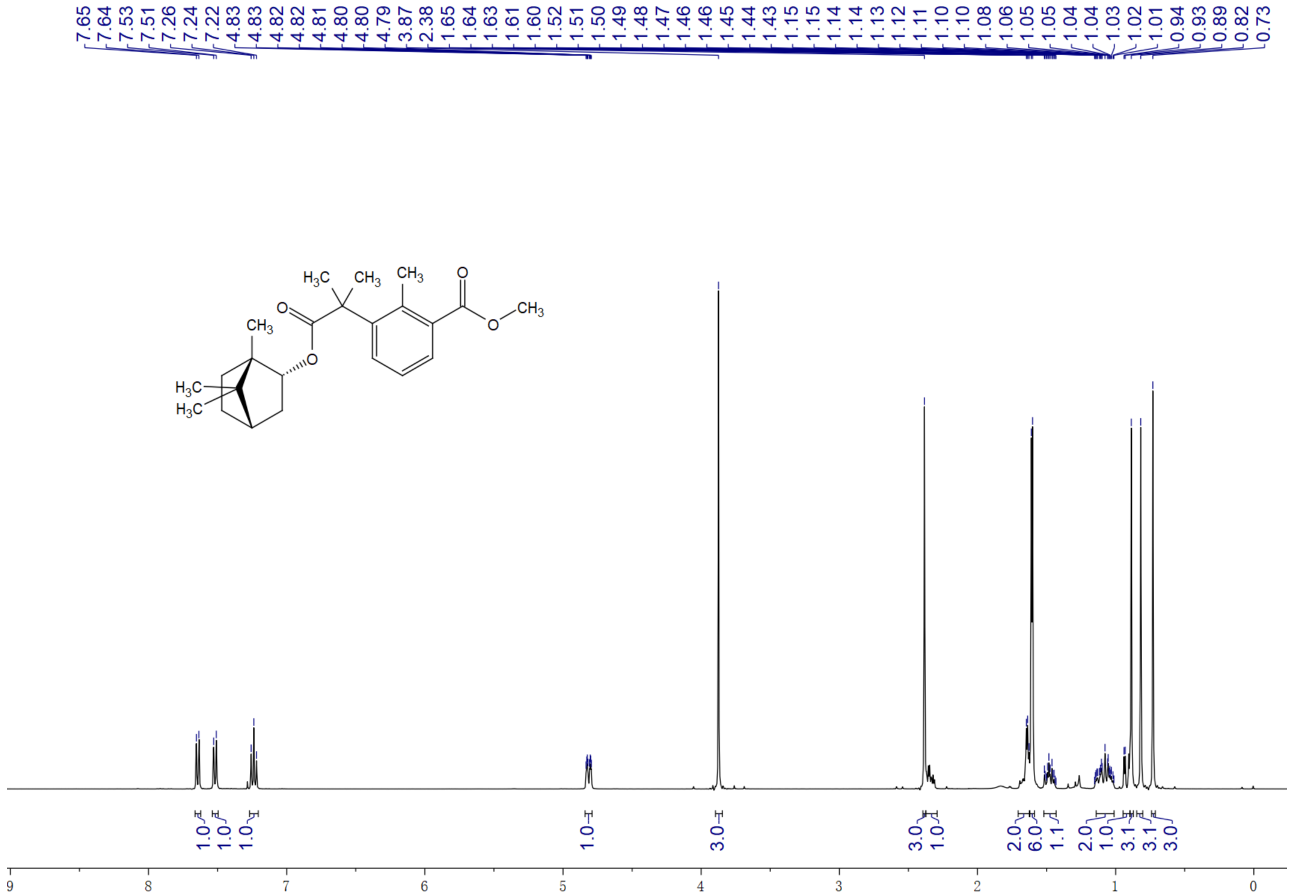

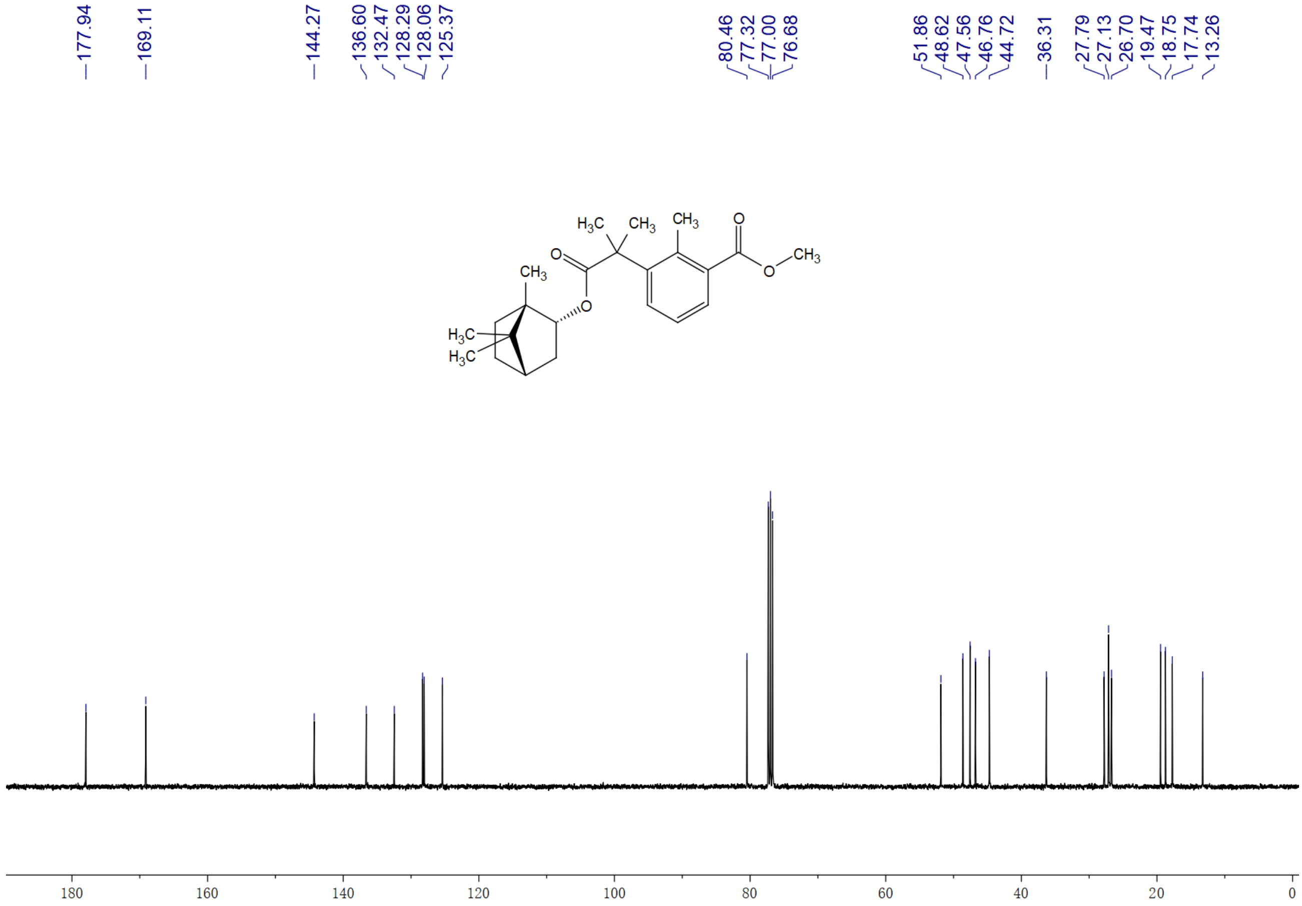

2-Methyl-3-(2-methyl-1-((14-methyl-15-oxo-7,8,9,11,12,13,14,15,16,17-decahydro-6H-cyclopenta[a]phenanthren-3-yl)oxy)-1-oxopropan-2-yl)benzoic acid, **3qb**, ^{1}H NMR (400 MHz, DMSO-d_6) and ^{13}C NMR (100 MHz, DMSO-d_6)

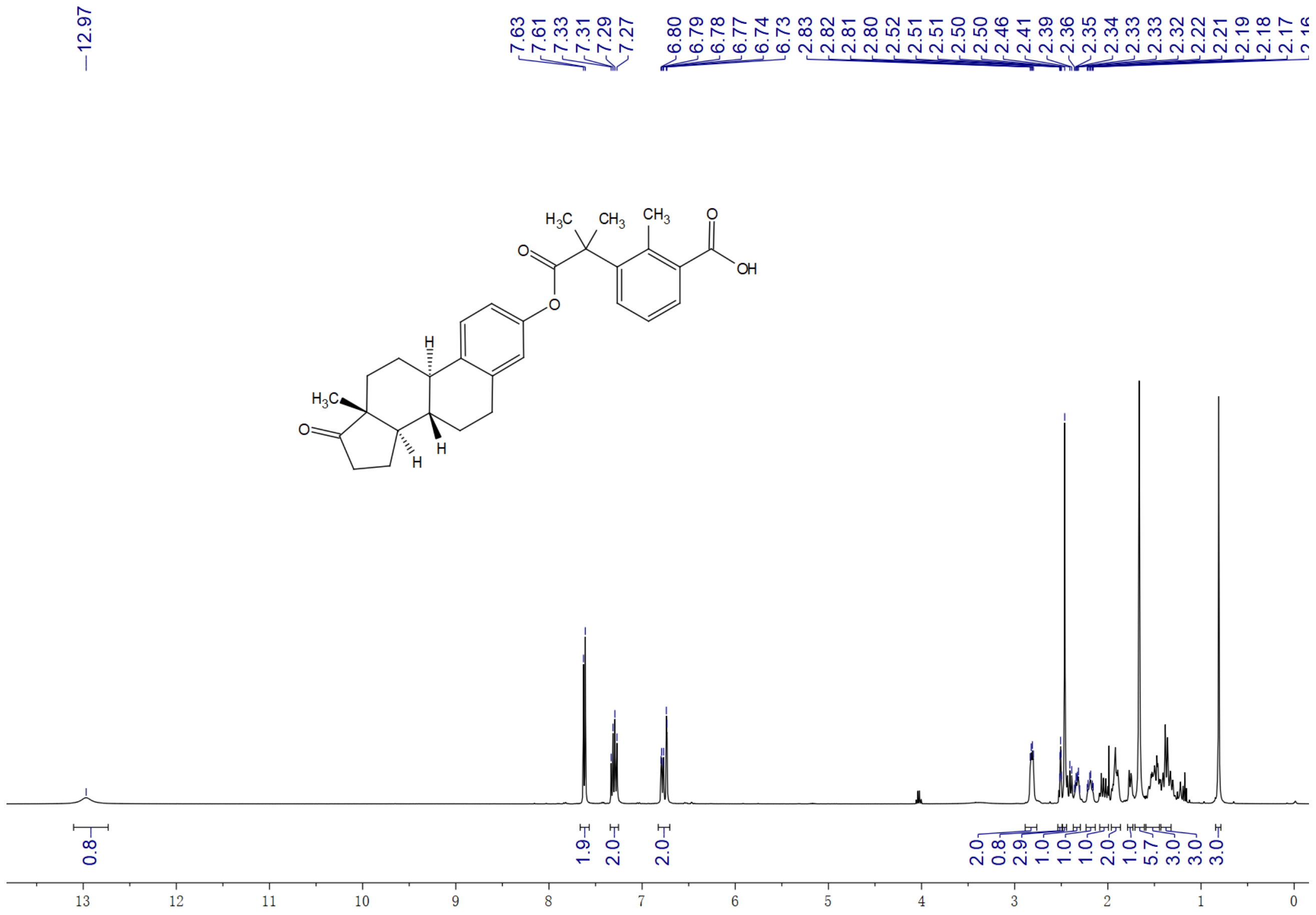

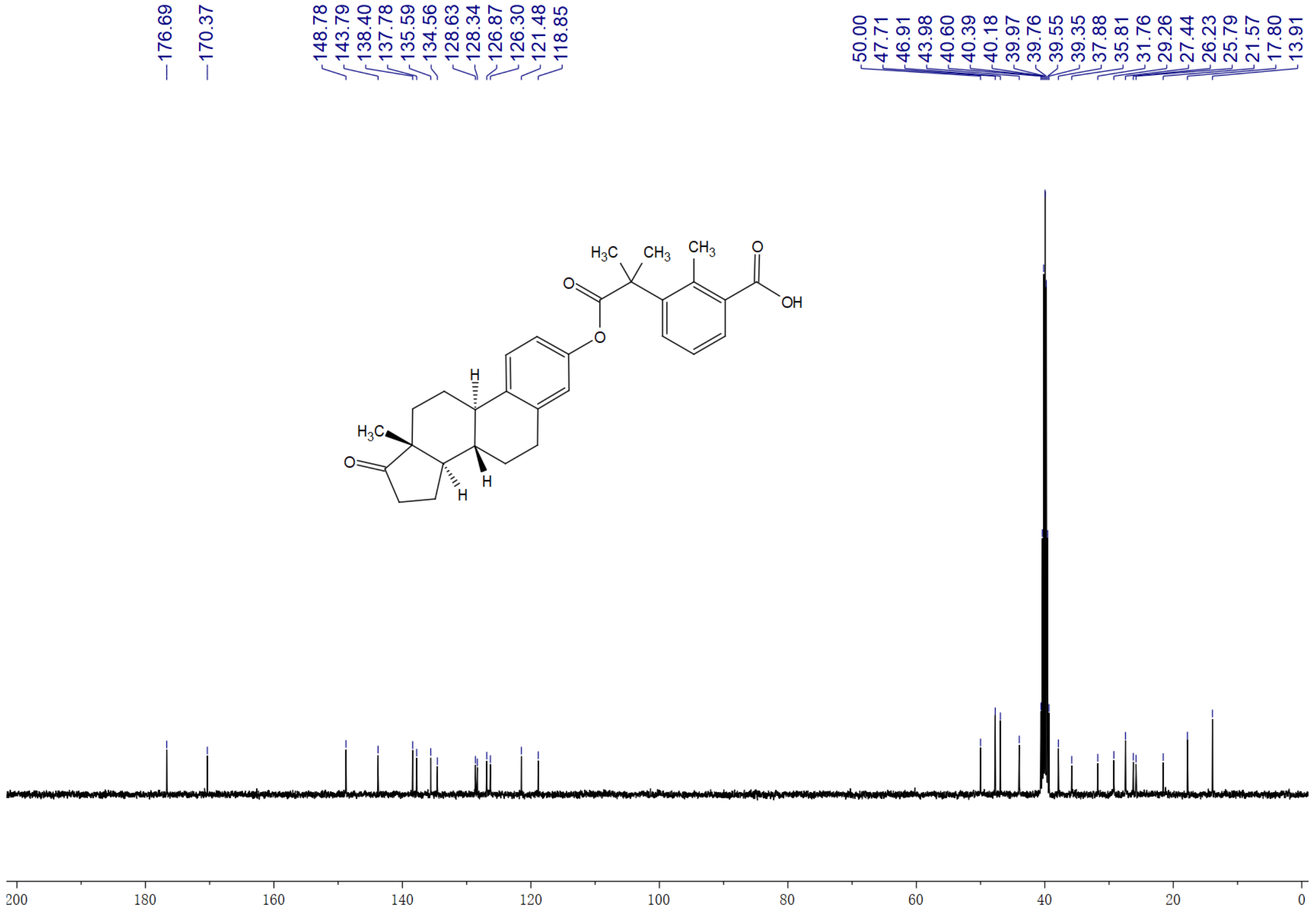

Methyl-3-(1-((1-(2,6-dimethylphenoxy)propan-2-yl)amino)-2-methyl-1-oxopropan-2-yl)-2-methylbenzoate, **3rb**, ^{1}H NMR (500 MHz, CDCl$_3$) and ^{13}C NMR (125 MHz, CDCl$_3$)

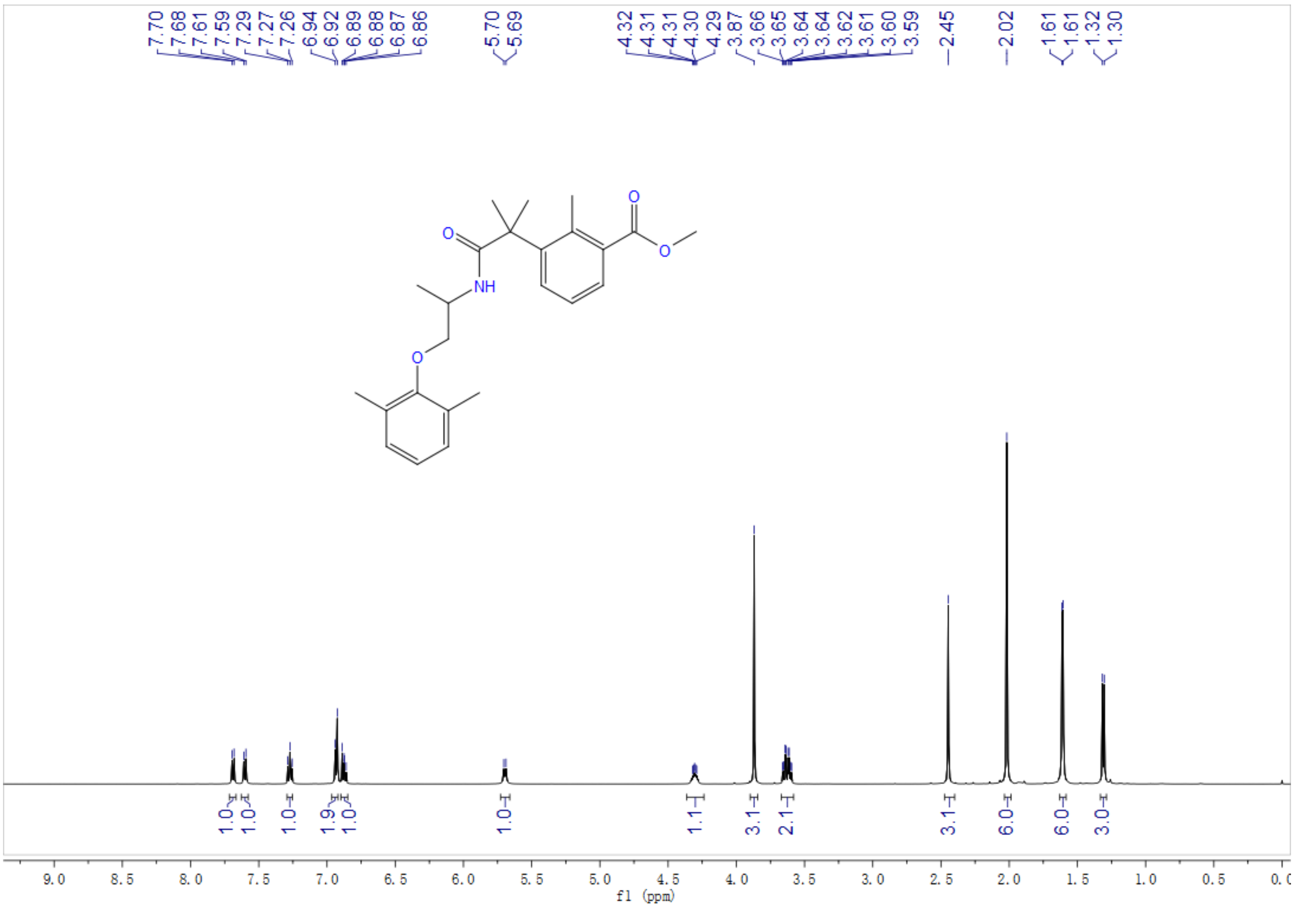

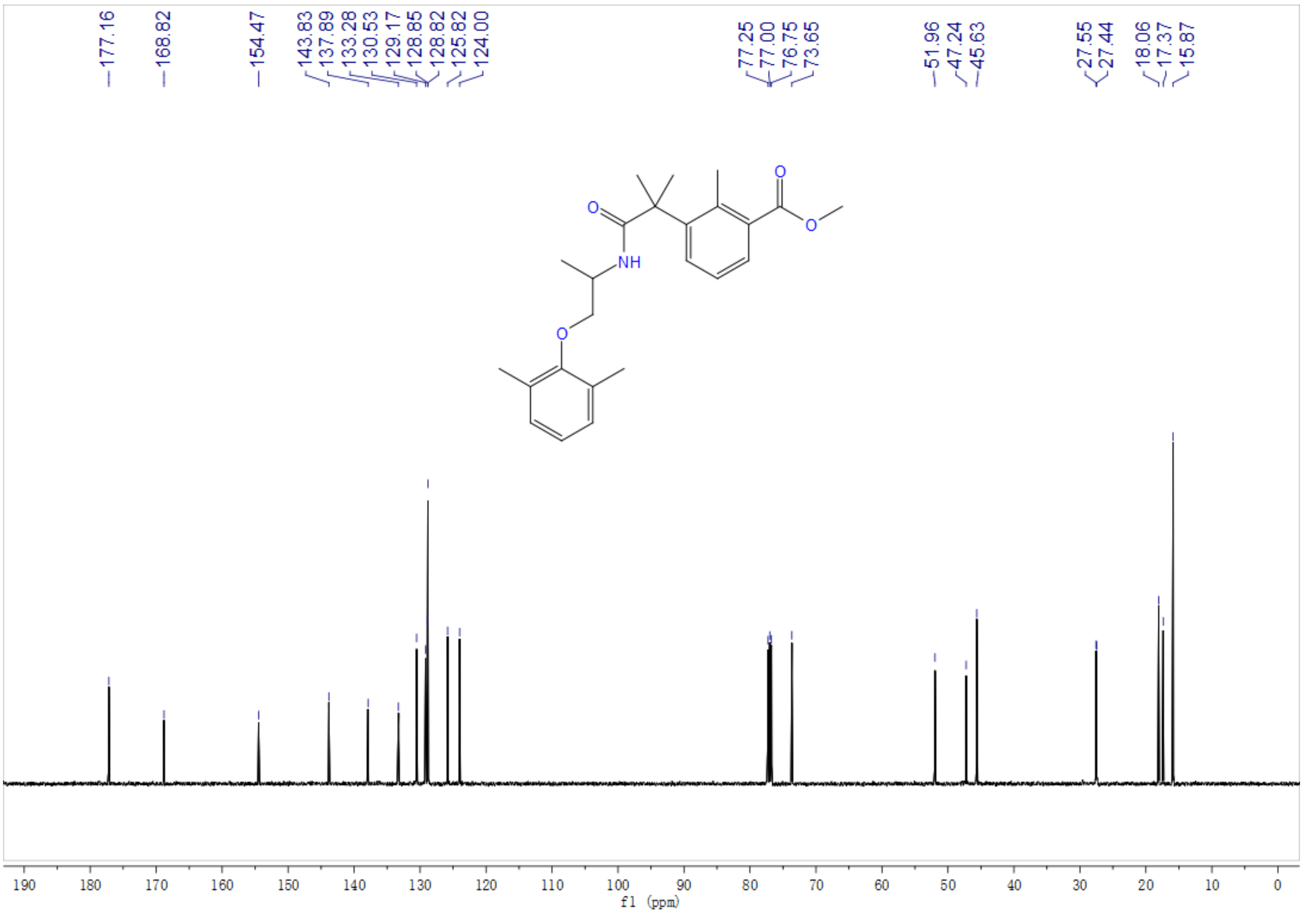

Methyl-3-(1-((4-(3-ethyl-2,6-dioxopiperidin-3-yl)phenyl)amino)-2-methyl-1-oxopropan-2-yl)-2 methylbenzoate, **3sb**, ^{1}H NMR (500 MHz, $CDCl_3$) and ^{13}C NMR (125 MHz, $CDCl_3$)

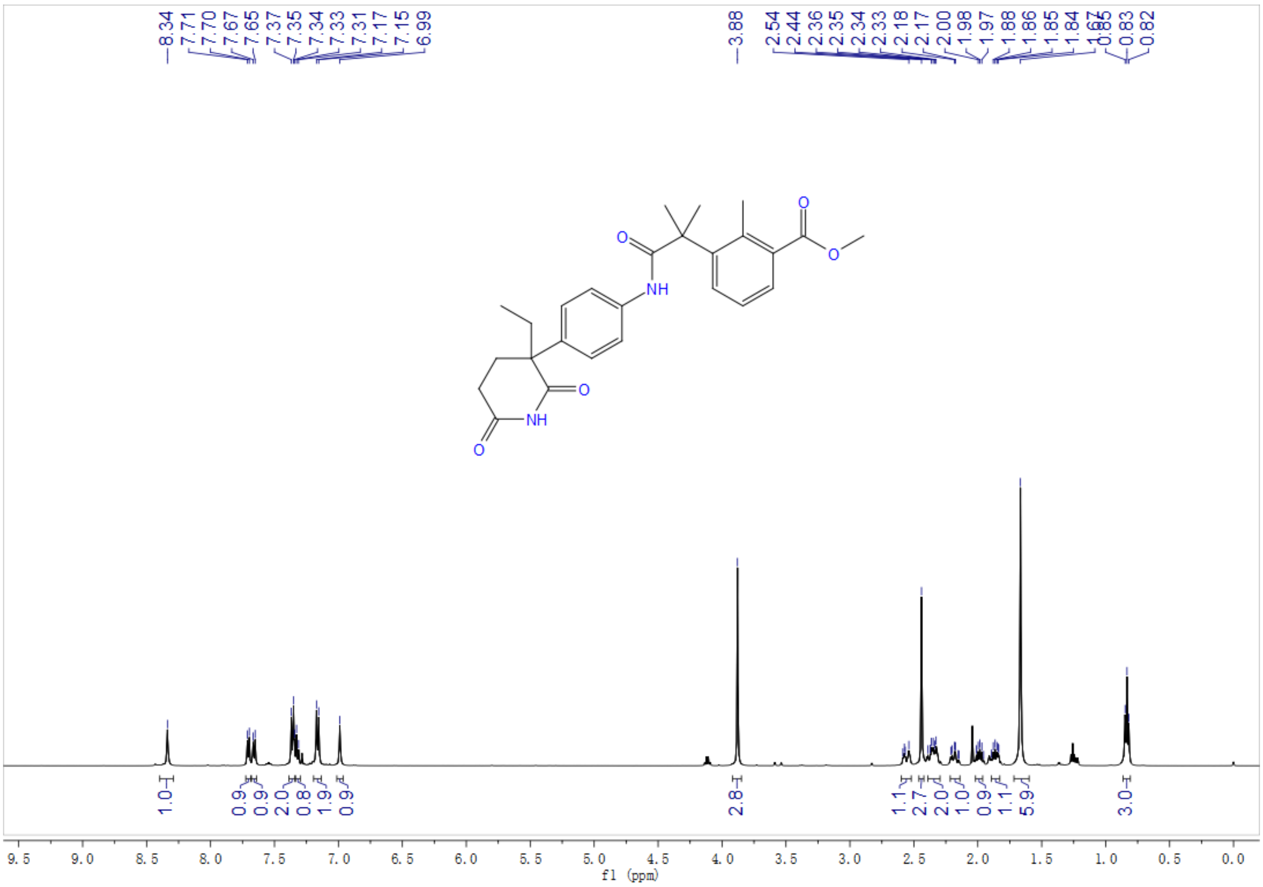

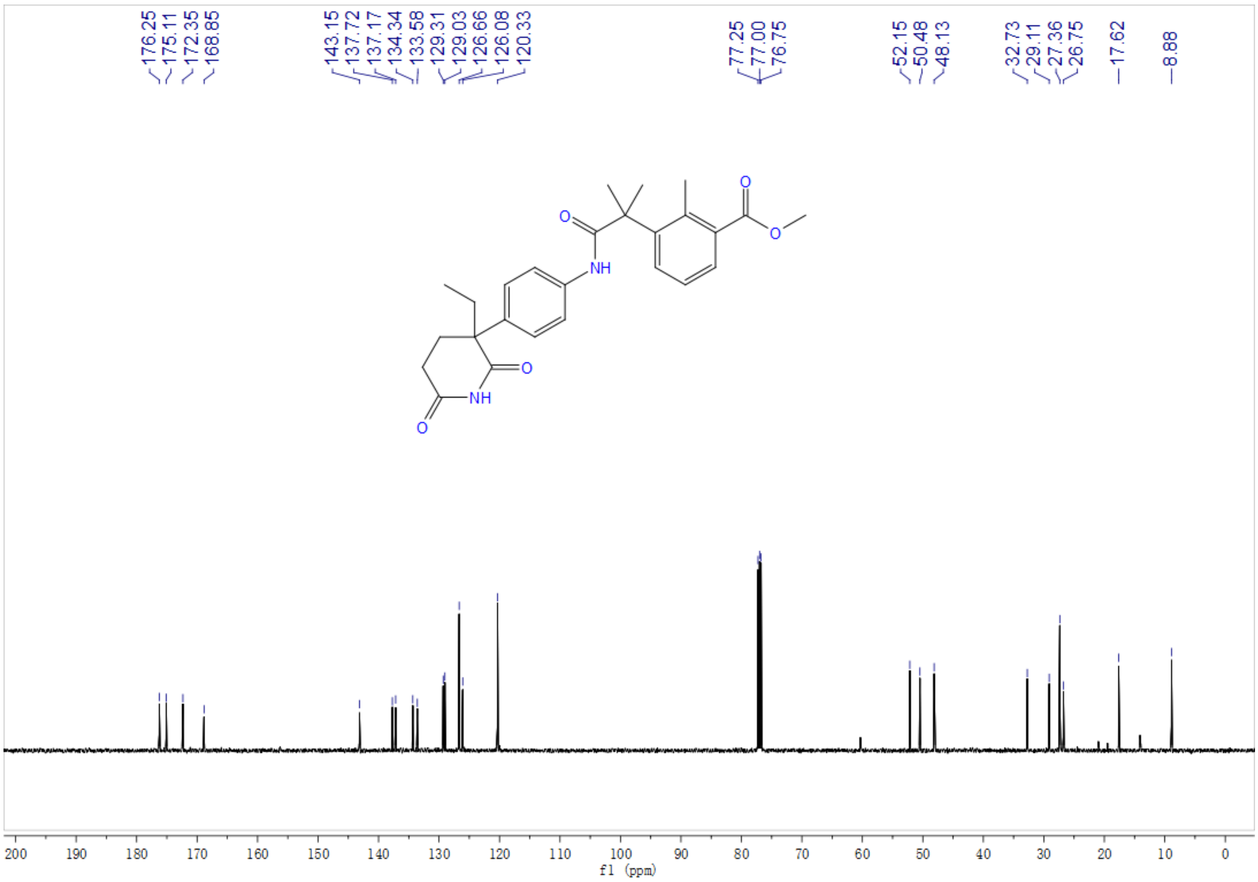

5-(2-Methyl-1-oxo-1-(phenylamino)propan-2-yl)-1-naphthoic acid, **4aa**, ^{1}H NMR (400 MHz, DMSO-d_6) and ^{13}C NMR (100 MHz, DMSO-d_6)

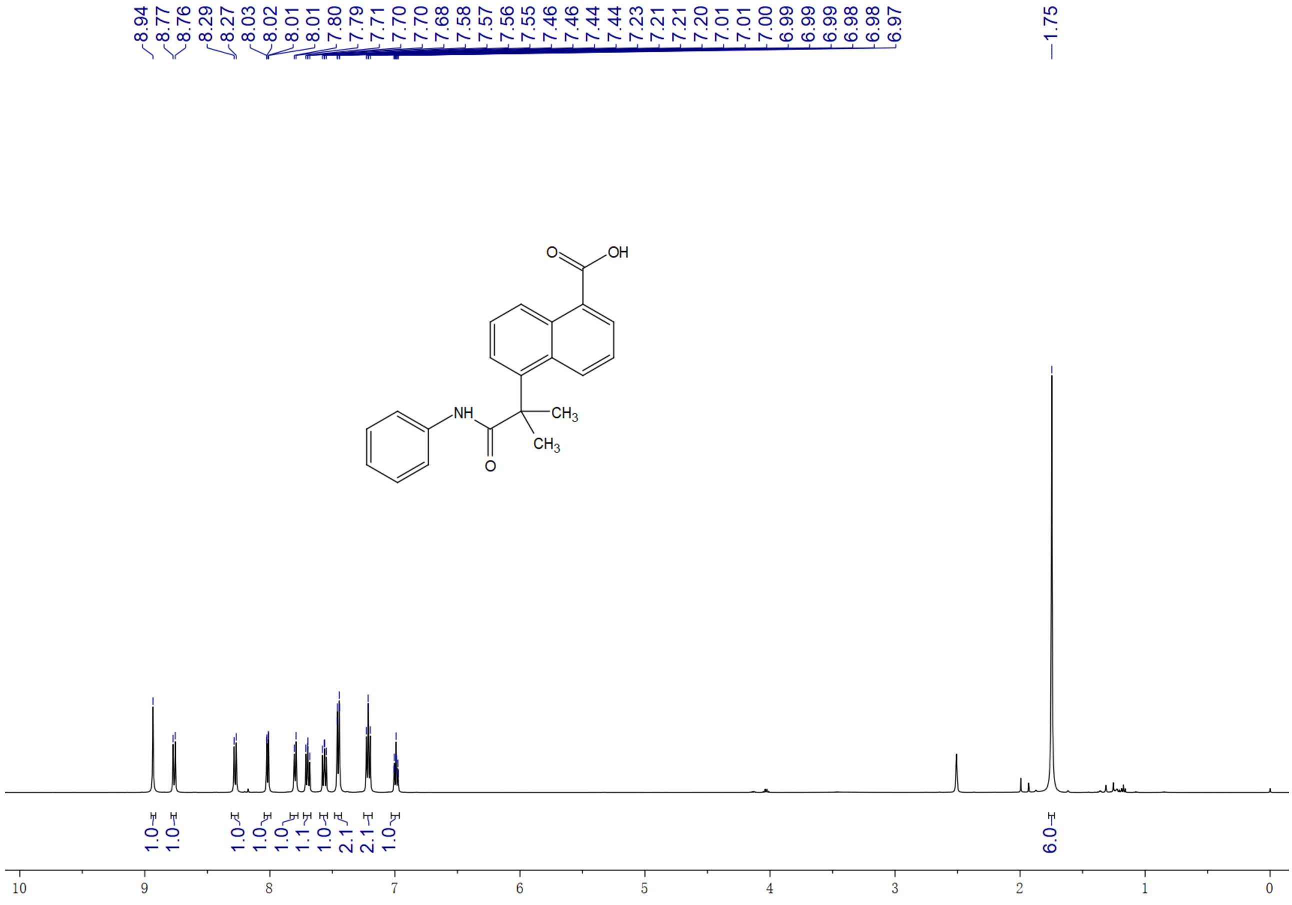

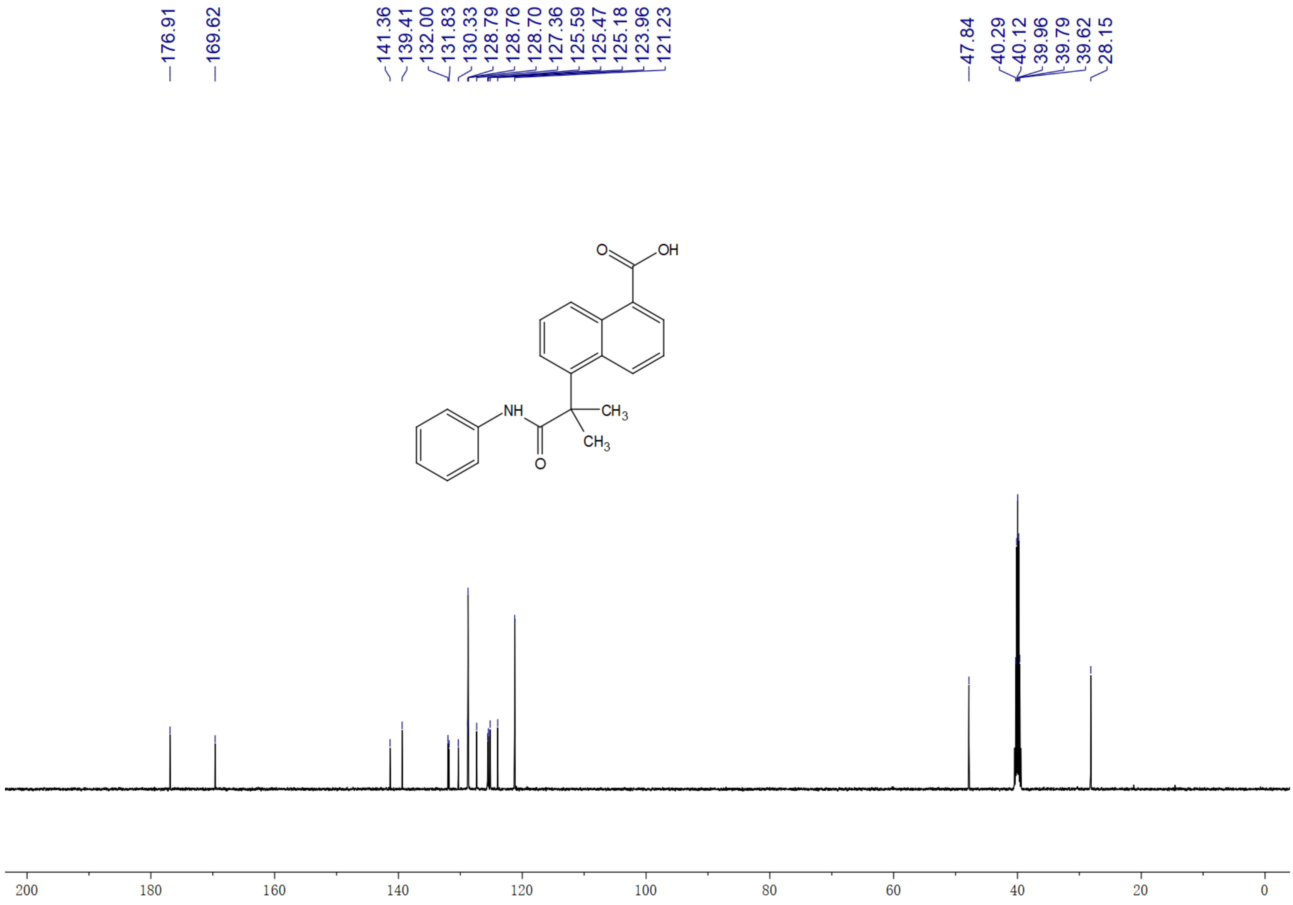

5-(2-Methyl-1-oxo-1-((4-(trifluoromethyl)phenyl)amino)propan-2-yl)-1-naphthoic acid, **4ba**, ^{1}H NMR (500 MHz, DMSO-d_6) and ^{13}C NMR (125 MHz, DMSO-d_6) and ^{19}F NMR (471 MHz, DMSO)

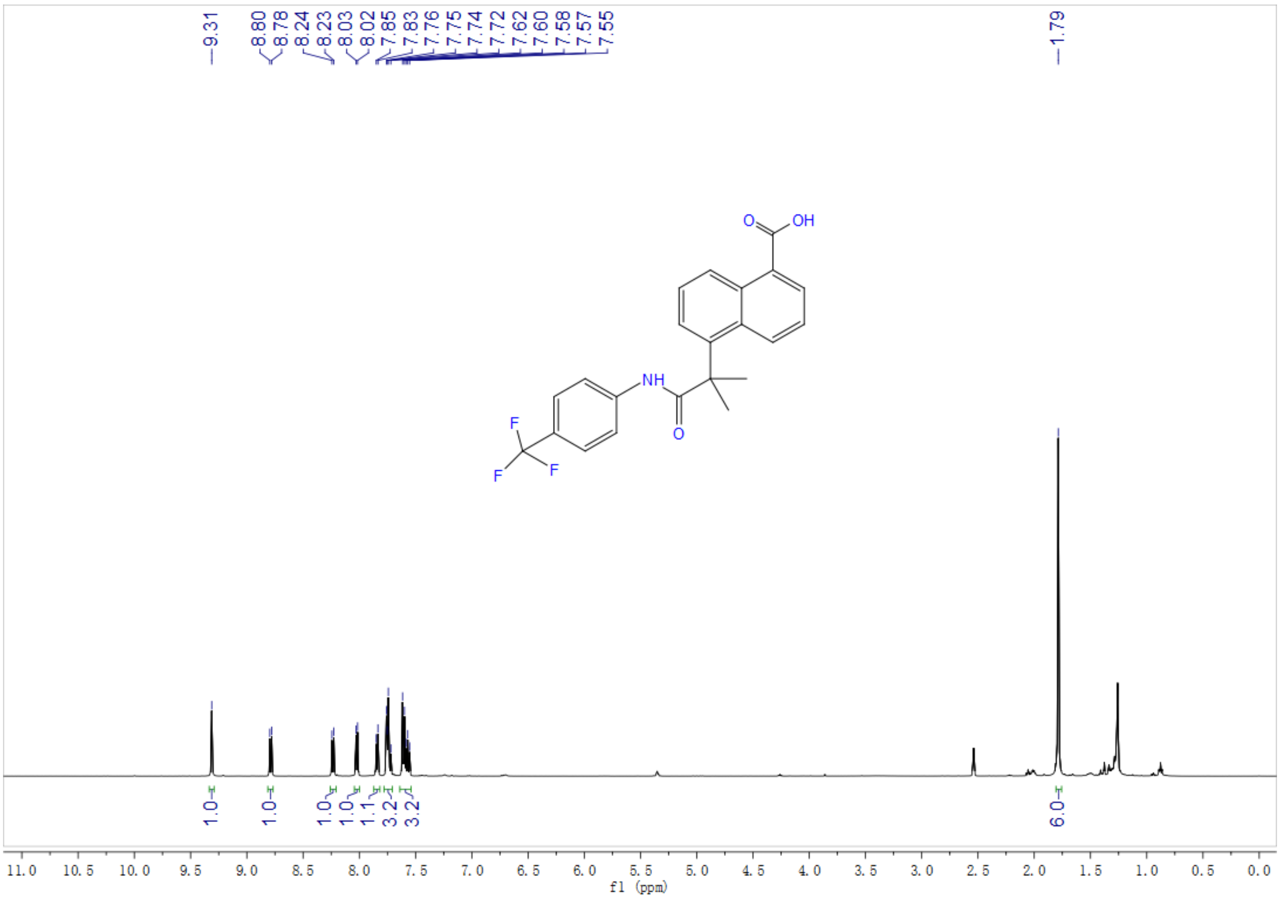

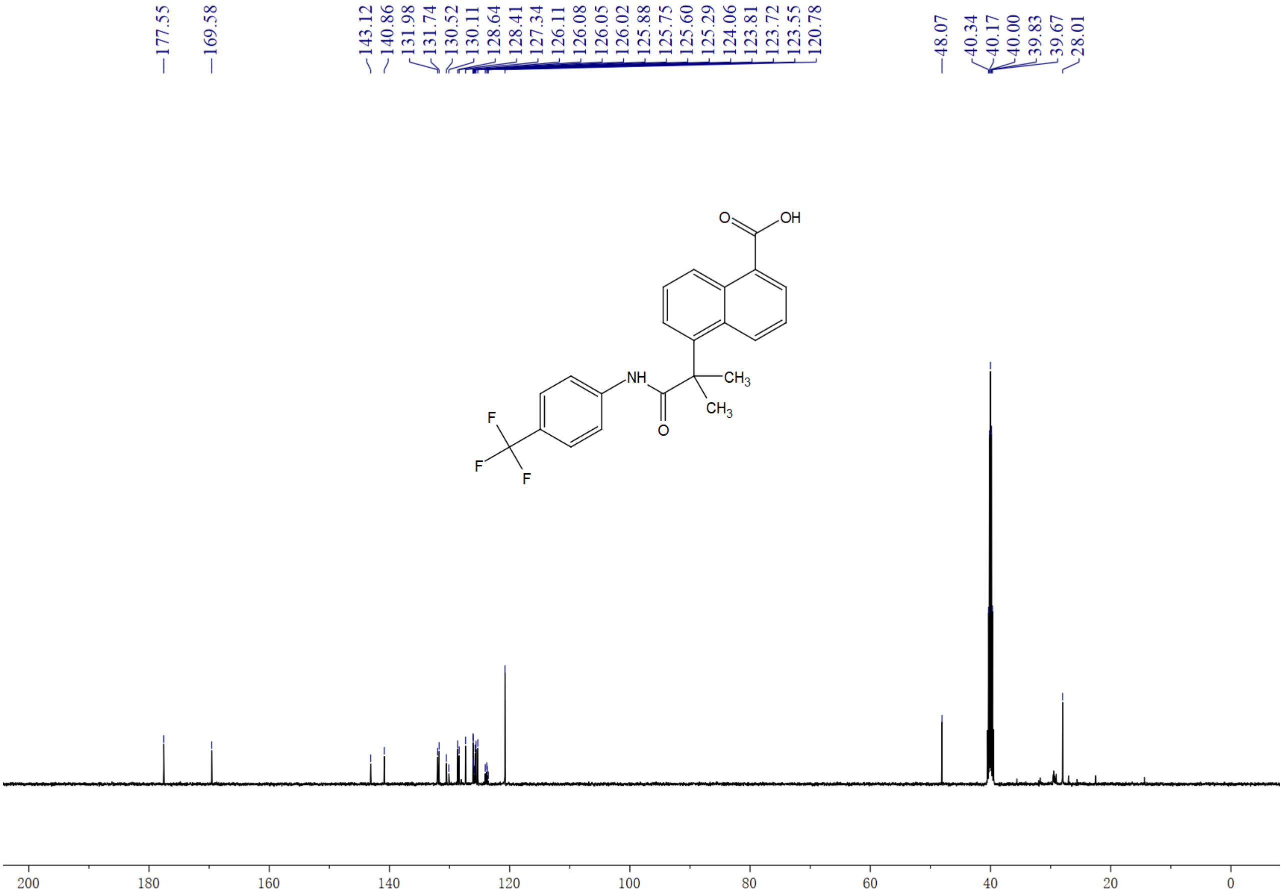

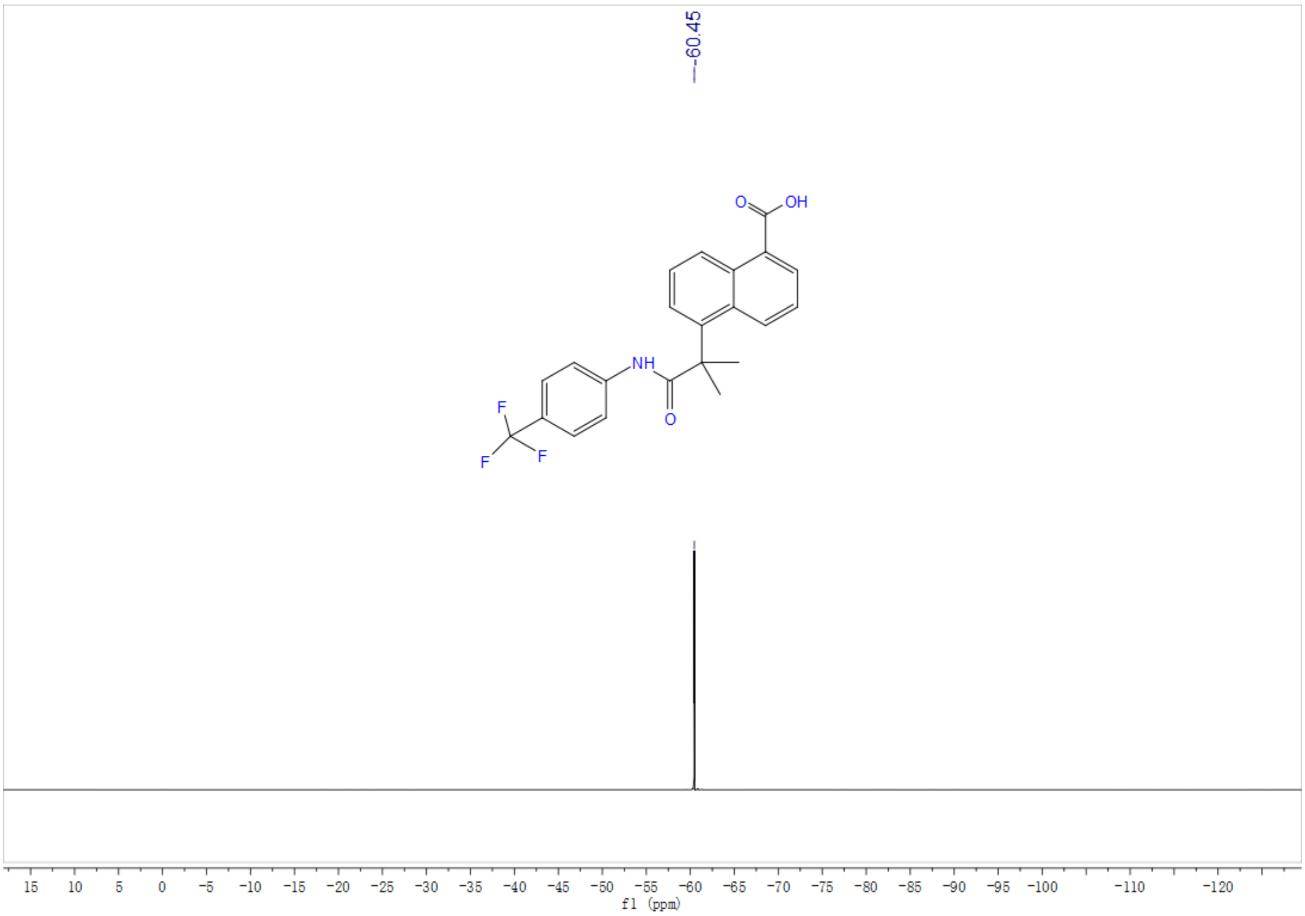

-60.45
O
OH
NH
O
F
F
F
15
10
5
0
-5
-10
-15
-20
-25
-30
-35
-40
-45
-50
-55
-60
-65
-70
-75
-80
-85
-90
-95
-100
-110
-120
f1 (ppm)

Methyl-5-(1-((4-methoxyphenyl)amino)-2-methyl-1-oxopropan-2-yl)-1-naphthoate, **4ca**, ^{1}H NMR (400 MHz, $CDCl_3$) and ^{13}C NMR (125 MHz, $CDCl_3$)

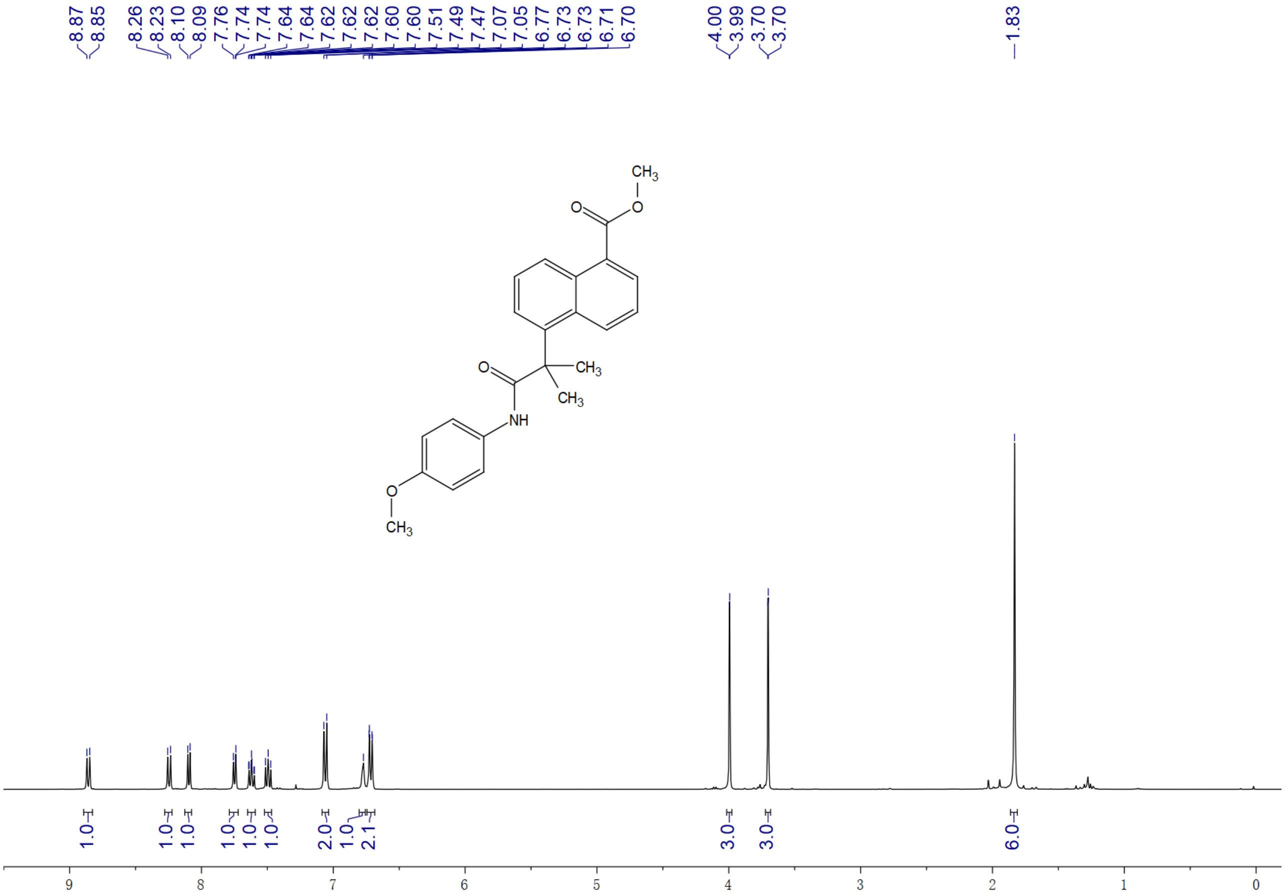

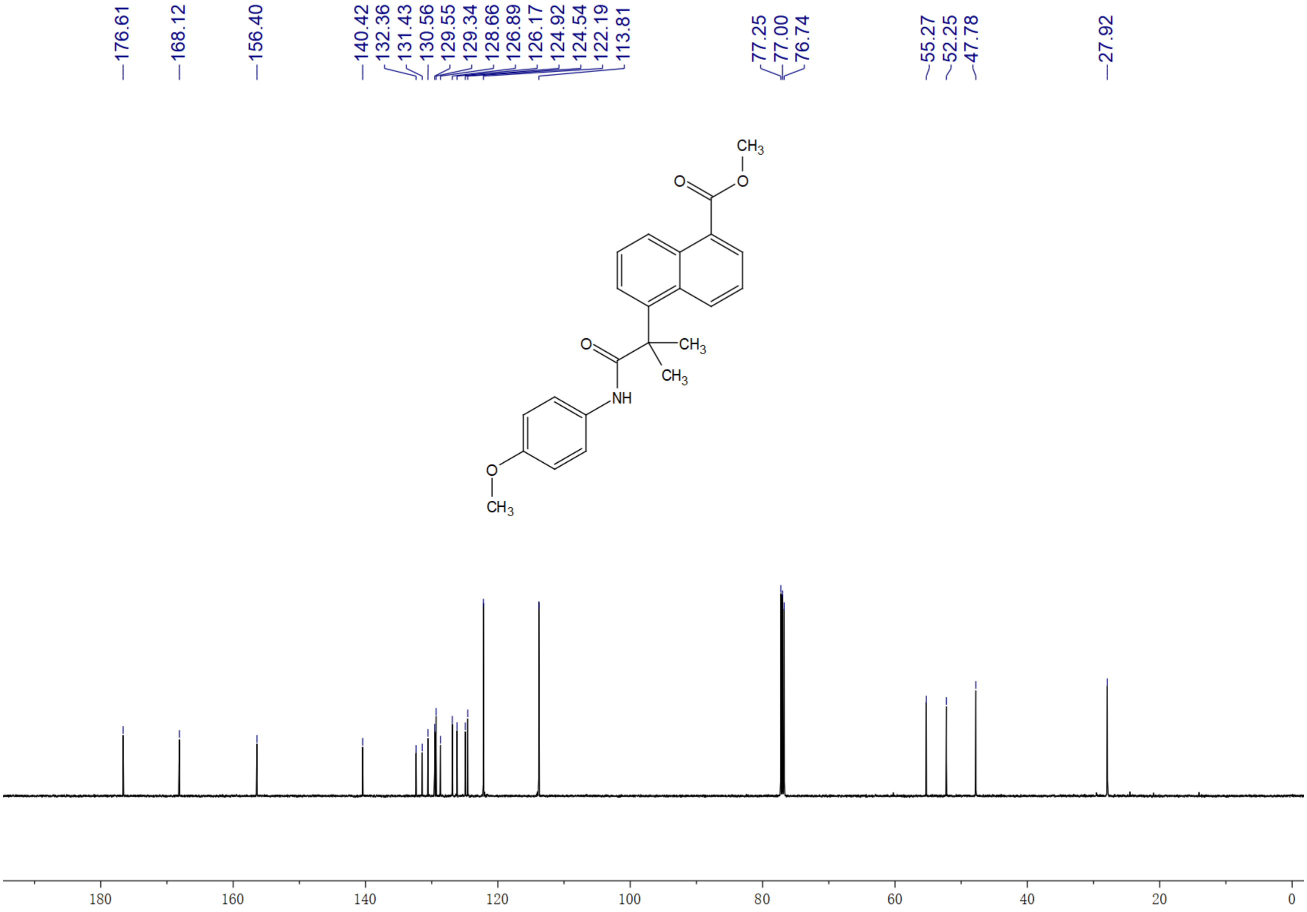

Methyl-5-(1-(butylamino)-2-methyl-1-oxopropan-2-yl)-1-naphthoate, **4da**, ^{1}H NMR (400 MHz, $CDCl_3$) and ^{13}C NMR (100 MHz, $CDCl_3$)

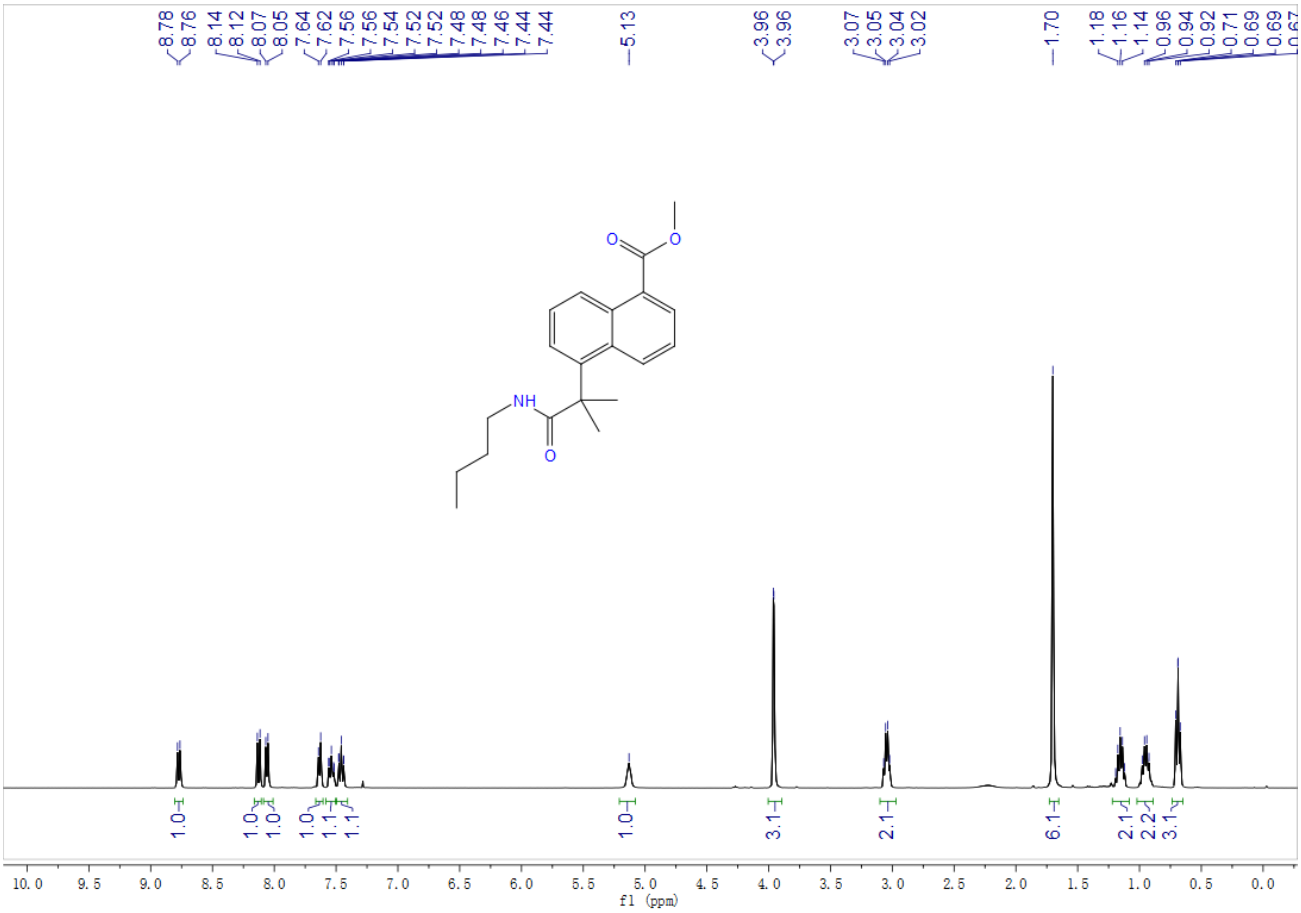

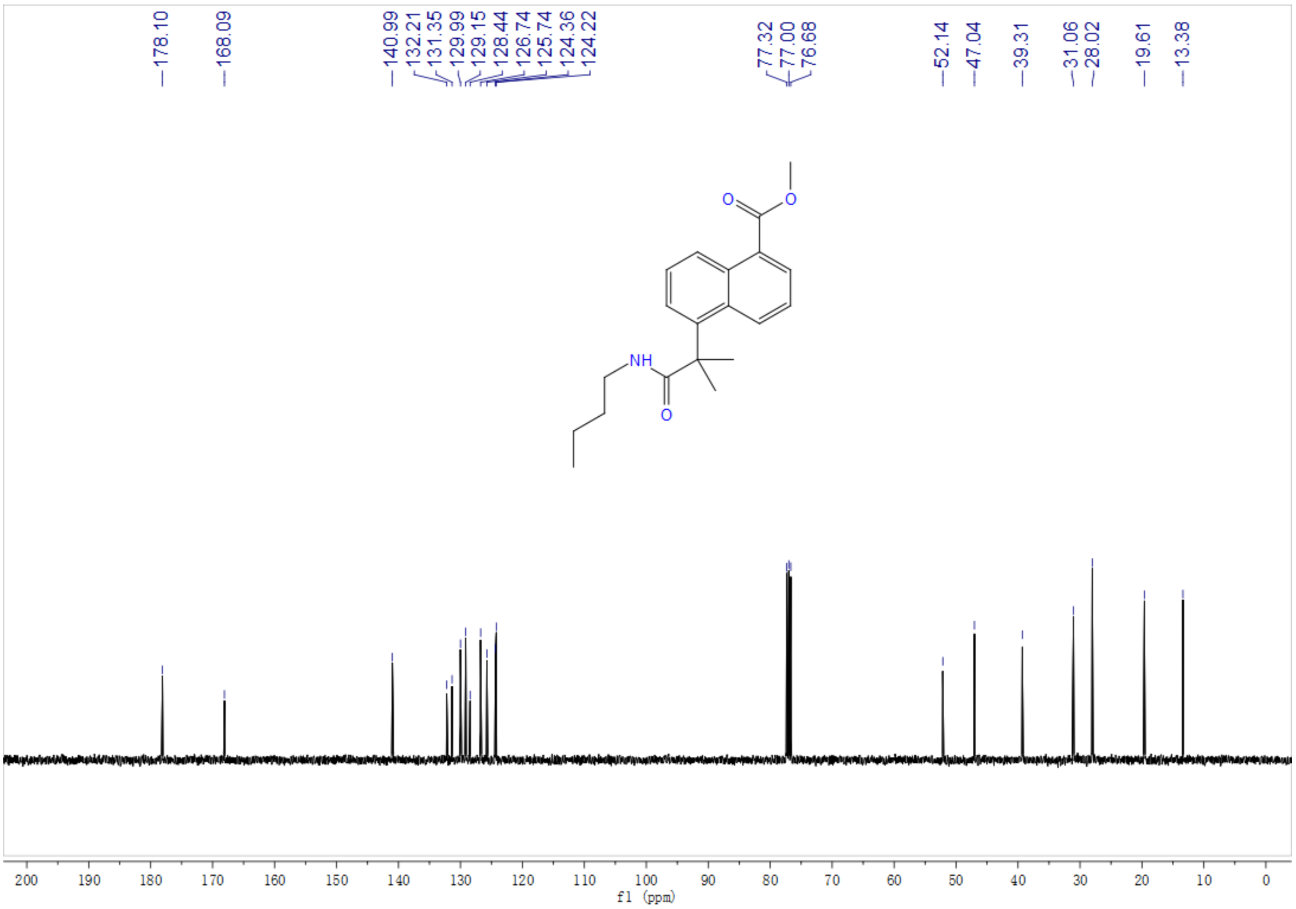

Methyl-5-(1-((furan-2-ylmethyl)amino)-2-methyl-1-oxopropan-2-yl)-1-naphthoate, **4ea**, ^{1}H NMR (500 MHz, $CDCl_3$) and ^{13}C NMR (125 MHz, $CDCl_3$)

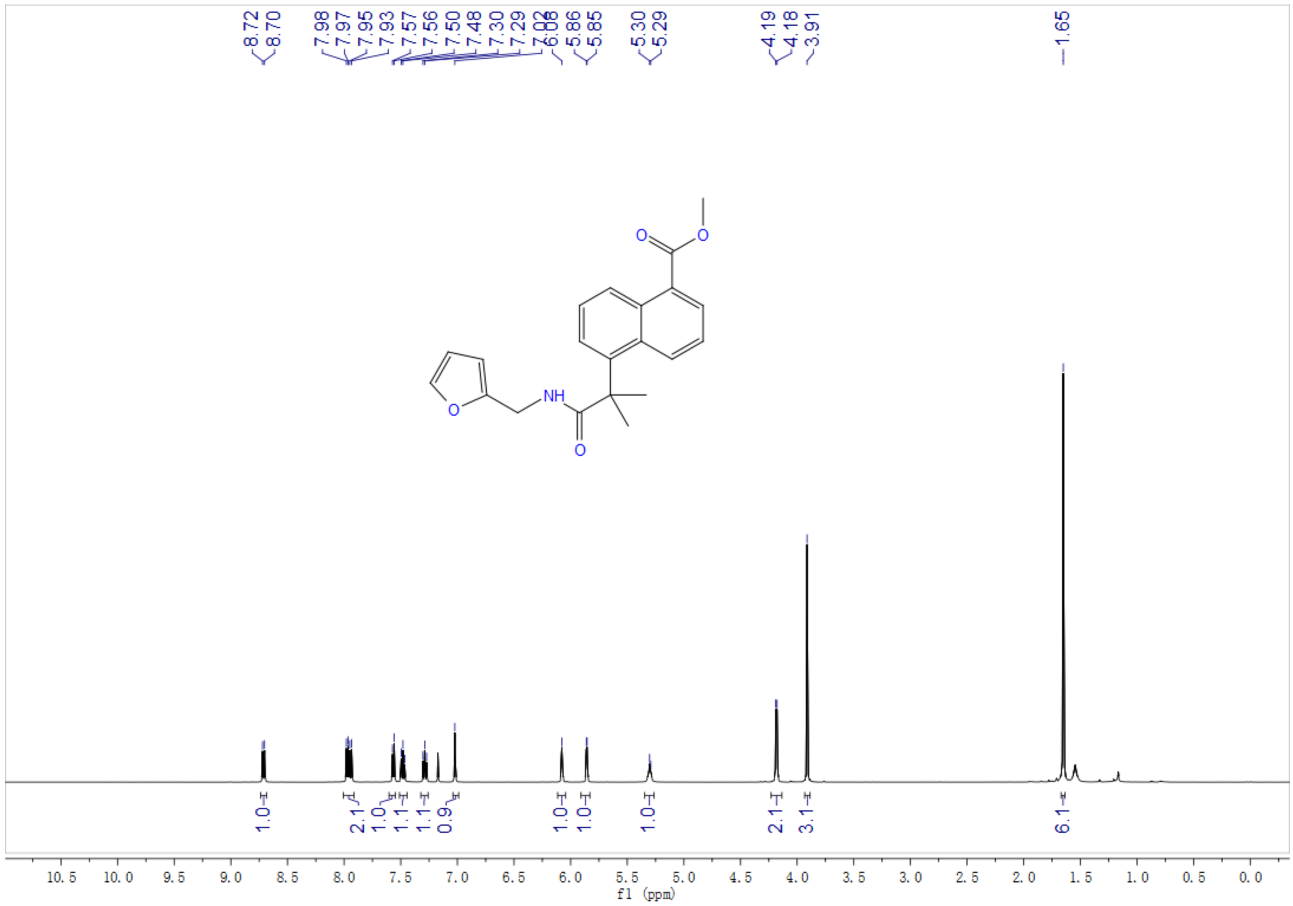

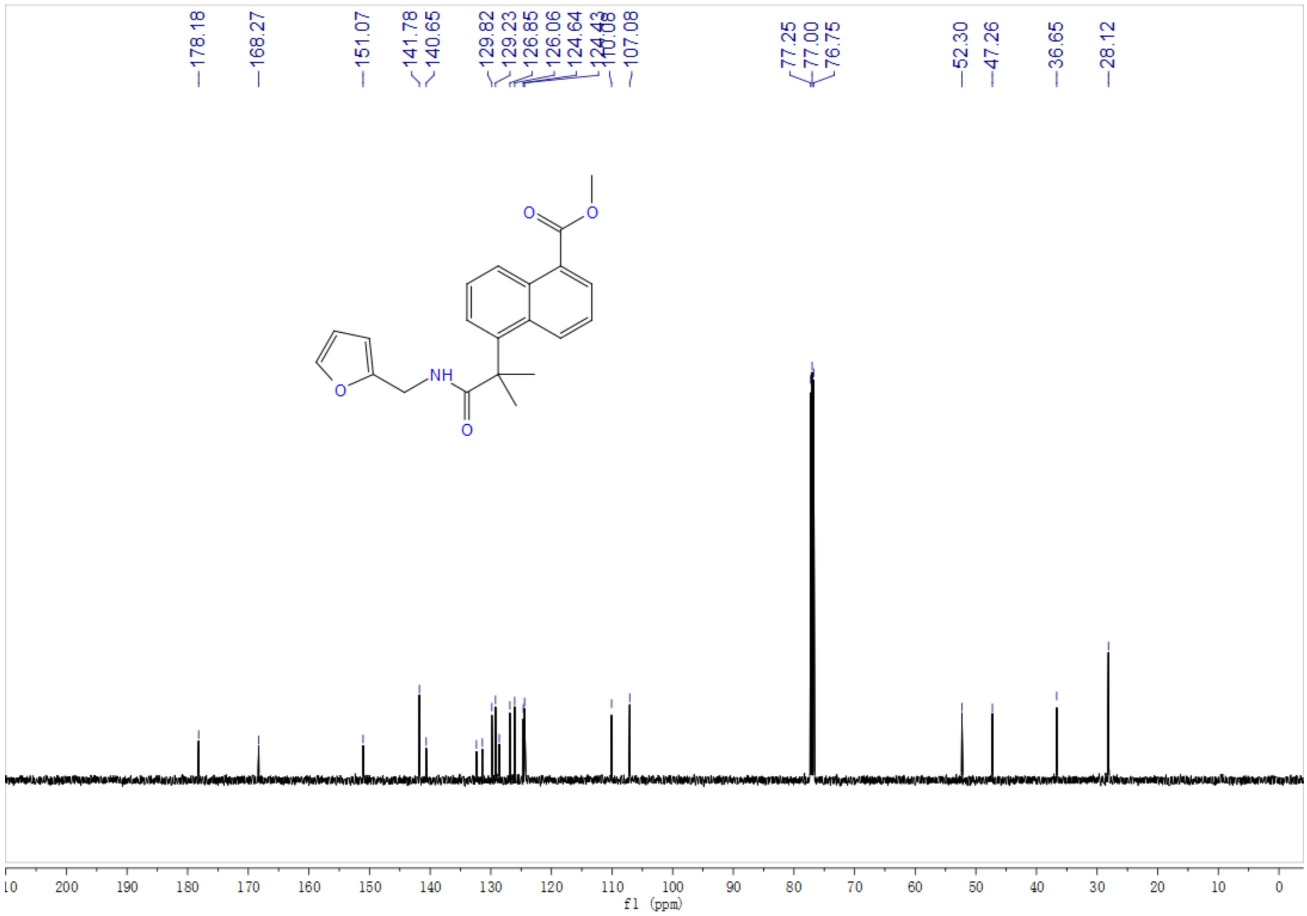

Methyl-5-(2-methyl-1-((6-methylpyridin-2-yl)amino)-1-oxopropan-2-yl)-1-naphthoate, **4fa**, ^{1}H NMR (500 MHz, $CDCl_3$) and ^{13}C NMR (125 MHz, $CDCl_3$)

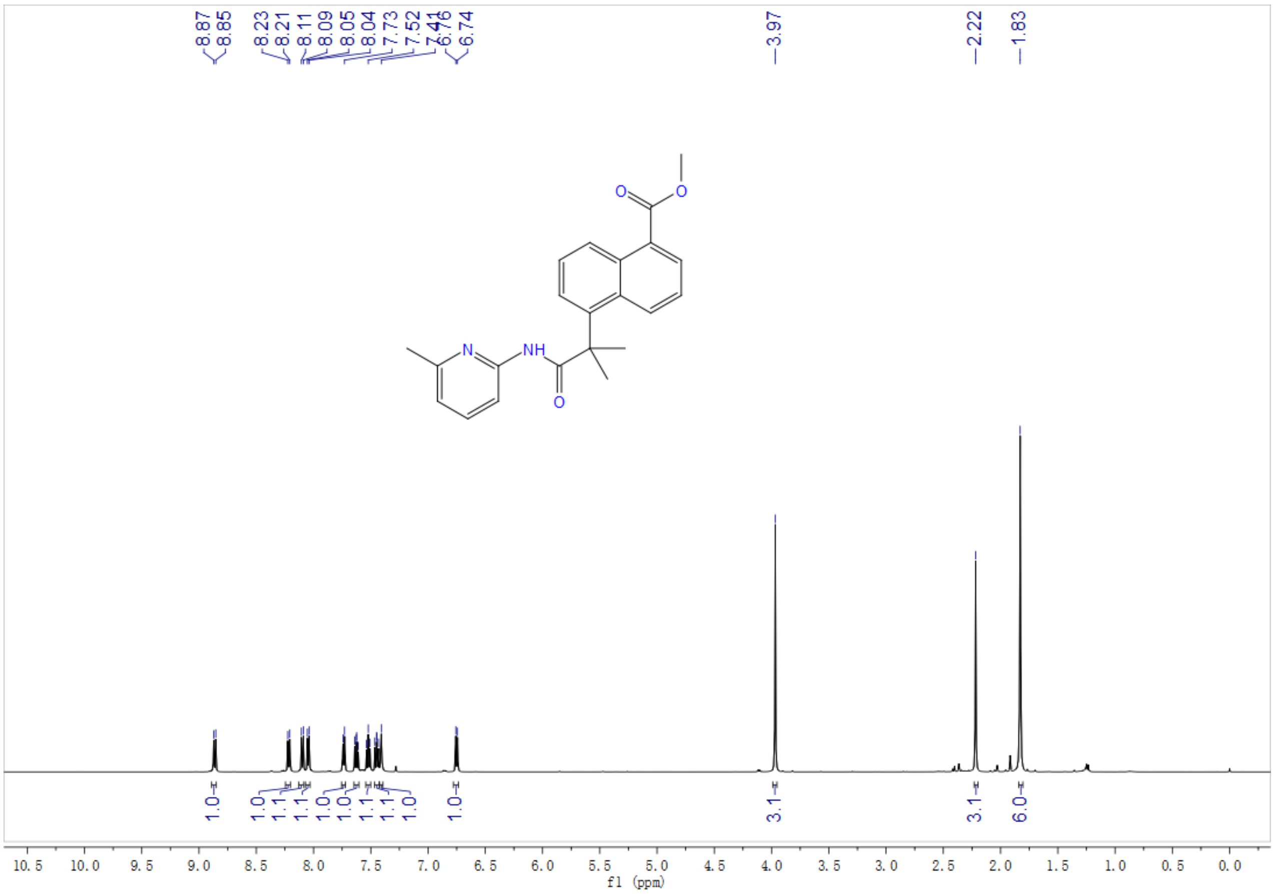

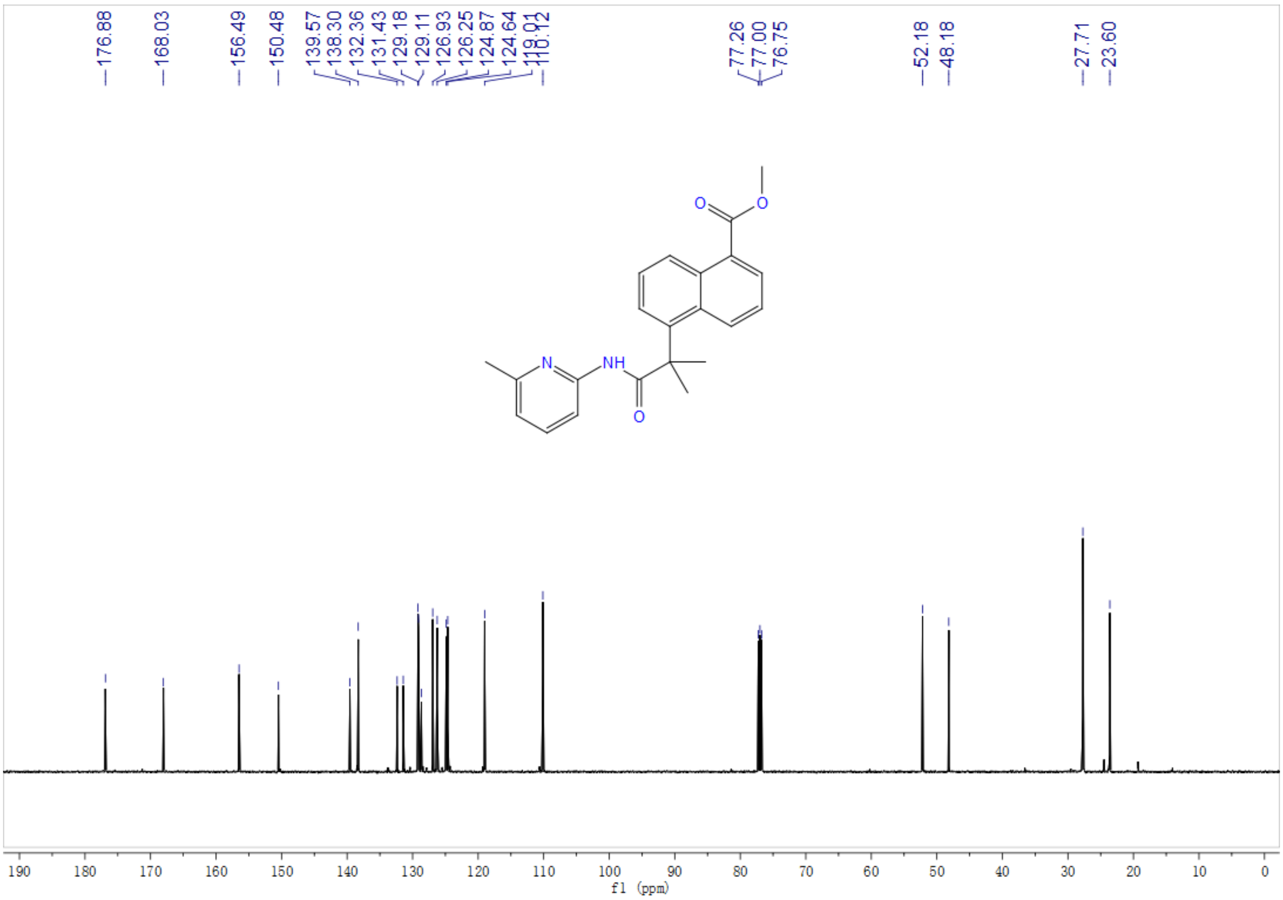

Methyl-5-(1-methoxy-2-methyl-1-oxopropan-2-yl)-1-naphthoate, **4ga**, ^{1}H NMR (500 MHz, $CDCl_3$) and ^{13}C NMR (125 MHz, $CDCl_3$)

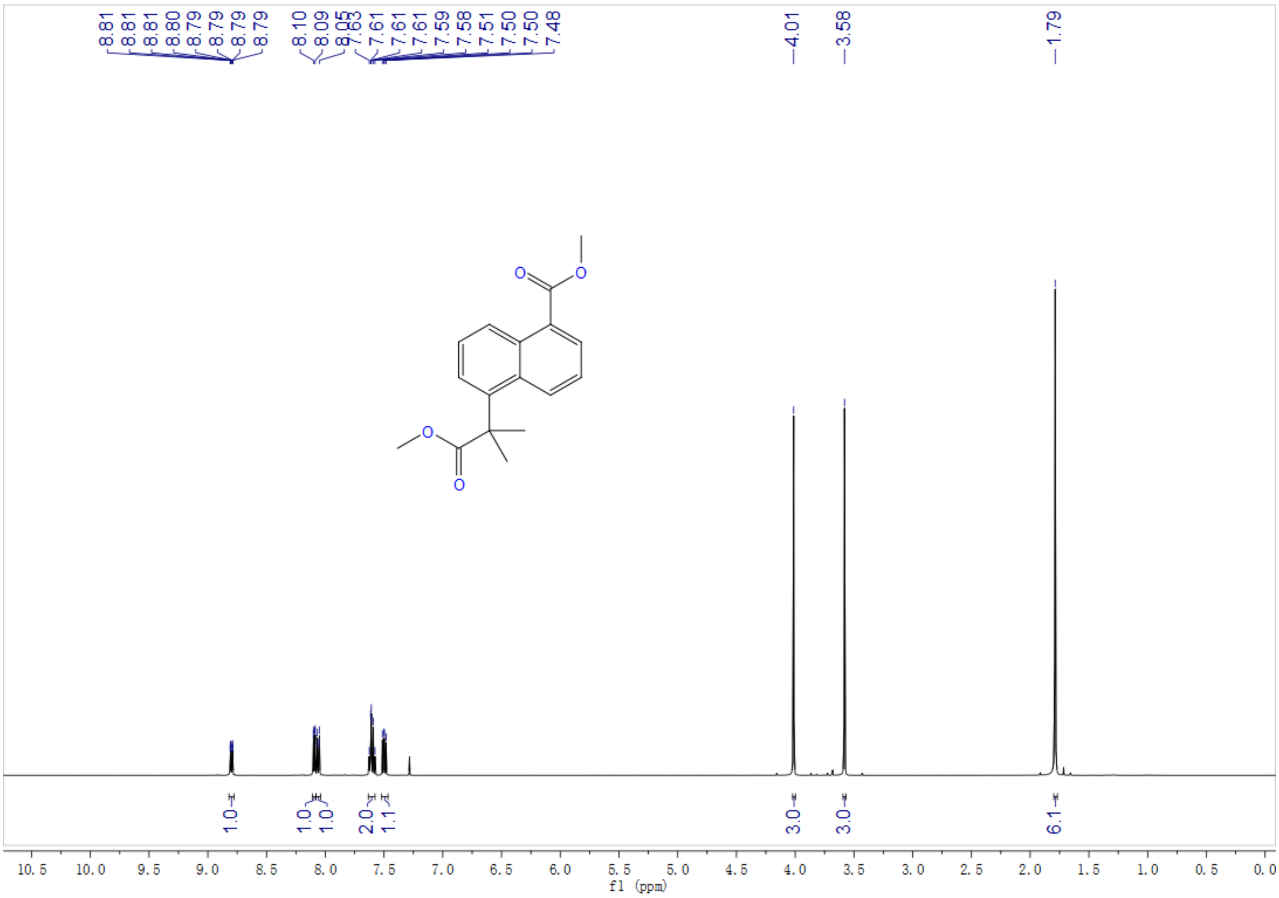

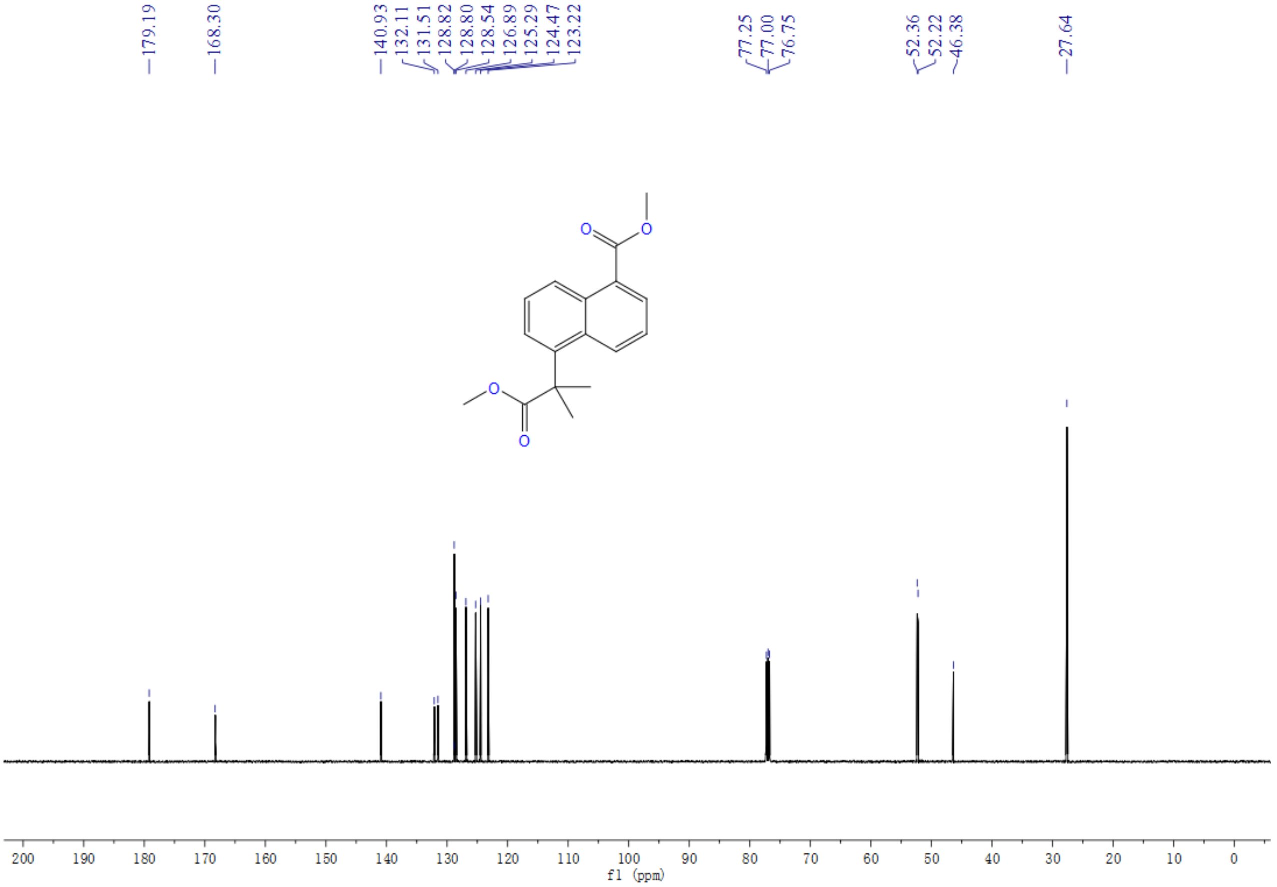

Methyl-5-(2-methyl-1-oxo-1-phenoxypropan-2-yl)-1-naphthoate, **4ha**, ^{1}H NMR (400 MHz, $CDCl_3$) and ^{13}C NMR (125 MHz, $CDCl_3$)

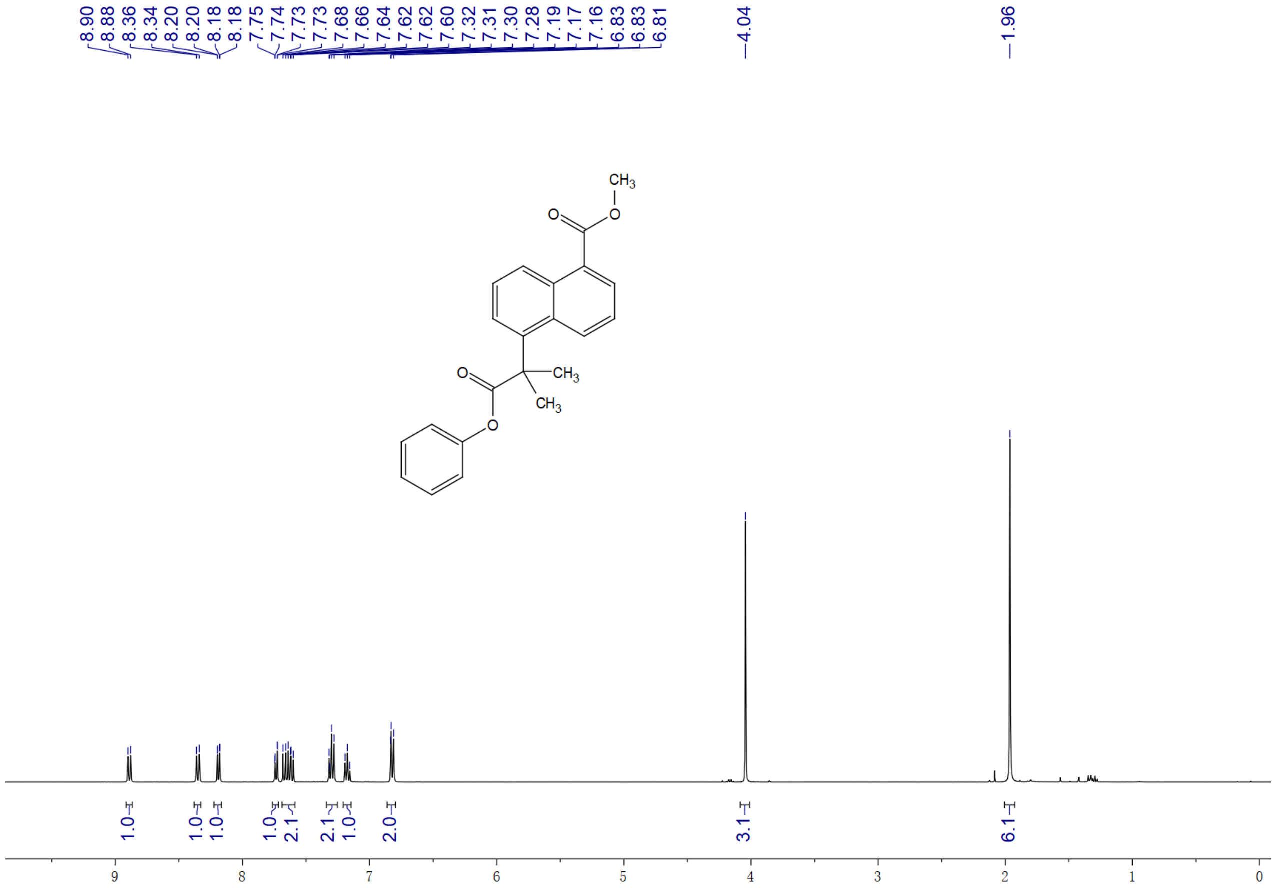

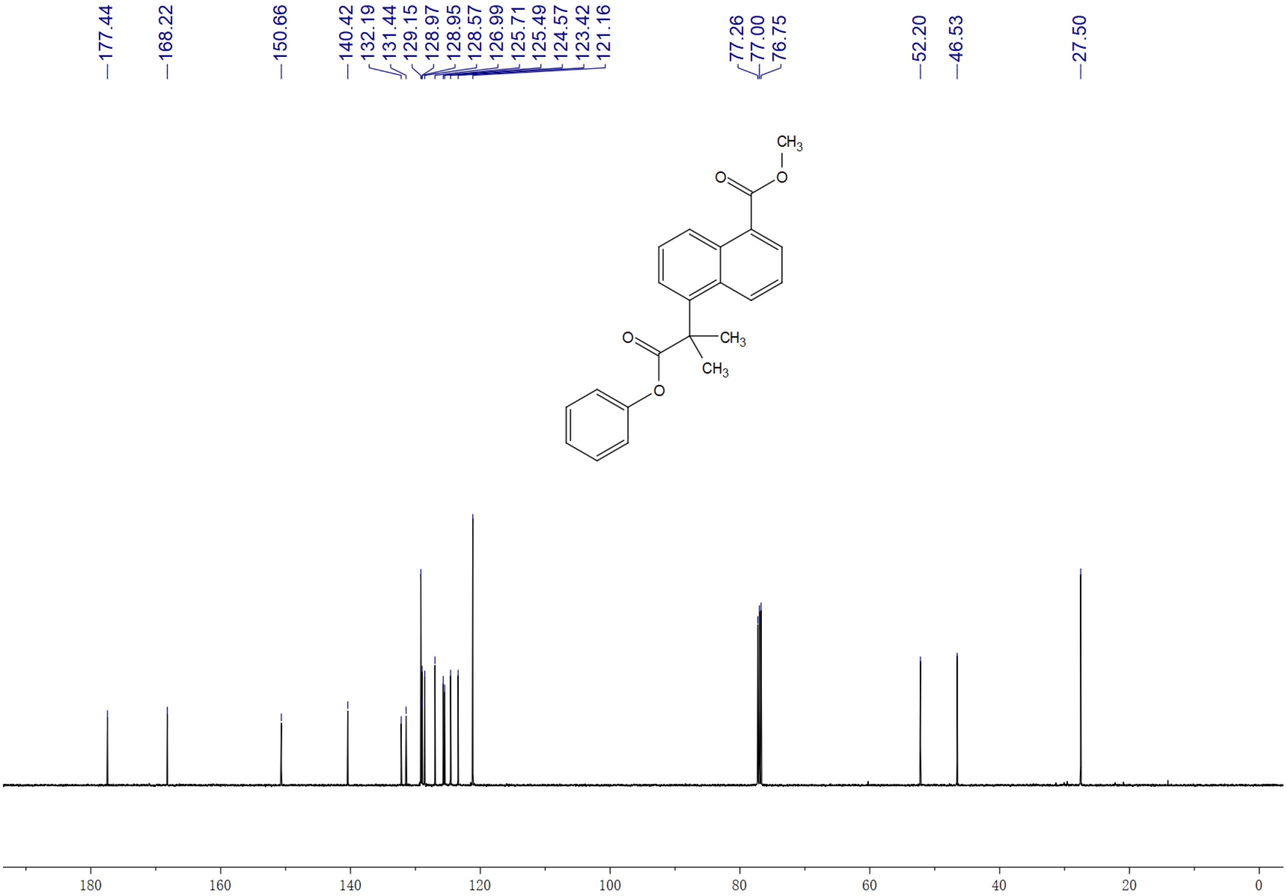

Methyl-5-(1-((2-methoxy-2-oxoethyl)amino)-2-methyl-1-oxopropan-2-yl)-1-naphthoate, **4ia**, ^{1}H NMR (500 MHz, $CDCl_3$) and ^{13}C NMR (125 MHz, $CDCl_3$)

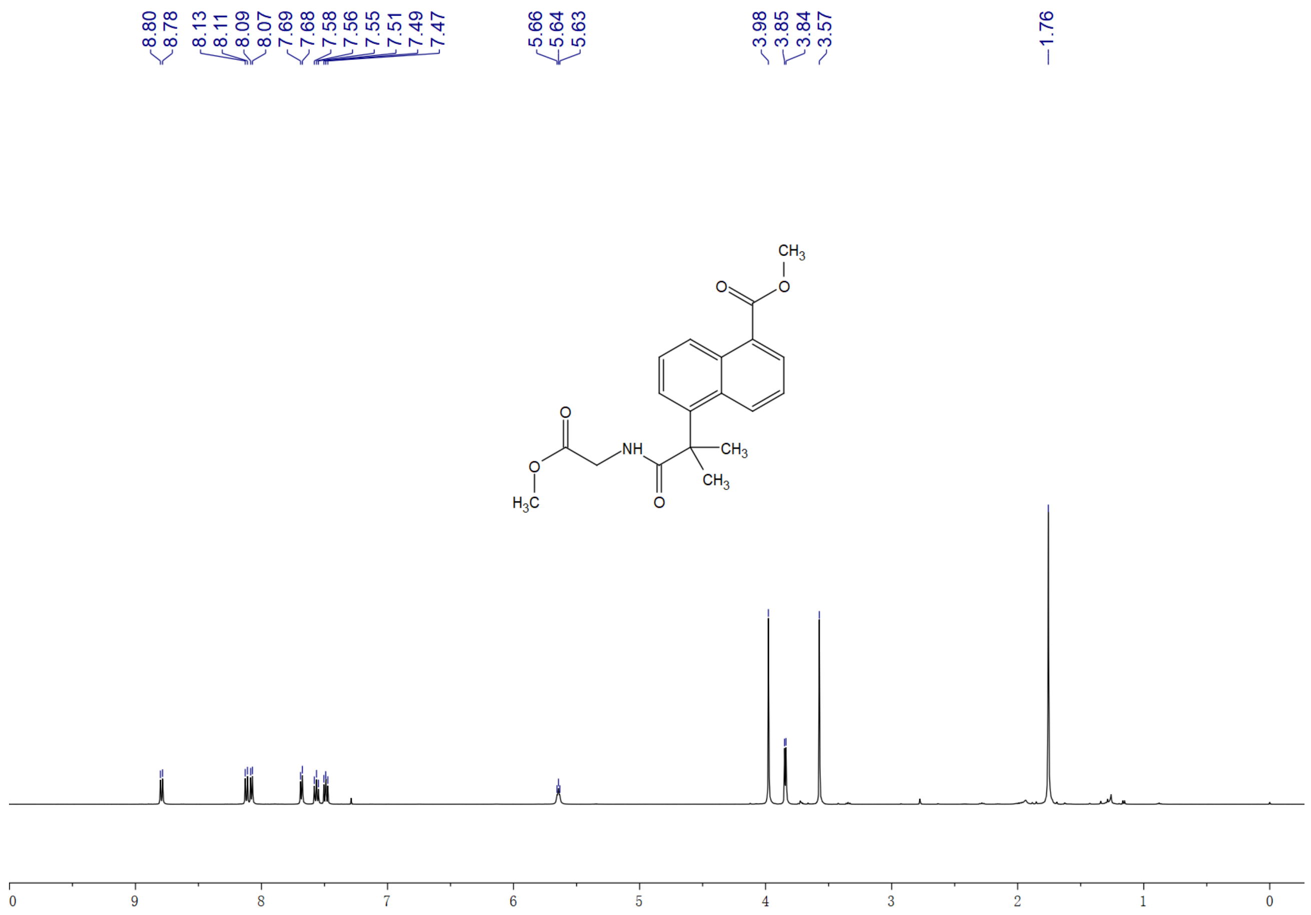

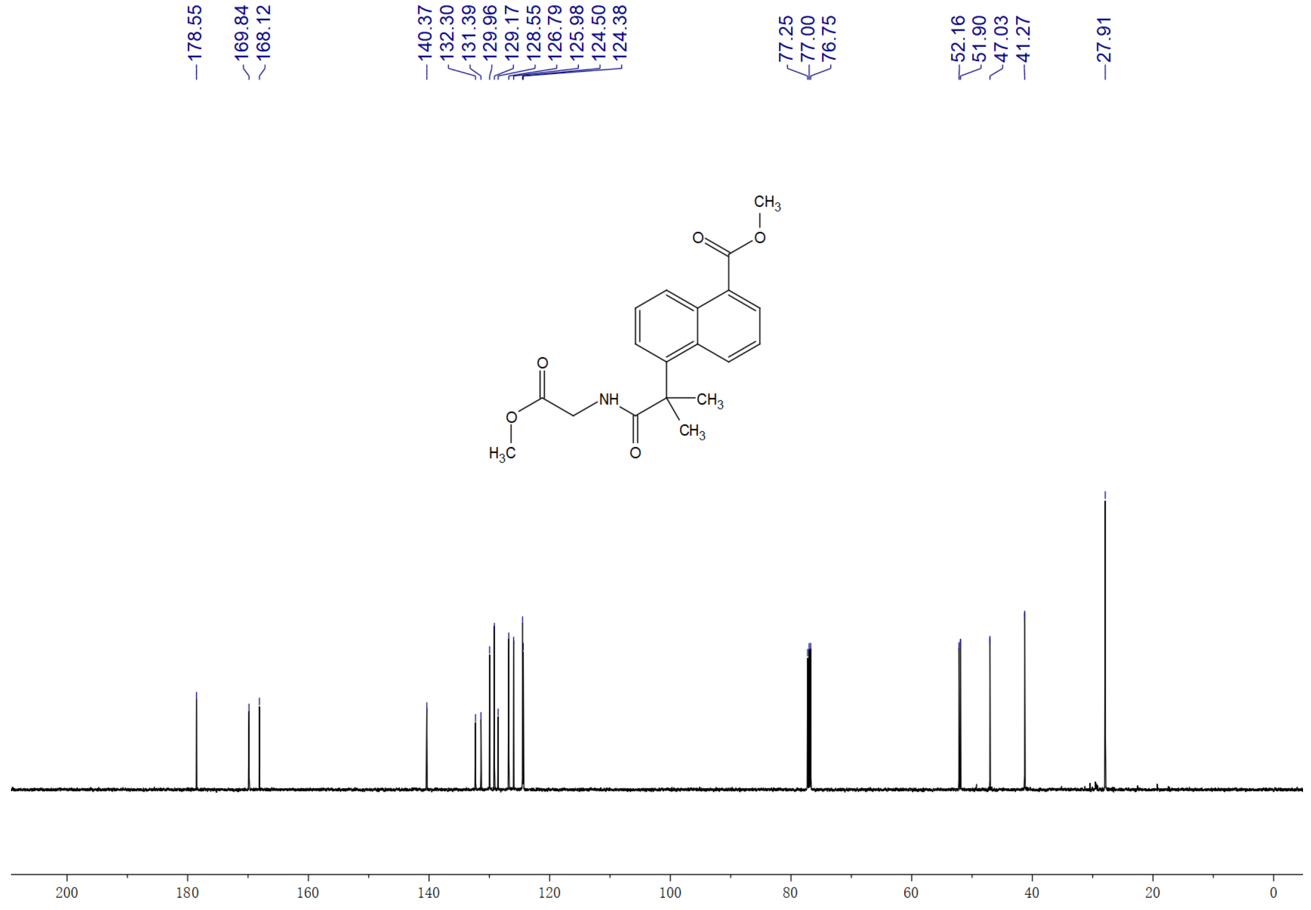

Methyl-5-(1-((1-methoxy-1-oxo-3-phenylpropan-2-yl)amino)-2-methyl-1-oxopropan-2-yl)-1-naphthoate, **4ja**, ^{1}H NMR (500 MHz, CDCl$_3$) and ^{13}C NMR (125 MHz, CDCl$_3$)

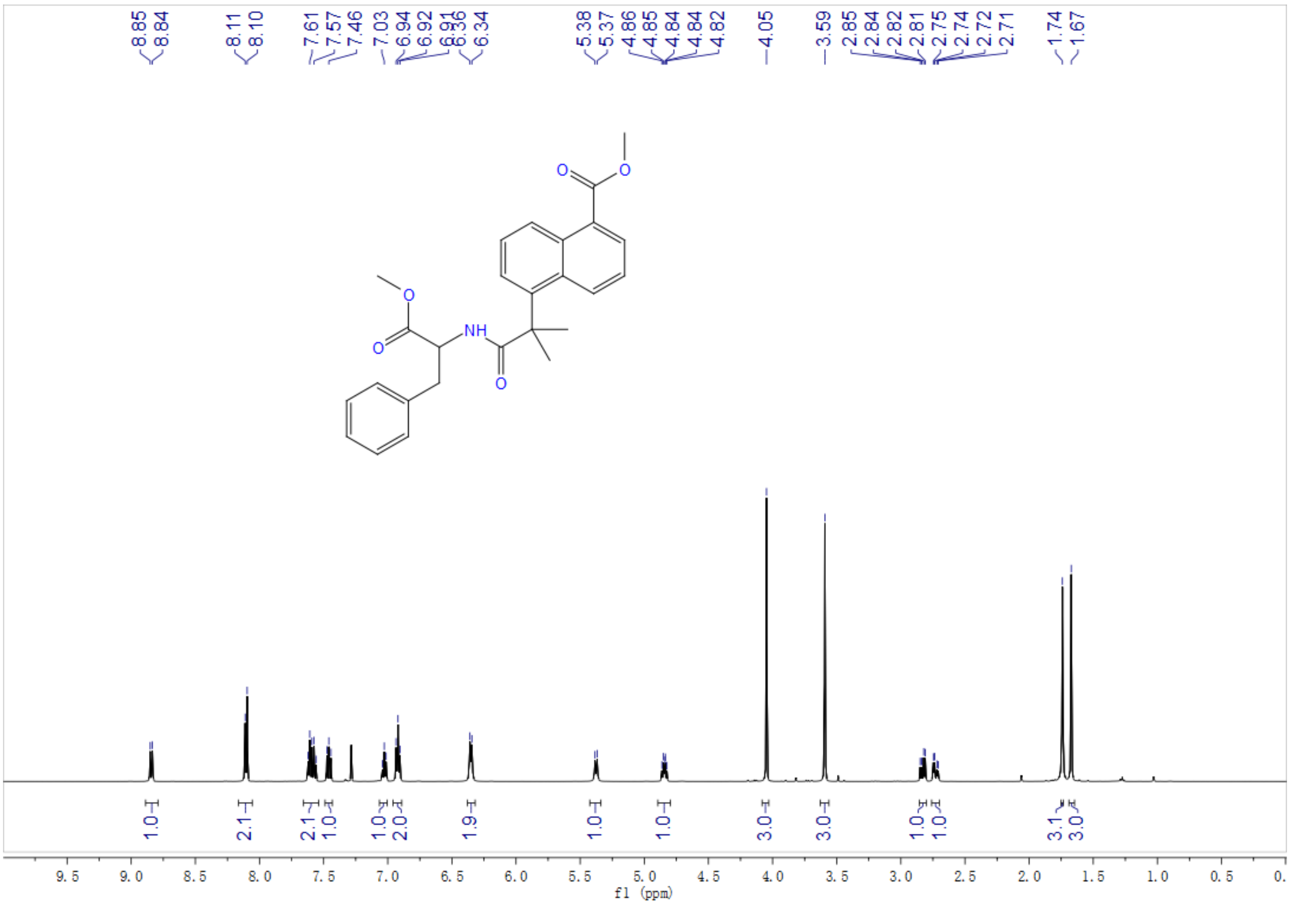

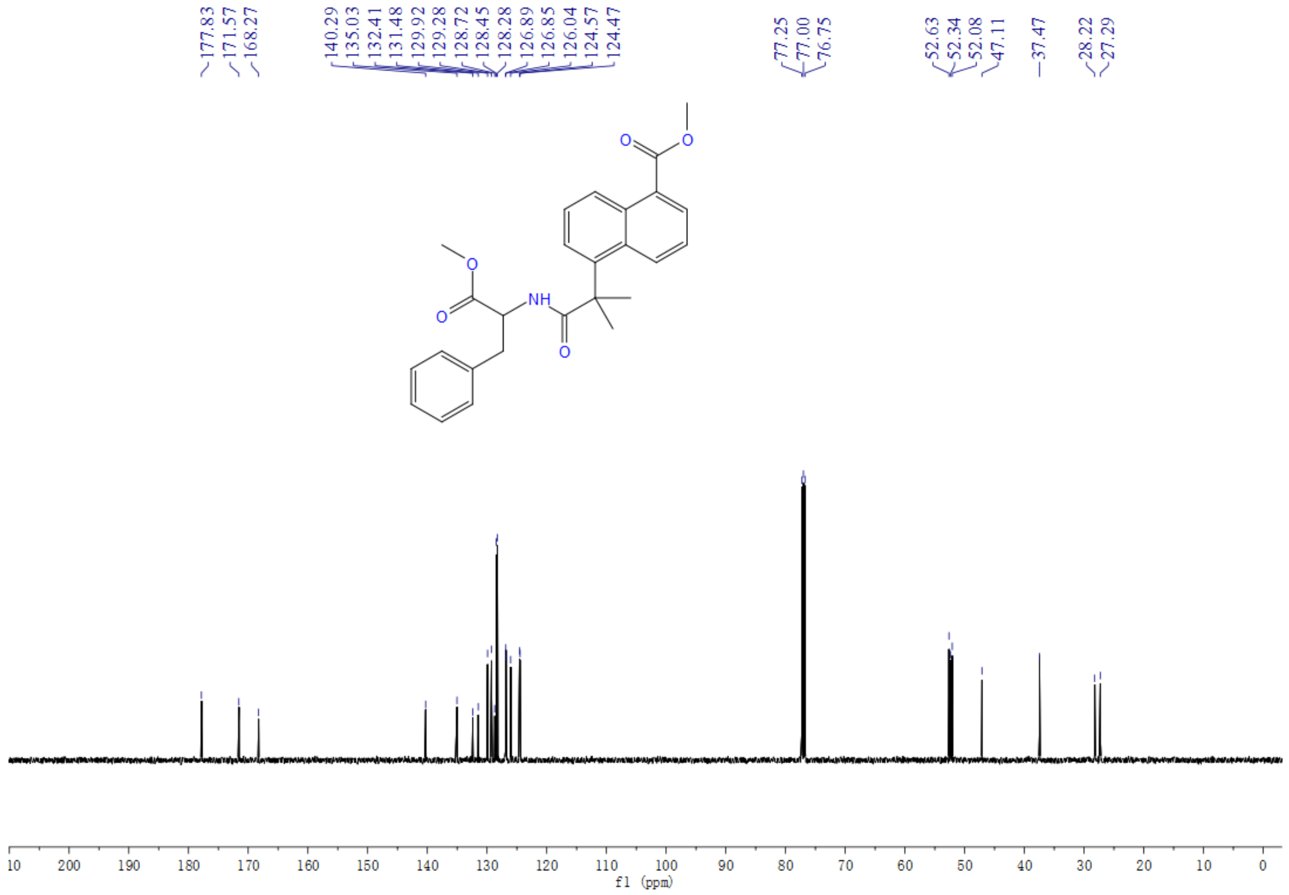

Methyl-5-(1-((1-methoxy-3-methyl-1-oxobutan-2-yl)amino)-2-methyl-1-oxopropan-2-yl)-1-naphthoate, **4ka**, ^{1}H NMR (500 MHz, $CDCl_3$) and ^{13}C NMR (125 MHz, $CDCl_3$)

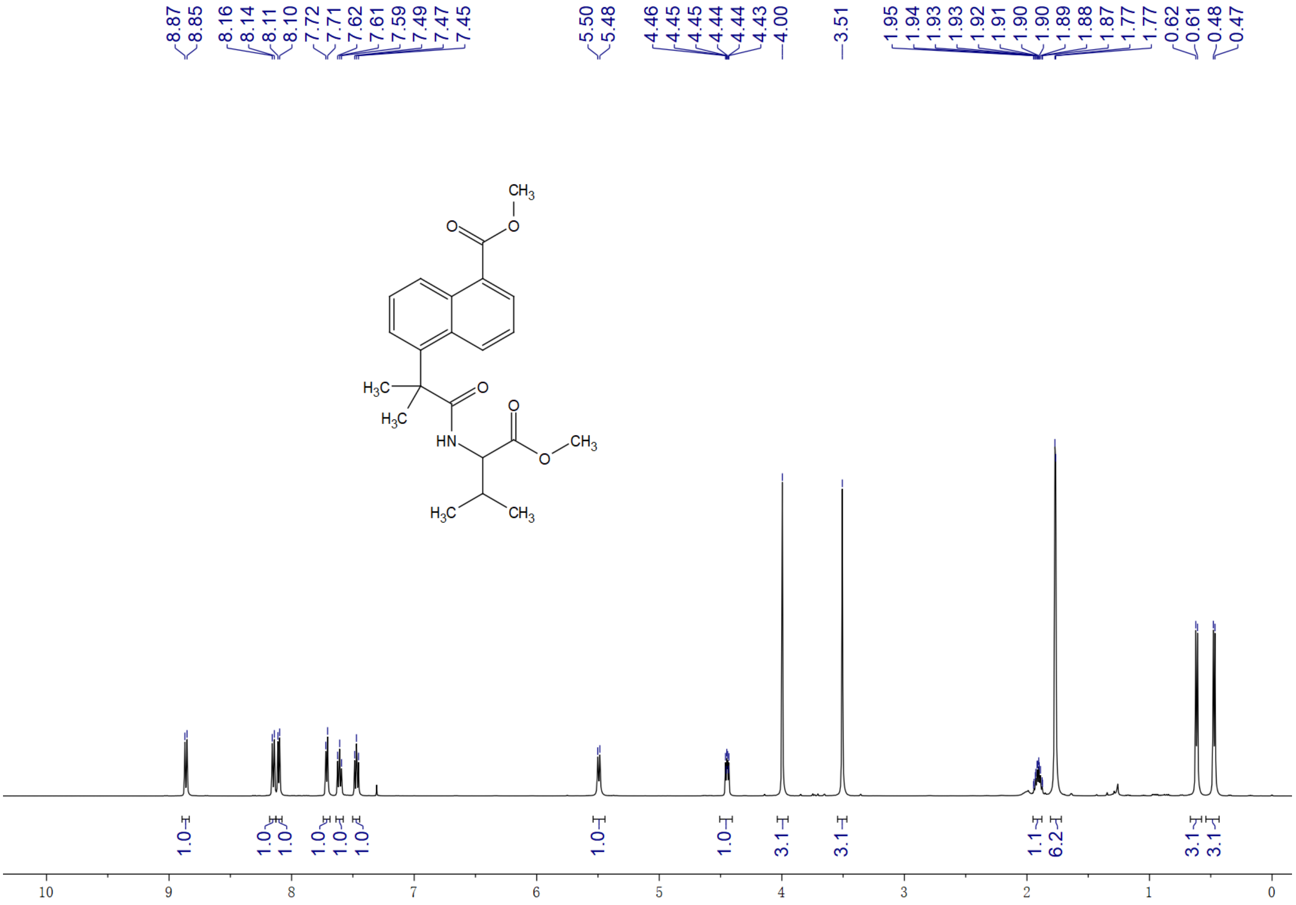

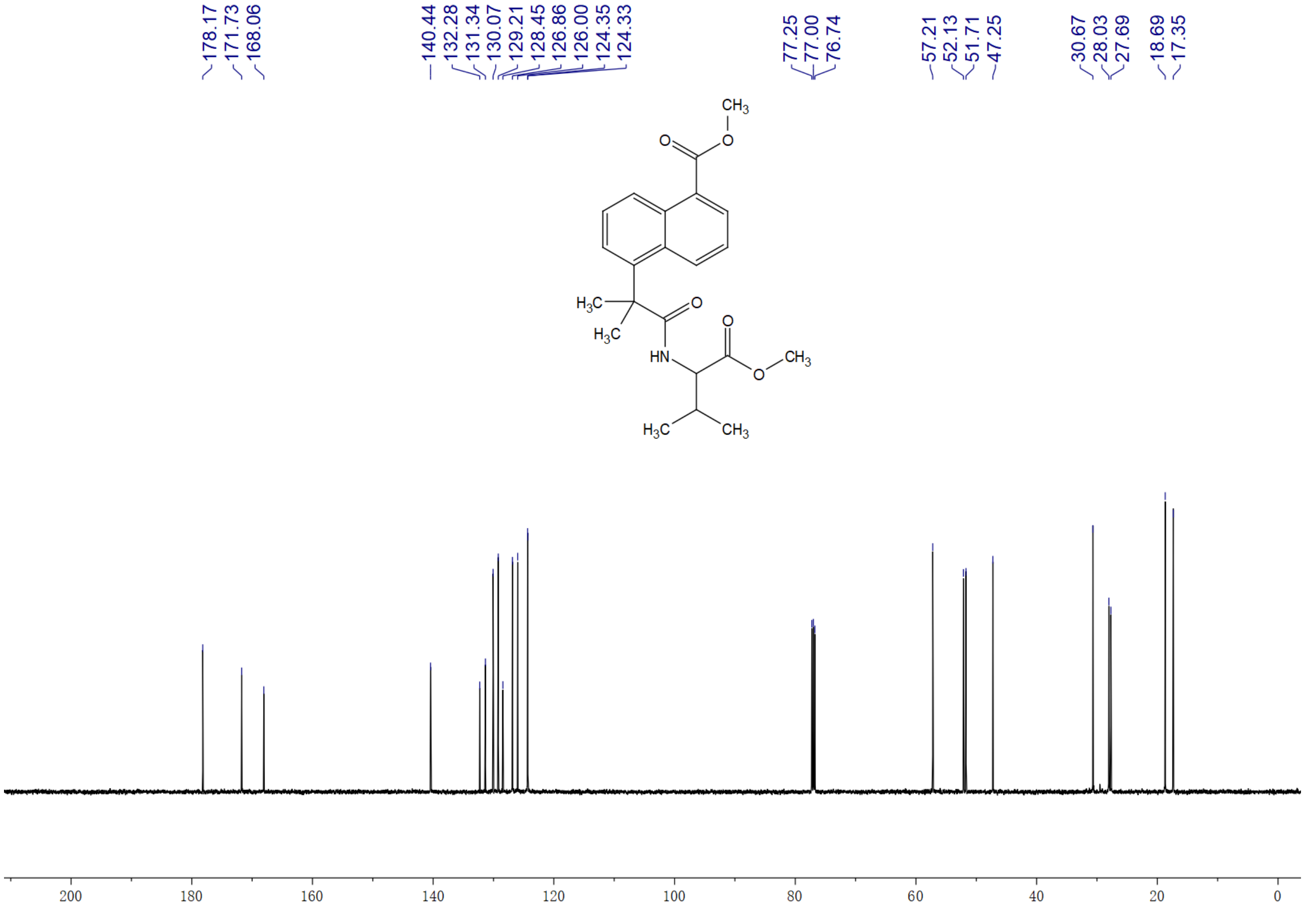

Methyl-5-(1-((3-(tert-butoxy)-1-methoxy-1-oxopropan-2-yl)amino)-2-methyl-1-oxopropan-2-yl)-1-naphthoate, **4la**, ^{1}H NMR (500 MHz, $CDCl_3$) and ^{13}C NMR (125 MHz, $CDCl_3$)

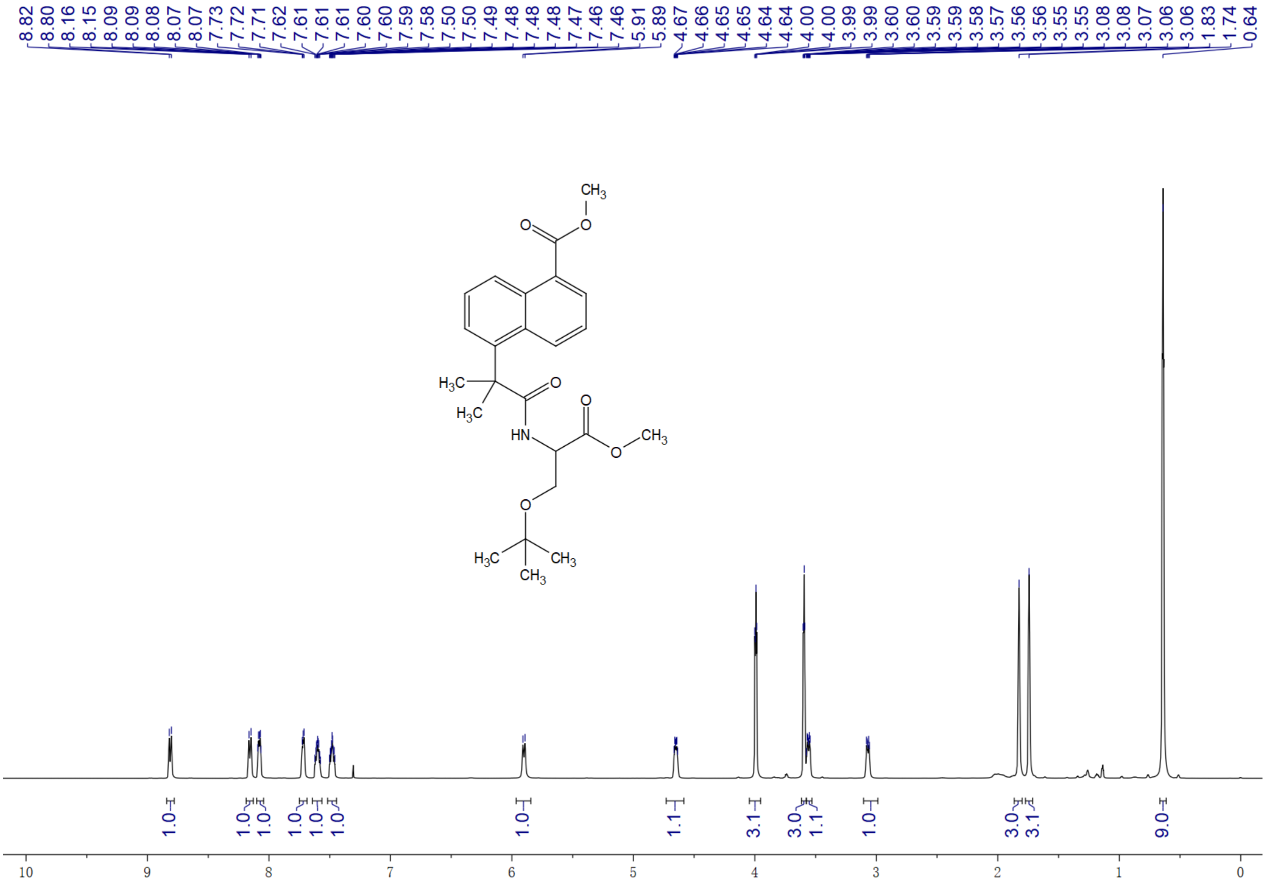

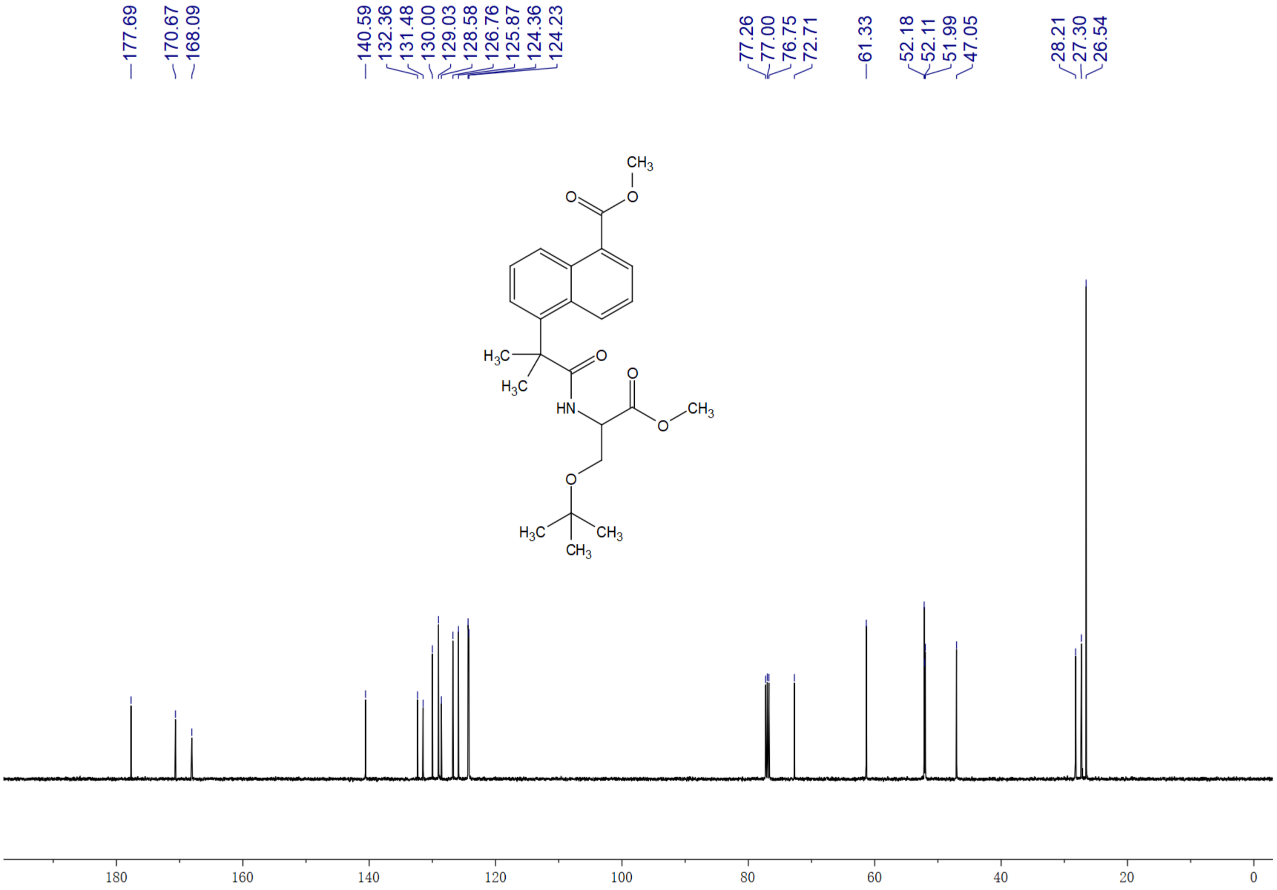

Dimethyl-(2-(5-(methoxycarbonyl)naphthalen-1-yl)-2-methylpropanoyl)aspartate, **4ma**, ^{1}H NMR (500 MHz, $CDCl_3$) and ^{13}C NMR (125 MHz, $CDCl_3$)

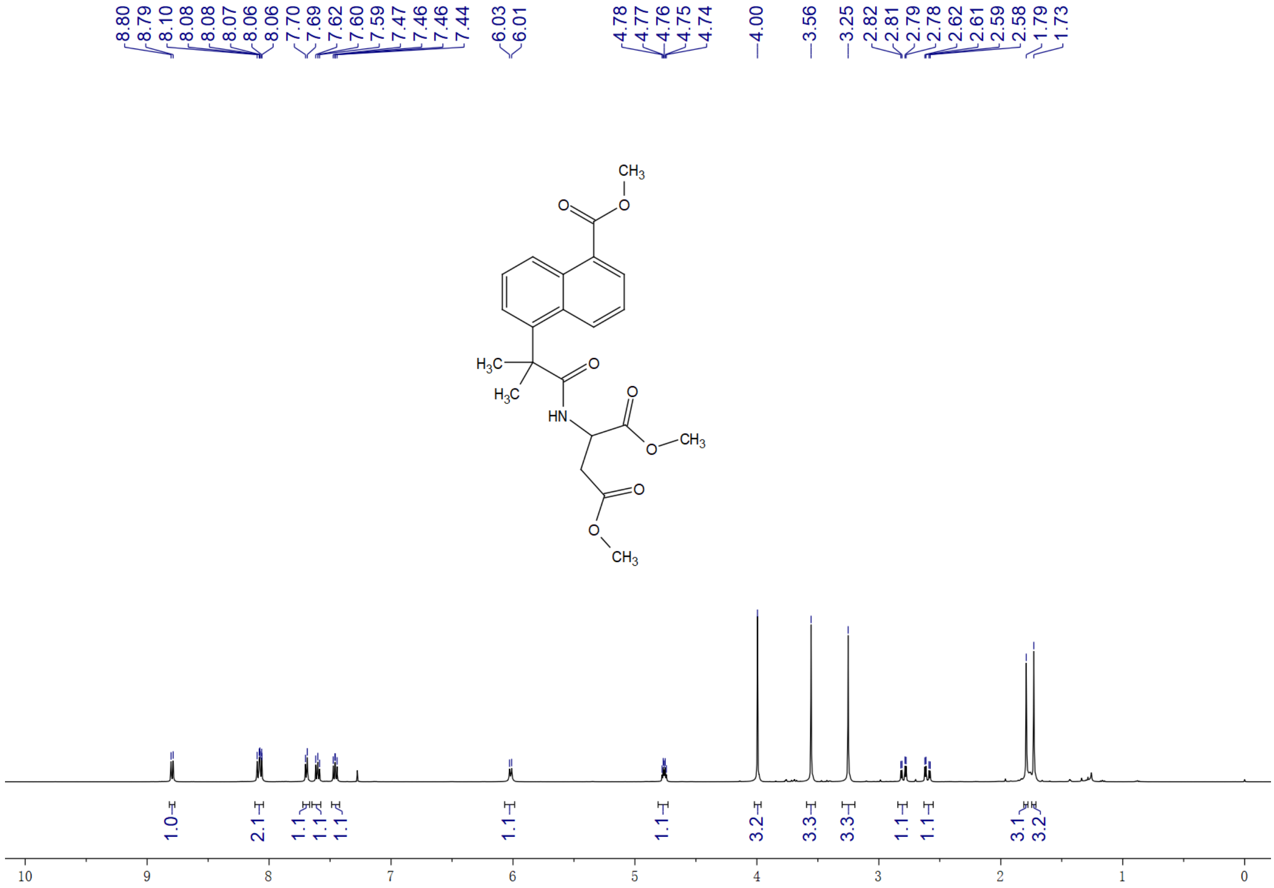

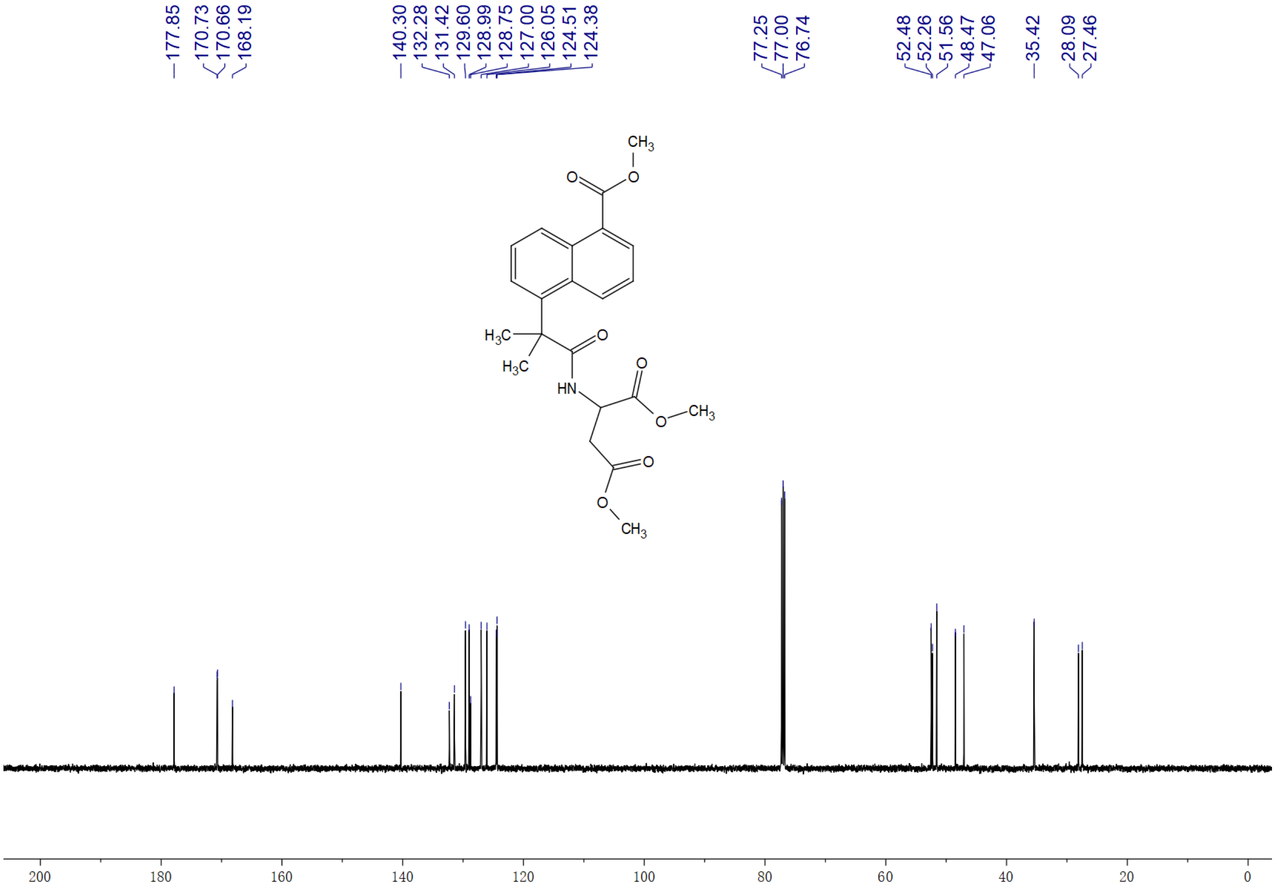

Methyl-6-methoxy-5-(2-methyl-1-oxo-1-(phenylamino)propan-2-yl)-1-naphthoate, **4na**, ^{1}H NMR (400 MHz, $CDCl_3$) and ^{13}C NMR (100 MHz, $CDCl_3$)

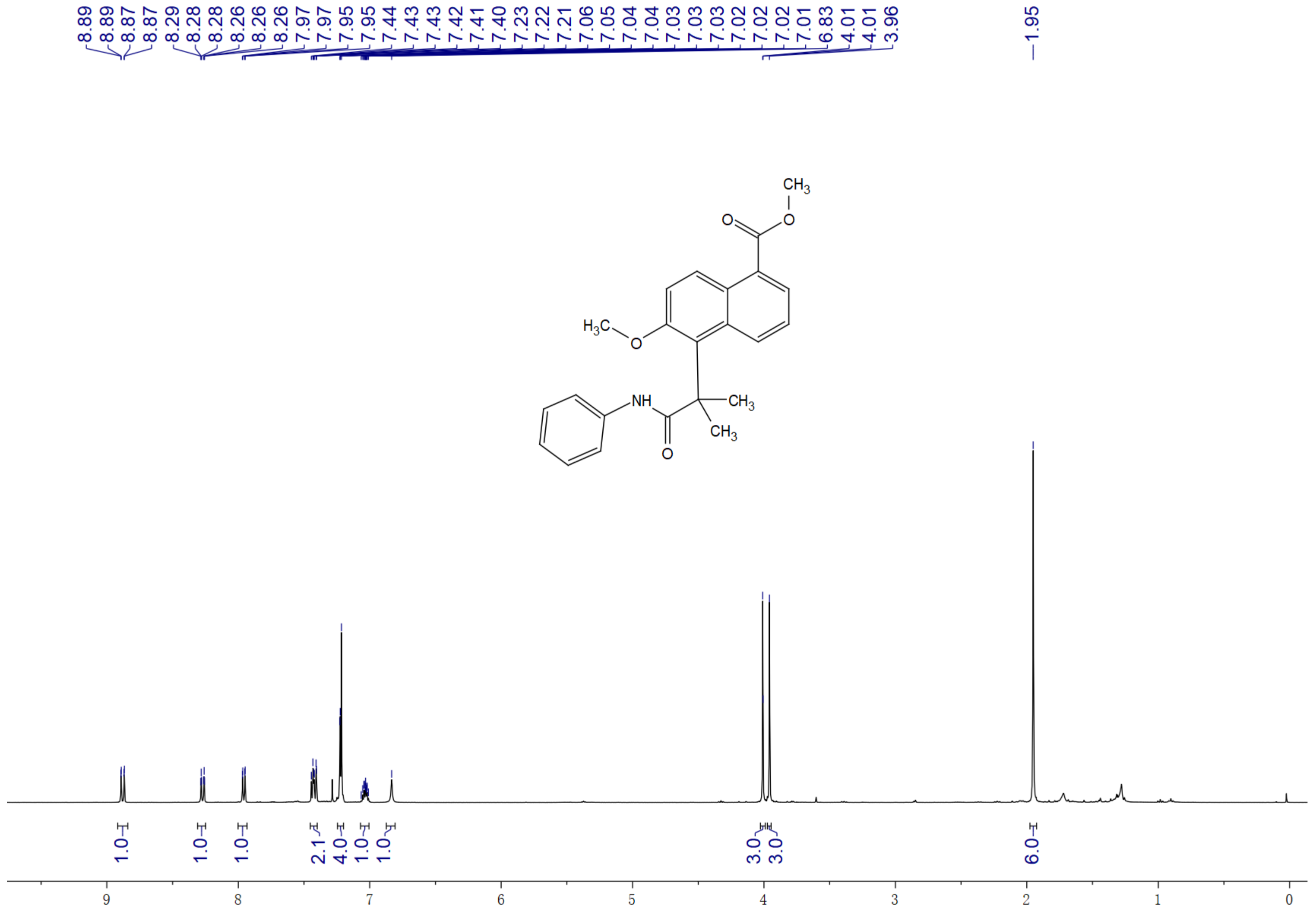

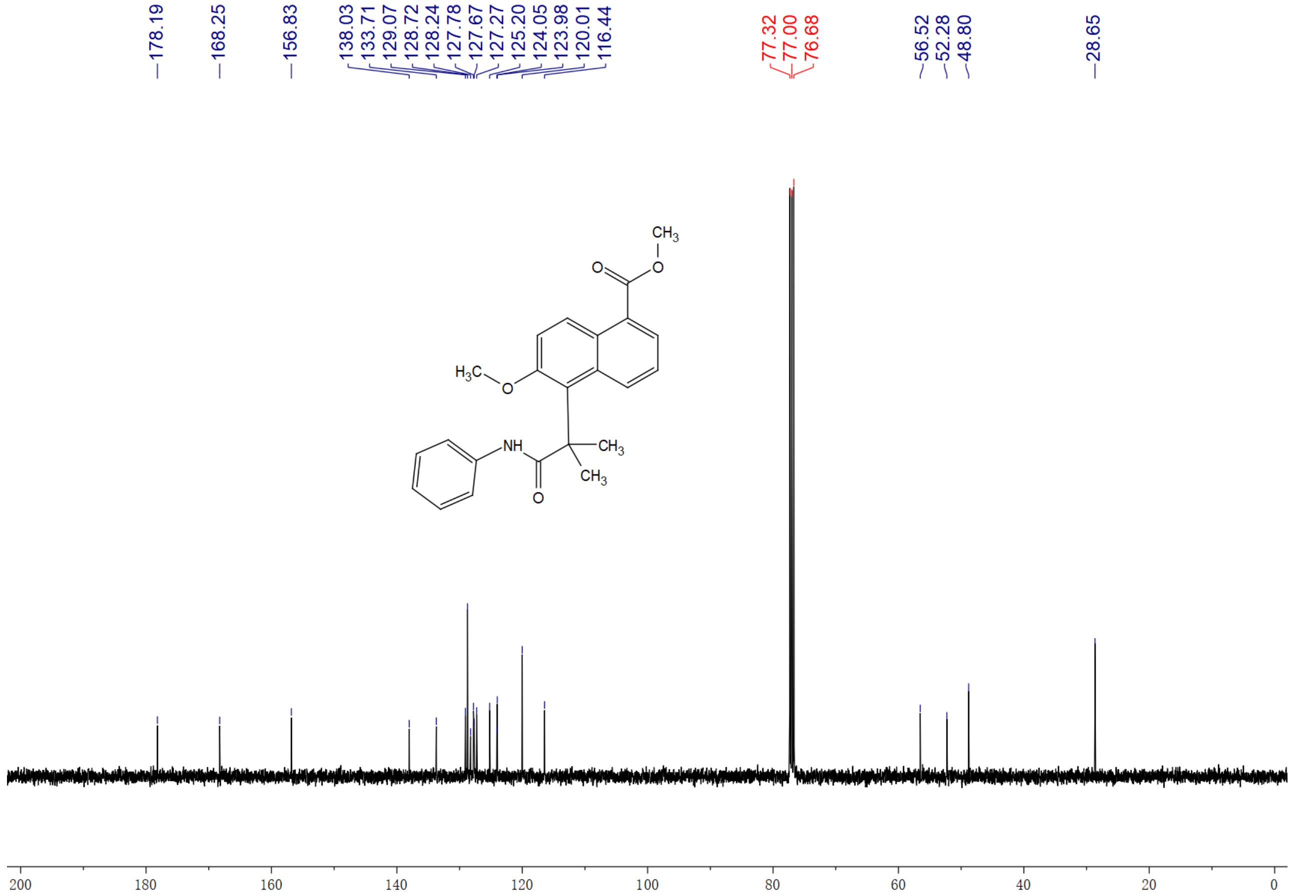

Methyl-5-(2-methyl-1-oxo-1-(((1S,2R,4S)-1,7,7-trimethylbicyclo[2.2.1]heptan-2-yl)oxy)propan-2-yl)-1-naphthoate, **4oa**, ^{1}H NMR (400 MHz, $CDCl_3$) and ^{13}C NMR (125 MHz, $CDCl_3$)

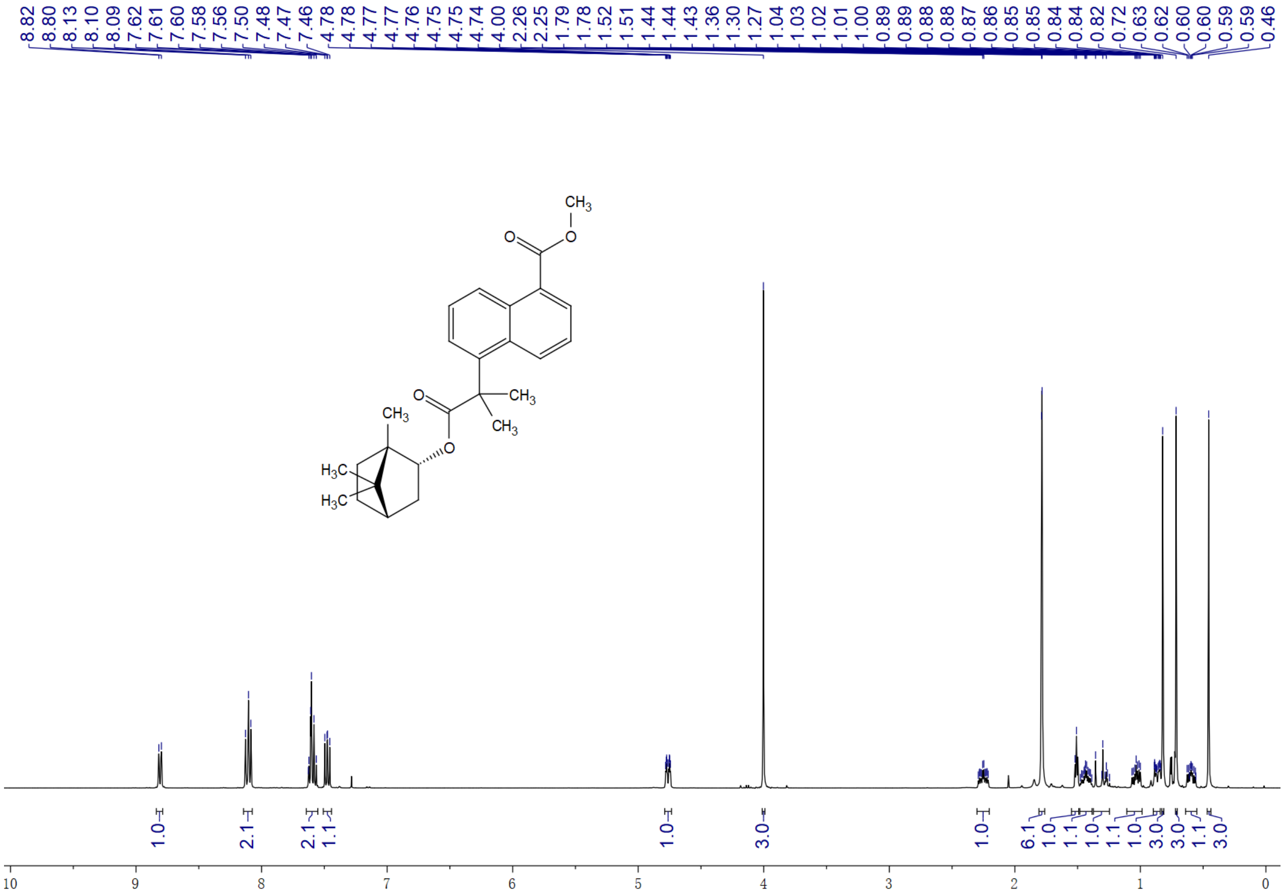

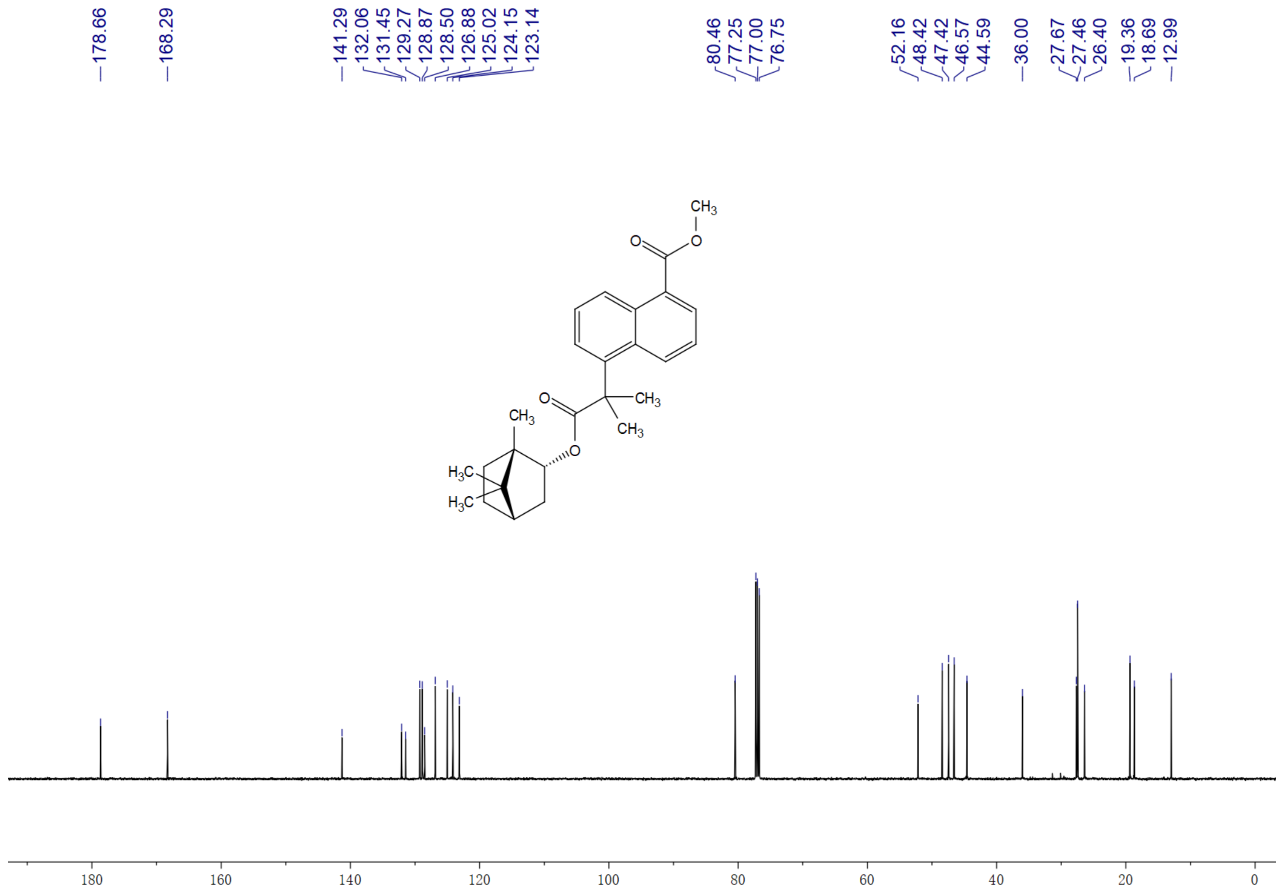

Methyl-5-(1-(((2S,5S)-5-isopropyl-2-methylcyclohexyl)oxy)-2-methyl-1-oxopropan-2-yl)-1-naphthoate, **4pa**, ^{1}H NMR (500 MHz, $CDCl_3$) and ^{13}C NMR (125 MHz, $CDCl_3$)

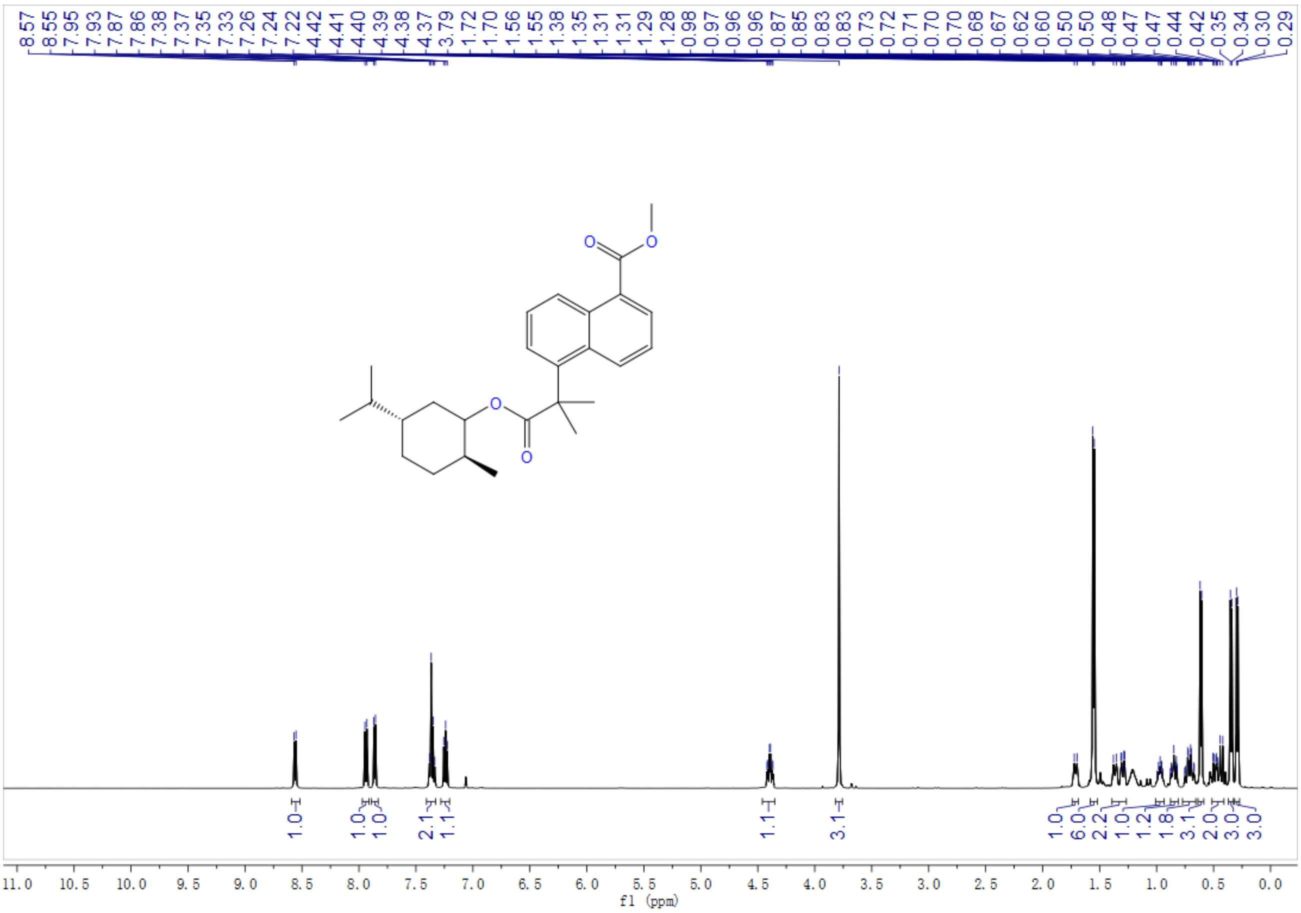

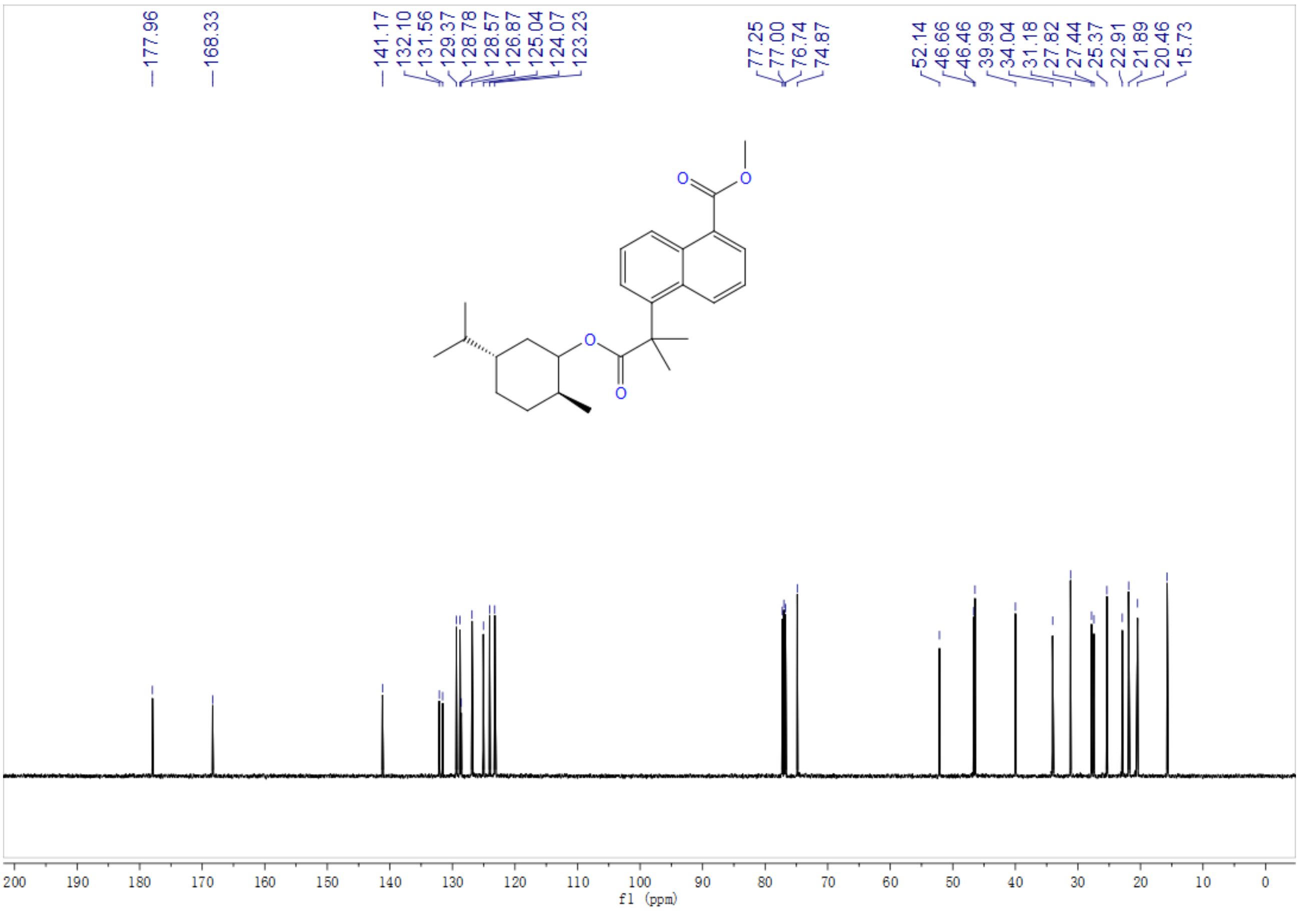

Methyl-5-(2-methyl-1-oxo-1-((2,2,7,7-tetramethyltetrahydro-5H-bis([1,3]dioxolo)[4,5-b:4',5'-d]pyran-5-yl)methoxy)propan-2-yl)-1-naphthoate, **4qa**, ^{1}H NMR (500 MHz, $CDCl_3$) and ^{13}C NMR (125 MHz, $CDCl_3$)

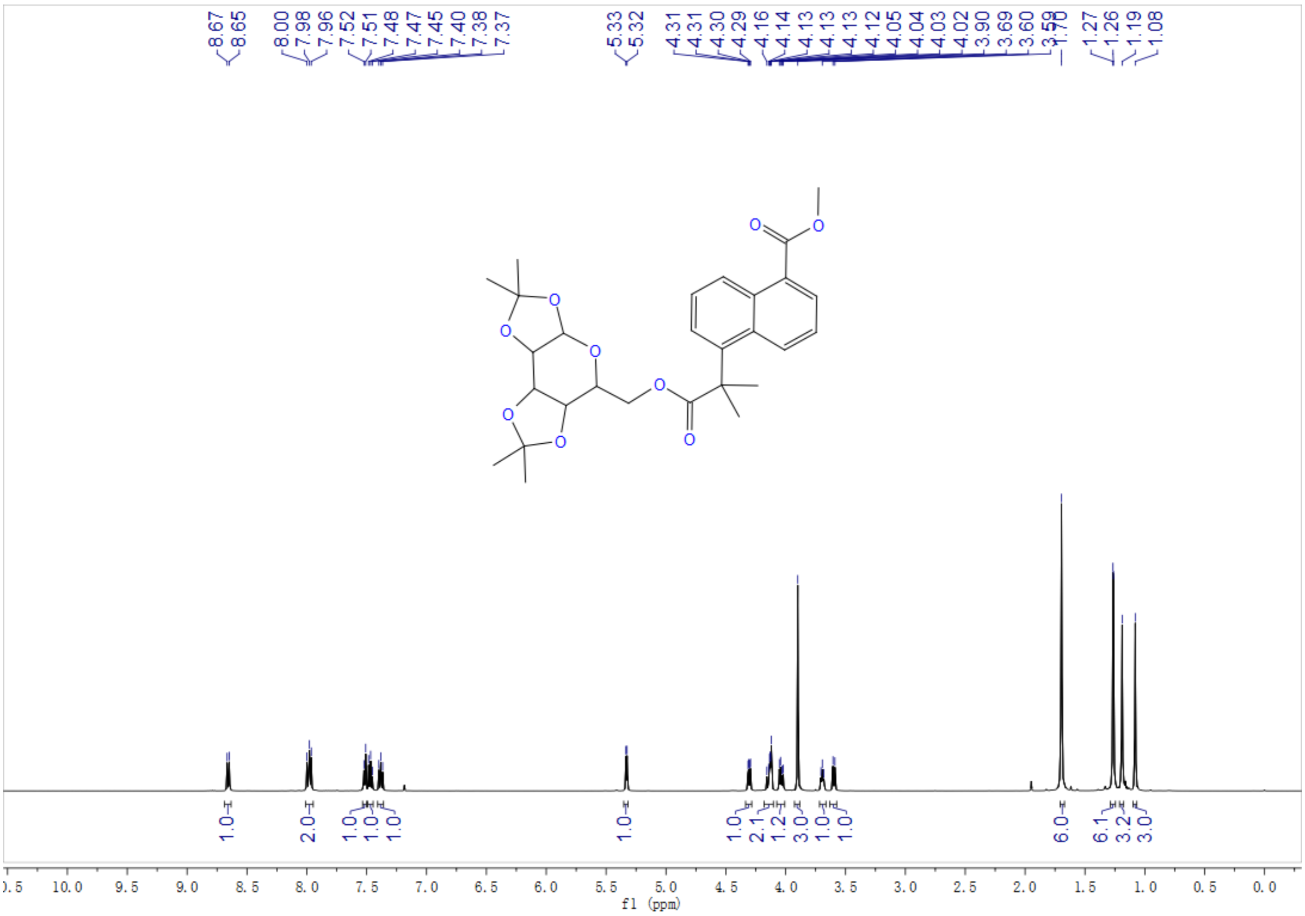

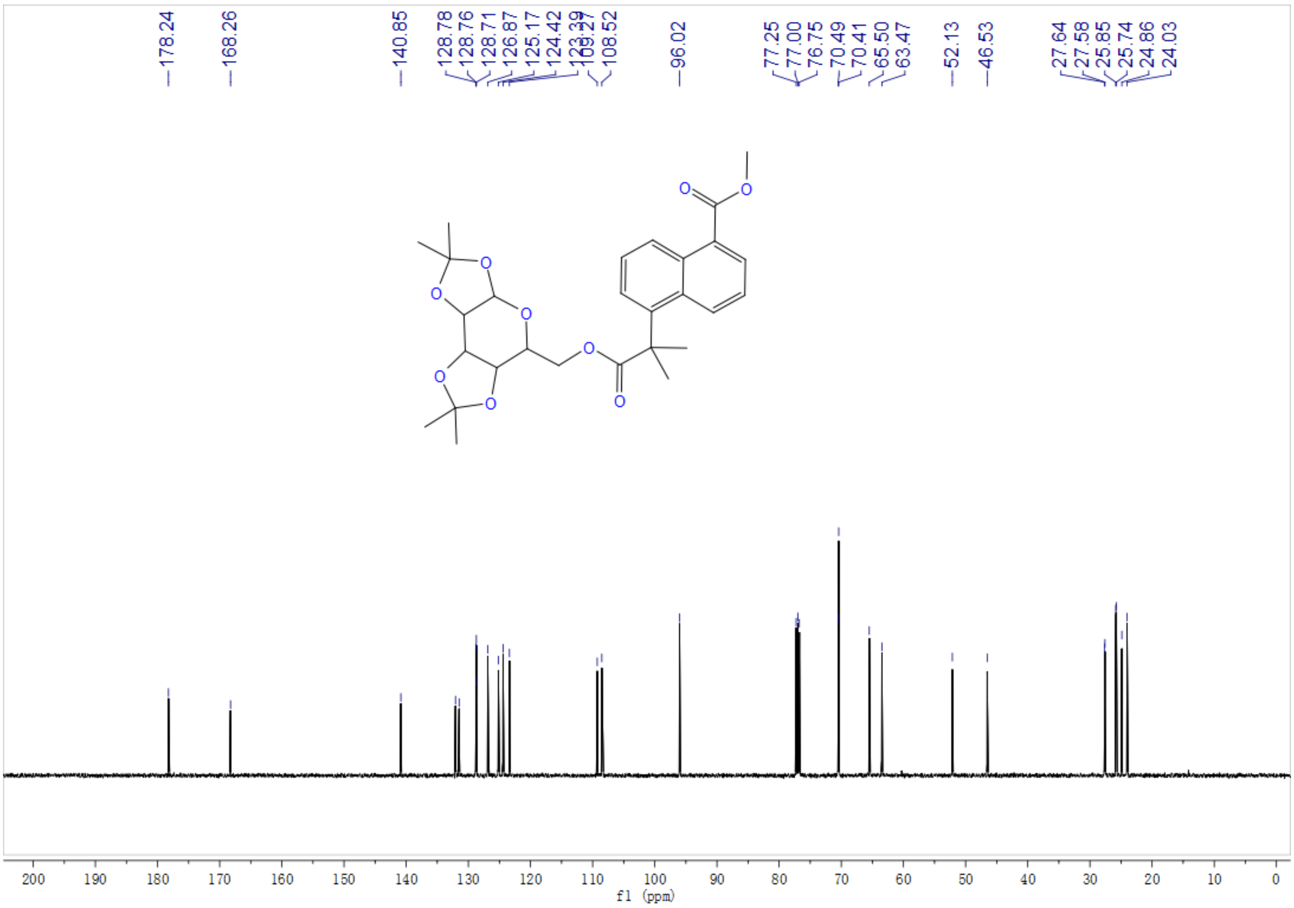

Methyl-5-(1-((1-(2,6-dimethylphenoxy)propan-2-yl)amino)-2-methyl-1-oxopropan-2-yl)-1-naphthoate, **4ra**, ^{1}H NMR (500 MHz, $CDCl_3$) and ^{13}C NMR (125 MHz, $CDCl_3$)

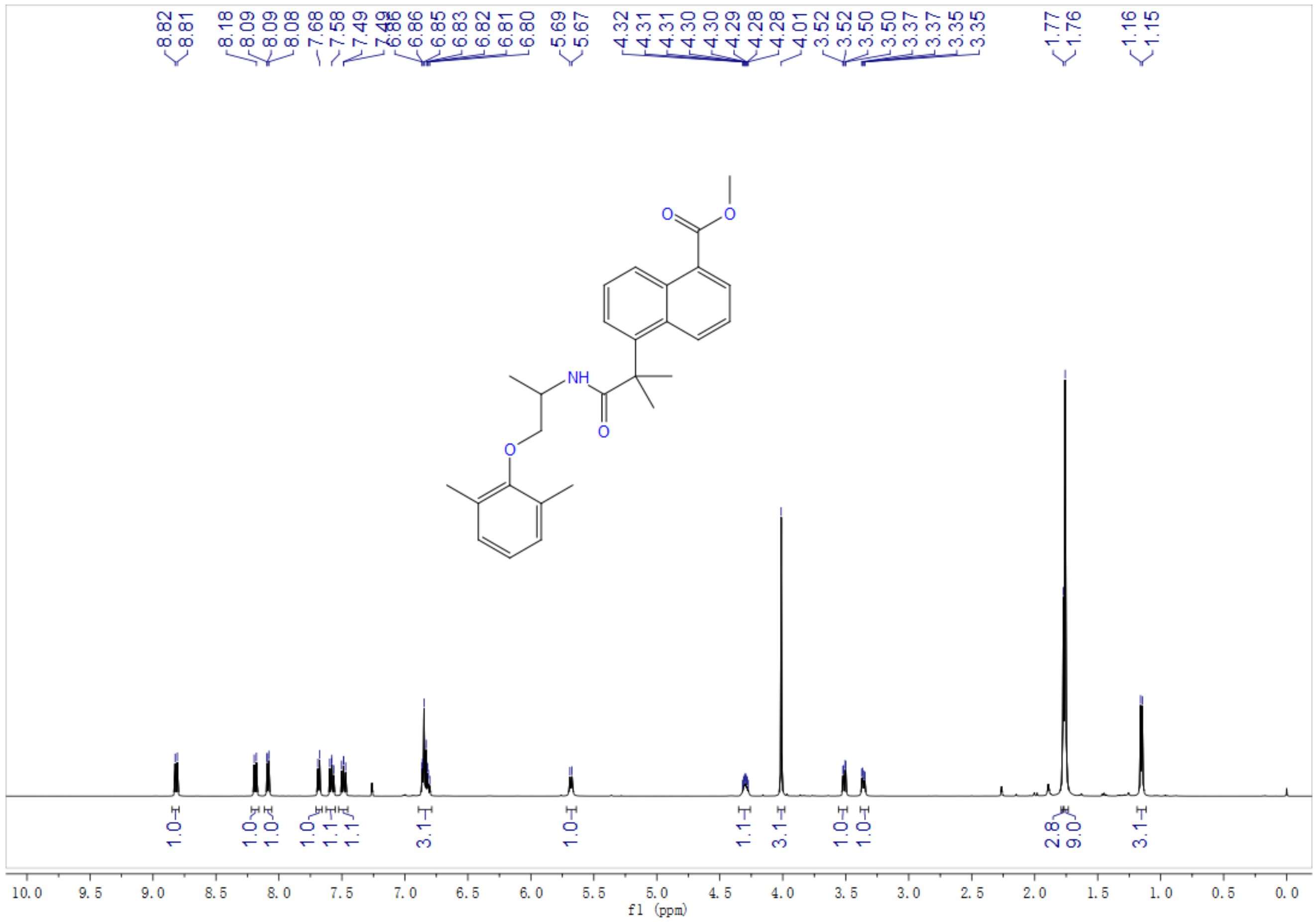

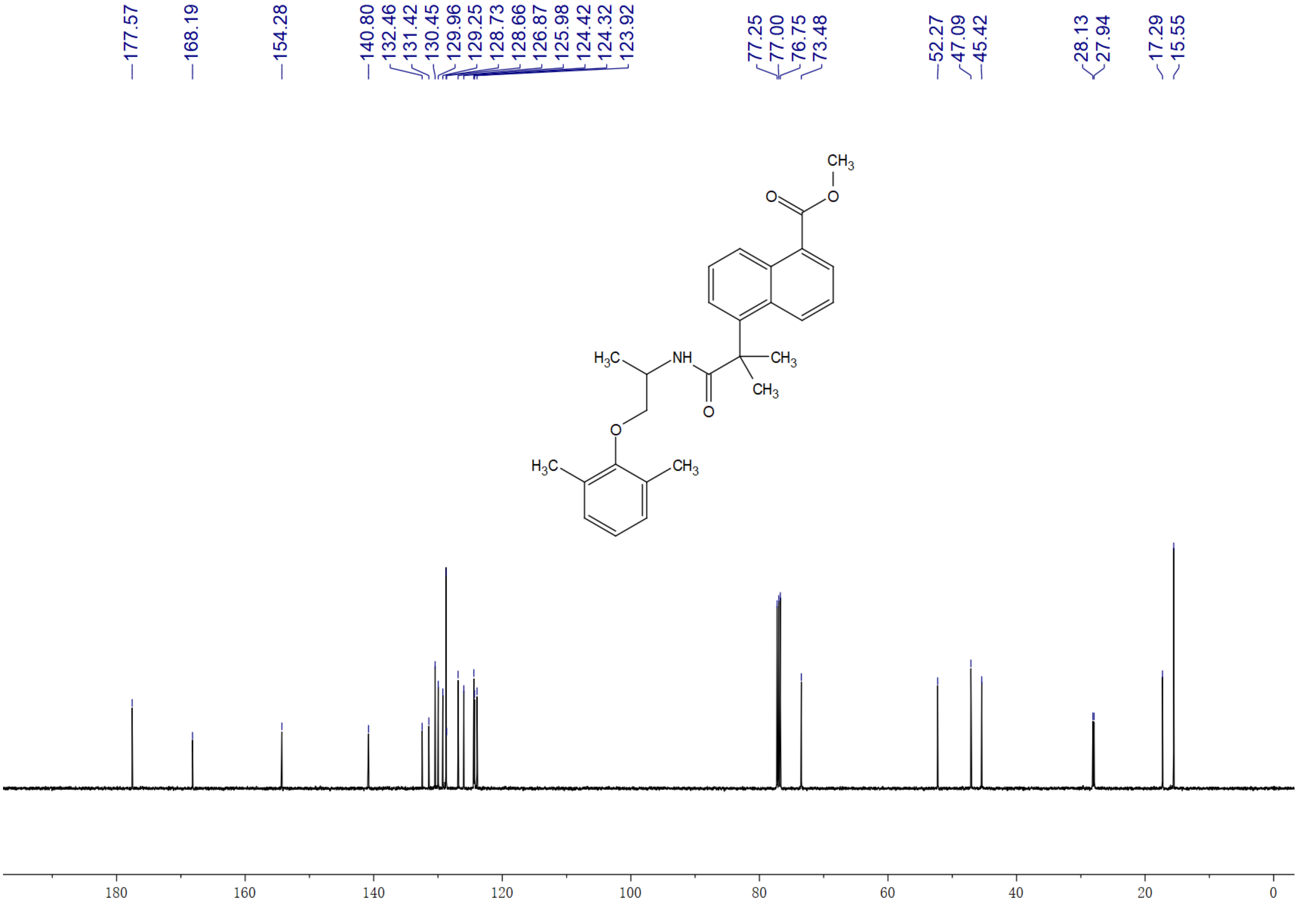

Methyl-5-(2-methyl-1-(((8S,9R,13R,14R)-13-methyl-17-oxo-7,8,9,11,12,13,14,15,16,17-decahydro-6H-cyclopenta[a]phenanthren-3-yl)oxy)-1-oxopropan-2-yl)-1-naphthoate, **4sa**, ^{1}H NMR (500 MHz, $CDCl_3$) and ^{13}C NMR (125 MHz, $CDCl_3$)

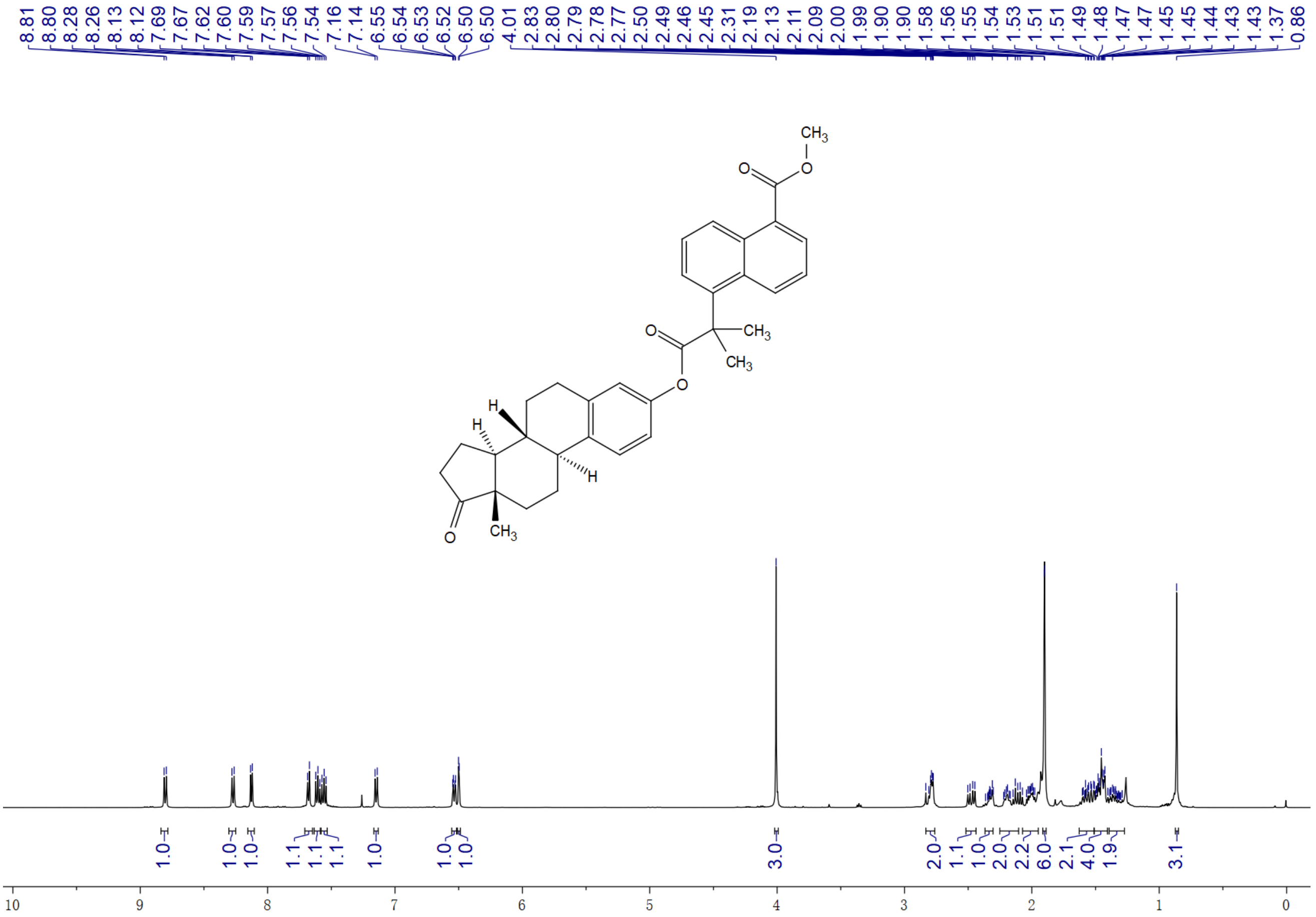

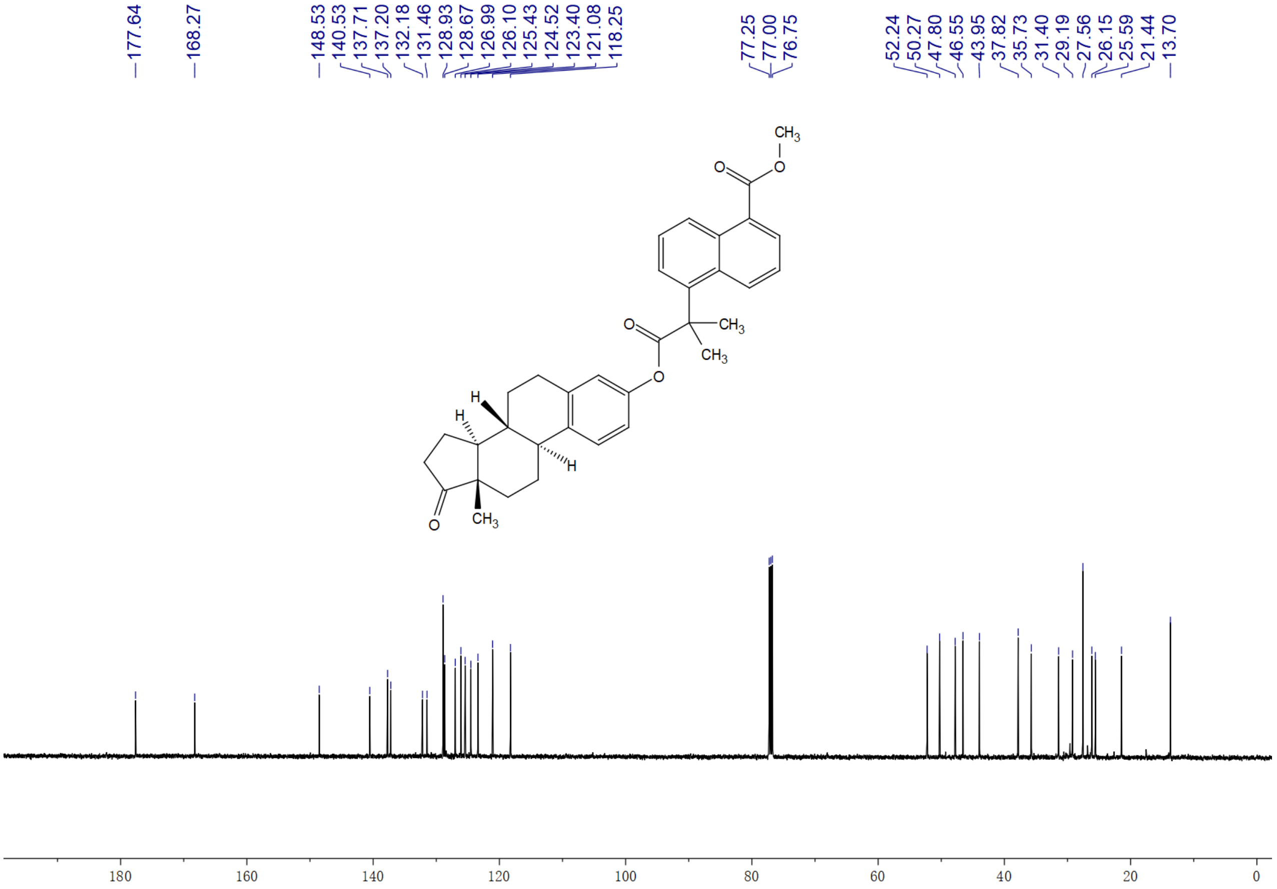

5-(1-(((3R,3aR,5aR,6aR,9S,11aS,11bS)-3a,6a-dimethyl-3-((R)-6-methylheptan-2-yl)-2,3,3a,4,5,5a,6,6a,7,8,9,10,11a,11b-tetradecahydro-1H-cyclopenta[a]anthracen-9-yl)oxy)-2-methyl-1-oxopropan-2-yl)-1-naphthoic acid, **4ta**, ^{1}H NMR (500 MHz, $CDCl_3$) and ^{13}C NMR (125 MHz, $CDCl_3$)

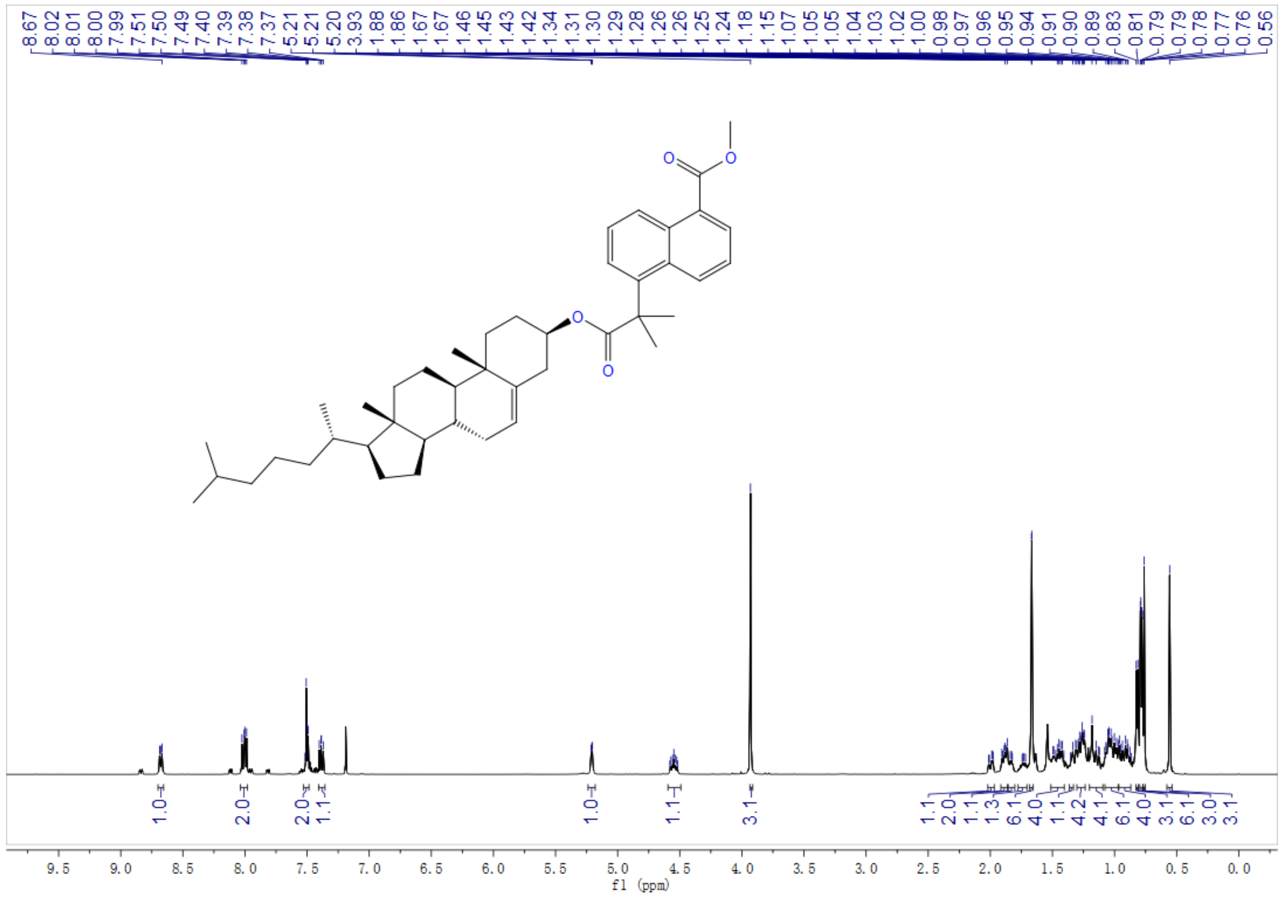

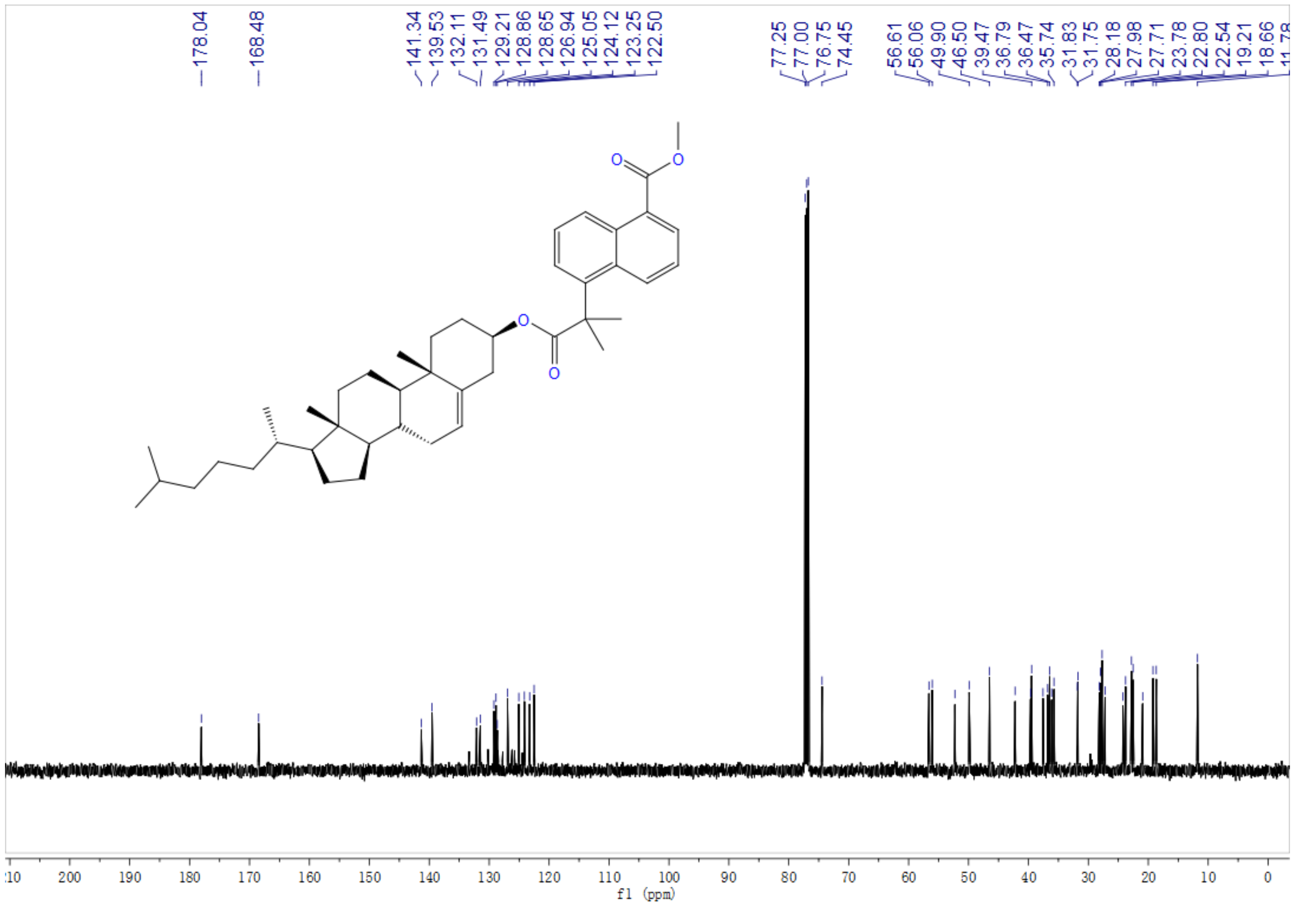

Methyl-5-(2-methyl-1-oxo-1-(((S)-2,5,7,8-tetramethyl-2-((4S,8S)-4,8,12-trimethyltridecyl)chroman-6-yl)oxy)propan-2-yl)-1-naphthoate, **4ua**, ^{1}H NMR (400 MHz, $CDCl_3$) and ^{13}C NMR (100 MHz, $CDCl_3$)

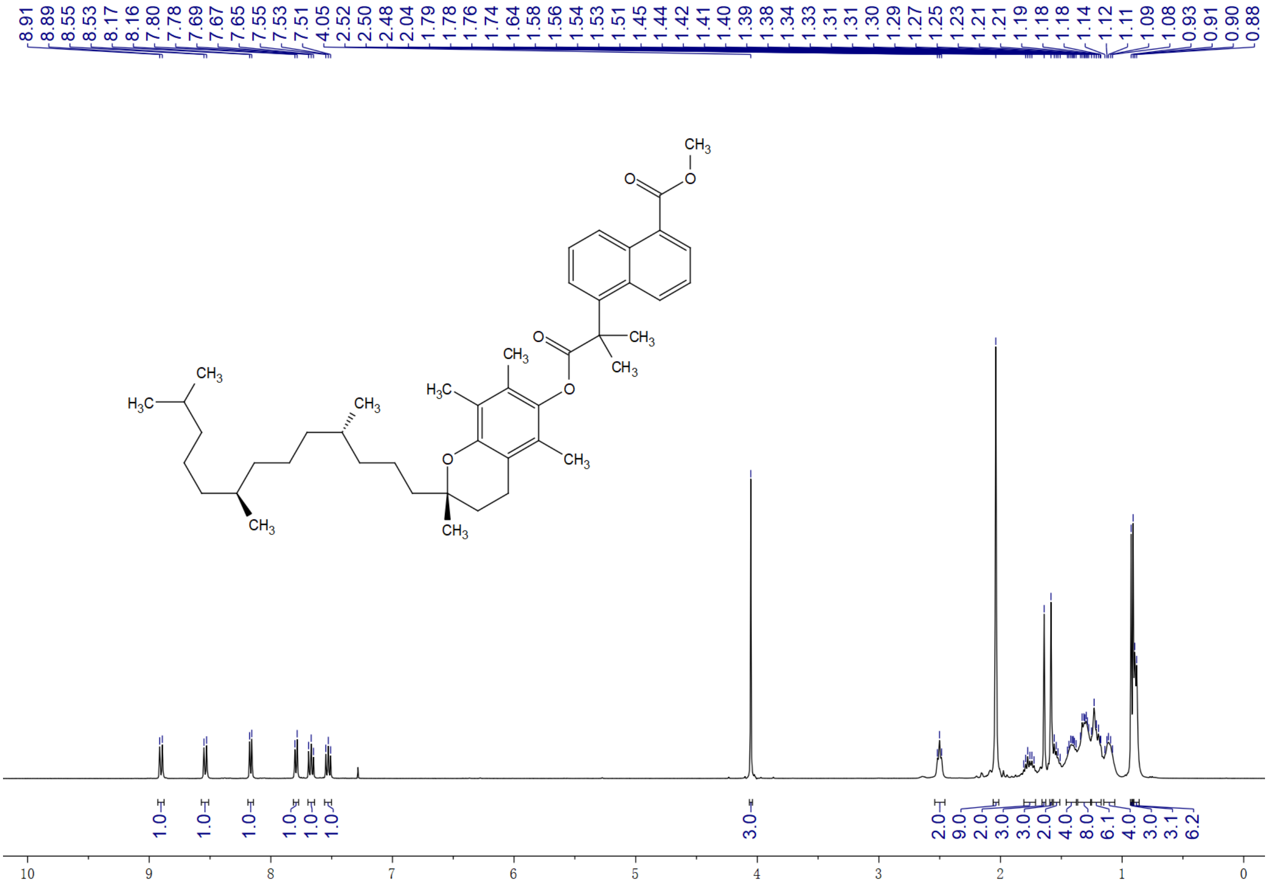

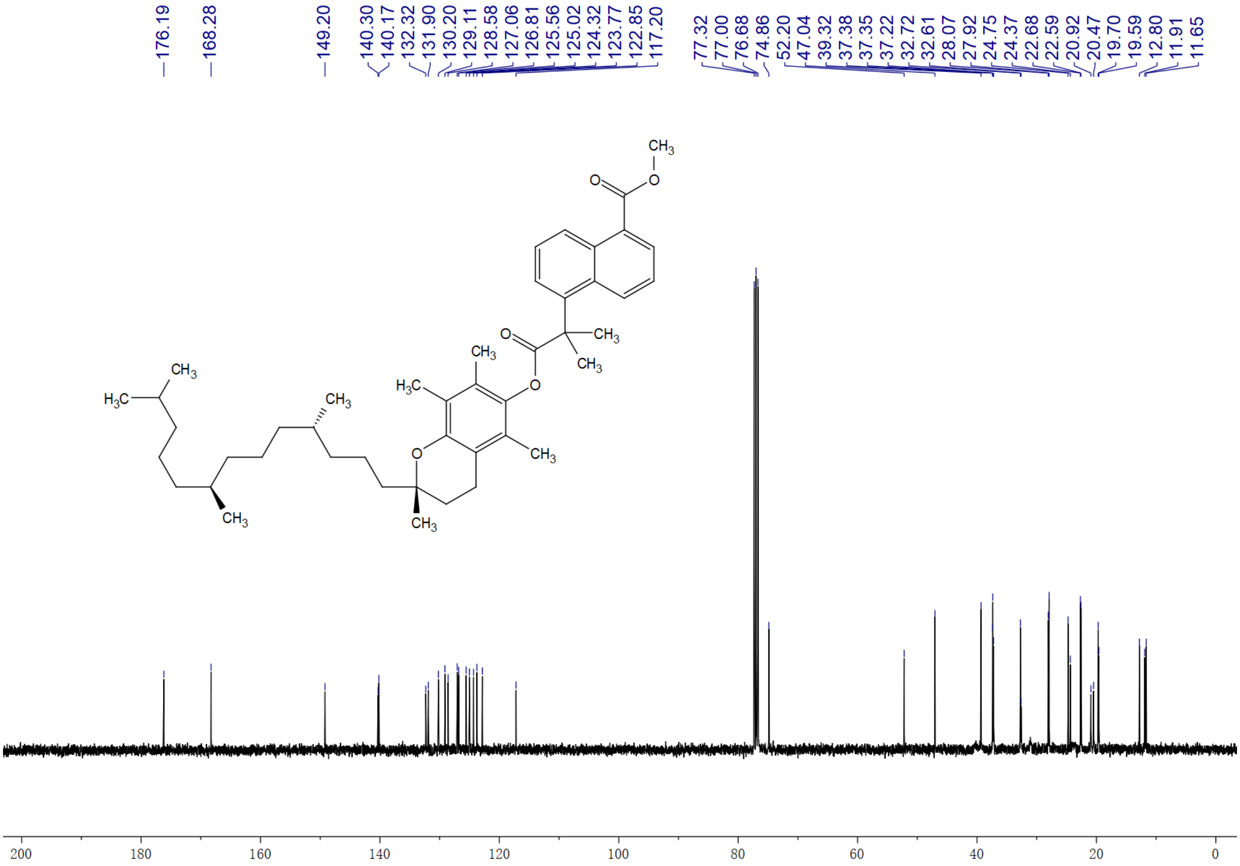

Methyl-3-methyl-4-(2-methyl-1-oxo-1-(phenylamino)propan-2-yl)-[1,1'-biphenyl]-2-carboxylate, **6a**, ^{1}H NMR (500 MHz, $CDCl_3$) and ^{13}C NMR (125 MHz, $CDCl_3$)

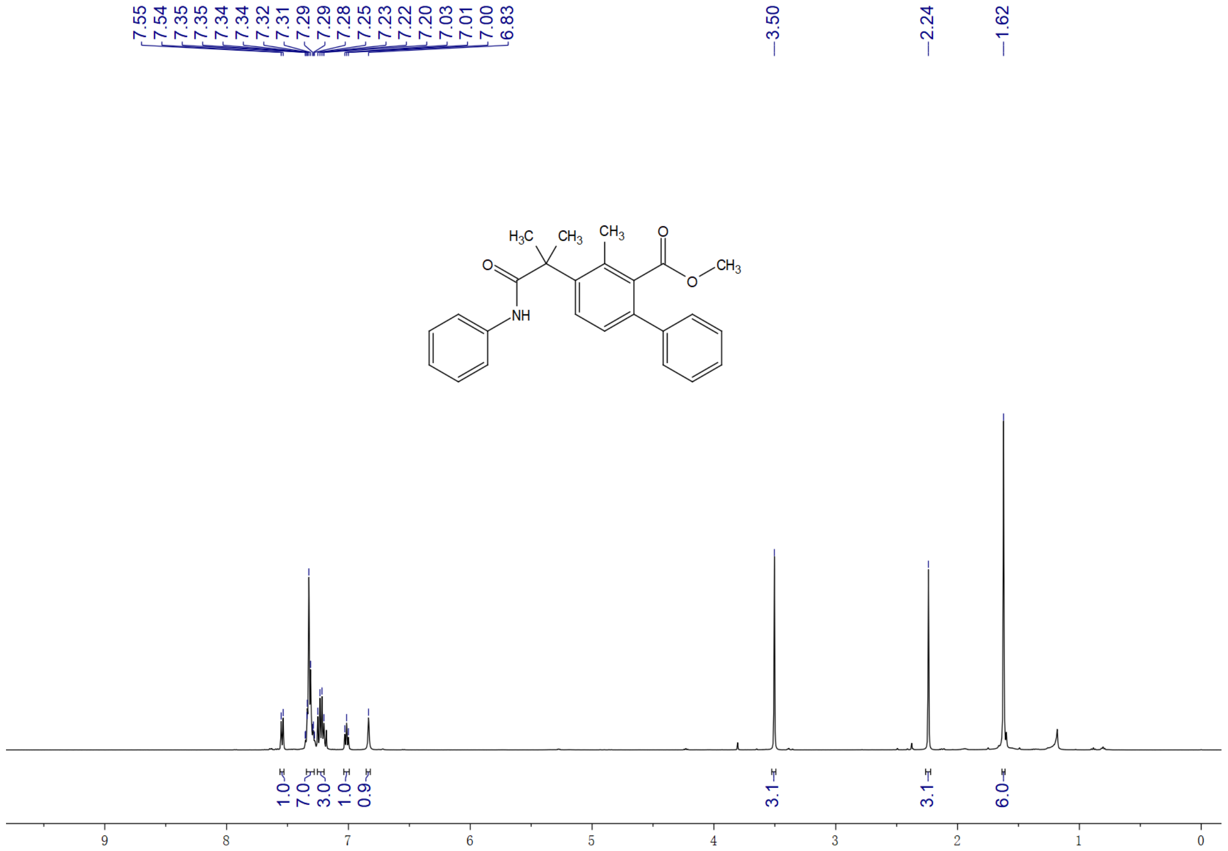

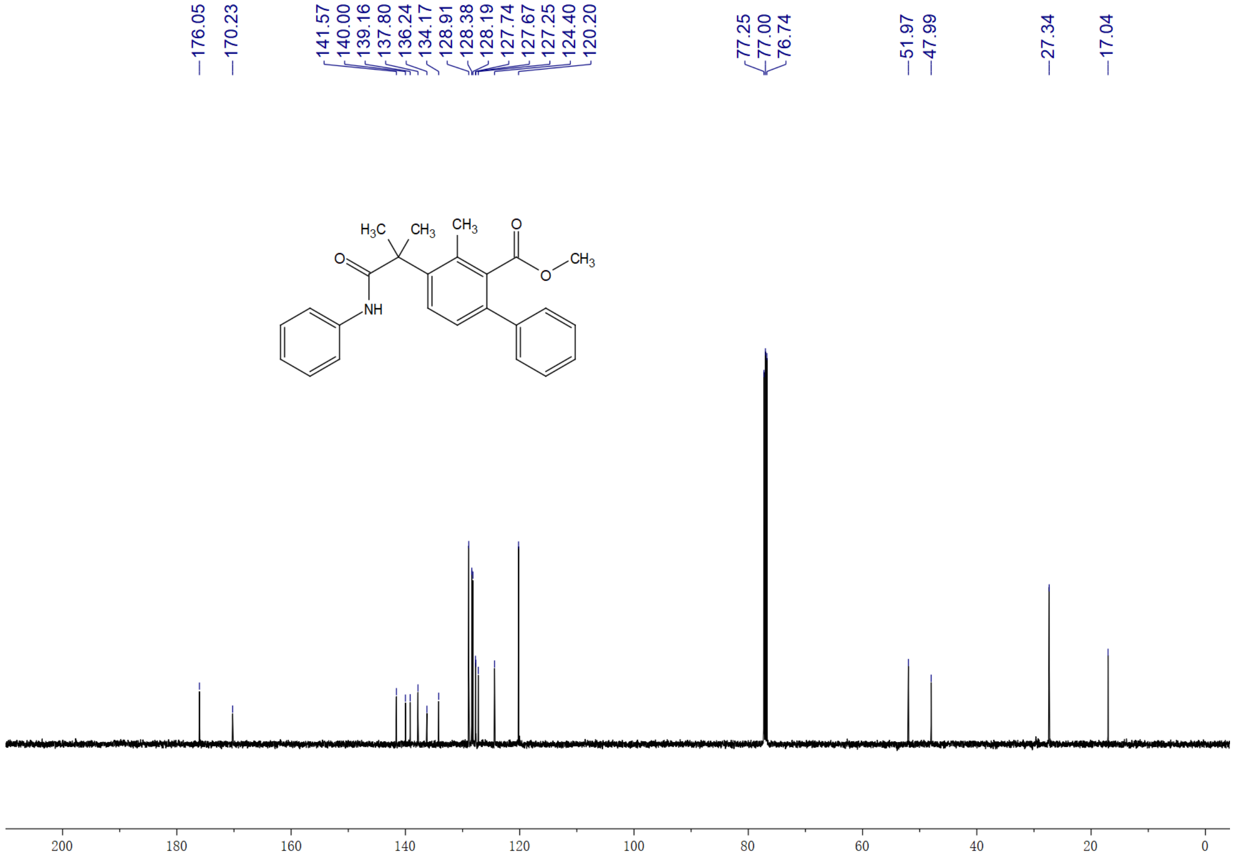

Ethyl-(*Z*)-2-(4-methyl-5-(2-methyl-1-oxo-1-(phenylamino)propan-2-yl)-3-oxoisobenzofuran-1(3H)-ylidene)acetate, **6b**, ^{1}H NMR (500 MHz, $CDCl_3$) and ^{13}C NMR (125 MHz, $CDCl_3$)

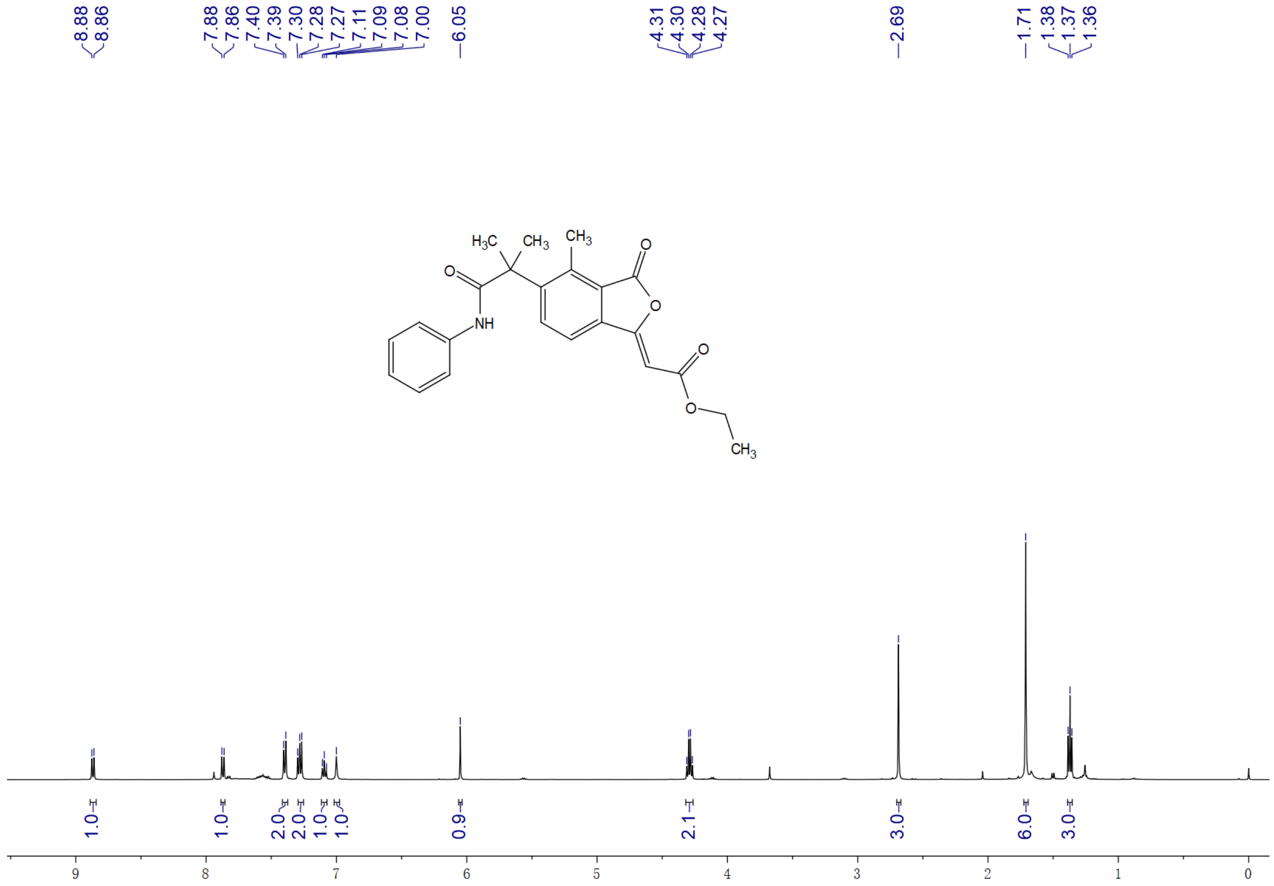

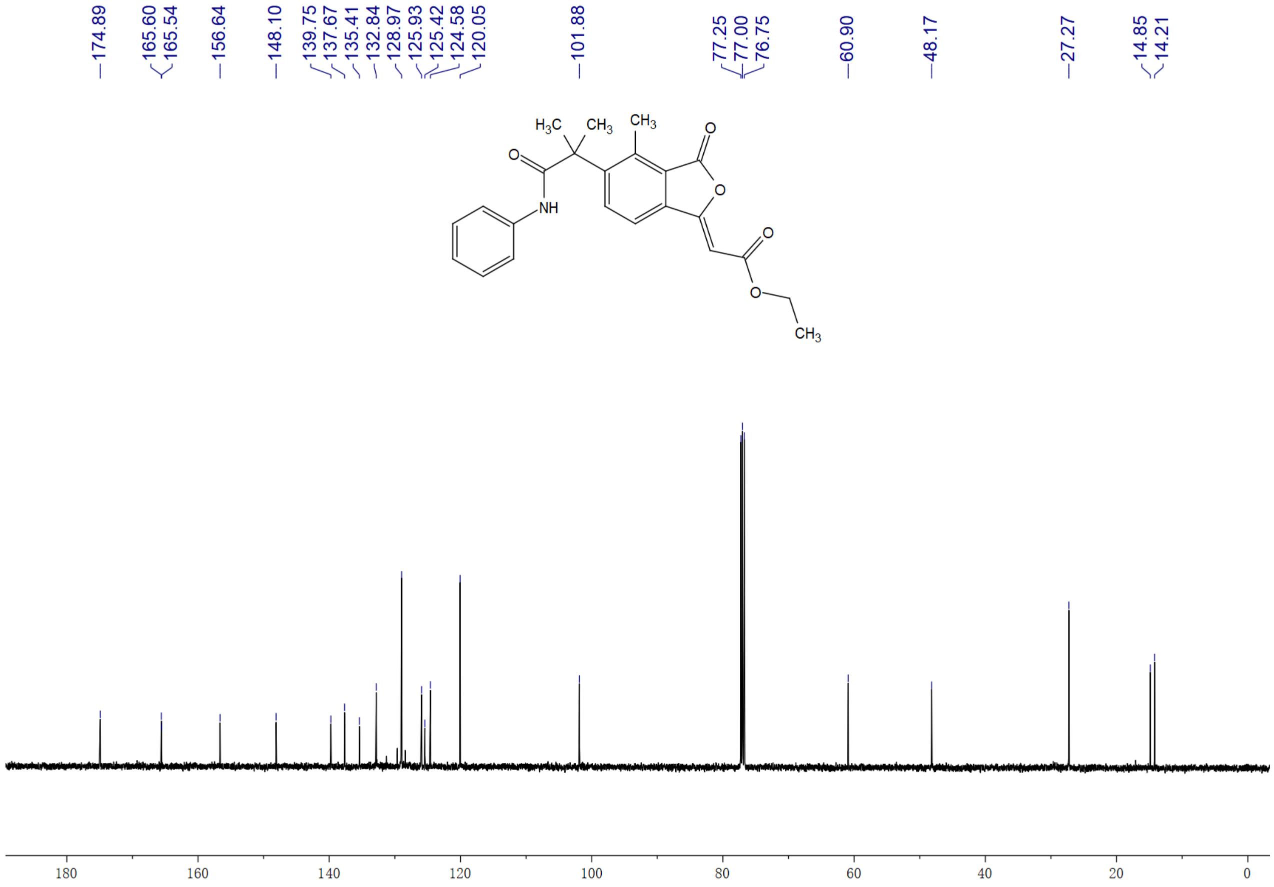

(*E*)-2-(4-(1,2-Diphenylvinyl)-2-methylphenyl)-2-methyl-N-phenylpropanamide, **6c**, ^{1}H NMR (500 MHz, $CDCl_3$) and ^{13}C NMR (125 MHz, $CDCl_3$)

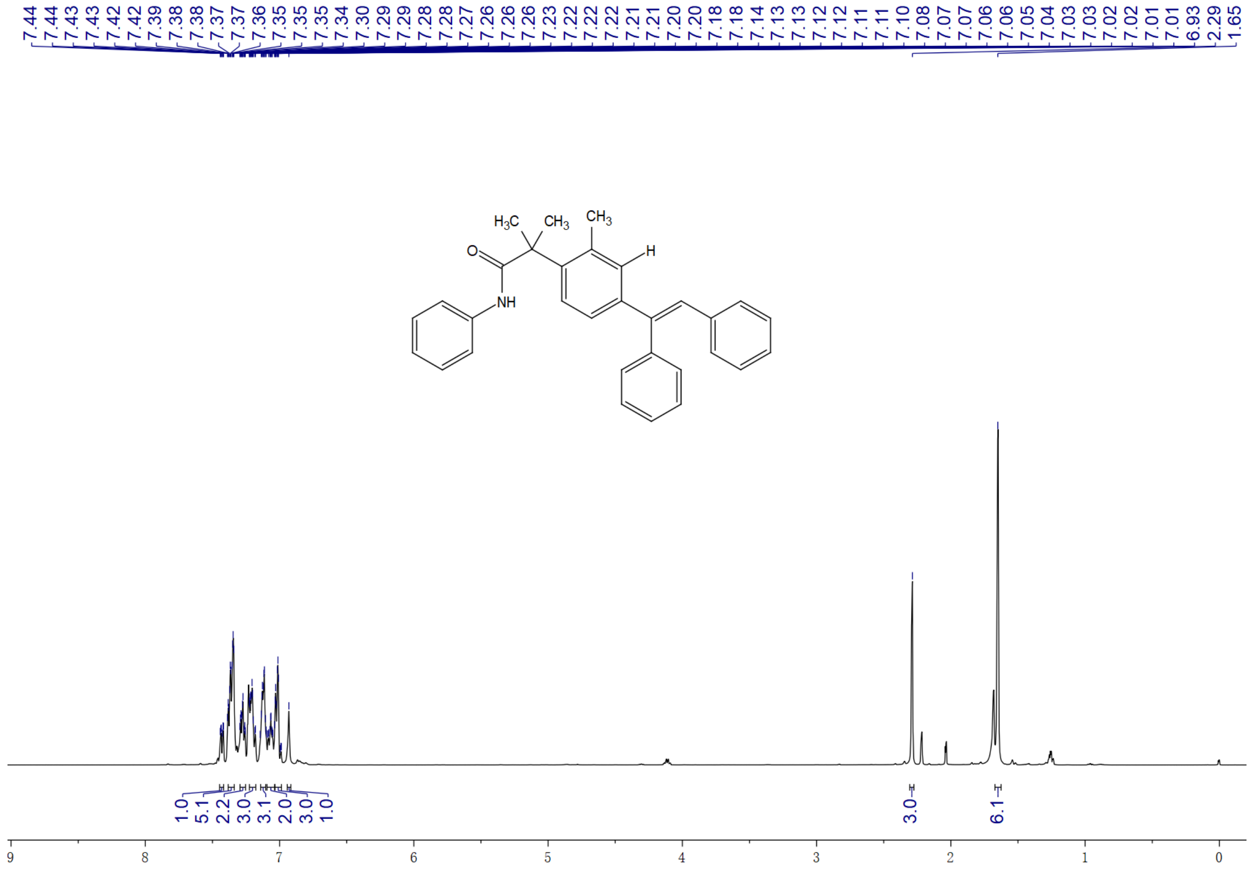

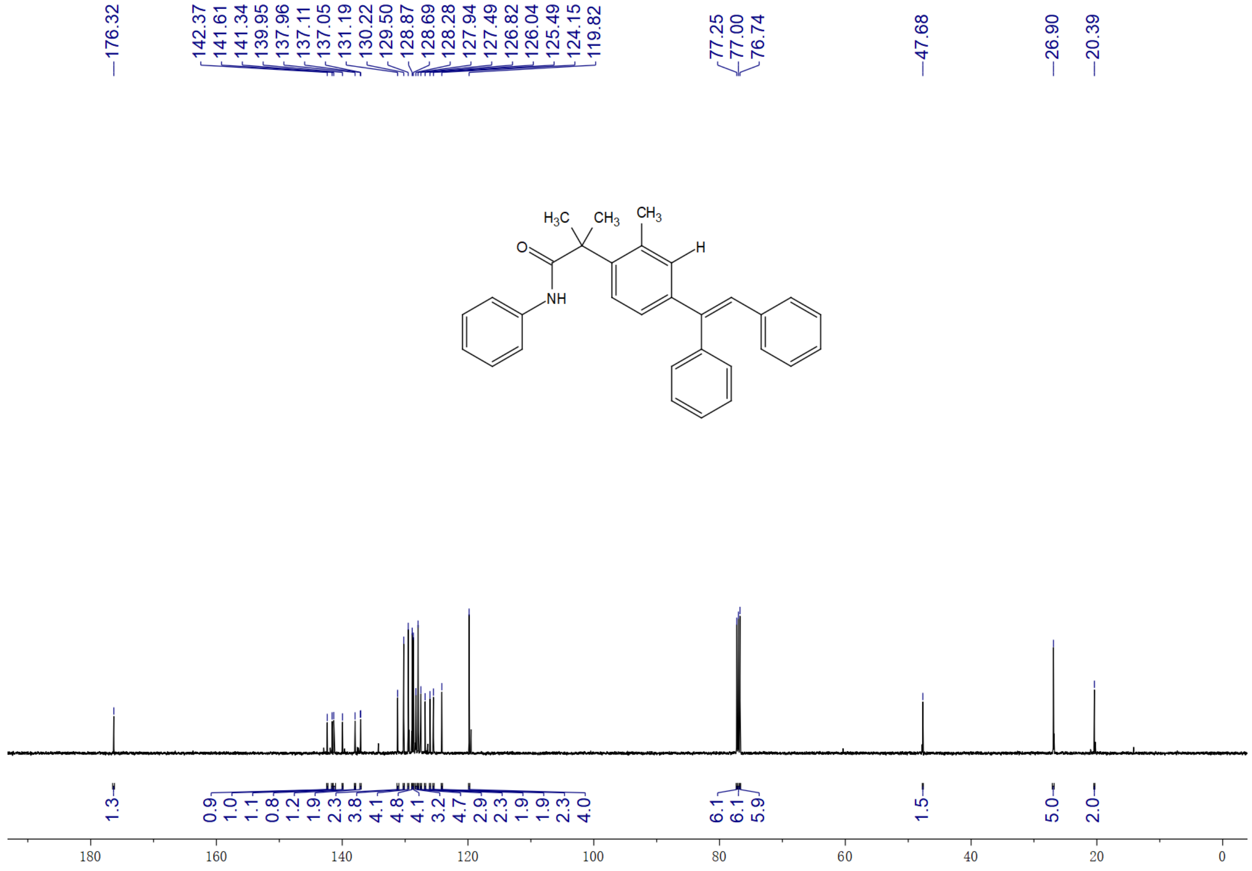

2-Methyl-*N*-phenyl-2-(o-tolyl)propenamide, **6d**, ^{1}H NMR (500 MHz, $CDCl_3$) and ^{13}C NMR (125 MHz, $CDCl_3$)

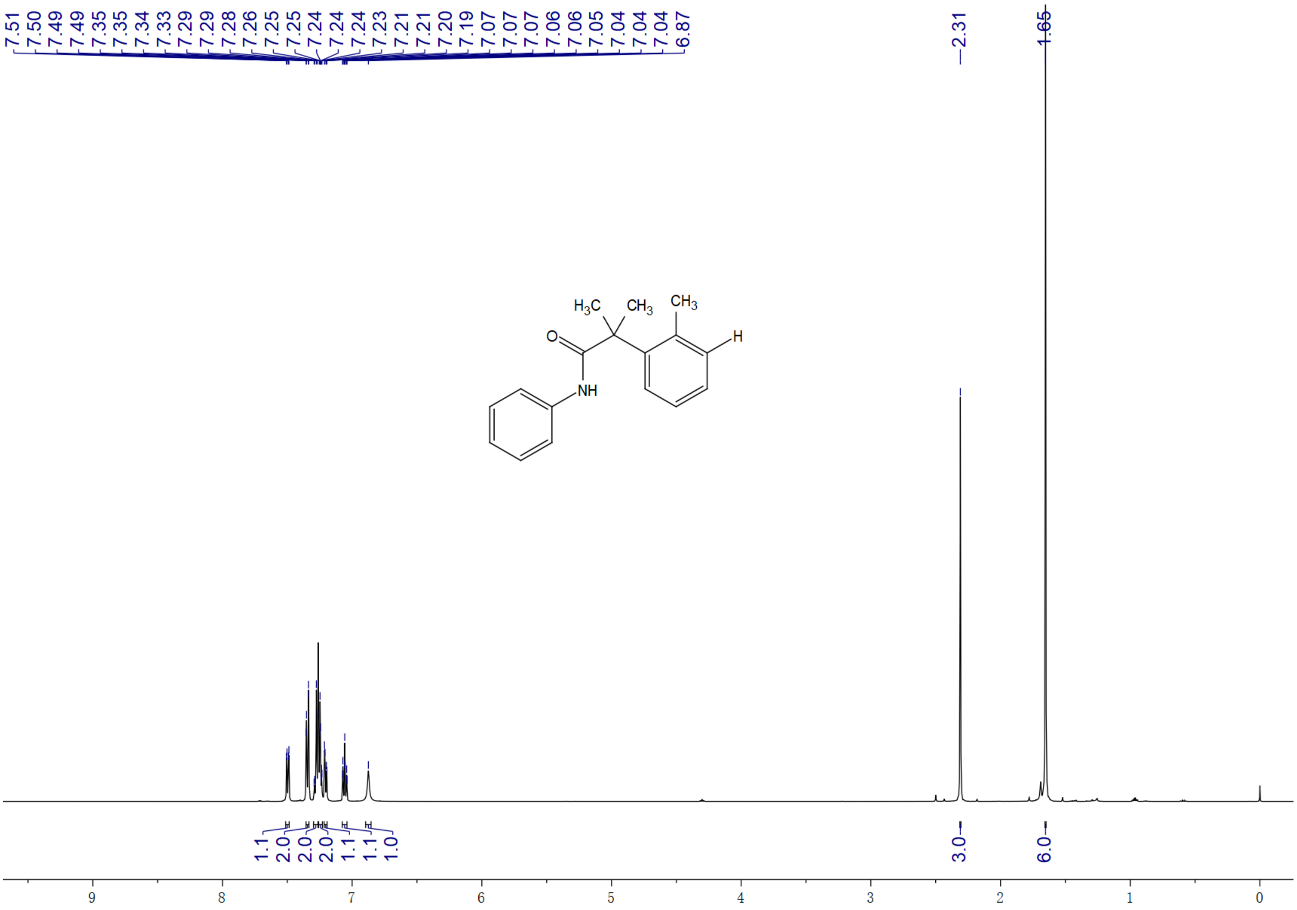

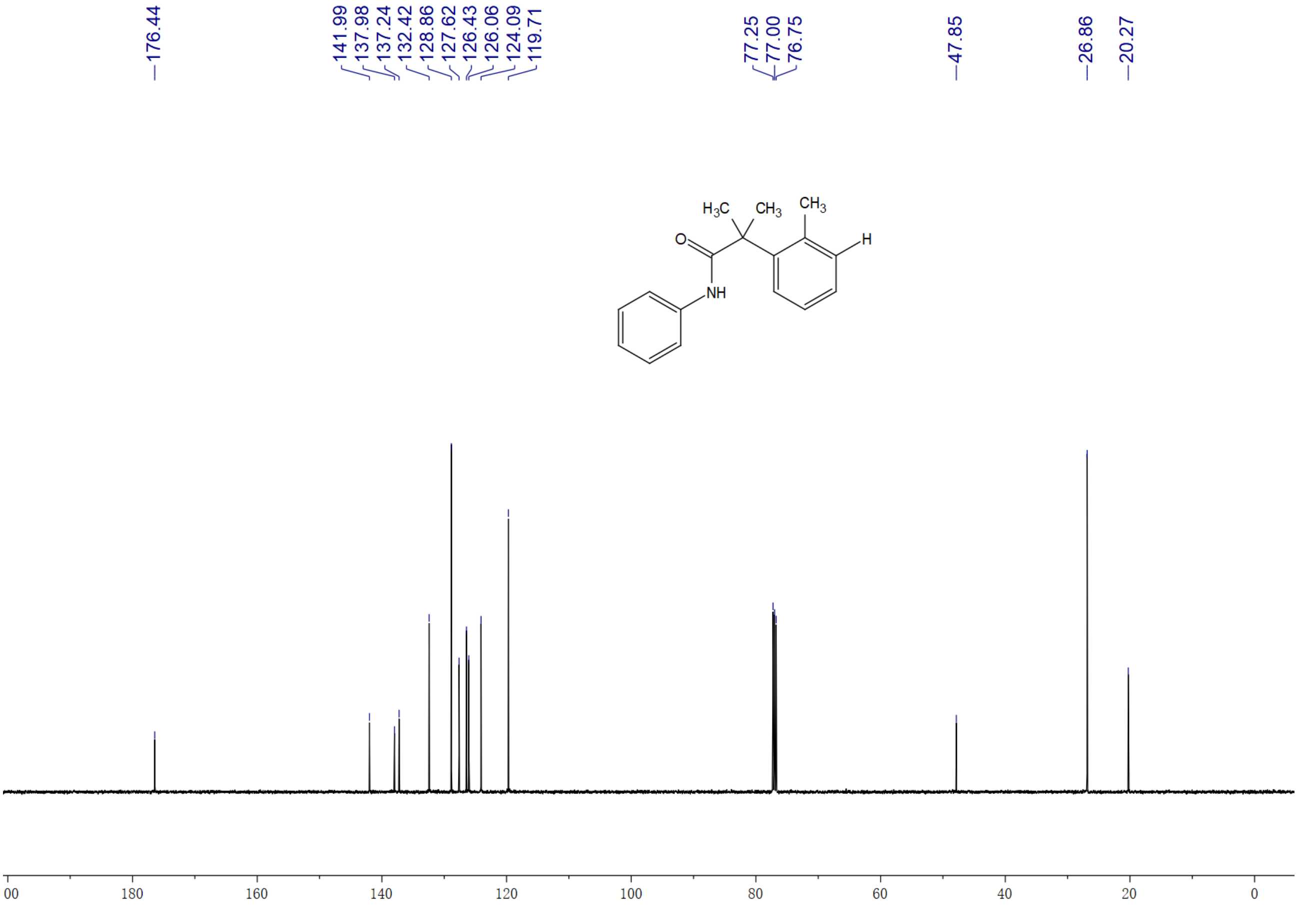

2-(3-Cyano-2-methylphenyl)-2-methyl-N-phenylpropanamide, **6e**, ^{1}H NMR (500 MHz, $CDCl_3$) and ^{13}C NMR (125 MHz, $CDCl_3$)

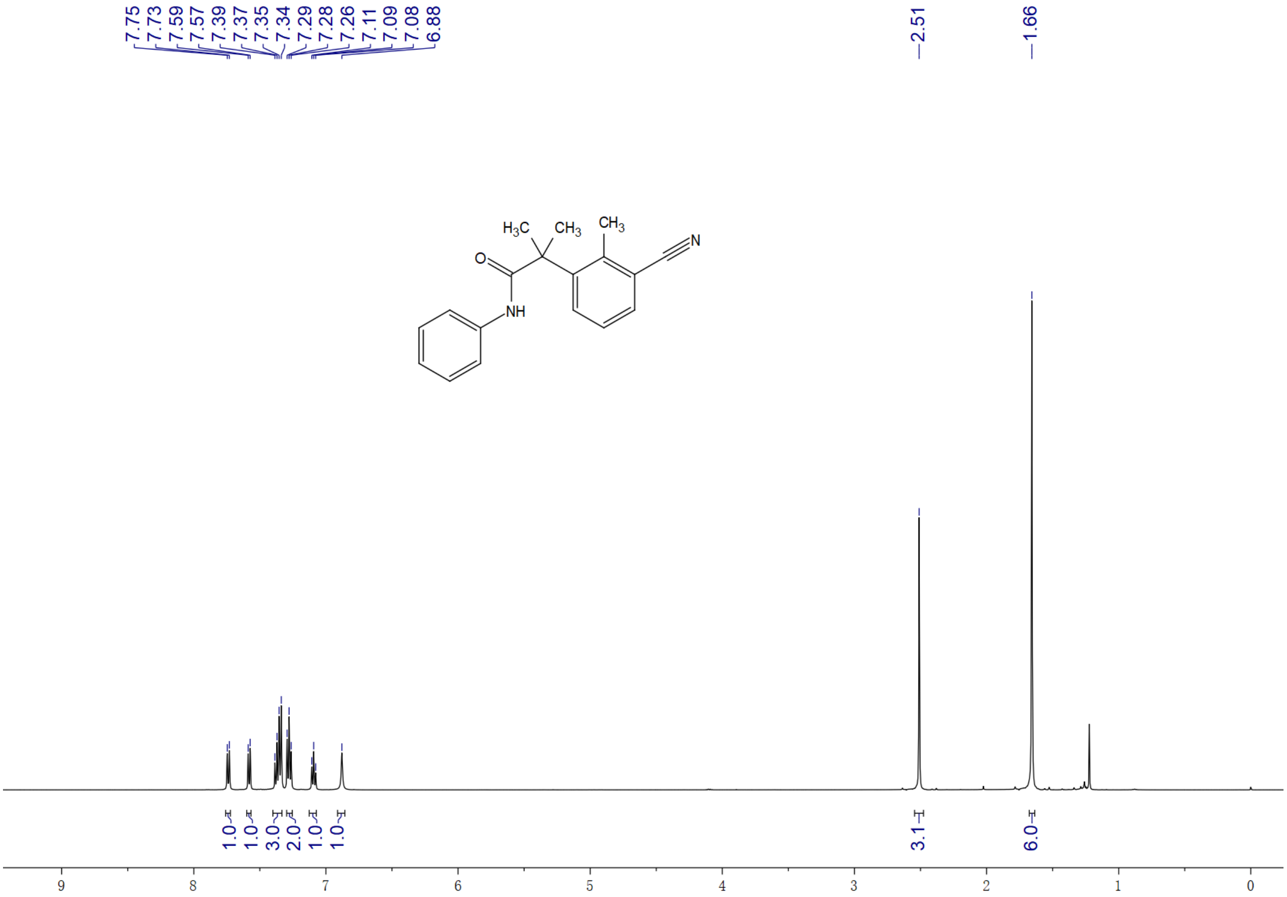

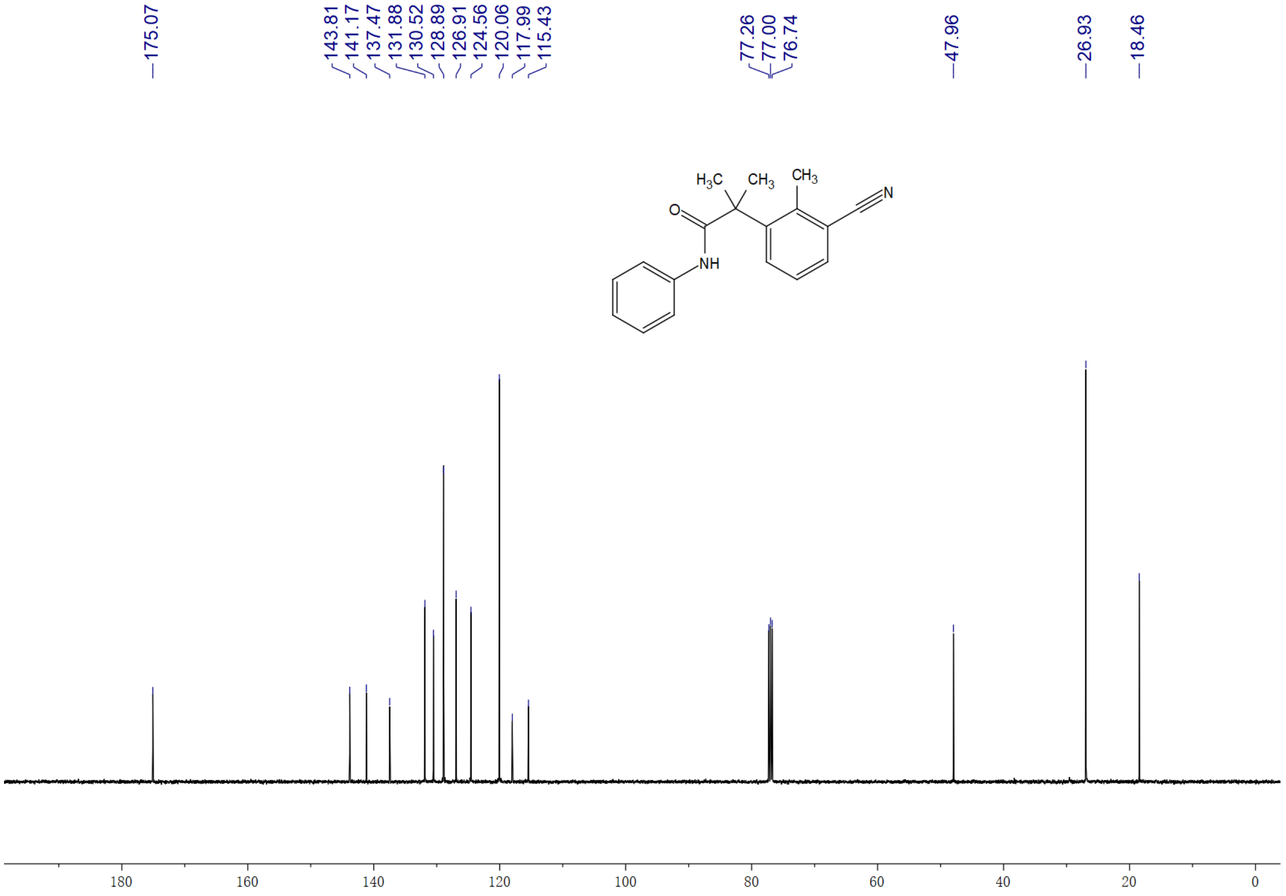

2-(3-(Hydroxymethyl)-2-methylphenyl)-2-methyl-N-phenylpropanamide, **6f**, ^{1}H NMR (500 MHz, $CDCl_3$) and ^{13}C NMR (125 MHz, $CDCl_3$)

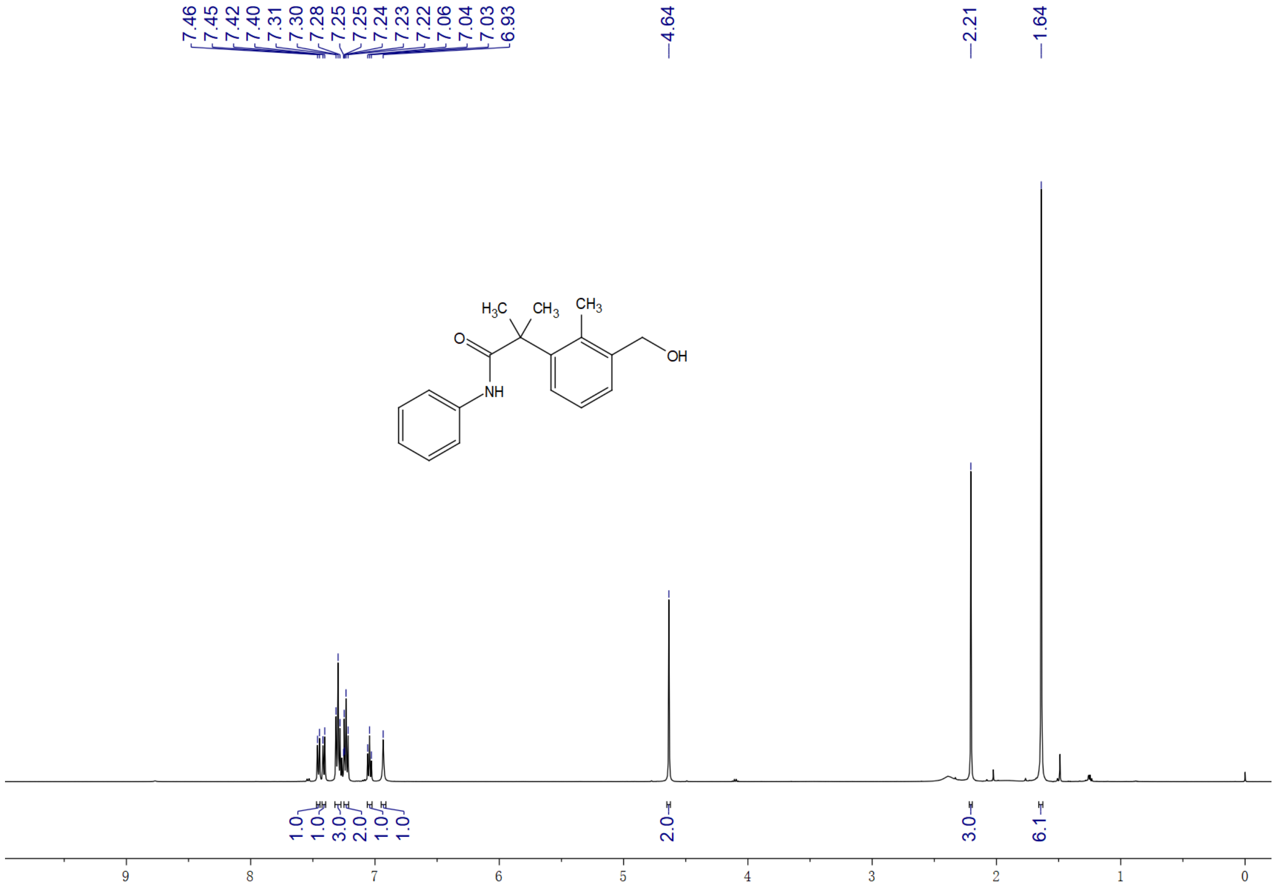

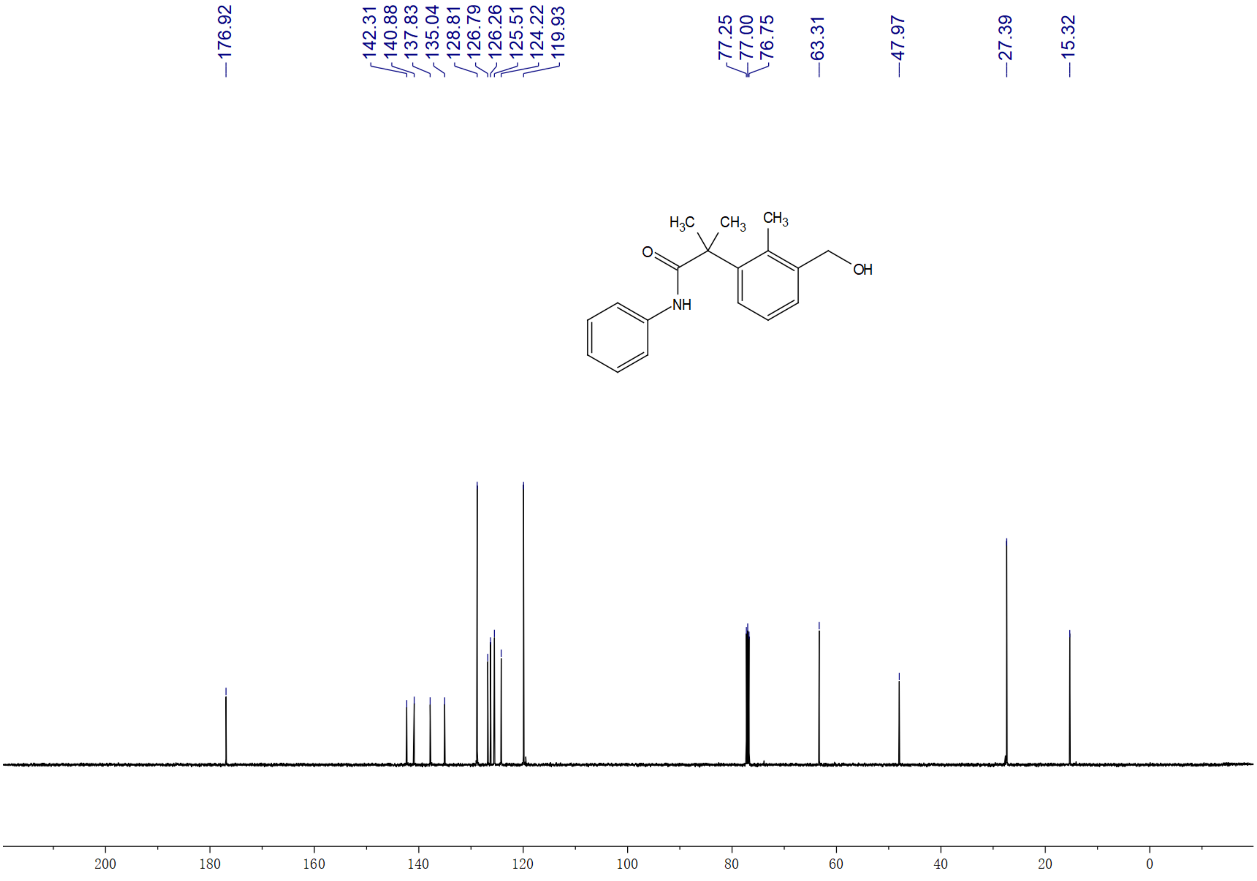

2-(3-Formyl-2-methylphenyl)-2-methyl-N-phenylpropanamide, **6g**, ^{1}H NMR (500 MHz, $CDCl_3$) and ^{13}C NMR (125 MHz, $CDCl_3$)

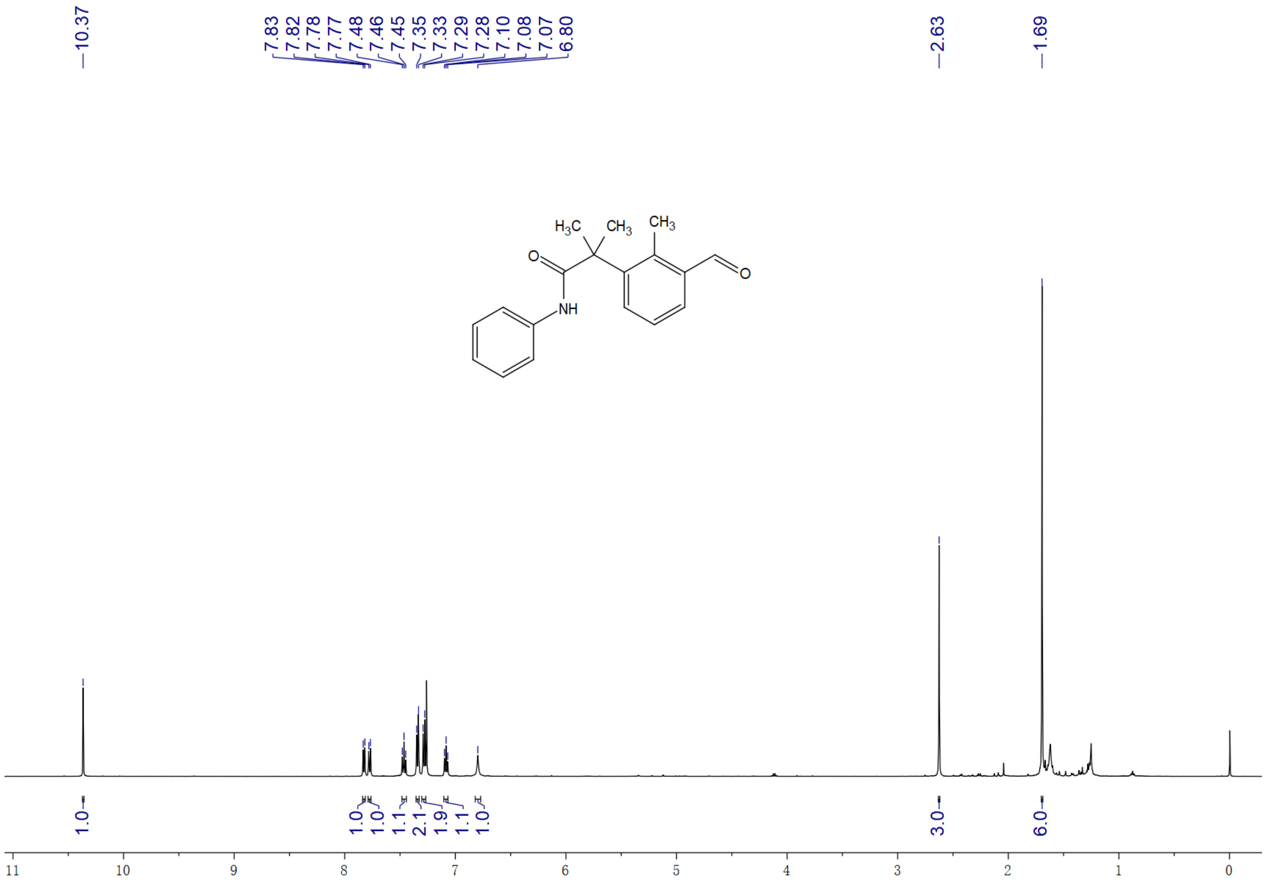

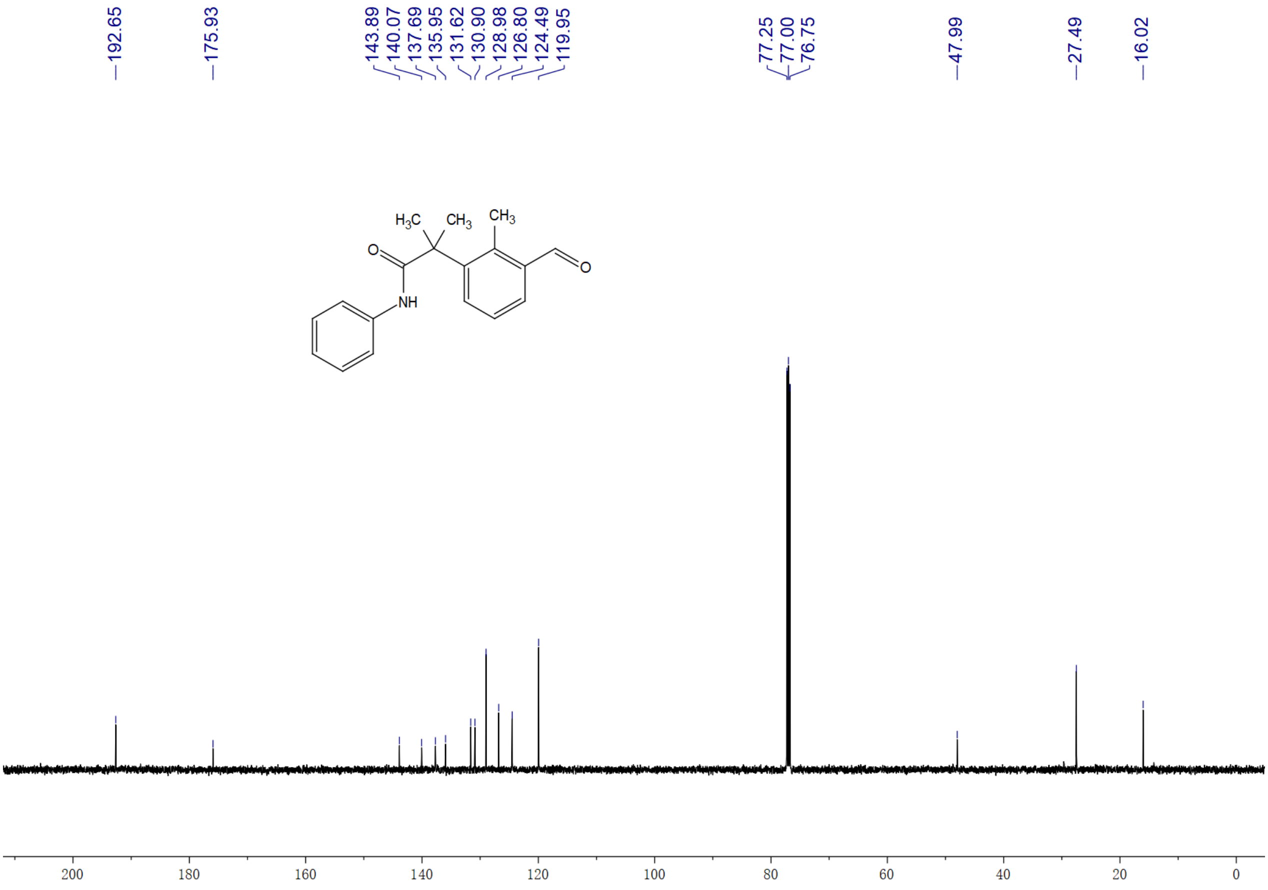

Difluoromethyl 2-methyl-3-(2-methyl-1-oxo-1-(phenylamino)propan-2-yl)benzoate, **6h**, ^{1}H NMR (500 MHz, $CDCl_3$) and ^{13}C NMR (125 MHz, $CDCl_3$)

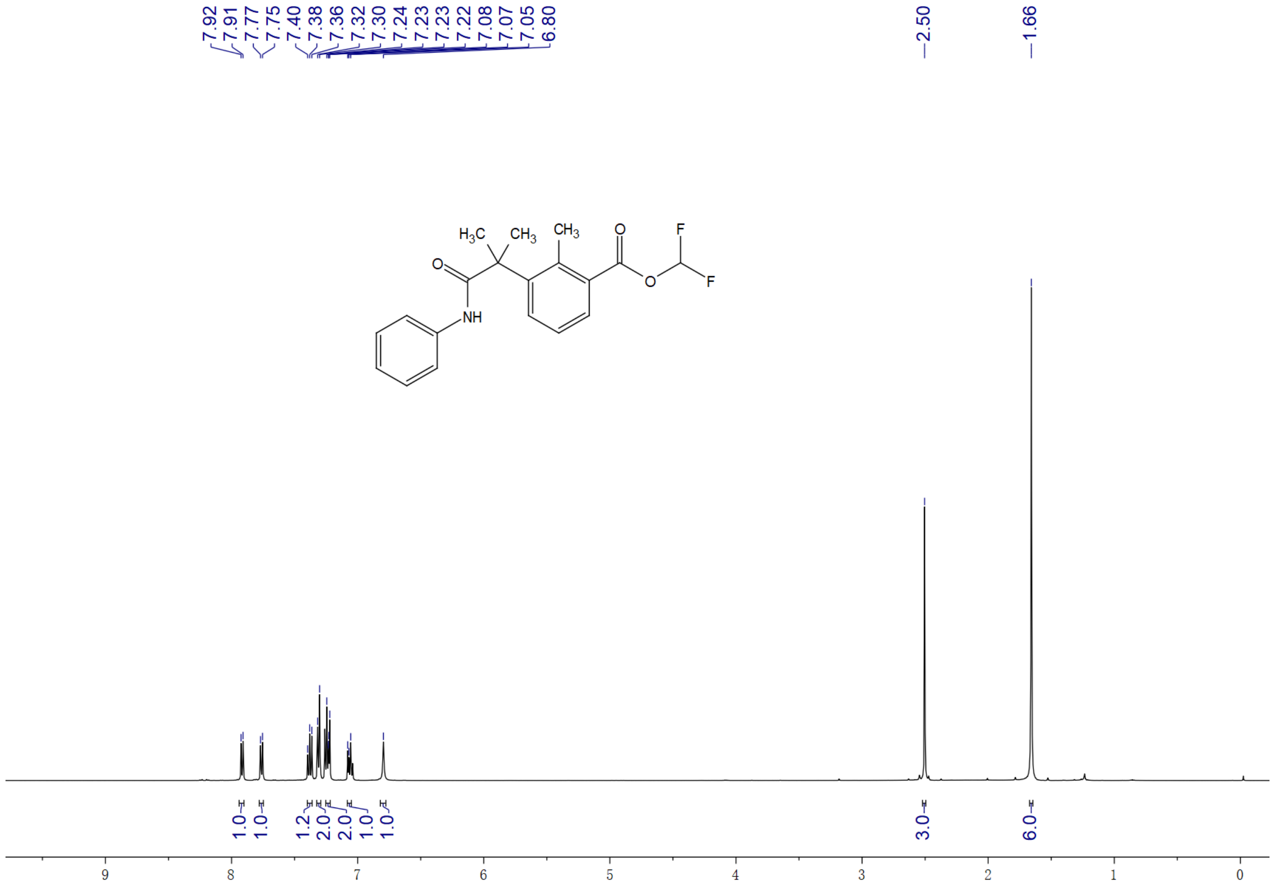

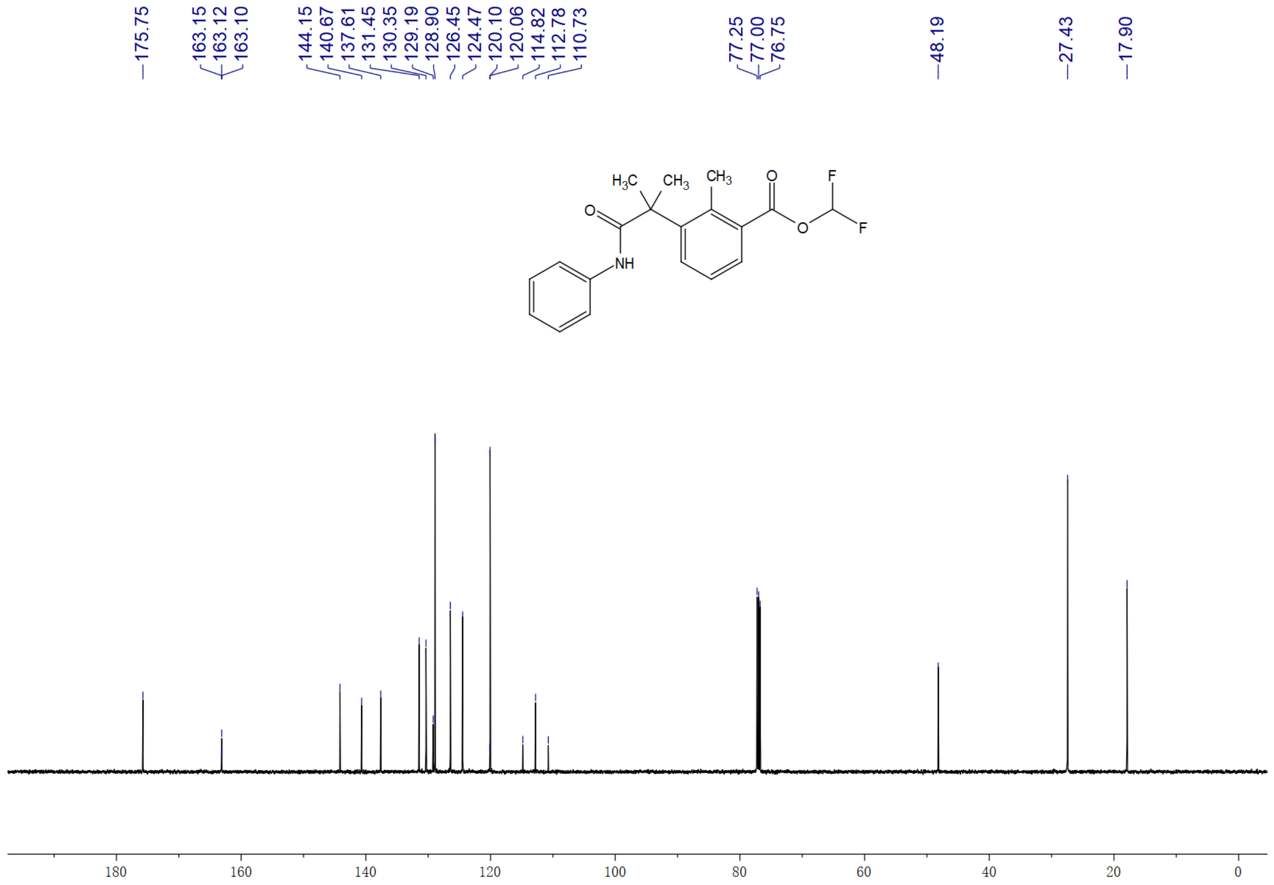

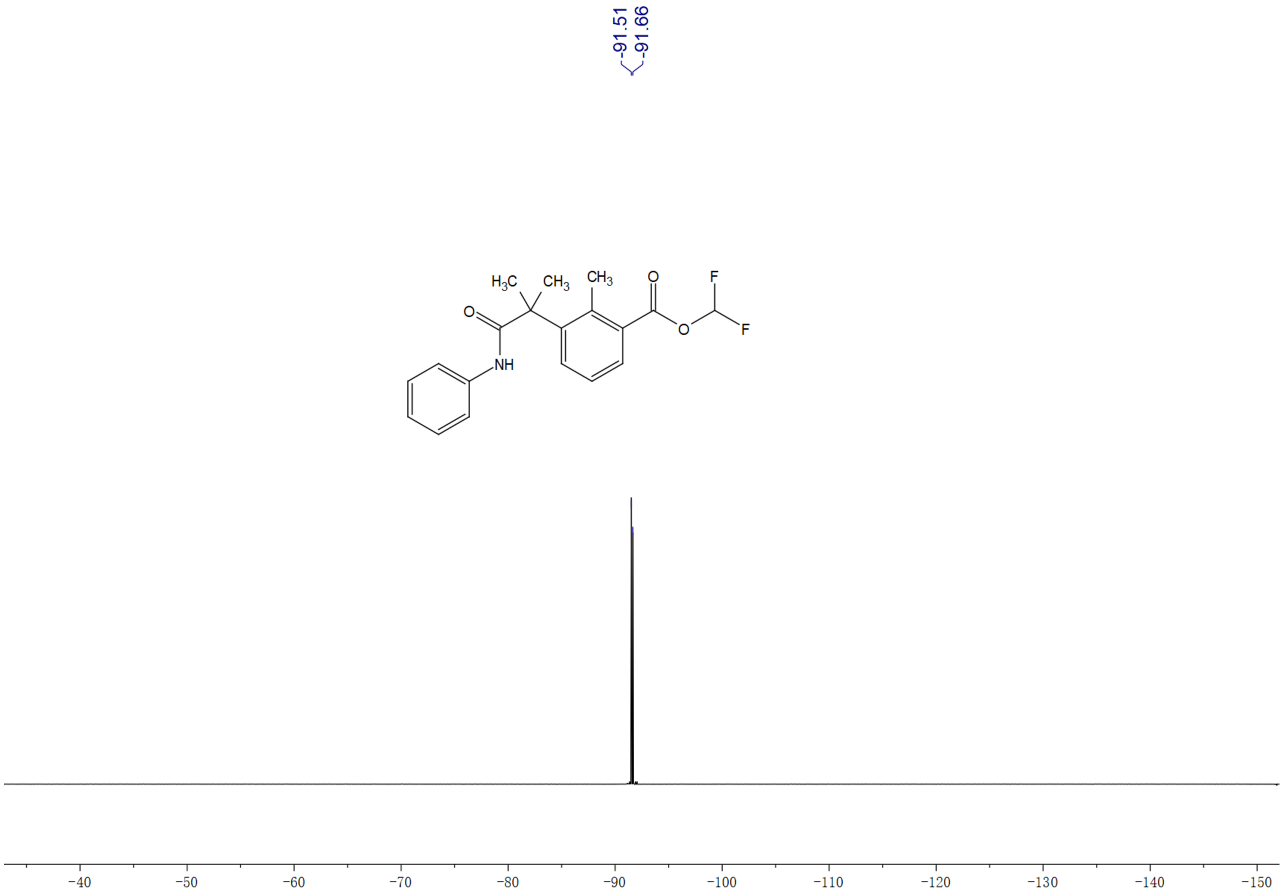
-91.51
-91.66
H3C
CH3
CH3
O
O
O
F
F
NH
-40
-50
-60
-70
-80
-90
-100
-110
-120
-130
-140
-150

2-(3-Benzoyl-2-methylphenyl)-2-methyl-N-phenylpropanamide, **6i**, ^{1}H NMR (500 MHz, $CDCl_3$) and ^{13}C NMR (125 MHz, $CDCl_3$)

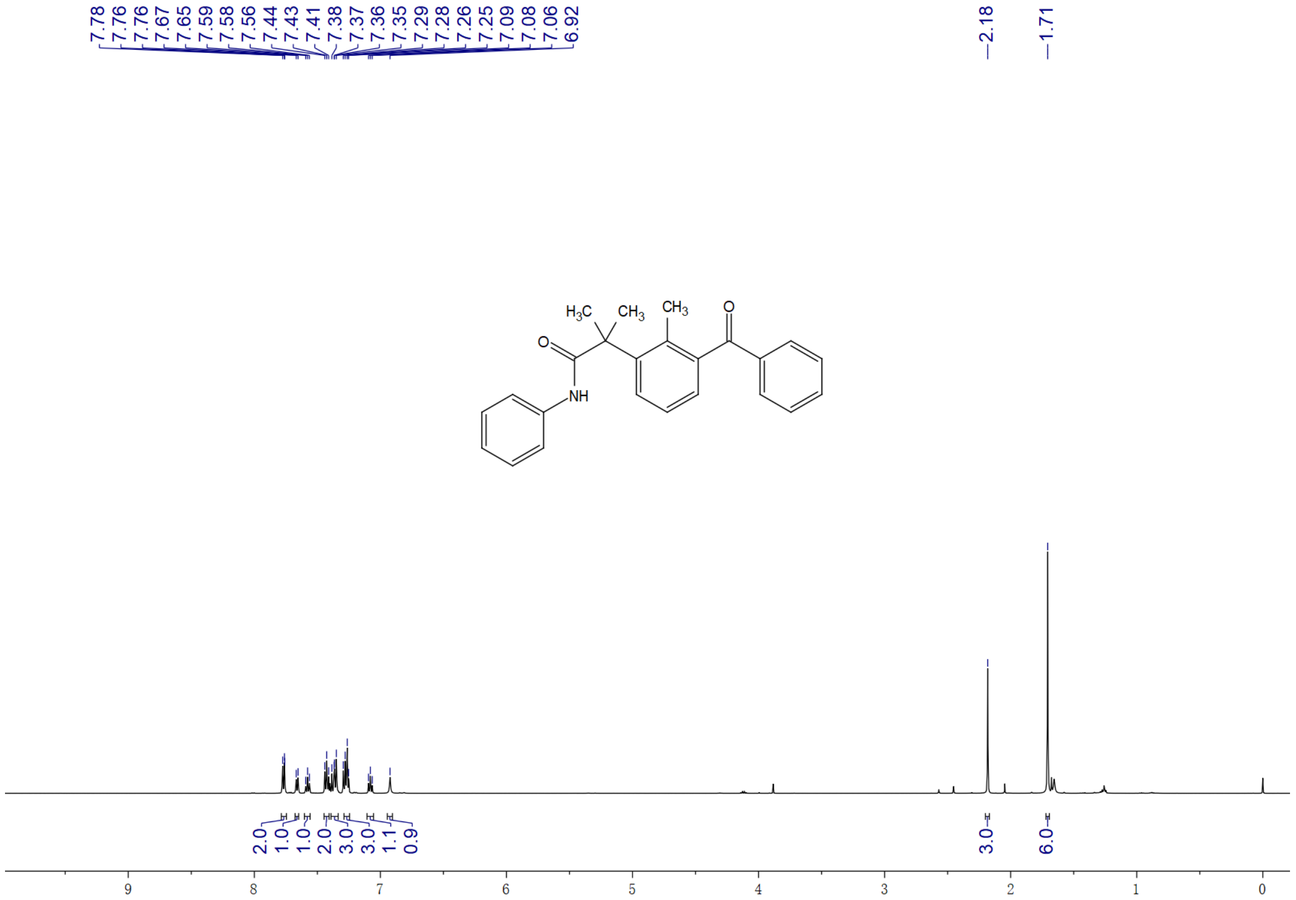

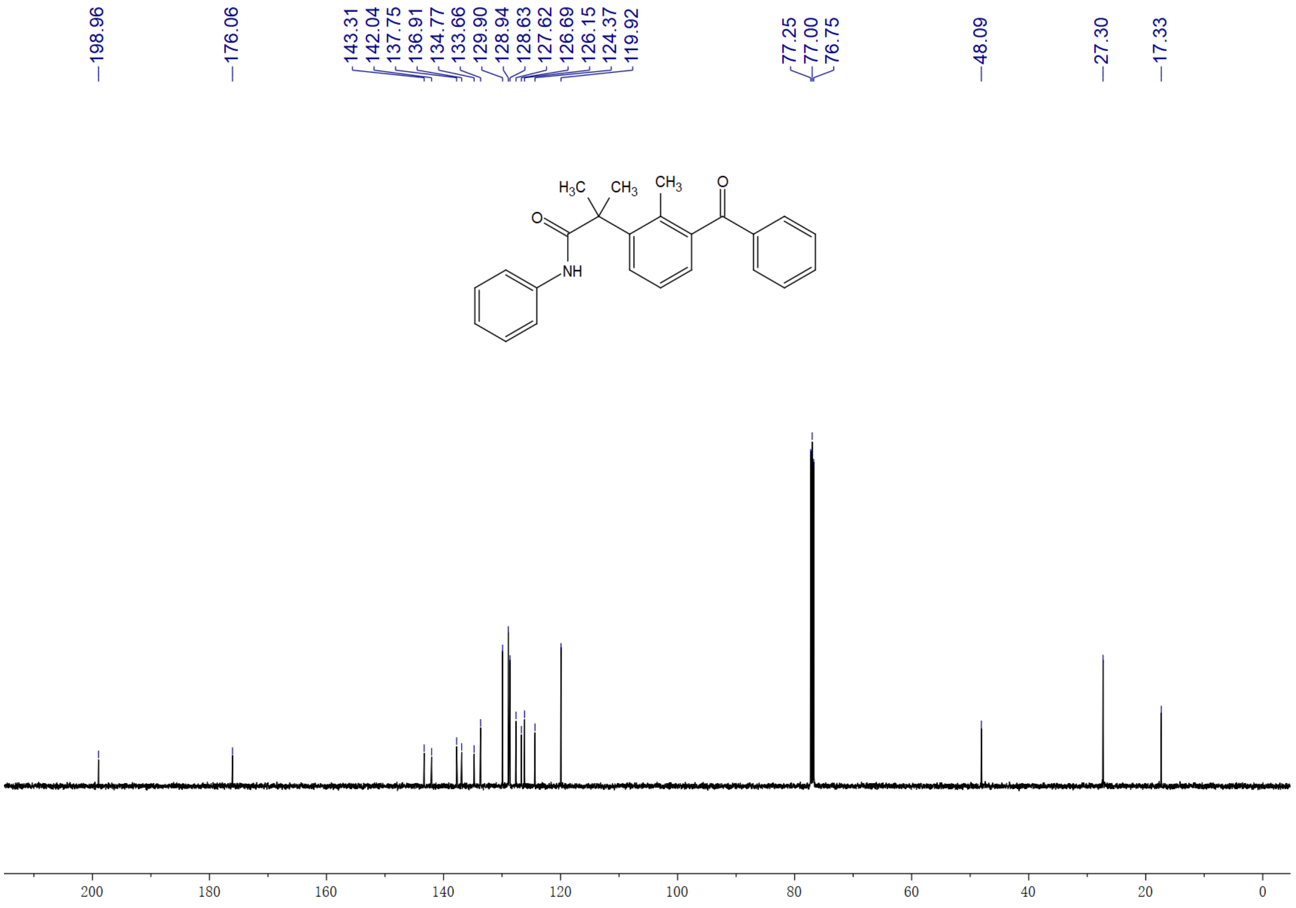

2-Methyl-2-(2-methyl-[1,1'-biphenyl]-3-yl)-N-phenylpropanamide, **6i'**, ^{1}H NMR (500 MHz, $CDCl_3$) and ^{13}C NMR (125 MHz, $CDCl_3$)

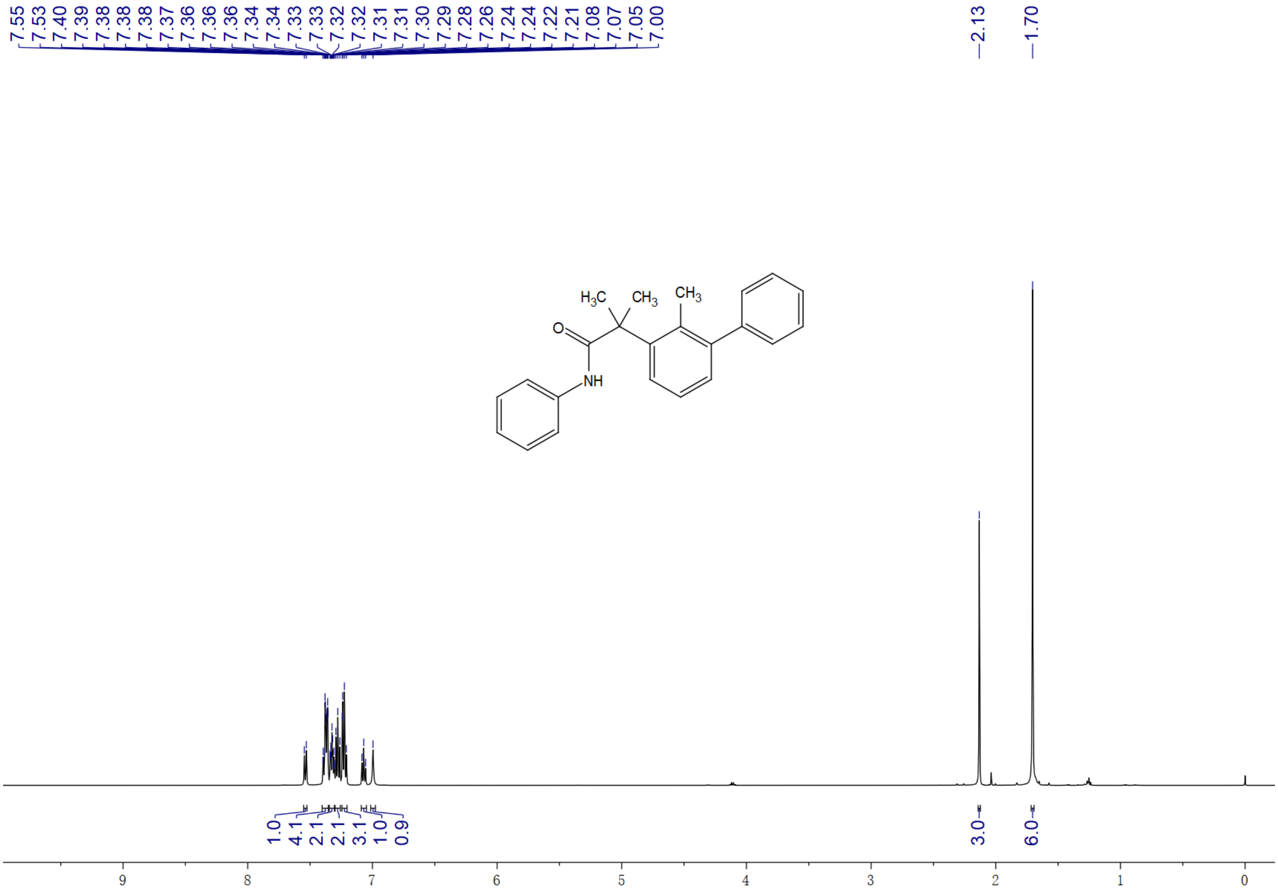

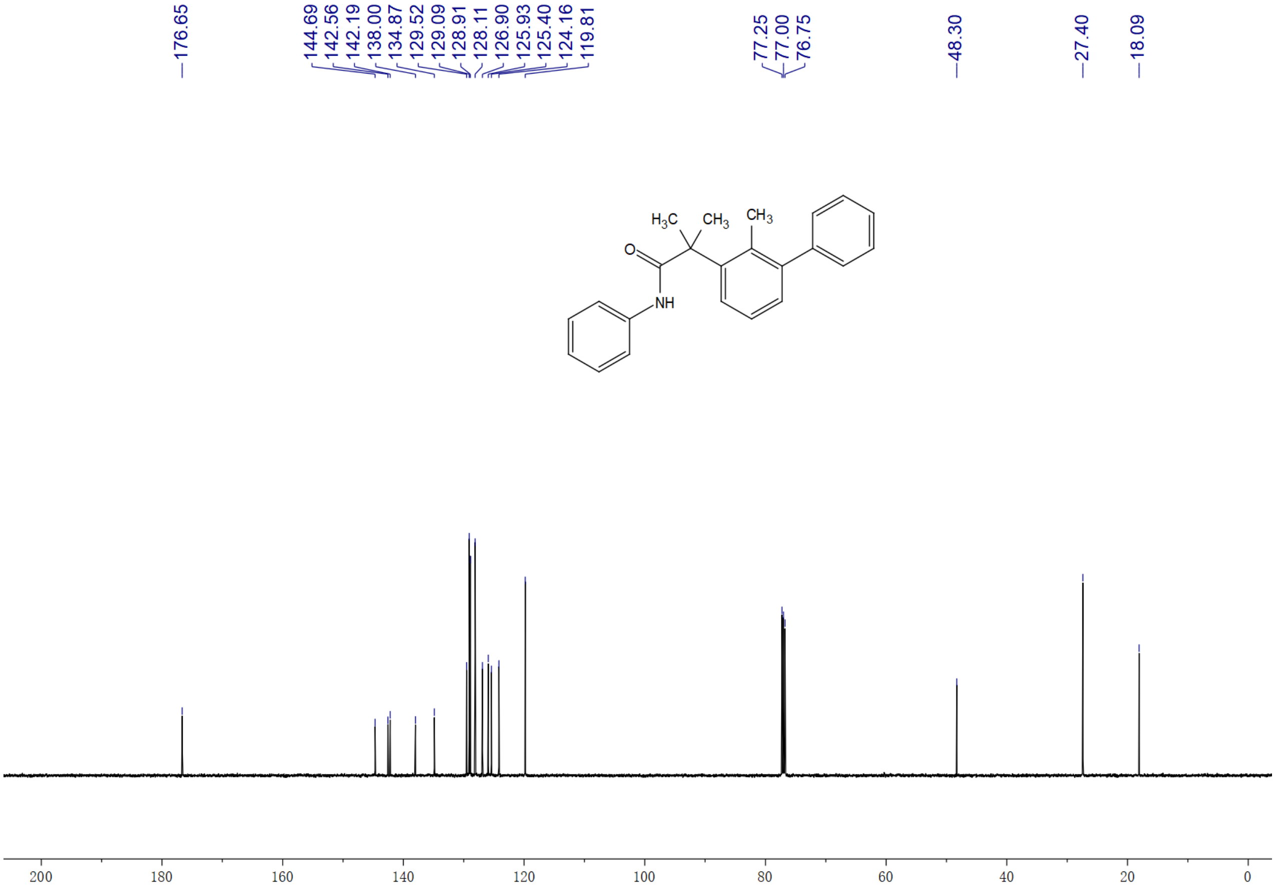

N-(1-(2,6-dimethylphenoxy)propan-2-yl)-2-methyl-3-(2-methyl-1-oxo-1-(phenylamino)propan-2-yl)benzamide, **6j**, ^{1}H NMR (500 MHz, $CDCl_3$) and ^{13}C NMR (125 MHz, $CDCl_3$)

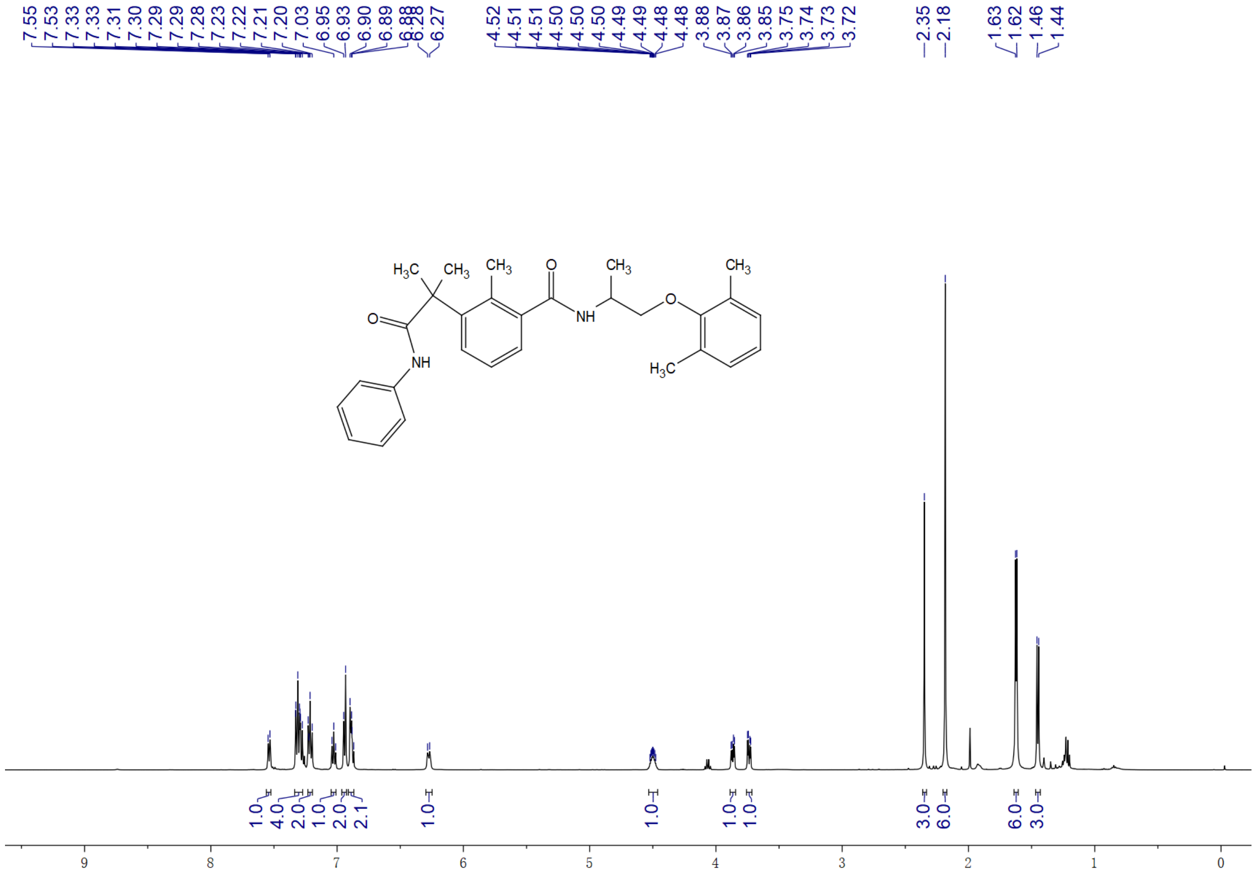

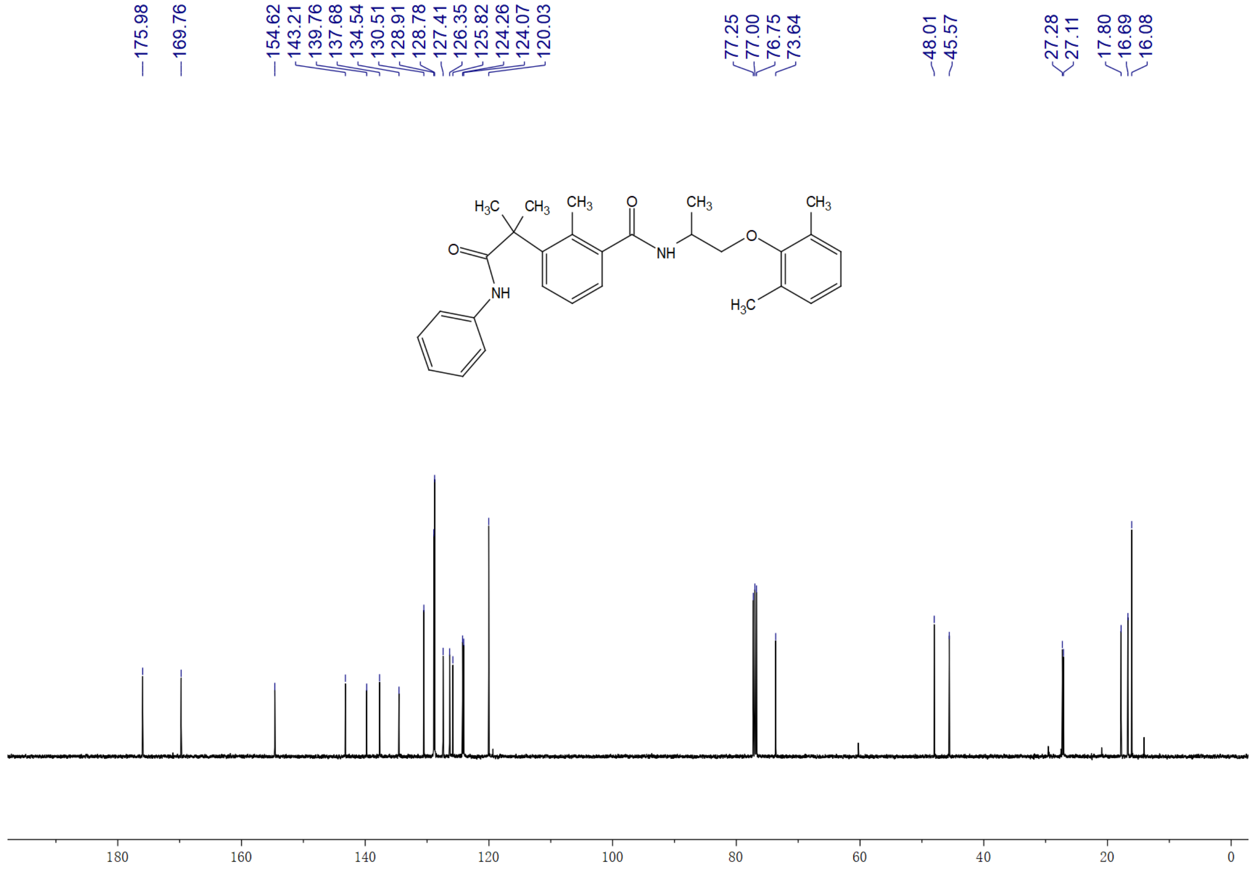

3-(Carboxydifluoromethyl)-2-methylbenzoic acid, **6k**, ^{1}H NMR (500 MHz, $CDCl_3$), ^{13}C NMR (125 MHz, $CDCl_3$) and ^{19}F NMR (471 MHz, $CDCl_3$)

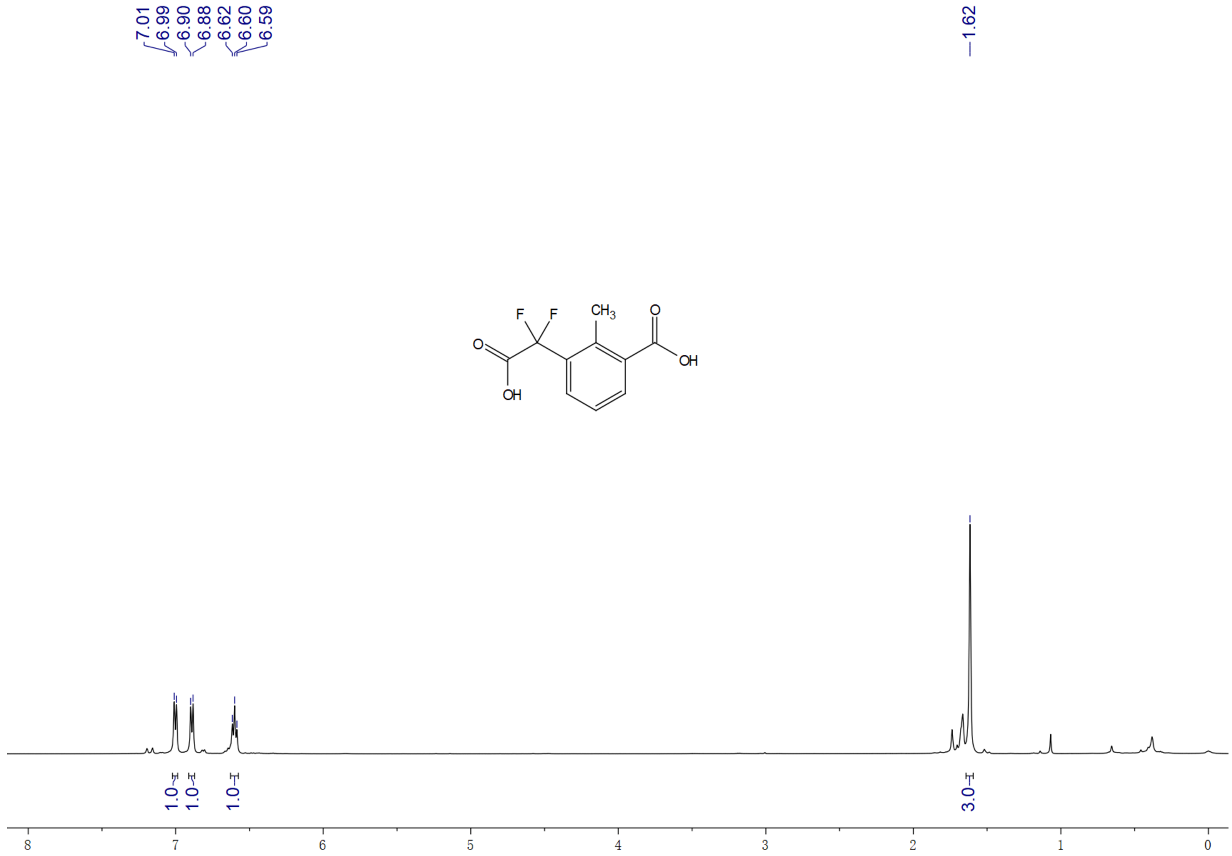

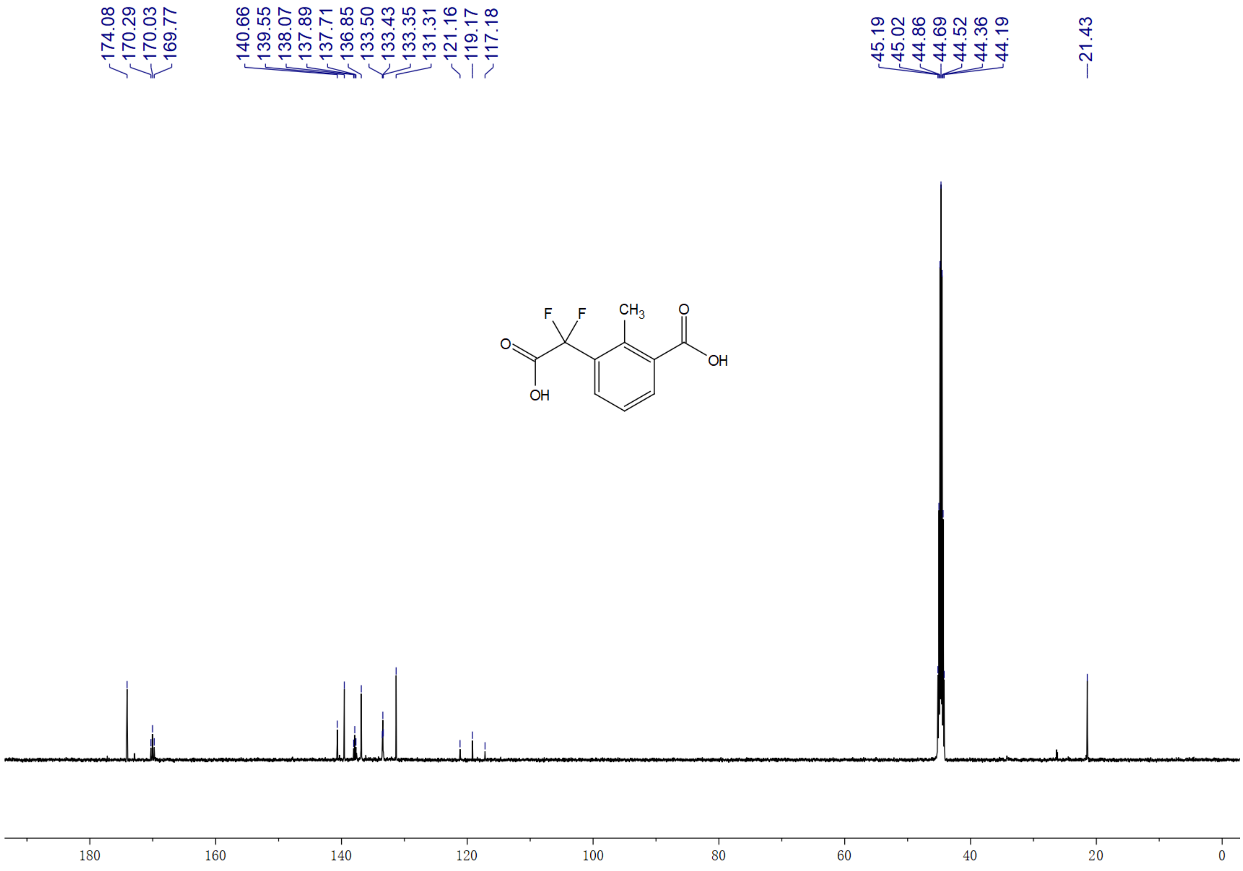

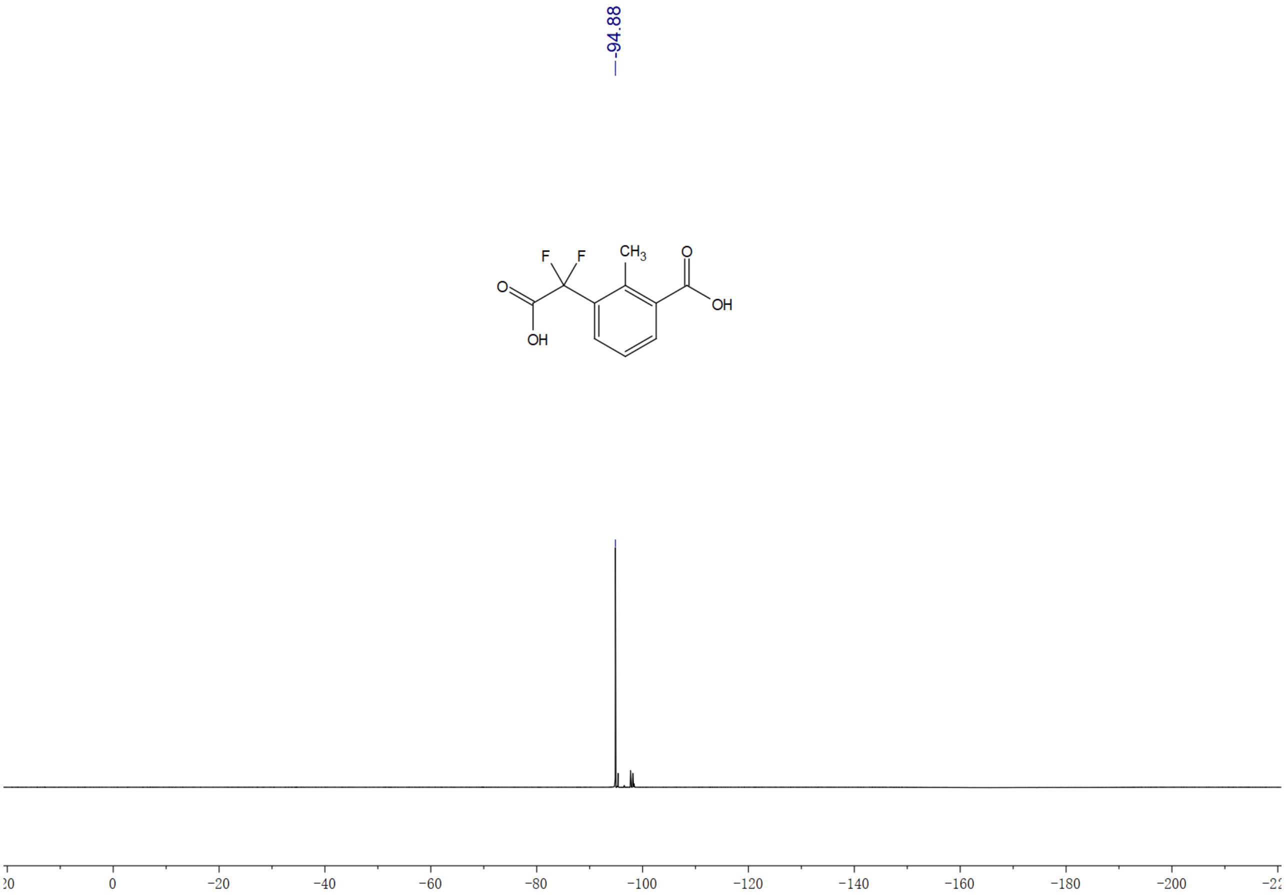
-94.88
F
F
CH3
O
O
OH
OH
20
0
−20
−40
−60
−80
−100
−120
−140
−160
−180
−200

Ru-A, ^{1}H NMR (400 MHz, $CDCl_3$) and ^{13}C NMR (100 MHz, $CDCl_3$)

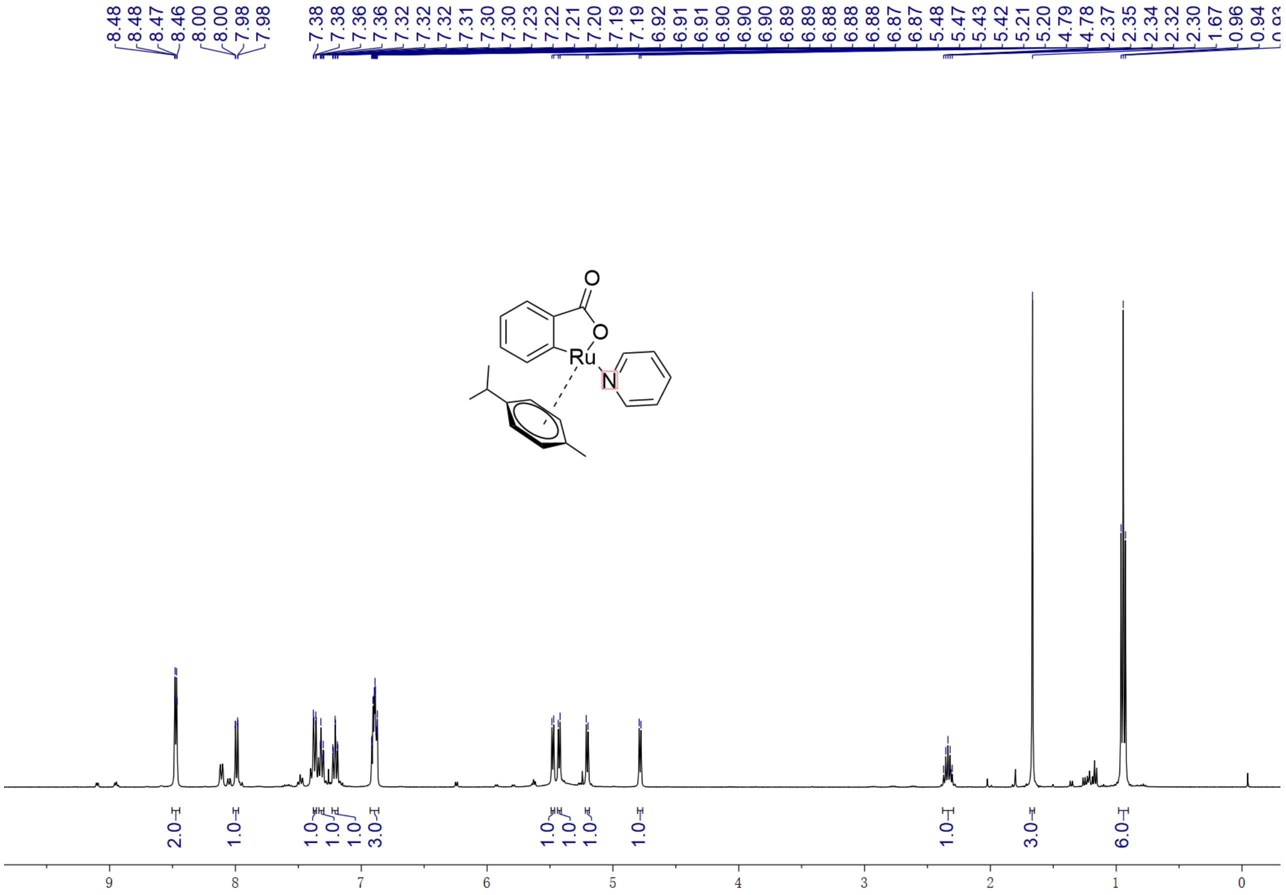

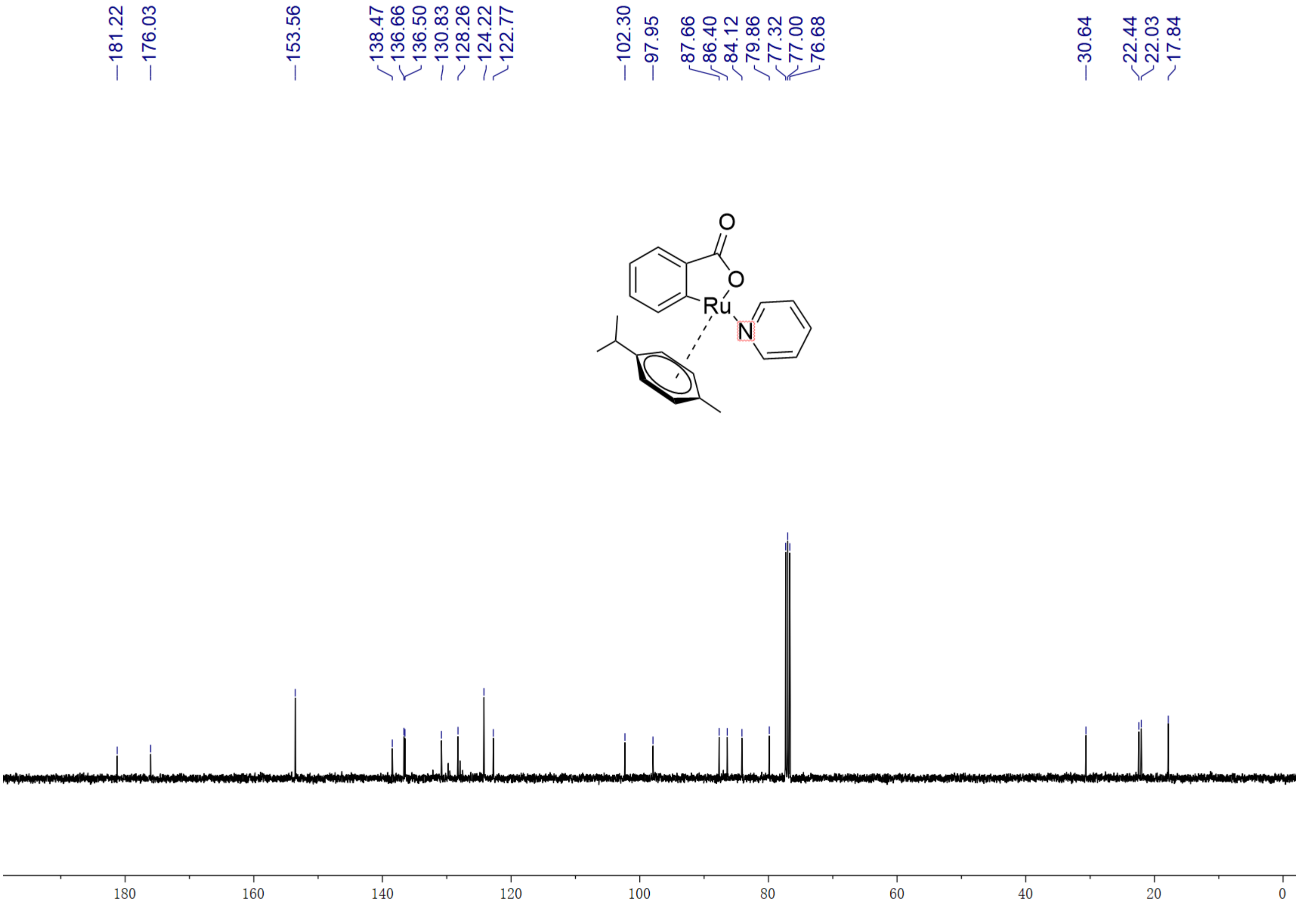

2-(2,6-Di-tert-butyl-4-methylphenoxy)-2-methyl-*N*-phenylpropanamide, **5aa**, ^{1}H NMR (500 MHz, $CDCl_3$) and ^{13}C NMR (125 MHz, $CDCl_3$)

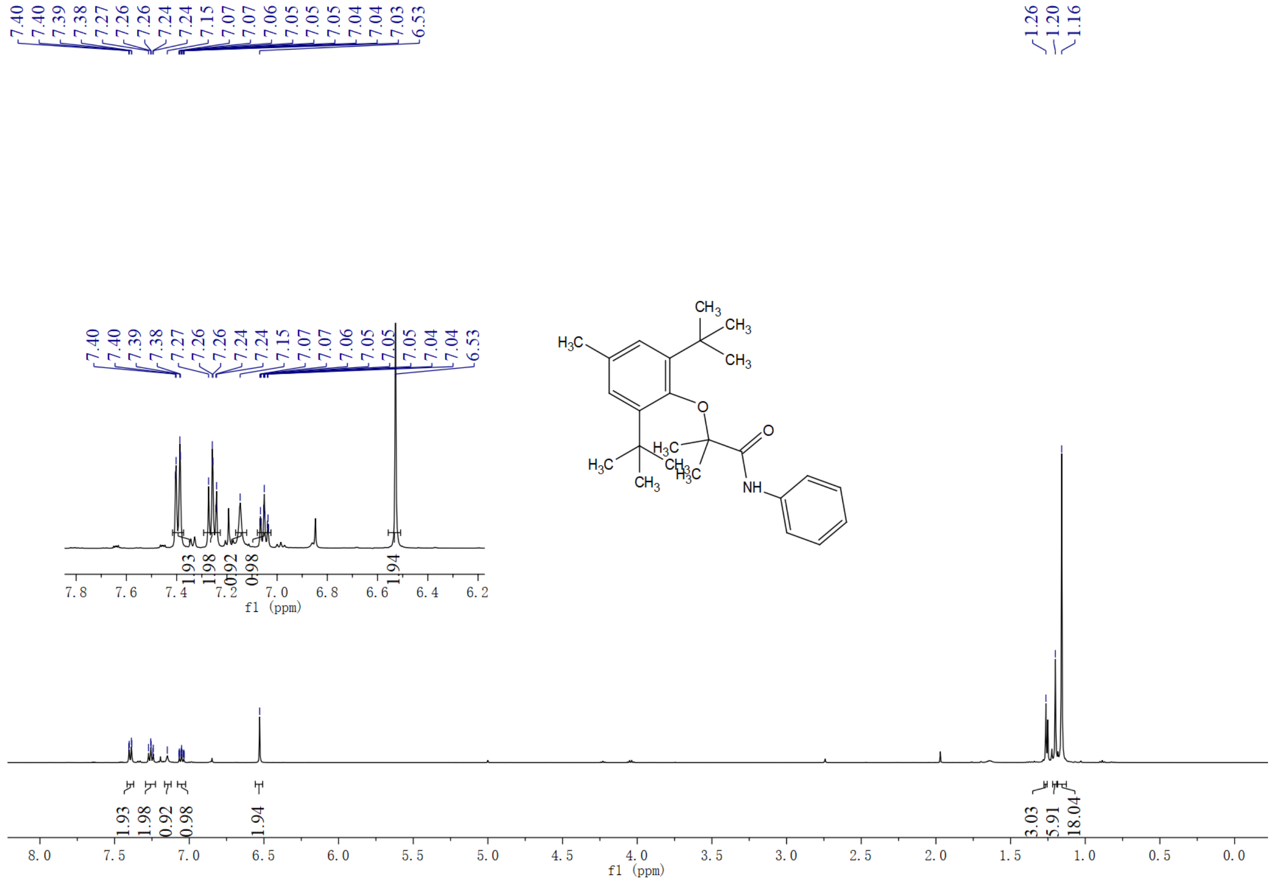

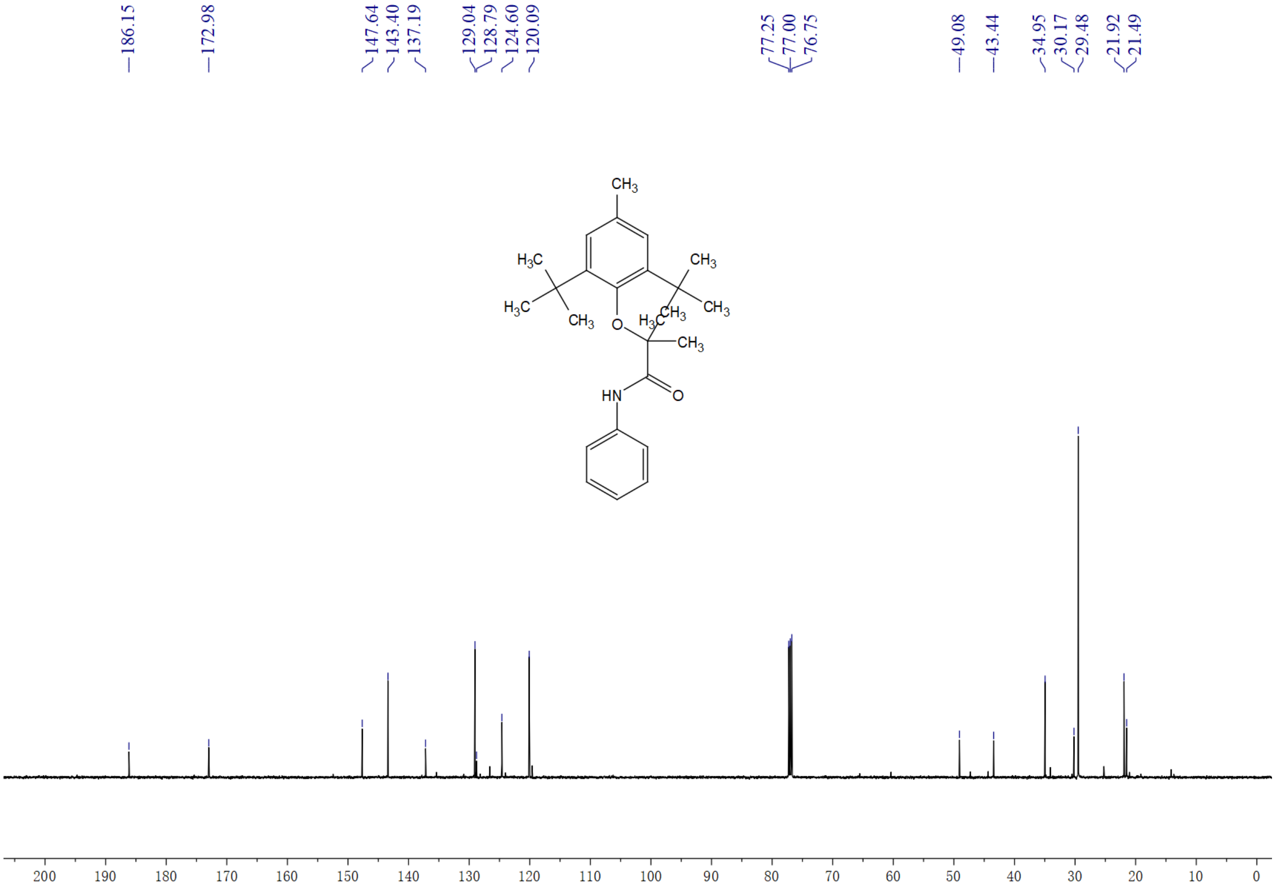

3,3-Dimethyl-1,5,5-triphenylpyrrolidin-2-one, **5ab**, ^{1}H NMR (500 MHz, $CDCl_3$) and ^{13}C NMR (125 MHz, $CDCl_3$)

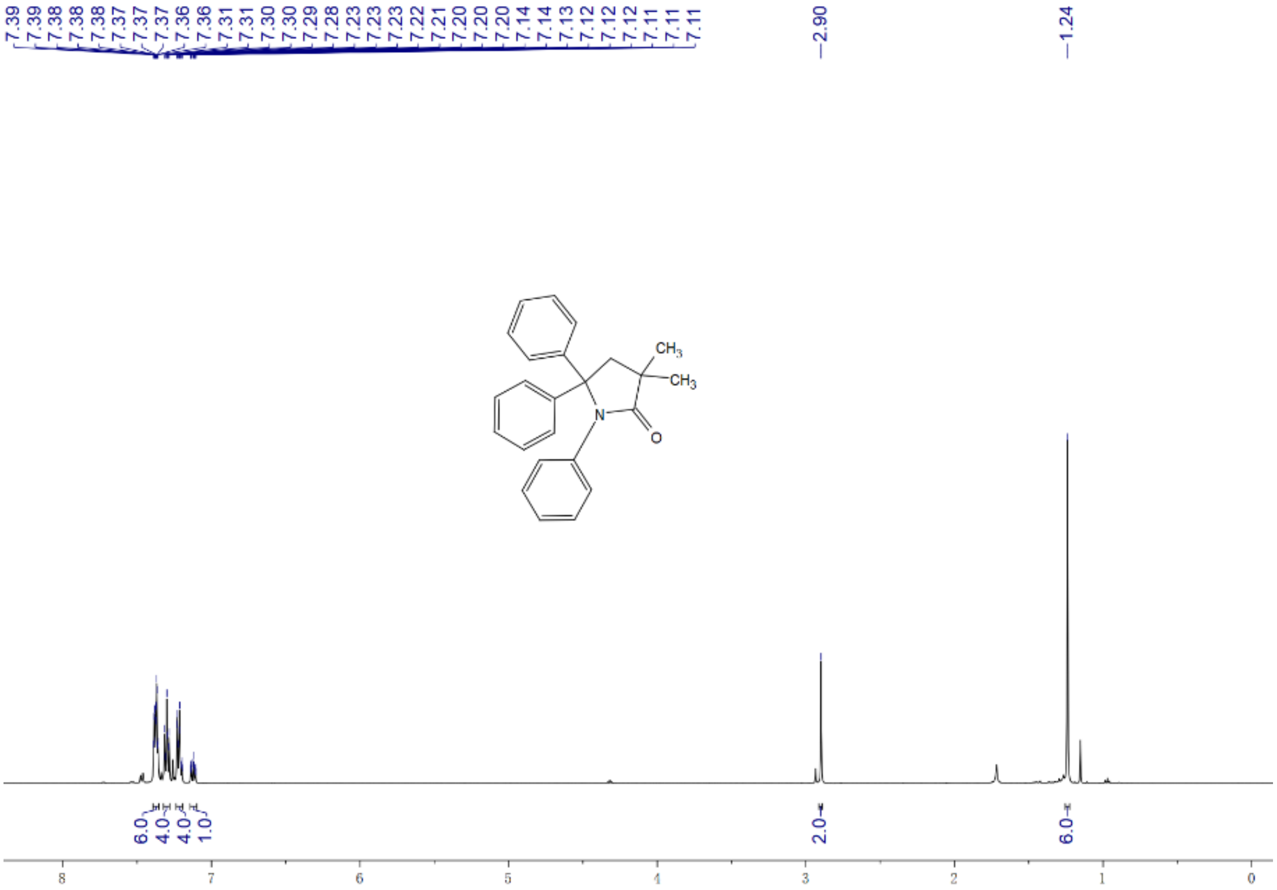

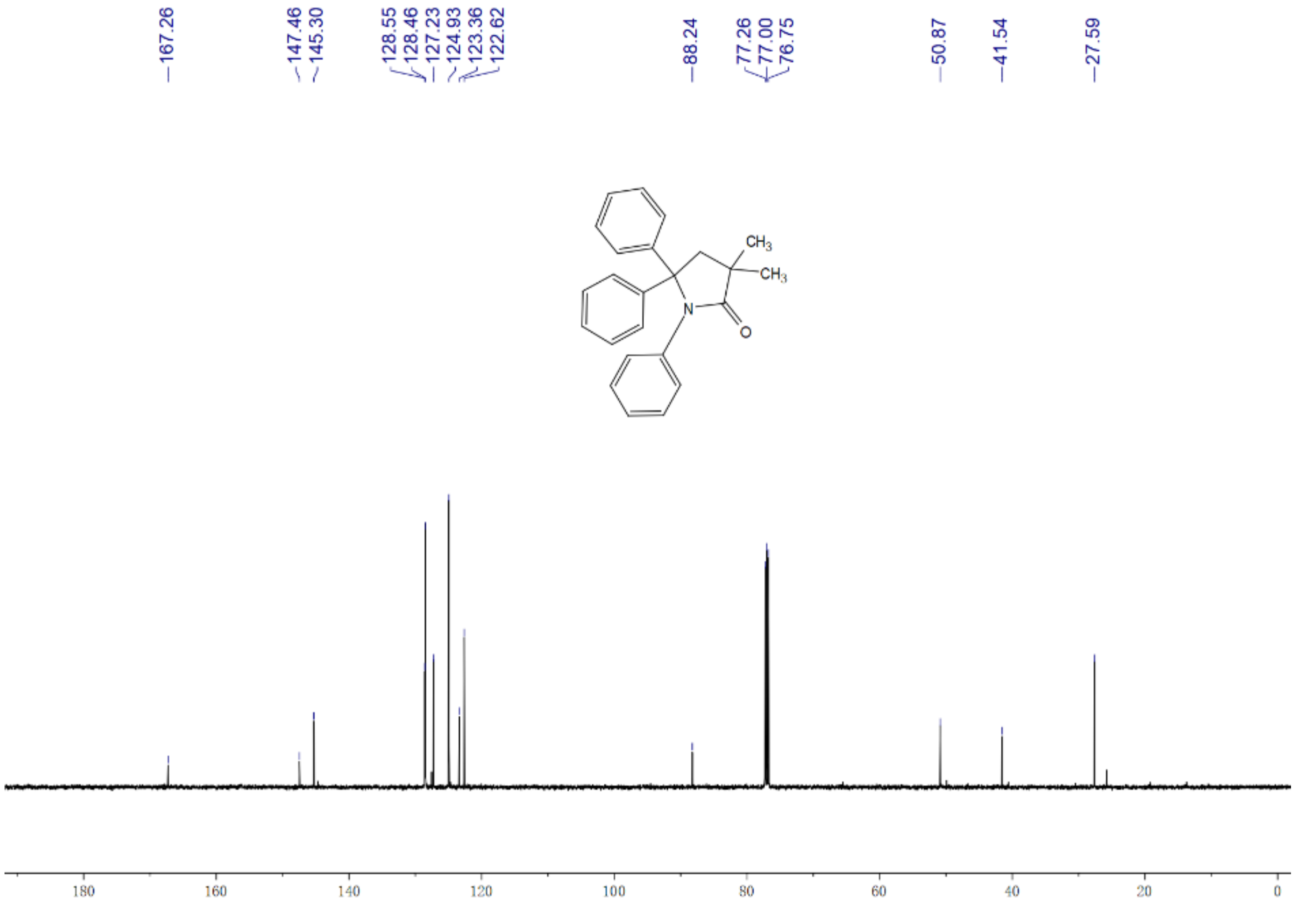

III. Supplementary References

1. Pan, W. et al. Highly 1,2 Regio- and Stereoselective Polymerization of 1,3-Butadiene Initiated by Iron Catalysts with Pyridinyl Phosphate. *Ind. Eng. Chem. Res.* **55**, 7580-7586 (2016).

2. Zhou, Z., Behnke, N. E. & Kurti, L. Copper-Catalyzed Synthesis of Hindered Ethers from alpha-Bromo Carbonyl Compounds. *Org. Lett.* **20**, 5452-5456 (2018).

3. Yamane, Y., Miwa, N. & Nishikata, T. Copper-Catalyzed Functionalized Tertiary-Alkylative Sonogashira Type Couplings via Copper Acetylide at Room Temperature. *ACS Catal.* **7**, 6872-6876 (2017).

4. Fu, Y. et al. Remote C5-Selective Functionalization of Naphthalene Enabled by P–Ru–C Bond-Directed δ-Activation. *ACS Catal.* **12**, 5036-5047 (2022).

5. Lv, X. et al. Visible-Light-Mediated Ruthenium-Catalyzed *para*-Selective Alkylation of Unprotected Anilines. *ACS Catal.* **13**, 7310-7321 (2023).

6. Ruan, Z. et al. Ruthenium(II)-Catalyzed *meta* C−H Mono- and Difluoromethylations by Phosphine/Carboxylate Cooperation. *Angew. Chem. Int. Ed.* **56**, 2045-2049 (2017).

7. Bao, Z.-P., Zhang, Y., Wang, L.-C. & Wu, X.-F. Difluoroalkylative carbonylation of alkenes to access carbonyl difluoro-containing heterocycles: convenient synthesis of gemigliptin. *Sci. China Chem.* **66**, 139-146 (2022).

8. Li, G., Li, D., Zhang, J., Shi, D.-Q. & Zhao, Y. Ligand-Enabled Regioselectivity in the Oxidative Cross-coupling of Arenes with Toluenes and Cycloalkanes Using Ruthenium Catalysts: Tuning the Site-Selectivity from the *ortho* to *meta* Positions. *ACS Catal.* **7**, 4138-4143 (2017).

9. Jing, K., Li, Z.-Y. & Wang, G.-W. Direct Decarboxylative *Meta*-Selective Acylation of Arenes via an *Ortho*-Ruthenation Strategy. *ACS Catal.* **8**, 11875-11881 (2018).

10. Saidi, O. et al. Ruthenium-Catalyzed *Meta* Sulfonation of 2-Phenylpyridines. *J. Am. Chem. Soc.* **133**, 19298-19301 (2011).

11. Fan, Z., Ni, J. & Zhang, A. *Meta*-Selective CAr–H Nitration of Arenes through a $Ru_3(CO)_{12}$-Catalyzed *Ortho*-Metalation Strategy. *J. Am. Chem. Soc.* **138**, 8470-8475 (2016).

12. Teskey, C. J., Lui, A. Y. W. & Greaney, M. F. Ruthenium‐Catalyzed *meta*‐Selective C-H Bromination. *Angew. Chem. Int. Ed.* **54**, 11677-11680 (2015).

13. Yu, Q., Hu, L. a., Wang, Y., Zheng, S. & Huang, J. Directed *meta*-Selective Bromination of Arenes with Ruthenium Catalysts. *Angew. Chem. Int. Ed.* **54**, 15284-15288 (2015).

14. Hofmann, N. & Ackermann, L. *meta*-Selective C–H Bond Alkylation with Secondary Alkyl Halides. *J. Am. Chem. Soc.* **135**, 5877-5884 (2013).

15. Li, J. et al. *N*-Acyl Amino Acid Ligands for Ruthenium(II)-Catalyzed *meta*-C–H tert-Alkylation with Removable Auxiliaries. *J. Am. Chem. Soc.* **137**, 13894-13901 (2015).

16. Warratz, S. et al. Ruthenium(II)-Catalyzed C-H Activation/Alkyne Annulation by Weak Coordination with O_2 as the Sole Oxidant. *Angew. Chem. Int. Ed.* **54**, 5513-5517 (2015).

17. Mei, R., Zhu, C. & Ackermann, L. Ruthenium(ii)-catalyzed C-H functionalizations on benzoic acids with aryl, alkenyl and alkynyl halides by weak-O-coordination. *Chem. Commun.* **52**, 13171-13174 (2016).

18. Han, W. J., Pu, F., Fan, J., Liu, Z. W. & Shi, X. Y. Rhodium(III)‐Catalyzed Tandem C−H Olefination and Oxidative Cyclization of Aromatic Acids with Acrylates for the Synthesis of (E)‐3‐Ylidenephthalides. *Adv. Syn. Catal.* **359**, 3520-3525 (2017).

19. Huang, L., Biafora, A., Zhang, G., Bragoni, V. & Gooßen, L. J. Regioselective C−H Hydroarylation of Internal Alkynes with Arenecarboxylates: Carboxylates as Deciduous Directing Groups. *Angew. Chem. Int. Ed.* **55**, 6933-6937 (2016).

20. He, Z. et al. A Strategy for Accessing Aldehydes via Palladium‐Catalyzed C−O/C−N Bond Cleavage in the Presence of Hydrosilanes. *Adv. Syn. Catal.* **362**, 5794-5800 (2020).

21. Xu, T. et al. Palladium-Catalyzed Decarbonylative Cyanation of Carboxylic Acids with TMSCN. *J. Org. Chem.* **87**, 11871-11879 (2022).

22. West, T. H. et al. Catalytic Enantioselective [2,3]-Rearrangements of Allylic Ammonium Ylides: A Mechanistic and Computational Study. *J. Am. Chem. Soc.* **139**, 4366-4375 (2017).

23. Chen, D. et al. Triflylpyridinium Enables Rapid and Scalable Controlled Reduction of Carboxylic Acids to Aldehydes using Pinacolborane. *Angew. Chem. Int. Ed.* **62**, e202215168 (2022).

24. Krishnamurti, V., Barrett, C., Ispizua-Rodriguez, X., Coe, M. & Prakash, G. K. S. Aqueous Base Promoted O-Difluoromethylation of Carboxylic Acids with $TMSCF_2Br$: Bench-Top Access to Difluoromethyl Esters. *Org. Lett.* **21**, 9377-9380 (2019).

25. Wu, H. et al. One-Pot Synthesis of Arylketones from Aromatic Acids via Palladium-Catalyzed Suzuki Coupling. *J. Org. Chem.* **81**, 2987-2992 (2016).

26. Li, X. & Zou, G. Acylative Suzuki coupling of amides: acyl-nitrogen activation via synergy of independently modifiable activating groups. *Chem. Commun.* **51**, 5089-5092 (2015).
